# Supplementary material for: In Utero Exposure to Caffeine and Acetaminophen, the Gut Microbiome, and Neurodevelopmental Outcomes: A Prospective Birth Cohort Study
Source: Int J Environ Res Public Health. 2022 Jul 30;19(15):9357. doi: 10.3390/ijerph19159357 (PMC9367926; doi:10.3390/ijerph19159357)
Supplement: Supplementary file 1 [file ijerph-19-09357-s001.zip › ijerph-1813294-supplementary.pdf]

Supplementary Online Content

Supplemental Methods.

Table S1. Associations of acetaminophen and caffeine with childhood bacterial diversity

Table S2. Associations of acetaminophen and caffeine exposure with bacterial phylum relative abundance

Table S3. Difference in bacterial species relative abundance comparing those exposed to acetaminophen to unexposed

Table S4. Difference in MetaCyc pathway relative abundance comparing acetaminophen exposed to unexposed

Table S5 Change in bacterial species relative abundance per doubling caffeine (ng/g)

Table S6. Change in MetaCyc pathway relative abundance per doubling caffeine (ng/g)

Table S7. Associations of acetaminophen and caffeine with beta diversity

Table S8. Acetaminophen-phylum interactions in association with neurodevelopmental outcomes

Table S9. Caffeine-phylum interactions in association with neurodevelopmental outcomes

Table S10. Caffeine-species interactions in association with neurodevelopment

Table S11. Acetaminophen-species interactions in associations with neurodevelopment

Table S12. Caffeine-pathway interactions in association with neurodevelopment

Table S13. Acetaminophen-pathway interactions in association with neurodevelopment

Supplemental Methods.

GESTation and Environment Cohort Description

The GESTation and Environment (GESTE) cohort recruited participants in early pregnancy and at the time of birth between 2007 and 2009. The cohort was designed to examine the associations between polybrominated diphenyl ether (PBDE) flame retardants and neurodevelopment,[1,2] with altered thyroid function as a proposed mechanism.[3] When children were 3 years old, their caregivers were mailed a neurodevelopmental assessment. At ages 6-7 years and 8-10 years, participants completed an in-person study visit, which included anthropometry measurements and neurodevelopmental assessment by a trained clinician. Throughout the study, there has been rich specimen collection including blood (pregnancy, birth, cord blood, and childhood), stool (meconium, childhood), urine (pregnancy, birth, childhood), and placenta, and thorough evaluation of sociodemographic and lifestyle factors.

Exposure assessment

Briefly, solid-liquid extraction was performed with ethyl acetate and purified with acetonitrile. Ultra-performance liquid chromatograph coupled with tandem mass spectrometry (UPLC-MS/MS) was used to detect concentrations in the extraction and these were normalized by the starting mass of meconium. The limit of detection (LOD) and limit of quantification (LOQ) for caffeine were 0.2ng/g and 0.4 ng/g, respectively; all concentrations were above the LOQ (minimum = 100 ng/g) thus no imputation was required. The LOD and LOQ for acetaminophen were 2ng/g and 5.6ng/g, respectively.

Covariates

Sociodemographic and lifestyle data were gathered from medical records and via questionnaires in early pregnancy, at delivery, and at follow-up visits. Child sex and mode of birth were determined from the medical record and dichotomized (male vs. female and vaginal vs. caesarean, respectively). Caregivers reported their child's breastfeeding history on questionnaires and we dichotomized their behavior as ever or never breastfed. Missing breastfeeding behavior (n=3) was imputed as the population mode (ever breastfed). Caregivers reported family income at delivery via questionnaire. Missing values were imp[1]uted with non-missing income at the 6-8-year-old follow-up (n=12) or with the population mean (n=1). Similarly, missing income were imputed with non-missing income at delivery (n=4) or with the population mean (n=2). Maternal IQ was assessed using Raven Matrices at the 6-8-year-old follow-up visit.[4] Percentiles were calculated in comparison to the normative population and were categorized as $>95^{th}$ percentile or $\leq 95^{th}$ percentile to provide roughly equal numbers in each of the categories.

REFERENCES

1. Solazzo G, Wu H, Laue HE, et al. The association between prenatal concentrations of polybrominated diphenyl ether and child cognitive and psychomotor function. *Environ Epidemiol*. Jun 2021;5(3):e156. doi:10.1097/ee9.0000000000000156
2. Sussman TJ, Baker BH, Wakhloo AJ, et al. The relationship between persistent organic pollutants and Attention Deficit Hyperactivity Disorder phenotypes: Evidence from task-based neural activity in an observational study of a community sample of Canadian mother-child dyads. *Environ Res*. Apr 15 2022;206:112593. doi:10.1016/j.envres.2021.112593
3. Abdelouahab N, Langlois MF, Lavoie L, Corbin F, Pasquier JC, Takser L. Maternal and cord-blood thyroid hormone levels and exposure to polybrominated diphenyl ethers and polychlorinated biphenyls during early pregnancy. *Am J Epidemiol*. Sep 1 2013;178(5):701-13. doi:10.1093/aje/kwt141
4. Raven J, Raven J. Raven Progressive Matrices. *Handbook of nonverbal assessment*. Kluwer Academic/Plenum Publishers; 2003:223-237.

Table S1. Associations of acetaminophen and caffeine with childhood bacterial diversity

Method	Exposure	Exposure Window	Effect Estimate ± Std. Error (95% CI)	p-value	FDR q-value
Shannon	Caffeine	Meconium	-0.059 ± 0.076 (-0.208, 0.089)	0.440	0.440
Pielou	Caffeine	Meconium	-0.077 ± 0.075 (-0.224, 0.071)	0.313	0.417
Shannon	Acetaminophen	Meconium	-0.173 ± 0.07 (-0.311, -0.035)	0.018	0.044
Pielou	Acetaminophen	Meconium	-0.035 ± 0.015 (-0.063, -0.006)	0.022	0.044
Shannon	Caffeine	Cross-sectional	-0.057 ± 0.045 (-0.145, 0.032)	0.212	0.565
Pielou	Caffeine	Cross-sectional	-0.049 ± 0.045 (-0.137, 0.039)	0.282	0.565
Shannon	Acetaminophen	Cross-sectional	-0.016 ± 0.103 (-0.217, 0.185)	0.880	0.880
Pielou	Acetaminophen	Cross-sectional	-0.007 ± 0.022 (-0.05, 0.036)	0.749	0.880
Shannon	Caffeine	Meconium Adj.[a]	-0.015 ± 0.018 (-0.051, 0.021)	0.431	0.431
Pielou	Caffeine	Meconium Adj.[a]	-0.004 ± 0.004 (-0.011, 0.004)	0.329	0.431
Shannon	Acetaminophen	Meconium Adj.[a]	-0.153 ± 0.071 (-0.292, -0.015)	0.036	0.074
Pielou	Acetaminophen	Meconium Adj.[a]	-0.032 ± 0.015 (-0.061, -0.003)	0.037	0.074

[a]Meconium Adj. includes cross-sectional exposure in the model

Table S2. Associations of acetaminophen and caffeine exposure with bacterial phylum relative abundance

Exposure	Exposure Window	Phylum	Effect Estimate[a]	Std. Error	p-value	q-value
Acetaminophen	Meconium	Firmicutes	-0.094	0.035	0.010	0.062
Acetaminophen	Meconium	Actinobacteria	0.183	0.081	0.028	0.085
Acetaminophen	Meconium	Verrucomicrobia	-0.939	0.479	0.056	0.113
Acetaminophen	Meconium	Euryarchaeota	-0.654	0.580	0.266	0.399
Acetaminophen	Meconium	Bacteroidetes	-0.058	0.206	0.779	0.779
Acetaminophen	Meconium	Proteobacteria	0.228	0.304	0.457	0.548
Caffeine	Meconium	Euryarchaeota	-0.101	0.292	0.730	0.876
Caffeine	Meconium	Actinobacteria	0.051	0.042	0.230	0.509
Caffeine	Meconium	Bacteroidetes	0.014	0.102	0.888	0.888
Caffeine	Meconium	Firmicutes	-0.035	0.018	0.058	0.350
Caffeine	Meconium	Proteobacteria	0.080	0.151	0.598	0.876
Caffeine	Meconium	Verrucomicrobia	-0.282	0.244	0.254	0.509
Acetaminophen	Cross-sectional	Verrucomicrobia	-1.555	0.749	0.041	0.248
Acetaminophen	Cross-sectional	Euryarchaeota	-0.338	0.753	0.655	0.782
Acetaminophen	Cross-sectional	Actinobacteria	-0.098	0.110	0.378	0.767
Acetaminophen	Cross-sectional	Bacteroidetes	-0.167	0.253	0.511	0.767
Acetaminophen	Cross-sectional	Firmicutes	0.032	0.046	0.495	0.767
Acetaminophen	Cross-sectional	Proteobacteria	0.128	0.460	0.782	0.782
Caffeine	Cross-sectional	Euryarchaeota	-0.287	0.210	0.175	0.540
Caffeine	Cross-sectional	Actinobacteria	0.024	0.031	0.450	0.540
Caffeine	Cross-sectional	Bacteroidetes	0.058	0.071	0.415	0.540
Caffeine	Cross-sectional	Proteobacteria	-0.104	0.129	0.424	0.540
Caffeine	Cross-sectional	Verrucomicrobia	-0.271	0.214	0.210	0.540
Caffeine	Cross-sectional	Firmicutes	-0.007	0.013	0.575	0.575
Acetaminophen	Meconium Adj.[b]	Firmicutes	-0.105	0.035	0.005	0.027
Acetaminophen	Meconium Adj.[b]	Actinobacteria	0.213	0.080	0.011	0.032
Acetaminophen	Meconium Adj.[b]	Verrucomicrobia	-0.945	0.494	0.062	0.125
Acetaminophen	Meconium Adj.[b]	Euryarchaeota	-0.527	0.589	0.376	0.466
Acetaminophen	Meconium Adj.[b]	Proteobacteria	0.271	0.312	0.389	0.466
Acetaminophen	Meconium Adj.[b]	Bacteroidetes	0.023	0.201	0.909	0.909
Caffeine	Meconium Adj.[b]	Euryarchaeota	-0.123	0.292	0.675	0.810
Caffeine	Meconium Adj.[b]	Actinobacteria	0.051	0.042	0.235	0.521
Caffeine	Meconium Adj.[b]	Bacteroidetes	0.017	0.103	0.872	0.872
Caffeine	Meconium Adj.[b]	Firmicutes	-0.034	0.018	0.065	0.393
Caffeine	Meconium Adj.[b]	Proteobacteria	0.074	0.153	0.631	0.810
Caffeine	Meconium Adj.[b]	Verrucomicrobia	-0.283	0.248	0.260	0.521

[a]Effect estimate for acetaminophen is difference in phylum relative abundance between exposed and unexposed. Effect estimate for caffeine is difference in phylum relative abundance per doubling of caffeine (ng/g). Models are adjusted for whether the child was ever breastfed, sex, mode of birth, and socioeconomic status.
[b]Meconium Adj. includes cross-sectional exposure in the model

Table S3. Difference in bacterial species relative abundance comparing those exposed to acetaminophen to unexposed

Exposure Window	Species	Effect Estimate[a]	Std. Error	p-value	q-value
Meconium	Methanobrevibacter smithii	-0.118	0.119	0.325	0.967
Meconium	Actinobaculum sp oral taxon 183	0.471	0.224	0.041	0.967
Meconium	Actinomyces graevenitzii	0.083	0.182	0.651	0.967
Meconium	Actinomyces johnsonii	0.240	0.261	0.363	0.967
Meconium	Actinomyces massiliensis	-0.051	0.136	0.710	0.967
Meconium	Actinomyces naeslundii	-0.223	0.205	0.282	0.967
Meconium	Actinomyces odontolyticus	-0.052	0.198	0.795	0.967
Meconium	Actinomyces oris	-0.080	0.227	0.727	0.967
Meconium	Actinomyces sp HMSC035G02	0.235	0.185	0.211	0.967
Meconium	Actinomyces sp ICM47	-0.056	0.291	0.849	0.967
Meconium	Actinomyces sp S6 Spd3	0.076	0.090	0.399	0.967
Meconium	Actinomyces sp oral taxon 181	-0.135	0.102	0.192	0.967
Meconium	Actinomyces turicensis	-0.113	0.134	0.406	0.967
Meconium	Bifidobacterium adolescentis	-0.145	0.356	0.687	0.967
Meconium	Bifidobacterium animalis	-0.369	0.270	0.179	0.967
Meconium	Bifidobacterium bifidum	0.320	0.280	0.260	0.967
Meconium	Bifidobacterium catenulatum	-0.101	0.340	0.768	0.967
Meconium	Bifidobacterium longum	0.143	0.153	0.355	0.967
Meconium	Bifidobacterium pseudocatenulatum	0.778	0.368	0.040	0.967
Meconium	Corynebacterium durum	0.152	0.132	0.255	0.967
Meconium	Rothia mucilaginosa	-0.262	0.235	0.270	0.967
Meconium	Collinsella aerofaciens	0.161	0.201	0.429	0.967
Meconium	Collinsella intestinalis	0.076	0.125	0.547	0.967
Meconium	Collinsella stercoris	0.071	0.246	0.776	0.967
Meconium	Enorma massiliensis	-0.063	0.071	0.380	0.967
Meconium	.Collinsella. massiliensis	0.094	0.095	0.328	0.967
Meconium	Adlercreutzia equolifaciens	-0.185	0.136	0.182	0.967
Meconium	Asaccharobacter celatus	-0.065	0.128	0.611	0.967
Meconium	Eggerthella lenta	-0.071	0.197	0.720	0.967
Meconium	Enterorhabdus caecimuris	-0.192	0.221	0.389	0.967
Meconium	Gordonibacter pamelaeae	-0.205	0.151	0.183	0.967
Meconium	Slackia isoflavoniconvertens	0.071	0.229	0.759	0.967
Meconium	Bacteroides caccae	0.150	0.312	0.633	0.967
Meconium	Bacteroides cellulosilyticus	-0.097	0.225	0.669	0.967
Meconium	Bacteroides coprocola	0.098	0.086	0.259	0.967
Meconium	Bacteroides dorei	-0.027	0.347	0.939	0.967
Meconium	Bacteroides faecis	-0.148	0.221	0.507	0.967
Meconium	Bacteroides finegoldii	-0.088	0.133	0.512	0.967
Meconium	Bacteroides fragilis	-0.272	0.413	0.514	0.967
Meconium	Bacteroides galacturonicus	-0.132	0.133	0.328	0.967
Meconium	Bacteroides intestinalis	-0.174	0.122	0.161	0.967
Meconium	Bacteroides massiliensis	0.111	0.162	0.496	0.967
Meconium	Bacteroides ovatus	-0.157	0.301	0.604	0.967
Meconium	Bacteroides salyersiae	-0.067	0.135	0.621	0.967
Meconium	Bacteroides stercoris	0.311	0.256	0.231	0.967
Meconium	Bacteroides thetaiotaomicron	-0.125	0.294	0.673	0.967
Meconium	Bacteroides uniformis	-0.038	0.252	0.880	0.967
Meconium	Bacteroides vulgatus	-0.218	0.313	0.490	0.967
Meconium	Bacteroides xylanisolvens	-0.092	0.262	0.727	0.967

Exposure Window	Species	Effect Estimate[a]	Std. Error	p-value	q-value
Meconium	Odoribacter splanchnicus	-0.187	0.250	0.460	0.967
Meconium	Paraprevotella xylaniphila	-0.198	0.114	0.090	0.967
Meconium	Prevotella copri	0.015	0.208	0.943	0.967
Meconium	Alistipes finegoldii	0.095	0.315	0.764	0.967
Meconium	Alistipes indistinctus	-0.295	0.255	0.254	0.967
Meconium	Alistipes putredinis	-0.057	0.318	0.858	0.967
Meconium	Alistipes shahii	-0.236	0.309	0.449	0.967
Meconium	Parabacteroides distasonis	-0.042	0.333	0.900	0.967
Meconium	Parabacteroides merdae	-0.360	0.349	0.308	0.967
Meconium	Gemella sanguinis	0.045	0.110	0.680	0.967
Meconium	Lactobacillus rhamnosus	-0.121	0.148	0.417	0.967
Meconium	Lactobacillus rogosae	-0.044	0.151	0.772	0.967
Meconium	Lactococcus lactis	-0.289	0.279	0.305	0.967
Meconium	Streptococcus australis	0.018	0.232	0.938	0.967
Meconium	Streptococcus infantis	0.012	0.102	0.910	0.967
Meconium	Streptococcus mitis	-0.207	0.254	0.420	0.967
Meconium	Streptococcus parasanguinis	0.041	0.280	0.884	0.967
Meconium	Streptococcus salivarius	0.182	0.184	0.327	0.967
Meconium	Streptococcus sanguinis	-0.200	0.150	0.190	0.967
Meconium	Streptococcus sp A12	-0.073	0.203	0.720	0.967
Meconium	Streptococcus sp F0442	0.105	0.150	0.488	0.967
Meconium	Streptococcus thermophilus	-0.040	0.334	0.904	0.967
Meconium	Catabacter hongkongensis	-0.218	0.291	0.458	0.967
Meconium	Christensenella minuta	-0.123	0.139	0.380	0.967
Meconium	Clostridium bolteae CAG 59	-0.077	0.168	0.648	0.967
Meconium	Clostridium disporicum	-0.574	0.373	0.131	0.967
Meconium	Clostridium sp CAG 167	0.075	0.212	0.725	0.967
Meconium	Clostridium sp CAG 58	0.047	0.193	0.809	0.967
Meconium	Clostridium ventriculi	-0.212	0.202	0.300	0.967
Meconium	Hungatella hathewayi	0.033	0.316	0.918	0.967
Meconium	Eubacterium sulci	-0.259	0.217	0.240	0.967
Meconium	Mogibacterium diversum	0.066	0.143	0.647	0.967
Meconium	Intestinimonas butyriciproducens	-0.061	0.198	0.761	0.967
Meconium	Lawsonibacter asaccharolyticus	0.068	0.173	0.696	0.967
Meconium	Monoglobus pectinilyticus	-0.483	0.343	0.166	0.967
Meconium	Anaerofustis stercorihominis	-0.095	0.093	0.311	0.967
Meconium	Eubacterium callanderi	0.008	0.110	0.945	0.967
Meconium	Eubacterium eligens	-0.368	0.331	0.273	0.967
Meconium	Eubacterium hallii	-0.051	0.087	0.560	0.967
Meconium	Eubacterium limosum	-0.119	0.106	0.270	0.967
Meconium	Eubacterium ramulus	-0.150	0.208	0.475	0.967
Meconium	Eubacterium sp CAG 180	0.051	0.363	0.890	0.967
Meconium	Eubacterium sp CAG 38	-0.086	0.315	0.785	0.967
Meconium	Eubacterium ventriosum	-0.375	0.270	0.171	0.967
Meconium	Anaerostipes hadrus	-0.012	0.094	0.899	0.967
Meconium	Blautia hydrogenotrophica	-0.063	0.179	0.725	0.967
Meconium	Blautia obeum	-0.050	0.156	0.750	0.967
Meconium	Blautia producta	-0.202	0.212	0.346	0.967
Meconium	Blautia sp CAG 257	-0.158	0.125	0.215	0.967
Meconium	Blautia wexlerae	0.065	0.169	0.700	0.967
Meconium	Ruminococcus gnavus	-0.446	0.349	0.207	0.967
Meconium	Ruminococcus torques	-0.084	0.125	0.506	0.967

Exposure Window	Species	Effect Estimate[a]	Std. Error	p-value	q-value
Meconium	Coprococcus catus	-0.046	0.208	0.824	0.967
Meconium	Coprococcus comes	-0.401	0.408	0.331	0.967
Meconium	Coprococcus eutactus	-0.031	0.297	0.916	0.967
Meconium	Dorea formicigenerans	0.143	0.121	0.244	0.967
Meconium	Dorea longicatena	-0.076	0.163	0.642	0.967
Meconium	Dorea sp CAG 317	-0.044	0.158	0.784	0.967
Meconium	Eisenbergiella massiliensis	0.269	0.227	0.243	0.967
Meconium	Eisenbergiella tayi	0.125	0.252	0.623	0.967
Meconium	Fusicatenibacter saccharivorans	-0.131	0.125	0.302	0.967
Meconium	Clostridium bolteae	-0.038	0.190	0.842	0.967
Meconium	Clostridium scindens	0.037	0.283	0.897	0.967
Meconium	Clostridium symbiosum	-0.115	0.095	0.232	0.967
Meconium	Eubacterium rectale	-0.079	0.402	0.845	0.967
Meconium	Lachnospira pectinoschiza	-0.128	0.270	0.636	0.967
Meconium	Roseburia faecis	-0.169	0.293	0.568	0.967
Meconium	Roseburia hominis	0.154	0.318	0.631	0.967
Meconium	Roseburia intestinalis	-0.379	0.375	0.318	0.967
Meconium	Roseburia inulinivorans	-0.132	0.282	0.642	0.967
Meconium	Roseburia sp CAG 182	0.359	0.257	0.170	0.967
Meconium	Roseburia sp CAG 309	-0.237	0.146	0.111	0.967
Meconium	Roseburia sp CAG 471	0.058	0.264	0.827	0.967
Meconium	Sellimonas intestinalis	-0.318	0.361	0.384	0.967
Meconium	Oscillibacter sp 57 20	0.074	0.241	0.761	0.967
Meconium	Oscillibacter sp CAG 241	0.324	0.325	0.324	0.967
Meconium	Intestinibacter bartlettii	-0.250	0.111	0.030	0.967
Meconium	Romboutsia ilealis	0.150	0.245	0.544	0.967
Meconium	Agathobaculum butyriciproducens	-0.148	0.395	0.710	0.967
Meconium	Anaerotruncus colihominis	-0.023	0.106	0.830	0.967
Meconium	Anaerotruncus sp CAG 528	-0.187	0.117	0.118	0.967
Meconium	Faecalibacterium prausnitzii	0.012	0.189	0.949	0.967
Meconium	Flavonifractor plautii	0.080	0.377	0.832	0.967
Meconium	Gemmiger formicilis	0.031	0.248	0.902	0.967
Meconium	Clostridium leptum	-0.101	0.224	0.654	0.967
Meconium	Eubacterium siraeum	-0.465	0.255	0.075	0.967
Meconium	Ruminococcus bicirculans	-0.122	0.324	0.709	0.967
Meconium	Ruminococcus bromii	-0.117	0.262	0.657	0.967
Meconium	Ruminococcus callidus	-0.147	0.235	0.536	0.967
Meconium	Ruminococcus lactaris	-0.418	0.294	0.163	0.967
Meconium	Ruthenibacterium lactatiformans	0.346	0.182	0.064	0.967
Meconium	Coprobacillus cateniformis	0.145	0.266	0.589	0.967
Meconium	Clostridium innocuum	0.082	0.329	0.804	0.967
Meconium	Clostridium spiroforme	-0.153	0.341	0.657	0.967
Meconium	Erysipelatoclostridium ramosum	-0.258	0.247	0.301	0.967
Meconium	Holdemania filiformis	-0.025	0.088	0.779	0.967
Meconium	Turicibacter sanguinis	-0.078	0.298	0.796	0.967
Meconium	Firmicutes bacterium CAG 110	-0.040	0.436	0.927	0.967
Meconium	Firmicutes bacterium CAG 145	-0.245	0.352	0.491	0.967
Meconium	Firmicutes bacterium CAG 83	0.258	0.290	0.380	0.967
Meconium	Firmicutes bacterium CAG 94	0.069	0.160	0.670	0.967
Meconium	Firmicutes bacterium CAG 95	-0.046	0.245	0.853	0.967
Meconium	Phascolarctobacterium faecium	0.150	0.266	0.574	0.967
Meconium	Dialister invisus	0.357	0.302	0.244	0.967

Exposure Window	Species	Effect Estimate[a]	Std. Error	p-value	q-value
Meconium	Veillonella atypica	-0.041	0.180	0.821	0.967
Meconium	Veillonella dispar	0.122	0.100	0.226	0.967
Meconium	Veillonella parvula	0.048	0.204	0.816	0.967
Meconium	Parvimonas micra	0.056	0.149	0.712	0.967
Meconium	Parasutterella excrementihominis	0.196	0.275	0.479	0.967
Meconium	Bilophila wadsworthia	0.103	0.141	0.469	0.967
Meconium	Desulfovibrio piger	0.019	0.121	0.876	0.967
Meconium	Escherichia coli	0.119	0.286	0.680	0.967
Meconium	Haemophilus parainfluenzae	0.141	0.149	0.349	0.967
Meconium	Proteobacteria bacterium CAG 139	-0.103	0.103	0.321	0.967
Meconium	Akkermansia muciniphila	-0.940	0.479	0.056	0.967
Meconium	Barnesiella intestinihominis	-0.016	0.326	0.961	0.973
Meconium	Tyzzerella nexilis	-0.007	0.217	0.973	0.979
Meconium	Anaeromassilibacillus sp An250	0.001	0.303	0.998	0.998
Cross-sectional	Clostridium sp CAG 167	0.806	0.283	0.006	0.555
Cross-sectional	Actinomyces johnsonii	0.931	0.349	0.009	0.555
Cross-sectional	Corynebacterium durum	0.610	0.239	0.013	0.555
Cross-sectional	Rothia mucilaginosa	0.678	0.293	0.024	0.555
Cross-sectional	Gemella sanguinis	0.300	0.135	0.030	0.555
Cross-sectional	Eubacterium limosum	0.380	0.166	0.024	0.555
Cross-sectional	Ruminococcus lactaris	-0.853	0.393	0.033	0.555
Cross-sectional	Bacteroides finegoldii	0.553	0.261	0.037	0.555
Cross-sectional	Eubacterium sp CAG 180	-1.055	0.498	0.037	0.555
Cross-sectional	Bacteroides caccae	-0.764	0.370	0.042	0.555
Cross-sectional	Clostridium scindens	-0.640	0.307	0.041	0.555
Cross-sectional	Akkermansia muciniphila	-1.556	0.750	0.041	0.555
Cross-sectional	Actinobaculum sp oral taxon 183	0.604	0.334	0.074	0.615
Cross-sectional	Bifidobacterium animalis	0.628	0.351	0.077	0.615
Cross-sectional	Streptococcus mitis	0.562	0.289	0.056	0.555
Cross-sectional	Streptococcus sp F0442	0.375	0.201	0.066	0.615
Cross-sectional	Mogibacterium diversum	0.534	0.274	0.055	0.555
Cross-sectional	Clostridium spiroforme	-0.830	0.427	0.055	0.555
Cross-sectional	Veillonella atypica	0.400	0.221	0.075	0.615
Cross-sectional	Veillonella dispar	0.228	0.113	0.048	0.555
Cross-sectional	Actinomyces odontolyticus	0.521	0.304	0.091	0.634
Cross-sectional	Faecalibacterium prausnitzii	-0.407	0.240	0.095	0.634
Cross-sectional	Ruthenibacterium lactatiformans	-0.398	0.236	0.095	0.634
Cross-sectional	Streptococcus salivarius	-0.419	0.251	0.099	0.634
Cross-sectional	Streptococcus sp A12	0.428	0.257	0.100	0.634
Cross-sectional	Clostridium sp CAG 58	-0.517	0.338	0.131	0.770
Cross-sectional	Lawsonibacter asaccharolyticus	0.384	0.252	0.131	0.770
Cross-sectional	Bacteroides massiliensis	-0.487	0.326	0.139	0.782
Cross-sectional	Bacteroides faecis	-0.502	0.339	0.143	0.782
Cross-sectional	Coprococcus eutactus	0.871	0.603	0.152	0.807
Cross-sectional	Actinomyces graevenitzii	0.280	0.203	0.172	0.858
Cross-sectional	Actinomyces massiliensis	0.301	0.220	0.175	0.858
Cross-sectional	Bacteroides ovatus	-0.560	0.412	0.178	0.858
Cross-sectional	Actinomyces sp ICM47	0.484	0.362	0.185	0.866
Cross-sectional	Methanobrevibacter smithii	-0.377	0.299	0.211	0.888
Cross-sectional	Actinomyces naeslundii	0.351	0.418	0.404	0.905
Cross-sectional	Actinomyces oris	-0.395	0.356	0.270	0.892
Cross-sectional	Actinomyces sp oral taxon 181	-0.090	0.120	0.455	0.905

Exposure Window	Species	Effect Estimate[a]	Std. Error	p-value	q-value
Cross-sectional	Actinomyces turicensis	0.143	0.190	0.456	0.905
Cross-sectional	Bifidobacterium adolescentis	-0.501	0.524	0.341	0.905
Cross-sectional	Bifidobacterium longum	-0.189	0.238	0.430	0.905
Cross-sectional	Collinsella aerofaciens	0.587	0.575	0.311	0.905
Cross-sectional	Collinsella massiliensis	-0.153	0.203	0.453	0.905
Cross-sectional	Eggerthella lenta	0.335	0.290	0.251	0.892
Cross-sectional	Gordonibacter pamelaeae	0.146	0.210	0.490	0.905
Cross-sectional	Bacteroides coprocola	-0.204	0.204	0.322	0.905
Cross-sectional	Bacteroides galacturonicus	0.183	0.204	0.373	0.905
Cross-sectional	Bacteroides vulgatus	-0.472	0.429	0.274	0.892
Cross-sectional	Bacteroides xylanisolvens	0.316	0.501	0.530	0.943
Cross-sectional	Barnesiella intestinihominis	-0.498	0.453	0.275	0.892
Cross-sectional	Prevotella copri	-0.256	0.316	0.421	0.905
Cross-sectional	Alistipes indistinctus	-0.396	0.318	0.218	0.888
Cross-sectional	Streptococcus australis	0.288	0.276	0.301	0.905
Cross-sectional	Streptococcus sanguinis	0.133	0.193	0.494	0.905
Cross-sectional	Streptococcus thermophilus	0.308	0.417	0.463	0.905
Cross-sectional	Clostridium disporicum	-0.305	0.520	0.559	0.943
Cross-sectional	Clostridium ventriculi	-0.238	0.294	0.420	0.905
Cross-sectional	Eubacterium hallii	0.079	0.119	0.511	0.924
Cross-sectional	Eubacterium sp CAG 251	-0.144	0.202	0.478	0.905
Cross-sectional	Eubacterium sp CAG 274	-0.152	0.199	0.448	0.905
Cross-sectional	Eubacterium sp CAG 38	0.431	0.480	0.372	0.905
Cross-sectional	Eubacterium ventriosum	-0.401	0.352	0.258	0.892
Cross-sectional	Blautia obeum	0.144	0.196	0.464	0.905
Cross-sectional	Blautia sp CAG 257	-0.277	0.222	0.217	0.888
Cross-sectional	Ruminococcus gnavus	-0.410	0.551	0.459	0.905
Cross-sectional	Coprococcus catus	0.473	0.385	0.223	0.888
Cross-sectional	Dorea longicatena	0.236	0.243	0.336	0.905
Cross-sectional	Dorea sp CAG 317	-0.223	0.188	0.239	0.892
Cross-sectional	Eisenbergiella massiliensis	-0.292	0.297	0.330	0.905
Cross-sectional	Clostridium bolteae	0.306	0.378	0.420	0.905
Cross-sectional	Clostridium symbiosum	0.124	0.152	0.416	0.905
Cross-sectional	Lachnospira pectinoschiza	-0.513	0.520	0.326	0.905
Cross-sectional	Roseburia hominis	0.447	0.424	0.295	0.905
Cross-sectional	Roseburia sp CAG 471	0.423	0.355	0.236	0.892
Cross-sectional	Sellimonas intestinalis	-0.517	0.447	0.250	0.892
Cross-sectional	Intestinibacter bartlettii	-0.142	0.174	0.420	0.905
Cross-sectional	Anaeromassilibacillus sp An250	-0.272	0.397	0.495	0.905
Cross-sectional	Anaerotruncus colihominis	0.237	0.251	0.347	0.905
Cross-sectional	Flavonifractor plautii	-0.437	0.510	0.394	0.905
Cross-sectional	Clostridium leptum	-0.502	0.404	0.218	0.888
Cross-sectional	Eubacterium siraeum	-0.298	0.421	0.482	0.905
Cross-sectional	Coprobacillus cateniformis	-0.307	0.438	0.486	0.905
Cross-sectional	Turicibacter sanguinis	-0.515	0.392	0.193	0.875
Cross-sectional	Firmicutes bacterium CAG 83	0.248	0.362	0.495	0.905
Cross-sectional	Phascolarctobacterium faecium	-0.257	0.371	0.490	0.905
Cross-sectional	Veillonella parvula	0.171	0.244	0.486	0.905
Cross-sectional	Parvimonas micra	0.174	0.192	0.369	0.905
Cross-sectional	Parasutterella excrementihominis	-0.388	0.348	0.268	0.892
Cross-sectional	Desulfovibrio piger	-0.175	0.227	0.444	0.905
Cross-sectional	Escherichia coli	-0.237	0.386	0.541	0.943

Exposure Window	Species	Effect Estimate[a]	Std. Error	p-value	q-value
Cross-sectional	Haemophilus parainfluenzae	0.145	0.154	0.347	0.905
Cross-sectional	Dialister invisus	-0.256	0.454	0.575	0.943
Cross-sectional	Actinomyces sp HMSC035G02	-0.148	0.266	0.578	0.943
Cross-sectional	Alistipes putredinis	0.189	0.382	0.623	0.943
Cross-sectional	Streptococcus parasanguinis	0.193	0.351	0.584	0.943
Cross-sectional	Eubacterium sulci	-0.126	0.244	0.606	0.943
Cross-sectional	Intestinimonas butyriciproducens	0.127	0.260	0.626	0.943
Cross-sectional	Blautia wexlerae	-0.109	0.217	0.619	0.943
Cross-sectional	Roseburia faecis	-0.203	0.415	0.626	0.943
Cross-sectional	Roseburia sp CAG 309	-0.123	0.223	0.581	0.943
Cross-sectional	Agathobaculum butyriciproducens	-0.263	0.480	0.585	0.943
Cross-sectional	Parabacteroides merdae	0.217	0.454	0.633	0.943
Cross-sectional	Fusicatenibacter saccharivorans	-0.086	0.179	0.634	0.943
Cross-sectional	Adlercreutzia equolifaciens	0.088	0.189	0.642	0.943
Cross-sectional	Blautia hydrogenotrophica	-0.142	0.308	0.645	0.943
Cross-sectional	Alistipes finegoldii	0.183	0.406	0.652	0.943
Cross-sectional	Bacteroides fragilis	0.216	0.493	0.663	0.943
Cross-sectional	Roseburia sp CAG 182	-0.179	0.410	0.663	0.943
Cross-sectional	Gemmiger formicilis	-0.139	0.318	0.664	0.943
Cross-sectional	Parabacteroides distasonis	-0.177	0.415	0.670	0.943
Cross-sectional	Lactobacillus rogosae	-0.085	0.213	0.689	0.943
Cross-sectional	Bifidobacterium bifidum	-0.167	0.450	0.711	0.943
Cross-sectional	Enterorhabdus caecimuris	0.088	0.279	0.753	0.943
Cross-sectional	Bacteroides eggerthii	0.071	0.226	0.753	0.943
Cross-sectional	Bacteroides uniformis	0.128	0.373	0.732	0.943
Cross-sectional	Paraprevotella xylaniphila	-0.094	0.256	0.715	0.943
Cross-sectional	Anaerostipes hadrus	-0.053	0.133	0.692	0.943
Cross-sectional	Dorea formicigenerans	0.075	0.200	0.710	0.943
Cross-sectional	Eisenbergiella tayi	-0.123	0.343	0.721	0.943
Cross-sectional	Roseburia intestinalis	0.182	0.488	0.711	0.943
Cross-sectional	Tyzzerella nexilis	0.076	0.240	0.752	0.943
Cross-sectional	Ruminococcus bicirculans	-0.170	0.453	0.708	0.943
Cross-sectional	Erysipelatoclostridium ramosum	0.112	0.317	0.725	0.943
Cross-sectional	Firmicutes bacterium CAG 110	-0.205	0.568	0.719	0.943
Cross-sectional	Firmicutes bacterium CAG 94	0.070	0.216	0.748	0.943
Cross-sectional	Firmicutes bacterium CAG 95	0.122	0.362	0.737	0.943
Cross-sectional	Romboutsia ilealis	-0.102	0.333	0.761	0.943
Cross-sectional	Collinsella intestinalis	-0.075	0.260	0.773	0.943
Cross-sectional	Holdemania filiformis	-0.052	0.191	0.784	0.943
Cross-sectional	Roseburia inulinivorans	0.093	0.348	0.790	0.943
Cross-sectional	Asaccharobacter celatus	-0.042	0.177	0.813	0.943
Cross-sectional	Bacteroides thetaiotaomicron	0.105	0.459	0.821	0.943
Cross-sectional	Christensenella minuta	0.048	0.219	0.827	0.943
Cross-sectional	Eubacterium rectale	0.115	0.525	0.826	0.943
Cross-sectional	Bifidobacterium catenulatum	0.071	0.450	0.876	0.943
Cross-sectional	Bifidobacterium pseudocatenulatum	0.089	0.499	0.859	0.943
Cross-sectional	Bacteroides cellulosilyticus	-0.066	0.365	0.857	0.943
Cross-sectional	Bacteroides dorei	-0.086	0.451	0.850	0.943
Cross-sectional	Bacteroides stercoris	0.078	0.395	0.844	0.943
Cross-sectional	Lactococcus lactis	0.070	0.368	0.850	0.943
Cross-sectional	Catabacter hongkongensis	0.063	0.364	0.863	0.943
Cross-sectional	Hungatella hathewayi	0.062	0.385	0.872	0.943

Exposure Window	Species	Effect Estimate[a]	Std. Error	p-value	q-value
Cross-sectional	Monoglobus pectinilyticus	0.102	0.483	0.833	0.943
Cross-sectional	Eubacterium ramulus	0.051	0.265	0.847	0.943
Cross-sectional	Coprococcus comes	0.102	0.533	0.848	0.943
Cross-sectional	Ruminococcus bromii	0.089	0.468	0.850	0.943
Cross-sectional	Ruminococcus callidus	-0.057	0.371	0.877	0.943
Cross-sectional	Clostridium innocuum	-0.075	0.425	0.860	0.943
Cross-sectional	Blautia producta	-0.038	0.276	0.892	0.951
Cross-sectional	Oscillibacter sp 57 20	0.042	0.349	0.904	0.957
Cross-sectional	Bilophila wadsworthia	0.028	0.246	0.909	0.957
Cross-sectional	Alistipes shahii	-0.042	0.432	0.923	0.966
Cross-sectional	Collinsella stercoris	0.027	0.340	0.938	0.975
Cross-sectional	Eubacterium eligens	-0.037	0.563	0.948	0.979
Cross-sectional	Actinomyces sp S6 Spd3	0.004	0.133	0.974	0.996
Cross-sectional	Odoribacter splanchnicus	-0.004	0.400	0.992	0.996
Cross-sectional	Ruminococcus torques	-0.001	0.209	0.995	0.996
Cross-sectional	Oscillibacter sp CAG 241	0.002	0.433	0.996	0.996
Cross-sectional	Firmicutes bacterium CAG 145	0.005	0.464	0.992	0.996
Meconium Adj.[b]	Methanobrevibacter smithii	-0.096	0.121	0.434	0.990
Meconium Adj.[b]	Actinobaculum sp oral taxon 183	0.493	0.230	0.038	0.990
Meconium Adj.[b]	Actinomyces johnsonii	0.195	0.267	0.468	0.990
Meconium Adj.[b]	Actinomyces massiliensis	-0.023	0.138	0.869	0.990
Meconium Adj.[b]	Actinomyces naeslundii	-0.203	0.211	0.340	0.990
Meconium Adj.[b]	Actinomyces odontolyticus	-0.083	0.202	0.684	0.990
Meconium Adj.[b]	Actinomyces sp HMSC035G02	0.227	0.190	0.240	0.990
Meconium Adj.[b]	Actinomyces sp ICM47	-0.073	0.300	0.808	0.990
Meconium Adj.[b]	Actinomyces sp S6 Spd3	0.081	0.092	0.383	0.990
Meconium Adj.[b]	Actinomyces sp oral taxon 181	-0.127	0.105	0.231	0.990
Meconium Adj.[b]	Actinomyces turicensis	-0.095	0.138	0.493	0.990
Meconium Adj.[b]	Bifidobacterium animalis	-0.466	0.266	0.088	0.990
Meconium Adj.[b]	Bifidobacterium bifidum	0.403	0.281	0.158	0.990
Meconium Adj.[b]	Bifidobacterium catenulatum	-0.054	0.348	0.877	0.990
Meconium Adj.[b]	Bifidobacterium longum	0.157	0.157	0.326	0.990
Meconium Adj.[b]	Bifidobacterium pseudocatenulatum	0.739	0.378	0.057	0.990
Meconium Adj.[b]	Corynebacterium durum	0.154	0.136	0.264	0.990
Meconium Adj.[b]	Rothia mucilaginosa	-0.367	0.227	0.113	0.990
Meconium Adj.[b]	Collinsella aerofaciens	0.256	0.192	0.190	0.990
Meconium Adj.[b]	Collinsella intestinalis	0.097	0.128	0.454	0.990
Meconium Adj.[b]	Collinsella stercoris	0.162	0.243	0.509	0.990
Meconium Adj.[b]	Enorma massiliensis	-0.058	0.073	0.431	0.990
Meconium Adj.[b]	.Collinsella. massiliensis	0.113	0.097	0.248	0.990
Meconium Adj.[b]	Adlercreutzia equolifaciens	-0.195	0.140	0.171	0.990
Meconium Adj.[b]	Asaccharobacter celatus	-0.090	0.130	0.493	0.990
Meconium Adj.[b]	Eggerthella lenta	-0.109	0.201	0.591	0.990
Meconium Adj.[b]	Enterorhabdus caecimuris	-0.248	0.223	0.273	0.990
Meconium Adj.[b]	Gordonibacter pamelaeae	-0.240	0.153	0.124	0.990
Meconium Adj.[b]	Slackia isoflavoniconvertens	0.107	0.234	0.650	0.990
Meconium Adj.[b]	Bacteroides caccae	0.192	0.319	0.551	0.990
Meconium Adj.[b]	Bacteroides cellulosilyticus	-0.060	0.230	0.796	0.990
Meconium Adj.[b]	Bacteroides coprocola	0.116	0.087	0.190	0.990
Meconium Adj.[b]	Bacteroides dorei	-0.096	0.354	0.788	0.990
Meconium Adj.[b]	Bacteroides faecis	-0.149	0.228	0.516	0.990
Meconium Adj.[b]	Bacteroides finegoldii	-0.067	0.137	0.626	0.990

Exposure Window	Species	Effect Estimate[a]	Std. Error	p-value	q-value
Meconium Adj.[b]	Bacteroides fragilis	-0.183	0.420	0.666	0.990
Meconium Adj.[b]	Bacteroides galacturonicus	-0.119	0.137	0.390	0.990
Meconium Adj.[b]	Bacteroides intestinalis	-0.162	0.125	0.203	0.990
Meconium Adj.[b]	Bacteroides massiliensis	0.110	0.167	0.516	0.990
Meconium Adj.[b]	Bacteroides ovatus	-0.099	0.306	0.749	0.990
Meconium Adj.[b]	Bacteroides salyersiae	-0.049	0.138	0.726	0.990
Meconium Adj.[b]	Bacteroides stercoris	0.328	0.264	0.220	0.990
Meconium Adj.[b]	Bacteroides thetaiotaomicron	-0.057	0.298	0.850	0.990
Meconium Adj.[b]	Bacteroides vulgatus	-0.098	0.307	0.752	0.990
Meconium Adj.[b]	Bacteroides xylanisolvens	-0.085	0.270	0.754	0.990
Meconium Adj.[b]	Barnesiella intestinihominis	0.079	0.327	0.811	0.990
Meconium Adj.[b]	Odoribacter splanchnicus	-0.156	0.257	0.548	0.990
Meconium Adj.[b]	Paraprevotella xylaniphila	-0.197	0.118	0.101	0.990
Meconium Adj.[b]	Prevotella copri	0.052	0.212	0.809	0.990
Meconium Adj.[b]	Alistipes finegoldii	0.093	0.325	0.775	0.990
Meconium Adj.[b]	Alistipes indistinctus	-0.268	0.262	0.313	0.990
Meconium Adj.[b]	Alistipes putredinis	-0.058	0.327	0.859	0.990
Meconium Adj.[b]	Alistipes shahii	-0.191	0.317	0.549	0.990
Meconium Adj.[b]	Parabacteroides merdae	-0.314	0.358	0.385	0.990
Meconium Adj.[b]	Gemella sanguinis	0.020	0.111	0.855	0.990
Meconium Adj.[b]	Lactobacillus rhamnosus	-0.112	0.152	0.466	0.990
Meconium Adj.[b]	Lactococcus lactis	-0.222	0.282	0.436	0.990
Meconium Adj.[b]	Streptococcus australis	-0.038	0.234	0.873	0.990
Meconium Adj.[b]	Streptococcus infantis	-0.055	0.090	0.541	0.990
Meconium Adj.[b]	Streptococcus mitis	-0.234	0.260	0.374	0.990
Meconium Adj.[b]	Streptococcus salivarius	0.140	0.186	0.457	0.990
Meconium Adj.[b]	Streptococcus sanguinis	-0.238	0.152	0.124	0.990
Meconium Adj.[b]	Streptococcus sp A12	-0.090	0.209	0.669	0.990
Meconium Adj.[b]	Streptococcus sp F0442	0.082	0.154	0.598	0.990
Meconium Adj.[b]	Streptococcus thermophilus	-0.104	0.340	0.760	0.990
Meconium Adj.[b]	Catabacter hongkongensis	-0.271	0.296	0.365	0.990
Meconium Adj.[b]	Christensenella minuta	-0.100	0.142	0.484	0.990
Meconium Adj.[b]	Clostridium bolteae CAG 59	-0.054	0.172	0.753	0.990
Meconium Adj.[b]	Clostridium disporicum	-0.741	0.359	0.045	0.990
Meconium Adj.[b]	Clostridium sp CAG 167	0.038	0.216	0.860	0.990
Meconium Adj.[b]	Clostridium sp CAG 58	0.067	0.199	0.736	0.990
Meconium Adj.[b]	Clostridium ventriculi	-0.187	0.207	0.373	0.990
Meconium Adj.[b]	Eubacterium sulci	-0.224	0.222	0.320	0.990
Meconium Adj.[b]	Mogibacterium diversum	0.055	0.147	0.713	0.990
Meconium Adj.[b]	Intestinimonas butyriciproducens	-0.095	0.202	0.641	0.990
Meconium Adj.[b]	Lawsonibacter asaccharolyticus	0.054	0.178	0.765	0.990
Meconium Adj.[b]	Monoglobus pectinilyticus	-0.502	0.353	0.162	0.990
Meconium Adj.[b]	Anaerofustis stercorihominis	-0.085	0.095	0.378	0.990
Meconium Adj.[b]	Eubacterium callanderi	0.022	0.113	0.846	0.990
Meconium Adj.[b]	Eubacterium eligens	-0.360	0.341	0.297	0.990
Meconium Adj.[b]	Eubacterium hallii	-0.058	0.090	0.522	0.990
Meconium Adj.[b]	Eubacterium limosum	-0.108	0.109	0.329	0.990
Meconium Adj.[b]	Eubacterium ramulus	-0.220	0.207	0.294	0.990
Meconium Adj.[b]	Eubacterium sp CAG 180	0.135	0.368	0.715	0.990
Meconium Adj.[b]	Eubacterium sp CAG 38	-0.098	0.325	0.764	0.990
Meconium Adj.[b]	Eubacterium ventriosum	-0.286	0.268	0.292	0.990
Meconium Adj.[b]	Anaerostipes hadrus	-0.047	0.093	0.618	0.990

Exposure Window	Species	Effect Estimate[a]	Std. Error	p-value	q-value
Meconium Adj.[b]	Blautia hydrogenotrophica	-0.081	0.184	0.662	0.990
Meconium Adj.[b]	Blautia obeum	-0.088	0.158	0.577	0.990
Meconium Adj.[b]	Blautia producta	-0.288	0.207	0.172	0.990
Meconium Adj.[b]	Blautia sp CAG 257	-0.165	0.129	0.209	0.990
Meconium Adj.[b]	Blautia wexlerae	0.040	0.173	0.817	0.990
Meconium Adj.[b]	Ruminococcus gnavus	-0.465	0.359	0.203	0.990
Meconium Adj.[b]	Ruminococcus torques	-0.051	0.126	0.689	0.990
Meconium Adj.[b]	Coprococcus comes	-0.378	0.420	0.373	0.990
Meconium Adj.[b]	Coprococcus eutactus	-0.057	0.305	0.853	0.990
Meconium Adj.[b]	Dorea formicigenerans	0.189	0.119	0.120	0.990
Meconium Adj.[b]	Dorea longicatena	-0.048	0.166	0.774	0.990
Meconium Adj.[b]	Dorea sp CAG 317	-0.080	0.160	0.621	0.990
Meconium Adj.[b]	Eisenbergiella massiliensis	0.226	0.231	0.333	0.990
Meconium Adj.[b]	Eisenbergiella tayi	0.157	0.259	0.548	0.990
Meconium Adj.[b]	Fusicatenibacter saccharivorans	-0.159	0.127	0.219	0.990
Meconium Adj.[b]	Clostridium scindens	0.055	0.291	0.852	0.990
Meconium Adj.[b]	Clostridium symbiosum	-0.102	0.097	0.299	0.990
Meconium Adj.[b]	Eubacterium rectale	-0.076	0.414	0.856	0.990
Meconium Adj.[b]	Roseburia faecis	-0.196	0.301	0.519	0.990
Meconium Adj.[b]	Roseburia hominis	0.127	0.327	0.699	0.990
Meconium Adj.[b]	Roseburia intestinalis	-0.407	0.386	0.298	0.990
Meconium Adj.[b]	Roseburia inulinivorans	-0.049	0.283	0.862	0.990
Meconium Adj.[b]	Roseburia sp CAG 182	0.349	0.265	0.196	0.990
Meconium Adj.[b]	Roseburia sp CAG 309	-0.225	0.150	0.141	0.990
Meconium Adj.[b]	Roseburia sp CAG 471	0.045	0.272	0.870	0.990
Meconium Adj.[b]	Sellimonas intestinalis	-0.426	0.362	0.245	0.990
Meconium Adj.[b]	Oscillibacter sp 57 20	0.129	0.245	0.602	0.990
Meconium Adj.[b]	Oscillibacter sp CAG 241	0.379	0.332	0.260	0.990
Meconium Adj.[b]	Intestinibacter bartlettii	-0.280	0.112	0.017	0.990
Meconium Adj.[b]	Romboutsia ilealis	0.125	0.251	0.621	0.990
Meconium Adj.[b]	Agathobaculum butyriciproducens	-0.190	0.405	0.641	0.990
Meconium Adj.[b]	Anaeromassilibacillus sp An250	-0.122	0.295	0.682	0.990
Meconium Adj.[b]	Anaerotruncus sp CAG 528	-0.174	0.120	0.155	0.990
Meconium Adj.[b]	Faecalibacterium prausnitzii	0.049	0.193	0.802	0.990
Meconium Adj.[b]	Flavonifractor plautii	0.217	0.372	0.563	0.990
Meconium Adj.[b]	Clostridium leptum	-0.134	0.229	0.561	0.990
Meconium Adj.[b]	Eubacterium siraeum	-0.511	0.260	0.056	0.990
Meconium Adj.[b]	Ruminococcus bromii	-0.181	0.265	0.498	0.990
Meconium Adj.[b]	Ruminococcus callidus	-0.154	0.243	0.530	0.990
Meconium Adj.[b]	Ruminococcus lactaris	-0.377	0.301	0.218	0.990
Meconium Adj.[b]	Ruthenibacterium lactatiformans	0.334	0.187	0.081	0.990
Meconium Adj.[b]	Coprobacillus cateniformis	0.153	0.274	0.580	0.990
Meconium Adj.[b]	Clostridium innocuum	0.065	0.338	0.849	0.990
Meconium Adj.[b]	Clostridium spiroforme	-0.199	0.350	0.573	0.990
Meconium Adj.[b]	Erysipelatoclostridium ramosum	-0.340	0.245	0.174	0.990
Meconium Adj.[b]	Holdemania filiformis	-0.014	0.090	0.876	0.990
Meconium Adj.[b]	Turicibacter sanguinis	-0.186	0.295	0.533	0.990
Meconium Adj.[b]	Firmicutes bacterium CAG 110	-0.095	0.447	0.834	0.990
Meconium Adj.[b]	Firmicutes bacterium CAG 145	-0.234	0.363	0.523	0.990
Meconium Adj.[b]	Firmicutes bacterium CAG 83	0.277	0.299	0.360	0.990
Meconium Adj.[b]	Phascolarctobacterium faecium	0.163	0.274	0.555	0.990
Meconium Adj.[b]	Dialister invisus	0.312	0.309	0.318	0.990

Exposure Window	Species	Effect Estimate[a]	Std. Error	p-value	q-value
Meconium Adj.[b]	Veillonella dispar	0.091	0.099	0.367	0.990
Meconium Adj.[b]	Veillonella parvula	0.053	0.210	0.804	0.990
Meconium Adj.[b]	Parasutterella excrementihominis	0.250	0.280	0.378	0.990
Meconium Adj.[b]	Bilophila wadsworthia	0.132	0.143	0.365	0.990
Meconium Adj.[b]	Desulfovibrio piger	0.035	0.124	0.781	0.990
Meconium Adj.[b]	Escherichia coli	0.154	0.293	0.604	0.990
Meconium Adj.[b]	Haemophilus parainfluenzae	0.119	0.152	0.439	0.990
Meconium Adj.[b]	Proteobacteria bacterium CAG 139	-0.100	0.106	0.349	0.990
Meconium Adj.[b]	Akkermansia muciniphila	-0.945	0.493	0.062	0.990
Meconium Adj.[b]	Actinomyces graevenitzii	0.026	0.182	0.889	0.990
Meconium Adj.[b]	Hungatella hathewayi	0.046	0.325	0.889	0.990
Meconium Adj.[b]	Actinomyces oris	0.005	0.224	0.983	0.995
Meconium Adj.[b]	Bifidobacterium adolescentis	0.023	0.341	0.947	0.995
Meconium Adj.[b]	Bacteroides uniformis	-0.013	0.259	0.960	0.995
Meconium Adj.[b]	Parabacteroides distasonis	0.003	0.341	0.993	0.995
Meconium Adj.[b]	Lactobacillus rogosae	-0.009	0.153	0.954	0.995
Meconium Adj.[b]	Streptococcus parasanguinis	0.022	0.288	0.941	0.995
Meconium Adj.[b]	Coprococcus catus	0.006	0.210	0.978	0.995
Meconium Adj.[b]	Clostridium bolteae	-0.007	0.194	0.972	0.995
Meconium Adj.[b]	Lachnospira pectinoschiza	-0.016	0.263	0.951	0.995
Meconium Adj.[b]	Tyzzerella nexilis	0.025	0.222	0.911	0.995
Meconium Adj.[b]	Anaerotruncus colihominis	-0.008	0.109	0.941	0.995
Meconium Adj.[b]	Gemmiger formicilis	0.008	0.255	0.975	0.995
Meconium Adj.[b]	Ruminococcus bicirculans	-0.018	0.324	0.956	0.995
Meconium Adj.[b]	Firmicutes bacterium CAG 94	-0.008	0.152	0.959	0.995
Meconium Adj.[b]	Firmicutes bacterium CAG 95	-0.008	0.251	0.976	0.995
Meconium Adj.[b]	Veillonella atypica	-0.015	0.185	0.935	0.995
Meconium Adj.[b]	Parvimonas micra	-0.001	0.147	0.995	0.995

[a]Models are adjusted for whether the child was ever breastfed, sex, mode of birth, and socioeconomic status
[b]Meconium Adj. includes cross-sectional exposure in the model

Table S4. Difference in MetaCyc pathway relative abundance comparing acetaminophen exposed to unexposed

Exposure Window	MetaCyc Pathway ID	Effect Estimate[a]	Std. Error	p-value	q-value
Meconium	PWY.5677	0.328	0.081	0.000	0.072
Meconium	PWY.922	-0.270	0.107	0.015	0.995
Meconium	THISYN.PWY	0.151	0.060	0.015	0.995
Meconium	BIOTIN.BIOSYNTHESIS.PWY	0.327	0.159	0.045	0.995
Meconium	POLYAMINSYN3.PWY	-0.312	0.140	0.031	0.995
Meconium	PROPFERM.PWY	0.090	0.046	0.056	0.995
Meconium	PWY.4041	-0.081	0.039	0.042	0.995
Meconium	PWY.5695	0.031	0.014	0.034	0.995
Meconium	PWY.6519	0.333	0.163	0.048	0.995
Meconium	PWY.6737	-0.029	0.015	0.059	0.995
Meconium	PWY.7288	0.228	0.116	0.055	0.995
Meconium	PWY66.391	0.237	0.113	0.042	0.995
Meconium	HISDEG.PWY	0.182	0.094	0.060	0.995
Meconium	HISTSYN.PWY	-0.024	0.014	0.093	0.995
Meconium	HOMOSER.METSYN.PWY	-0.093	0.050	0.073	0.995
Meconium	ILEUSYN.PWY	-0.025	0.014	0.087	0.995
Meconium	MET.SAM.PWY	-0.068	0.040	0.096	0.995
Meconium	METSYN.PWY	-0.075	0.042	0.080	0.995
Meconium	P125.PWY	-0.376	0.214	0.085	0.995
Meconium	P621.PWY	-0.255	0.138	0.071	0.995
Meconium	PWY.3001	-0.025	0.014	0.077	0.995
Meconium	PWY.5347	-0.067	0.037	0.079	0.995
Meconium	PWY.5910	-0.170	0.094	0.077	0.995
Meconium	PWY.7111	-0.025	0.015	0.092	0.995
Meconium	PWY.821	-0.273	0.162	0.100	0.995
Meconium	PWY0.1061	-0.146	0.084	0.087	0.995
Meconium	VALSYN.PWY	-0.025	0.014	0.087	0.995
Meconium	PWY.7385	-0.191	0.114	0.101	0.995
Meconium	ARGININE.SYN4.PWY	0.235	0.161	0.152	0.995
Meconium	ASPASN.PWY	-0.038	0.024	0.127	0.995
Meconium	BRANCHED.CHAIN.AA.SYN.PWY	-0.023	0.015	0.133	0.995
Meconium	COA.PWY.1	-0.013	0.009	0.136	0.995
Meconium	FAO.PWY	0.175	0.108	0.111	0.995
Meconium	GLYCOLYSIS.E.D	0.170	0.116	0.152	0.995
Meconium	GLYCOLYSIS	-0.054	0.036	0.138	0.995
Meconium	PWY.5030	0.170	0.109	0.126	0.995

Exposure Window	MetaCyc Pathway ID	Effect Estimate[a]	Std. Error	p-value	q-value
Meconium	PWY.5100	-0.036	0.023	0.133	0.995
Meconium	PWY.5103	-0.025	0.016	0.136	0.995
Meconium	PWY.5136	0.182	0.111	0.107	0.995
Meconium	PWY.5138	0.218	0.147	0.146	0.995
Meconium	PWY.5505	0.237	0.150	0.122	0.995
Meconium	PWY.6263	0.202	0.137	0.148	0.995
Meconium	PWY.7323	0.094	0.063	0.146	0.995
Meconium	PWY0.1241	0.244	0.168	0.152	0.995
Meconium	PWY0.1479	-0.068	0.045	0.140	0.995
Meconium	THRESYN.PWY	-0.025	0.016	0.135	0.995
Meconium	METHGLYUT.PWY	0.209	0.144	0.154	0.995
Meconium	ORNDEG.PWY	-0.092	0.065	0.163	0.995
Meconium	PWY.6318	0.101	0.070	0.161	0.995
Meconium	PWY.6396	-0.116	0.082	0.163	0.995
Meconium	PWY.4984	0.152	0.109	0.169	0.995
Meconium	PWY.5022	0.150	0.109	0.178	0.995
Meconium	CITRULBIO.PWY	0.147	0.108	0.181	0.995
Meconium	PWY.5994	0.219	0.162	0.182	0.995
Meconium	GLUCONEO.PWY	-0.028	0.021	0.188	0.995
Meconium	KETOGLUCONMET.PWY	-0.300	0.230	0.198	0.995
Meconium	PWY.4242	-0.021	0.016	0.203	0.995
Meconium	OANTIGEN.PWY	-0.027	0.021	0.207	0.995
Meconium	GLUTORN.PWY	-0.022	0.017	0.213	0.995
Meconium	LACTOSECAT.PWY	-0.097	0.077	0.211	0.995
Meconium	PWY.6572	0.080	0.063	0.213	0.995
Meconium	PWY.7237	-0.047	0.037	0.213	0.995
Meconium	ARGSYNBSUB.PWY	-0.017	0.014	0.226	0.995
Meconium	PWY.5173	-0.227	0.186	0.227	0.995
Meconium	THREOCAT.PWY	-0.080	0.065	0.226	0.995
Meconium	UDPNAGSYN.PWY	-0.037	0.030	0.225	0.995
Meconium	ARGSYN.PWY	-0.016	0.014	0.253	0.995
Meconium	COA.PWY	-0.017	0.014	0.241	0.995
Meconium	GALACTARDEG.PWY	0.208	0.173	0.234	0.995
Meconium	GLUCARDEG.PWY	0.191	0.161	0.242	0.995
Meconium	GLUCARGALACTSUPER.PWY	0.208	0.173	0.234	0.995
Meconium	GLUDEG.I.PWY	0.144	0.124	0.252	0.995
Meconium	PWY.5177	-0.050	0.042	0.236	0.995

Exposure Window	MetaCyc Pathway ID	Effect Estimate[a]	Std. Error	p-value	q-value
Meconium	PWY.6122	-0.015	0.013	0.238	0.995
Meconium	PWY.6277	-0.015	0.013	0.238	0.995
Meconium	PWY.7199	0.027	0.023	0.240	0.995
Meconium	PWY.7400	-0.016	0.014	0.254	0.995
Meconium	PWY66.400	-0.049	0.041	0.239	0.995
Meconium	UNMAPPED	0.012	0.014	0.394	0.995
Meconium	UNINTEGRATED	-0.004	0.004	0.345	0.995
Meconium	X1CMET2.PWY	-0.005	0.010	0.635	0.995
Meconium	X7ALPHADEHYDROX.PWY	0.140	0.128	0.280	0.995
Meconium	ALLANTOINDEG.PWY	0.051	0.090	0.577	0.995
Meconium	ANAEROFRUCAT.PWY	-0.024	0.030	0.432	0.995
Meconium	ANAGLYCOLYSIS.PWY	-0.014	0.019	0.454	0.995
Meconium	ARG.POLYAMINE.SYN	-0.079	0.080	0.330	0.995
Meconium	ARGDEG.PWY	-0.113	0.110	0.314	0.995
Meconium	ARGORNPROST.PWY	0.068	0.099	0.496	0.995
Meconium	ARO.PWY	-0.014	0.013	0.299	0.995
Meconium	CALVIN.PWY	-0.014	0.018	0.429	0.995
Meconium	CENTFERM.PWY	-0.053	0.086	0.541	0.995
Meconium	COBALSYN.PWY	-0.030	0.029	0.302	0.995
Meconium	COMPLETE.ARO.PWY	-0.014	0.015	0.341	0.995
Meconium	DAPLYSINESYN.PWY	-0.034	0.049	0.494	0.995
Meconium	DTDPRHAMSYN.PWY	-0.017	0.020	0.419	0.995
Meconium	ENTBACSYN.PWY	0.020	0.060	0.735	0.995
Meconium	FASYN.ELONG.PWY	0.053	0.109	0.627	0.995
Meconium	FERMENTATION.PWY	0.020	0.037	0.591	0.995
Meconium	FOLSYN.PWY	0.083	0.086	0.341	0.995
Meconium	FUC.RHAMCAT.PWY	0.036	0.102	0.724	0.995
Meconium	FUCCAT.PWY	0.051	0.073	0.494	0.995
Meconium	GALACT.GLUCUROCAT.PWY	0.041	0.105	0.701	0.995
Meconium	GLCMANNANAUT.PWY	-0.025	0.045	0.585	0.995
Meconium	GLUCOSE1PMETAB.PWY	-0.082	0.126	0.519	0.995
Meconium	GLUCUROCAT.PWY	0.026	0.082	0.752	0.995
Meconium	GLYCOCAT.PWY	-0.075	0.120	0.536	0.995
Meconium	GLYCOGENSYNTH.PWY	-0.016	0.023	0.499	0.995
Meconium	GLYCOL.GLYOXDEG.PWY	-0.056	0.129	0.665	0.995
Meconium	GLYCOLYSIS.TCA.GLYOX.BYPASS	-0.061	0.131	0.645	0.995
Meconium	GLYOXYLATE.BYPASS	-0.153	0.158	0.338	0.995

Exposure Window	MetaCyc Pathway ID	Effect Estimate[a]	Std. Error	p-value	q-value
Meconium	HCAMHPDEG.PWY	-0.043	0.066	0.513	0.995
Meconium	HEME.BIOSYNTHESIS.II	0.128	0.156	0.416	0.995
Meconium	HEMESYN2.PWY	0.023	0.082	0.781	0.995
Meconium	HSERMETANA.PWY	-0.021	0.021	0.326	0.995
Meconium	METH.ACETATE.PWY	-0.051	0.046	0.267	0.995
Meconium	METHANOGENESIS.PWY	-0.047	0.120	0.696	0.995
Meconium	NAD.BIOSYNTHESIS.II	-0.102	0.221	0.646	0.995
Meconium	NAGLIPASYN.PWY	0.077	0.156	0.623	0.995
Meconium	NONMEVIPP.PWY	-0.023	0.067	0.731	0.995
Meconium	NONOXIPENT.PWY	-0.018	0.026	0.507	0.995
Meconium	ORNARGDEG.PWY	-0.113	0.110	0.314	0.995
Meconium	P105.PWY	-0.080	0.133	0.549	0.995
Meconium	P122.PWY	-0.125	0.226	0.584	0.995
Meconium	P124.PWY	0.097	0.122	0.433	0.995
Meconium	P162.PWY	0.043	0.083	0.609	0.995
Meconium	P164.PWY	0.024	0.051	0.638	0.995
Meconium	P221.PWY	-0.134	0.140	0.343	0.995
Meconium	P4.PWY	-0.038	0.067	0.576	0.995
Meconium	P42.PWY	0.029	0.099	0.770	0.995
Meconium	P441.PWY	0.025	0.066	0.712	0.995
Meconium	P562.PWY	0.020	0.071	0.784	0.995
Meconium	PANTO.PWY	-0.022	0.021	0.319	0.995
Meconium	PANTOSYN.PWY	-0.019	0.018	0.283	0.995
Meconium	PEPTIDOGLYCANSYN.PWY	-0.004	0.009	0.638	0.995
Meconium	PHOSLIPSYN.PWY	0.028	0.066	0.677	0.995
Meconium	POLYAMSYN.PWY	-0.086	0.085	0.320	0.995
Meconium	POLYISOPRENSYN.PWY	0.040	0.132	0.764	0.995
Meconium	PRPP.PWY	0.039	0.069	0.579	0.995
Meconium	PWY.1042	-0.013	0.014	0.379	0.995
Meconium	PWY.1269	0.111	0.100	0.274	0.995
Meconium	PWY.1861	-0.032	0.058	0.585	0.995
Meconium	PWY.241	-0.042	0.054	0.447	0.995
Meconium	PWY.2723	-0.082	0.127	0.521	0.995
Meconium	PWY.2942	-0.008	0.011	0.466	0.995
Meconium	PWY.3781	-0.120	0.149	0.423	0.995
Meconium	PWY.3841	-0.003	0.009	0.714	0.995
Meconium	PWY.4722	-0.083	0.132	0.534	0.995

Exposure Window	MetaCyc Pathway ID	Effect Estimate[a]	Std. Error	p-value	q-value
Meconium	PWY.5005	0.129	0.180	0.477	0.995
Meconium	PWY.5088	0.046	0.043	0.287	0.995
Meconium	PWY.5097	-0.007	0.011	0.492	0.995
Meconium	PWY.5101	-0.066	0.253	0.795	0.995
Meconium	PWY.5104	0.013	0.042	0.766	0.995
Meconium	PWY.5121	0.022	0.069	0.746	0.995
Meconium	PWY.5189	-0.047	0.144	0.747	0.995
Meconium	PWY.5198	-0.037	0.111	0.744	0.995
Meconium	PWY.5265	0.088	0.130	0.501	0.995
Meconium	PWY.5304	-0.075	0.082	0.367	0.995
Meconium	PWY.5345	-0.110	0.144	0.450	0.995
Meconium	PWY.5367	0.114	0.152	0.458	0.995
Meconium	PWY.5384	-0.082	0.086	0.346	0.995
Meconium	PWY.5392	0.044	0.080	0.582	0.995
Meconium	PWY.5464	-0.088	0.155	0.572	0.995
Meconium	PWY.5484	-0.040	0.042	0.346	0.995
Meconium	PWY.561	-0.111	0.150	0.463	0.995
Meconium	PWY.5656	-0.025	0.092	0.789	0.995
Meconium	PWY.5667	-0.011	0.019	0.574	0.995
Meconium	PWY.5675	-0.078	0.145	0.591	0.995
Meconium	PWY.5690	0.031	0.047	0.514	0.995
Meconium	PWY.5723	-0.030	0.116	0.796	0.995
Meconium	PWY.5747	-0.070	0.146	0.637	0.995
Meconium	PWY.5845	0.154	0.152	0.317	0.995
Meconium	PWY.5850	0.054	0.076	0.477	0.995
Meconium	PWY.5860	0.055	0.076	0.475	0.995
Meconium	PWY.5862	0.155	0.154	0.319	0.995
Meconium	PWY.5863	0.070	0.143	0.625	0.995
Meconium	PWY.5896	0.054	0.076	0.477	0.995
Meconium	PWY.5913	-0.038	0.066	0.565	0.995
Meconium	PWY.5918	0.093	0.141	0.514	0.995
Meconium	PWY.5920	0.029	0.096	0.763	0.995
Meconium	PWY.5941	-0.086	0.207	0.681	0.995
Meconium	PWY.5971	0.046	0.102	0.659	0.995
Meconium	PWY.5973	0.073	0.095	0.450	0.995
Meconium	PWY.5989	0.112	0.113	0.326	0.995
Meconium	PWY.6113	0.042	0.095	0.663	0.995

Exposure Window	MetaCyc Pathway ID	Effect Estimate[a]	Std. Error	p-value	q-value
Meconium	PWY.6121	-0.015	0.014	0.293	0.995
Meconium	PWY.6123	0.011	0.016	0.526	0.995
Meconium	PWY.6124	0.010	0.017	0.556	0.995
Meconium	PWY.6126	-0.008	0.020	0.681	0.995
Meconium	PWY.6151	-0.004	0.016	0.785	0.995
Meconium	PWY.6163	-0.014	0.015	0.351	0.995
Meconium	PWY.6168	-0.010	0.027	0.709	0.995
Meconium	PWY.621	-0.019	0.042	0.648	0.995
Meconium	PWY.622	-0.045	0.118	0.704	0.995
Meconium	PWY.6270	-0.030	0.090	0.741	0.995
Meconium	PWY.6282	0.078	0.117	0.511	0.995
Meconium	PWY.6284	0.066	0.104	0.529	0.995
Meconium	PWY.6285	-0.058	0.065	0.375	0.995
Meconium	PWY.6305	0.011	0.041	0.794	0.995
Meconium	PWY.6317	-0.025	0.027	0.356	0.995
Meconium	PWY.6349	-0.043	0.125	0.733	0.995
Meconium	PWY.6385	-0.008	0.010	0.441	0.995
Meconium	PWY.6386	-0.007	0.009	0.488	0.995
Meconium	PWY.6387	-0.006	0.009	0.477	0.995
Meconium	PWY.6435	0.149	0.176	0.403	0.995
Meconium	PWY.6470	-0.047	0.066	0.482	0.995
Meconium	PWY.6478	0.087	0.094	0.360	0.995
Meconium	PWY.6527	-0.035	0.032	0.288	0.995
Meconium	PWY.6531	0.086	0.172	0.620	0.995
Meconium	PWY.6549	0.029	0.035	0.410	0.995
Meconium	PWY.6588	0.071	0.147	0.632	0.995
Meconium	PWY.6590	-0.053	0.085	0.538	0.995
Meconium	PWY.6595	0.064	0.181	0.726	0.995
Meconium	PWY.6608	-0.011	0.038	0.764	0.995
Meconium	PWY.6609	-0.009	0.018	0.604	0.995
Meconium	PWY.6612	0.085	0.088	0.341	0.995
Meconium	PWY.6628	-0.153	0.158	0.336	0.995
Meconium	PWY.6630	-0.035	0.108	0.746	0.995
Meconium	PWY.6690	-0.043	0.066	0.513	0.995
Meconium	PWY.6703	-0.024	0.029	0.412	0.995
Meconium	PWY.6731	-0.036	0.104	0.732	0.995
Meconium	PWY.6749	0.116	0.106	0.280	0.995

Exposure Window	MetaCyc Pathway ID	Effect Estimate[a]	Std. Error	p-value	q-value
Meconium	PWY.6837	-0.035	0.052	0.512	0.995
Meconium	PWY.6859	0.133	0.180	0.465	0.995
Meconium	PWY.6876	-0.127	0.117	0.286	0.995
Meconium	PWY.6895	0.115	0.118	0.336	0.995
Meconium	PWY.6897	0.017	0.037	0.648	0.995
Meconium	PWY.6936	-0.007	0.023	0.765	0.995
Meconium	PWY.7003	0.081	0.132	0.541	0.995
Meconium	PWY.7013	-0.028	0.057	0.628	0.995
Meconium	PWY.7046	0.054	0.143	0.706	0.995
Meconium	PWY.7115	-0.075	0.067	0.269	0.995
Meconium	PWY.7117	-0.041	0.062	0.513	0.995
Meconium	PWY.7187	0.011	0.039	0.771	0.995
Meconium	PWY.7196	-0.022	0.028	0.445	0.995
Meconium	PWY.7204	0.069	0.077	0.376	0.995
Meconium	PWY.7208	-0.025	0.037	0.507	0.995
Meconium	PWY.7209	0.101	0.169	0.554	0.995
Meconium	PWY.7210	-0.105	0.179	0.561	0.995
Meconium	PWY.7219	-0.008	0.014	0.580	0.995
Meconium	PWY.7229	-0.010	0.013	0.467	0.995
Meconium	PWY.7242	0.034	0.091	0.712	0.995
Meconium	PWY.724	-0.007	0.009	0.434	0.995
Meconium	PWY.7282	0.152	0.138	0.279	0.995
Meconium	PWY.7286	-0.059	0.132	0.655	0.995
Meconium	PWY.7315	-0.038	0.111	0.736	0.995
Meconium	PWY.7316	0.140	0.128	0.278	0.995
Meconium	PWY.7328	-0.078	0.115	0.498	0.995
Meconium	PWY.7332	0.151	0.155	0.335	0.995
Meconium	PWY.7357	-0.022	0.021	0.298	0.995
Meconium	PWY.7371	0.176	0.179	0.330	0.995
Meconium	PWY.7392	0.123	0.166	0.462	0.995
Meconium	PWY.7456	0.036	0.061	0.564	0.995
Meconium	PWY.7560	-0.031	0.093	0.741	0.995
Meconium	PWY.7616	-0.199	0.209	0.347	0.995
Meconium	PWY.7663	0.107	0.101	0.297	0.995
Meconium	PWY.7664	0.068	0.118	0.566	0.995
Meconium	PWY0.1261	0.057	0.123	0.644	0.995
Meconium	PWY0.1277	-0.027	0.051	0.597	0.995

Exposure Window	MetaCyc Pathway ID	Effect Estimate[a]	Std. Error	p-value	q-value
Meconium	PWY0.1296	-0.024	0.025	0.332	0.995
Meconium	PWY0.1319	-0.011	0.019	0.573	0.995
Meconium	PWY0.1415	-0.067	0.126	0.598	0.995
Meconium	PWY0.42	-0.019	0.074	0.796	0.995
Meconium	PWY0.781	-0.043	0.060	0.483	0.995
Meconium	PWY0.845	0.123	0.157	0.436	0.995
Meconium	PWY0.862	0.064	0.121	0.598	0.995
Meconium	PWY3O.355	0.063	0.163	0.701	0.995
Meconium	PWY490.3	0.068	0.089	0.444	0.995
Meconium	PWY4FS.7	0.036	0.072	0.620	0.995
Meconium	PWY4FS.8	0.036	0.072	0.620	0.995
Meconium	PWY66.389	-0.188	0.203	0.360	0.995
Meconium	PWY66.399	0.013	0.032	0.697	0.995
Meconium	PWY66.422	-0.019	0.018	0.313	0.995
Meconium	PWYG.321	0.059	0.119	0.626	0.995
Meconium	PYRIDNUCSAL.PWY	-0.095	0.115	0.414	0.995
Meconium	PYRIDNUCSYN.PWY	-0.006	0.016	0.714	0.995
Meconium	PYRIDOXSYN.PWY	0.093	0.161	0.568	0.995
Meconium	REDCITCYC	0.042	0.148	0.778	0.995
Meconium	RHAMCAT.PWY	0.038	0.043	0.379	0.995
Meconium	RIBOSYN2.PWY	-0.008	0.024	0.740	0.995
Meconium	RUMP.PWY	0.104	0.175	0.555	0.995
Meconium	SER.GLYSYN.PWY	-0.017	0.016	0.283	0.995
Meconium	SO4ASSIM.PWY	-0.121	0.153	0.433	0.995
Meconium	SULFATE.CYS.PWY	-0.111	0.146	0.451	0.995
Meconium	TCA.GLYOX.BYPASS	-0.068	0.137	0.619	0.995
Meconium	TCA	0.038	0.046	0.417	0.995
Meconium	THISYNARA.PWY	-0.015	0.039	0.697	0.995
Meconium	TRNA.CHARGING.PWY	-0.002	0.009	0.799	0.995
Meconium	TRPSYN.PWY	-0.011	0.028	0.690	0.995
Meconium	PWY.5659	0.011	0.046	0.803	0.995
Meconium	COLANSYN.PWY	0.006	0.034	0.872	0.995
Meconium	CRNFORCAT.PWY	-0.015	0.099	0.881	0.995
Meconium	FASYN.INITIAL.PWY	0.024	0.133	0.859	0.995
Meconium	GALACTUROCAT.PWY	0.017	0.085	0.839	0.995
Meconium	GOLPDLCAT.PWY	0.009	0.051	0.865	0.995
Meconium	HEXITOLDEGSUPER.PWY	-0.004	0.029	0.898	0.995

Exposure Window	MetaCyc Pathway ID	Effect Estimate[a]	Std. Error	p-value	q-value
Meconium	P161.PWY	-0.035	0.180	0.845	0.995
Meconium	P23.PWY	0.030	0.186	0.871	0.995
Meconium	P461.PWY	0.011	0.051	0.828	0.995
Meconium	PPGPPMET.PWY	-0.010	0.047	0.836	0.995
Meconium	PWY.2941	0.006	0.060	0.924	0.995
Meconium	PWY.4981	-0.012	0.065	0.853	0.995
Meconium	PWY.5004	0.009	0.079	0.908	0.995
Meconium	PWY.5154	-0.012	0.072	0.864	0.995
Meconium	PWY.5188	-0.006	0.035	0.865	0.995
Meconium	PWY.5676	0.004	0.047	0.933	0.995
Meconium	PWY.5686	-0.002	0.010	0.876	0.995
Meconium	PWY.5791	0.012	0.144	0.931	0.995
Meconium	PWY.5837	0.012	0.144	0.931	0.995
Meconium	PWY.5838	0.026	0.148	0.860	0.995
Meconium	PWY.5861	0.026	0.150	0.864	0.995
Meconium	PWY.6167	-0.025	0.108	0.819	0.995
Meconium	PWY.6353	0.005	0.031	0.867	0.995
Meconium	PWY.6471	0.007	0.054	0.890	0.995
Meconium	PWY.6606	0.003	0.036	0.926	0.995
Meconium	PWY.6629	-0.023	0.108	0.829	0.995
Meconium	PWY.6700	0.002	0.016	0.913	0.995
Meconium	PWY.6803	-0.021	0.103	0.840	0.995
Meconium	PWY.6823	-0.008	0.060	0.899	0.995
Meconium	PWY.6891	0.014	0.096	0.883	0.995
Meconium	PWY.7184	0.005	0.048	0.909	0.995
Meconium	PWY.7197	0.008	0.055	0.879	0.995
Meconium	PWY.7198	0.005	0.053	0.918	0.995
Meconium	PWY.7221	0.001	0.011	0.890	0.995
Meconium	PWY.7228	0.005	0.046	0.922	0.995
Meconium	PWY.7234	-0.012	0.076	0.871	0.995
Meconium	PWY.7254	0.017	0.138	0.904	0.995
Meconium	PWY.7383	-0.006	0.040	0.886	0.995
Meconium	PWY.7388	0.022	0.135	0.869	0.995
Meconium	PWY0.1298	-0.011	0.077	0.891	0.995
Meconium	PWY0.162	0.003	0.028	0.926	0.995
Meconium	PWY0.881	-0.013	0.135	0.926	0.995
Meconium	PWY4LZ.257	-0.037	0.178	0.835	0.995

Exposure Window	MetaCyc Pathway ID	Effect Estimate[a]	Std. Error	p-value	q-value
Meconium	PWY66.409	-0.007	0.043	0.877	0.995
Meconium	SALVADEHYPOX.PWY	0.004	0.037	0.919	0.995
Meconium	TEICHOICACID.PWY	-0.004	0.040	0.914	0.995
Meconium	PWY66.398	-0.011	0.132	0.934	0.995
Meconium	PWY.5897	0.010	0.140	0.946	0.995
Meconium	PWY.5898	0.010	0.140	0.946	0.995
Meconium	PWY.5899	0.010	0.140	0.946	0.995
Meconium	PWY.6125	0.003	0.043	0.937	0.995
Meconium	PWY.6969	0.004	0.061	0.943	0.995
Meconium	PWY.7094	0.004	0.053	0.942	0.995
Meconium	PWY.7312	-0.006	0.093	0.949	0.995
Meconium	PWY0.166	0.003	0.038	0.937	0.995
Meconium	P108.PWY	0.013	0.214	0.953	0.995
Meconium	P185.PWY	-0.004	0.059	0.952	0.995
Meconium	PWY.4702	-0.008	0.135	0.954	0.995
Meconium	PWY.6892	0.002	0.034	0.954	0.995
Meconium	DENOVOPURINE2.PWY	-0.002	0.035	0.961	0.995
Meconium	PENTOSE.P.PWY	0.000	0.092	0.996	0.996
Meconium	PWY.5083	-0.005	0.175	0.977	0.996
Meconium	PWY.5840	-0.001	0.131	0.995	0.996
Meconium	PWY.6147	-0.001	0.087	0.994	0.996
Meconium	PWY.6507	0.004	0.091	0.964	0.995
Meconium	PWY.6545	0.001	0.046	0.984	0.996
Meconium	PWY.6901	0.000	0.078	0.996	0.996
Meconium	PWY.7211	0.000	0.041	0.996	0.996
Meconium	PWY.7220	0.002	0.041	0.966	0.995
Meconium	PWY.7222	0.002	0.041	0.966	0.995
Meconium	PWY.7539	-0.003	0.086	0.972	0.995
Meconium	PWY.841	-0.001	0.037	0.984	0.996
Meconium	PWY0.1297	-0.002	0.060	0.967	0.995
Meconium	PWY0.1586	-0.001	0.044	0.973	0.995
Cross-sectional	UNMAPPED	-0.013	0.019	0.483	0.997
Cross-sectional	UNINTEGRATED	0.004	0.006	0.477	0.997
Cross-sectional	X1CMET2.PWY	0.002	0.015	0.903	0.998
Cross-sectional	X7ALPHADEHYDROX.PWY	-0.057	0.150	0.704	0.997
Cross-sectional	ALLANTOINDEG.PWY	-0.152	0.164	0.356	0.997
Cross-sectional	ANAEROFRUCAT.PWY	0.018	0.044	0.686	0.997

Exposure Window	MetaCyc Pathway ID	Effect Estimate[a]	Std. Error	p-value	q-value
Cross-sectional	ANAGLYCOLYSIS.PWY	0.003	0.024	0.899	0.998
Cross-sectional	ARG.POLYAMINE.SYN	-0.094	0.106	0.380	0.997
Cross-sectional	ARGDEG.PWY	-0.215	0.172	0.215	0.997
Cross-sectional	ARGININE.SYN4.PWY	-0.030	0.245	0.904	0.998
Cross-sectional	ARGORNPROST.PWY	0.220	0.158	0.166	0.997
Cross-sectional	ARGSYN.PWY	0.007	0.017	0.690	0.997
Cross-sectional	ARGSYNBSUB.PWY	0.005	0.017	0.760	0.997
Cross-sectional	ARO.PWY	0.011	0.017	0.497	0.997
Cross-sectional	ASPASN.PWY	0.008	0.036	0.833	0.997
Cross-sectional	AST.PWY	-0.088	0.108	0.416	0.997
Cross-sectional	BIOTIN.BIOSYNTHESIS.PWY	0.064	0.228	0.780	0.997
Cross-sectional	BRANCHED.CHAIN.AA.SYN.PWY	0.016	0.019	0.399	0.997
Cross-sectional	CALVIN.PWY	0.010	0.024	0.674	0.997
Cross-sectional	CENTFERM.PWY	0.077	0.107	0.476	0.997
Cross-sectional	CITRULBIO.PWY	0.063	0.146	0.670	0.997
Cross-sectional	COA.PWY.1	-0.007	0.012	0.592	0.997
Cross-sectional	COA.PWY	-0.017	0.019	0.382	0.997
Cross-sectional	COBALSYN.PWY	-0.011	0.045	0.799	0.997
Cross-sectional	COLANSYN.PWY	0.074	0.051	0.153	0.997
Cross-sectional	COMPLETE.ARO.PWY	0.018	0.018	0.337	0.997
Cross-sectional	CRNFORCAT.PWY	-0.085	0.143	0.556	0.997
Cross-sectional	DAPLYSINESYN.PWY	-0.019	0.069	0.784	0.997
Cross-sectional	DENOVOPURINE2.PWY	0.017	0.056	0.757	0.997
Cross-sectional	DTDPRHAMSYN.PWY	-0.012	0.027	0.662	0.997
Cross-sectional	ENTBACSYN.PWY	-0.162	0.149	0.280	0.997
Cross-sectional	FAO.PWY	-0.120	0.159	0.451	0.997
Cross-sectional	FASYN.ELONG.PWY	-0.092	0.133	0.488	0.997
Cross-sectional	FASYN.INITIAL.PWY	-0.116	0.163	0.479	0.997
Cross-sectional	FERMENTATION.PWY	0.096	0.062	0.129	0.997
Cross-sectional	FOLSYN.PWY	0.023	0.104	0.825	0.997
Cross-sectional	FUC.RHAMCAT.PWY	-0.185	0.153	0.229	0.997
Cross-sectional	FUCCAT.PWY	-0.103	0.098	0.297	0.997
Cross-sectional	GALACT.GLUCUROCAT.PWY	-0.066	0.160	0.681	0.997
Cross-sectional	GALACTARDEG.PWY	-0.005	0.247	0.985	0.998
Cross-sectional	GALACTUROCAT.PWY	0.023	0.121	0.848	0.997
Cross-sectional	GLCMANNANAUT.PWY	-0.032	0.067	0.632	0.997
Cross-sectional	GLUCARDEG.PWY	0.049	0.235	0.837	0.997

Exposure Window	MetaCyc Pathway ID	Effect Estimate[a]	Std. Error	p-value	q-value
Cross-sectional	GLUCARGALACTSUPER.PWY	-0.005	0.247	0.985	0.998
Cross-sectional	GLUCONEO.PWY	0.022	0.027	0.406	0.997
Cross-sectional	GLUCOSE1PMETAB.PWY	-0.180	0.175	0.306	0.997
Cross-sectional	GLUCUROCAT.PWY	0.044	0.125	0.726	0.997
Cross-sectional	GLUDEG.I.PWY	-0.189	0.195	0.336	0.997
Cross-sectional	GLUTORN.PWY	0.021	0.021	0.316	0.997
Cross-sectional	GLYCOCAT.PWY	-0.178	0.166	0.289	0.997
Cross-sectional	GLYCOGENSYNTH.PWY	0.004	0.032	0.890	0.998
Cross-sectional	GLYCOL.GLYOXDEG.PWY	-0.230	0.194	0.238	0.997
Cross-sectional	GLYCOLYSIS.E.D	0.081	0.159	0.611	0.997
Cross-sectional	GLYCOLYSIS.TCA.GLYOX.BYPASS	-0.327	0.203	0.111	0.997
Cross-sectional	GLYCOLYSIS	0.026	0.052	0.613	0.997
Cross-sectional	GLYOXYLATE.BYPASS	-0.416	0.228	0.072	0.997
Cross-sectional	GOLPDLCAT.PWY	0.002	0.069	0.980	0.998
Cross-sectional	HCAMHPDEG.PWY	-0.124	0.113	0.274	0.997
Cross-sectional	HEME.BIOSYNTHESIS.II	-0.122	0.244	0.617	0.997
Cross-sectional	HEMESYN2.PWY	-0.083	0.116	0.474	0.997
Cross-sectional	HEXITOLDEGSUPER.PWY	0.055	0.045	0.225	0.997
Cross-sectional	HISDEG.PWY	0.079	0.126	0.529	0.997
Cross-sectional	HISTSYN.PWY	-0.001	0.017	0.963	0.998
Cross-sectional	HOMOSER.METSYN.PWY	-0.020	0.075	0.792	0.997
Cross-sectional	HSERMETANA.PWY	-0.003	0.028	0.920	0.998
Cross-sectional	ILEUSYN.PWY	0.019	0.018	0.287	0.997
Cross-sectional	KETOGLUCONMET.PWY	0.158	0.344	0.648	0.997
Cross-sectional	LACTOSECAT.PWY	-0.041	0.103	0.696	0.997
Cross-sectional	MET.SAM.PWY	-0.014	0.059	0.811	0.997
Cross-sectional	METH.ACETATE.PWY	0.028	0.059	0.642	0.997
Cross-sectional	METHANOGENESIS.PWY	0.152	0.274	0.580	0.997
Cross-sectional	METHGLYUT.PWY	-0.052	0.201	0.798	0.997
Cross-sectional	METSYN.PWY	-0.017	0.063	0.784	0.997
Cross-sectional	NAD.BIOSYNTHESIS.II	0.375	0.344	0.279	0.997
Cross-sectional	NAGLIPASYN.PWY	0.141	0.283	0.620	0.997
Cross-sectional	NONMEVIPP.PWY	-0.009	0.094	0.926	0.998
Cross-sectional	NONOXIPENT.PWY	0.008	0.035	0.817	0.997
Cross-sectional	OANTIGEN.PWY	0.013	0.030	0.674	0.997
Cross-sectional	ORNARGDEG.PWY	-0.215	0.172	0.215	0.997
Cross-sectional	ORNDEG.PWY	-0.125	0.098	0.206	0.997

Exposure Window	MetaCyc Pathway ID	Effect Estimate[a]	Std. Error	p-value	q-value
Cross-sectional	P105.PWY	-0.307	0.198	0.124	0.997
Cross-sectional	P108.PWY	0.014	0.258	0.957	0.998
Cross-sectional	P122.PWY	0.072	0.288	0.805	0.997
Cross-sectional	P124.PWY	0.067	0.160	0.675	0.997
Cross-sectional	P125.PWY	0.289	0.299	0.337	0.997
Cross-sectional	P161.PWY	-0.129	0.229	0.576	0.997
Cross-sectional	P162.PWY	0.115	0.141	0.418	0.997
Cross-sectional	P164.PWY	-0.012	0.065	0.854	0.997
Cross-sectional	P185.PWY	0.046	0.077	0.550	0.997
Cross-sectional	P221.PWY	-0.240	0.286	0.404	0.997
Cross-sectional	P23.PWY	-0.122	0.236	0.606	0.997
Cross-sectional	P4.PWY	0.033	0.096	0.735	0.997
Cross-sectional	P42.PWY	-0.238	0.142	0.097	0.997
Cross-sectional	P441.PWY	0.020	0.091	0.830	0.997
Cross-sectional	P461.PWY	0.042	0.077	0.588	0.997
Cross-sectional	P562.PWY	0.156	0.117	0.188	0.997
Cross-sectional	P621.PWY	0.064	0.201	0.752	0.997
Cross-sectional	PANTO.PWY	-0.011	0.036	0.762	0.997
Cross-sectional	PANTOSYN.PWY	-0.007	0.029	0.809	0.997
Cross-sectional	PENTOSE.P.PWY	0.006	0.112	0.959	0.998
Cross-sectional	PEPTIDOGLYCANSYN.PWY	0.003	0.012	0.794	0.997
Cross-sectional	PHOSLIPSYN.PWY	0.035	0.083	0.671	0.997
Cross-sectional	POLYAMINSYN3.PWY	0.260	0.217	0.234	0.997
Cross-sectional	POLYAMSYN.PWY	-0.093	0.115	0.421	0.997
Cross-sectional	POLYISOPRENSYN.PWY	0.126	0.192	0.512	0.997
Cross-sectional	PPGPPMET.PWY	0.020	0.062	0.742	0.997
Cross-sectional	PROPFERM.PWY	0.163	0.088	0.067	0.997
Cross-sectional	PRPP.PWY	0.029	0.077	0.709	0.997
Cross-sectional	PWY.1042	0.009	0.019	0.631	0.997
Cross-sectional	PWY.1269	-0.152	0.135	0.264	0.997
Cross-sectional	PWY.1861	0.003	0.075	0.967	0.998
Cross-sectional	PWY.241	0.068	0.082	0.408	0.997
Cross-sectional	PWY.2723	-0.175	0.176	0.324	0.997
Cross-sectional	PWY.2941	0.023	0.084	0.782	0.997
Cross-sectional	PWY.2942	-0.013	0.016	0.425	0.997
Cross-sectional	PWY.3001	0.010	0.020	0.625	0.997
Cross-sectional	PWY.3781	-0.035	0.189	0.855	0.997

Exposure Window	MetaCyc Pathway ID	Effect Estimate[a]	Std. Error	p-value	q-value
Cross-sectional	PWY.3841	0.000	0.012	0.992	0.998
Cross-sectional	PWY.4041	-0.018	0.058	0.754	0.997
Cross-sectional	PWY.4242	-0.014	0.023	0.550	0.997
Cross-sectional	PWY.4702	0.101	0.207	0.627	0.997
Cross-sectional	PWY.4722	0.026	0.170	0.881	0.998
Cross-sectional	PWY.4981	-0.135	0.086	0.122	0.997
Cross-sectional	PWY.4984	0.066	0.148	0.656	0.997
Cross-sectional	PWY.5004	-0.147	0.128	0.255	0.997
Cross-sectional	PWY.5005	0.058	0.250	0.818	0.997
Cross-sectional	PWY.5022	-0.161	0.160	0.319	0.997
Cross-sectional	PWY.5030	0.141	0.148	0.345	0.997
Cross-sectional	PWY.5083	-0.206	0.262	0.434	0.997
Cross-sectional	PWY.5097	0.000	0.015	0.981	0.998
Cross-sectional	PWY.5100	0.035	0.034	0.306	0.997
Cross-sectional	PWY.5101	0.020	0.321	0.951	0.998
Cross-sectional	PWY.5103	0.015	0.021	0.473	0.997
Cross-sectional	PWY.5104	0.057	0.063	0.368	0.997
Cross-sectional	PWY.5121	0.067	0.100	0.502	0.997
Cross-sectional	PWY.5136	-0.125	0.163	0.445	0.997
Cross-sectional	PWY.5138	0.095	0.248	0.703	0.997
Cross-sectional	PWY.5154	-0.126	0.099	0.208	0.997
Cross-sectional	PWY.5173	-0.361	0.261	0.170	0.997
Cross-sectional	PWY.5177	-0.041	0.062	0.510	0.997
Cross-sectional	PWY.5188	0.026	0.046	0.573	0.997
Cross-sectional	PWY.5189	0.008	0.197	0.966	0.998
Cross-sectional	PWY.5198	0.114	0.256	0.659	0.997
Cross-sectional	PWY.5265	-0.006	0.177	0.972	0.998
Cross-sectional	PWY.5304	0.136	0.106	0.205	0.997
Cross-sectional	PWY.5345	-0.057	0.226	0.800	0.997
Cross-sectional	PWY.5347	-0.007	0.057	0.897	0.998
Cross-sectional	PWY.5367	0.007	0.220	0.974	0.998
Cross-sectional	PWY.5384	-0.007	0.122	0.957	0.998
Cross-sectional	PWY.5392	0.030	0.118	0.801	0.997
Cross-sectional	PWY.5464	-0.390	0.194	0.048	0.997
Cross-sectional	PWY.5484	0.020	0.059	0.741	0.997
Cross-sectional	PWY.5505	0.080	0.221	0.719	0.997
Cross-sectional	PWY.561	-0.347	0.222	0.122	0.997

Exposure Window	MetaCyc Pathway ID	Effect Estimate[a]	Std. Error	p-value	q-value
Cross-sectional	PWY.5656	-0.108	0.145	0.459	0.997
Cross-sectional	PWY.5659	0.102	0.056	0.073	0.997
Cross-sectional	PWY.5667	0.009	0.025	0.717	0.997
Cross-sectional	PWY.5675	-0.253	0.221	0.256	0.997
Cross-sectional	PWY.5676	0.000	0.065	0.998	0.998
Cross-sectional	PWY.5677	-0.025	0.132	0.851	0.997
Cross-sectional	PWY.5686	0.005	0.014	0.733	0.997
Cross-sectional	PWY.5690	-0.083	0.062	0.185	0.997
Cross-sectional	PWY.5695	0.011	0.020	0.597	0.997
Cross-sectional	PWY.5723	-0.394	0.180	0.031	0.997
Cross-sectional	PWY.5747	-0.284	0.216	0.192	0.997
Cross-sectional	PWY.5791	-0.314	0.204	0.127	0.997
Cross-sectional	PWY.5837	-0.314	0.204	0.127	0.997
Cross-sectional	PWY.5838	-0.357	0.202	0.081	0.997
Cross-sectional	PWY.5840	-0.356	0.185	0.059	0.997
Cross-sectional	PWY.5845	-0.047	0.213	0.826	0.997
Cross-sectional	PWY.5850	0.034	0.123	0.782	0.997
Cross-sectional	PWY.5860	0.035	0.124	0.779	0.997
Cross-sectional	PWY.5861	-0.363	0.205	0.080	0.997
Cross-sectional	PWY.5862	-0.047	0.215	0.826	0.997
Cross-sectional	PWY.5863	-0.298	0.203	0.146	0.997
Cross-sectional	PWY.5896	0.034	0.123	0.782	0.997
Cross-sectional	PWY.5897	-0.316	0.197	0.114	0.997
Cross-sectional	PWY.5898	-0.316	0.197	0.114	0.997
Cross-sectional	PWY.5899	-0.316	0.197	0.114	0.997
Cross-sectional	PWY.5910	-0.048	0.119	0.691	0.997
Cross-sectional	PWY.5913	0.062	0.103	0.548	0.997
Cross-sectional	PWY.5918	-0.043	0.232	0.852	0.997
Cross-sectional	PWY.5920	-0.208	0.151	0.173	0.997
Cross-sectional	PWY.5941	0.066	0.261	0.800	0.997
Cross-sectional	PWY.5971	-0.026	0.154	0.865	0.997
Cross-sectional	PWY.5973	-0.087	0.110	0.433	0.997
Cross-sectional	PWY.5989	-0.140	0.126	0.270	0.997
Cross-sectional	PWY.5994	0.290	0.247	0.243	0.997
Cross-sectional	PWY.6113	-0.001	0.145	0.993	0.998
Cross-sectional	PWY.6121	-0.001	0.019	0.967	0.998
Cross-sectional	PWY.6122	0.009	0.017	0.581	0.997

Exposure Window	MetaCyc Pathway ID	Effect Estimate[a]	Std. Error	p-value	q-value
Cross-sectional	PWY.6123	-0.012	0.023	0.602	0.997
Cross-sectional	PWY.6124	-0.016	0.024	0.514	0.997
Cross-sectional	PWY.6125	0.019	0.070	0.783	0.997
Cross-sectional	PWY.6126	-0.009	0.030	0.761	0.997
Cross-sectional	PWY.6143	-0.101	0.117	0.388	0.997
Cross-sectional	PWY.6147	0.105	0.130	0.424	0.997
Cross-sectional	PWY.6151	0.022	0.020	0.288	0.997
Cross-sectional	PWY.6163	0.017	0.018	0.365	0.997
Cross-sectional	PWY.6167	0.127	0.256	0.621	0.997
Cross-sectional	PWY.6168	0.023	0.040	0.576	0.997
Cross-sectional	PWY.621	0.060	0.058	0.300	0.997
Cross-sectional	PWY.622	-0.117	0.168	0.489	0.997
Cross-sectional	PWY.6263	-0.059	0.188	0.753	0.997
Cross-sectional	PWY.6270	-0.013	0.125	0.916	0.998
Cross-sectional	PWY.6277	0.009	0.017	0.581	0.997
Cross-sectional	PWY.6282	-0.102	0.138	0.463	0.997
Cross-sectional	PWY.6284	0.039	0.161	0.808	0.997
Cross-sectional	PWY.6285	0.006	0.152	0.970	0.998
Cross-sectional	PWY.6305	0.007	0.061	0.915	0.998
Cross-sectional	PWY.6317	0.020	0.040	0.620	0.997
Cross-sectional	PWY.6318	0.010	0.118	0.930	0.998
Cross-sectional	PWY.6349	0.051	0.177	0.774	0.997
Cross-sectional	PWY.6353	-0.023	0.049	0.635	0.997
Cross-sectional	PWY.6385	0.000	0.014	0.985	0.998
Cross-sectional	PWY.6386	0.004	0.013	0.757	0.997
Cross-sectional	PWY.6387	0.006	0.012	0.612	0.997
Cross-sectional	PWY.6435	-0.032	0.245	0.896	0.998
Cross-sectional	PWY.6470	-0.004	0.082	0.957	0.998
Cross-sectional	PWY.6471	-0.009	0.066	0.890	0.998
Cross-sectional	PWY.6507	-0.001	0.124	0.996	0.998
Cross-sectional	PWY.6519	0.065	0.235	0.783	0.997
Cross-sectional	PWY.6527	0.030	0.045	0.506	0.997
Cross-sectional	PWY.6531	-0.142	0.243	0.562	0.997
Cross-sectional	PWY.6545	0.027	0.070	0.702	0.997
Cross-sectional	PWY.6549	0.091	0.053	0.090	0.997
Cross-sectional	PWY.6562	-0.077	0.124	0.536	0.997
Cross-sectional	PWY.6572	0.199	0.092	0.033	0.997

Exposure Window	MetaCyc Pathway ID	Effect Estimate[a]	Std. Error	p-value	q-value
Cross-sectional	PWY.6588	-0.106	0.197	0.589	0.997
Cross-sectional	PWY.6590	0.076	0.105	0.472	0.997
Cross-sectional	PWY.6595	-0.022	0.212	0.917	0.998
Cross-sectional	PWY.6606	-0.036	0.055	0.517	0.997
Cross-sectional	PWY.6608	-0.024	0.055	0.671	0.997
Cross-sectional	PWY.6609	-0.004	0.025	0.864	0.997
Cross-sectional	PWY.6612	0.023	0.106	0.830	0.997
Cross-sectional	PWY.6628	-0.401	0.226	0.081	0.997
Cross-sectional	PWY.6629	-0.316	0.173	0.072	0.997
Cross-sectional	PWY.6630	-0.317	0.172	0.070	0.997
Cross-sectional	PWY.6690	-0.124	0.113	0.274	0.997
Cross-sectional	PWY.6700	-0.004	0.023	0.866	0.997
Cross-sectional	PWY.6703	0.012	0.048	0.807	0.997
Cross-sectional	PWY.6731	-0.150	0.148	0.315	0.997
Cross-sectional	PWY.6737	0.010	0.023	0.661	0.997
Cross-sectional	PWY.6749	0.045	0.165	0.786	0.997
Cross-sectional	PWY.6803	-0.267	0.176	0.133	0.997
Cross-sectional	PWY.6823	-0.150	0.215	0.486	0.997
Cross-sectional	PWY.6837	-0.089	0.098	0.370	0.997
Cross-sectional	PWY.6859	0.255	0.257	0.324	0.997
Cross-sectional	PWY.6876	0.067	0.164	0.683	0.997
Cross-sectional	PWY.6891	0.059	0.116	0.613	0.997
Cross-sectional	PWY.6892	0.015	0.047	0.743	0.997
Cross-sectional	PWY.6895	0.056	0.145	0.702	0.997
Cross-sectional	PWY.6897	0.049	0.054	0.369	0.997
Cross-sectional	PWY.6901	-0.026	0.093	0.784	0.997
Cross-sectional	PWY.6936	0.028	0.031	0.375	0.997
Cross-sectional	PWY.6969	-0.137	0.080	0.092	0.997
Cross-sectional	PWY.7003	-0.052	0.176	0.767	0.997
Cross-sectional	PWY.7013	0.038	0.081	0.640	0.997
Cross-sectional	PWY.7046	0.057	0.187	0.761	0.997
Cross-sectional	PWY.7094	0.001	0.122	0.994	0.998
Cross-sectional	PWY.7111	0.020	0.018	0.287	0.997
Cross-sectional	PWY.7115	0.111	0.099	0.267	0.997
Cross-sectional	PWY.7117	0.056	0.095	0.554	0.997
Cross-sectional	PWY.7184	0.018	0.076	0.815	0.997
Cross-sectional	PWY.7187	0.013	0.060	0.835	0.997

Exposure Window	MetaCyc Pathway ID	Effect Estimate[a]	Std. Error	p-value	q-value
Cross-sectional	PWY.7196	0.022	0.055	0.697	0.997
Cross-sectional	PWY.7197	0.024	0.085	0.777	0.997
Cross-sectional	PWY.7198	0.006	0.081	0.937	0.998
Cross-sectional	PWY.7199	-0.033	0.030	0.280	0.997
Cross-sectional	PWY.7204	0.050	0.131	0.702	0.997
Cross-sectional	PWY.7208	0.012	0.062	0.848	0.997
Cross-sectional	PWY.7209	0.253	0.231	0.278	0.997
Cross-sectional	PWY.7210	-0.003	0.254	0.989	0.998
Cross-sectional	PWY.7211	-0.010	0.061	0.865	0.997
Cross-sectional	PWY.7219	-0.004	0.017	0.830	0.997
Cross-sectional	PWY.7220	-0.012	0.055	0.834	0.997
Cross-sectional	PWY.7221	0.007	0.013	0.588	0.997
Cross-sectional	PWY.7222	-0.012	0.055	0.834	0.997
Cross-sectional	PWY.7228	0.020	0.073	0.783	0.997
Cross-sectional	PWY.7229	-0.004	0.021	0.840	0.997
Cross-sectional	PWY.7234	0.082	0.099	0.411	0.997
Cross-sectional	PWY.7237	0.013	0.053	0.811	0.997
Cross-sectional	PWY.7242	0.049	0.138	0.724	0.997
Cross-sectional	PWY.724	0.004	0.013	0.727	0.997
Cross-sectional	PWY.7254	-0.237	0.208	0.257	0.997
Cross-sectional	PWY.7282	0.033	0.199	0.870	0.998
Cross-sectional	PWY.7286	0.086	0.212	0.688	0.997
Cross-sectional	PWY.7288	0.133	0.186	0.476	0.997
Cross-sectional	PWY.7312	-0.090	0.155	0.564	0.997
Cross-sectional	PWY.7315	0.123	0.165	0.459	0.997
Cross-sectional	PWY.7316	-0.163	0.168	0.334	0.997
Cross-sectional	PWY.7323	0.167	0.104	0.114	0.997
Cross-sectional	PWY.7328	-0.169	0.161	0.298	0.997
Cross-sectional	PWY.7332	-0.006	0.214	0.978	0.998
Cross-sectional	PWY.7357	-0.001	0.030	0.982	0.998
Cross-sectional	PWY.7371	-0.275	0.216	0.207	0.997
Cross-sectional	PWY.7383	0.006	0.058	0.915	0.998
Cross-sectional	PWY.7385	0.032	0.180	0.860	0.997
Cross-sectional	PWY.7388	-0.117	0.166	0.481	0.997
Cross-sectional	PWY.7392	0.253	0.235	0.285	0.997
Cross-sectional	PWY.7400	0.007	0.017	0.681	0.997
Cross-sectional	PWY.7456	0.112	0.077	0.148	0.997

Exposure Window	MetaCyc Pathway ID	Effect Estimate[a]	Std. Error	p-value	q-value
Cross-sectional	PWY.7539	0.105	0.127	0.408	0.997
Cross-sectional	PWY.7560	-0.013	0.129	0.917	0.998
Cross-sectional	PWY.7616	-0.115	0.259	0.659	0.997
Cross-sectional	PWY.7663	-0.077	0.121	0.526	0.997
Cross-sectional	PWY.7664	-0.087	0.141	0.539	0.997
Cross-sectional	PWY.821	-0.013	0.224	0.954	0.998
Cross-sectional	PWY.841	0.020	0.060	0.746	0.997
Cross-sectional	PWY.922	-0.059	0.149	0.693	0.997
Cross-sectional	PWY0.1061	0.146	0.128	0.257	0.997
Cross-sectional	PWY0.1241	-0.133	0.218	0.544	0.997
Cross-sectional	PWY0.1261	0.187	0.156	0.234	0.997
Cross-sectional	PWY0.1277	-0.100	0.092	0.281	0.997
Cross-sectional	PWY0.1296	0.002	0.037	0.965	0.998
Cross-sectional	PWY0.1297	0.052	0.096	0.588	0.997
Cross-sectional	PWY0.1298	0.021	0.123	0.863	0.997
Cross-sectional	PWY0.1319	0.009	0.025	0.718	0.997
Cross-sectional	PWY0.1415	-0.027	0.241	0.912	0.998
Cross-sectional	PWY0.1479	-0.030	0.072	0.681	0.997
Cross-sectional	PWY0.1586	-0.023	0.057	0.690	0.997
Cross-sectional	PWY0.162	0.016	0.045	0.720	0.997
Cross-sectional	PWY0.166	0.016	0.061	0.800	0.997
Cross-sectional	PWY0.42	-0.155	0.150	0.304	0.997
Cross-sectional	PWY0.781	0.045	0.091	0.621	0.997
Cross-sectional	PWY0.845	-0.106	0.224	0.638	0.997
Cross-sectional	PWY0.862	-0.089	0.145	0.538	0.997
Cross-sectional	PWY0.881	-0.009	0.247	0.969	0.998
Cross-sectional	PWY3DJ.35471	-0.082	0.112	0.466	0.997
Cross-sectional	PWY3O.355	0.086	0.199	0.668	0.997
Cross-sectional	PWY490.3	-0.127	0.110	0.251	0.997
Cross-sectional	PWY4FS.7	0.034	0.095	0.722	0.997
Cross-sectional	PWY4FS.8	0.034	0.095	0.722	0.997
Cross-sectional	PWY4LZ.257	-0.128	0.227	0.574	0.997
Cross-sectional	PWY66.389	-0.233	0.343	0.498	0.997
Cross-sectional	PWY66.391	0.117	0.179	0.516	0.997
Cross-sectional	PWY66.398	0.066	0.167	0.694	0.997
Cross-sectional	PWY66.399	0.009	0.048	0.852	0.997
Cross-sectional	PWY66.400	0.029	0.060	0.631	0.997

Exposure Window	MetaCyc Pathway ID	Effect Estimate[a]	Std. Error	p-value	q-value
Cross-sectional	PWY66.409	0.045	0.074	0.549	0.997
Cross-sectional	PWY66.422	0.006	0.028	0.839	0.997
Cross-sectional	PWYG.321	-0.093	0.143	0.520	0.997
Cross-sectional	PYRIDNUCSAL.PWY	0.138	0.161	0.396	0.997
Cross-sectional	PYRIDNUCSYN.PWY	-0.008	0.022	0.722	0.997
Cross-sectional	PYRIDOXSYN.PWY	-0.128	0.233	0.585	0.997
Cross-sectional	REDCITCYC	-0.227	0.222	0.308	0.997
Cross-sectional	RHAMCAT.PWY	-0.031	0.075	0.679	0.997
Cross-sectional	RIBOSYN2.PWY	0.015	0.038	0.699	0.997
Cross-sectional	RUMP.PWY	0.238	0.217	0.274	0.997
Cross-sectional	SALVADEHYPOX.PWY	-0.038	0.058	0.512	0.997
Cross-sectional	SER.GLYSYN.PWY	-0.005	0.024	0.837	0.997
Cross-sectional	SO4ASSIM.PWY	-0.069	0.238	0.772	0.997
Cross-sectional	SULFATE.CYS.PWY	-0.060	0.228	0.795	0.997
Cross-sectional	TCA.GLYOX.BYPASS	-0.339	0.207	0.105	0.997
Cross-sectional	TCA	-0.064	0.059	0.285	0.997
Cross-sectional	TEICHOICACID.PWY	-0.034	0.058	0.560	0.997
Cross-sectional	THISYN.PWY	-0.046	0.082	0.575	0.997
Cross-sectional	THISYNARA.PWY	0.062	0.055	0.262	0.997
Cross-sectional	THREOCAT.PWY	-0.119	0.126	0.352	0.997
Cross-sectional	THRESYN.PWY	0.006	0.024	0.803	0.997
Cross-sectional	TRNA.CHARGING.PWY	0.001	0.012	0.926	0.998
Cross-sectional	TRPSYN.PWY	0.066	0.036	0.067	0.997
Cross-sectional	UDPNAGSYN.PWY	0.024	0.047	0.606	0.997
Cross-sectional	VALSYN.PWY	0.019	0.018	0.287	0.997
Meconium Adj.[b]	PWY.5677	0.353	0.080	0.000	0.027
Meconium Adj.[b]	THISYN.PWY	0.164	0.061	0.010	0.998
Meconium Adj.[b]	POLYAMINSYN3.PWY	-0.360	0.139	0.013	0.998
Meconium Adj.[b]	PWY.4041	-0.094	0.038	0.018	0.998
Meconium Adj.[b]	PWY.922	-0.276	0.110	0.016	0.998
Meconium Adj.[b]	HOMOSER.METSYN.PWY	-0.111	0.050	0.031	0.998
Meconium Adj.[b]	P621.PWY	-0.308	0.136	0.029	0.998
Meconium Adj.[b]	BIOTIN.BIOSYNTHESIS.PWY	0.319	0.163	0.057	0.998
Meconium Adj.[b]	ILEUSYN.PWY	-0.028	0.014	0.056	0.998
Meconium Adj.[b]	MET.SAM.PWY	-0.082	0.039	0.043	0.998
Meconium Adj.[b]	METSYN.PWY	-0.090	0.042	0.036	0.998
Meconium Adj.[b]	P125.PWY	-0.438	0.214	0.047	0.998

Exposure Window	MetaCyc Pathway ID	Effect Estimate[a]	Std. Error	p-value	q-value
Meconium Adj.[b]	PROPFERM.PWY	0.099	0.047	0.042	0.998
Meconium Adj.[b]	PWY.5347	-0.080	0.037	0.035	0.998
Meconium Adj.[b]	PWY.5695	0.031	0.015	0.039	0.998
Meconium Adj.[b]	PWY.6396	-0.159	0.077	0.045	0.998
Meconium Adj.[b]	PWY.7111	-0.029	0.015	0.058	0.998
Meconium Adj.[b]	PWY.821	-0.322	0.163	0.054	0.998
Meconium Adj.[b]	PWY66.391	0.238	0.117	0.047	0.998
Meconium Adj.[b]	VALSYN.PWY	-0.028	0.014	0.056	0.998
Meconium Adj.[b]	PWY.6519	0.324	0.168	0.061	0.998
Meconium Adj.[b]	PWY.6737	-0.029	0.015	0.064	0.998
Meconium Adj.[b]	PWY.7288	0.226	0.119	0.065	0.998
Meconium Adj.[b]	PWY.6263	0.254	0.135	0.067	0.998
Meconium Adj.[b]	HISDEG.PWY	0.177	0.097	0.076	0.998
Meconium Adj.[b]	PWY.3001	-0.026	0.014	0.074	0.998
Meconium Adj.[b]	KETOGLUCONMET.PWY	-0.399	0.223	0.081	0.998
Meconium Adj.[b]	ASPASN.PWY	-0.041	0.025	0.108	0.998
Meconium Adj.[b]	GLYCOLYSIS	-0.061	0.036	0.098	0.998
Meconium Adj.[b]	HISTSYN.PWY	-0.024	0.014	0.107	0.998
Meconium Adj.[b]	PWY.5100	-0.041	0.024	0.089	0.998
Meconium Adj.[b]	PWY.5910	-0.171	0.096	0.083	0.998
Meconium Adj.[b]	PWY.6318	0.117	0.071	0.108	0.998
Meconium Adj.[b]	PWY.7385	-0.198	0.117	0.098	0.998
Meconium Adj.[b]	PWY0.1061	-0.145	0.086	0.100	0.998
Meconium Adj.[b]	PWY0.1479	-0.076	0.046	0.108	0.998
Meconium Adj.[b]	GLUCONEO.PWY	-0.034	0.021	0.112	0.998
Meconium Adj.[b]	PWY.5136	0.186	0.114	0.111	0.998
Meconium Adj.[b]	FAO.PWY	0.178	0.111	0.116	0.998
Meconium Adj.[b]	BRANCHED.CHAIN.AA.SYN.PWY	-0.024	0.015	0.126	0.998
Meconium Adj.[b]	PWY.5103	-0.025	0.017	0.135	0.998
Meconium Adj.[b]	PWY.7199	0.034	0.023	0.139	0.998
Meconium Adj.[b]	PWY0.1241	0.260	0.172	0.139	0.998
Meconium Adj.[b]	THRESYN.PWY	-0.025	0.017	0.135	0.998
Meconium Adj.[b]	ARGININE.SYN4.PWY	0.244	0.166	0.149	0.998
Meconium Adj.[b]	COA.PWY.1	-0.013	0.009	0.152	0.998
Meconium Adj.[b]	GLYCOLYSIS.E.D	0.174	0.120	0.155	0.998
Meconium Adj.[b]	METHGLYUT.PWY	0.220	0.148	0.145	0.998
Meconium Adj.[b]	PWY.5138	0.220	0.152	0.155	0.998

Exposure Window	MetaCyc Pathway ID	Effect Estimate[a]	Std. Error	p-value	q-value
Meconium Adj.[b]	PWY.5505	0.223	0.154	0.156	0.998
Meconium Adj.[b]	PWY.5022	0.159	0.112	0.165	0.998
Meconium Adj.[b]	UNMAPPED	0.015	0.015	0.318	0.998
Meconium Adj.[b]	UNINTEGRATED	-0.005	0.004	0.276	0.998
Meconium Adj.[b]	X1CMET2.PWY	-0.004	0.011	0.680	0.998
Meconium Adj.[b]	X7ALPHADEHYDROX.PWY	0.162	0.130	0.222	0.998
Meconium Adj.[b]	ALLANTOINDEG.PWY	0.040	0.092	0.666	0.998
Meconium Adj.[b]	ANAEROFRUCAT.PWY	-0.028	0.031	0.380	0.998
Meconium Adj.[b]	ANAGLYCOLYSIS.PWY	-0.017	0.019	0.368	0.998
Meconium Adj.[b]	ARG.POLYAMINE.SYN	-0.088	0.083	0.295	0.998
Meconium Adj.[b]	ARGDEG.PWY	-0.095	0.113	0.405	0.998
Meconium Adj.[b]	ARGORNPROST.PWY	0.067	0.102	0.515	0.998
Meconium Adj.[b]	ARGSYN.PWY	-0.016	0.014	0.270	0.998
Meconium Adj.[b]	ARGSYNBSUB.PWY	-0.017	0.014	0.235	0.998
Meconium Adj.[b]	ARO.PWY	-0.015	0.014	0.283	0.998
Meconium Adj.[b]	CALVIN.PWY	-0.014	0.019	0.445	0.998
Meconium Adj.[b]	CENTFERM.PWY	-0.057	0.089	0.523	0.998
Meconium Adj.[b]	CITRULBIO.PWY	0.139	0.111	0.218	0.998
Meconium Adj.[b]	COA.PWY	-0.015	0.014	0.290	0.998
Meconium Adj.[b]	COBALSYN.PWY	-0.024	0.029	0.424	0.998
Meconium Adj.[b]	COLANSYN.PWY	-0.008	0.033	0.812	0.998
Meconium Adj.[b]	COMPLETE.ARO.PWY	-0.017	0.015	0.268	0.998
Meconium Adj.[b]	DAPLYSINESYN.PWY	-0.037	0.051	0.466	0.998
Meconium Adj.[b]	DTDPRHAMSYN.PWY	-0.017	0.021	0.409	0.998
Meconium Adj.[b]	ENTBACSYN.PWY	0.029	0.061	0.638	0.998
Meconium Adj.[b]	FASYN.ELONG.PWY	0.036	0.111	0.747	0.998
Meconium Adj.[b]	FERMENTATION.PWY	0.011	0.037	0.779	0.998
Meconium Adj.[b]	FOLSYN.PWY	0.063	0.087	0.471	0.998
Meconium Adj.[b]	FUC.RHAMCAT.PWY	0.060	0.103	0.565	0.998
Meconium Adj.[b]	FUCCAT.PWY	0.070	0.074	0.350	0.998
Meconium Adj.[b]	GALACT.GLUCUROCAT.PWY	0.061	0.107	0.572	0.998
Meconium Adj.[b]	GALACTARDEG.PWY	0.187	0.177	0.297	0.998
Meconium Adj.[b]	GLCMANNANAUT.PWY	-0.025	0.046	0.599	0.998
Meconium Adj.[b]	GLUCARDEG.PWY	0.166	0.165	0.319	0.998
Meconium Adj.[b]	GLUCARGALACTSUPER.PWY	0.187	0.177	0.297	0.998
Meconium Adj.[b]	GLUCOSE1PMETAB.PWY	-0.099	0.129	0.447	0.998
Meconium Adj.[b]	GLUDEG.I.PWY	0.149	0.128	0.250	0.998

Exposure Window	MetaCyc Pathway ID	Effect Estimate[a]	Std. Error	p-value	q-value
Meconium Adj.[b]	GLUTORN.PWY	-0.024	0.018	0.190	0.998
Meconium Adj.[b]	GLYCOCAT.PWY	-0.091	0.123	0.462	0.998
Meconium Adj.[b]	GLYCOGENSYNTH.PWY	-0.018	0.024	0.444	0.998
Meconium Adj.[b]	GLYCOL.GLYOXDEG.PWY	-0.035	0.132	0.790	0.998
Meconium Adj.[b]	GLYCOLYSIS.TCA.GLYOX.BYPASS	-0.037	0.134	0.781	0.998
Meconium Adj.[b]	GLYOXYLATE.BYPASS	-0.120	0.161	0.460	0.998
Meconium Adj.[b]	GOLPDLCAT.PWY	0.014	0.052	0.797	0.998
Meconium Adj.[b]	HCAMHPDEG.PWY	-0.033	0.067	0.629	0.998
Meconium Adj.[b]	HEME.BIOSYNTHESIS.II	0.077	0.156	0.625	0.998
Meconium Adj.[b]	HSERMETANA.PWY	-0.021	0.022	0.342	0.998
Meconium Adj.[b]	LACTOSECAT.PWY	-0.089	0.079	0.263	0.998
Meconium Adj.[b]	METH.ACETATE.PWY	-0.056	0.047	0.238	0.998
Meconium Adj.[b]	METHANOGENESIS.PWY	-0.046	0.124	0.712	0.998
Meconium Adj.[b]	NAD.BIOSYNTHESIS.II	-0.124	0.227	0.589	0.998
Meconium Adj.[b]	NAGLIPASYN.PWY	0.056	0.160	0.727	0.998
Meconium Adj.[b]	NONMEVIPP.PWY	-0.023	0.069	0.742	0.998
Meconium Adj.[b]	NONOXIPENT.PWY	-0.019	0.027	0.492	0.998
Meconium Adj.[b]	OANTIGEN.PWY	-0.028	0.022	0.199	0.998
Meconium Adj.[b]	ORNARGDEG.PWY	-0.095	0.113	0.405	0.998
Meconium Adj.[b]	ORNDEG.PWY	-0.083	0.067	0.222	0.998
Meconium Adj.[b]	P105.PWY	-0.055	0.135	0.687	0.998
Meconium Adj.[b]	P122.PWY	-0.252	0.209	0.233	0.998
Meconium Adj.[b]	P124.PWY	0.083	0.126	0.513	0.998
Meconium Adj.[b]	P161.PWY	-0.083	0.182	0.651	0.998
Meconium Adj.[b]	P162.PWY	0.037	0.085	0.662	0.998
Meconium Adj.[b]	P164.PWY	0.034	0.052	0.516	0.998
Meconium Adj.[b]	P221.PWY	-0.106	0.142	0.460	0.998
Meconium Adj.[b]	P4.PWY	-0.047	0.069	0.502	0.998
Meconium Adj.[b]	P42.PWY	0.053	0.101	0.601	0.998
Meconium Adj.[b]	P441.PWY	0.023	0.068	0.742	0.998
Meconium Adj.[b]	P461.PWY	0.013	0.052	0.811	0.998
Meconium Adj.[b]	PANTO.PWY	-0.020	0.022	0.363	0.998
Meconium Adj.[b]	PANTOSYN.PWY	-0.018	0.018	0.332	0.998
Meconium Adj.[b]	PEPTIDOGLYCANSYN.PWY	-0.004	0.009	0.695	0.998
Meconium Adj.[b]	PHOSLIPSYN.PWY	0.021	0.068	0.760	0.998
Meconium Adj.[b]	POLYAMSYN.PWY	-0.095	0.087	0.285	0.998
Meconium Adj.[b]	PRPP.PWY	0.038	0.071	0.600	0.998

Exposure Window	MetaCyc Pathway ID	Effect Estimate[a]	Std. Error	p-value	q-value
Meconium Adj.[b]	PWY.1042	-0.013	0.015	0.387	0.998
Meconium Adj.[b]	PWY.1269	0.131	0.102	0.204	0.998
Meconium Adj.[b]	PWY.1861	-0.034	0.060	0.572	0.998
Meconium Adj.[b]	PWY.241	-0.052	0.055	0.353	0.998
Meconium Adj.[b]	PWY.2723	-0.100	0.130	0.446	0.998
Meconium Adj.[b]	PWY.2942	-0.007	0.011	0.543	0.998
Meconium Adj.[b]	PWY.3781	-0.113	0.153	0.466	0.998
Meconium Adj.[b]	PWY.3841	-0.003	0.009	0.770	0.998
Meconium Adj.[b]	PWY.4242	-0.019	0.017	0.255	0.998
Meconium Adj.[b]	PWY.4722	-0.131	0.130	0.320	0.998
Meconium Adj.[b]	PWY.4984	0.145	0.112	0.203	0.998
Meconium Adj.[b]	PWY.5005	0.132	0.185	0.478	0.998
Meconium Adj.[b]	PWY.5030	0.151	0.111	0.181	0.998
Meconium Adj.[b]	PWY.5083	-0.043	0.178	0.812	0.998
Meconium Adj.[b]	PWY.5088	0.056	0.043	0.205	0.998
Meconium Adj.[b]	PWY.5097	-0.008	0.011	0.497	0.998
Meconium Adj.[b]	PWY.5104	0.013	0.043	0.759	0.998
Meconium Adj.[b]	PWY.5173	-0.219	0.191	0.258	0.998
Meconium Adj.[b]	PWY.5177	-0.051	0.043	0.249	0.998
Meconium Adj.[b]	PWY.5188	-0.014	0.036	0.705	0.998
Meconium Adj.[b]	PWY.5189	-0.041	0.148	0.782	0.998
Meconium Adj.[b]	PWY.5198	-0.034	0.115	0.766	0.998
Meconium Adj.[b]	PWY.5265	0.083	0.134	0.540	0.998
Meconium Adj.[b]	PWY.5304	-0.078	0.084	0.359	0.998
Meconium Adj.[b]	PWY.5345	-0.128	0.148	0.393	0.998
Meconium Adj.[b]	PWY.5367	0.097	0.156	0.537	0.998
Meconium Adj.[b]	PWY.5384	-0.088	0.088	0.321	0.998
Meconium Adj.[b]	PWY.5464	-0.118	0.158	0.457	0.998
Meconium Adj.[b]	PWY.5484	-0.046	0.043	0.290	0.998
Meconium Adj.[b]	PWY.561	-0.083	0.153	0.590	0.998
Meconium Adj.[b]	PWY.5667	-0.011	0.020	0.602	0.998
Meconium Adj.[b]	PWY.5675	-0.065	0.149	0.663	0.998
Meconium Adj.[b]	PWY.5690	0.025	0.048	0.603	0.998
Meconium Adj.[b]	PWY.5747	-0.042	0.149	0.778	0.998
Meconium Adj.[b]	PWY.5845	0.156	0.157	0.326	0.998
Meconium Adj.[b]	PWY.5850	0.045	0.078	0.564	0.998
Meconium Adj.[b]	PWY.5860	0.046	0.078	0.565	0.998

Exposure Window	MetaCyc Pathway ID	Effect Estimate[a]	Std. Error	p-value	q-value
Meconium Adj.[b]	PWY.5862	0.157	0.159	0.328	0.998
Meconium Adj.[b]	PWY.5863	0.038	0.145	0.796	0.998
Meconium Adj.[b]	PWY.5896	0.045	0.078	0.564	0.998
Meconium Adj.[b]	PWY.5913	-0.046	0.067	0.495	0.998
Meconium Adj.[b]	PWY.5918	0.062	0.143	0.668	0.998
Meconium Adj.[b]	PWY.5920	0.046	0.099	0.643	0.998
Meconium Adj.[b]	PWY.5941	-0.054	0.212	0.799	0.998
Meconium Adj.[b]	PWY.5971	0.027	0.104	0.797	0.998
Meconium Adj.[b]	PWY.5973	0.074	0.098	0.453	0.998
Meconium Adj.[b]	PWY.5989	0.100	0.116	0.392	0.998
Meconium Adj.[b]	PWY.5994	0.191	0.165	0.253	0.998
Meconium Adj.[b]	PWY.6121	-0.014	0.015	0.332	0.998
Meconium Adj.[b]	PWY.6122	-0.016	0.013	0.231	0.998
Meconium Adj.[b]	PWY.6123	0.015	0.016	0.354	0.998
Meconium Adj.[b]	PWY.6124	0.016	0.017	0.374	0.998
Meconium Adj.[b]	PWY.6126	-0.012	0.020	0.560	0.998
Meconium Adj.[b]	PWY.6151	-0.007	0.016	0.675	0.998
Meconium Adj.[b]	PWY.6163	-0.015	0.015	0.317	0.998
Meconium Adj.[b]	PWY.6168	-0.008	0.028	0.762	0.998
Meconium Adj.[b]	PWY.621	-0.033	0.042	0.435	0.998
Meconium Adj.[b]	PWY.622	-0.061	0.121	0.614	0.998
Meconium Adj.[b]	PWY.6270	-0.032	0.092	0.730	0.998
Meconium Adj.[b]	PWY.6277	-0.016	0.013	0.231	0.998
Meconium Adj.[b]	PWY.6282	0.060	0.120	0.617	0.998
Meconium Adj.[b]	PWY.6284	0.045	0.105	0.672	0.998
Meconium Adj.[b]	PWY.6285	-0.086	0.063	0.177	0.998
Meconium Adj.[b]	PWY.6305	0.013	0.042	0.755	0.998
Meconium Adj.[b]	PWY.6317	-0.030	0.028	0.282	0.998
Meconium Adj.[b]	PWY.6349	-0.042	0.128	0.746	0.998
Meconium Adj.[b]	PWY.6385	-0.007	0.010	0.514	0.998
Meconium Adj.[b]	PWY.6386	-0.006	0.010	0.546	0.998
Meconium Adj.[b]	PWY.6387	-0.006	0.009	0.517	0.998
Meconium Adj.[b]	PWY.6435	0.145	0.181	0.429	0.998
Meconium Adj.[b]	PWY.6470	-0.052	0.068	0.449	0.998
Meconium Adj.[b]	PWY.6471	0.019	0.054	0.723	0.998
Meconium Adj.[b]	PWY.6478	0.086	0.097	0.379	0.998
Meconium Adj.[b]	PWY.6527	-0.043	0.033	0.200	0.998

Exposure Window	MetaCyc Pathway ID	Effect Estimate[a]	Std. Error	p-value	q-value
Meconium Adj.[b]	PWY.6531	0.127	0.174	0.467	0.998
Meconium Adj.[b]	PWY.6549	0.024	0.036	0.506	0.998
Meconium Adj.[b]	PWY.6572	0.088	0.065	0.179	0.998
Meconium Adj.[b]	PWY.6588	0.077	0.151	0.611	0.998
Meconium Adj.[b]	PWY.6590	-0.057	0.088	0.518	0.998
Meconium Adj.[b]	PWY.6609	-0.009	0.019	0.616	0.998
Meconium Adj.[b]	PWY.6612	0.065	0.089	0.472	0.998
Meconium Adj.[b]	PWY.6628	-0.092	0.154	0.556	0.998
Meconium Adj.[b]	PWY.6690	-0.033	0.067	0.629	0.998
Meconium Adj.[b]	PWY.6703	-0.024	0.030	0.423	0.998
Meconium Adj.[b]	PWY.6731	-0.052	0.106	0.628	0.998
Meconium Adj.[b]	PWY.6749	0.109	0.109	0.323	0.998
Meconium Adj.[b]	PWY.6823	-0.033	0.058	0.572	0.998
Meconium Adj.[b]	PWY.6837	-0.028	0.054	0.604	0.998
Meconium Adj.[b]	PWY.6859	0.089	0.182	0.629	0.998
Meconium Adj.[b]	PWY.6876	-0.136	0.120	0.264	0.998
Meconium Adj.[b]	PWY.6895	0.114	0.122	0.355	0.998
Meconium Adj.[b]	PWY.6897	0.015	0.038	0.692	0.998
Meconium Adj.[b]	PWY.6936	-0.006	0.023	0.784	0.998
Meconium Adj.[b]	PWY.7003	0.081	0.136	0.552	0.998
Meconium Adj.[b]	PWY.7013	-0.029	0.059	0.630	0.998
Meconium Adj.[b]	PWY.7046	0.055	0.147	0.712	0.998
Meconium Adj.[b]	PWY.7115	-0.075	0.069	0.282	0.998
Meconium Adj.[b]	PWY.7117	-0.052	0.064	0.421	0.998
Meconium Adj.[b]	PWY.7184	0.013	0.049	0.789	0.998
Meconium Adj.[b]	PWY.7187	0.018	0.039	0.642	0.998
Meconium Adj.[b]	PWY.7196	-0.019	0.029	0.516	0.998
Meconium Adj.[b]	PWY.7197	0.019	0.056	0.742	0.998
Meconium Adj.[b]	PWY.7198	0.015	0.054	0.776	0.998
Meconium Adj.[b]	PWY.7204	0.045	0.077	0.557	0.998
Meconium Adj.[b]	PWY.7208	-0.024	0.038	0.535	0.998
Meconium Adj.[b]	PWY.7209	0.101	0.174	0.565	0.998
Meconium Adj.[b]	PWY.7210	-0.062	0.181	0.735	0.998
Meconium Adj.[b]	PWY.7219	-0.005	0.014	0.714	0.998
Meconium Adj.[b]	PWY.7228	0.012	0.047	0.805	0.998
Meconium Adj.[b]	PWY.7229	-0.012	0.013	0.366	0.998
Meconium Adj.[b]	PWY.7234	-0.024	0.078	0.763	0.998

Exposure Window	MetaCyc Pathway ID	Effect Estimate[a]	Std. Error	p-value	q-value
Meconium Adj.[b]	PWY.7237	-0.045	0.038	0.245	0.998
Meconium Adj.[b]	PWY.724	-0.007	0.009	0.471	0.998
Meconium Adj.[b]	PWY.7282	0.126	0.141	0.375	0.998
Meconium Adj.[b]	PWY.7286	-0.055	0.136	0.686	0.998
Meconium Adj.[b]	PWY.7315	-0.073	0.110	0.511	0.998
Meconium Adj.[b]	PWY.7316	0.150	0.132	0.260	0.998
Meconium Adj.[b]	PWY.7323	0.078	0.064	0.229	0.998
Meconium Adj.[b]	PWY.7328	-0.097	0.117	0.414	0.998
Meconium Adj.[b]	PWY.7332	0.142	0.159	0.377	0.998
Meconium Adj.[b]	PWY.7357	-0.021	0.022	0.344	0.998
Meconium Adj.[b]	PWY.7371	0.221	0.180	0.227	0.998
Meconium Adj.[b]	PWY.7392	0.084	0.168	0.619	0.998
Meconium Adj.[b]	PWY.7400	-0.016	0.014	0.270	0.998
Meconium Adj.[b]	PWY.7456	0.016	0.061	0.794	0.998
Meconium Adj.[b]	PWY.7560	-0.033	0.095	0.728	0.998
Meconium Adj.[b]	PWY.7616	-0.207	0.216	0.341	0.998
Meconium Adj.[b]	PWY.7663	0.098	0.104	0.350	0.998
Meconium Adj.[b]	PWY.7664	0.050	0.121	0.682	0.998
Meconium Adj.[b]	PWY0.1261	0.047	0.126	0.712	0.998
Meconium Adj.[b]	PWY0.1277	-0.018	0.052	0.727	0.998
Meconium Adj.[b]	PWY0.1296	-0.024	0.025	0.356	0.998
Meconium Adj.[b]	PWY0.1297	-0.018	0.060	0.770	0.998
Meconium Adj.[b]	PWY0.1298	-0.047	0.074	0.531	0.998
Meconium Adj.[b]	PWY0.1319	-0.011	0.020	0.601	0.998
Meconium Adj.[b]	PWY0.1415	-0.115	0.124	0.358	0.998
Meconium Adj.[b]	PWY0.166	0.009	0.039	0.812	0.998
Meconium Adj.[b]	PWY0.781	-0.049	0.062	0.435	0.998
Meconium Adj.[b]	PWY0.845	0.122	0.162	0.455	0.998
Meconium Adj.[b]	PWY0.862	0.045	0.124	0.718	0.998
Meconium Adj.[b]	PWY0.881	-0.084	0.127	0.512	0.998
Meconium Adj.[b]	PWY490.3	0.046	0.089	0.613	0.998
Meconium Adj.[b]	PWY4FS.7	0.028	0.074	0.711	0.998
Meconium Adj.[b]	PWY4FS.8	0.028	0.074	0.711	0.998
Meconium Adj.[b]	PWY4LZ.257	-0.084	0.180	0.643	0.998
Meconium Adj.[b]	PWY66.389	-0.226	0.206	0.279	0.998
Meconium Adj.[b]	PWY66.398	-0.072	0.127	0.574	0.998
Meconium Adj.[b]	PWY66.399	0.017	0.033	0.614	0.998

Exposure Window	MetaCyc Pathway ID	Effect Estimate[a]	Std. Error	p-value	q-value
Meconium Adj.[b]	PWY66.400	-0.056	0.042	0.190	0.998
Meconium Adj.[b]	PWY66.409	-0.012	0.044	0.780	0.998
Meconium Adj.[b]	PWY66.422	-0.021	0.019	0.278	0.998
Meconium Adj.[b]	PWYG.321	0.038	0.122	0.755	0.998
Meconium Adj.[b]	PYRIDNUCSAL.PWY	-0.099	0.118	0.407	0.998
Meconium Adj.[b]	PYRIDOXSYN.PWY	0.095	0.166	0.569	0.998
Meconium Adj.[b]	RHAMCAT.PWY	0.039	0.044	0.376	0.998
Meconium Adj.[b]	RIBOSYN2.PWY	-0.007	0.025	0.785	0.998
Meconium Adj.[b]	RUMP.PWY	0.088	0.180	0.627	0.998
Meconium Adj.[b]	SER.GLYSYN.PWY	-0.017	0.016	0.289	0.998
Meconium Adj.[b]	SO4ASSIM.PWY	-0.141	0.157	0.375	0.998
Meconium Adj.[b]	SULFATE.CYS.PWY	-0.129	0.150	0.393	0.998
Meconium Adj.[b]	TCA.GLYOX.BYPASS	-0.043	0.139	0.757	0.998
Meconium Adj.[b]	TCA	0.034	0.048	0.483	0.998
Meconium Adj.[b]	THISYNARA.PWY	-0.021	0.039	0.599	0.998
Meconium Adj.[b]	THREOCAT.PWY	-0.072	0.066	0.284	0.998
Meconium Adj.[b]	TRPSYN.PWY	-0.022	0.027	0.422	0.998
Meconium Adj.[b]	UDPNAGSYN.PWY	-0.040	0.031	0.205	0.998
Meconium Adj.[b]	CRNFORCAT.PWY	-0.017	0.102	0.865	0.998
Meconium Adj.[b]	DENOVOPURINE2.PWY	0.003	0.036	0.926	0.998
Meconium Adj.[b]	FASYN.INITIAL.PWY	-0.002	0.136	0.989	0.998
Meconium Adj.[b]	GALACTUROCAT.PWY	0.013	0.088	0.882	0.998
Meconium Adj.[b]	GLUCUROCAT.PWY	0.013	0.083	0.880	0.998
Meconium Adj.[b]	HEMESYN2.PWY	0.006	0.084	0.939	0.998
Meconium Adj.[b]	HEXITOLDEGSUPER.PWY	-0.003	0.029	0.930	0.998
Meconium Adj.[b]	P108.PWY	0.034	0.219	0.879	0.998
Meconium Adj.[b]	P185.PWY	-0.005	0.061	0.937	0.998
Meconium Adj.[b]	P23.PWY	-0.009	0.189	0.960	0.998
Meconium Adj.[b]	P562.PWY	0.003	0.072	0.971	0.998
Meconium Adj.[b]	PENTOSE.P.PWY	-0.004	0.094	0.969	0.998
Meconium Adj.[b]	POLYISOPRENSYN.PWY	0.003	0.133	0.980	0.998
Meconium Adj.[b]	PPGPPMET.PWY	-0.004	0.048	0.930	0.998
Meconium Adj.[b]	PWY.2941	-0.006	0.061	0.928	0.998
Meconium Adj.[b]	PWY.4702	-0.026	0.138	0.850	0.998
Meconium Adj.[b]	PWY.4981	0.012	0.064	0.848	0.998
Meconium Adj.[b]	PWY.5004	-0.001	0.081	0.993	0.998
Meconium Adj.[b]	PWY.5101	-0.054	0.260	0.838	0.998

Exposure Window	MetaCyc Pathway ID	Effect Estimate[a]	Std. Error	p-value	q-value
Meconium Adj.[b]	PWY.5121	0.012	0.071	0.870	0.998
Meconium Adj.[b]	PWY.5154	-0.002	0.074	0.976	0.998
Meconium Adj.[b]	PWY.5392	0.001	0.075	0.985	0.998
Meconium Adj.[b]	PWY.5656	-0.011	0.094	0.910	0.998
Meconium Adj.[b]	PWY.5659	-0.006	0.045	0.892	0.998
Meconium Adj.[b]	PWY.5676	0.009	0.048	0.859	0.998
Meconium Adj.[b]	PWY.5686	-0.001	0.010	0.945	0.998
Meconium Adj.[b]	PWY.5723	0.003	0.116	0.981	0.998
Meconium Adj.[b]	PWY.5791	-0.022	0.146	0.882	0.998
Meconium Adj.[b]	PWY.5837	-0.022	0.146	0.882	0.998
Meconium Adj.[b]	PWY.5838	0.014	0.152	0.928	0.998
Meconium Adj.[b]	PWY.5840	-0.018	0.135	0.892	0.998
Meconium Adj.[b]	PWY.5861	0.012	0.154	0.936	0.998
Meconium Adj.[b]	PWY.5897	-0.020	0.142	0.887	0.998
Meconium Adj.[b]	PWY.5898	-0.020	0.142	0.887	0.998
Meconium Adj.[b]	PWY.5899	-0.020	0.142	0.887	0.998
Meconium Adj.[b]	PWY.6113	0.022	0.097	0.820	0.998
Meconium Adj.[b]	PWY.6125	0.010	0.044	0.822	0.998
Meconium Adj.[b]	PWY.6147	-0.007	0.089	0.941	0.998
Meconium Adj.[b]	PWY.6167	-0.025	0.111	0.825	0.998
Meconium Adj.[b]	PWY.6353	0.008	0.032	0.817	0.998
Meconium Adj.[b]	PWY.6507	0.003	0.093	0.972	0.998
Meconium Adj.[b]	PWY.6545	0.008	0.047	0.872	0.998
Meconium Adj.[b]	PWY.6595	0.019	0.183	0.917	0.998
Meconium Adj.[b]	PWY.6606	0.007	0.037	0.848	0.998
Meconium Adj.[b]	PWY.6608	-0.007	0.039	0.851	0.998
Meconium Adj.[b]	PWY.6629	0.002	0.109	0.984	0.998
Meconium Adj.[b]	PWY.6630	-0.009	0.109	0.936	0.998
Meconium Adj.[b]	PWY.6700	0.004	0.016	0.828	0.998
Meconium Adj.[b]	PWY.6803	-0.007	0.106	0.951	0.998
Meconium Adj.[b]	PWY.6891	0.002	0.098	0.980	0.998
Meconium Adj.[b]	PWY.6892	0.000	0.035	0.994	0.998
Meconium Adj.[b]	PWY.6969	0.010	0.063	0.871	0.998
Meconium Adj.[b]	PWY.7094	-0.009	0.053	0.871	0.998
Meconium Adj.[b]	PWY.7211	0.004	0.043	0.919	0.998
Meconium Adj.[b]	PWY.7220	-0.007	0.041	0.874	0.998
Meconium Adj.[b]	PWY.7221	0.001	0.011	0.901	0.998

Exposure Window	MetaCyc Pathway ID	Effect Estimate[a]	Std. Error	p-value	q-value
Meconium Adj.[b]	PWY.7222	-0.007	0.041	0.874	0.998
Meconium Adj.[b]	PWY.7242	0.019	0.093	0.836	0.998
Meconium Adj.[b]	PWY.7254	0.009	0.142	0.950	0.998
Meconium Adj.[b]	PWY.7312	-0.012	0.095	0.901	0.998
Meconium Adj.[b]	PWY.7383	0.000	0.041	0.995	0.998
Meconium Adj.[b]	PWY.7388	-0.004	0.137	0.977	0.998
Meconium Adj.[b]	PWY.7539	-0.009	0.089	0.922	0.998
Meconium Adj.[b]	PWY.841	0.005	0.038	0.887	0.998
Meconium Adj.[b]	PWY0.1586	0.004	0.045	0.928	0.998
Meconium Adj.[b]	PWY0.162	-0.001	0.028	0.963	0.998
Meconium Adj.[b]	PWY0.42	-0.010	0.076	0.898	0.998
Meconium Adj.[b]	PWY3O.355	-0.029	0.150	0.847	0.998
Meconium Adj.[b]	PYRIDNUCSYN.PWY	-0.002	0.016	0.889	0.998
Meconium Adj.[b]	REDCITCYC	0.009	0.150	0.953	0.998
Meconium Adj.[b]	SALVADEHYPOX.PWY	0.006	0.038	0.882	0.998
Meconium Adj.[b]	TEICHOICACID.PWY	0.005	0.040	0.901	0.998
Meconium Adj.[b]	TRNA.CHARGING.PWY	-0.001	0.010	0.935	0.998
Meconium Adj.[b]	PWY.6901	0.000	0.081	0.998	0.998

[a]Models are adjusted for whether the child was ever breastfed, sex, mode of birth, and socioeconomic status
[b]Meconium Adj. includes cross-sectional exposure in the model

Table S5. Change in bacterial species relative abundance per doubling caffeine (ng/g)

Exposure Window	Species	Effect Estimate[a]	Std. Error	p-value	q-value
Meconium	Actinobaculum sp oral taxon 183	0.328	0.105	0.003	0.259
Meconium	Roseburia intestinalis	-0.511	0.171	0.005	0.259
Meconium	Firmicutes bacterium CAG 83	0.397	0.132	0.004	0.259
Meconium	Methanobrevibacter smithii	-0.013	0.059	0.829	0.975
Meconium	Actinomyces graevenitzii	-0.177	0.086	0.046	0.833
Meconium	Actinomyces johnsonii	0.144	0.129	0.271	0.975
Meconium	Actinomyces massiliensis	-0.038	0.067	0.576	0.975
Meconium	Actinomyces naeslundii	0.010	0.103	0.926	0.981
Meconium	Actinomyces odontolyticus	0.015	0.098	0.881	0.975
Meconium	Actinomyces oris	-0.046	0.113	0.683	0.975
Meconium	Actinomyces sp HMSC035G02	-0.008	0.093	0.928	0.981
Meconium	Actinomyces sp ICM47	-0.108	0.144	0.458	0.975
Meconium	Actinomyces sp S6 Spd3	0.017	0.045	0.705	0.975
Meconium	Actinomyces sp oral taxon 181	-0.074	0.050	0.147	0.975
Meconium	Actinomyces turicensis	-0.049	0.067	0.466	0.975
Meconium	Bifidobacterium adolescentis	0.095	0.176	0.593	0.975
Meconium	Bifidobacterium animalis	-0.033	0.137	0.808	0.975
Meconium	Bifidobacterium bifidum	0.266	0.135	0.056	0.833
Meconium	Bifidobacterium catenulatum	0.344	0.160	0.038	0.833
Meconium	Bifidobacterium longum	0.008	0.077	0.912	0.981
Meconium	Bifidobacterium pseudocatenulatum	-0.066	0.191	0.732	0.975
Meconium	Corynebacterium durum	-0.121	0.064	0.064	0.833
Meconium	Rothia mucilaginosa	-0.080	0.117	0.498	0.975
Meconium	Collinsella aerofaciens	0.019	0.100	0.851	0.975
Meconium	Collinsella intestinalis	0.043	0.062	0.494	0.975
Meconium	Collinsella stercoris	0.038	0.122	0.756	0.975
Meconium	Enorma massiliensis	-0.059	0.034	0.095	0.833
Meconium	.Collinsella. massiliensis	-0.029	0.048	0.549	0.975
Meconium	Adlercreutzia equolifaciens	0.089	0.068	0.195	0.975
Meconium	Asaccharobacter celatus	0.087	0.062	0.168	0.975
Meconium	Eggerthella lenta	-0.145	0.095	0.135	0.975
Meconium	Enterorhabdus caecimuris	0.064	0.110	0.560	0.975
Meconium	Gordonibacter pamelaeae	-0.040	0.076	0.603	0.975
Meconium	Slackia isoflavoniconvertens	0.052	0.113	0.647	0.975
Meconium	Bacteroides caccae	-0.057	0.155	0.714	0.975
Meconium	Bacteroides cellulosilyticus	-0.003	0.112	0.975	0.981
Meconium	Bacteroides coprocola	0.040	0.043	0.354	0.975
Meconium	Bacteroides dorei	-0.158	0.171	0.359	0.975
Meconium	Bacteroides faecis	-0.063	0.110	0.569	0.975
Meconium	Bacteroides finegoldii	-0.018	0.066	0.790	0.975
Meconium	Bacteroides fragilis	-0.110	0.205	0.595	0.975
Meconium	Bacteroides galacturonicus	0.063	0.066	0.349	0.975
Meconium	Bacteroides intestinalis	-0.003	0.062	0.965	0.981
Meconium	Bacteroides massiliensis	0.031	0.081	0.699	0.975
Meconium	Bacteroides ovatus	-0.109	0.149	0.467	0.975
Meconium	Bacteroides salyersiae	-0.047	0.067	0.486	0.975
Meconium	Bacteroides stercoris	0.166	0.127	0.197	0.975
Meconium	Bacteroides thetaiotaomicron	0.097	0.146	0.510	0.975
Meconium	Bacteroides uniformis	-0.051	0.125	0.682	0.975

Exposure Window	Species	Effect Estimate[a]	Std. Error	p-value	q-value
Meconium	Bacteroides vulgatus	-0.042	0.156	0.788	0.975
Meconium	Bacteroides xylanisolvens	-0.075	0.130	0.564	0.975
Meconium	Barnesiella intestinihominis	0.098	0.161	0.546	0.975
Meconium	Odoribacter splanchnicus	0.084	0.124	0.503	0.975
Meconium	Paraprevotella xylaniphila	0.021	0.058	0.724	0.975
Meconium	Prevotella copri	0.044	0.103	0.671	0.975
Meconium	Alistipes finegoldii	0.111	0.156	0.481	0.975
Meconium	Alistipes indistinctus	0.061	0.128	0.636	0.975
Meconium	Alistipes putredinis	0.121	0.156	0.445	0.975
Meconium	Alistipes shahii	0.024	0.154	0.875	0.975
Meconium	Parabacteroides distasonis	0.103	0.164	0.535	0.975
Meconium	Parabacteroides merdae	0.292	0.169	0.092	0.833
Meconium	Gemella sanguinis	-0.015	0.054	0.781	0.975
Meconium	Lactobacillus rhamnosus	0.040	0.074	0.588	0.975
Meconium	Lactobacillus rogosae	0.071	0.074	0.348	0.975
Meconium	Lactococcus lactis	-0.034	0.140	0.809	0.975
Meconium	Streptococcus australis	-0.080	0.114	0.490	0.975
Meconium	Streptococcus infantis	-0.056	0.050	0.271	0.975
Meconium	Streptococcus mitis	-0.212	0.122	0.091	0.833
Meconium	Streptococcus parasanguinis	-0.188	0.136	0.173	0.975
Meconium	Streptococcus salivarius	-0.069	0.092	0.456	0.975
Meconium	Streptococcus sanguinis	-0.032	0.076	0.671	0.975
Meconium	Streptococcus sp A12	-0.024	0.101	0.814	0.975
Meconium	Streptococcus sp F0442	-0.004	0.075	0.954	0.981
Meconium	Streptococcus thermophilus	-0.046	0.165	0.780	0.975
Meconium	Catabacter hongkongensis	-0.139	0.144	0.337	0.975
Meconium	Christensenella minuta	0.006	0.069	0.928	0.981
Meconium	Clostridium bolteae CAG 59	0.009	0.083	0.910	0.981
Meconium	Clostridium disporicum	-0.093	0.189	0.625	0.975
Meconium	Clostridium sp CAG 167	-0.130	0.103	0.215	0.975
Meconium	Clostridium sp CAG 58	0.079	0.095	0.411	0.975
Meconium	Clostridium ventriculi	0.058	0.101	0.571	0.975
Meconium	Hungatella hathewayi	-0.045	0.156	0.777	0.975
Meconium	Eubacterium sulci	-0.021	0.109	0.846	0.975
Meconium	Mogibacterium diversum	-0.082	0.070	0.249	0.975
Meconium	Intestinimonas butyriciproducens	-0.056	0.098	0.572	0.975
Meconium	Lawsonibacter asaccharolyticus	0.025	0.086	0.773	0.975
Meconium	Monoglobus pectinilyticus	-0.372	0.164	0.029	0.795
Meconium	Anaerofustis stercorihominis	-0.066	0.045	0.152	0.975
Meconium	Eubacterium callanderi	-0.037	0.054	0.501	0.975
Meconium	Eubacterium eligens	-0.038	0.166	0.819	0.975
Meconium	Eubacterium hallii	0.047	0.043	0.274	0.975
Meconium	Eubacterium limosum	-0.021	0.053	0.697	0.975
Meconium	Eubacterium ramulus	-0.019	0.104	0.855	0.975
Meconium	Eubacterium sp CAG 180	0.043	0.180	0.813	0.975
Meconium	Eubacterium sp CAG 38	-0.254	0.152	0.101	0.833
Meconium	Eubacterium ventriosum	0.119	0.135	0.384	0.975
Meconium	Anaerostipes hadrus	-0.101	0.044	0.028	0.795
Meconium	Blautia hydrogenotrophica	-0.026	0.089	0.770	0.975
Meconium	Blautia obeum	-0.003	0.077	0.974	0.981
Meconium	Blautia producta	-0.005	0.106	0.965	0.981
Meconium	Blautia sp CAG 257	-0.087	0.062	0.167	0.975

Exposure Window	Species	Effect Estimate[a]	Std. Error	p-value	q-value
Meconium	Blautia wexlerae	-0.050	0.083	0.552	0.975
Meconium	Ruminococcus gnavus	0.040	0.176	0.823	0.975
Meconium	Ruminococcus torques	-0.012	0.062	0.852	0.975
Meconium	Coprococcus catus	0.124	0.102	0.229	0.975
Meconium	Coprococcus comes	0.093	0.204	0.651	0.975
Meconium	Coprococcus eutactus	0.144	0.146	0.327	0.975
Meconium	Dorea formicigenerans	0.026	0.061	0.672	0.975
Meconium	Dorea longicatena	0.130	0.078	0.105	0.833
Meconium	Dorea sp CAG 317	-0.033	0.078	0.674	0.975
Meconium	Eisenbergiella massiliensis	-0.008	0.114	0.947	0.981
Meconium	Eisenbergiella tayi	-0.088	0.125	0.485	0.975
Meconium	Fusicatenibacter saccharivorans	-0.077	0.062	0.221	0.975
Meconium	Clostridium bolteae	-0.004	0.094	0.966	0.981
Meconium	Clostridium scindens	0.019	0.140	0.893	0.981
Meconium	Clostridium symbiosum	-0.060	0.047	0.209	0.975
Meconium	Eubacterium rectale	0.124	0.198	0.534	0.975
Meconium	Lachnospira pectinoschiza	-0.151	0.132	0.260	0.975
Meconium	Roseburia faecis	-0.253	0.141	0.078	0.833
Meconium	Roseburia hominis	-0.010	0.158	0.951	0.981
Meconium	Roseburia inulinivorans	-0.117	0.139	0.405	0.975
Meconium	Roseburia sp CAG 182	-0.022	0.130	0.865	0.975
Meconium	Roseburia sp CAG 309	-0.165	0.070	0.023	0.795
Meconium	Roseburia sp CAG 471	-0.076	0.130	0.564	0.975
Meconium	Sellimonas intestinalis	-0.228	0.177	0.206	0.975
Meconium	Tyzzerella nexilis	-0.104	0.107	0.336	0.975
Meconium	Oscillibacter sp 57 20	0.133	0.118	0.267	0.975
Meconium	Oscillibacter sp CAG 241	0.038	0.163	0.815	0.975
Meconium	Intestinibacter bartlettii	-0.095	0.057	0.100	0.833
Meconium	Romboutsia ilealis	0.030	0.122	0.804	0.975
Meconium	Agathobaculum butyriciproducens	-0.178	0.194	0.365	0.975
Meconium	Anaeromassilibacillus sp An250	-0.149	0.148	0.320	0.975
Meconium	Anaerotruncus colihominis	0.102	0.050	0.049	0.833
Meconium	Anaerotruncus sp CAG 528	-0.041	0.060	0.500	0.975
Meconium	Faecalibacterium prausnitzii	0.015	0.094	0.872	0.975
Meconium	Flavonifractor plautii	0.081	0.187	0.668	0.975
Meconium	Gemmiger formicilis	0.209	0.119	0.086	0.833
Meconium	Clostridium leptum	-0.024	0.111	0.830	0.975
Meconium	Eubacterium siraeum	-0.217	0.127	0.095	0.833
Meconium	Ruminococcus bicirculans	-0.042	0.161	0.794	0.975
Meconium	Ruminococcus bromii	-0.020	0.130	0.881	0.975
Meconium	Ruminococcus callidus	-0.171	0.114	0.141	0.975
Meconium	Ruminococcus lactaris	0.038	0.149	0.800	0.975
Meconium	Ruthenibacterium lactatiformans	0.067	0.093	0.474	0.975
Meconium	Coprobacillus cateniformis	0.105	0.131	0.429	0.975
Meconium	Clostridium innocuum	-0.195	0.160	0.231	0.975
Meconium	Clostridium spiroforme	0.158	0.168	0.351	0.975
Meconium	Erysipelatoclostridium ramosum	-0.036	0.124	0.775	0.975
Meconium	Holdemania filiformis	-0.057	0.043	0.185	0.975
Meconium	Turicibacter sanguinis	-0.196	0.145	0.183	0.975
Meconium	Firmicutes bacterium CAG 110	0.145	0.215	0.504	0.975
Meconium	Firmicutes bacterium CAG 145	0.009	0.176	0.961	0.981
Meconium	Firmicutes bacterium CAG 94	-0.040	0.079	0.620	0.975

Exposure Window	Species	Effect Estimate[a]	Std. Error	p-value	q-value
Meconium	Firmicutes bacterium CAG 95	0.199	0.118	0.098	0.833
Meconium	Phascolarctobacterium faecium	0.065	0.132	0.622	0.975
Meconium	Dialister invisus	0.050	0.152	0.742	0.975
Meconium	Veillonella atypica	-0.068	0.089	0.451	0.975
Meconium	Veillonella dispar	-0.074	0.049	0.139	0.975
Meconium	Veillonella parvula	-0.092	0.100	0.361	0.975
Meconium	Parvimonas micra	0.064	0.073	0.388	0.975
Meconium	Parasutterella excrementihominis	0.066	0.137	0.633	0.975
Meconium	Bilophila wadsworthia	0.021	0.070	0.767	0.975
Meconium	Desulfovibrio piger	0.001	0.060	0.981	0.981
Meconium	Escherichia coli	0.152	0.140	0.285	0.975
Meconium	Haemophilus parainfluenzae	-0.059	0.074	0.431	0.975
Meconium	Proteobacteria bacterium CAG 139	-0.030	0.051	0.556	0.975
Meconium	Akkermansia muciniphila	-0.282	0.244	0.254	0.975
Cross-sectional	Ruminococcus bicirculans	0.269	0.124	0.033	0.962
Cross-sectional	Escherichia coli	-0.240	0.105	0.025	0.962
Cross-sectional	Bacteroides dorei	0.261	0.123	0.037	0.962
Cross-sectional	Eubacterium sp CAG 251	0.118	0.055	0.037	0.962
Cross-sectional	Ruminococcus torques	-0.109	0.057	0.061	0.962
Cross-sectional	Dialister invisus	-0.222	0.126	0.081	0.962
Cross-sectional	Blautia wexlerae	-0.105	0.060	0.085	0.962
Cross-sectional	Anaeromassilibacillus sp An250	-0.188	0.110	0.091	0.962
Cross-sectional	Eubacterium siraeum	-0.201	0.117	0.090	0.962
Cross-sectional	Methanobrevibacter smithii	-0.127	0.084	0.134	0.962
Cross-sectional	Actinomyces massiliensis	0.093	0.062	0.134	0.962
Cross-sectional	Collinsella intestinalis	0.110	0.072	0.132	0.962
Cross-sectional	Bacteroides galacturonicus	-0.090	0.057	0.114	0.962
Cross-sectional	Bacteroides stercoris	0.171	0.109	0.122	0.962
Cross-sectional	Streptococcus sp A12	0.109	0.072	0.138	0.962
Cross-sectional	Phascolarctobacterium faecium	0.169	0.103	0.105	0.962
Cross-sectional	Actinomyces odontolyticus	-0.055	0.087	0.529	0.962
Cross-sectional	Actinomyces sp S6 Spd3	0.037	0.037	0.316	0.962
Cross-sectional	Actinomyces turicensis	-0.074	0.053	0.170	0.962
Cross-sectional	Bifidobacterium adolescentis	0.160	0.147	0.280	0.962
Cross-sectional	Bifidobacterium animalis	-0.053	0.101	0.600	0.962
Cross-sectional	Bifidobacterium longum	0.051	0.067	0.447	0.962
Cross-sectional	Corynebacterium durum	0.045	0.070	0.522	0.962
Cross-sectional	Collinsella aerofaciens	-0.209	0.161	0.199	0.962
Cross-sectional	Collinsella stercoris	-0.109	0.095	0.254	0.962
Cross-sectional	.Collinsella. massiliensis	0.049	0.057	0.389	0.962
Cross-sectional	Adlercreutzia equolifaciens	-0.027	0.053	0.619	0.962
Cross-sectional	Eggerthella lenta	0.066	0.082	0.423	0.962
Cross-sectional	Enterorhabdus caecimuris	-0.097	0.078	0.214	0.962
Cross-sectional	Gordonibacter pamelaeae	-0.044	0.059	0.455	0.962
Cross-sectional	Bacteroides cellulosilyticus	-0.092	0.102	0.373	0.962
Cross-sectional	Bacteroides coprocola	0.032	0.058	0.575	0.962
Cross-sectional	Bacteroides faecis	-0.086	0.096	0.371	0.962
Cross-sectional	Bacteroides fragilis	-0.128	0.138	0.356	0.962
Cross-sectional	Bacteroides massiliensis	-0.124	0.092	0.182	0.962
Cross-sectional	Bacteroides ovatus	0.068	0.117	0.565	0.962
Cross-sectional	Bacteroides thetaiotaomicron	0.118	0.129	0.361	0.962
Cross-sectional	Bacteroides uniformis	0.127	0.104	0.226	0.962

Exposure Window	Species	Effect Estimate[a]	Std. Error	p-value	q-value
Cross-sectional	Bacteroides vulgatus	-0.071	0.121	0.562	0.962
Cross-sectional	Bacteroides xylanisolvens	0.128	0.141	0.366	0.962
Cross-sectional	Prevotella copri	0.049	0.089	0.585	0.962
Cross-sectional	Alistipes shahii	-0.069	0.121	0.569	0.962
Cross-sectional	Parabacteroides distasonis	-0.153	0.115	0.188	0.962
Cross-sectional	Gemella sanguinis	-0.028	0.039	0.480	0.962
Cross-sectional	Lactococcus lactis	0.129	0.103	0.212	0.962
Cross-sectional	Streptococcus australis	-0.054	0.078	0.491	0.962
Cross-sectional	Streptococcus mitis	-0.073	0.083	0.380	0.962
Cross-sectional	Streptococcus salivarius	-0.053	0.072	0.463	0.962
Cross-sectional	Streptococcus sanguinis	-0.033	0.054	0.550	0.962
Cross-sectional	Streptococcus sp F0442	0.040	0.058	0.490	0.962
Cross-sectional	Streptococcus thermophilus	-0.108	0.117	0.359	0.962
Cross-sectional	Catabacter hongkongensis	-0.138	0.101	0.177	0.962
Cross-sectional	Clostridium sp CAG 167	-0.049	0.083	0.560	0.962
Cross-sectional	Clostridium sp CAG 58	-0.109	0.096	0.258	0.962
Cross-sectional	Clostridium ventriculi	-0.090	0.082	0.281	0.962
Cross-sectional	Eubacterium sulci	-0.036	0.069	0.605	0.962
Cross-sectional	Mogibacterium diversum	0.079	0.078	0.314	0.962
Cross-sectional	Intestinimonas butyriciproducens	0.072	0.073	0.323	0.962
Cross-sectional	Lawsonibacter asaccharolyticus	0.041	0.072	0.571	0.962
Cross-sectional	Eubacterium sp CAG 180	0.144	0.143	0.316	0.962
Cross-sectional	Eubacterium sp CAG 274	0.043	0.056	0.445	0.962
Cross-sectional	Eubacterium sp CAG 38	0.150	0.135	0.269	0.962
Cross-sectional	Blautia hydrogenotrophica	-0.087	0.086	0.314	0.962
Cross-sectional	Blautia producta	-0.064	0.077	0.408	0.962
Cross-sectional	Ruminococcus gnavus	-0.125	0.155	0.423	0.962
Cross-sectional	Coprococcus comes	0.098	0.149	0.515	0.962
Cross-sectional	Coprococcus eutactus	0.206	0.170	0.229	0.962
Cross-sectional	Dorea longicatena	-0.097	0.068	0.158	0.962
Cross-sectional	Dorea sp CAG 317	-0.054	0.053	0.315	0.962
Cross-sectional	Eisenbergiella tayi	-0.057	0.096	0.558	0.962
Cross-sectional	Clostridium bolteae	-0.060	0.107	0.576	0.962
Cross-sectional	Clostridium symbiosum	-0.031	0.043	0.465	0.962
Cross-sectional	Eubacterium rectale	0.147	0.147	0.319	0.962
Cross-sectional	Lachnospira pectinoschiza	-0.094	0.147	0.524	0.962
Cross-sectional	Roseburia faecis	-0.107	0.116	0.359	0.962
Cross-sectional	Roseburia hominis	0.082	0.120	0.493	0.962
Cross-sectional	Roseburia intestinalis	-0.109	0.137	0.429	0.962
Cross-sectional	Roseburia inulinivorans	0.079	0.097	0.423	0.962
Cross-sectional	Roseburia sp CAG 309	0.040	0.063	0.523	0.962
Cross-sectional	Roseburia sp CAG 471	-0.087	0.100	0.388	0.962
Cross-sectional	Tyzzerella nexilis	0.081	0.067	0.231	0.962
Cross-sectional	Oscillibacter sp CAG 241	-0.138	0.121	0.258	0.962
Cross-sectional	Intestinibacter bartlettii	-0.026	0.049	0.594	0.962
Cross-sectional	Romboutsia ilealis	-0.102	0.093	0.277	0.962
Cross-sectional	Agathobaculum butyriciproducens	-0.068	0.135	0.617	0.962
Cross-sectional	Anaerotruncus colihominis	0.062	0.071	0.379	0.962
Cross-sectional	Gemmiger formicilis	0.047	0.089	0.601	0.962
Cross-sectional	Ruminococcus bromii	0.130	0.131	0.322	0.962
Cross-sectional	Ruminococcus lactaris	-0.100	0.113	0.377	0.962
Cross-sectional	Coprobacillus cateniformis	-0.176	0.122	0.153	0.962

Exposure Window	Species	Effect Estimate[a]	Std. Error	p-value	q-value
Cross-sectional	Clostridium spiroforme	-0.173	0.121	0.158	0.962
Cross-sectional	Erysipelatoclostridium ramosum	-0.052	0.089	0.562	0.962
Cross-sectional	Holdemania filiformis	0.043	0.053	0.421	0.962
Cross-sectional	Firmicutes bacterium CAG 83	0.119	0.101	0.245	0.962
Cross-sectional	Firmicutes bacterium CAG 94	-0.049	0.061	0.421	0.962
Cross-sectional	Firmicutes bacterium CAG 95	0.061	0.102	0.547	0.962
Cross-sectional	Veillonella dispar	0.026	0.033	0.424	0.962
Cross-sectional	Veillonella parvula	0.040	0.069	0.565	0.962
Cross-sectional	Parasutterella excrementihominis	0.071	0.098	0.474	0.962
Cross-sectional	Desulfovibrio piger	0.034	0.064	0.600	0.962
Cross-sectional	Haemophilus parainfluenzae	0.055	0.043	0.201	0.962
Cross-sectional	Akkermansia muciniphila	-0.271	0.214	0.209	0.962
Cross-sectional	Ruthenibacterium lactatiformans	-0.033	0.067	0.624	0.962
Cross-sectional	Asaccharobacter celatus	-0.024	0.050	0.634	0.962
Cross-sectional	Lactobacillus rogosae	-0.028	0.060	0.639	0.962
Cross-sectional	Odoribacter splanchnicus	0.053	0.112	0.641	0.962
Cross-sectional	Bifidobacterium catenulatum	-0.052	0.126	0.681	0.967
Cross-sectional	Bifidobacterium pseudocatenulatum	-0.063	0.140	0.654	0.964
Cross-sectional	Monoglobus pectinilyticus	0.056	0.136	0.680	0.967
Cross-sectional	Blautia obeum	-0.024	0.055	0.662	0.964
Cross-sectional	Firmicutes bacterium CAG 145	0.056	0.130	0.667	0.964
Cross-sectional	Bilophila wadsworthia	-0.031	0.069	0.657	0.964
Cross-sectional	Bacteroides caccae	-0.039	0.107	0.713	0.968
Cross-sectional	Bacteroides eggerthii	-0.024	0.064	0.712	0.968
Cross-sectional	Hungatella hathewayi	0.042	0.108	0.702	0.968
Cross-sectional	Eubacterium ramulus	-0.026	0.075	0.724	0.968
Cross-sectional	Eubacterium ventriosum	-0.037	0.100	0.711	0.968
Cross-sectional	Coprococcus catus	0.039	0.109	0.721	0.968
Cross-sectional	Flavonifractor plautii	0.053	0.144	0.712	0.968
Cross-sectional	Clostridium leptum	-0.037	0.115	0.745	0.969
Cross-sectional	Actinomyces sp ICM47	-0.032	0.103	0.757	0.969
Cross-sectional	Dorea formicigenerans	-0.017	0.056	0.759	0.969
Cross-sectional	Faecalibacterium prausnitzii	0.021	0.069	0.757	0.969
Cross-sectional	Eisenbergiella massiliensis	0.025	0.084	0.766	0.969
Cross-sectional	Rothia mucilaginosa	-0.025	0.085	0.768	0.969
Cross-sectional	Paraprevotella xylaniphila	0.021	0.072	0.771	0.969
Cross-sectional	Veillonella atypica	0.018	0.063	0.774	0.969
Cross-sectional	Actinomyces graevenitzii	-0.014	0.058	0.807	0.976
Cross-sectional	Actinomyces johnsonii	-0.025	0.102	0.806	0.976
Cross-sectional	Alistipes finegoldii	-0.028	0.114	0.804	0.976
Cross-sectional	Eubacterium hallii	0.008	0.034	0.806	0.976
Cross-sectional	Actinomyces oris	0.024	0.101	0.810	0.976
Cross-sectional	Fusicatenibacter saccharivorans	-0.012	0.050	0.817	0.976
Cross-sectional	Actinomyces sp HMSC035G02	0.015	0.075	0.845	0.997
Cross-sectional	Actinobaculum sp oral taxon 183	-0.001	0.096	0.989	0.997
Cross-sectional	Actinomyces naeslundii	0.012	0.118	0.920	0.997
Cross-sectional	Actinomyces sp oral taxon 181	0.004	0.034	0.901	0.997
Cross-sectional	Bifidobacterium bifidum	-0.016	0.127	0.899	0.997
Cross-sectional	Bacteroides finegoldii	-0.005	0.075	0.947	0.997
Cross-sectional	Barnesiella intestinihominis	0.003	0.128	0.981	0.997
Cross-sectional	Alistipes indistinctus	0.001	0.090	0.990	0.997
Cross-sectional	Alistipes putredinis	0.003	0.107	0.978	0.997

Exposure Window	Species	Effect Estimate[a]	Std. Error	p-value	q-value
Cross-sectional	Parabacteroides merdae	-0.010	0.128	0.939	0.997
Cross-sectional	Streptococcus parasanguinis	-0.016	0.099	0.868	0.997
Cross-sectional	Christensenella minuta	0.010	0.062	0.870	0.997
Cross-sectional	Clostridium disporicum	-0.021	0.146	0.889	0.997
Cross-sectional	Eubacterium eligens	0.028	0.158	0.859	0.997
Cross-sectional	Eubacterium limosum	-0.002	0.048	0.964	0.997
Cross-sectional	Anaerostipes hadrus	-0.003	0.037	0.939	0.997
Cross-sectional	Blautia sp CAG 257	-0.004	0.063	0.946	0.997
Cross-sectional	Clostridium scindens	-0.005	0.089	0.957	0.997
Cross-sectional	Roseburia sp CAG 182	0.007	0.115	0.951	0.997
Cross-sectional	Sellimonas intestinalis	-0.009	0.127	0.941	0.997
Cross-sectional	Ruminococcus callidus	0.010	0.104	0.921	0.997
Cross-sectional	Clostridium innocuum	-0.006	0.120	0.957	0.997
Cross-sectional	Turicibacter sanguinis	0.007	0.111	0.949	0.997
Cross-sectional	Firmicutes bacterium CAG 110	-0.015	0.160	0.926	0.997
Cross-sectional	Parvimonas micra	-0.002	0.054	0.975	0.997
Cross-sectional	Oscillibacter sp 57 20	0.000	0.098	0.997	0.997
Meconium Adj.[b]	Methanobrevibacter smithii	-0.018	0.059	0.768	0.963
Meconium Adj.[b]	Actinobaculum sp oral taxon 183	0.339	0.104	0.002	0.316
Meconium Adj.[b]	Actinomyces graevenitzii	-0.184	0.086	0.038	0.853
Meconium Adj.[b]	Actinomyces johnsonii	0.139	0.130	0.293	0.963
Meconium Adj.[b]	Actinomyces massiliensis	-0.041	0.068	0.547	0.963
Meconium Adj.[b]	Actinomyces naeslundii	0.014	0.104	0.890	0.975
Meconium Adj.[b]	Actinomyces odontolyticus	0.017	0.099	0.868	0.975
Meconium Adj.[b]	Actinomyces oris	-0.045	0.114	0.695	0.963
Meconium Adj.[b]	Actinomyces sp HMSC035G02	-0.007	0.095	0.941	0.975
Meconium Adj.[b]	Actinomyces sp ICM47	-0.101	0.145	0.490	0.963
Meconium Adj.[b]	Actinomyces sp S6 Spd3	0.016	0.045	0.728	0.963
Meconium Adj.[b]	Actinomyces sp oral taxon 181	-0.077	0.051	0.136	0.944
Meconium Adj.[b]	Actinomyces turicensis	-0.053	0.067	0.439	0.963
Meconium Adj.[b]	Bifidobacterium adolescentis	0.093	0.179	0.605	0.963
Meconium Adj.[b]	Bifidobacterium animalis	-0.034	0.138	0.808	0.963
Meconium Adj.[b]	Bifidobacterium bifidum	0.259	0.136	0.064	0.877
Meconium Adj.[b]	Bifidobacterium catenulatum	0.342	0.162	0.042	0.853
Meconium Adj.[b]	Bifidobacterium longum	0.008	0.078	0.915	0.975
Meconium Adj.[b]	Bifidobacterium pseudocatenulatum	-0.079	0.192	0.683	0.963
Meconium Adj.[b]	Corynebacterium durum	-0.128	0.063	0.049	0.853
Meconium Adj.[b]	Rothia mucilaginosa	-0.086	0.118	0.472	0.963
Meconium Adj.[b]	Collinsella aerofaciens	0.024	0.101	0.814	0.963
Meconium Adj.[b]	Collinsella intestinalis	0.042	0.063	0.507	0.963
Meconium Adj.[b]	Collinsella stercoris	0.037	0.124	0.766	0.963
Meconium Adj.[b]	Enorma massiliensis	-0.061	0.035	0.087	0.877
Meconium Adj.[b]	.Collinsella. massiliensis	-0.029	0.048	0.549	0.963
Meconium Adj.[b]	Adlercreutzia equolifaciens	0.088	0.069	0.206	0.963
Meconium Adj.[b]	Asaccharobacter celatus	0.087	0.063	0.175	0.963
Meconium Adj.[b]	Eggerthella lenta	-0.143	0.097	0.147	0.944
Meconium Adj.[b]	Enterorhabdus caecimuris	0.061	0.111	0.586	0.963
Meconium Adj.[b]	Gordonibacter pamelaeae	-0.040	0.077	0.609	0.963
Meconium Adj.[b]	Slackia isoflavoniconvertens	0.059	0.114	0.607	0.963
Meconium Adj.[b]	Bacteroides caccae	-0.073	0.153	0.634	0.963
Meconium Adj.[b]	Bacteroides cellulosilyticus	-0.021	0.107	0.849	0.975
Meconium Adj.[b]	Bacteroides coprocola	0.045	0.042	0.281	0.963

Exposure Window	Species	Effect Estimate[a]	Std. Error	p-value	q-value
Meconium Adj.[b]	Bacteroides dorei	-0.152	0.172	0.383	0.963
Meconium Adj.[b]	Bacteroides faecis	-0.076	0.107	0.480	0.963
Meconium Adj.[b]	Bacteroides finegoldii	-0.014	0.067	0.840	0.975
Meconium Adj.[b]	Bacteroides fragilis	-0.085	0.201	0.673	0.963
Meconium Adj.[b]	Bacteroides galacturonicus	0.068	0.066	0.308	0.963
Meconium Adj.[b]	Bacteroides intestinalis	-0.007	0.062	0.915	0.975
Meconium Adj.[b]	Bacteroides massiliensis	0.022	0.079	0.779	0.963
Meconium Adj.[b]	Bacteroides ovatus	-0.083	0.139	0.554	0.963
Meconium Adj.[b]	Bacteroides salyersiae	-0.051	0.067	0.451	0.963
Meconium Adj.[b]	Bacteroides stercoris	0.159	0.127	0.220	0.963
Meconium Adj.[b]	Bacteroides thetaiotaomicron	0.111	0.144	0.447	0.963
Meconium Adj.[b]	Bacteroides uniformis	-0.063	0.124	0.615	0.963
Meconium Adj.[b]	Bacteroides vulgatus	-0.053	0.156	0.736	0.963
Meconium Adj.[b]	Bacteroides xylanisolvens	-0.094	0.125	0.455	0.963
Meconium Adj.[b]	Barnesiella intestinihominis	0.091	0.163	0.577	0.963
Meconium Adj.[b]	Odoribacter splanchnicus	0.074	0.124	0.555	0.963
Meconium Adj.[b]	Paraprevotella xylaniphila	0.019	0.059	0.754	0.963
Meconium Adj.[b]	Prevotella copri	0.048	0.104	0.644	0.963
Meconium Adj.[b]	Alistipes finegoldii	0.101	0.156	0.521	0.963
Meconium Adj.[b]	Alistipes indistinctus	0.051	0.128	0.692	0.963
Meconium Adj.[b]	Alistipes putredinis	0.118	0.159	0.461	0.963
Meconium Adj.[b]	Alistipes shahii	0.018	0.156	0.909	0.975
Meconium Adj.[b]	Parabacteroides distasonis	0.118	0.163	0.472	0.963
Meconium Adj.[b]	Parabacteroides merdae	0.284	0.171	0.104	0.877
Meconium Adj.[b]	Gemella sanguinis	-0.011	0.054	0.838	0.975
Meconium Adj.[b]	Lactobacillus rhamnosus	0.038	0.074	0.616	0.963
Meconium Adj.[b]	Lactobacillus rogosae	0.084	0.070	0.237	0.963
Meconium Adj.[b]	Lactococcus lactis	-0.033	0.142	0.819	0.963
Meconium Adj.[b]	Streptococcus australis	-0.086	0.115	0.456	0.963
Meconium Adj.[b]	Streptococcus infantis	-0.056	0.051	0.273	0.963
Meconium Adj.[b]	Streptococcus mitis	-0.203	0.122	0.106	0.877
Meconium Adj.[b]	Streptococcus parasanguinis	-0.170	0.131	0.203	0.963
Meconium Adj.[b]	Streptococcus salivarius	-0.060	0.091	0.511	0.963
Meconium Adj.[b]	Streptococcus sanguinis	-0.033	0.077	0.670	0.963
Meconium Adj.[b]	Streptococcus sp A12	-0.032	0.100	0.752	0.963
Meconium Adj.[b]	Streptococcus sp F0442	-0.008	0.075	0.917	0.975
Meconium Adj.[b]	Streptococcus thermophilus	-0.062	0.164	0.709	0.963
Meconium Adj.[b]	Catabacter hongkongensis	-0.137	0.145	0.351	0.963
Meconium Adj.[b]	Christensenella minuta	0.000	0.069	0.998	0.998
Meconium Adj.[b]	Clostridium bolteae CAG 59	0.003	0.083	0.971	0.983
Meconium Adj.[b]	Clostridium disporicum	-0.116	0.185	0.534	0.963
Meconium Adj.[b]	Clostridium sp CAG 167	-0.123	0.104	0.242	0.963
Meconium Adj.[b]	Clostridium sp CAG 58	0.081	0.096	0.407	0.963
Meconium Adj.[b]	Clostridium ventriculi	0.051	0.101	0.616	0.963
Meconium Adj.[b]	Hungatella hathewayi	-0.058	0.156	0.711	0.963
Meconium Adj.[b]	Eubacterium sulci	-0.026	0.111	0.814	0.963
Meconium Adj.[b]	Mogibacterium diversum	-0.083	0.071	0.247	0.963
Meconium Adj.[b]	Intestinimonas butyriciproducens	-0.056	0.099	0.573	0.963
Meconium Adj.[b]	Lawsonibacter asaccharolyticus	0.023	0.087	0.790	0.963
Meconium Adj.[b]	Monoglobus pectinilyticus	-0.369	0.166	0.032	0.853
Meconium Adj.[b]	Anaerofustis stercorihominis	-0.071	0.045	0.119	0.882
Meconium Adj.[b]	Eubacterium callanderi	-0.035	0.055	0.523	0.963

Exposure Window	Species	Effect Estimate[a]	Std. Error	p-value	q-value
Meconium Adj.[b]	Eubacterium eligens	-0.051	0.166	0.763	0.963
Meconium Adj.[b]	Eubacterium hallii	0.050	0.043	0.249	0.963
Meconium Adj.[b]	Eubacterium limosum	-0.012	0.050	0.815	0.963
Meconium Adj.[b]	Eubacterium ramulus	-0.012	0.104	0.910	0.975
Meconium Adj.[b]	Eubacterium sp CAG 180	0.046	0.182	0.802	0.963
Meconium Adj.[b]	Eubacterium sp CAG 38	-0.260	0.153	0.098	0.877
Meconium Adj.[b]	Eubacterium ventriosum	0.118	0.137	0.395	0.963
Meconium Adj.[b]	Anaerostipes hadrus	-0.097	0.044	0.034	0.853
Meconium Adj.[b]	Blautia hydrogenotrophica	-0.025	0.090	0.779	0.963
Meconium Adj.[b]	Blautia obeum	0.003	0.077	0.967	0.983
Meconium Adj.[b]	Blautia producta	-0.010	0.107	0.926	0.975
Meconium Adj.[b]	Blautia sp CAG 257	-0.081	0.061	0.194	0.963
Meconium Adj.[b]	Blautia wexlerae	-0.041	0.082	0.622	0.963
Meconium Adj.[b]	Ruminococcus gnavus	0.043	0.178	0.812	0.963
Meconium Adj.[b]	Ruminococcus torques	-0.004	0.061	0.946	0.975
Meconium Adj.[b]	Coprococcus catus	0.126	0.103	0.228	0.963
Meconium Adj.[b]	Coprococcus comes	0.107	0.204	0.602	0.963
Meconium Adj.[b]	Coprococcus eutactus	0.125	0.141	0.381	0.963
Meconium Adj.[b]	Dorea formicigenerans	0.034	0.059	0.572	0.963
Meconium Adj.[b]	Dorea longicatena	0.130	0.079	0.110	0.877
Meconium Adj.[b]	Dorea sp CAG 317	-0.045	0.075	0.552	0.963
Meconium Adj.[b]	Eisenbergiella massiliensis	-0.010	0.116	0.931	0.975
Meconium Adj.[b]	Eisenbergiella tayi	-0.093	0.126	0.466	0.963
Meconium Adj.[b]	Fusicatenibacter saccharivorans	-0.078	0.063	0.220	0.963
Meconium Adj.[b]	Clostridium bolteae	-0.012	0.094	0.900	0.975
Meconium Adj.[b]	Clostridium scindens	0.019	0.142	0.897	0.975
Meconium Adj.[b]	Clostridium symbiosum	-0.060	0.048	0.219	0.963
Meconium Adj.[b]	Eubacterium rectale	0.145	0.196	0.462	0.963
Meconium Adj.[b]	Lachnospira pectinoschiza	-0.145	0.133	0.283	0.963
Meconium Adj.[b]	Roseburia faecis	-0.234	0.136	0.092	0.877
Meconium Adj.[b]	Roseburia hominis	-0.013	0.160	0.936	0.975
Meconium Adj.[b]	Roseburia intestinalis	-0.504	0.173	0.006	0.316
Meconium Adj.[b]	Roseburia inulinivorans	-0.121	0.141	0.393	0.963
Meconium Adj.[b]	Roseburia sp CAG 182	-0.032	0.131	0.809	0.963
Meconium Adj.[b]	Roseburia sp CAG 309	-0.170	0.070	0.020	0.839
Meconium Adj.[b]	Roseburia sp CAG 471	-0.070	0.132	0.598	0.963
Meconium Adj.[b]	Sellimonas intestinalis	-0.230	0.180	0.208	0.963
Meconium Adj.[b]	Tyzzerella nexilis	-0.098	0.107	0.368	0.963
Meconium Adj.[b]	Oscillibacter sp 57 20	0.136	0.120	0.264	0.963
Meconium Adj.[b]	Oscillibacter sp CAG 241	0.039	0.165	0.814	0.963
Meconium Adj.[b]	Intestinibacter bartlettii	-0.100	0.056	0.081	0.877
Meconium Adj.[b]	Romboutsia ilealis	0.021	0.122	0.864	0.975
Meconium Adj.[b]	Agathobaculum butyriciproducens	-0.162	0.194	0.408	0.963
Meconium Adj.[b]	Anaeromassilibacillus sp An250	-0.172	0.141	0.231	0.963
Meconium Adj.[b]	Anaerotruncus colihominis	0.101	0.051	0.055	0.853
Meconium Adj.[b]	Anaerotruncus sp CAG 528	-0.042	0.060	0.485	0.963
Meconium Adj.[b]	Faecalibacterium prausnitzii	0.007	0.093	0.938	0.975
Meconium Adj.[b]	Flavonifractor plautii	0.084	0.189	0.659	0.963
Meconium Adj.[b]	Gemmiger formicilis	0.206	0.120	0.095	0.877
Meconium Adj.[b]	Clostridium leptum	-0.019	0.112	0.865	0.975
Meconium Adj.[b]	Eubacterium siraeum	-0.220	0.128	0.094	0.877
Meconium Adj.[b]	Ruminococcus bicirculans	-0.055	0.161	0.735	0.963

Exposure Window	Species	Effect Estimate[a]	Std. Error	p-value	q-value
Meconium Adj.[b]	Ruminococcus bromii	-0.037	0.126	0.770	0.963
Meconium Adj.[b]	Ruminococcus callidus	-0.180	0.114	0.122	0.882
Meconium Adj.[b]	Ruminococcus lactaris	0.042	0.151	0.780	0.963
Meconium Adj.[b]	Ruthenibacterium lactatiformans	0.063	0.094	0.508	0.963
Meconium Adj.[b]	Coprobacillus cateniformis	0.089	0.128	0.492	0.963
Meconium Adj.[b]	Clostridium innocuum	-0.191	0.162	0.246	0.963
Meconium Adj.[b]	Clostridium spiroforme	0.140	0.165	0.402	0.963
Meconium Adj.[b]	Erysipelatoclostridium ramosum	-0.046	0.123	0.709	0.963
Meconium Adj.[b]	Holdemania filiformis	-0.062	0.042	0.147	0.944
Meconium Adj.[b]	Turicibacter sanguinis	-0.208	0.145	0.159	0.949
Meconium Adj.[b]	Firmicutes bacterium CAG 110	0.149	0.218	0.498	0.963
Meconium Adj.[b]	Firmicutes bacterium CAG 145	0.008	0.178	0.963	0.983
Meconium Adj.[b]	Firmicutes bacterium CAG 83	0.384	0.131	0.005	0.316
Meconium Adj.[b]	Firmicutes bacterium CAG 94	-0.048	0.078	0.545	0.963
Meconium Adj.[b]	Firmicutes bacterium CAG 95	0.218	0.111	0.056	0.853
Meconium Adj.[b]	Phascolarctobacterium faecium	0.066	0.134	0.624	0.963
Meconium Adj.[b]	Dialister invisus	0.051	0.154	0.742	0.963
Meconium Adj.[b]	Veillonella atypica	-0.073	0.089	0.419	0.963
Meconium Adj.[b]	Veillonella dispar	-0.071	0.049	0.156	0.949
Meconium Adj.[b]	Veillonella parvula	-0.095	0.101	0.356	0.963
Meconium Adj.[b]	Parvimonas micra	0.061	0.074	0.418	0.963
Meconium Adj.[b]	Parasutterella excrementihominis	0.067	0.139	0.630	0.963
Meconium Adj.[b]	Bilophila wadsworthia	0.022	0.071	0.763	0.963
Meconium Adj.[b]	Desulfovibrio piger	0.000	0.061	0.994	0.998
Meconium Adj.[b]	Escherichia coli	0.152	0.142	0.292	0.963
Meconium Adj.[b]	Haemophilus parainfluenzae	-0.061	0.075	0.424	0.963
Meconium Adj.[b]	Proteobacteria bacterium CAG 139	-0.036	0.050	0.479	0.963
Meconium Adj.[b]	Akkermansia muciniphila	-0.283	0.248	0.260	0.963

[a]Models are adjusted for whether the child was ever breastfed, sex, mode of birth, and socioeconomic status
[b]Meconium Adj. includes cross-sectional exposure in the model

Table S6. Change in MetaCyc pathway relative abundance per doubling caffeine (ng/g)

Exposure Window	MetaCyc Pathway ID	Effect Estimate[a]	Std. Error	p-value	q-value
Meconium	P164.PWY	0.063	0.023	0.010	0.978
Meconium	P562.PWY	-0.074	0.034	0.032	0.978
Meconium	PWY0.1415	-0.132	0.059	0.031	0.978
Meconium	PWY.6285	-0.066	0.031	0.036	0.978
Meconium	PWY.6936	0.023	0.011	0.037	0.978
Meconium	FAO.PWY	0.101	0.053	0.063	0.978
Meconium	HEXITOLDEGSUPER.PWY	-0.023	0.014	0.101	0.978
Meconium	HSERMETANA.PWY	0.018	0.010	0.085	0.978
Meconium	OANTIGEN.PWY	0.017	0.010	0.097	0.978
Meconium	P161.PWY	0.138	0.087	0.120	0.978
Meconium	P461.PWY	-0.039	0.024	0.115	0.978
Meconium	PWY.1269	-0.079	0.049	0.115	0.978
Meconium	PWY.3781	-0.116	0.072	0.114	0.978
Meconium	PWY.5004	-0.062	0.038	0.112	0.978
Meconium	PWY.5136	0.106	0.054	0.057	0.978
Meconium	PWY.6588	0.132	0.070	0.067	0.978
Meconium	PWY.7003	0.119	0.063	0.066	0.978
Meconium	PWY.7094	-0.044	0.025	0.088	0.978
Meconium	PWY.7312	-0.073	0.045	0.110	0.978
Meconium	PWY.7385	-0.110	0.056	0.055	0.978
Meconium	PWY0.1241	-0.134	0.083	0.113	0.978
Meconium	PWY0.1261	-0.095	0.059	0.116	0.978
Meconium	PWY4LZ.257	0.136	0.086	0.121	0.978
Meconium	PWY66.398	-0.118	0.063	0.068	0.978
Meconium	PWY.5863	-0.108	0.069	0.126	0.978
Meconium	PWY.6471	0.040	0.026	0.127	0.978
Meconium	HEME.BIOSYNTHESIS.II	-0.115	0.076	0.139	0.978
Meconium	PWY.5656	-0.068	0.044	0.133	0.978
Meconium	PWY.5677	0.070	0.046	0.135	0.978
Meconium	PWY.6837	-0.038	0.025	0.146	0.978
Meconium	PWY66.409	0.031	0.021	0.147	0.978
Meconium	NAGLIPASYN.PWY	-0.103	0.076	0.183	0.978
Meconium	PWY.4722	-0.089	0.064	0.170	0.978
Meconium	PWY.5392	-0.054	0.039	0.173	0.978
Meconium	PWY.5791	-0.094	0.070	0.189	0.978
Meconium	PWY.5837	-0.094	0.070	0.189	0.978

Exposure Window	MetaCyc Pathway ID	Effect Estimate[a]	Std. Error	p-value	q-value
Meconium	PWY.5897	-0.093	0.068	0.177	0.978
Meconium	PWY.5898	-0.093	0.068	0.177	0.978
Meconium	PWY.5899	-0.093	0.068	0.177	0.978
Meconium	PWY.6549	0.023	0.017	0.185	0.978
Meconium	PWY.6895	-0.083	0.058	0.161	0.978
Meconium	X1CMET2.PWY	0.001	0.005	0.796	0.978
Meconium	X7ALPHADEHYDROX.PWY	-0.024	0.064	0.713	0.978
Meconium	ANAGLYCOLYSIS.PWY	-0.006	0.009	0.534	0.978
Meconium	ARG.POLYAMINE.SYN	0.024	0.040	0.552	0.978
Meconium	ARGININE.SYN4.PWY	-0.097	0.080	0.232	0.978
Meconium	ARGORNPROST.PWY	-0.032	0.049	0.515	0.978
Meconium	ARGSYN.PWY	0.004	0.007	0.548	0.978
Meconium	ARGSYNBSUB.PWY	0.005	0.007	0.471	0.978
Meconium	ARO.PWY	0.007	0.007	0.313	0.978
Meconium	ASPASN.PWY	0.005	0.012	0.714	0.978
Meconium	BIOTIN.BIOSYNTHESIS.PWY	-0.042	0.082	0.612	0.978
Meconium	BRANCHED.CHAIN.AA.SYN.PWY	0.004	0.007	0.570	0.978
Meconium	CALVIN.PWY	0.006	0.009	0.516	0.978
Meconium	COA.PWY	0.004	0.007	0.620	0.978
Meconium	COBALSYN.PWY	0.014	0.014	0.329	0.978
Meconium	COMPLETE.ARO.PWY	0.006	0.007	0.452	0.978
Meconium	CRNFORCAT.PWY	0.040	0.049	0.410	0.978
Meconium	DENOVOPURINE2.PWY	0.015	0.017	0.396	0.978
Meconium	DTDPRHAMSYN.PWY	0.012	0.010	0.254	0.978
Meconium	FASYN.ELONG.PWY	-0.038	0.054	0.486	0.978
Meconium	FASYN.INITIAL.PWY	-0.019	0.066	0.778	0.978
Meconium	FERMENTATION.PWY	0.018	0.018	0.342	0.978
Meconium	FOLSYN.PWY	0.054	0.042	0.205	0.978
Meconium	FUC.RHAMCAT.PWY	-0.014	0.050	0.776	0.978
Meconium	FUCCAT.PWY	-0.015	0.037	0.691	0.978
Meconium	GALACT.GLUCUROCAT.PWY	0.022	0.052	0.680	0.978
Meconium	GALACTARDEG.PWY	0.040	0.087	0.648	0.978
Meconium	GALACTUROCAT.PWY	-0.016	0.042	0.705	0.978
Meconium	GLUCARDEG.PWY	0.032	0.081	0.698	0.978
Meconium	GLUCARGALACTSUPER.PWY	0.040	0.087	0.648	0.978
Meconium	GLUCONEO.PWY	-0.008	0.011	0.461	0.978
Meconium	GLUCOSE1PMETAB.PWY	-0.052	0.062	0.407	0.978

Exposure Window	MetaCyc Pathway ID	Effect Estimate[a]	Std. Error	p-value	q-value
Meconium	GLUDEG.I.PWY	-0.058	0.062	0.350	0.978
Meconium	GLUTORN.PWY	0.006	0.009	0.495	0.978
Meconium	GLYCOCAT.PWY	-0.048	0.059	0.424	0.978
Meconium	GLYCOGENSYNTH.PWY	0.012	0.011	0.291	0.978
Meconium	GLYCOL.GLYOXDEG.PWY	0.021	0.064	0.742	0.978
Meconium	GLYCOLYSIS.E.D	-0.018	0.059	0.762	0.978
Meconium	GLYCOLYSIS.TCA.GLYOX.BYPASS	-0.022	0.065	0.734	0.978
Meconium	GLYOXYLATE.BYPASS	-0.044	0.079	0.584	0.978
Meconium	GOLPDLCAT.PWY	0.008	0.025	0.757	0.978
Meconium	HCAMHPDEG.PWY	-0.016	0.033	0.630	0.978
Meconium	HEMESYN2.PWY	-0.048	0.040	0.238	0.978
Meconium	HISDEG.PWY	-0.045	0.048	0.359	0.978
Meconium	HISTSYN.PWY	0.006	0.007	0.436	0.978
Meconium	KETOGLUCONMET.PWY	-0.146	0.114	0.206	0.978
Meconium	LACTOSECAT.PWY	0.047	0.038	0.226	0.978
Meconium	MET.SAM.PWY	0.009	0.020	0.675	0.978
Meconium	METH.ACETATE.PWY	0.025	0.023	0.268	0.978
Meconium	METHANOGENESIS.PWY	-0.049	0.059	0.416	0.978
Meconium	METHGLYUT.PWY	-0.068	0.073	0.355	0.978
Meconium	METSYN.PWY	0.006	0.022	0.775	0.978
Meconium	NAD.BIOSYNTHESIS.II	-0.059	0.109	0.590	0.978
Meconium	NONOXIPENT.PWY	0.006	0.013	0.636	0.978
Meconium	P122.PWY	-0.044	0.112	0.699	0.978
Meconium	P124.PWY	0.034	0.061	0.578	0.978
Meconium	P125.PWY	-0.055	0.109	0.618	0.978
Meconium	P162.PWY	0.022	0.041	0.593	0.978
Meconium	P185.PWY	-0.018	0.029	0.531	0.978
Meconium	P23.PWY	-0.071	0.092	0.443	0.978
Meconium	P42.PWY	0.026	0.049	0.602	0.978
Meconium	P441.PWY	0.037	0.032	0.261	0.978
Meconium	P621.PWY	-0.021	0.071	0.774	0.978
Meconium	PANTOSYN.PWY	0.003	0.009	0.759	0.978
Meconium	PENTOSE.P.PWY	0.032	0.045	0.488	0.978
Meconium	PEPTIDOGLYCANSYN.PWY	0.003	0.004	0.492	0.978
Meconium	PHOSLIPSYN.PWY	0.026	0.033	0.430	0.978
Meconium	POLYAMINSYN3.PWY	-0.025	0.073	0.738	0.978
Meconium	POLYAMSYN.PWY	0.023	0.043	0.588	0.978

Exposure Window	MetaCyc Pathway ID	Effect Estimate[a]	Std. Error	p-value	q-value
Meconium	POLYISOPRENSYN.PWY	-0.039	0.065	0.555	0.978
Meconium	PPGPPMET.PWY	0.012	0.023	0.601	0.978
Meconium	PROPFERM.PWY	0.008	0.024	0.738	0.978
Meconium	PRPP.PWY	0.031	0.034	0.373	0.978
Meconium	PWY.1861	-0.014	0.029	0.626	0.978
Meconium	PWY.2723	-0.055	0.063	0.383	0.978
Meconium	PWY.3001	0.002	0.007	0.741	0.978
Meconium	PWY.3841	-0.002	0.004	0.671	0.978
Meconium	PWY.4041	-0.011	0.020	0.600	0.978
Meconium	PWY.4242	0.004	0.008	0.584	0.978
Meconium	PWY.4702	-0.082	0.066	0.217	0.978
Meconium	PWY.4981	0.037	0.032	0.253	0.978
Meconium	PWY.5005	-0.074	0.089	0.407	0.978
Meconium	PWY.5022	-0.032	0.055	0.569	0.978
Meconium	PWY.5030	-0.060	0.055	0.281	0.978
Meconium	PWY.5083	-0.038	0.087	0.663	0.978
Meconium	PWY.5097	0.002	0.005	0.662	0.978
Meconium	PWY.5100	0.011	0.012	0.334	0.978
Meconium	PWY.5101	-0.057	0.125	0.654	0.978
Meconium	PWY.5103	0.005	0.008	0.538	0.978
Meconium	PWY.5104	-0.009	0.021	0.654	0.978
Meconium	PWY.5121	-0.012	0.034	0.724	0.978
Meconium	PWY.5154	-0.023	0.036	0.521	0.978
Meconium	PWY.5173	0.032	0.094	0.736	0.978
Meconium	PWY.5177	-0.016	0.021	0.448	0.978
Meconium	PWY.5188	-0.007	0.017	0.680	0.978
Meconium	PWY.5189	0.042	0.071	0.557	0.978
Meconium	PWY.5198	-0.038	0.055	0.489	0.978
Meconium	PWY.5265	-0.033	0.064	0.613	0.978
Meconium	PWY.5304	-0.019	0.041	0.637	0.978
Meconium	PWY.5367	0.055	0.076	0.474	0.978
Meconium	PWY.5384	-0.018	0.043	0.679	0.978
Meconium	PWY.5464	-0.096	0.076	0.213	0.978
Meconium	PWY.5505	-0.090	0.075	0.241	0.978
Meconium	PWY.561	-0.025	0.075	0.736	0.978
Meconium	PWY.5675	-0.052	0.071	0.473	0.978
Meconium	PWY.5676	-0.016	0.023	0.507	0.978

Exposure Window	MetaCyc Pathway ID	Effect Estimate[a]	Std. Error	p-value	q-value
Meconium	PWY.5686	0.003	0.005	0.519	0.978
Meconium	PWY.5690	-0.023	0.023	0.333	0.978
Meconium	PWY.5695	0.007	0.007	0.369	0.978
Meconium	PWY.5723	-0.017	0.057	0.766	0.978
Meconium	PWY.5747	-0.068	0.072	0.351	0.978
Meconium	PWY.5838	-0.076	0.072	0.302	0.978
Meconium	PWY.5840	-0.044	0.065	0.499	0.978
Meconium	PWY.5845	-0.032	0.076	0.680	0.978
Meconium	PWY.5850	0.013	0.038	0.724	0.978
Meconium	PWY.5860	0.013	0.038	0.730	0.978
Meconium	PWY.5861	-0.076	0.074	0.305	0.978
Meconium	PWY.5862	-0.033	0.077	0.675	0.978
Meconium	PWY.5896	0.013	0.038	0.724	0.978
Meconium	PWY.5918	-0.079	0.069	0.261	0.978
Meconium	PWY.5920	-0.039	0.048	0.419	0.978
Meconium	PWY.5941	-0.028	0.103	0.790	0.978
Meconium	PWY.5971	-0.062	0.050	0.225	0.978
Meconium	PWY.5973	-0.033	0.047	0.490	0.978
Meconium	PWY.5994	-0.091	0.081	0.267	0.978
Meconium	PWY.6113	-0.040	0.047	0.403	0.978
Meconium	PWY.6121	0.006	0.007	0.388	0.978
Meconium	PWY.6122	0.006	0.006	0.362	0.978
Meconium	PWY.6123	0.003	0.008	0.754	0.978
Meconium	PWY.6124	0.002	0.009	0.791	0.978
Meconium	PWY.6125	0.019	0.021	0.383	0.978
Meconium	PWY.6147	-0.047	0.042	0.273	0.978
Meconium	PWY.6151	0.003	0.008	0.696	0.978
Meconium	PWY.6163	0.008	0.007	0.299	0.978
Meconium	PWY.6167	-0.047	0.053	0.385	0.978
Meconium	PWY.6168	-0.010	0.013	0.478	0.978
Meconium	PWY.621	-0.021	0.021	0.322	0.978
Meconium	PWY.622	-0.021	0.059	0.718	0.978
Meconium	PWY.6263	-0.022	0.070	0.749	0.978
Meconium	PWY.6277	0.006	0.006	0.362	0.978
Meconium	PWY.6282	-0.032	0.058	0.589	0.978
Meconium	PWY.6305	0.014	0.020	0.501	0.978
Meconium	PWY.6317	0.006	0.014	0.635	0.978

Exposure Window	MetaCyc Pathway ID	Effect Estimate[a]	Std. Error	p-value	q-value
Meconium	PWY.6349	-0.052	0.061	0.398	0.978
Meconium	PWY.6353	0.008	0.015	0.624	0.978
Meconium	PWY.6385	0.003	0.005	0.574	0.978
Meconium	PWY.6386	0.003	0.005	0.511	0.978
Meconium	PWY.6387	0.002	0.004	0.590	0.978
Meconium	PWY.6396	-0.032	0.041	0.448	0.978
Meconium	PWY.6435	0.045	0.088	0.611	0.978
Meconium	PWY.6470	0.009	0.033	0.787	0.978
Meconium	PWY.6507	-0.020	0.045	0.661	0.978
Meconium	PWY.6519	-0.044	0.085	0.608	0.978
Meconium	PWY.6527	0.006	0.016	0.703	0.978
Meconium	PWY.6531	-0.039	0.085	0.651	0.978
Meconium	PWY.6545	0.017	0.023	0.463	0.978
Meconium	PWY.6572	0.020	0.032	0.529	0.978
Meconium	PWY.6606	0.011	0.018	0.538	0.978
Meconium	PWY.6608	0.016	0.019	0.397	0.978
Meconium	PWY.6609	0.004	0.009	0.660	0.978
Meconium	PWY.6612	0.055	0.043	0.209	0.978
Meconium	PWY.6628	0.045	0.079	0.571	0.978
Meconium	PWY.6630	-0.017	0.054	0.750	0.978
Meconium	PWY.6690	-0.016	0.033	0.630	0.978
Meconium	PWY.6700	0.004	0.008	0.609	0.978
Meconium	PWY.6703	-0.005	0.014	0.706	0.978
Meconium	PWY.6731	-0.041	0.051	0.424	0.978
Meconium	PWY.6803	0.019	0.051	0.712	0.978
Meconium	PWY.6823	0.020	0.030	0.497	0.978
Meconium	PWY.6859	-0.038	0.090	0.675	0.978
Meconium	PWY.6891	0.029	0.047	0.545	0.978
Meconium	PWY.6892	0.005	0.017	0.747	0.978
Meconium	PWY.6897	0.012	0.018	0.537	0.978
Meconium	PWY.6901	0.024	0.039	0.538	0.978
Meconium	PWY.7013	-0.026	0.028	0.361	0.978
Meconium	PWY.7111	0.002	0.007	0.795	0.978
Meconium	PWY.7115	-0.024	0.034	0.486	0.978
Meconium	PWY.7184	0.019	0.024	0.437	0.978
Meconium	PWY.7187	0.018	0.019	0.349	0.978
Meconium	PWY.7196	0.015	0.014	0.275	0.978

Exposure Window	MetaCyc Pathway ID	Effect Estimate[a]	Std. Error	p-value	q-value
Meconium	PWY.7197	0.022	0.027	0.427	0.978
Meconium	PWY.7198	0.020	0.026	0.440	0.978
Meconium	PWY.7199	0.006	0.011	0.622	0.978
Meconium	PWY.7204	-0.024	0.038	0.526	0.978
Meconium	PWY.7208	-0.021	0.018	0.255	0.978
Meconium	PWY.7209	0.050	0.084	0.553	0.978
Meconium	PWY.7210	-0.111	0.087	0.209	0.978
Meconium	PWY.7211	0.022	0.020	0.274	0.978
Meconium	PWY.7219	0.004	0.007	0.576	0.978
Meconium	PWY.7220	-0.010	0.020	0.631	0.978
Meconium	PWY.7221	0.006	0.005	0.263	0.978
Meconium	PWY.7222	-0.010	0.020	0.631	0.978
Meconium	PWY.7228	0.019	0.023	0.417	0.978
Meconium	PWY.7229	0.004	0.006	0.562	0.978
Meconium	PWY.7234	0.016	0.037	0.681	0.978
Meconium	PWY.7237	-0.008	0.019	0.682	0.978
Meconium	PWY.724	0.002	0.005	0.597	0.978
Meconium	PWY.7282	-0.024	0.069	0.730	0.978
Meconium	PWY.7286	-0.053	0.065	0.418	0.978
Meconium	PWY.7288	0.038	0.060	0.527	0.978
Meconium	PWY.7315	0.025	0.055	0.648	0.978
Meconium	PWY.7316	0.037	0.064	0.569	0.978
Meconium	PWY.7323	0.022	0.032	0.499	0.978
Meconium	PWY.7328	-0.052	0.057	0.360	0.978
Meconium	PWY.7332	-0.073	0.077	0.348	0.978
Meconium	PWY.7357	0.009	0.011	0.375	0.978
Meconium	PWY.7371	-0.095	0.088	0.290	0.978
Meconium	PWY.7388	-0.019	0.067	0.773	0.978
Meconium	PWY.7392	-0.035	0.083	0.670	0.978
Meconium	PWY.7400	0.004	0.007	0.545	0.978
Meconium	PWY.7456	0.033	0.030	0.271	0.978
Meconium	PWY.7539	-0.047	0.042	0.265	0.978
Meconium	PWY.7616	-0.095	0.104	0.366	0.978
Meconium	PWY.7663	-0.030	0.051	0.555	0.978
Meconium	PWY.7664	-0.039	0.059	0.512	0.978
Meconium	PWY.821	-0.070	0.082	0.403	0.978
Meconium	PWY.841	0.016	0.018	0.374	0.978

Exposure Window	MetaCyc Pathway ID	Effect Estimate[a]	Std. Error	p-value	q-value
Meconium	PWY0.1061	-0.021	0.043	0.623	0.978
Meconium	PWY0.1277	-0.012	0.025	0.651	0.978
Meconium	PWY0.1296	0.005	0.012	0.661	0.978
Meconium	PWY0.1297	0.030	0.029	0.309	0.978
Meconium	PWY0.1298	-0.017	0.038	0.658	0.978
Meconium	PWY0.162	-0.005	0.014	0.691	0.978
Meconium	PWY0.166	0.016	0.019	0.397	0.978
Meconium	PWY0.42	-0.040	0.036	0.281	0.978
Meconium	PWY0.845	-0.054	0.078	0.490	0.978
Meconium	PWY0.862	-0.040	0.060	0.511	0.978
Meconium	PWY0.881	-0.045	0.067	0.500	0.978
Meconium	PWY490.3	0.049	0.044	0.270	0.978
Meconium	PWY4FS.7	0.028	0.036	0.438	0.978
Meconium	PWY4FS.8	0.028	0.036	0.438	0.978
Meconium	PWY66.391	0.044	0.058	0.456	0.978
Meconium	PWY66.399	-0.011	0.016	0.476	0.978
Meconium	PWY66.400	0.009	0.021	0.671	0.978
Meconium	PWY66.422	0.012	0.009	0.210	0.978
Meconium	PWYG.321	-0.033	0.059	0.578	0.978
Meconium	PYRIDNUCSAL.PWY	0.042	0.057	0.465	0.978
Meconium	PYRIDOXSYN.PWY	-0.077	0.079	0.339	0.978
Meconium	RHAMCAT.PWY	-0.014	0.021	0.502	0.978
Meconium	RIBOSYN2.PWY	-0.006	0.012	0.630	0.978
Meconium	RUMP.PWY	0.061	0.087	0.488	0.978
Meconium	SALVADEHYPOX.PWY	0.005	0.018	0.771	0.978
Meconium	SER.GLYSYN.PWY	0.008	0.008	0.322	0.978
Meconium	TCA.GLYOX.BYPASS	-0.022	0.068	0.751	0.978
Meconium	TCA	-0.022	0.023	0.348	0.978
Meconium	TEICHOICACID.PWY	0.010	0.020	0.622	0.978
Meconium	THISYN.PWY	-0.020	0.032	0.536	0.978
Meconium	THISYNARA.PWY	0.009	0.019	0.652	0.978
Meconium	THREOCAT.PWY	0.022	0.032	0.499	0.978
Meconium	TRNA.CHARGING.PWY	0.001	0.005	0.757	0.978
Meconium	UDPNAGSYN.PWY	0.017	0.015	0.266	0.978
Meconium	PWY.2941	0.008	0.029	0.800	0.978
Meconium	P221.PWY	-0.017	0.070	0.809	0.978
Meconium	PWY0.1586	-0.005	0.022	0.806	0.978

Exposure Window	MetaCyc Pathway ID	Effect Estimate[a]	Std. Error	p-value	q-value
Meconium	PWY.5138	0.018	0.075	0.815	0.978
Meconium	UNMAPPED	-0.001	0.007	0.897	0.978
Meconium	ALLANTOINDEG.PWY	-0.007	0.045	0.875	0.978
Meconium	ANAEROFRUCAT.PWY	-0.001	0.015	0.936	0.982
Meconium	ARGDEG.PWY	0.006	0.055	0.914	0.978
Meconium	CENTFERM.PWY	0.004	0.043	0.918	0.978
Meconium	CITRULBIO.PWY	-0.010	0.055	0.857	0.978
Meconium	COA.PWY.1	0.001	0.004	0.851	0.978
Meconium	COLANSYN.PWY	0.003	0.017	0.872	0.978
Meconium	DAPLYSINESYN.PWY	0.002	0.025	0.945	0.984
Meconium	ENTBACSYN.PWY	-0.005	0.030	0.860	0.978
Meconium	HOMOSER.METSYN.PWY	0.006	0.026	0.824	0.978
Meconium	ILEUSYN.PWY	0.001	0.007	0.857	0.978
Meconium	NONMEVIPP.PWY	0.007	0.033	0.842	0.978
Meconium	ORNARGDEG.PWY	0.006	0.055	0.914	0.978
Meconium	ORNDEG.PWY	-0.007	0.033	0.828	0.978
Meconium	P105.PWY	-0.014	0.066	0.829	0.978
Meconium	P108.PWY	-0.022	0.106	0.837	0.978
Meconium	P4.PWY	0.004	0.033	0.904	0.978
Meconium	PANTO.PWY	0.002	0.011	0.875	0.978
Meconium	PWY.1042	0.001	0.007	0.871	0.978
Meconium	PWY.241	0.003	0.027	0.924	0.978
Meconium	PWY.4984	-0.012	0.055	0.835	0.978
Meconium	PWY.5088	0.002	0.021	0.933	0.982
Meconium	PWY.5345	0.009	0.072	0.898	0.978
Meconium	PWY.5347	0.003	0.019	0.890	0.978
Meconium	PWY.5484	-0.003	0.021	0.900	0.978
Meconium	PWY.5659	0.001	0.023	0.949	0.984
Meconium	PWY.5910	0.005	0.048	0.915	0.978
Meconium	PWY.5913	0.007	0.033	0.822	0.978
Meconium	PWY.5989	-0.006	0.057	0.915	0.978
Meconium	PWY.6126	0.002	0.010	0.876	0.978
Meconium	PWY.6270	0.004	0.045	0.922	0.978
Meconium	PWY.6284	-0.008	0.052	0.879	0.978
Meconium	PWY.6318	0.006	0.036	0.867	0.978
Meconium	PWY.6478	0.010	0.047	0.839	0.978
Meconium	PWY.6590	0.005	0.042	0.898	0.978

Exposure Window	MetaCyc Pathway ID	Effect Estimate[a]	Std. Error	p-value	q-value
Meconium	PWY.6629	0.011	0.054	0.836	0.978
Meconium	PWY.6737	0.001	0.008	0.911	0.978
Meconium	PWY.6749	0.012	0.053	0.829	0.978
Meconium	PWY.6876	0.013	0.059	0.830	0.978
Meconium	PWY.7046	0.012	0.071	0.867	0.978
Meconium	PWY.7117	0.003	0.031	0.926	0.978
Meconium	PWY.7254	-0.007	0.069	0.920	0.978
Meconium	PWY.7560	0.004	0.046	0.927	0.978
Meconium	PWY.922	0.004	0.057	0.948	0.984
Meconium	PWY0.781	0.002	0.030	0.944	0.984
Meconium	PWY3O.355	0.013	0.081	0.875	0.978
Meconium	PWY66.389	-0.010	0.101	0.921	0.978
Meconium	PYRIDNUCSYN.PWY	0.002	0.008	0.826	0.978
Meconium	REDCITCYC	-0.013	0.074	0.859	0.978
Meconium	SULFATE.CYS.PWY	0.009	0.073	0.902	0.978
Meconium	THRESYN.PWY	0.001	0.008	0.875	0.978
Meconium	TRPSYN.PWY	-0.001	0.014	0.918	0.978
Meconium	VALSYN.PWY	0.001	0.007	0.857	0.978
Meconium	SO4ASSIM.PWY	0.005	0.076	0.953	0.986
Meconium	UNINTEGRATED	0.000	0.002	0.959	0.987
Meconium	PWY.2942	0.000	0.005	0.962	0.987
Meconium	PWY.5667	0.000	0.010	0.965	0.987
Meconium	PWY.7383	0.001	0.020	0.969	0.988
Meconium	PWY0.1319	0.000	0.010	0.965	0.987
Meconium	GLYCOLYSIS	0.001	0.018	0.975	0.990
Meconium	GLUCUROCAT.PWY	0.001	0.041	0.979	0.990
Meconium	PWY0.1479	-0.001	0.023	0.979	0.990
Meconium	GLCMANNANAUT.PWY	0.000	0.022	0.987	0.995
Meconium	PWY.6969	0.000	0.030	0.990	0.996
Meconium	PWY.6595	0.001	0.090	0.993	0.996
Meconium	PWY.7242	0.000	0.045	0.997	0.997
Cross-sectional	PWY.5345	0.235	0.058	0.000	0.018
Cross-sectional	SO4ASSIM.PWY	0.243	0.061	0.000	0.018
Cross-sectional	SULFATE.CYS.PWY	0.237	0.058	0.000	0.018
Cross-sectional	UNMAPPED	0.012	0.005	0.021	0.460
Cross-sectional	UNINTEGRATED	-0.003	0.002	0.026	0.460
Cross-sectional	CENTFERM.PWY	0.081	0.029	0.006	0.330

Exposure Window	MetaCyc Pathway ID	Effect Estimate[a]	Std. Error	p-value	q-value
Cross-sectional	KETOGLUCONMET.PWY	0.248	0.093	0.009	0.351
Cross-sectional	P161.PWY	0.164	0.062	0.010	0.351
Cross-sectional	POLYAMINSYN3.PWY	0.170	0.058	0.005	0.330
Cross-sectional	PWY.5791	-0.126	0.056	0.028	0.460
Cross-sectional	PWY.5837	-0.126	0.056	0.028	0.460
Cross-sectional	PWY.5863	-0.124	0.056	0.030	0.460
Cross-sectional	PWY.5897	-0.121	0.055	0.030	0.460
Cross-sectional	PWY.5898	-0.121	0.055	0.030	0.460
Cross-sectional	PWY.5899	-0.121	0.055	0.030	0.460
Cross-sectional	PWY.6588	0.120	0.054	0.028	0.460
Cross-sectional	PWY.6590	0.080	0.028	0.006	0.330
Cross-sectional	PWY.7003	0.106	0.048	0.029	0.460
Cross-sectional	PWY.7220	0.033	0.015	0.030	0.460
Cross-sectional	PWY.7222	0.033	0.015	0.030	0.460
Cross-sectional	PWY.821	0.172	0.060	0.005	0.330
Cross-sectional	PWY0.1298	0.084	0.033	0.014	0.454
Cross-sectional	PWY4LZ.257	0.163	0.061	0.010	0.351
Cross-sectional	PWY0.1415	0.145	0.066	0.030	0.460
Cross-sectional	NAD.BIOSYNTHESIS.II	-0.192	0.095	0.047	0.547
Cross-sectional	PWY.1861	-0.042	0.021	0.046	0.547
Cross-sectional	PWY.5177	-0.034	0.017	0.051	0.583
Cross-sectional	PWY.5189	0.113	0.054	0.040	0.518
Cross-sectional	PWY.5840	-0.110	0.052	0.036	0.518
Cross-sectional	PWY.5918	0.133	0.063	0.038	0.518
Cross-sectional	PWY.7229	0.012	0.006	0.043	0.536
Cross-sectional	PWY0.1479	0.041	0.020	0.039	0.518
Cross-sectional	PWY.6305	-0.033	0.017	0.054	0.597
Cross-sectional	PWY.6126	0.016	0.008	0.058	0.616
Cross-sectional	CITRULBIO.PWY	0.076	0.040	0.062	0.628
Cross-sectional	P108.PWY	0.134	0.071	0.062	0.628
Cross-sectional	PWY.4984	0.076	0.041	0.066	0.654
Cross-sectional	RUMP.PWY	0.108	0.060	0.076	0.725
Cross-sectional	PWY.6969	0.040	0.023	0.078	0.725
Cross-sectional	X1CMET2.PWY	0.001	0.004	0.732	0.992
Cross-sectional	X7ALPHADEHYDROX.PWY	-0.005	0.042	0.902	0.998
Cross-sectional	ALLANTOINDEG.PWY	-0.009	0.046	0.848	0.992
Cross-sectional	ANAEROFRUCAT.PWY	0.011	0.012	0.381	0.992

Exposure Window	MetaCyc Pathway ID	Effect Estimate[a]	Std. Error	p-value	q-value
Cross-sectional	ANAGLYCOLYSIS.PWY	-0.003	0.007	0.666	0.992
Cross-sectional	ARG.POLYAMINE.SYN	0.001	0.030	0.970	0.998
Cross-sectional	ARGDEG.PWY	-0.022	0.049	0.661	0.992
Cross-sectional	ARGININE.SYN4.PWY	-0.052	0.069	0.453	0.992
Cross-sectional	ARGORNPROST.PWY	0.032	0.045	0.473	0.992
Cross-sectional	ARGSYN.PWY	-0.002	0.005	0.630	0.992
Cross-sectional	ARGSYNBSUB.PWY	-0.002	0.005	0.755	0.992
Cross-sectional	ARO.PWY	-0.004	0.005	0.447	0.992
Cross-sectional	ASPASN.PWY	-0.007	0.010	0.493	0.992
Cross-sectional	AST.PWY	-0.012	0.030	0.701	0.992
Cross-sectional	BIOTIN.BIOSYNTHESIS.PWY	-0.003	0.064	0.964	0.998
Cross-sectional	BRANCHED.CHAIN.AA.SYN.PWY	-0.003	0.005	0.640	0.992
Cross-sectional	CALVIN.PWY	-0.006	0.007	0.369	0.992
Cross-sectional	COA.PWY.1	0.003	0.003	0.384	0.992
Cross-sectional	COA.PWY	-0.006	0.005	0.231	0.992
Cross-sectional	COBALSYN.PWY	-0.016	0.012	0.215	0.992
Cross-sectional	COLANSYN.PWY	0.007	0.015	0.647	0.992
Cross-sectional	COMPLETE.ARO.PWY	-0.004	0.005	0.432	0.992
Cross-sectional	CRNFORCAT.PWY	-0.064	0.040	0.109	0.943
Cross-sectional	DAPLYSINESYN.PWY	0.005	0.019	0.816	0.992
Cross-sectional	DENOVOPURINE2.PWY	0.011	0.016	0.492	0.992
Cross-sectional	DTDPRHAMSYN.PWY	-0.005	0.007	0.525	0.992
Cross-sectional	ENTBACSYN.PWY	-0.016	0.042	0.711	0.992
Cross-sectional	FAO.PWY	0.058	0.044	0.196	0.992
Cross-sectional	FASYN.ELONG.PWY	0.002	0.037	0.961	0.998
Cross-sectional	FASYN.INITIAL.PWY	-0.021	0.046	0.656	0.992
Cross-sectional	FERMENTATION.PWY	0.007	0.018	0.684	0.992
Cross-sectional	FOLSYN.PWY	0.039	0.029	0.175	0.992
Cross-sectional	FUC.RHAMCAT.PWY	0.044	0.043	0.316	0.992
Cross-sectional	FUCCAT.PWY	-0.027	0.028	0.340	0.992
Cross-sectional	GALACT.GLUCUROCAT.PWY	0.023	0.045	0.608	0.992
Cross-sectional	GALACTARDEG.PWY	0.069	0.069	0.319	0.992
Cross-sectional	GALACTUROCAT.PWY	0.017	0.034	0.619	0.992
Cross-sectional	GLCMANNANAUT.PWY	-0.025	0.019	0.184	0.992
Cross-sectional	GLUCARDEG.PWY	0.061	0.066	0.356	0.992
Cross-sectional	GLUCARGALACTSUPER.PWY	0.069	0.069	0.319	0.992
Cross-sectional	GLUCONEO.PWY	0.000	0.008	0.966	0.998

Exposure Window	MetaCyc Pathway ID	Effect Estimate[a]	Std. Error	p-value	q-value
Cross-sectional	GLUCOSE1PMETAB.PWY	0.014	0.049	0.785	0.992
Cross-sectional	GLUCUROCAT.PWY	0.004	0.035	0.919	0.998
Cross-sectional	GLUDEG.I.PWY	0.034	0.055	0.536	0.992
Cross-sectional	GLUTORN.PWY	-0.004	0.006	0.523	0.992
Cross-sectional	GLYCOCAT.PWY	0.010	0.047	0.837	0.992
Cross-sectional	GLYCOGENSYNTH.PWY	-0.010	0.009	0.266	0.992
Cross-sectional	GLYCOL.GLYOXDEG.PWY	0.030	0.055	0.584	0.992
Cross-sectional	GLYCOLYSIS.E.D	-0.031	0.045	0.490	0.992
Cross-sectional	GLYCOLYSIS.TCA.GLYOX.BYPASS	0.037	0.058	0.525	0.992
Cross-sectional	GLYCOLYSIS	0.008	0.015	0.581	0.992
Cross-sectional	GLYOXYLATE.BYPASS	0.028	0.065	0.668	0.992
Cross-sectional	GOLPDLCAT.PWY	-0.010	0.019	0.608	0.992
Cross-sectional	HCAMHPDEG.PWY	0.009	0.032	0.779	0.992
Cross-sectional	HEME.BIOSYNTHESIS.II	0.015	0.069	0.832	0.992
Cross-sectional	HEMESYN2.PWY	0.037	0.032	0.254	0.992
Cross-sectional	HEXITOLDEGSUPER.PWY	-0.003	0.013	0.828	0.992
Cross-sectional	HISDEG.PWY	0.047	0.035	0.186	0.992
Cross-sectional	HISTSYN.PWY	-0.005	0.005	0.310	0.992
Cross-sectional	HOMOSER.METSYN.PWY	0.022	0.021	0.299	0.992
Cross-sectional	HSERMETANA.PWY	-0.005	0.008	0.514	0.992
Cross-sectional	ILEUSYN.PWY	-0.007	0.005	0.159	0.992
Cross-sectional	LACTOSECAT.PWY	0.022	0.029	0.444	0.992
Cross-sectional	MET.SAM.PWY	0.018	0.016	0.283	0.992
Cross-sectional	METH.ACETATE.PWY	0.001	0.017	0.940	0.998
Cross-sectional	METHANOGENESIS.PWY	-0.004	0.077	0.958	0.998
Cross-sectional	METHGLYUT.PWY	-0.027	0.057	0.635	0.992
Cross-sectional	METSYN.PWY	0.019	0.018	0.282	0.992
Cross-sectional	NAGLIPASYN.PWY	0.006	0.080	0.944	0.998
Cross-sectional	NONMEVIPP.PWY	0.012	0.027	0.652	0.992
Cross-sectional	NONOXIPENT.PWY	-0.011	0.010	0.287	0.992
Cross-sectional	OANTIGEN.PWY	0.006	0.008	0.505	0.992
Cross-sectional	ORNARGDEG.PWY	-0.022	0.049	0.661	0.992
Cross-sectional	ORNDEG.PWY	0.003	0.028	0.915	0.998
Cross-sectional	P105.PWY	0.046	0.056	0.416	0.992
Cross-sectional	P122.PWY	-0.032	0.081	0.696	0.992
Cross-sectional	P124.PWY	0.017	0.045	0.711	0.992
Cross-sectional	P125.PWY	-0.056	0.084	0.511	0.992

Exposure Window	MetaCyc Pathway ID	Effect Estimate[a]	Std. Error	p-value	q-value
Cross-sectional	P162.PWY	0.025	0.040	0.538	0.992
Cross-sectional	P164.PWY	0.008	0.018	0.666	0.992
Cross-sectional	P185.PWY	-0.021	0.022	0.339	0.992
Cross-sectional	P221.PWY	0.019	0.081	0.812	0.992
Cross-sectional	P23.PWY	-0.037	0.066	0.576	0.992
Cross-sectional	P4.PWY	0.012	0.027	0.657	0.992
Cross-sectional	P42.PWY	0.037	0.040	0.362	0.992
Cross-sectional	P441.PWY	0.024	0.025	0.346	0.992
Cross-sectional	P461.PWY	-0.021	0.022	0.332	0.992
Cross-sectional	P562.PWY	-0.014	0.033	0.675	0.992
Cross-sectional	P621.PWY	-0.024	0.057	0.672	0.992
Cross-sectional	PANTO.PWY	0.001	0.010	0.910	0.998
Cross-sectional	PANTOSYN.PWY	-0.002	0.008	0.820	0.992
Cross-sectional	PENTOSE.P.PWY	0.011	0.031	0.728	0.992
Cross-sectional	PEPTIDOGLYCANSYN.PWY	0.002	0.003	0.591	0.992
Cross-sectional	PHOSLIPSYN.PWY	-0.006	0.023	0.808	0.992
Cross-sectional	POLYAMSYN.PWY	0.002	0.033	0.940	0.998
Cross-sectional	POLYISOPRENSYN.PWY	0.057	0.054	0.292	0.992
Cross-sectional	PPGPPMET.PWY	-0.019	0.017	0.274	0.992
Cross-sectional	PRPP.PWY	0.010	0.022	0.657	0.992
Cross-sectional	PWY.1042	-0.005	0.005	0.327	0.992
Cross-sectional	PWY.1269	-0.044	0.038	0.248	0.992
Cross-sectional	PWY.241	-0.006	0.023	0.812	0.992
Cross-sectional	PWY.2723	0.015	0.050	0.759	0.992
Cross-sectional	PWY.2941	0.021	0.023	0.383	0.992
Cross-sectional	PWY.2942	0.002	0.005	0.585	0.992
Cross-sectional	PWY.3001	0.000	0.006	0.935	0.998
Cross-sectional	PWY.3781	0.074	0.053	0.166	0.992
Cross-sectional	PWY.3841	0.003	0.003	0.369	0.992
Cross-sectional	PWY.4041	-0.011	0.016	0.484	0.992
Cross-sectional	PWY.4242	-0.009	0.006	0.171	0.992
Cross-sectional	PWY.4702	0.059	0.058	0.309	0.992
Cross-sectional	PWY.4722	0.011	0.048	0.820	0.992
Cross-sectional	PWY.4981	0.042	0.024	0.087	0.776
Cross-sectional	PWY.5004	0.038	0.036	0.293	0.992
Cross-sectional	PWY.5005	-0.036	0.070	0.610	0.992
Cross-sectional	PWY.5022	0.016	0.045	0.726	0.992

Exposure Window	MetaCyc Pathway ID	Effect Estimate[a]	Std. Error	p-value	q-value
Cross-sectional	PWY.5030	0.043	0.042	0.303	0.992
Cross-sectional	PWY.5083	-0.042	0.074	0.569	0.992
Cross-sectional	PWY.5097	-0.001	0.004	0.791	0.992
Cross-sectional	PWY.5100	0.002	0.010	0.831	0.992
Cross-sectional	PWY.5103	-0.002	0.006	0.789	0.992
Cross-sectional	PWY.5104	-0.016	0.018	0.377	0.992
Cross-sectional	PWY.5121	0.014	0.028	0.617	0.992
Cross-sectional	PWY.5136	0.061	0.046	0.187	0.992
Cross-sectional	PWY.5138	-0.077	0.069	0.270	0.992
Cross-sectional	PWY.5154	-0.026	0.028	0.350	0.992
Cross-sectional	PWY.5173	0.103	0.073	0.163	0.992
Cross-sectional	PWY.5188	-0.008	0.013	0.558	0.992
Cross-sectional	PWY.5198	-0.014	0.072	0.852	0.994
Cross-sectional	PWY.5347	0.015	0.016	0.359	0.992
Cross-sectional	PWY.5367	-0.013	0.062	0.839	0.992
Cross-sectional	PWY.5384	-0.004	0.034	0.910	0.998
Cross-sectional	PWY.5392	-0.021	0.033	0.519	0.992
Cross-sectional	PWY.5464	0.043	0.056	0.448	0.992
Cross-sectional	PWY.5484	0.011	0.017	0.526	0.992
Cross-sectional	PWY.5505	-0.047	0.062	0.449	0.992
Cross-sectional	PWY.561	0.049	0.063	0.442	0.992
Cross-sectional	PWY.5656	0.020	0.041	0.621	0.992
Cross-sectional	PWY.5659	-0.011	0.016	0.491	0.992
Cross-sectional	PWY.5667	-0.005	0.007	0.483	0.992
Cross-sectional	PWY.5675	0.050	0.062	0.426	0.992
Cross-sectional	PWY.5676	-0.023	0.018	0.200	0.992
Cross-sectional	PWY.5677	0.007	0.037	0.845	0.992
Cross-sectional	PWY.5686	-0.001	0.004	0.834	0.992
Cross-sectional	PWY.5690	0.015	0.018	0.397	0.992
Cross-sectional	PWY.5695	-0.006	0.006	0.301	0.992
Cross-sectional	PWY.5723	0.065	0.052	0.214	0.992
Cross-sectional	PWY.5747	0.041	0.061	0.510	0.992
Cross-sectional	PWY.5838	-0.049	0.058	0.395	0.992
Cross-sectional	PWY.5850	0.022	0.035	0.529	0.992
Cross-sectional	PWY.5860	0.022	0.035	0.529	0.992
Cross-sectional	PWY.5861	-0.051	0.058	0.390	0.992
Cross-sectional	PWY.5896	0.022	0.035	0.529	0.992

Exposure Window	MetaCyc Pathway ID	Effect Estimate[a]	Std. Error	p-value	q-value
Cross-sectional	PWY.5910	0.019	0.034	0.572	0.992
Cross-sectional	PWY.5913	-0.002	0.029	0.957	0.998
Cross-sectional	PWY.5920	0.026	0.043	0.546	0.992
Cross-sectional	PWY.5941	-0.015	0.073	0.841	0.992
Cross-sectional	PWY.5971	-0.022	0.043	0.612	0.992
Cross-sectional	PWY.5973	0.025	0.031	0.416	0.992
Cross-sectional	PWY.5989	-0.008	0.036	0.827	0.992
Cross-sectional	PWY.5994	-0.022	0.070	0.754	0.992
Cross-sectional	PWY.6113	-0.021	0.041	0.603	0.992
Cross-sectional	PWY.6121	-0.001	0.005	0.889	0.998
Cross-sectional	PWY.6122	-0.001	0.005	0.844	0.992
Cross-sectional	PWY.6123	-0.009	0.006	0.177	0.992
Cross-sectional	PWY.6124	-0.009	0.007	0.187	0.992
Cross-sectional	PWY.6125	0.016	0.020	0.409	0.992
Cross-sectional	PWY.6143	-0.033	0.033	0.320	0.992
Cross-sectional	PWY.6147	-0.032	0.037	0.392	0.992
Cross-sectional	PWY.6151	-0.002	0.006	0.753	0.992
Cross-sectional	PWY.6163	-0.001	0.005	0.838	0.992
Cross-sectional	PWY.6168	-0.005	0.011	0.646	0.992
Cross-sectional	PWY.621	-0.005	0.016	0.748	0.992
Cross-sectional	PWY.622	0.025	0.047	0.602	0.992
Cross-sectional	PWY.6263	-0.002	0.053	0.967	0.998
Cross-sectional	PWY.6270	0.012	0.035	0.727	0.992
Cross-sectional	PWY.6277	-0.001	0.005	0.844	0.992
Cross-sectional	PWY.6282	0.001	0.039	0.972	0.998
Cross-sectional	PWY.6284	-0.012	0.045	0.788	0.992
Cross-sectional	PWY.6285	0.027	0.043	0.529	0.992
Cross-sectional	PWY.6317	-0.016	0.011	0.148	0.992
Cross-sectional	PWY.6318	-0.047	0.033	0.156	0.992
Cross-sectional	PWY.6349	-0.012	0.050	0.813	0.992
Cross-sectional	PWY.6353	-0.004	0.014	0.768	0.992
Cross-sectional	PWY.6385	0.004	0.004	0.320	0.992
Cross-sectional	PWY.6386	0.001	0.004	0.840	0.992
Cross-sectional	PWY.6387	0.001	0.003	0.792	0.992
Cross-sectional	PWY.6435	0.054	0.069	0.430	0.992
Cross-sectional	PWY.6470	0.026	0.023	0.256	0.992
Cross-sectional	PWY.6471	-0.018	0.018	0.331	0.992

Exposure Window	MetaCyc Pathway ID	Effect Estimate[a]	Std. Error	p-value	q-value
Cross-sectional	PWY.6507	0.011	0.035	0.750	0.992
Cross-sectional	PWY.6519	-0.005	0.066	0.944	0.998
Cross-sectional	PWY.6527	-0.014	0.013	0.277	0.992
Cross-sectional	PWY.6531	-0.074	0.068	0.277	0.992
Cross-sectional	PWY.6545	0.019	0.020	0.330	0.992
Cross-sectional	PWY.6549	0.007	0.015	0.662	0.992
Cross-sectional	PWY.6562	-0.038	0.035	0.282	0.992
Cross-sectional	PWY.6572	0.017	0.026	0.517	0.992
Cross-sectional	PWY.6595	0.057	0.059	0.337	0.992
Cross-sectional	PWY.6606	-0.004	0.015	0.778	0.992
Cross-sectional	PWY.6608	-0.008	0.016	0.598	0.992
Cross-sectional	PWY.6609	0.002	0.007	0.729	0.992
Cross-sectional	PWY.6612	0.040	0.030	0.183	0.992
Cross-sectional	PWY.6628	0.022	0.065	0.737	0.992
Cross-sectional	PWY.6629	0.015	0.050	0.766	0.992
Cross-sectional	PWY.6630	0.014	0.049	0.784	0.992
Cross-sectional	PWY.6690	0.009	0.032	0.779	0.992
Cross-sectional	PWY.6700	-0.001	0.006	0.932	0.998
Cross-sectional	PWY.6703	0.012	0.014	0.358	0.992
Cross-sectional	PWY.6731	-0.007	0.042	0.872	0.998
Cross-sectional	PWY.6737	-0.008	0.006	0.205	0.992
Cross-sectional	PWY.6749	0.060	0.046	0.198	0.992
Cross-sectional	PWY.6803	-0.007	0.050	0.882	0.998
Cross-sectional	PWY.6823	0.014	0.061	0.820	0.992
Cross-sectional	PWY.6859	0.064	0.072	0.381	0.992
Cross-sectional	PWY.6876	0.064	0.046	0.162	0.992
Cross-sectional	PWY.6891	-0.018	0.033	0.587	0.992
Cross-sectional	PWY.6892	-0.020	0.013	0.131	0.992
Cross-sectional	PWY.6895	-0.047	0.040	0.249	0.992
Cross-sectional	PWY.6897	0.001	0.015	0.960	0.998
Cross-sectional	PWY.6901	0.008	0.026	0.762	0.992
Cross-sectional	PWY.6936	-0.001	0.009	0.880	0.998
Cross-sectional	PWY.7013	-0.027	0.023	0.231	0.992
Cross-sectional	PWY.7046	0.045	0.052	0.395	0.992
Cross-sectional	PWY.7094	0.013	0.034	0.694	0.992
Cross-sectional	PWY.7111	-0.005	0.005	0.308	0.992
Cross-sectional	PWY.7115	0.004	0.028	0.883	0.998

Exposure Window	MetaCyc Pathway ID	Effect Estimate[a]	Std. Error	p-value	q-value
Cross-sectional	PWY.7184	0.018	0.021	0.406	0.992
Cross-sectional	PWY.7187	0.011	0.017	0.520	0.992
Cross-sectional	PWY.7196	0.004	0.016	0.781	0.992
Cross-sectional	PWY.7197	0.014	0.024	0.553	0.992
Cross-sectional	PWY.7198	0.014	0.023	0.531	0.992
Cross-sectional	PWY.7199	0.001	0.009	0.887	0.998
Cross-sectional	PWY.7204	0.026	0.037	0.475	0.992
Cross-sectional	PWY.7208	0.002	0.017	0.894	0.998
Cross-sectional	PWY.7209	-0.010	0.066	0.879	0.998
Cross-sectional	PWY.7210	-0.029	0.071	0.689	0.992
Cross-sectional	PWY.7211	0.011	0.017	0.527	0.992
Cross-sectional	PWY.7219	0.000	0.005	0.950	0.998
Cross-sectional	PWY.7221	-0.001	0.004	0.747	0.992
Cross-sectional	PWY.7228	0.017	0.021	0.406	0.992
Cross-sectional	PWY.7234	-0.008	0.028	0.779	0.992
Cross-sectional	PWY.7237	-0.017	0.015	0.256	0.992
Cross-sectional	PWY.7254	0.032	0.059	0.583	0.992
Cross-sectional	PWY.7282	-0.041	0.056	0.466	0.992
Cross-sectional	PWY.7286	-0.007	0.060	0.907	0.998
Cross-sectional	PWY.7288	-0.016	0.052	0.762	0.992
Cross-sectional	PWY.7312	-0.021	0.044	0.631	0.992
Cross-sectional	PWY.7315	0.079	0.046	0.087	0.776
Cross-sectional	PWY.7316	0.050	0.047	0.287	0.992
Cross-sectional	PWY.7323	0.021	0.030	0.491	0.992
Cross-sectional	PWY.7328	0.013	0.046	0.783	0.992
Cross-sectional	PWY.7332	0.057	0.060	0.348	0.992
Cross-sectional	PWY.7357	-0.011	0.008	0.215	0.992
Cross-sectional	PWY.7371	-0.038	0.061	0.539	0.992
Cross-sectional	PWY.7383	0.024	0.016	0.139	0.992
Cross-sectional	PWY.7385	0.030	0.051	0.555	0.992
Cross-sectional	PWY.7388	-0.020	0.047	0.666	0.992
Cross-sectional	PWY.7392	0.065	0.066	0.326	0.992
Cross-sectional	PWY.7400	-0.002	0.005	0.631	0.992
Cross-sectional	PWY.7456	0.011	0.022	0.630	0.992
Cross-sectional	PWY.7539	-0.030	0.036	0.404	0.992
Cross-sectional	PWY.7560	0.012	0.036	0.738	0.992
Cross-sectional	PWY.7616	-0.021	0.073	0.770	0.992

Exposure Window	MetaCyc Pathway ID	Effect Estimate[a]	Std. Error	p-value	q-value
Cross-sectional	PWY.7663	0.006	0.034	0.865	0.998
Cross-sectional	PWY.7664	0.002	0.040	0.959	0.998
Cross-sectional	PWY.841	0.014	0.017	0.420	0.992
Cross-sectional	PWY.922	0.013	0.042	0.750	0.992
Cross-sectional	PWY0.1061	0.002	0.036	0.957	0.998
Cross-sectional	PWY0.1241	-0.048	0.061	0.438	0.992
Cross-sectional	PWY0.1261	0.050	0.044	0.253	0.992
Cross-sectional	PWY0.1277	0.006	0.026	0.805	0.992
Cross-sectional	PWY0.1296	-0.006	0.010	0.567	0.992
Cross-sectional	PWY0.1297	-0.007	0.027	0.806	0.992
Cross-sectional	PWY0.1319	-0.005	0.007	0.482	0.992
Cross-sectional	PWY0.1586	-0.008	0.016	0.600	0.992
Cross-sectional	PWY0.162	0.011	0.013	0.388	0.992
Cross-sectional	PWY0.166	0.015	0.017	0.371	0.992
Cross-sectional	PWY0.42	0.005	0.042	0.914	0.998
Cross-sectional	PWY0.781	0.015	0.026	0.562	0.992
Cross-sectional	PWY0.845	-0.056	0.063	0.380	0.992
Cross-sectional	PWY0.862	0.002	0.041	0.963	0.998
Cross-sectional	PWY0.881	0.008	0.069	0.906	0.998
Cross-sectional	PWY3DJ.35471	0.046	0.031	0.141	0.992
Cross-sectional	PWY3O.355	0.026	0.056	0.643	0.992
Cross-sectional	PWY490.3	0.009	0.031	0.774	0.992
Cross-sectional	PWY4FS.7	-0.009	0.027	0.736	0.992
Cross-sectional	PWY4FS.8	-0.009	0.027	0.736	0.992
Cross-sectional	PWY66.389	0.056	0.096	0.563	0.992
Cross-sectional	PWY66.391	-0.018	0.050	0.725	0.992
Cross-sectional	PWY66.398	0.069	0.046	0.143	0.992
Cross-sectional	PWY66.399	0.019	0.013	0.161	0.992
Cross-sectional	PWY66.400	0.010	0.017	0.563	0.992
Cross-sectional	PWY66.409	-0.005	0.021	0.811	0.992
Cross-sectional	PWY66.422	-0.009	0.008	0.271	0.992
Cross-sectional	PWYG.321	-0.003	0.040	0.944	0.998
Cross-sectional	PYRIDNUCSAL.PWY	-0.030	0.045	0.512	0.992
Cross-sectional	PYRIDNUCSYN.PWY	0.002	0.006	0.804	0.992
Cross-sectional	PYRIDOXSYN.PWY	-0.058	0.065	0.378	0.992
Cross-sectional	REDCITCYC	0.040	0.063	0.524	0.992
Cross-sectional	RHAMCAT.PWY	-0.001	0.021	0.964	0.998

Exposure Window	MetaCyc Pathway ID	Effect Estimate[a]	Std. Error	p-value	q-value
Cross-sectional	RIBOSYN2.PWY	-0.003	0.011	0.817	0.992
Cross-sectional	SALVADEHYPOX.PWY	0.001	0.016	0.943	0.998
Cross-sectional	SER.GLYSYN.PWY	-0.002	0.007	0.746	0.992
Cross-sectional	TCA.GLYOX.BYPASS	0.038	0.059	0.525	0.992
Cross-sectional	TCA	0.016	0.017	0.329	0.992
Cross-sectional	TEICHOICACID.PWY	-0.002	0.016	0.889	0.998
Cross-sectional	THISYN.PWY	-0.007	0.023	0.753	0.992
Cross-sectional	THISYNARA.PWY	-0.004	0.016	0.780	0.992
Cross-sectional	THREOCAT.PWY	-0.005	0.036	0.891	0.998
Cross-sectional	THRESYN.PWY	0.002	0.007	0.785	0.992
Cross-sectional	TRNA.CHARGING.PWY	0.002	0.003	0.574	0.992
Cross-sectional	TRPSYN.PWY	-0.006	0.010	0.580	0.992
Cross-sectional	UDPNAGSYN.PWY	0.017	0.013	0.212	0.992
Cross-sectional	VALSYN.PWY	-0.007	0.005	0.159	0.992
Cross-sectional	PWY.5101	-0.002	0.090	0.980	0.998
Cross-sectional	PWY.5304	0.001	0.030	0.980	0.998
Cross-sectional	PWY.6167	-0.002	0.072	0.980	0.998
Cross-sectional	PWY.7117	-0.001	0.027	0.979	0.998
Cross-sectional	PROPFERM.PWY	0.000	0.025	0.998	0.998
Cross-sectional	PWY.5265	-0.001	0.050	0.990	0.998
Cross-sectional	PWY.5845	-0.001	0.060	0.988	0.998
Cross-sectional	PWY.5862	0.000	0.061	0.994	0.998
Cross-sectional	PWY.6837	0.000	0.028	0.991	0.998
Cross-sectional	PWY.7242	0.000	0.039	0.996	0.998
Cross-sectional	PWY.724	0.000	0.004	0.995	0.998
Meconium Adj.[b]	P164.PWY	0.063	0.024	0.011	0.963
Meconium Adj.[b]	PWY0.1415	-0.142	0.057	0.016	0.963
Meconium Adj.[b]	PWY.6285	-0.069	0.031	0.030	0.963
Meconium Adj.[b]	PWY.6936	0.024	0.011	0.030	0.963
Meconium Adj.[b]	P562.PWY	-0.073	0.034	0.037	0.963
Meconium Adj.[b]	PWY.7385	-0.115	0.056	0.046	0.963
Meconium Adj.[b]	FAO.PWY	0.103	0.053	0.059	0.963
Meconium Adj.[b]	PWY.5136	0.109	0.055	0.054	0.963
Meconium Adj.[b]	PWY66.398	-0.125	0.062	0.052	0.963
Meconium Adj.[b]	PWY.7094	-0.047	0.025	0.065	0.963
Meconium Adj.[b]	HSERMETANA.PWY	0.019	0.010	0.075	0.963
Meconium Adj.[b]	OANTIGEN.PWY	0.018	0.010	0.085	0.963

Exposure Window	MetaCyc Pathway ID	Effect Estimate[a]	Std. Error	p-value	q-value
Meconium Adj.[b]	PWY.6471	0.044	0.025	0.084	0.963
Meconium Adj.[b]	PWY.6588	0.128	0.071	0.077	0.963
Meconium Adj.[b]	PWY.7003	0.115	0.063	0.076	0.963
Meconium Adj.[b]	PWY0.1261	-0.102	0.058	0.088	0.963
Meconium Adj.[b]	NAGLIPASYN.PWY	-0.117	0.070	0.104	0.963
Meconium Adj.[b]	PWY.1269	-0.081	0.049	0.107	0.963
Meconium Adj.[b]	PWY.3781	-0.123	0.072	0.094	0.963
Meconium Adj.[b]	PWY.5004	-0.065	0.038	0.094	0.963
Meconium Adj.[b]	PWY.7312	-0.075	0.045	0.102	0.963
Meconium Adj.[b]	HEXITOLDEGSUPER.PWY	-0.021	0.013	0.119	0.963
Meconium Adj.[b]	KETOGLUCONMET.PWY	-0.167	0.106	0.124	0.963
Meconium Adj.[b]	PWY.5656	-0.070	0.045	0.122	0.963
Meconium Adj.[b]	PWY0.1241	-0.135	0.084	0.115	0.963
Meconium Adj.[b]	HEME.BIOSYNTHESIS.II	-0.118	0.077	0.131	0.963
Meconium Adj.[b]	PWY.6837	-0.039	0.026	0.130	0.963
Meconium Adj.[b]	P461.PWY	-0.036	0.024	0.136	0.963
Meconium Adj.[b]	P161.PWY	0.132	0.087	0.137	0.963
Meconium Adj.[b]	PWY.5677	0.070	0.046	0.138	0.963
Meconium Adj.[b]	PWY.5863	-0.104	0.070	0.143	0.963
Meconium Adj.[b]	PWY4LZ.257	0.130	0.086	0.139	0.963
Meconium Adj.[b]	PWY66.409	0.031	0.021	0.146	0.963
Meconium Adj.[b]	ARGININE.SYN4.PWY	-0.105	0.080	0.198	0.963
Meconium Adj.[b]	HEMESYN2.PWY	-0.052	0.040	0.194	0.963
Meconium Adj.[b]	PWY.4722	-0.088	0.065	0.182	0.963
Meconium Adj.[b]	PWY.5030	-0.069	0.052	0.193	0.963
Meconium Adj.[b]	PWY.5392	-0.054	0.039	0.176	0.963
Meconium Adj.[b]	PWY.5464	-0.102	0.075	0.185	0.963
Meconium Adj.[b]	PWY.5897	-0.089	0.068	0.198	0.963
Meconium Adj.[b]	PWY.5898	-0.089	0.068	0.198	0.963
Meconium Adj.[b]	PWY.5899	-0.089	0.068	0.198	0.963
Meconium Adj.[b]	PWY.5918	-0.088	0.068	0.202	0.963
Meconium Adj.[b]	PWY.6549	0.024	0.017	0.183	0.963
Meconium Adj.[b]	PWY.6895	-0.083	0.059	0.163	0.963
Meconium Adj.[b]	PWY66.422	0.012	0.009	0.189	0.963
Meconium Adj.[b]	PWY.5505	-0.096	0.075	0.204	0.963
Meconium Adj.[b]	LACTOSECAT.PWY	0.049	0.038	0.207	0.963
Meconium Adj.[b]	PWY.4702	-0.085	0.066	0.208	0.963

Exposure Window	MetaCyc Pathway ID	Effect Estimate[a]	Std. Error	p-value	q-value
Meconium Adj.[b]	PWY.5791	-0.090	0.071	0.211	0.963
Meconium Adj.[b]	PWY.5837	-0.090	0.071	0.211	0.963
Meconium Adj.[b]	PWY.7221	0.006	0.005	0.214	0.963
Meconium Adj.[b]	DTDPRHAMSYN.PWY	0.012	0.010	0.255	0.963
Meconium Adj.[b]	FOLSYN.PWY	0.050	0.042	0.239	0.963
Meconium Adj.[b]	HISDEG.PWY	-0.052	0.046	0.259	0.963
Meconium Adj.[b]	PWY.4981	0.037	0.032	0.250	0.963
Meconium Adj.[b]	PWY.5971	-0.060	0.051	0.243	0.963
Meconium Adj.[b]	PWY.5994	-0.098	0.080	0.230	0.963
Meconium Adj.[b]	PWY.6612	0.050	0.042	0.243	0.963
Meconium Adj.[b]	PWY.7196	0.017	0.014	0.235	0.963
Meconium Adj.[b]	PWY.7210	-0.108	0.088	0.228	0.963
Meconium Adj.[b]	PWY.7211	0.024	0.020	0.254	0.963
Meconium Adj.[b]	UDPNAGSYN.PWY	0.018	0.015	0.247	0.963
Meconium Adj.[b]	SER.GLYSYN.PWY	0.009	0.008	0.260	0.963
Meconium Adj.[b]	GLYCOGENSYNTH.PWY	0.013	0.011	0.261	0.963
Meconium Adj.[b]	PWY.5690	-0.025	0.023	0.268	0.963
Meconium Adj.[b]	PWY0.42	-0.041	0.037	0.266	0.963
Meconium Adj.[b]	GLUDEG.I.PWY	-0.066	0.060	0.277	0.963
Meconium Adj.[b]	METH.ACETATE.PWY	0.025	0.023	0.278	0.963
Meconium Adj.[b]	PWY.7539	-0.047	0.043	0.279	0.963
Meconium Adj.[b]	PWY.7371	-0.098	0.089	0.280	0.963
Meconium Adj.[b]	PWY.7332	-0.081	0.075	0.287	0.963
Meconium Adj.[b]	ARO.PWY	0.007	0.007	0.295	0.963
Meconium Adj.[b]	COBALSYN.PWY	0.015	0.014	0.304	0.963
Meconium Adj.[b]	METHGLYUT.PWY	-0.074	0.072	0.307	0.963
Meconium Adj.[b]	P441.PWY	0.033	0.032	0.303	0.963
Meconium Adj.[b]	PWY.5100	0.012	0.012	0.302	0.963
Meconium Adj.[b]	PWY.5747	-0.074	0.072	0.307	0.963
Meconium Adj.[b]	PWY.5838	-0.078	0.073	0.294	0.963
Meconium Adj.[b]	PWY.5861	-0.079	0.075	0.298	0.963
Meconium Adj.[b]	PWY.6147	-0.046	0.043	0.288	0.963
Meconium Adj.[b]	PWY.6163	0.008	0.007	0.304	0.963
Meconium Adj.[b]	PWY.7187	0.020	0.019	0.308	0.963
Meconium Adj.[b]	PWY.7208	-0.019	0.018	0.291	0.963
Meconium Adj.[b]	PWY490.3	0.047	0.044	0.291	0.963
Meconium Adj.[b]	PYRIDOXSYN.PWY	-0.082	0.079	0.307	0.963

Exposure Window	MetaCyc Pathway ID	Effect Estimate[a]	Std. Error	p-value	q-value
Meconium Adj.[b]	TCA	-0.024	0.023	0.292	0.963
Meconium Adj.[b]	CRNFORCAT.PWY	0.047	0.047	0.329	0.963
Meconium Adj.[b]	FERMENTATION.PWY	0.018	0.018	0.325	0.963
Meconium Adj.[b]	PWY.7456	0.030	0.029	0.316	0.963
Meconium Adj.[b]	PWY0.1297	0.030	0.030	0.315	0.963
Meconium Adj.[b]	PWY.6122	0.006	0.006	0.331	0.963
Meconium Adj.[b]	PWY.6277	0.006	0.006	0.331	0.963
Meconium Adj.[b]	PWY.821	-0.079	0.081	0.335	0.963
Meconium Adj.[b]	DENOVOPURINE2.PWY	0.016	0.017	0.351	0.963
Meconium Adj.[b]	PRPP.PWY	0.032	0.034	0.363	0.963
Meconium Adj.[b]	PWY.5920	-0.044	0.047	0.357	0.963
Meconium Adj.[b]	PWY.6125	0.020	0.021	0.350	0.963
Meconium Adj.[b]	PWY.621	-0.020	0.021	0.353	0.963
Meconium Adj.[b]	PWY.7328	-0.053	0.057	0.365	0.963
Meconium Adj.[b]	PWY.7357	0.010	0.011	0.361	0.963
Meconium Adj.[b]	PWY.841	0.018	0.018	0.340	0.963
Meconium Adj.[b]	PWY0.166	0.017	0.019	0.356	0.963
Meconium Adj.[b]	PWY.7616	-0.096	0.105	0.369	0.963
Meconium Adj.[b]	COMPLETE.ARO.PWY	0.006	0.007	0.431	0.963
Meconium Adj.[b]	GLUCONEO.PWY	-0.009	0.010	0.405	0.963
Meconium Adj.[b]	GLUCOSE1PMETAB.PWY	-0.052	0.063	0.415	0.963
Meconium Adj.[b]	GLYCOCAT.PWY	-0.048	0.060	0.432	0.963
Meconium Adj.[b]	HISTSYN.PWY	0.006	0.007	0.389	0.963
Meconium Adj.[b]	POLYISOPRENSYN.PWY	-0.050	0.062	0.428	0.963
Meconium Adj.[b]	PWY.2723	-0.055	0.064	0.389	0.963
Meconium Adj.[b]	PWY.5005	-0.078	0.090	0.393	0.963
Meconium Adj.[b]	PWY.5675	-0.058	0.071	0.416	0.963
Meconium Adj.[b]	PWY.5695	0.007	0.007	0.379	0.963
Meconium Adj.[b]	PWY.6113	-0.038	0.047	0.426	0.963
Meconium Adj.[b]	PWY.6121	0.006	0.007	0.382	0.963
Meconium Adj.[b]	PWY.6167	-0.044	0.054	0.416	0.963
Meconium Adj.[b]	PWY.6349	-0.049	0.062	0.430	0.963
Meconium Adj.[b]	PWY.6545	0.018	0.023	0.428	0.963
Meconium Adj.[b]	PWY.6608	0.016	0.019	0.401	0.963
Meconium Adj.[b]	PWY.6731	-0.041	0.052	0.429	0.963
Meconium Adj.[b]	PWY.7013	-0.023	0.028	0.412	0.963
Meconium Adj.[b]	PWY.7184	0.020	0.024	0.402	0.963

Exposure Window	MetaCyc Pathway ID	Effect Estimate[a]	Std. Error	p-value	q-value
Meconium Adj.[b]	PWY.7197	0.024	0.027	0.385	0.963
Meconium Adj.[b]	PWY.7198	0.022	0.026	0.393	0.963
Meconium Adj.[b]	PWY.7228	0.020	0.023	0.384	0.963
Meconium Adj.[b]	PWY66.391	0.048	0.059	0.423	0.963
Meconium Adj.[b]	PYRIDNUCSAL.PWY	0.047	0.057	0.415	0.963
Meconium Adj.[b]	RHAMCAT.PWY	-0.018	0.020	0.396	0.963
Meconium Adj.[b]	PHOSLIPSYN.PWY	0.026	0.033	0.440	0.963
Meconium Adj.[b]	PWY66.399	-0.012	0.016	0.443	0.963
Meconium Adj.[b]	PWY.6168	-0.010	0.013	0.444	0.963
Meconium Adj.[b]	PWY.6396	-0.032	0.042	0.446	0.963
Meconium Adj.[b]	ARGORNPROST.PWY	-0.037	0.049	0.450	0.963
Meconium Adj.[b]	METHANOGENESIS.PWY	-0.045	0.060	0.451	0.963
Meconium Adj.[b]	PWY.7286	-0.050	0.066	0.451	0.963
Meconium Adj.[b]	PWY4FS.7	0.028	0.036	0.448	0.963
Meconium Adj.[b]	PWY4FS.8	0.028	0.036	0.448	0.963
Meconium Adj.[b]	ARGSYNBSUB.PWY	0.005	0.007	0.453	0.963
Meconium Adj.[b]	PWY0.845	-0.059	0.078	0.453	0.963
Meconium Adj.[b]	PWY.5973	-0.035	0.048	0.462	0.963
Meconium Adj.[b]	ANAGLYCOLYSIS.PWY	-0.005	0.009	0.587	0.963
Meconium Adj.[b]	ARG.POLYAMINE.SYN	0.021	0.040	0.605	0.963
Meconium Adj.[b]	ARGSYN.PWY	0.004	0.007	0.523	0.963
Meconium Adj.[b]	BIOTIN.BIOSYNTHESIS.PWY	-0.049	0.082	0.549	0.963
Meconium Adj.[b]	BRANCHED.CHAIN.AA.SYN.PWY	0.005	0.008	0.535	0.963
Meconium Adj.[b]	CALVIN.PWY	0.006	0.009	0.484	0.963
Meconium Adj.[b]	COA.PWY	0.004	0.007	0.573	0.963
Meconium Adj.[b]	FASYN.ELONG.PWY	-0.037	0.054	0.499	0.963
Meconium Adj.[b]	GLUTORN.PWY	0.006	0.009	0.476	0.963
Meconium Adj.[b]	GLYOXYLATE.BYPASS	-0.049	0.079	0.543	0.963
Meconium Adj.[b]	HCAMHPDEG.PWY	-0.018	0.033	0.576	0.963
Meconium Adj.[b]	NONOXIPENT.PWY	0.007	0.013	0.596	0.963
Meconium Adj.[b]	P124.PWY	0.037	0.061	0.555	0.963
Meconium Adj.[b]	P185.PWY	-0.016	0.029	0.580	0.963
Meconium Adj.[b]	P23.PWY	-0.067	0.092	0.473	0.963
Meconium Adj.[b]	PENTOSE.P.PWY	0.033	0.046	0.478	0.963
Meconium Adj.[b]	PEPTIDOGLYCANSYN.PWY	0.003	0.005	0.492	0.963
Meconium Adj.[b]	PPGPPMET.PWY	0.015	0.023	0.529	0.963
Meconium Adj.[b]	PWY.4242	0.005	0.008	0.535	0.963

Exposure Window	MetaCyc Pathway ID	Effect Estimate[a]	Std. Error	p-value	q-value
Meconium Adj.[b]	PWY.5022	-0.037	0.055	0.498	0.963
Meconium Adj.[b]	PWY.5083	-0.050	0.083	0.549	0.963
Meconium Adj.[b]	PWY.5097	0.003	0.005	0.623	0.963
Meconium Adj.[b]	PWY.5103	0.006	0.008	0.507	0.963
Meconium Adj.[b]	PWY.5154	-0.023	0.036	0.522	0.963
Meconium Adj.[b]	PWY.5177	-0.013	0.020	0.514	0.963
Meconium Adj.[b]	PWY.5189	0.041	0.072	0.569	0.963
Meconium Adj.[b]	PWY.5198	-0.035	0.055	0.525	0.963
Meconium Adj.[b]	PWY.5265	-0.036	0.065	0.577	0.963
Meconium Adj.[b]	PWY.5304	-0.021	0.041	0.619	0.963
Meconium Adj.[b]	PWY.5367	0.054	0.077	0.482	0.963
Meconium Adj.[b]	PWY.5676	-0.013	0.023	0.581	0.963
Meconium Adj.[b]	PWY.5686	0.003	0.005	0.500	0.963
Meconium Adj.[b]	PWY.5840	-0.041	0.065	0.536	0.963
Meconium Adj.[b]	PWY.5845	-0.039	0.075	0.610	0.963
Meconium Adj.[b]	PWY.5862	-0.040	0.076	0.605	0.963
Meconium Adj.[b]	PWY.6123	0.004	0.008	0.618	0.963
Meconium Adj.[b]	PWY.6282	-0.031	0.059	0.599	0.963
Meconium Adj.[b]	PWY.6305	0.014	0.020	0.490	0.963
Meconium Adj.[b]	PWY.6317	0.007	0.014	0.627	0.963
Meconium Adj.[b]	PWY.6353	0.008	0.016	0.607	0.963
Meconium Adj.[b]	PWY.6385	0.003	0.005	0.591	0.963
Meconium Adj.[b]	PWY.6386	0.003	0.005	0.502	0.963
Meconium Adj.[b]	PWY.6387	0.002	0.004	0.593	0.963
Meconium Adj.[b]	PWY.6435	0.043	0.089	0.627	0.963
Meconium Adj.[b]	PWY.6519	-0.051	0.084	0.547	0.963
Meconium Adj.[b]	PWY.6531	-0.047	0.084	0.579	0.963
Meconium Adj.[b]	PWY.6572	0.021	0.032	0.527	0.963
Meconium Adj.[b]	PWY.6606	0.012	0.018	0.514	0.963
Meconium Adj.[b]	PWY.6628	0.041	0.079	0.610	0.963
Meconium Adj.[b]	PWY.6690	-0.018	0.033	0.576	0.963
Meconium Adj.[b]	PWY.6823	0.018	0.029	0.547	0.963
Meconium Adj.[b]	PWY.6859	-0.048	0.088	0.584	0.963
Meconium Adj.[b]	PWY.6891	0.031	0.048	0.512	0.963
Meconium Adj.[b]	PWY.6897	0.011	0.019	0.564	0.963
Meconium Adj.[b]	PWY.6901	0.024	0.039	0.536	0.963
Meconium Adj.[b]	PWY.7115	-0.023	0.034	0.508	0.963

Exposure Window	MetaCyc Pathway ID	Effect Estimate[a]	Std. Error	p-value	q-value
Meconium Adj.[b]	PWY.7204	-0.024	0.039	0.541	0.963
Meconium Adj.[b]	PWY.7209	0.058	0.083	0.493	0.963
Meconium Adj.[b]	PWY.7219	0.004	0.007	0.576	0.963
Meconium Adj.[b]	PWY.7220	-0.010	0.020	0.611	0.963
Meconium Adj.[b]	PWY.7222	-0.010	0.020	0.611	0.963
Meconium Adj.[b]	PWY.7229	0.004	0.007	0.591	0.963
Meconium Adj.[b]	PWY.7234	0.019	0.037	0.614	0.963
Meconium Adj.[b]	PWY.724	0.003	0.004	0.508	0.963
Meconium Adj.[b]	PWY.7288	0.041	0.060	0.494	0.963
Meconium Adj.[b]	PWY.7316	0.032	0.064	0.619	0.963
Meconium Adj.[b]	PWY.7323	0.016	0.030	0.595	0.963
Meconium Adj.[b]	PWY.7392	-0.045	0.081	0.577	0.963
Meconium Adj.[b]	PWY.7400	0.004	0.007	0.521	0.963
Meconium Adj.[b]	PWY.7663	-0.030	0.051	0.566	0.963
Meconium Adj.[b]	PWY.7664	-0.038	0.059	0.523	0.963
Meconium Adj.[b]	PWY0.1277	-0.013	0.025	0.602	0.963
Meconium Adj.[b]	PWY0.862	-0.039	0.061	0.521	0.963
Meconium Adj.[b]	PWY0.881	-0.044	0.068	0.519	0.963
Meconium Adj.[b]	PWY66.400	0.010	0.021	0.615	0.963
Meconium Adj.[b]	PWYG.321	-0.032	0.060	0.597	0.963
Meconium Adj.[b]	RIBOSYN2.PWY	-0.007	0.012	0.571	0.963
Meconium Adj.[b]	RUMP.PWY	0.057	0.088	0.518	0.963
Meconium Adj.[b]	THISYN.PWY	-0.020	0.032	0.539	0.963
Meconium Adj.[b]	THREOCAT.PWY	0.020	0.033	0.539	0.963
Meconium Adj.[b]	PWY.6507	-0.022	0.045	0.631	0.963
Meconium Adj.[b]	PWY.7315	0.027	0.056	0.632	0.963
Meconium Adj.[b]	X7ALPHADEHYDROX.PWY	-0.021	0.065	0.747	0.963
Meconium Adj.[b]	ALLANTOINDEG.PWY	-0.012	0.044	0.790	0.963
Meconium Adj.[b]	ASPASN.PWY	0.005	0.012	0.663	0.963
Meconium Adj.[b]	CITRULBIO.PWY	-0.019	0.052	0.723	0.963
Meconium Adj.[b]	FUC.RHAMCAT.PWY	-0.022	0.048	0.649	0.963
Meconium Adj.[b]	FUCCAT.PWY	-0.016	0.037	0.660	0.963
Meconium Adj.[b]	GALACT.GLUCUROCAT.PWY	0.018	0.052	0.731	0.963
Meconium Adj.[b]	GALACTARDEG.PWY	0.038	0.088	0.672	0.963
Meconium Adj.[b]	GALACTUROCAT.PWY	-0.019	0.042	0.662	0.963
Meconium Adj.[b]	GLUCARDEG.PWY	0.028	0.082	0.736	0.963
Meconium Adj.[b]	GLUCARGALACTSUPER.PWY	0.038	0.088	0.672	0.963

Exposure Window	MetaCyc Pathway ID	Effect Estimate[a]	Std. Error	p-value	q-value
Meconium Adj.[b]	GLYCOLYSIS.E.D	-0.018	0.060	0.765	0.963
Meconium Adj.[b]	GLYCOLYSIS.TCA.GLYOX.BYPASS	-0.027	0.065	0.678	0.963
Meconium Adj.[b]	GOLPDLCAT.PWY	0.009	0.025	0.713	0.963
Meconium Adj.[b]	MET.SAM.PWY	0.010	0.021	0.646	0.963
Meconium Adj.[b]	METSYN.PWY	0.007	0.022	0.747	0.963
Meconium Adj.[b]	NAD.BIOSYNTHESIS.II	-0.049	0.108	0.653	0.963
Meconium Adj.[b]	ORNDEG.PWY	-0.009	0.033	0.778	0.963
Meconium Adj.[b]	P105.PWY	-0.019	0.066	0.779	0.963
Meconium Adj.[b]	P108.PWY	-0.033	0.105	0.756	0.963
Meconium Adj.[b]	P122.PWY	-0.043	0.114	0.709	0.963
Meconium Adj.[b]	P125.PWY	-0.044	0.108	0.684	0.963
Meconium Adj.[b]	P162.PWY	0.018	0.040	0.663	0.963
Meconium Adj.[b]	P221.PWY	-0.019	0.071	0.787	0.963
Meconium Adj.[b]	P42.PWY	0.022	0.049	0.656	0.963
Meconium Adj.[b]	PANTOSYN.PWY	0.003	0.009	0.779	0.963
Meconium Adj.[b]	POLYAMINSYN3.PWY	-0.031	0.073	0.677	0.963
Meconium Adj.[b]	POLYAMSYN.PWY	0.020	0.042	0.645	0.963
Meconium Adj.[b]	PROPFERM.PWY	0.009	0.024	0.709	0.963
Meconium Adj.[b]	PWY.1861	-0.011	0.029	0.693	0.963
Meconium Adj.[b]	PWY.3001	0.003	0.007	0.635	0.963
Meconium Adj.[b]	PWY.3841	-0.002	0.004	0.637	0.963
Meconium Adj.[b]	PWY.4041	-0.009	0.020	0.652	0.963
Meconium Adj.[b]	PWY.4984	-0.020	0.053	0.706	0.963
Meconium Adj.[b]	PWY.5101	-0.056	0.127	0.664	0.963
Meconium Adj.[b]	PWY.5104	-0.008	0.021	0.716	0.963
Meconium Adj.[b]	PWY.5121	-0.014	0.034	0.690	0.963
Meconium Adj.[b]	PWY.5138	0.020	0.076	0.789	0.963
Meconium Adj.[b]	PWY.5173	0.029	0.095	0.760	0.963
Meconium Adj.[b]	PWY.5188	-0.008	0.018	0.668	0.963
Meconium Adj.[b]	PWY.5384	-0.014	0.043	0.737	0.963
Meconium Adj.[b]	PWY.561	-0.030	0.075	0.689	0.963
Meconium Adj.[b]	PWY.5723	-0.022	0.057	0.706	0.963
Meconium Adj.[b]	PWY.5850	0.010	0.038	0.784	0.963
Meconium Adj.[b]	PWY.5860	0.010	0.038	0.790	0.963
Meconium Adj.[b]	PWY.5896	0.010	0.038	0.784	0.963
Meconium Adj.[b]	PWY.5913	0.010	0.032	0.747	0.963
Meconium Adj.[b]	PWY.6124	0.004	0.008	0.647	0.963

Exposure Window	MetaCyc Pathway ID	Effect Estimate[a]	Std. Error	p-value	q-value
Meconium Adj.[b]	PWY.6151	0.003	0.008	0.675	0.963
Meconium Adj.[b]	PWY.622	-0.021	0.059	0.731	0.963
Meconium Adj.[b]	PWY.6263	-0.026	0.070	0.708	0.963
Meconium Adj.[b]	PWY.6527	0.006	0.016	0.713	0.963
Meconium Adj.[b]	PWY.6609	0.003	0.009	0.734	0.963
Meconium Adj.[b]	PWY.6630	-0.020	0.054	0.710	0.963
Meconium Adj.[b]	PWY.6700	0.004	0.008	0.649	0.963
Meconium Adj.[b]	PWY.6703	-0.007	0.014	0.646	0.963
Meconium Adj.[b]	PWY.6803	0.017	0.052	0.740	0.963
Meconium Adj.[b]	PWY.6892	0.006	0.017	0.718	0.963
Meconium Adj.[b]	PWY.7111	0.002	0.008	0.765	0.963
Meconium Adj.[b]	PWY.7199	0.005	0.011	0.683	0.963
Meconium Adj.[b]	PWY.7237	-0.007	0.019	0.703	0.963
Meconium Adj.[b]	PWY.7282	-0.030	0.069	0.666	0.963
Meconium Adj.[b]	PWY0.1061	-0.019	0.043	0.663	0.963
Meconium Adj.[b]	PWY0.1296	0.005	0.012	0.709	0.963
Meconium Adj.[b]	PWY0.1298	-0.018	0.039	0.643	0.963
Meconium Adj.[b]	PWY0.162	-0.005	0.014	0.743	0.963
Meconium Adj.[b]	PYRIDNUCSYN.PWY	0.002	0.008	0.769	0.963
Meconium Adj.[b]	REDCITCYC	-0.020	0.073	0.784	0.963
Meconium Adj.[b]	SALVADEHYPOX.PWY	0.006	0.019	0.742	0.963
Meconium Adj.[b]	TCA.GLYOX.BYPASS	-0.027	0.068	0.688	0.963
Meconium Adj.[b]	TEICHOICACID.PWY	0.008	0.019	0.685	0.963
Meconium Adj.[b]	THISYNARA.PWY	0.008	0.019	0.687	0.963
Meconium Adj.[b]	THRESYN.PWY	0.002	0.008	0.761	0.963
Meconium Adj.[b]	TRNA.CHARGING.PWY	0.001	0.005	0.762	0.963
Meconium Adj.[b]	X1CMET2.PWY	0.001	0.005	0.799	0.963
Meconium Adj.[b]	HOMOSER.METSYN.PWY	0.007	0.026	0.797	0.963
Meconium Adj.[b]	NONMEVIPP.PWY	0.009	0.033	0.797	0.963
Meconium Adj.[b]	PWY.5941	-0.026	0.104	0.801	0.963
Meconium Adj.[b]	PWY.6318	0.009	0.036	0.804	0.963
Meconium Adj.[b]	ILEUSYN.PWY	0.002	0.007	0.810	0.963
Meconium Adj.[b]	PWY.2941	0.007	0.030	0.808	0.963
Meconium Adj.[b]	VALSYN.PWY	0.002	0.007	0.810	0.963
Meconium Adj.[b]	UNMAPPED	-0.002	0.007	0.812	0.963
Meconium Adj.[b]	PWY.7388	-0.016	0.067	0.815	0.963
Meconium Adj.[b]	FASYN.INITIAL.PWY	-0.015	0.066	0.819	0.965

Exposure Window	MetaCyc Pathway ID	Effect Estimate[a]	Std. Error	p-value	q-value
Meconium Adj.[b]	PWY.7254	-0.014	0.068	0.837	0.975
Meconium Adj.[b]	PWY.1042	0.001	0.007	0.843	0.975
Meconium Adj.[b]	PWY.6470	0.006	0.033	0.847	0.975
Meconium Adj.[b]	GLYCOL.GLYOXDEG.PWY	0.011	0.061	0.855	0.975
Meconium Adj.[b]	P621.PWY	-0.013	0.070	0.857	0.975
Meconium Adj.[b]	PWY.6749	0.010	0.054	0.855	0.975
Meconium Adj.[b]	PWY.7117	0.006	0.031	0.850	0.975
Meconium Adj.[b]	PWY0.1586	-0.004	0.022	0.856	0.975
Meconium Adj.[b]	COA.PWY.1	0.001	0.004	0.864	0.975
Meconium Adj.[b]	P4.PWY	0.006	0.034	0.864	0.975
Meconium Adj.[b]	PWY.5347	0.003	0.019	0.861	0.975
Meconium Adj.[b]	PWY66.389	-0.018	0.101	0.860	0.975
Meconium Adj.[b]	PWY.6876	0.010	0.059	0.866	0.975
Meconium Adj.[b]	UNINTEGRATED	0.000	0.002	0.893	0.975
Meconium Adj.[b]	DAPLYSINESYN.PWY	0.003	0.025	0.888	0.975
Meconium Adj.[b]	ENTBACSYN.PWY	-0.005	0.030	0.878	0.975
Meconium Adj.[b]	GLYCOLYSIS	0.002	0.018	0.912	0.976
Meconium Adj.[b]	PANTO.PWY	0.001	0.011	0.905	0.975
Meconium Adj.[b]	PWY.241	0.005	0.027	0.868	0.975
Meconium Adj.[b]	PWY.5910	0.005	0.049	0.914	0.976
Meconium Adj.[b]	PWY.6126	0.001	0.010	0.905	0.975
Meconium Adj.[b]	PWY.6270	0.007	0.045	0.879	0.975
Meconium Adj.[b]	PWY.6284	-0.007	0.052	0.891	0.975
Meconium Adj.[b]	PWY.6478	0.006	0.047	0.905	0.975
Meconium Adj.[b]	PWY.6629	0.008	0.054	0.882	0.975
Meconium Adj.[b]	PWY.6737	0.001	0.008	0.897	0.975
Meconium Adj.[b]	PWY.6969	-0.004	0.029	0.886	0.975
Meconium Adj.[b]	PWY.7046	0.012	0.072	0.872	0.975
Meconium Adj.[b]	PWY.7560	0.007	0.046	0.883	0.975
Meconium Adj.[b]	PWY0.1479	-0.003	0.023	0.904	0.975
Meconium Adj.[b]	PWY3O.355	0.013	0.082	0.874	0.975
Meconium Adj.[b]	ARGDEG.PWY	0.006	0.056	0.921	0.976
Meconium Adj.[b]	ORNARGDEG.PWY	0.006	0.056	0.921	0.976
Meconium Adj.[b]	PWY.5989	-0.006	0.057	0.923	0.976
Meconium Adj.[b]	PWY0.781	0.003	0.031	0.922	0.976
Meconium Adj.[b]	SO4ASSIM.PWY	-0.007	0.073	0.927	0.977
Meconium Adj.[b]	TRPSYN.PWY	-0.001	0.014	0.929	0.977

Exposure Window	MetaCyc Pathway ID	Effect Estimate[a]	Std. Error	p-value	q-value
Meconium Adj.[b]	PWY.922	0.005	0.057	0.937	0.983
Meconium Adj.[b]	PWY.2942	0.000	0.005	0.950	0.988
Meconium Adj.[b]	CENTFERM.PWY	-0.002	0.041	0.964	0.988
Meconium Adj.[b]	COLANSYN.PWY	0.001	0.016	0.963	0.988
Meconium Adj.[b]	PWY.5088	0.001	0.022	0.952	0.988
Meconium Adj.[b]	PWY.5484	-0.001	0.021	0.958	0.988
Meconium Adj.[b]	PWY.6595	-0.005	0.090	0.956	0.988
Meconium Adj.[b]	PWY.7242	-0.003	0.045	0.953	0.988
Meconium Adj.[b]	PWY.7383	-0.001	0.020	0.955	0.988
Meconium Adj.[b]	GLUCUROCAT.PWY	-0.002	0.041	0.968	0.990
Meconium Adj.[b]	PWY.5345	-0.001	0.069	0.983	0.993
Meconium Adj.[b]	PWY.5667	0.000	0.010	0.985	0.993
Meconium Adj.[b]	PWY.6590	-0.001	0.041	0.985	0.993
Meconium Adj.[b]	PWY0.1319	0.000	0.010	0.985	0.993
Meconium Adj.[b]	SULFATE.CYS.PWY	-0.002	0.070	0.979	0.993
Meconium Adj.[b]	ANAEROFRUCAT.PWY	0.000	0.015	0.990	0.995
Meconium Adj.[b]	GLCMANNANAUT.PWY	0.000	0.023	0.994	0.997
Meconium Adj.[b]	PWY.5659	0.000	0.023	0.999	0.999

[a]Models are adjusted for whether the child was ever breastfed, sex, mode of birth, and socioeconomic status
[b]Meconium Adj. includes cross-sectional exposure in the model

Table S7. Associations of acetaminophen and caffeine with beta diversity

Exposure	Exposure Window	Method	p-value[a]	q-value	R2
Caffeine	Meconium	Weighted UniFrac	0.713	0.990	0.014
Caffeine	Meconium	Bray-Curtis	0.512	0.990	0.020
Caffeine	Meconium	Unweighted UniFrac	0.982	0.990	0.012
Caffeine	Meconium	Jaccard	0.930	0.990	0.015
Acetaminophen	Meconium	Weighted UniFrac	0.317	0.990	0.024
Acetaminophen	Meconium	Bray-Curtis	0.358	0.990	0.023
Acetaminophen	Meconium	Unweighted UniFrac	0.928	0.990	0.014
Acetaminophen	Meconium	Jaccard	0.990	0.990	0.013
Caffeine	Cross-sectional	Weighted UniFrac	0.333	0.593	0.013
Caffeine	Cross-sectional	Bray-Curtis	0.445	0.593	0.012
Caffeine	Cross-sectional	Unweighted UniFrac	0.288	0.593	0.013
Caffeine	Cross-sectional	Jaccard	0.519	0.593	0.011
Acetaminophen	Cross-sectional	Weighted UniFrac	0.480	0.593	0.012
Acetaminophen	Cross-sectional	Bray-Curtis	0.818	0.818	0.008
Acetaminophen	Cross-sectional	Unweighted UniFrac	0.100	0.520	0.016
Acetaminophen	Cross-sectional	Jaccard	0.130	0.520	0.015
Caffeine	Meconium Adj.[b]	Weighted UniFrac	0.389	0.990	0.022
Caffeine	Meconium Adj.[b]	Bray-Curtis	0.517	0.990	0.020
Caffeine	Meconium Adj.[b]	Unweighted UniFrac	0.799	0.990	0.017
Caffeine	Meconium Adj.[b]	Jaccard	0.931	0.990	0.015
Acetaminophen	Meconium Adj.[b]	Weighted UniFrac	0.400	0.990	0.022
Acetaminophen	Meconium Adj.[b]	Bray-Curtis	0.338	0.990	0.023
Acetaminophen	Meconium Adj.[b]	Unweighted UniFrac	0.924	0.990	0.014
Acetaminophen	Meconium Adj.[b]	Jaccard	0.990	0.990	0.013

[a]Models are adjusted for whether the child was ever breastfed, sex, mode of birth, and socioeconomic status
[b]Meconium Adj. includes cross-sectional exposure in the model

Table S8. Acetaminophen-phylum interactions in association with neurodevelopmental outcomes

Exposure Window	Phylum	Outcome	Variable	Effect Estimate[a]	Std. Error	LRT[b]	
						p-value	q-value
Meconium	Actinobacteria	Block Design	Acetaminophen	0.866	1.969	0.968	0.968
Meconium	Actinobacteria	Block Design	Phylum	0.003	0.035		
Meconium	Actinobacteria	Block Design	Interaction	0.002	0.059		
Meconium	Actinobacteria	Coding	Acetaminophen	-0.789	1.714	0.456	0.95
Meconium	Actinobacteria	Coding	Phylum	-0.029	0.03		
Meconium	Actinobacteria	Coding	Interaction	0.035	0.051		
Meconium	Actinobacteria	Digit span	Acetaminophen	-0.244	1.482	0.898	0.952
Meconium	Actinobacteria	Digit span	Phylum	-0.028	0.026		
Meconium	Actinobacteria	Digit span	Interaction	-0.005	0.044		
Meconium	Actinobacteria	Information	Acetaminophen	-0.424	1.471	0.877	0.952
Meconium	Actinobacteria	Information	Phylum	-0.023	0.026		
Meconium	Actinobacteria	Information	Interaction	0.006	0.044		
Meconium	Actinobacteria	QTAC	Acetaminophen	2.405	5.099	0.189	0.736
Meconium	Actinobacteria	QTAC	Phylum	0.123	0.09		
Meconium	Actinobacteria	QTAC	Interaction	-0.185	0.153		
Meconium	Actinobacteria	Vocabulary	Acetaminophen	-0.121	2.233	0.838	0.952
Meconium	Actinobacteria	Vocabulary	Phylum	-0.02	0.04		
Meconium	Actinobacteria	Vocabulary	Interaction	0.013	0.067		
Meconium	Actinobacteria	WISC sum	Acetaminophen	-0.712	5.058	0.714	0.952
Meconium	Actinobacteria	WISC sum	Phylum	-0.097	0.09		
Meconium	Actinobacteria	WISC sum	Interaction	0.051	0.152		
Meconium	Bacteroidetes	Block Design	Acetaminophen	1.196	1.254	0.678	0.952
Meconium	Bacteroidetes	Block Design	Phylum	0.057	0.152		
Meconium	Bacteroidetes	Block Design	Interaction	-0.075	0.198		
Meconium	Bacteroidetes	Coding	Acetaminophen	0.63	1.072	0.358	0.95
Meconium	Bacteroidetes	Coding	Phylum	0.196	0.13		
Meconium	Bacteroidetes	Coding	Interaction	-0.143	0.169		
Meconium	Bacteroidetes	Digit span	Acetaminophen	0.879	0.928	0.089	0.415
Meconium	Bacteroidetes	Digit span	Phylum	0.08	0.113		
Meconium	Bacteroidetes	Digit span	Interaction	-0.232	0.147		
Meconium	Bacteroidetes	Information	Acetaminophen	-0.466	0.941	0.756	0.952
Meconium	Bacteroidetes	Information	Phylum	0.027	0.114		

Exposure Window	Phylum	Outcome	Variable	Effect Estimate[a]	Std. Error	LRT[b]	
						p-value	q-value
Meconium	Bacteroidetes	Information	Interaction	0.042	0.149		
Meconium	Bacteroidetes	QTAC	Acetaminophen	-3.865	3.277	0.88	0.952
Meconium	Bacteroidetes	QTAC	Phylum	0.337	0.398		
Meconium	Bacteroidetes	QTAC	Interaction	-0.071	0.517		
Meconium	Bacteroidetes	Vocabulary	Acetaminophen	0.993	1.421	0.489	0.95
Meconium	Bacteroidetes	Vocabulary	Phylum	0.061	0.173		
Meconium	Bacteroidetes	Vocabulary	Interaction	-0.142	0.224		
Meconium	Bacteroidetes	WISC sum	Acetaminophen	3.232	3.224	0.241	0.766
Meconium	Bacteroidetes	WISC sum	Phylum	0.421	0.392		
Meconium	Bacteroidetes	WISC sum	Interaction	-0.55	0.509		
Meconium	Firmicutes	Block Design	Acetaminophen	0.211	3.745	0.831	0.952
Meconium	Firmicutes	Block Design	Phylum	-0.013	0.037		
Meconium	Firmicutes	Block Design	Interaction	0.011	0.058		
Meconium	Firmicutes	Coding	Acetaminophen	3.363	3.265	0.297	0.867
Meconium	Firmicutes	Coding	Phylum	0.022	0.032		
Meconium	Firmicutes	Coding	Interaction	-0.048	0.05		
Meconium	Firmicutes	Digit span	Acetaminophen	-2.096	2.77	0.468	0.95
Meconium	Firmicutes	Digit span	Phylum	0.025	0.027		
Meconium	Firmicutes	Digit span	Interaction	0.028	0.043		
Meconium	Firmicutes	Information	Acetaminophen	1.778	2.805	0.438	0.95
Meconium	Firmicutes	Information	Phylum	0.025	0.027		
Meconium	Firmicutes	Information	Interaction	-0.031	0.043		
Meconium	Firmicutes	QTAC	Acetaminophen	-17.961	9.576	0.089	0.415
Meconium	Firmicutes	QTAC	Phylum	-0.161	0.094		
Meconium	Firmicutes	QTAC	Interaction	0.234	0.148		
Meconium	Firmicutes	Vocabulary	Acetaminophen	0.148	4.254	0.967	0.968
Meconium	Firmicutes	Vocabulary	Phylum	0.015	0.042		
Meconium	Firmicutes	Vocabulary	Interaction	0.002	0.066		
Meconium	Firmicutes	WISC sum	Acetaminophen	3.405	9.695	0.788	0.952
Meconium	Firmicutes	WISC sum	Phylum	0.074	0.095		
Meconium	Firmicutes	WISC sum	Interaction	-0.037	0.15		
Meconium	Proteobacteria	Block Design	Acetaminophen	1.631	0.986	0.081	0.415
Meconium	Proteobacteria	Block Design	Phylum	3.367	2.929		

Exposure Window	Phylum	Outcome	Variable	Effect Estimate[a]	Std. Error	LRT[b]	
						p-value	q-value
Meconium	Proteobacteria	Block Design	Interaction	-6.5	4.008		
Meconium	Proteobacteria	Coding	Acetaminophen	0.947	0.867	0.095	0.415
Meconium	Proteobacteria	Coding	Phylum	1.636	2.575		
Meconium	Proteobacteria	Coding	Interaction	-5.459	3.523		
Meconium	Proteobacteria	Digit span	Acetaminophen	0.813	0.68	0.001	0.007
Meconium	Proteobacteria	Digit span	Phylum	3.163	2.019		
Meconium	Proteobacteria	Digit span	Interaction	-9.536	2.762		
Meconium	Proteobacteria	Information	Acetaminophen	0.687	0.695	0.007	0.081
Meconium	Proteobacteria	Information	Phylum	1.335	2.065		
Meconium	Proteobacteria	Information	Interaction	-7.248	2.826		
Meconium	Proteobacteria	QTAC	Acetaminophen	-3.013	2.688	0.787	0.952
Meconium	Proteobacteria	QTAC	Phylum	-2.196	7.984		
Meconium	Proteobacteria	QTAC	Interaction	-2.705	10.924		
Meconium	Proteobacteria	Vocabulary	Acetaminophen	1.355	1.047	0.028	0.241
Meconium	Proteobacteria	Vocabulary	Phylum	-0.453	3.108		
Meconium	Proteobacteria	Vocabulary	Interaction	-8.792	4.253		
Meconium	Proteobacteria	WISC sum	Acetaminophen	5.433	2.101	<0.001	<0.001
Meconium	Proteobacteria	WISC sum	Phylum	9.048	6.239		
Meconium	Proteobacteria	WISC sum	Interaction	-37.536	8.536		
Meconium	Verrucomicrobia	Block Design	Acetaminophen	1.076	1.004	0.634	0.952
Meconium	Verrucomicrobia	Block Design	Phylum	0.119	0.273		
Meconium	Verrucomicrobia	Block Design	Interaction	-0.146	0.335		
Meconium	Verrucomicrobia	Coding	Acetaminophen	0.156	0.884	0.686	0.952
Meconium	Verrucomicrobia	Coding	Phylum	-0.03	0.24		
Meconium	Verrucomicrobia	Coding	Interaction	0.109	0.295		
Meconium	Verrucomicrobia	Digit span	Acetaminophen	0.062	0.761	0.416	0.95
Meconium	Verrucomicrobia	Digit span	Phylum	0.027	0.207		
Meconium	Verrucomicrobia	Digit span	Interaction	-0.19	0.254		
Meconium	Verrucomicrobia	Information	Acetaminophen	-0.28	0.759	0.698	0.952
Meconium	Verrucomicrobia	Information	Phylum	-0.033	0.206		
Meconium	Verrucomicrobia	Information	Interaction	0.09	0.253		
Meconium	Verrucomicrobia	QTAC	Acetaminophen	-1.491	2.492	0.222	0.766
Meconium	Verrucomicrobia	QTAC	Phylum	-0.169	0.677		

Exposure Window	Phylum	Outcome	Variable	Effect Estimate[a]	Std. Error	LRT[b]	
						p-value	q-value
Meconium	Verrucomicrobia	QTAC	Interaction	-0.935	0.832		
Meconium	Verrucomicrobia	Vocabulary	Acetaminophen	0.596	1.141	0.606	0.952
Meconium	Verrucomicrobia	Vocabulary	Phylum	0.065	0.31		
Meconium	Verrucomicrobia	Vocabulary	Interaction	-0.18	0.381		
Meconium	Verrucomicrobia	WISC sum	Acetaminophen	1.61	2.62	0.693	0.952
Meconium	Verrucomicrobia	WISC sum	Phylum	0.149	0.711		
Meconium	Verrucomicrobia	WISC sum	Interaction	-0.316	0.874		
Meconium Adj.[c]	Actinobacteria	Block Design	Acetaminophen	0.875	1.994	0.982	0.982
Meconium Adj.[c]	Actinobacteria	Block Design	Phylum	0.003	0.035		
Meconium Adj.[c]	Actinobacteria	Block Design	Interaction	0.124	0.969		
Meconium Adj.[c]	Actinobacteria	Coding	Acetaminophen	-0.751	1.727	0.511	0.963
Meconium Adj.[c]	Actinobacteria	Coding	Phylum	-0.029	0.031		
Meconium Adj.[c]	Actinobacteria	Coding	Interaction	0.546	0.839		
Meconium Adj.[c]	Actinobacteria	Digit span	Acetaminophen	-0.248	1.502	0.908	0.982
Meconium Adj.[c]	Actinobacteria	Digit span	Phylum	-0.028	0.027		
Meconium Adj.[c]	Actinobacteria	Digit span	Interaction	-0.066	0.73		
Meconium Adj.[c]	Actinobacteria	Information	Acetaminophen	-0.351	1.451	0.969	0.982
Meconium Adj.[c]	Actinobacteria	Information	Phylum	-0.022	0.026		
Meconium Adj.[c]	Actinobacteria	Information	Interaction	1.051	0.705		
Meconium Adj.[c]	Actinobacteria	QTAC	Acetaminophen	2.123	4.996	0.257	0.903
Meconium Adj.[c]	Actinobacteria	QTAC	Phylum	0.119	0.089		
Meconium Adj.[c]	Actinobacteria	QTAC	Interaction	-4.036	2.427		
Meconium Adj.[c]	Actinobacteria	Vocabulary	Acetaminophen	-0.048	2.236	0.938	0.982
Meconium Adj.[c]	Actinobacteria	Vocabulary	Phylum	-0.019	0.04		
Meconium Adj.[c]	Actinobacteria	Vocabulary	Interaction	1.05	1.087		
Meconium Adj.[c]	Actinobacteria	WISC sum	Acetaminophen	-0.523	5.048	0.823	0.982
Meconium Adj.[c]	Actinobacteria	WISC sum	Phylum	-0.095	0.09		
Meconium Adj.[c]	Actinobacteria	WISC sum	Interaction	2.705	2.453		
Meconium Adj.[c]	Bacteroidetes	Block Design	Acetaminophen	1.177	1.295	0.692	0.982
Meconium Adj.[c]	Bacteroidetes	Block Design	Phylum	0.055	0.156		

Exposure Window	Phylum	Outcome	Variable	Effect Estimate[a]	Std. Error	LRT[b]	
						p-value	q-value
Meconium Adj.[c]	Bacteroidetes	Block Design	Interaction	0.072	0.973		
Meconium Adj.[c]	Bacteroidetes	Coding	Acetaminophen	0.521	1.104	0.409	0.963
Meconium Adj.[c]	Bacteroidetes	Coding	Phylum	0.185	0.133		
Meconium Adj.[c]	Bacteroidetes	Coding	Interaction	0.416	0.829		
Meconium Adj.[c]	Bacteroidetes	Digit span	Acetaminophen	0.949	0.957	0.081	0.436
Meconium Adj.[c]	Bacteroidetes	Digit span	Phylum	0.087	0.115		
Meconium Adj.[c]	Bacteroidetes	Digit span	Interaction	-0.265	0.719		
Meconium Adj.[c]	Bacteroidetes	Information	Acetaminophen	-0.739	0.947	0.571	0.963
Meconium Adj.[c]	Bacteroidetes	Information	Phylum	0	0.114		
Meconium Adj.[c]	Bacteroidetes	Information	Interaction	1.04	0.712		
Meconium Adj.[c]	Bacteroidetes	QTAC	Acetaminophen	-2.665	3.245	0.633	0.963
Meconium Adj.[c]	Bacteroidetes	QTAC	Phylum	0.453	0.392		
Meconium Adj.[c]	Bacteroidetes	QTAC	Interaction	-4.57	2.438		
Meconium Adj.[c]	Bacteroidetes	Vocabulary	Acetaminophen	0.739	1.453	0.59	0.963
Meconium Adj.[c]	Bacteroidetes	Vocabulary	Phylum	0.036	0.175		
Meconium Adj.[c]	Bacteroidetes	Vocabulary	Interaction	0.967	1.092		
Meconium Adj.[c]	Bacteroidetes	WISC sum	Acetaminophen	2.647	3.297	0.309	0.943
Meconium Adj.[c]	Bacteroidetes	WISC sum	Phylum	0.364	0.398		
Meconium Adj.[c]	Bacteroidetes	WISC sum	Interaction	2.23	2.477		
Meconium Adj.[c]	Firmicutes	Block Design	Acetaminophen	0.156	3.816	0.823	0.982
Meconium Adj.[c]	Firmicutes	Block Design	Phylum	-0.013	0.037		
Meconium Adj.[c]	Firmicutes	Block Design	Interaction	0.12	0.969		
Meconium Adj.[c]	Firmicutes	Coding	Acetaminophen	3.121	3.312	0.323	0.943
Meconium Adj.[c]	Firmicutes	Coding	Phylum	0.022	0.032		
Meconium Adj.[c]	Firmicutes	Coding	Interaction	0.529	0.841		
Meconium Adj.[c]	Firmicutes	Digit span	Acetaminophen	-2.118	2.824	0.466	0.963
Meconium Adj.[c]	Firmicutes	Digit span	Phylum	0.025	0.027		
Meconium Adj.[c]	Firmicutes	Digit span	Interaction	0.048	0.717		
Meconium Adj.[c]	Firmicutes	Information	Acetaminophen	1.304	2.785	0.508	0.963

Exposure Window	Phylum	Outcome	Variable	Effect Estimate[a]	Std. Error	LRT[b]	
						p-value	q-value
Meconium Adj.[c]	Firmicutes	Information	Phylum	0.026	0.027		
Meconium Adj.[c]	Firmicutes	Information	Interaction	1.034	0.707		
Meconium Adj.[c]	Firmicutes	QTAC	Acetaminophen	-16.021	9.398	0.108	0.473
Meconium Adj.[c]	Firmicutes	QTAC	Phylum	-0.166	0.091		
Meconium Adj.[c]	Firmicutes	QTAC	Interaction	-4.233	2.385		
Meconium Adj.[c]	Firmicutes	Vocabulary	Acetaminophen	-0.361	4.281	0.893	0.982
Meconium Adj.[c]	Firmicutes	Vocabulary	Phylum	0.016	0.042		
Meconium Adj.[c]	Firmicutes	Vocabulary	Interaction	1.11	1.087		
Meconium Adj.[c]	Firmicutes	WISC sum	Acetaminophen	2.103	9.723	0.867	0.982
Meconium Adj.[c]	Firmicutes	WISC sum	Phylum	0.077	0.094		
Meconium Adj.[c]	Firmicutes	WISC sum	Interaction	2.841	2.468		
Meconium Adj.[c]	Proteobacteria	Block Design	Acetaminophen	1.608	1.005	0.08	0.436
Meconium Adj.[c]	Proteobacteria	Block Design	Phylum	3.347	2.966		
Meconium Adj.[c]	Proteobacteria	Block Design	Interaction	0.181	0.935		
Meconium Adj.[c]	Proteobacteria	Coding	Acetaminophen	0.866	0.876	0.087	0.436
Meconium Adj.[c]	Proteobacteria	Coding	Phylum	1.565	2.587		
Meconium Adj.[c]	Proteobacteria	Coding	Interaction	0.66	0.815		
Meconium Adj.[c]	Proteobacteria	Digit span	Acetaminophen	0.812	0.693	<0.001	0.007
Meconium Adj.[c]	Proteobacteria	Digit span	Phylum	3.162	2.045		
Meconium Adj.[c]	Proteobacteria	Digit span	Interaction	0.008	0.645		
Meconium Adj.[c]	Proteobacteria	Information	Acetaminophen	0.544	0.681	0.004	0.049
Meconium Adj.[c]	Proteobacteria	Information	Phylum	1.211	2.01		
Meconium Adj.[c]	Proteobacteria	Information	Interaction	1.156	0.634		
Meconium Adj.[c]	Proteobacteria	QTAC	Acetaminophen	-2.504	2.65	0.827	0.982
Meconium Adj.[c]	Proteobacteria	QTAC	Phylum	-1.753	7.821		
Meconium Adj.[c]	Proteobacteria	QTAC	Interaction	-4.106	2.465		
Meconium Adj.[c]	Proteobacteria	Vocabulary	Acetaminophen	1.196	1.044	0.022	0.192
Meconium Adj.[c]	Proteobacteria	Vocabulary	Phylum	-0.591	3.083		
Meconium Adj.[c]	Proteobacteria	Vocabulary	Interaction	1.277	0.972		

Exposure Window	Phylum	Outcome	Variable	Effect Estimate[a]	Std. Error	LRT[b]	
						p-value	q-value
Meconium Adj.[c]	Proteobacteria	WISC sum	Acetaminophen	5.026	2.067	<0.001	<0.001
Meconium Adj.[c]	Proteobacteria	WISC sum	Phylum	8.694	6.101		
Meconium Adj.[c]	Proteobacteria	WISC sum	Interaction	3.282	1.923		
Meconium Adj.[c]	Verrucomicrobia	Block Design	Acetaminophen	1.06	1.021	0.626	0.963
Meconium Adj.[c]	Verrucomicrobia	Block Design	Phylum	0.12	0.276		
Meconium Adj.[c]	Verrucomicrobia	Block Design	Interaction	0.155	0.964		
Meconium Adj.[c]	Verrucomicrobia	Coding	Acetaminophen	0.101	0.894	0.722	0.982
Meconium Adj.[c]	Verrucomicrobia	Coding	Phylum	-0.027	0.242		
Meconium Adj.[c]	Verrucomicrobia	Coding	Interaction	0.537	0.844		
Meconium Adj.[c]	Verrucomicrobia	Digit span	Acetaminophen	0.065	0.774	0.419	0.963
Meconium Adj.[c]	Verrucomicrobia	Digit span	Phylum	0.027	0.209		
Meconium Adj.[c]	Verrucomicrobia	Digit span	Interaction	-0.036	0.73		
Meconium Adj.[c]	Verrucomicrobia	Information	Acetaminophen	-0.379	0.754	0.775	0.982
Meconium Adj.[c]	Verrucomicrobia	Information	Phylum	-0.026	0.204		
Meconium Adj.[c]	Verrucomicrobia	Information	Interaction	0.99	0.711		
Meconium Adj.[c]	Verrucomicrobia	QTAC	Acetaminophen	-1.119	2.458	0.258	0.903
Meconium Adj.[c]	Verrucomicrobia	QTAC	Phylum	-0.192	0.665		
Meconium Adj.[c]	Verrucomicrobia	QTAC	Interaction	-3.688	2.319		
Meconium Adj.[c]	Verrucomicrobia	Vocabulary	Acetaminophen	0.485	1.145	0.547	0.963
Meconium Adj.[c]	Verrucomicrobia	Vocabulary	Phylum	0.072	0.31		
Meconium Adj.[c]	Verrucomicrobia	Vocabulary	Interaction	1.106	1.081		
Meconium Adj.[c]	Verrucomicrobia	WISC sum	Acetaminophen	1.332	2.625	0.626	0.963
Meconium Adj.[c]	Verrucomicrobia	WISC sum	Phylum	0.166	0.71		
Meconium Adj.[c]	Verrucomicrobia	WISC sum	Interaction	2.751	2.476		

[a] Models are adjusted for whether the child was ever breastfed, sex, mode of birth, and socioeconomic status. Effect estimates for acetaminophen are the difference in outcome score between exposed and unexposed. Effect estimates for phylum are the change in outcome score for each 1% increase in phylum relative abundance. Effect estimates for the interaction are for the multiplicative interaction between acetaminophen and phylum relative abundance.
[b] Likelihood ratio test comparing to model without interaction
[c] Meconium Adj. includes cross-sectional exposure in the model

Table S9. Caffeine-phylum interactions in association with neurodevelopmental outcomes

Exposure Window	Phylum	Outcome	Variable	Effect Estimate[a]	Std. Error	LRT[b]	
						p-value	q-value
Meconium	Actinobacteria	Block Design	Caffeine	-0.105	0.533	0.675	0.913
Meconium	Actinobacteria	Block Design	Phylum	0.052	0.134		
Meconium	Actinobacteria	Block Design	Interaction	-0.006	0.016		
Meconium	Actinobacteria	Coding	Caffeine	-0.097	0.469	0.597	0.913
Meconium	Actinobacteria	Coding	Phylum	-0.076	0.118		
Meconium	Actinobacteria	Coding	Interaction	0.007	0.014		
Meconium	Actinobacteria	Digit span	Caffeine	-0.428	0.39	0.705	0.913
Meconium	Actinobacteria	Digit span	Phylum	-0.058	0.098		
Meconium	Actinobacteria	Digit span	Interaction	0.004	0.011		
Meconium	Actinobacteria	Information	Caffeine	0.284	0.395	0.235	0.748
Meconium	Actinobacteria	Information	Phylum	0.087	0.1		
Meconium	Actinobacteria	Information	Interaction	-0.013	0.011		
Meconium	Actinobacteria	QTAC	Caffeine	-1.04	1.412	0.861	0.972
Meconium	Actinobacteria	QTAC	Phylum	0.029	0.356		
Meconium	Actinobacteria	QTAC	Interaction	0.007	0.041		
Meconium	Actinobacteria	Vocabulary	Caffeine	-0.127	0.61	0.987	0.988
Meconium	Actinobacteria	Vocabulary	Phylum	-0.014	0.154		
Meconium	Actinobacteria	Vocabulary	Interaction	0	0.018		
Meconium	Actinobacteria	WISC sum	Caffeine	-0.472	1.359	0.82	0.97
Meconium	Actinobacteria	WISC sum	Phylum	-0.009	0.342		
Meconium	Actinobacteria	WISC sum	Interaction	-0.008	0.04		
Meconium	Bacteroidetes	Block Design	Caffeine	-0.558	0.297	0.15	0.65
Meconium	Bacteroidetes	Block Design	Phylum	-0.401	0.331		
Meconium	Bacteroidetes	Block Design	Interaction	0.053	0.039		
Meconium	Bacteroidetes	Coding	Caffeine	0.23	0.262	0.526	0.913
Meconium	Bacteroidetes	Coding	Phylum	0.276	0.291		
Meconium	Bacteroidetes	Coding	Interaction	-0.02	0.035		

Exposure Window	Phylum	Outcome	Variable	Effect Estimate[a]	Std. Error	LRT[b]	
						p-value	q-value
Meconium	Bacteroidetes	Digit span	Caffeine	-0.1	0.216	0.093	0.65
Meconium	Bacteroidetes	Digit span	Phylum	0.286	0.24		
Meconium	Bacteroidetes	Digit span	Interaction	-0.045	0.029		
Meconium	Bacteroidetes	Information	Caffeine	-0.105	0.229	0.976	0.988
Meconium	Bacteroidetes	Information	Phylum	0.048	0.255		
Meconium	Bacteroidetes	Information	Interaction	-0.001	0.03		
Meconium	Bacteroidetes	QTAC	Caffeine	-0.091	0.797	0.186	0.65
Meconium	Bacteroidetes	QTAC	Phylum	1.204	0.886		
Meconium	Bacteroidetes	QTAC	Interaction	-0.129	0.106		
Meconium	Bacteroidetes	Vocabulary	Caffeine	0.045	0.345	0.379	0.885
Meconium	Bacteroidetes	Vocabulary	Phylum	0.278	0.384		
Meconium	Bacteroidetes	Vocabulary	Interaction	-0.037	0.046		
Meconium	Bacteroidetes	WISC sum	Caffeine	-0.488	0.781	0.598	0.913
Meconium	Bacteroidetes	WISC sum	Phylum	0.487	0.869		
Meconium	Bacteroidetes	WISC sum	Interaction	-0.05	0.104		
Meconium	Firmicutes	Block Design	Caffeine	0.061	0.945	0.675	0.913
Meconium	Firmicutes	Block Design	Phylum	0.043	0.135		
Meconium	Firmicutes	Block Design	Interaction	-0.006	0.015		
Meconium	Firmicutes	Coding	Caffeine	0.528	0.837	0.569	0.913
Meconium	Firmicutes	Coding	Phylum	0.064	0.119		
Meconium	Firmicutes	Coding	Interaction	-0.007	0.014		
Meconium	Firmicutes	Digit span	Caffeine	-0.229	0.68	0.892	0.976
Meconium	Firmicutes	Digit span	Phylum	0.047	0.097		
Meconium	Firmicutes	Digit span	Interaction	-0.001	0.011		
Meconium	Firmicutes	Information	Caffeine	-0.741	0.708	0.324	0.81
Meconium	Firmicutes	Information	Phylum	-0.077	0.101		
Meconium	Firmicutes	Information	Interaction	0.011	0.012		
Meconium	Firmicutes	QTAC	Caffeine	-1.673	2.509	0.698	0.913

Exposure Window	Phylum	Outcome	Variable	Effect Estimate[a]	Std. Error	LRT[b]	
						p-value	q-value
Meconium	Firmicutes	QTAC	Phylum	-0.199	0.357		
Meconium	Firmicutes	QTAC	Interaction	0.015	0.041		
Meconium	Firmicutes	Vocabulary	Caffeine	-0.345	1.081	0.832	0.97
Meconium	Firmicutes	Vocabulary	Phylum	-0.014	0.154		
Meconium	Firmicutes	Vocabulary	Interaction	0.003	0.018		
Meconium	Firmicutes	WISC sum	Caffeine	-0.726	2.426	0.988	0.988
Meconium	Firmicutes	WISC sum	Phylum	0.063	0.346		
Meconium	Firmicutes	WISC sum	Interaction	-0.001	0.04		
Meconium	Proteobacteria	Block Design	Caffeine	-0.238	0.247	0.559	0.913
Meconium	Proteobacteria	Block Design	Phylum	5.533	11.185		
Meconium	Proteobacteria	Block Design	Interaction	-0.766	1.432		
Meconium	Proteobacteria	Coding	Caffeine	0.212	0.214	0.145	0.65
Meconium	Proteobacteria	Coding	Phylum	11.861	9.716		
Meconium	Proteobacteria	Coding	Interaction	-1.676	1.244		
Meconium	Proteobacteria	Digit span	Caffeine	-0.089	0.153	<0.001	0.003
Meconium	Proteobacteria	Digit span	Phylum	24.452	6.953		
Meconium	Proteobacteria	Digit span	Interaction	-3.485	0.89		
Meconium	Proteobacteria	Information	Caffeine	-0.051	0.177	0.128	0.65
Meconium	Proteobacteria	Information	Phylum	8.529	8.052		
Meconium	Proteobacteria	Information	Interaction	-1.454	1.031		
Meconium	Proteobacteria	QTAC	Caffeine	-0.418	0.637	0.076	0.65
Meconium	Proteobacteria	QTAC	Phylum	41.629	28.896		
Meconium	Proteobacteria	QTAC	Interaction	-6.106	3.699		
Meconium	Proteobacteria	Vocabulary	Caffeine	-0.064	0.261	0.124	0.65
Meconium	Proteobacteria	Vocabulary	Phylum	11.284	11.844		
Meconium	Proteobacteria	Vocabulary	Interaction	-2.158	1.516		
Meconium	Proteobacteria	WISC sum	Caffeine	-0.23	0.548	0.002	0.032
Meconium	Proteobacteria	WISC sum	Phylum	61.659	24.885		

Exposure Window	Phylum	Outcome	Variable	Effect Estimate[a]	Std. Error	LRT[b]	
						p-value	q-value
Meconium	Proteobacteria	WISC sum	Interaction	-9.539	3.185		
Meconium	Verrucomicrobia	Block Design	Caffeine	-0.35	0.256	0.624	0.913
Meconium	Verrucomicrobia	Block Design	Phylum	-0.178	0.501		
Meconium	Verrucomicrobia	Block Design	Interaction	0.024	0.054		
Meconium	Verrucomicrobia	Coding	Caffeine	-0.006	0.225	0.31	0.81
Meconium	Verrucomicrobia	Coding	Phylum	-0.342	0.44		
Meconium	Verrucomicrobia	Coding	Interaction	0.044	0.047		
Meconium	Verrucomicrobia	Digit span	Caffeine	-0.387	0.187	0.431	0.913
Meconium	Verrucomicrobia	Digit span	Phylum	-0.363	0.367		
Meconium	Verrucomicrobia	Digit span	Interaction	0.028	0.039		
Meconium	Verrucomicrobia	Information	Caffeine	-0.212	0.192	0.303	0.81
Meconium	Verrucomicrobia	Information	Phylum	-0.317	0.377		
Meconium	Verrucomicrobia	Information	Interaction	0.038	0.04		
Meconium	Verrucomicrobia	QTAC	Caffeine	-0.738	0.644	0.819	0.97
Meconium	Verrucomicrobia	QTAC	Phylum	-0.622	1.261		
Meconium	Verrucomicrobia	QTAC	Interaction	-0.028	0.135		
Meconium	Verrucomicrobia	Vocabulary	Caffeine	-0.228	0.292	0.541	0.913
Meconium	Verrucomicrobia	Vocabulary	Phylum	-0.358	0.572		
Meconium	Verrucomicrobia	Vocabulary	Interaction	0.034	0.061		
Meconium	Verrucomicrobia	WISC sum	Caffeine	-1.184	0.65	0.179	0.65
Meconium	Verrucomicrobia	WISC sum	Phylum	-1.559	1.273		
Meconium	Verrucomicrobia	WISC sum	Interaction	0.17	0.137		
Meconium Adj.[c]	Actinobacteria	Block Design	Caffeine	-0.117	0.541	0.693	0.961
Meconium Adj.[c]	Actinobacteria	Block Design	Phylum	0.049	0.136		
Meconium Adj.[c]	Actinobacteria	Block Design	Interaction	0.266	0.943		
Meconium Adj.[c]	Actinobacteria	Coding	Caffeine	-0.125	0.472	0.55	0.949
Meconium Adj.[c]	Actinobacteria	Coding	Phylum	-0.084	0.119		
Meconium Adj.[c]	Actinobacteria	Coding	Interaction	0.655	0.824		

Exposure Window	Phylum	Outcome	Variable	Effect Estimate[a]	Std. Error	LRT[b]	
						p-value	q-value
Meconium Adj.[c]	Actinobacteria	Digit span	Caffeine	-0.424	0.396	0.714	0.961
Meconium Adj.[c]	Actinobacteria	Digit span	Phylum	-0.057	0.1		
Meconium Adj.[c]	Actinobacteria	Digit span	Interaction	-0.094	0.69		
Meconium Adj.[c]	Actinobacteria	Information	Caffeine	0.243	0.392	0.272	0.866
Meconium Adj.[c]	Actinobacteria	Information	Phylum	0.077	0.099		
Meconium Adj.[c]	Actinobacteria	Information	Interaction	0.944	0.684		
Meconium Adj.[c]	Actinobacteria	QTAC	Caffeine	-0.837	1.37	0.983	0.989
Meconium Adj.[c]	Actinobacteria	QTAC	Phylum	0.082	0.345		
Meconium Adj.[c]	Actinobacteria	QTAC	Interaction	-4.643	2.39		
Meconium Adj.[c]	Actinobacteria	Vocabulary	Caffeine	-0.175	0.611	0.945	0.989
Meconium Adj.[c]	Actinobacteria	Vocabulary	Phylum	-0.026	0.154		
Meconium Adj.[c]	Actinobacteria	Vocabulary	Interaction	1.089	1.066		
Meconium Adj.[c]	Actinobacteria	WISC sum	Caffeine	-0.597	1.355	0.897	0.989
Meconium Adj.[c]	Actinobacteria	WISC sum	Phylum	-0.042	0.341		
Meconium Adj.[c]	Actinobacteria	WISC sum	Interaction	2.861	2.364		
Meconium Adj.[c]	Bacteroidetes	Block Design	Caffeine	-0.581	0.303	0.128	0.56
Meconium Adj.[c]	Bacteroidetes	Block Design	Phylum	-0.435	0.34		
Meconium Adj.[c]	Bacteroidetes	Block Design	Interaction	0.494	0.937		
Meconium Adj.[c]	Bacteroidetes	Coding	Caffeine	0.211	0.267	0.597	0.949
Meconium Adj.[c]	Bacteroidetes	Coding	Phylum	0.247	0.299		
Meconium Adj.[c]	Bacteroidetes	Coding	Interaction	0.426	0.824		
Meconium Adj.[c]	Bacteroidetes	Digit span	Caffeine	-0.089	0.221	0.085	0.56
Meconium Adj.[c]	Bacteroidetes	Digit span	Phylum	0.302	0.247		
Meconium Adj.[c]	Bacteroidetes	Digit span	Interaction	-0.241	0.682		
Meconium Adj.[c]	Bacteroidetes	Information	Caffeine	-0.149	0.229	0.818	0.988
Meconium Adj.[c]	Bacteroidetes	Information	Phylum	-0.019	0.257		
Meconium Adj.[c]	Bacteroidetes	Information	Interaction	0.97	0.709		
Meconium Adj.[c]	Bacteroidetes	QTAC	Caffeine	0.154	0.766	0.071	0.56

Exposure Window	Phylum	Outcome	Variable	Effect Estimate[a]	Std. Error	LRT[b]	
						p-value	q-value
Meconium Adj.[c]	Bacteroidetes	QTAC	Phylum	1.579	0.859		
Meconium Adj.[c]	Bacteroidetes	QTAC	Interaction	-5.448	2.368		
Meconium Adj.[c]	Bacteroidetes	Vocabulary	Caffeine	0.001	0.349	0.482	0.936
Meconium Adj.[c]	Bacteroidetes	Vocabulary	Phylum	0.21	0.392		
Meconium Adj.[c]	Bacteroidetes	Vocabulary	Interaction	0.984	1.08		
Meconium Adj.[c]	Bacteroidetes	WISC sum	Caffeine	-0.606	0.787	0.747	0.968
Meconium Adj.[c]	Bacteroidetes	WISC sum	Phylum	0.306	0.883		
Meconium Adj.[c]	Bacteroidetes	WISC sum	Interaction	2.634	2.435		
Meconium Adj.[c]	Firmicutes	Block Design	Caffeine	0.117	0.968	0.63	0.949
Meconium Adj.[c]	Firmicutes	Block Design	Phylum	0.052	0.139		
Meconium Adj.[c]	Firmicutes	Block Design	Interaction	0.341	0.958		
Meconium Adj.[c]	Firmicutes	Coding	Caffeine	0.645	0.852	0.467	0.936
Meconium Adj.[c]	Firmicutes	Coding	Phylum	0.084	0.122		
Meconium Adj.[c]	Firmicutes	Coding	Interaction	0.716	0.843		
Meconium Adj.[c]	Firmicutes	Digit span	Caffeine	-0.229	0.698	0.894	0.989
Meconium Adj.[c]	Firmicutes	Digit span	Phylum	0.047	0.1		
Meconium Adj.[c]	Firmicutes	Digit span	Interaction	-0.001	0.69		
Meconium Adj.[c]	Firmicutes	Information	Caffeine	-0.586	0.711	0.459	0.936
Meconium Adj.[c]	Firmicutes	Information	Phylum	-0.052	0.102		
Meconium Adj.[c]	Firmicutes	Information	Interaction	0.948	0.703		
Meconium Adj.[c]	Firmicutes	QTAC	Caffeine	-2.522	2.438	0.419	0.936
Meconium Adj.[c]	Firmicutes	QTAC	Phylum	-0.34	0.349		
Meconium Adj.[c]	Firmicutes	QTAC	Interaction	-5.172	2.412		
Meconium Adj.[c]	Firmicutes	Vocabulary	Caffeine	-0.159	1.094	0.989	0.989
Meconium Adj.[c]	Firmicutes	Vocabulary	Phylum	0.017	0.157		
Meconium Adj.[c]	Firmicutes	Vocabulary	Interaction	1.131	1.083		
Meconium Adj.[c]	Firmicutes	WISC sum	Caffeine	-0.211	2.438	0.794	0.988
Meconium Adj.[c]	Firmicutes	WISC sum	Phylum	0.149	0.349		

Exposure Window	Phylum	Outcome	Variable	Effect Estimate[a]	Std. Error	LRT[b]	
						p-value	q-value
Meconium Adj.[c]	Firmicutes	WISC sum	Interaction	3.136	2.413		
Meconium Adj.[c]	Proteobacteria	Block Design	Caffeine	-0.241	0.249	0.564	0.949
Meconium Adj.[c]	Proteobacteria	Block Design	Phylum	5.4	11.318		
Meconium Adj.[c]	Proteobacteria	Block Design	Interaction	0.298	0.942		
Meconium Adj.[c]	Proteobacteria	Coding	Caffeine	0.206	0.215	0.147	0.571
Meconium Adj.[c]	Proteobacteria	Coding	Phylum	11.582	9.772		
Meconium Adj.[c]	Proteobacteria	Coding	Interaction	0.623	0.813		
Meconium Adj.[c]	Proteobacteria	Digit span	Caffeine	-0.088	0.155	<0.001	0.003
Meconium Adj.[c]	Proteobacteria	Digit span	Phylum	24.495	7.042		
Meconium Adj.[c]	Proteobacteria	Digit span	Interaction	-0.096	0.586		
Meconium Adj.[c]	Proteobacteria	Information	Caffeine	-0.062	0.174	0.125	0.56
Meconium Adj.[c]	Proteobacteria	Information	Phylum	8.053	7.904		
Meconium Adj.[c]	Proteobacteria	Information	Interaction	1.064	0.658		
Meconium Adj.[c]	Proteobacteria	QTAC	Caffeine	-0.371	0.617	0.058	0.56
Meconium Adj.[c]	Proteobacteria	QTAC	Phylum	43.641	28		
Meconium Adj.[c]	Proteobacteria	QTAC	Interaction	-4.497	2.33		
Meconium Adj.[c]	Proteobacteria	Vocabulary	Caffeine	-0.078	0.259	0.124	0.56
Meconium Adj.[c]	Proteobacteria	Vocabulary	Phylum	10.727	11.764		
Meconium Adj.[c]	Proteobacteria	Vocabulary	Interaction	1.246	0.979		
Meconium Adj.[c]	Proteobacteria	WISC sum	Caffeine	-0.263	0.54	0.002	0.027
Meconium Adj.[c]	Proteobacteria	WISC sum	Phylum	60.257	24.498		
Meconium Adj.[c]	Proteobacteria	WISC sum	Interaction	3.134	2.038		
Meconium Adj.[c]	Verrucomicrobia	Block Design	Caffeine	-0.347	0.259	0.651	0.949
Meconium Adj.[c]	Verrucomicrobia	Block Design	Phylum	-0.166	0.51		
Meconium Adj.[c]	Verrucomicrobia	Block Design	Interaction	0.222	0.951		
Meconium Adj.[c]	Verrucomicrobia	Coding	Caffeine	0	0.227	0.354	0.936
Meconium Adj.[c]	Verrucomicrobia	Coding	Phylum	-0.316	0.446		
Meconium Adj.[c]	Verrucomicrobia	Coding	Interaction	0.485	0.833		

Exposure Window	Phylum	Outcome	Variable	Effect Estimate[a]	Std. Error	LRT[b]	
						p-value	q-value
Meconium Adj.[c]	Verrucomicrobia	Digit span	Caffeine	-0.389	0.19	0.418	0.936
Meconium Adj.[c]	Verrucomicrobia	Digit span	Phylum	-0.37	0.373		
Meconium Adj.[c]	Verrucomicrobia	Digit span	Interaction	-0.136	0.697		
Meconium Adj.[c]	Verrucomicrobia	Information	Caffeine	-0.2	0.191	0.392	0.936
Meconium Adj.[c]	Verrucomicrobia	Information	Phylum	-0.269	0.376		
Meconium Adj.[c]	Verrucomicrobia	Information	Interaction	0.9	0.701		
Meconium Adj.[c]	Verrucomicrobia	QTAC	Caffeine	-0.795	0.629	0.981	0.989
Meconium Adj.[c]	Verrucomicrobia	QTAC	Phylum	-0.843	1.236		
Meconium Adj.[c]	Verrucomicrobia	QTAC	Interaction	-4.092	2.307		
Meconium Adj.[c]	Verrucomicrobia	Vocabulary	Caffeine	-0.213	0.293	0.637	0.949
Meconium Adj.[c]	Verrucomicrobia	Vocabulary	Phylum	-0.302	0.576		
Meconium Adj.[c]	Verrucomicrobia	Vocabulary	Interaction	1.049	1.075		
Meconium Adj.[c]	Verrucomicrobia	WISC sum	Caffeine	-1.149	0.65	0.23	0.807
Meconium Adj.[c]	Verrucomicrobia	WISC sum	Phylum	-1.423	1.278		
Meconium Adj.[c]	Verrucomicrobia	WISC sum	Interaction	2.519	2.387		

[a] Models are adjusted for whether the child was ever breastfed, sex, mode of birth, and socioeconomic status. Effect estimates for caffeine are the change in outcome score for each doubling of exposure. Effect estimates for phylum are the change in outcome score for each 1% increase in phylum relative abundance. Effect estimates for the interaction are for the multiplicative interaction between acetaminophen and phylum relative abundance.
[b] Likelihood ratio test comparing to model without interaction
[c] Meconium Adj. includes cross-sectional exposure in the model

Table S10. Caffeine-species interactions in association with neurodevelopment

Exposure Window	Species	Outcome	Variable	Effect Estimate[a]	Std. Error	LRT[a]	
						p-value	q-value
Meconium	Agathobaculum butyriciproducens	Block Design	Caffeine	-0.188	0.257	0.558	0.969
Meconium	Agathobaculum butyriciproducens	Block Design	Species	0.974	2.717		
Meconium	Agathobaculum butyriciproducens	Block Design	Interaction	-0.166	0.31		
Meconium	Agathobaculum butyriciproducens	Coding	Caffeine	-0.041	0.226	0.15	0.916
Meconium	Agathobaculum butyriciproducens	Coding	Species	-3.408	2.389		
Meconium	Agathobaculum butyriciproducens	Coding	Interaction	0.363	0.273		
Meconium	Agathobaculum butyriciproducens	Digit span	Caffeine	-0.267	0.192	0.492	0.969
Meconium	Agathobaculum butyriciproducens	Digit span	Species	1.522	2.031		
Meconium	Agathobaculum butyriciproducens	Digit span	Interaction	-0.146	0.232		
Meconium	Agathobaculum butyriciproducens	Information	Caffeine	-0.119	0.191	0.719	0.969
Meconium	Agathobaculum butyriciproducens	Information	Species	1.205	2.019		
Meconium	Agathobaculum butyriciproducens	Information	Interaction	-0.076	0.231		
Meconium	Agathobaculum butyriciproducens	QTAC	Caffeine	-1	0.687	0.407	0.969
Meconium	Agathobaculum butyriciproducens	QTAC	Species	-6.663	7.275		
Meconium	Agathobaculum butyriciproducens	QTAC	Interaction	0.632	0.831		
Meconium	Agathobaculum butyriciproducens	Vocabulary	Caffeine	-0.197	0.297	0.836	0.971
Meconium	Agathobaculum butyriciproducens	Vocabulary	Species	-0.198	3.141		
Meconium	Agathobaculum butyriciproducens	Vocabulary	Interaction	0.068	0.359		
Meconium	Agathobaculum butyriciproducens	WISC sum	Caffeine	-0.812	0.673	0.955	0.978
Meconium	Agathobaculum butyriciproducens	WISC sum	Species	0.096	7.127		
Meconium	Agathobaculum butyriciproducens	WISC sum	Interaction	0.042	0.814		
Meconium	Akkermansia muciniphila	Block Design	Caffeine	-0.35	0.256	0.624	0.969
Meconium	Akkermansia muciniphila	Block Design	Species	-0.178	0.501		
Meconium	Akkermansia muciniphila	Block Design	Interaction	0.024	0.054		
Meconium	Akkermansia muciniphila	Coding	Caffeine	-0.006	0.225	0.31	0.957
Meconium	Akkermansia muciniphila	Coding	Species	-0.342	0.44		
Meconium	Akkermansia muciniphila	Coding	Interaction	0.044	0.047		
Meconium	Akkermansia muciniphila	Digit span	Caffeine	-0.387	0.187	0.431	0.969
Meconium	Akkermansia muciniphila	Digit span	Species	-0.363	0.367		
Meconium	Akkermansia muciniphila	Digit span	Interaction	0.028	0.039		
Meconium	Akkermansia muciniphila	Information	Caffeine	-0.212	0.192	0.303	0.949
Meconium	Akkermansia muciniphila	Information	Species	-0.317	0.377		
Meconium	Akkermansia muciniphila	Information	Interaction	0.038	0.04		
Meconium	Akkermansia muciniphila	QTAC	Caffeine	-0.738	0.644	0.819	0.969
Meconium	Akkermansia muciniphila	QTAC	Species	-0.622	1.261		

Exposure Window	Species	Outcome	Variable	Effect Estimate[a]	Std. Error	LRT[a]	
						p-value	q-value
Meconium	Akkermansia muciniphila	QTAC	Interaction	-0.028	0.135		
Meconium	Akkermansia muciniphila	Vocabulary	Caffeine	-0.228	0.292	0.541	0.969
Meconium	Akkermansia muciniphila	Vocabulary	Species	-0.358	0.572		
Meconium	Akkermansia muciniphila	Vocabulary	Interaction	0.034	0.061		
Meconium	Akkermansia muciniphila	WISC sum	Caffeine	-1.184	0.65	0.179	0.916
Meconium	Akkermansia muciniphila	WISC sum	Species	-1.559	1.273		
Meconium	Akkermansia muciniphila	WISC sum	Interaction	0.17	0.137		
Meconium	Alistipes putredinis	Block Design	Caffeine	-0.485	0.262	0.16	0.916
Meconium	Alistipes putredinis	Block Design	Species	-1.899	1.461		
Meconium	Alistipes putredinis	Block Design	Interaction	0.209	0.161		
Meconium	Alistipes putredinis	Coding	Caffeine	0.184	0.229	0.506	0.969
Meconium	Alistipes putredinis	Coding	Species	1.279	1.279		
Meconium	Alistipes putredinis	Coding	Interaction	-0.086	0.141		
Meconium	Alistipes putredinis	Digit span	Caffeine	-0.234	0.195	0.416	0.969
Meconium	Alistipes putredinis	Digit span	Species	0.474	1.086		
Meconium	Alistipes putredinis	Digit span	Interaction	-0.089	0.12		
Meconium	Alistipes putredinis	Information	Caffeine	-0.043	0.194	0.47	0.969
Meconium	Alistipes putredinis	Information	Species	1.204	1.081		
Meconium	Alistipes putredinis	Information	Interaction	-0.079	0.119		
Meconium	Alistipes putredinis	QTAC	Caffeine	-0.461	0.711	0.371	0.969
Meconium	Alistipes putredinis	QTAC	Species	3.341	3.967		
Meconium	Alistipes putredinis	QTAC	Interaction	-0.359	0.437		
Meconium	Alistipes putredinis	Vocabulary	Caffeine	0.079	0.3	0.163	0.916
Meconium	Alistipes putredinis	Vocabulary	Species	2.191	1.672		
Meconium	Alistipes putredinis	Vocabulary	Interaction	-0.238	0.184		
Meconium	Alistipes putredinis	WISC sum	Caffeine	-0.499	0.681	0.461	0.969
Meconium	Alistipes putredinis	WISC sum	Species	3.248	3.8		
Meconium	Alistipes putredinis	WISC sum	Interaction	-0.283	0.419		
Meconium	Anaerostipes hadrus	Block Design	Caffeine	0.028	0.386	0.267	0.938
Meconium	Anaerostipes hadrus	Block Design	Species	0.483	0.49		
Meconium	Anaerostipes hadrus	Block Design	Interaction	-0.068	0.067		
Meconium	Anaerostipes hadrus	Coding	Caffeine	-0.108	0.335	0.24	0.916
Meconium	Anaerostipes hadrus	Coding	Species	-0.291	0.424		
Meconium	Anaerostipes hadrus	Coding	Interaction	0.063	0.058		
Meconium	Anaerostipes hadrus	Digit span	Caffeine	-0.43	0.29	0.656	0.969
Meconium	Anaerostipes hadrus	Digit span	Species	-0.179	0.367		

Exposure Window	Species	Outcome	Variable	Effect Estimate[a]	Std. Error	LRT[a]	
						p-value	q-value
Meconium	Anaerostipes hadrus	Digit span	Interaction	0.02	0.05		
Meconium	Anaerostipes hadrus	Information	Caffeine	0.064	0.293	0.503	0.969
Meconium	Anaerostipes hadrus	Information	Species	0.288	0.372		
Meconium	Anaerostipes hadrus	Information	Interaction	-0.031	0.051		
Meconium	Anaerostipes hadrus	QTAC	Caffeine	-1.745	1.027	0.174	0.916
Meconium	Anaerostipes hadrus	QTAC	Species	-1.408	1.302		
Meconium	Anaerostipes hadrus	QTAC	Interaction	0.223	0.178		
Meconium	Anaerostipes hadrus	Vocabulary	Caffeine	0.31	0.438	0.233	0.916
Meconium	Anaerostipes hadrus	Vocabulary	Species	0.705	0.555		
Meconium	Anaerostipes hadrus	Vocabulary	Interaction	-0.083	0.076		
Meconium	Anaerostipes hadrus	WISC sum	Caffeine	-0.136	0.995	0.528	0.969
Meconium	Anaerostipes hadrus	WISC sum	Species	1.006	1.262		
Meconium	Anaerostipes hadrus	WISC sum	Interaction	-0.1	0.172		
Meconium	Asaccharobacter celatus	Block Design	Caffeine	-0.244	0.365	0.8	0.969
Meconium	Asaccharobacter celatus	Block Design	Species	0.448	3.877		
Meconium	Asaccharobacter celatus	Block Design	Interaction	-0.106	0.457		
Meconium	Asaccharobacter celatus	Coding	Caffeine	0.738	0.303	0.006	0.659
Meconium	Asaccharobacter celatus	Coding	Species	8.353	3.213		
Meconium	Asaccharobacter celatus	Coding	Interaction	-1	0.379		
Meconium	Asaccharobacter celatus	Digit span	Caffeine	0.001	0.267	0.118	0.916
Meconium	Asaccharobacter celatus	Digit span	Species	4.206	2.838		
Meconium	Asaccharobacter celatus	Digit span	Interaction	-0.484	0.335		
Meconium	Asaccharobacter celatus	Information	Caffeine	0.029	0.278	0.484	0.969
Meconium	Asaccharobacter celatus	Information	Species	1.964	2.953		
Meconium	Asaccharobacter celatus	Information	Interaction	-0.224	0.348		
Meconium	Asaccharobacter celatus	QTAC	Caffeine	-1.408	0.979	0.372	0.969
Meconium	Asaccharobacter celatus	QTAC	Species	-7.821	10.4		
Meconium	Asaccharobacter celatus	QTAC	Interaction	1.005	1.227		
Meconium	Asaccharobacter celatus	Vocabulary	Caffeine	-0.17	0.422	0.923	0.978
Meconium	Asaccharobacter celatus	Vocabulary	Species	-0.351	4.484		
Meconium	Asaccharobacter celatus	Vocabulary	Interaction	0.047	0.529		
Meconium	Asaccharobacter celatus	WISC sum	Caffeine	0.354	0.926	0.1	0.916
Meconium	Asaccharobacter celatus	WISC sum	Species	14.621	9.833		
Meconium	Asaccharobacter celatus	WISC sum	Interaction	-1.768	1.16		
Meconium	Bacteroides vulgatus	Block Design	Caffeine	-0.475	0.25	0.09	0.916
Meconium	Bacteroides vulgatus	Block Design	Species	-2.931	1.947		

Exposure Window	Species	Outcome	Variable	Effect Estimate[a]	Std. Error	LRT[a]	
						p-value	q-value
Meconium	Bacteroides vulgatus	Block Design	Interaction	0.465	0.295		
Meconium	Bacteroides vulgatus	Coding	Caffeine	0.148	0.22	0.723	0.969
Meconium	Bacteroides vulgatus	Coding	Species	0.187	1.712		
Meconium	Bacteroides vulgatus	Coding	Interaction	0.084	0.259		
Meconium	Bacteroides vulgatus	Digit span	Caffeine	-0.201	0.188	0.184	0.916
Meconium	Bacteroides vulgatus	Digit span	Species	1.776	1.465		
Meconium	Bacteroides vulgatus	Digit span	Interaction	-0.272	0.222		
Meconium	Bacteroides vulgatus	Information	Caffeine	-0.082	0.195	0.765	0.969
Meconium	Bacteroides vulgatus	Information	Species	0.504	1.517		
Meconium	Bacteroides vulgatus	Information	Interaction	-0.063	0.23		
Meconium	Bacteroides vulgatus	QTAC	Caffeine	-0.524	0.679	0.718	0.969
Meconium	Bacteroides vulgatus	QTAC	Species	3.182	5.286		
Meconium	Bacteroides vulgatus	QTAC	Interaction	-0.265	0.801		
Meconium	Bacteroides vulgatus	Vocabulary	Caffeine	-0.107	0.294	0.741	0.969
Meconium	Bacteroides vulgatus	Vocabulary	Species	0.607	2.293		
Meconium	Bacteroides vulgatus	Vocabulary	Interaction	-0.105	0.347		
Meconium	Bacteroides vulgatus	WISC sum	Caffeine	-0.717	0.661	0.879	0.978
Meconium	Bacteroides vulgatus	WISC sum	Species	0.143	5.146		
Meconium	Bacteroides vulgatus	WISC sum	Interaction	0.109	0.779		
Meconium	Bifidobacterium adolescentis	Block Design	Caffeine	-0.26	0.305	0.864	0.978
Meconium	Bifidobacterium adolescentis	Block Design	Species	0.008	0.298		
Meconium	Bifidobacterium adolescentis	Block Design	Interaction	-0.006	0.036		
Meconium	Bifidobacterium adolescentis	Coding	Caffeine	-0.281	0.257	0.024	0.659
Meconium	Bifidobacterium adolescentis	Coding	Species	-0.561	0.251		
Meconium	Bifidobacterium adolescentis	Coding	Interaction	0.063	0.03		
Meconium	Bifidobacterium adolescentis	Digit span	Caffeine	-0.239	0.227	0.592	0.969
Meconium	Bifidobacterium adolescentis	Digit span	Species	0.087	0.222		
Meconium	Bifidobacterium adolescentis	Digit span	Interaction	-0.013	0.026		
Meconium	Bifidobacterium adolescentis	Information	Caffeine	0.221	0.218	0.017	0.659
Meconium	Bifidobacterium adolescentis	Information	Species	0.431	0.213		
Meconium	Bifidobacterium adolescentis	Information	Interaction	-0.057	0.025		
Meconium	Bifidobacterium adolescentis	QTAC	Caffeine	-1.481	0.79	0.187	0.916
Meconium	Bifidobacterium adolescentis	QTAC	Species	-1.153	0.771		
Meconium	Bifidobacterium adolescentis	QTAC	Interaction	0.112	0.092		
Meconium	Bifidobacterium adolescentis	Vocabulary	Caffeine	0.116	0.333	0.215	0.916
Meconium	Bifidobacterium adolescentis	Vocabulary	Species	0.238	0.325		

Exposure Window	Species	Outcome	Variable	Effect Estimate[a]	Std. Error	LRT[a]	
						p-value	q-value
Meconium	Bifidobacterium adolescentis	Vocabulary	Interaction	-0.044	0.039		
Meconium	Bifidobacterium adolescentis	WISC sum	Caffeine	-0.443	0.764	0.49	0.969
Meconium	Bifidobacterium adolescentis	WISC sum	Species	0.202	0.746		
Meconium	Bifidobacterium adolescentis	WISC sum	Interaction	-0.056	0.089		
Meconium	Bifidobacterium animalis	Block Design	Caffeine	-0.245	0.221	0.301	0.949
Meconium	Bifidobacterium animalis	Block Design	Species	0.917	0.63		
Meconium	Bifidobacterium animalis	Block Design	Interaction	-0.064	0.067		
Meconium	Bifidobacterium animalis	Coding	Caffeine	0.11	0.207	0.82	0.969
Meconium	Bifidobacterium animalis	Coding	Species	0.203	0.592		
Meconium	Bifidobacterium animalis	Coding	Interaction	-0.013	0.063		
Meconium	Bifidobacterium animalis	Digit span	Caffeine	-0.363	0.173	0.384	0.969
Meconium	Bifidobacterium animalis	Digit span	Species	-0.398	0.494		
Meconium	Bifidobacterium animalis	Digit span	Interaction	0.042	0.053		
Meconium	Bifidobacterium animalis	Information	Caffeine	-0.141	0.177	0.763	0.969
Meconium	Bifidobacterium animalis	Information	Species	-0.07	0.506		
Meconium	Bifidobacterium animalis	Information	Interaction	0.015	0.054		
Meconium	Bifidobacterium animalis	QTAC	Caffeine	-0.691	0.626	0.548	0.969
Meconium	Bifidobacterium animalis	QTAC	Species	1.149	1.787		
Meconium	Bifidobacterium animalis	QTAC	Interaction	-0.105	0.191		
Meconium	Bifidobacterium animalis	Vocabulary	Caffeine	-0.122	0.265	0.661	0.969
Meconium	Bifidobacterium animalis	Vocabulary	Species	0.495	0.755		
Meconium	Bifidobacterium animalis	Vocabulary	Interaction	-0.032	0.081		
Meconium	Bifidobacterium animalis	WISC sum	Caffeine	-0.76	0.588	0.749	0.969
Meconium	Bifidobacterium animalis	WISC sum	Species	1.147	1.676		
Meconium	Bifidobacterium animalis	WISC sum	Interaction	-0.052	0.179		
Meconium	Bifidobacterium bifidum	Block Design	Caffeine	-0.298	0.244	0.993	0.993
Meconium	Bifidobacterium bifidum	Block Design	Species	0.02	0.516		
Meconium	Bifidobacterium bifidum	Block Design	Interaction	0	0.055		
Meconium	Bifidobacterium bifidum	Coding	Caffeine	0.175	0.208	0.734	0.969
Meconium	Bifidobacterium bifidum	Coding	Species	-0.02	0.44		
Meconium	Bifidobacterium bifidum	Coding	Interaction	-0.014	0.046		
Meconium	Bifidobacterium bifidum	Digit span	Caffeine	-0.304	0.18	0.924	0.978
Meconium	Bifidobacterium bifidum	Digit span	Species	-0.093	0.381		
Meconium	Bifidobacterium bifidum	Digit span	Interaction	0.004	0.04		
Meconium	Bifidobacterium bifidum	Information	Caffeine	-0.134	0.176	0.406	0.969
Meconium	Bifidobacterium bifidum	Information	Species	-0.415	0.373		

Exposure Window	Species	Outcome	Variable	Effect Estimate[a]	Std. Error	LRT[a]	
						p-value	q-value
Meconium	Bifidobacterium bifidum	Information	Interaction	0.03	0.039		
Meconium	Bifidobacterium bifidum	QTAC	Caffeine	-0.8	0.651	0.868	0.978
Meconium	Bifidobacterium bifidum	QTAC	Species	0.359	1.38		
Meconium	Bifidobacterium bifidum	QTAC	Interaction	-0.022	0.146		
Meconium	Bifidobacterium bifidum	Vocabulary	Caffeine	-0.228	0.272	0.246	0.916
Meconium	Bifidobacterium bifidum	Vocabulary	Species	-0.721	0.575		
Meconium	Bifidobacterium bifidum	Vocabulary	Interaction	0.065	0.061		
Meconium	Bifidobacterium bifidum	WISC sum	Caffeine	-0.791	0.602	0.495	0.969
Meconium	Bifidobacterium bifidum	WISC sum	Species	-1.229	1.277		
Meconium	Bifidobacterium bifidum	WISC sum	Interaction	0.084	0.135		
Meconium	Bifidobacterium longum	Block Design	Caffeine	-0.202	0.353	0.706	0.969
Meconium	Bifidobacterium longum	Block Design	Species	0.07	0.24		
Meconium	Bifidobacterium longum	Block Design	Interaction	-0.01	0.03		
Meconium	Bifidobacterium longum	Coding	Caffeine	0.123	0.314	0.931	0.978
Meconium	Bifidobacterium longum	Coding	Species	0.019	0.213		
Meconium	Bifidobacterium longum	Coding	Interaction	-0.002	0.027		
Meconium	Bifidobacterium longum	Digit span	Caffeine	-0.457	0.259	0.519	0.969
Meconium	Bifidobacterium longum	Digit span	Species	-0.14	0.176		
Meconium	Bifidobacterium longum	Digit span	Interaction	0.013	0.022		
Meconium	Bifidobacterium longum	Information	Caffeine	-0.225	0.267	0.584	0.969
Meconium	Bifidobacterium longum	Information	Species	-0.092	0.181		
Meconium	Bifidobacterium longum	Information	Interaction	0.011	0.023		
Meconium	Bifidobacterium longum	QTAC	Caffeine	-0.254	0.91	0.534	0.969
Meconium	Bifidobacterium longum	QTAC	Species	0.584	0.618		
Meconium	Bifidobacterium longum	QTAC	Interaction	-0.044	0.077		
Meconium	Bifidobacterium longum	Vocabulary	Caffeine	-0.2	0.403	0.784	0.969
Meconium	Bifidobacterium longum	Vocabulary	Species	-0.031	0.273		
Meconium	Bifidobacterium longum	Vocabulary	Interaction	0.009	0.034		
Meconium	Bifidobacterium longum	WISC sum	Caffeine	-0.961	0.912	0.773	0.969
Meconium	Bifidobacterium longum	WISC sum	Species	-0.174	0.619		
Meconium	Bifidobacterium longum	WISC sum	Interaction	0.02	0.077		
Meconium	Bifidobacterium pseudocatenulatum	Block Design	Caffeine	-0.291	0.26	0.957	0.978
Meconium	Bifidobacterium pseudocatenulatum	Block Design	Species	-0.085	0.844		
Meconium	Bifidobacterium pseudocatenulatum	Block Design	Interaction	0.005	0.094		
Meconium	Bifidobacterium pseudocatenulatum	Coding	Caffeine	0.237	0.225	0.186	0.916
Meconium	Bifidobacterium pseudocatenulatum	Coding	Species	0.962	0.731		

Exposure Window	Species	Outcome	Variable	Effect Estimate[a]	Std. Error	LRT[a]	
						p-value	q-value
Meconium	Bifidobacterium pseudocatenulatum	Coding	Interaction	-0.099	0.081		
Meconium	Bifidobacterium pseudocatenulatum	Digit span	Caffeine	-0.343	0.193	0.732	0.969
Meconium	Bifidobacterium pseudocatenulatum	Digit span	Species	-0.226	0.627		
Meconium	Bifidobacterium pseudocatenulatum	Digit span	Interaction	0.022	0.07		
Meconium	Bifidobacterium pseudocatenulatum	Information	Caffeine	-0.132	0.197	0.888	0.978
Meconium	Bifidobacterium pseudocatenulatum	Information	Species	-0.085	0.641		
Meconium	Bifidobacterium pseudocatenulatum	Information	Interaction	0.009	0.071		
Meconium	Bifidobacterium pseudocatenulatum	QTAC	Caffeine	-0.43	0.664	0.182	0.916
Meconium	Bifidobacterium pseudocatenulatum	QTAC	Species	3.103	2.156		
Meconium	Bifidobacterium pseudocatenulatum	QTAC	Interaction	-0.294	0.239		
Meconium	Bifidobacterium pseudocatenulatum	Vocabulary	Caffeine	-0.077	0.297	0.608	0.969
Meconium	Bifidobacterium pseudocatenulatum	Vocabulary	Species	0.508	0.963		
Meconium	Bifidobacterium pseudocatenulatum	Vocabulary	Interaction	-0.05	0.107		
Meconium	Bifidobacterium pseudocatenulatum	WISC sum	Caffeine	-0.606	0.67	0.608	0.969
Meconium	Bifidobacterium pseudocatenulatum	WISC sum	Species	1.074	2.177		
Meconium	Bifidobacterium pseudocatenulatum	WISC sum	Interaction	-0.113	0.241		
Meconium	Blautia obeum	Block Design	Caffeine	-0.287	0.304	0.971	0.983
Meconium	Blautia obeum	Block Design	Species	-0.068	0.793		
Meconium	Blautia obeum	Block Design	Interaction	-0.004	0.111		
Meconium	Blautia obeum	Coding	Caffeine	0.359	0.258	0.131	0.916
Meconium	Blautia obeum	Coding	Species	1.106	0.674		
Meconium	Blautia obeum	Coding	Interaction	-0.131	0.094		
Meconium	Blautia obeum	Digit span	Caffeine	-0.409	0.215	0.46	0.969
Meconium	Blautia obeum	Digit span	Species	-0.166	0.562		
Meconium	Blautia obeum	Digit span	Interaction	0.053	0.078		
Meconium	Blautia obeum	Information	Caffeine	-0.03	0.228	0.563	0.969
Meconium	Blautia obeum	Information	Species	0.407	0.597		
Meconium	Blautia obeum	Information	Interaction	-0.044	0.083		
Meconium	Blautia obeum	QTAC	Caffeine	-0.248	0.804	0.276	0.938
Meconium	Blautia obeum	QTAC	Species	1.783	2.1		
Meconium	Blautia obeum	QTAC	Interaction	-0.293	0.293		
Meconium	Blautia obeum	Vocabulary	Caffeine	-0.035	0.347	0.638	0.969
Meconium	Blautia obeum	Vocabulary	Species	0.478	0.907		
Meconium	Blautia obeum	Vocabulary	Interaction	-0.054	0.126		
Meconium	Blautia obeum	WISC sum	Caffeine	-0.402	0.768	0.481	0.969
Meconium	Blautia obeum	WISC sum	Species	1.758	2.005		

Exposure Window	Species	Outcome	Variable	Effect Estimate[a]	Std. Error	LRT[a]	
						p-value	q-value
Meconium	Blautia obeum	WISC sum	Interaction	-0.181	0.28		
Meconium	Blautia wexlerae	Block Design	Caffeine	-0.238	0.276	0.734	0.969
Meconium	Blautia wexlerae	Block Design	Species	0.265	0.886		
Meconium	Blautia wexlerae	Block Design	Interaction	-0.028	0.091		
Meconium	Blautia wexlerae	Coding	Caffeine	0.176	0.24	0.544	0.969
Meconium	Blautia wexlerae	Coding	Species	0.217	0.771		
Meconium	Blautia wexlerae	Coding	Interaction	-0.044	0.079		
Meconium	Blautia wexlerae	Digit span	Caffeine	-0.501	0.2	0.105	0.916
Meconium	Blautia wexlerae	Digit span	Species	-1.015	0.64		
Meconium	Blautia wexlerae	Digit span	Interaction	0.098	0.065		
Meconium	Blautia wexlerae	Information	Caffeine	-0.077	0.197	0.782	0.969
Meconium	Blautia wexlerae	Information	Species	0.477	0.632		
Meconium	Blautia wexlerae	Information	Interaction	-0.016	0.065		
Meconium	Blautia wexlerae	QTAC	Caffeine	-0.752	0.739	0.893	0.978
Meconium	Blautia wexlerae	QTAC	Species	-0.042	2.369		
Meconium	Blautia wexlerae	QTAC	Interaction	-0.03	0.242		
Meconium	Blautia wexlerae	Vocabulary	Caffeine	0.063	0.302	0.254	0.93
Meconium	Blautia wexlerae	Vocabulary	Species	1.357	0.969		
Meconium	Blautia wexlerae	Vocabulary	Interaction	-0.104	0.099		
Meconium	Blautia wexlerae	WISC sum	Caffeine	-0.577	0.707	0.658	0.969
Meconium	Blautia wexlerae	WISC sum	Species	1.301	2.267		
Meconium	Blautia wexlerae	WISC sum	Interaction	-0.094	0.232		
Meconium	Collinsella aerofaciens	Block Design	Caffeine	-0.368	0.35	0.773	0.969
Meconium	Collinsella aerofaciens	Block Design	Species	-0.073	0.317		
Meconium	Collinsella aerofaciens	Block Design	Interaction	0.009	0.036		
Meconium	Collinsella aerofaciens	Coding	Caffeine	-0.116	0.307	0.274	0.938
Meconium	Collinsella aerofaciens	Coding	Species	-0.298	0.277		
Meconium	Collinsella aerofaciens	Coding	Interaction	0.031	0.031		
Meconium	Collinsella aerofaciens	Digit span	Caffeine	-0.373	0.26	0.724	0.969
Meconium	Collinsella aerofaciens	Digit span	Species	-0.084	0.236		
Meconium	Collinsella aerofaciens	Digit span	Interaction	0.009	0.026		
Meconium	Collinsella aerofaciens	Information	Caffeine	0.194	0.257	0.077	0.916
Meconium	Collinsella aerofaciens	Information	Species	0.39	0.233		
Meconium	Collinsella aerofaciens	Information	Interaction	-0.043	0.026		
Meconium	Collinsella aerofaciens	QTAC	Caffeine	-1.222	0.937	0.539	0.969
Meconium	Collinsella aerofaciens	QTAC	Species	-0.438	0.848		

Exposure Window	Species	Outcome	Variable	Effect Estimate[a]	Std. Error	LRT[a]	
						p-value	q-value
Meconium	Collinsella aerofaciens	QTAC	Interaction	0.054	0.095		
Meconium	Collinsella aerofaciens	Vocabulary	Caffeine	-0.128	0.401	0.941	0.978
Meconium	Collinsella aerofaciens	Vocabulary	Species	0.032	0.363		
Meconium	Collinsella aerofaciens	Vocabulary	Interaction	-0.003	0.041		
Meconium	Collinsella aerofaciens	WISC sum	Caffeine	-0.79	0.905	0.967	0.983
Meconium	Collinsella aerofaciens	WISC sum	Species	-0.033	0.819		
Meconium	Collinsella aerofaciens	WISC sum	Interaction	0.004	0.092		
Meconium	Coprococcus catus	Block Design	Caffeine	-0.632	0.421	0.28	0.938
Meconium	Coprococcus catus	Block Design	Species	-3.803	3.676		
Meconium	Coprococcus catus	Block Design	Interaction	0.446	0.449		
Meconium	Coprococcus catus	Coding	Caffeine	0.3	0.377	0.493	0.969
Meconium	Coprococcus catus	Coding	Species	2.081	3.287		
Meconium	Coprococcus catus	Coding	Interaction	-0.252	0.401		
Meconium	Coprococcus catus	Digit span	Caffeine	-0.678	0.31	0.132	0.916
Meconium	Coprococcus catus	Digit span	Species	-3.759	2.706		
Meconium	Coprococcus catus	Digit span	Interaction	0.46	0.33		
Meconium	Coprococcus catus	Information	Caffeine	-0.619	0.31	0.042	0.777
Meconium	Coprococcus catus	Information	Species	-5.014	2.706		
Meconium	Coprococcus catus	Information	Interaction	0.626	0.33		
Meconium	Coprococcus catus	QTAC	Caffeine	-2.175	1.035	0.196	0.916
Meconium	Coprococcus catus	QTAC	Species	-5.278	9.032		
Meconium	Coprococcus catus	QTAC	Interaction	1.316	1.102		
Meconium	Coprococcus catus	Vocabulary	Caffeine	-0.966	0.465	0.028	0.659
Meconium	Coprococcus catus	Vocabulary	Species	-7.966	4.056		
Meconium	Coprococcus catus	Vocabulary	Interaction	1.017	0.495		
Meconium	Coprococcus catus	WISC sum	Caffeine	-2.595	1.049	0.028	0.659
Meconium	Coprococcus catus	WISC sum	Species	-18.462	9.151		
Meconium	Coprococcus catus	WISC sum	Interaction	2.297	1.117		
Meconium	Coprococcus comes	Block Design	Caffeine	-0.81	0.342	0.039	0.777
Meconium	Coprococcus comes	Block Design	Species	-2.693	1.437		
Meconium	Coprococcus comes	Block Design	Interaction	0.33	0.171		
Meconium	Coprococcus comes	Coding	Caffeine	0.105	0.314	0.936	0.978
Meconium	Coprococcus comes	Coding	Species	0.353	1.317		
Meconium	Coprococcus comes	Coding	Interaction	-0.012	0.157		
Meconium	Coprococcus comes	Digit span	Caffeine	-0.631	0.256	0.104	0.916
Meconium	Coprococcus comes	Digit span	Species	-1.368	1.075		

Exposure Window	Species	Outcome	Variable	Effect Estimate[a]	Std. Error	LRT[a]	
						p-value	q-value
Meconium	Coprococcus comes	Digit span	Interaction	0.193	0.128		
Meconium	Coprococcus comes	Information	Caffeine	-0.293	0.268	0.362	0.969
Meconium	Coprococcus comes	Information	Species	-0.957	1.127		
Meconium	Coprococcus comes	Information	Interaction	0.113	0.135		
Meconium	Coprococcus comes	QTAC	Caffeine	-2.346	0.868	0.023	0.659
Meconium	Coprococcus comes	QTAC	Species	-5.987	3.644		
Meconium	Coprococcus comes	QTAC	Interaction	0.927	0.435		
Meconium	Coprococcus comes	Vocabulary	Caffeine	-0.568	0.396	0.159	0.916
Meconium	Coprococcus comes	Vocabulary	Species	-1.783	1.665		
Meconium	Coprococcus comes	Vocabulary	Interaction	0.258	0.199		
Meconium	Coprococcus comes	WISC sum	Caffeine	-2.198	0.869	0.03	0.659
Meconium	Coprococcus comes	WISC sum	Species	-6.449	3.649		
Meconium	Coprococcus comes	WISC sum	Interaction	0.883	0.435		
Meconium	Coprococcus eutactus	Block Design	Caffeine	-0.458	0.239	0.077	0.916
Meconium	Coprococcus eutactus	Block Design	Species	-1.632	0.969		
Meconium	Coprococcus eutactus	Block Design	Interaction	0.176	0.107		
Meconium	Coprococcus eutactus	Coding	Caffeine	0.072	0.217	0.607	0.969
Meconium	Coprococcus eutactus	Coding	Species	-0.567	0.88		
Meconium	Coprococcus eutactus	Coding	Interaction	0.046	0.097		
Meconium	Coprococcus eutactus	Digit span	Caffeine	-0.411	0.18	0.246	0.916
Meconium	Coprococcus eutactus	Digit span	Species	-0.659	0.732		
Meconium	Coprococcus eutactus	Digit span	Interaction	0.086	0.081		
Meconium	Coprococcus eutactus	Information	Caffeine	-0.126	0.187	0.866	0.978
Meconium	Coprococcus eutactus	Information	Species	-0.173	0.758		
Meconium	Coprococcus eutactus	Information	Interaction	0.013	0.084		
Meconium	Coprococcus eutactus	QTAC	Caffeine	-0.769	0.66	0.987	0.991
Meconium	Coprococcus eutactus	QTAC	Species	-0.371	2.68		
Meconium	Coprococcus eutactus	QTAC	Interaction	0.004	0.296		
Meconium	Coprococcus eutactus	Vocabulary	Caffeine	-0.096	0.282	0.771	0.969
Meconium	Coprococcus eutactus	Vocabulary	Species	0.163	1.143		
Meconium	Coprococcus eutactus	Vocabulary	Interaction	-0.034	0.126		
Meconium	Coprococcus eutactus	WISC sum	Caffeine	-1.019	0.627	0.267	0.938
Meconium	Coprococcus eutactus	WISC sum	Species	-2.867	2.544		
Meconium	Coprococcus eutactus	WISC sum	Interaction	0.287	0.281		
Meconium	Dorea formicigenerans	Block Design	Caffeine	-0.28	0.392	0.94	0.978
Meconium	Dorea formicigenerans	Block Design	Species	0.797	2.683		

Exposure Window	Species	Outcome	Variable	Effect Estimate[a]	Std. Error	LRT[a]	
						p-value	q-value
Meconium	Dorea formicigenerans	Block Design	Interaction	-0.022	0.323		
Meconium	Dorea formicigenerans	Coding	Caffeine	-0.052	0.344	0.522	0.969
Meconium	Dorea formicigenerans	Coding	Species	-2.055	2.355		
Meconium	Dorea formicigenerans	Coding	Interaction	0.167	0.284		
Meconium	Dorea formicigenerans	Digit span	Caffeine	-0.597	0.271	0.144	0.916
Meconium	Dorea formicigenerans	Digit span	Species	-3.602	1.856		
Meconium	Dorea formicigenerans	Digit span	Interaction	0.302	0.224		
Meconium	Dorea formicigenerans	Information	Caffeine	-0.775	0.273	0.002	0.593
Meconium	Dorea formicigenerans	Information	Species	-5.189	1.868		
Meconium	Dorea formicigenerans	Information	Interaction	0.653	0.225		
Meconium	Dorea formicigenerans	QTAC	Caffeine	-0.746	1.06	0.952	0.978
Meconium	Dorea formicigenerans	QTAC	Species	0.342	7.259		
Meconium	Dorea formicigenerans	QTAC	Interaction	-0.048	0.875		
Meconium	Dorea formicigenerans	Vocabulary	Caffeine	-0.81	0.434	0.048	0.814
Meconium	Dorea formicigenerans	Vocabulary	Species	-5.166	2.975		
Meconium	Dorea formicigenerans	Vocabulary	Interaction	0.662	0.358		
Meconium	Dorea formicigenerans	WISC sum	Caffeine	-2.515	0.958	0.018	0.659
Meconium	Dorea formicigenerans	WISC sum	Species	-15.215	6.565		
Meconium	Dorea formicigenerans	WISC sum	Interaction	1.761	0.791		
Meconium	Dorea longicatena	Block Design	Caffeine	-0.681	0.409	0.216	0.916
Meconium	Dorea longicatena	Block Design	Species	-1.164	1.052		
Meconium	Dorea longicatena	Block Design	Interaction	0.14	0.123		
Meconium	Dorea longicatena	Coding	Caffeine	0.345	0.364	0.487	0.969
Meconium	Dorea longicatena	Coding	Species	0.41	0.935		
Meconium	Dorea longicatena	Coding	Interaction	-0.07	0.109		
Meconium	Dorea longicatena	Digit span	Caffeine	-0.166	0.307	0.602	0.969
Meconium	Dorea longicatena	Digit span	Species	0.276	0.79		
Meconium	Dorea longicatena	Digit span	Interaction	-0.044	0.092		
Meconium	Dorea longicatena	Information	Caffeine	-0.298	0.306	0.663	0.969
Meconium	Dorea longicatena	Information	Species	-0.042	0.786		
Meconium	Dorea longicatena	Information	Interaction	0.037	0.092		
Meconium	Dorea longicatena	QTAC	Caffeine	-1.101	1.113	0.696	0.969
Meconium	Dorea longicatena	QTAC	Species	-1.09	2.862		
Meconium	Dorea longicatena	QTAC	Interaction	0.12	0.335		
Meconium	Dorea longicatena	Vocabulary	Caffeine	-0.674	0.445	0.28	0.938
Meconium	Dorea longicatena	Vocabulary	Species	-0.555	1.143		

Exposure Window	Species	Outcome	Variable	Effect Estimate[a]	Std. Error	LRT[a]	
						p-value	q-value
Meconium	Dorea longicatena	Vocabulary	Interaction	0.133	0.134		
Meconium	Dorea longicatena	WISC sum	Caffeine	-1.474	1.056	0.5	0.969
Meconium	Dorea longicatena	WISC sum	Species	-1.075	2.715		
Meconium	Dorea longicatena	WISC sum	Interaction	0.196	0.318		
Meconium	Eubacterium hallii	Block Design	Caffeine	0.142	0.41	0.186	0.916
Meconium	Eubacterium hallii	Block Design	Species	1.681	1.338		
Meconium	Eubacterium hallii	Block Design	Interaction	-0.201	0.165		
Meconium	Eubacterium hallii	Coding	Caffeine	0.139	0.364	0.822	0.969
Meconium	Eubacterium hallii	Coding	Species	-0.097	1.188		
Meconium	Eubacterium hallii	Coding	Interaction	-0.03	0.146		
Meconium	Eubacterium hallii	Digit span	Caffeine	-0.211	0.307	0.718	0.969
Meconium	Eubacterium hallii	Digit span	Species	0.56	1		
Meconium	Eubacterium hallii	Digit span	Interaction	-0.041	0.123		
Meconium	Eubacterium hallii	Information	Caffeine	0.256	0.308	0.134	0.916
Meconium	Eubacterium hallii	Information	Species	1.508	1.005		
Meconium	Eubacterium hallii	Information	Interaction	-0.172	0.124		
Meconium	Eubacterium hallii	QTAC	Caffeine	-2.717	1.064	0.023	0.659
Meconium	Eubacterium hallii	QTAC	Species	-7.167	3.471		
Meconium	Eubacterium hallii	QTAC	Interaction	0.911	0.428		
Meconium	Eubacterium hallii	Vocabulary	Caffeine	-0.23	0.47	0.742	0.969
Meconium	Eubacterium hallii	Vocabulary	Species	-0.034	1.535		
Meconium	Eubacterium hallii	Vocabulary	Interaction	0.057	0.189		
Meconium	Eubacterium hallii	WISC sum	Caffeine	0.096	1.062	0.324	0.967
Meconium	Eubacterium hallii	WISC sum	Species	3.619	3.463		
Meconium	Eubacterium hallii	WISC sum	Interaction	-0.387	0.427		
Meconium	Eubacterium rectale	Block Design	Caffeine	-0.114	0.233	0.063	0.916
Meconium	Eubacterium rectale	Block Design	Species	0.43	0.293		
Meconium	Eubacterium rectale	Block Design	Interaction	-0.056	0.032		
Meconium	Eubacterium rectale	Coding	Caffeine	0.137	0.215	0.683	0.969
Meconium	Eubacterium rectale	Coding	Species	0.076	0.271		
Meconium	Eubacterium rectale	Coding	Interaction	-0.011	0.03		
Meconium	Eubacterium rectale	Digit span	Caffeine	-0.331	0.18	0.851	0.978
Meconium	Eubacterium rectale	Digit span	Species	-0.001	0.226		
Meconium	Eubacterium rectale	Digit span	Interaction	0.004	0.025		
Meconium	Eubacterium rectale	Information	Caffeine	-0.176	0.181	0.392	0.969
Meconium	Eubacterium rectale	Information	Species	-0.232	0.227		

Exposure Window	Species	Outcome	Variable	Effect Estimate[a]	Std. Error	LRT[a]	
						p-value	q-value
Meconium	Eubacterium rectale	Information	Interaction	0.02	0.025		
Meconium	Eubacterium rectale	QTAC	Caffeine	-0.464	0.641	0.196	0.916
Meconium	Eubacterium rectale	QTAC	Species	0.873	0.806		
Meconium	Eubacterium rectale	QTAC	Interaction	-0.106	0.089		
Meconium	Eubacterium rectale	Vocabulary	Caffeine	-0.084	0.269	0.635	0.969
Meconium	Eubacterium rectale	Vocabulary	Species	-0.001	0.338		
Meconium	Eubacterium rectale	Vocabulary	Interaction	-0.016	0.037		
Meconium	Eubacterium rectale	WISC sum	Caffeine	-0.568	0.612	0.446	0.969
Meconium	Eubacterium rectale	WISC sum	Species	0.271	0.77		
Meconium	Eubacterium rectale	WISC sum	Interaction	-0.06	0.085		
Meconium	Eubacterium siraeum	Block Design	Caffeine	-0.278	0.225	0.887	0.978
Meconium	Eubacterium siraeum	Block Design	Species	-0.31	2.15		
Meconium	Eubacterium siraeum	Block Design	Interaction	-0.03	0.232		
Meconium	Eubacterium siraeum	Coding	Caffeine	0.086	0.209	0.817	0.969
Meconium	Eubacterium siraeum	Coding	Species	-0.373	1.99		
Meconium	Eubacterium siraeum	Coding	Interaction	0.046	0.215		
Meconium	Eubacterium siraeum	Digit span	Caffeine	-0.337	0.175	0.715	0.969
Meconium	Eubacterium siraeum	Digit span	Species	-0.561	1.668		
Meconium	Eubacterium siraeum	Digit span	Interaction	0.06	0.18		
Meconium	Eubacterium siraeum	Information	Caffeine	-0.122	0.178	0.954	0.978
Meconium	Eubacterium siraeum	Information	Species	-0.037	1.701		
Meconium	Eubacterium siraeum	Information	Interaction	0.01	0.184		
Meconium	Eubacterium siraeum	QTAC	Caffeine	-0.86	0.629	0.761	0.969
Meconium	Eubacterium siraeum	QTAC	Species	-1.296	6.007		
Meconium	Eubacterium siraeum	QTAC	Interaction	0.181	0.648		
Meconium	Eubacterium siraeum	Vocabulary	Caffeine	-0.083	0.261	0.477	0.969
Meconium	Eubacterium siraeum	Vocabulary	Species	2.133	2.492		
Meconium	Eubacterium siraeum	Vocabulary	Interaction	-0.175	0.269		
Meconium	Eubacterium siraeum	WISC sum	Caffeine	-0.734	0.607	0.874	0.978
Meconium	Eubacterium siraeum	WISC sum	Species	0.852	5.794		
Meconium	Eubacterium siraeum	WISC sum	Interaction	-0.09	0.625		
Meconium	Eubacterium sp CAG 180	Block Design	Caffeine	-0.352	0.224	0.506	0.969
Meconium	Eubacterium sp CAG 180	Block Design	Species	-1.621	1.893		
Meconium	Eubacterium sp CAG 180	Block Design	Interaction	0.143	0.234		
Meconium	Eubacterium sp CAG 180	Coding	Caffeine	0.083	0.205	0.836	0.971
Meconium	Eubacterium sp CAG 180	Coding	Species	-0.477	1.73		

Exposure Window	Species	Outcome	Variable	Effect Estimate[a]	Std. Error	LRT[a]	
						p-value	q-value
Meconium	Eubacterium sp CAG 180	Coding	Interaction	0.041	0.214		
Meconium	Eubacterium sp CAG 180	Digit span	Caffeine	-0.315	0.169	0.827	0.969
Meconium	Eubacterium sp CAG 180	Digit span	Species	0.026	1.424		
Meconium	Eubacterium sp CAG 180	Digit span	Interaction	0.035	0.176		
Meconium	Eubacterium sp CAG 180	Information	Caffeine	-0.027	0.157	0.146	0.916
Meconium	Eubacterium sp CAG 180	Information	Species	2.379	1.327		
Meconium	Eubacterium sp CAG 180	Information	Interaction	-0.221	0.164		
Meconium	Eubacterium sp CAG 180	QTAC	Caffeine	-0.992	0.616	0.297	0.949
Meconium	Eubacterium sp CAG 180	QTAC	Species	-5.063	5.194		
Meconium	Eubacterium sp CAG 180	QTAC	Interaction	0.617	0.643		
Meconium	Eubacterium sp CAG 180	Vocabulary	Caffeine	-0.115	0.259	0.909	0.978
Meconium	Eubacterium sp CAG 180	Vocabulary	Species	0.73	2.186		
Meconium	Eubacterium sp CAG 180	Vocabulary	Interaction	-0.028	0.271		
Meconium	Eubacterium sp CAG 180	WISC sum	Caffeine	-0.727	0.592	0.957	0.978
Meconium	Eubacterium sp CAG 180	WISC sum	Species	1.037	4.993		
Meconium	Eubacterium sp CAG 180	WISC sum	Interaction	-0.03	0.619		
Meconium	Faecalibacterium prausnitzii	Block Design	Caffeine	0.004	0.336	0.211	0.916
Meconium	Faecalibacterium prausnitzii	Block Design	Species	0.365	0.345		
Meconium	Faecalibacterium prausnitzii	Block Design	Interaction	-0.051	0.044		
Meconium	Faecalibacterium prausnitzii	Coding	Caffeine	-0.085	0.294	0.334	0.969
Meconium	Faecalibacterium prausnitzii	Coding	Species	-0.161	0.301		
Meconium	Faecalibacterium prausnitzii	Coding	Interaction	0.034	0.039		
Meconium	Faecalibacterium prausnitzii	Digit span	Caffeine	-0.237	0.253	0.676	0.969
Meconium	Faecalibacterium prausnitzii	Digit span	Species	0.13	0.259		
Meconium	Faecalibacterium prausnitzii	Digit span	Interaction	-0.013	0.033		
Meconium	Faecalibacterium prausnitzii	Information	Caffeine	-0.291	0.253	0.361	0.969
Meconium	Faecalibacterium prausnitzii	Information	Species	-0.285	0.259		
Meconium	Faecalibacterium prausnitzii	Information	Interaction	0.028	0.033		
Meconium	Faecalibacterium prausnitzii	QTAC	Caffeine	-0.453	0.913	0.608	0.969
Meconium	Faecalibacterium prausnitzii	QTAC	Species	0.53	0.935		
Meconium	Faecalibacterium prausnitzii	QTAC	Interaction	-0.056	0.12		
Meconium	Faecalibacterium prausnitzii	Vocabulary	Caffeine	-0.131	0.383	0.913	0.978
Meconium	Faecalibacterium prausnitzii	Vocabulary	Species	-0.09	0.392		
Meconium	Faecalibacterium prausnitzii	Vocabulary	Interaction	-0.005	0.05		
Meconium	Faecalibacterium prausnitzii	WISC sum	Caffeine	-0.74	0.881	0.951	0.978
Meconium	Faecalibacterium prausnitzii	WISC sum	Species	-0.042	0.902		

Exposure Window	Species	Outcome	Variable	Effect Estimate[a]	Std. Error	LRT[a]	
						p-value	q-value
Meconium	Faecalibacterium prausnitzii	WISC sum	Interaction	-0.007	0.116		
Meconium	Fusicatenibacter saccharivorans	Block Design	Caffeine	-0.413	0.377	0.654	0.969
Meconium	Fusicatenibacter saccharivorans	Block Design	Species	-0.204	0.491		
Meconium	Fusicatenibacter saccharivorans	Block Design	Interaction	0.022	0.053		
Meconium	Fusicatenibacter saccharivorans	Coding	Caffeine	0.229	0.332	0.681	0.969
Meconium	Fusicatenibacter saccharivorans	Coding	Species	0.094	0.432		
Meconium	Fusicatenibacter saccharivorans	Coding	Interaction	-0.018	0.047		
Meconium	Fusicatenibacter saccharivorans	Digit span	Caffeine	-0.144	0.279	0.432	0.969
Meconium	Fusicatenibacter saccharivorans	Digit span	Species	0.233	0.363		
Meconium	Fusicatenibacter saccharivorans	Digit span	Interaction	-0.028	0.039		
Meconium	Fusicatenibacter saccharivorans	Information	Caffeine	-0.336	0.283	0.303	0.949
Meconium	Fusicatenibacter saccharivorans	Information	Species	-0.34	0.369		
Meconium	Fusicatenibacter saccharivorans	Information	Interaction	0.038	0.04		
Meconium	Fusicatenibacter saccharivorans	QTAC	Caffeine	-1.331	1.005	0.427	0.969
Meconium	Fusicatenibacter saccharivorans	QTAC	Species	-1.073	1.306		
Meconium	Fusicatenibacter saccharivorans	QTAC	Interaction	0.103	0.141		
Meconium	Fusicatenibacter saccharivorans	Vocabulary	Caffeine	-0.594	0.419	0.199	0.916
Meconium	Fusicatenibacter saccharivorans	Vocabulary	Species	-0.506	0.544		
Meconium	Fusicatenibacter saccharivorans	Vocabulary	Interaction	0.07	0.059		
Meconium	Fusicatenibacter saccharivorans	WISC sum	Caffeine	-1.258	0.971	0.505	0.969
Meconium	Fusicatenibacter saccharivorans	WISC sum	Species	-0.723	1.263		
Meconium	Fusicatenibacter saccharivorans	WISC sum	Interaction	0.083	0.136		
Meconium	Methanobrevibacter smithii	Block Design	Caffeine	-0.449	0.263	0.221	0.916
Meconium	Methanobrevibacter smithii	Block Design	Species	-0.851	0.906		
Meconium	Methanobrevibacter smithii	Block Design	Interaction	0.117	0.104		
Meconium	Methanobrevibacter smithii	Coding	Caffeine	0.044	0.236	0.575	0.969
Meconium	Methanobrevibacter smithii	Coding	Species	-0.26	0.812		
Meconium	Methanobrevibacter smithii	Coding	Interaction	0.048	0.093		
Meconium	Methanobrevibacter smithii	Digit span	Caffeine	-0.39	0.198	0.437	0.969
Meconium	Methanobrevibacter smithii	Digit span	Species	-0.396	0.681		
Meconium	Methanobrevibacter smithii	Digit span	Interaction	0.056	0.078		
Meconium	Methanobrevibacter smithii	Information	Caffeine	-0.058	0.203	0.612	0.969
Meconium	Methanobrevibacter smithii	Information	Species	0.396	0.697		
Meconium	Methanobrevibacter smithii	Information	Interaction	-0.037	0.08		
Meconium	Methanobrevibacter smithii	QTAC	Caffeine	-1.312	0.692	0.119	0.916
Meconium	Methanobrevibacter smithii	QTAC	Species	-2.661	2.38		

Exposure Window	Species	Outcome	Variable	Effect Estimate[a]	Std. Error	LRT[a]	
						p-value	q-value
Meconium	Methanobrevibacter smithii	QTAC	Interaction	0.392	0.272		
Meconium	Methanobrevibacter smithii	Vocabulary	Caffeine	-0.079	0.302	0.806	0.969
Meconium	Methanobrevibacter smithii	Vocabulary	Species	0.526	1.038		
Meconium	Methanobrevibacter smithii	Vocabulary	Interaction	-0.027	0.119		
Meconium	Methanobrevibacter smithii	WISC sum	Caffeine	-0.932	0.676	0.521	0.969
Meconium	Methanobrevibacter smithii	WISC sum	Species	-0.586	2.324		
Meconium	Methanobrevibacter smithii	WISC sum	Interaction	0.156	0.266		
Meconium	Prevotella copri	Block Design	Caffeine	-0.349	0.236	0.474	0.969
Meconium	Prevotella copri	Block Design	Species	-1.16	1.835		
Meconium	Prevotella copri	Block Design	Interaction	0.133	0.202		
Meconium	Prevotella copri	Coding	Caffeine	0.031	0.208	0.321	0.967
Meconium	Prevotella copri	Coding	Species	-1.431	1.62		
Meconium	Prevotella copri	Coding	Interaction	0.163	0.178		
Meconium	Prevotella copri	Digit span	Caffeine	-0.28	0.173	0.926	0.978
Meconium	Prevotella copri	Digit span	Species	-0.146	1.346		
Meconium	Prevotella copri	Digit span	Interaction	-0.013	0.148		
Meconium	Prevotella copri	Information	Caffeine	-0.188	0.177	0.187	0.916
Meconium	Prevotella copri	Information	Species	-1.712	1.372		
Meconium	Prevotella copri	Information	Interaction	0.184	0.151		
Meconium	Prevotella copri	QTAC	Caffeine	-0.779	0.63	0.653	0.969
Meconium	Prevotella copri	QTAC	Species	2.643	4.895		
Meconium	Prevotella copri	QTAC	Interaction	-0.222	0.539		
Meconium	Prevotella copri	Vocabulary	Caffeine	-0.043	0.269	0.325	0.967
Meconium	Prevotella copri	Vocabulary	Species	1.75	2.088		
Meconium	Prevotella copri	Vocabulary	Interaction	-0.208	0.23		
Meconium	Prevotella copri	WISC sum	Caffeine	-0.829	0.609	0.588	0.969
Meconium	Prevotella copri	WISC sum	Species	-2.698	4.732		
Meconium	Prevotella copri	WISC sum	Interaction	0.259	0.521		
Meconium	Roseburia faecis	Block Design	Caffeine	-0.289	0.251	0.985	0.991
Meconium	Roseburia faecis	Block Design	Species	-0.07	1.671		
Meconium	Roseburia faecis	Block Design	Interaction	0.003	0.196		
Meconium	Roseburia faecis	Coding	Caffeine	-0.023	0.212	0.136	0.916
Meconium	Roseburia faecis	Coding	Species	-2.109	1.408		
Meconium	Roseburia faecis	Coding	Interaction	0.228	0.165		
Meconium	Roseburia faecis	Digit span	Caffeine	-0.283	0.184	0.604	0.969
Meconium	Roseburia faecis	Digit span	Species	0.679	1.223		

Exposure Window	Species	Outcome	Variable	Effect Estimate[a]	Std. Error	LRT[a]	
						p-value	q-value
Meconium	Roseburia faecis	Digit span	Interaction	-0.068	0.143		
Meconium	Roseburia faecis	Information	Caffeine	-0.163	0.187	0.51	0.969
Meconium	Roseburia faecis	Information	Species	-0.852	1.244		
Meconium	Roseburia faecis	Information	Interaction	0.088	0.146		
Meconium	Roseburia faecis	QTAC	Caffeine	-0.49	0.659	0.362	0.969
Meconium	Roseburia faecis	QTAC	Species	3.29	4.385		
Meconium	Roseburia faecis	QTAC	Interaction	-0.43	0.514		
Meconium	Roseburia faecis	Vocabulary	Caffeine	-0.322	0.278	0.121	0.916
Meconium	Roseburia faecis	Vocabulary	Species	-2.783	1.848		
Meconium	Roseburia faecis	Vocabulary	Interaction	0.311	0.217		
Meconium	Roseburia faecis	WISC sum	Caffeine	-1.079	0.63	0.214	0.916
Meconium	Roseburia faecis	WISC sum	Species	-5.135	4.189		
Meconium	Roseburia faecis	WISC sum	Interaction	0.562	0.491		
Meconium	Roseburia intestinalis	Block Design	Caffeine	-0.242	0.25	0.724	0.969
Meconium	Roseburia intestinalis	Block Design	Species	0.771	1.893		
Meconium	Roseburia intestinalis	Block Design	Interaction	-0.079	0.243		
Meconium	Roseburia intestinalis	Coding	Caffeine	0.137	0.222	0.683	0.969
Meconium	Roseburia intestinalis	Coding	Species	0.551	1.682		
Meconium	Roseburia intestinalis	Coding	Interaction	-0.081	0.216		
Meconium	Roseburia intestinalis	Digit span	Caffeine	-0.3	0.177	0.795	0.969
Meconium	Roseburia intestinalis	Digit span	Species	0.267	1.343		
Meconium	Roseburia intestinalis	Digit span	Interaction	0.041	0.172		
Meconium	Roseburia intestinalis	Information	Caffeine	-0.129	0.188	0.939	0.978
Meconium	Roseburia intestinalis	Information	Species	-0.186	1.424		
Meconium	Roseburia intestinalis	Information	Interaction	-0.013	0.183		
Meconium	Roseburia intestinalis	QTAC	Caffeine	-0.371	0.641	0.077	0.916
Meconium	Roseburia intestinalis	QTAC	Species	6.575	4.857		
Meconium	Roseburia intestinalis	QTAC	Interaction	-1.023	0.623		
Meconium	Roseburia intestinalis	Vocabulary	Caffeine	-0.19	0.287	0.713	0.969
Meconium	Roseburia intestinalis	Vocabulary	Species	-0.742	2.172		
Meconium	Roseburia intestinalis	Vocabulary	Interaction	0.094	0.279		
Meconium	Roseburia intestinalis	WISC sum	Caffeine	-0.724	0.646	0.948	0.978
Meconium	Roseburia intestinalis	WISC sum	Species	0.661	4.896		
Meconium	Roseburia intestinalis	WISC sum	Interaction	-0.037	0.628		
Meconium	Ruminococcus bicirculans	Block Design	Caffeine	-0.341	0.29	0.757	0.969
Meconium	Ruminococcus bicirculans	Block Design	Species	-0.507	1.528		

Exposure Window	Species	Outcome	Variable	Effect Estimate[a]	Std. Error	LRT[a]	
						p-value	q-value
Meconium	Ruminococcus bicirculans	Block Design	Interaction	0.05	0.176		
Meconium	Ruminococcus bicirculans	Coding	Caffeine	0.019	0.257	0.577	0.969
Meconium	Ruminococcus bicirculans	Coding	Species	-0.754	1.353		
Meconium	Ruminococcus bicirculans	Coding	Interaction	0.08	0.156		
Meconium	Ruminococcus bicirculans	Digit span	Caffeine	-0.265	0.215	0.673	0.969
Meconium	Ruminococcus bicirculans	Digit span	Species	0.538	1.133		
Meconium	Ruminococcus bicirculans	Digit span	Interaction	-0.05	0.131		
Meconium	Ruminococcus bicirculans	Information	Caffeine	-0.234	0.218	0.365	0.969
Meconium	Ruminococcus bicirculans	Information	Species	-1.026	1.149		
Meconium	Ruminococcus bicirculans	Information	Interaction	0.11	0.132		
Meconium	Ruminococcus bicirculans	QTAC	Caffeine	-1.042	0.776	0.618	0.969
Meconium	Ruminococcus bicirculans	QTAC	Species	-1.495	4.088		
Meconium	Ruminococcus bicirculans	QTAC	Interaction	0.215	0.471		
Meconium	Ruminococcus bicirculans	Vocabulary	Caffeine	-0.454	0.307	0.072	0.916
Meconium	Ruminococcus bicirculans	Vocabulary	Species	-3.279	1.62		
Meconium	Ruminococcus bicirculans	Vocabulary	Interaction	0.312	0.187		
Meconium	Ruminococcus bicirculans	WISC sum	Caffeine	-1.275	0.729	0.219	0.916
Meconium	Ruminococcus bicirculans	WISC sum	Species	-5.029	3.841		
Meconium	Ruminococcus bicirculans	WISC sum	Interaction	0.502	0.443		
Meconium	Ruminococcus bromii	Block Design	Caffeine	-0.437	0.407	0.626	0.969
Meconium	Ruminococcus bromii	Block Design	Species	-0.075	0.304		
Meconium	Ruminococcus bromii	Block Design	Interaction	0.016	0.037		
Meconium	Ruminococcus bromii	Coding	Caffeine	0.049	0.356	0.821	0.969
Meconium	Ruminococcus bromii	Coding	Species	0.025	0.266		
Meconium	Ruminococcus bromii	Coding	Interaction	0.007	0.032		
Meconium	Ruminococcus bromii	Digit span	Caffeine	-0.481	0.296	0.448	0.969
Meconium	Ruminococcus bromii	Digit span	Species	-0.08	0.221		
Meconium	Ruminococcus bromii	Digit span	Interaction	0.018	0.027		
Meconium	Ruminococcus bromii	Information	Caffeine	-0.198	0.308	0.721	0.969
Meconium	Ruminococcus bromii	Information	Species	-0.028	0.231		
Meconium	Ruminococcus bromii	Information	Interaction	0.009	0.028		
Meconium	Ruminococcus bromii	QTAC	Caffeine	-1.6	1.095	0.347	0.969
Meconium	Ruminococcus bromii	QTAC	Species	-0.726	0.819		
Meconium	Ruminococcus bromii	QTAC	Interaction	0.085	0.098		
Meconium	Ruminococcus bromii	Vocabulary	Caffeine	-0.001	0.466	0.712	0.969
Meconium	Ruminococcus bromii	Vocabulary	Species	0.179	0.349		

Exposure Window	Species	Outcome	Variable	Effect Estimate[a]	Std. Error	LRT[a]	
						p-value	q-value
Meconium	Ruminococcus bromii	Vocabulary	Interaction	-0.014	0.042		
Meconium	Ruminococcus bromii	WISC sum	Caffeine	-1.067	1.011	0.662	0.969
Meconium	Ruminococcus bromii	WISC sum	Species	0.02	0.756		
Meconium	Ruminococcus bromii	WISC sum	Interaction	0.036	0.091		
Meconium	Ruminococcus lactaris	Block Design	Caffeine	-0.355	0.249	0.755	0.969
Meconium	Ruminococcus lactaris	Block Design	Species	-0.089	1.794		
Meconium	Ruminococcus lactaris	Block Design	Interaction	0.056	0.198		
Meconium	Ruminococcus lactaris	Coding	Caffeine	0.125	0.225	0.794	0.969
Meconium	Ruminococcus lactaris	Coding	Species	0.43	1.623		
Meconium	Ruminococcus lactaris	Coding	Interaction	-0.043	0.179		
Meconium	Ruminococcus lactaris	Digit span	Caffeine	-0.211	0.185	0.303	0.949
Meconium	Ruminococcus lactaris	Digit span	Species	1	1.331		
Meconium	Ruminococcus lactaris	Digit span	Interaction	-0.139	0.147		
Meconium	Ruminococcus lactaris	Information	Caffeine	-0.316	0.183	0.022	0.659
Meconium	Ruminococcus lactaris	Information	Species	-2.749	1.316		
Meconium	Ruminococcus lactaris	Information	Interaction	0.312	0.145		
Meconium	Ruminococcus lactaris	QTAC	Caffeine	-0.423	0.672	0.224	0.916
Meconium	Ruminococcus lactaris	QTAC	Species	5.368	4.842		
Meconium	Ruminococcus lactaris	QTAC	Interaction	-0.598	0.534		
Meconium	Ruminococcus lactaris	Vocabulary	Caffeine	-0.382	0.282	0.079	0.916
Meconium	Ruminococcus lactaris	Vocabulary	Species	-3.103	2.028		
Meconium	Ruminococcus lactaris	Vocabulary	Interaction	0.365	0.224		
Meconium	Ruminococcus lactaris	WISC sum	Caffeine	-1.139	0.646	0.243	0.916
Meconium	Ruminococcus lactaris	WISC sum	Species	-4.51	4.649		
Meconium	Ruminococcus lactaris	WISC sum	Interaction	0.551	0.513		
Meconium	Ruminococcus torques	Block Design	Caffeine	-0.127	0.389	0.541	0.969
Meconium	Ruminococcus torques	Block Design	Species	0.686	0.793		
Meconium	Ruminococcus torques	Block Design	Interaction	-0.051	0.091		
Meconium	Ruminococcus torques	Coding	Caffeine	-0.003	0.347	0.653	0.969
Meconium	Ruminococcus torques	Coding	Species	-0.491	0.708		
Meconium	Ruminococcus torques	Coding	Interaction	0.034	0.081		
Meconium	Ruminococcus torques	Digit span	Caffeine	-0.569	0.279	0.205	0.916
Meconium	Ruminococcus torques	Digit span	Species	-0.907	0.568		
Meconium	Ruminococcus torques	Digit span	Interaction	0.076	0.065		
Meconium	Ruminococcus torques	Information	Caffeine	-0.219	0.303	0.649	0.969
Meconium	Ruminococcus torques	Information	Species	-0.314	0.617		

Exposure Window	Species	Outcome	Variable	Effect Estimate[a]	Std. Error	LRT[a]	
						p-value	q-value
Meconium	Ruminococcus torques	Information	Interaction	0.03	0.071		
Meconium	Ruminococcus torques	QTAC	Caffeine	-1.79	1.046	0.236	0.916
Meconium	Ruminococcus torques	QTAC	Species	-1.77	2.132		
Meconium	Ruminococcus torques	QTAC	Interaction	0.268	0.245		
Meconium	Ruminococcus torques	Vocabulary	Caffeine	-0.464	0.455	0.357	0.969
Meconium	Ruminococcus torques	Vocabulary	Species	-0.78	0.928		
Meconium	Ruminococcus torques	Vocabulary	Interaction	0.09	0.107		
Meconium	Ruminococcus torques	WISC sum	Caffeine	-1.382	1.024	0.417	0.969
Meconium	Ruminococcus torques	WISC sum	Species	-1.806	2.086		
Meconium	Ruminococcus torques	WISC sum	Interaction	0.178	0.24		
Meconium	Streptococcus thermophilus	Block Design	Caffeine	-0.537	0.267	0.082	0.916
Meconium	Streptococcus thermophilus	Block Design	Species	-2.355	1.658		
Meconium	Streptococcus thermophilus	Block Design	Interaction	0.317	0.196		
Meconium	Streptococcus thermophilus	Coding	Caffeine	-0.019	0.238	0.465	0.969
Meconium	Streptococcus thermophilus	Coding	Species	-1.401	1.479		
Meconium	Streptococcus thermophilus	Coding	Interaction	0.117	0.175		
Meconium	Streptococcus thermophilus	Digit span	Caffeine	-0.226	0.203	0.505	0.969
Meconium	Streptococcus thermophilus	Digit span	Species	1.011	1.259		
Meconium	Streptococcus thermophilus	Digit span	Interaction	-0.091	0.149		
Meconium	Streptococcus thermophilus	Information	Caffeine	0.084	0.201	0.102	0.916
Meconium	Streptococcus thermophilus	Information	Species	2.154	1.25		
Meconium	Streptococcus thermophilus	Information	Interaction	-0.224	0.148		
Meconium	Streptococcus thermophilus	QTAC	Caffeine	-0.884	0.617	0.817	0.969
Meconium	Streptococcus thermophilus	QTAC	Species	-2.212	3.837		
Meconium	Streptococcus thermophilus	QTAC	Interaction	-0.096	0.454		
Meconium	Streptococcus thermophilus	Vocabulary	Caffeine	-0.078	0.305	0.865	0.978
Meconium	Streptococcus thermophilus	Vocabulary	Species	0.934	1.896		
Meconium	Streptococcus thermophilus	Vocabulary	Interaction	-0.035	0.224		
Meconium	Streptococcus thermophilus	WISC sum	Caffeine	-0.777	0.702	0.86	0.978
Meconium	Streptococcus thermophilus	WISC sum	Species	0.343	4.364		
Meconium	Streptococcus thermophilus	WISC sum	Interaction	0.084	0.516		
Meconium Adj.[c]	Agathobaculum butyriciproducens	Block Design	Caffeine	-0.19	0.259	0.56	0.975
Meconium Adj.[c]	Agathobaculum butyriciproducens	Block Design	Species	0.963	2.747		
Meconium Adj.[c]	Agathobaculum butyriciproducens	Block Design	Interaction	-0.165	0.314		
Meconium Adj.[c]	Agathobaculum butyriciproducens	Coding	Caffeine	-0.045	0.227	0.145	0.844
Meconium Adj.[c]	Agathobaculum butyriciproducens	Coding	Species	-3.431	2.401		

Exposure Window	Species	Outcome	Variable	Effect Estimate[a]	Std. Error	LRT[a]	
						p-value	q-value
Meconium Adj.[c]	Agathobaculum butyriciproducens	Coding	Interaction	0.365	0.274		
Meconium Adj.[c]	Agathobaculum butyriciproducens	Digit span	Caffeine	-0.266	0.194	0.49	0.975
Meconium Adj.[c]	Agathobaculum butyriciproducens	Digit span	Species	1.528	2.056		
Meconium Adj.[c]	Agathobaculum butyriciproducens	Digit span	Interaction	-0.147	0.235		
Meconium Adj.[c]	Agathobaculum butyriciproducens	Information	Caffeine	-0.125	0.188	0.724	0.975
Meconium Adj.[c]	Agathobaculum butyriciproducens	Information	Species	1.17	1.994		
Meconium Adj.[c]	Agathobaculum butyriciproducens	Information	Interaction	-0.073	0.228		
Meconium Adj.[c]	Agathobaculum butyriciproducens	QTAC	Caffeine	-0.974	0.666	0.398	0.971
Meconium Adj.[c]	Agathobaculum butyriciproducens	QTAC	Species	-6.497	7.055		
Meconium Adj.[c]	Agathobaculum butyriciproducens	QTAC	Interaction	0.618	0.806		
Meconium Adj.[c]	Agathobaculum butyriciproducens	Vocabulary	Caffeine	-0.203	0.297	0.826	0.994
Meconium Adj.[c]	Agathobaculum butyriciproducens	Vocabulary	Species	-0.237	3.141		
Meconium Adj.[c]	Agathobaculum butyriciproducens	Vocabulary	Interaction	0.071	0.359		
Meconium Adj.[c]	Agathobaculum butyriciproducens	WISC sum	Caffeine	-0.828	0.67	0.944	0.994
Meconium Adj.[c]	Agathobaculum butyriciproducens	WISC sum	Species	-0.007	7.095		
Meconium Adj.[c]	Agathobaculum butyriciproducens	WISC sum	Interaction	0.051	0.81		
Meconium Adj.[c]	Akkermansia muciniphila	Block Design	Caffeine	-0.347	0.259	0.651	0.975
Meconium Adj.[c]	Akkermansia muciniphila	Block Design	Species	-0.166	0.51		
Meconium Adj.[c]	Akkermansia muciniphila	Block Design	Interaction	0.022	0.055		
Meconium Adj.[c]	Akkermansia muciniphila	Coding	Caffeine	0	0.227	0.354	0.968
Meconium Adj.[c]	Akkermansia muciniphila	Coding	Species	-0.316	0.446		
Meconium Adj.[c]	Akkermansia muciniphila	Coding	Interaction	0.04	0.048		
Meconium Adj.[c]	Akkermansia muciniphila	Digit span	Caffeine	-0.389	0.19	0.418	0.971
Meconium Adj.[c]	Akkermansia muciniphila	Digit span	Species	-0.37	0.373		
Meconium Adj.[c]	Akkermansia muciniphila	Digit span	Interaction	0.03	0.04		
Meconium Adj.[c]	Akkermansia muciniphila	Information	Caffeine	-0.2	0.191	0.392	0.971
Meconium Adj.[c]	Akkermansia muciniphila	Information	Species	-0.269	0.376		
Meconium Adj.[c]	Akkermansia muciniphila	Information	Interaction	0.031	0.04		
Meconium Adj.[c]	Akkermansia muciniphila	QTAC	Caffeine	-0.795	0.629	0.981	0.994
Meconium Adj.[c]	Akkermansia muciniphila	QTAC	Species	-0.843	1.236		
Meconium Adj.[c]	Akkermansia muciniphila	QTAC	Interaction	0.003	0.133		
Meconium Adj.[c]	Akkermansia muciniphila	Vocabulary	Caffeine	-0.213	0.293	0.637	0.975
Meconium Adj.[c]	Akkermansia muciniphila	Vocabulary	Species	-0.302	0.576		
Meconium Adj.[c]	Akkermansia muciniphila	Vocabulary	Interaction	0.026	0.062		
Meconium Adj.[c]	Akkermansia muciniphila	WISC sum	Caffeine	-1.149	0.65	0.23	0.844
Meconium Adj.[c]	Akkermansia muciniphila	WISC sum	Species	-1.423	1.278		

Exposure Window	Species	Outcome	Variable	Effect Estimate[a]	Std. Error	LRT[a]	
						p-value	q-value
Meconium Adj.[c]	Akkermansia muciniphila	WISC sum	Interaction	0.15	0.138		
Meconium Adj.[c]	Alistipes putredinis	Block Design	Caffeine	-0.504	0.266	0.133	0.844
Meconium Adj.[c]	Alistipes putredinis	Block Design	Species	-2.043	1.495		
Meconium Adj.[c]	Alistipes putredinis	Block Design	Interaction	0.227	0.166		
Meconium Adj.[c]	Alistipes putredinis	Coding	Caffeine	0.16	0.232	0.624	0.975
Meconium Adj.[c]	Alistipes putredinis	Coding	Species	1.108	1.304		
Meconium Adj.[c]	Alistipes putredinis	Coding	Interaction	-0.064	0.144		
Meconium Adj.[c]	Alistipes putredinis	Digit span	Caffeine	-0.223	0.198	0.372	0.971
Meconium Adj.[c]	Alistipes putredinis	Digit span	Species	0.555	1.113		
Meconium Adj.[c]	Alistipes putredinis	Digit span	Interaction	-0.1	0.123		
Meconium Adj.[c]	Alistipes putredinis	Information	Caffeine	-0.081	0.192	0.688	0.975
Meconium Adj.[c]	Alistipes putredinis	Information	Species	0.927	1.08		
Meconium Adj.[c]	Alistipes putredinis	Information	Interaction	-0.043	0.12		
Meconium Adj.[c]	Alistipes putredinis	QTAC	Caffeine	-0.273	0.688	0.169	0.844
Meconium Adj.[c]	Alistipes putredinis	QTAC	Species	4.725	3.864		
Meconium Adj.[c]	Alistipes putredinis	QTAC	Interaction	-0.537	0.428		
Meconium Adj.[c]	Alistipes putredinis	Vocabulary	Caffeine	0.047	0.304	0.226	0.844
Meconium Adj.[c]	Alistipes putredinis	Vocabulary	Species	1.958	1.704		
Meconium Adj.[c]	Alistipes putredinis	Vocabulary	Interaction	-0.208	0.189		
Meconium Adj.[c]	Alistipes putredinis	WISC sum	Caffeine	-0.6	0.684	0.625	0.975
Meconium Adj.[c]	Alistipes putredinis	WISC sum	Species	2.506	3.841		
Meconium Adj.[c]	Alistipes putredinis	WISC sum	Interaction	-0.188	0.425		
Meconium Adj.[c]	Anaerostipes hadrus	Block Design	Caffeine	0.066	0.396	0.227	0.844
Meconium Adj.[c]	Anaerostipes hadrus	Block Design	Species	0.545	0.507		
Meconium Adj.[c]	Anaerostipes hadrus	Block Design	Interaction	-0.076	0.069		
Meconium Adj.[c]	Anaerostipes hadrus	Coding	Caffeine	-0.064	0.342	0.319	0.949
Meconium Adj.[c]	Anaerostipes hadrus	Coding	Species	-0.218	0.438		
Meconium Adj.[c]	Anaerostipes hadrus	Coding	Interaction	0.054	0.06		
Meconium Adj.[c]	Anaerostipes hadrus	Digit span	Caffeine	-0.447	0.298	0.61	0.975
Meconium Adj.[c]	Anaerostipes hadrus	Digit span	Species	-0.208	0.381		
Meconium Adj.[c]	Anaerostipes hadrus	Digit span	Interaction	0.024	0.052		
Meconium Adj.[c]	Anaerostipes hadrus	Information	Caffeine	0.151	0.291	0.291	0.924
Meconium Adj.[c]	Anaerostipes hadrus	Information	Species	0.433	0.373		
Meconium Adj.[c]	Anaerostipes hadrus	Information	Interaction	-0.049	0.051		
Meconium Adj.[c]	Anaerostipes hadrus	QTAC	Caffeine	-2.134	0.997	0.059	0.777
Meconium Adj.[c]	Anaerostipes hadrus	QTAC	Species	-2.053	1.277		

Exposure Window	Species	Outcome	Variable	Effect Estimate[a]	Std. Error	LRT[a]	
						p-value	q-value
Meconium Adj.[c]	Anaerostipes hadrus	QTAC	Interaction	0.301	0.173		
Meconium Adj.[c]	Anaerostipes hadrus	Vocabulary	Caffeine	0.419	0.44	0.133	0.844
Meconium Adj.[c]	Anaerostipes hadrus	Vocabulary	Species	0.886	0.563		
Meconium Adj.[c]	Anaerostipes hadrus	Vocabulary	Interaction	-0.105	0.077		
Meconium Adj.[c]	Anaerostipes hadrus	WISC sum	Caffeine	0.125	0.997	0.335	0.963
Meconium Adj.[c]	Anaerostipes hadrus	WISC sum	Species	1.438	1.277		
Meconium Adj.[c]	Anaerostipes hadrus	WISC sum	Interaction	-0.152	0.173		
Meconium Adj.[c]	Asaccharobacter celatus	Block Design	Caffeine	-0.237	0.371	0.782	0.994
Meconium Adj.[c]	Asaccharobacter celatus	Block Design	Species	0.555	3.97		
Meconium Adj.[c]	Asaccharobacter celatus	Block Design	Interaction	-0.117	0.467		
Meconium Adj.[c]	Asaccharobacter celatus	Coding	Caffeine	0.774	0.303	0.003	0.408
Meconium Adj.[c]	Asaccharobacter celatus	Coding	Species	8.913	3.24		
Meconium Adj.[c]	Asaccharobacter celatus	Coding	Interaction	-1.056	0.381		
Meconium Adj.[c]	Asaccharobacter celatus	Digit span	Caffeine	0.002	0.272	0.119	0.844
Meconium Adj.[c]	Asaccharobacter celatus	Digit span	Species	4.233	2.907		
Meconium Adj.[c]	Asaccharobacter celatus	Digit span	Interaction	-0.487	0.342		
Meconium Adj.[c]	Asaccharobacter celatus	Information	Caffeine	0.074	0.275	0.347	0.963
Meconium Adj.[c]	Asaccharobacter celatus	Information	Species	2.674	2.934		
Meconium Adj.[c]	Asaccharobacter celatus	Information	Interaction	-0.295	0.345		
Meconium Adj.[c]	Asaccharobacter celatus	QTAC	Caffeine	-1.604	0.951	0.228	0.844
Meconium Adj.[c]	Asaccharobacter celatus	QTAC	Species	-10.888	10.167		
Meconium Adj.[c]	Asaccharobacter celatus	QTAC	Interaction	1.312	1.195		
Meconium Adj.[c]	Asaccharobacter celatus	Vocabulary	Caffeine	-0.124	0.424	0.959	0.994
Meconium Adj.[c]	Asaccharobacter celatus	Vocabulary	Species	0.364	4.533		
Meconium Adj.[c]	Asaccharobacter celatus	Vocabulary	Interaction	-0.025	0.533		
Meconium Adj.[c]	Asaccharobacter celatus	WISC sum	Caffeine	0.489	0.92	0.063	0.777
Meconium Adj.[c]	Asaccharobacter celatus	WISC sum	Species	16.739	9.83		
Meconium Adj.[c]	Asaccharobacter celatus	WISC sum	Interaction	-1.979	1.156		
Meconium Adj.[c]	Bacteroides vulgatus	Block Design	Caffeine	-0.48	0.254	0.09	0.844
Meconium Adj.[c]	Bacteroides vulgatus	Block Design	Species	-2.963	1.972		
Meconium Adj.[c]	Bacteroides vulgatus	Block Design	Interaction	0.464	0.298		
Meconium Adj.[c]	Bacteroides vulgatus	Coding	Caffeine	0.143	0.223	0.723	0.975
Meconium Adj.[c]	Bacteroides vulgatus	Coding	Species	0.155	1.733		
Meconium Adj.[c]	Bacteroides vulgatus	Coding	Interaction	0.084	0.262		
Meconium Adj.[c]	Bacteroides vulgatus	Digit span	Caffeine	-0.198	0.191	0.184	0.844
Meconium Adj.[c]	Bacteroides vulgatus	Digit span	Species	1.793	1.484		

Exposure Window	Species	Outcome	Variable	Effect Estimate[a]	Std. Error	LRT[a]	
						p-value	q-value
Meconium Adj.[c]	Bacteroides vulgatus	Digit span	Interaction	-0.272	0.225		
Meconium Adj.[c]	Bacteroides vulgatus	Information	Caffeine	-0.099	0.193	0.755	0.992
Meconium Adj.[c]	Bacteroides vulgatus	Information	Species	0.392	1.503		
Meconium Adj.[c]	Bacteroides vulgatus	Information	Interaction	-0.064	0.227		
Meconium Adj.[c]	Bacteroides vulgatus	QTAC	Caffeine	-0.426	0.647	0.708	0.975
Meconium Adj.[c]	Bacteroides vulgatus	QTAC	Species	3.808	5.033		
Meconium Adj.[c]	Bacteroides vulgatus	QTAC	Interaction	-0.258	0.761		
Meconium Adj.[c]	Bacteroides vulgatus	Vocabulary	Caffeine	-0.127	0.295	0.734	0.981
Meconium Adj.[c]	Bacteroides vulgatus	Vocabulary	Species	0.476	2.292		
Meconium Adj.[c]	Bacteroides vulgatus	Vocabulary	Interaction	-0.107	0.347		
Meconium Adj.[c]	Bacteroides vulgatus	WISC sum	Caffeine	-0.762	0.662	0.881	0.994
Meconium Adj.[c]	Bacteroides vulgatus	WISC sum	Species	-0.147	5.147		
Meconium Adj.[c]	Bacteroides vulgatus	WISC sum	Interaction	0.105	0.779		
Meconium Adj.[c]	Bifidobacterium adolescentis	Block Design	Caffeine	-0.291	0.318	0.985	0.994
Meconium Adj.[c]	Bifidobacterium adolescentis	Block Design	Species	-0.039	0.321		
Meconium Adj.[c]	Bifidobacterium adolescentis	Block Design	Interaction	-0.001	0.038		
Meconium Adj.[c]	Bifidobacterium adolescentis	Coding	Caffeine	-0.391	0.258	0.005	0.426
Meconium Adj.[c]	Bifidobacterium adolescentis	Coding	Species	-0.72	0.261		
Meconium Adj.[c]	Bifidobacterium adolescentis	Coding	Interaction	0.08	0.031		
Meconium Adj.[c]	Bifidobacterium adolescentis	Digit span	Caffeine	-0.223	0.237	0.544	0.975
Meconium Adj.[c]	Bifidobacterium adolescentis	Digit span	Species	0.111	0.239		
Meconium Adj.[c]	Bifidobacterium adolescentis	Digit span	Interaction	-0.015	0.028		
Meconium Adj.[c]	Bifidobacterium adolescentis	Information	Caffeine	0.166	0.225	0.05	0.777
Meconium Adj.[c]	Bifidobacterium adolescentis	Information	Species	0.35	0.227		
Meconium Adj.[c]	Bifidobacterium adolescentis	Information	Interaction	-0.048	0.027		
Meconium Adj.[c]	Bifidobacterium adolescentis	QTAC	Caffeine	-1.215	0.806	0.416	0.971
Meconium Adj.[c]	Bifidobacterium adolescentis	QTAC	Species	-0.769	0.815		
Meconium Adj.[c]	Bifidobacterium adolescentis	QTAC	Interaction	0.071	0.096		
Meconium Adj.[c]	Bifidobacterium adolescentis	Vocabulary	Caffeine	0.02	0.342	0.424	0.971
Meconium Adj.[c]	Bifidobacterium adolescentis	Vocabulary	Species	0.099	0.346		
Meconium Adj.[c]	Bifidobacterium adolescentis	Vocabulary	Interaction	-0.03	0.041		
Meconium Adj.[c]	Bifidobacterium adolescentis	WISC sum	Caffeine	-0.72	0.777	0.871	0.994
Meconium Adj.[c]	Bifidobacterium adolescentis	WISC sum	Species	-0.199	0.785		
Meconium Adj.[c]	Bifidobacterium adolescentis	WISC sum	Interaction	-0.014	0.092		
Meconium Adj.[c]	Bifidobacterium animalis	Block Design	Caffeine	-0.238	0.224	0.266	0.884
Meconium Adj.[c]	Bifidobacterium animalis	Block Design	Species	0.975	0.652		

Exposure Window	Species	Outcome	Variable	Effect Estimate[a]	Std. Error	LRT[a]	
						p-value	q-value
Meconium Adj.[c]	Bifidobacterium animalis	Block Design	Interaction	-0.071	0.07		
Meconium Adj.[c]	Bifidobacterium animalis	Coding	Caffeine	0.122	0.209	0.669	0.975
Meconium Adj.[c]	Bifidobacterium animalis	Coding	Species	0.304	0.609		
Meconium Adj.[c]	Bifidobacterium animalis	Coding	Interaction	-0.025	0.065		
Meconium Adj.[c]	Bifidobacterium animalis	Digit span	Caffeine	-0.368	0.176	0.343	0.963
Meconium Adj.[c]	Bifidobacterium animalis	Digit span	Species	-0.441	0.512		
Meconium Adj.[c]	Bifidobacterium animalis	Digit span	Interaction	0.047	0.055		
Meconium Adj.[c]	Bifidobacterium animalis	Information	Caffeine	-0.124	0.176	0.95	0.994
Meconium Adj.[c]	Bifidobacterium animalis	Information	Species	0.079	0.513		
Meconium Adj.[c]	Bifidobacterium animalis	Information	Interaction	-0.003	0.055		
Meconium Adj.[c]	Bifidobacterium animalis	QTAC	Caffeine	-0.774	0.61	0.914	0.994
Meconium Adj.[c]	Bifidobacterium animalis	QTAC	Species	0.433	1.778		
Meconium Adj.[c]	Bifidobacterium animalis	QTAC	Interaction	-0.019	0.191		
Meconium Adj.[c]	Bifidobacterium animalis	Vocabulary	Caffeine	-0.101	0.265	0.472	0.975
Meconium Adj.[c]	Bifidobacterium animalis	Vocabulary	Species	0.675	0.772		
Meconium Adj.[c]	Bifidobacterium animalis	Vocabulary	Interaction	-0.054	0.083		
Meconium Adj.[c]	Bifidobacterium animalis	WISC sum	Caffeine	-0.709	0.586	0.523	0.975
Meconium Adj.[c]	Bifidobacterium animalis	WISC sum	Species	1.592	1.708		
Meconium Adj.[c]	Bifidobacterium animalis	WISC sum	Interaction	-0.106	0.184		
Meconium Adj.[c]	Bifidobacterium bifidum	Block Design	Caffeine	-0.304	0.247	0.957	0.994
Meconium Adj.[c]	Bifidobacterium bifidum	Block Design	Species	-0.002	0.527		
Meconium Adj.[c]	Bifidobacterium bifidum	Block Design	Interaction	0.003	0.056		
Meconium Adj.[c]	Bifidobacterium bifidum	Coding	Caffeine	0.163	0.209	0.822	0.994
Meconium Adj.[c]	Bifidobacterium bifidum	Coding	Species	-0.067	0.446		
Meconium Adj.[c]	Bifidobacterium bifidum	Coding	Interaction	-0.01	0.047		
Meconium Adj.[c]	Bifidobacterium bifidum	Digit span	Caffeine	-0.302	0.182	0.943	0.994
Meconium Adj.[c]	Bifidobacterium bifidum	Digit span	Species	-0.084	0.389		
Meconium Adj.[c]	Bifidobacterium bifidum	Digit span	Interaction	0.003	0.041		
Meconium Adj.[c]	Bifidobacterium bifidum	Information	Caffeine	-0.154	0.173	0.275	0.884
Meconium Adj.[c]	Bifidobacterium bifidum	Information	Species	-0.499	0.368		
Meconium Adj.[c]	Bifidobacterium bifidum	Information	Interaction	0.039	0.039		
Meconium Adj.[c]	Bifidobacterium bifidum	QTAC	Caffeine	-0.713	0.632	0.647	0.975
Meconium Adj.[c]	Bifidobacterium bifidum	QTAC	Species	0.717	1.348		
Meconium Adj.[c]	Bifidobacterium bifidum	QTAC	Interaction	-0.059	0.142		
Meconium Adj.[c]	Bifidobacterium bifidum	Vocabulary	Caffeine	-0.252	0.271	0.179	0.844
Meconium Adj.[c]	Bifidobacterium bifidum	Vocabulary	Species	-0.817	0.577		

Exposure Window	Species	Outcome	Variable	Effect Estimate[a]	Std. Error	LRT[a]	
						p-value	q-value
Meconium Adj.[c]	Bifidobacterium bifidum	Vocabulary	Interaction	0.075	0.061		
Meconium Adj.[c]	Bifidobacterium bifidum	WISC sum	Caffeine	-0.849	0.597	0.371	0.971
Meconium Adj.[c]	Bifidobacterium bifidum	WISC sum	Species	-1.47	1.274		
Meconium Adj.[c]	Bifidobacterium bifidum	WISC sum	Interaction	0.109	0.135		
Meconium Adj.[c]	Bifidobacterium longum	Block Design	Caffeine	-0.184	0.361	0.665	0.975
Meconium Adj.[c]	Bifidobacterium longum	Block Design	Species	0.089	0.25		
Meconium Adj.[c]	Bifidobacterium longum	Block Design	Interaction	-0.012	0.031		
Meconium Adj.[c]	Bifidobacterium longum	Coding	Caffeine	0.162	0.319	0.809	0.994
Meconium Adj.[c]	Bifidobacterium longum	Coding	Species	0.063	0.22		
Meconium Adj.[c]	Bifidobacterium longum	Coding	Interaction	-0.006	0.027		
Meconium Adj.[c]	Bifidobacterium longum	Digit span	Caffeine	-0.483	0.263	0.444	0.975
Meconium Adj.[c]	Bifidobacterium longum	Digit span	Species	-0.169	0.182		
Meconium Adj.[c]	Bifidobacterium longum	Digit span	Interaction	0.016	0.022		
Meconium Adj.[c]	Bifidobacterium longum	Information	Caffeine	-0.169	0.267	0.774	0.994
Meconium Adj.[c]	Bifidobacterium longum	Information	Species	-0.031	0.185		
Meconium Adj.[c]	Bifidobacterium longum	Information	Interaction	0.006	0.023		
Meconium Adj.[c]	Bifidobacterium longum	QTAC	Caffeine	-0.436	0.912	0.708	0.975
Meconium Adj.[c]	Bifidobacterium longum	QTAC	Species	0.384	0.631		
Meconium Adj.[c]	Bifidobacterium longum	QTAC	Interaction	-0.026	0.078		
Meconium Adj.[c]	Bifidobacterium longum	Vocabulary	Caffeine	-0.123	0.404	0.973	0.994
Meconium Adj.[c]	Bifidobacterium longum	Vocabulary	Species	0.054	0.28		
Meconium Adj.[c]	Bifidobacterium longum	Vocabulary	Interaction	0.001	0.034		
Meconium Adj.[c]	Bifidobacterium longum	WISC sum	Caffeine	-0.798	0.918	0.949	0.994
Meconium Adj.[c]	Bifidobacterium longum	WISC sum	Species	0.006	0.635		
Meconium Adj.[c]	Bifidobacterium longum	WISC sum	Interaction	0.005	0.078		
Meconium Adj.[c]	Bifidobacterium pseudocatenulatum	Block Design	Caffeine	-0.285	0.263	0.995	0.995
Meconium Adj.[c]	Bifidobacterium pseudocatenulatum	Block Design	Species	-0.055	0.857		
Meconium Adj.[c]	Bifidobacterium pseudocatenulatum	Block Design	Interaction	0.001	0.095		
Meconium Adj.[c]	Bifidobacterium pseudocatenulatum	Coding	Caffeine	0.246	0.227	0.158	0.844
Meconium Adj.[c]	Bifidobacterium pseudocatenulatum	Coding	Species	1.011	0.738		
Meconium Adj.[c]	Bifidobacterium pseudocatenulatum	Coding	Interaction	-0.106	0.082		
Meconium Adj.[c]	Bifidobacterium pseudocatenulatum	Digit span	Caffeine	-0.345	0.196	0.719	0.975
Meconium Adj.[c]	Bifidobacterium pseudocatenulatum	Digit span	Species	-0.235	0.637		
Meconium Adj.[c]	Bifidobacterium pseudocatenulatum	Digit span	Interaction	0.023	0.071		
Meconium Adj.[c]	Bifidobacterium pseudocatenulatum	Information	Caffeine	-0.116	0.195	0.973	0.994
Meconium Adj.[c]	Bifidobacterium pseudocatenulatum	Information	Species	-0.004	0.636		

Exposure Window	Species	Outcome	Variable	Effect Estimate[a]	Std. Error	LRT[a]	
						p-value	q-value
Meconium Adj.[c]	Bifidobacterium pseudocatenulatum	Information	Interaction	-0.002	0.071		
Meconium Adj.[c]	Bifidobacterium pseudocatenulatum	QTAC	Caffeine	-0.508	0.635	0.257	0.884
Meconium Adj.[c]	Bifidobacterium pseudocatenulatum	QTAC	Species	2.69	2.066		
Meconium Adj.[c]	Bifidobacterium pseudocatenulatum	QTAC	Interaction	-0.237	0.23		
Meconium Adj.[c]	Bifidobacterium pseudocatenulatum	Vocabulary	Caffeine	-0.061	0.297	0.523	0.975
Meconium Adj.[c]	Bifidobacterium pseudocatenulatum	Vocabulary	Species	0.594	0.967		
Meconium Adj.[c]	Bifidobacterium pseudocatenulatum	Vocabulary	Interaction	-0.062	0.107		
Meconium Adj.[c]	Bifidobacterium pseudocatenulatum	WISC sum	Caffeine	-0.562	0.667	0.503	0.975
Meconium Adj.[c]	Bifidobacterium pseudocatenulatum	WISC sum	Species	1.311	2.172		
Meconium Adj.[c]	Bifidobacterium pseudocatenulatum	WISC sum	Interaction	-0.146	0.241		
Meconium Adj.[c]	Blautia obeum	Block Design	Caffeine	-0.292	0.307	0.987	0.994
Meconium Adj.[c]	Blautia obeum	Block Design	Species	-0.082	0.803		
Meconium Adj.[c]	Blautia obeum	Block Design	Interaction	-0.002	0.112		
Meconium Adj.[c]	Blautia obeum	Coding	Caffeine	0.349	0.26	0.141	0.844
Meconium Adj.[c]	Blautia obeum	Coding	Species	1.079	0.68		
Meconium Adj.[c]	Blautia obeum	Coding	Interaction	-0.128	0.095		
Meconium Adj.[c]	Blautia obeum	Digit span	Caffeine	-0.407	0.218	0.466	0.975
Meconium Adj.[c]	Blautia obeum	Digit span	Species	-0.161	0.57		
Meconium Adj.[c]	Blautia obeum	Digit span	Interaction	0.052	0.079		
Meconium Adj.[c]	Blautia obeum	Information	Caffeine	-0.048	0.226	0.616	0.975
Meconium Adj.[c]	Blautia obeum	Information	Species	0.36	0.591		
Meconium Adj.[c]	Blautia obeum	Information	Interaction	-0.037	0.082		
Meconium Adj.[c]	Blautia obeum	QTAC	Caffeine	-0.162	0.777	0.205	0.844
Meconium Adj.[c]	Blautia obeum	QTAC	Species	2.017	2.031		
Meconium Adj.[c]	Blautia obeum	QTAC	Interaction	-0.327	0.283		
Meconium Adj.[c]	Blautia obeum	Vocabulary	Caffeine	-0.054	0.348	0.681	0.975
Meconium Adj.[c]	Blautia obeum	Vocabulary	Species	0.426	0.909		
Meconium Adj.[c]	Blautia obeum	Vocabulary	Interaction	-0.047	0.127		
Meconium Adj.[c]	Blautia obeum	WISC sum	Caffeine	-0.452	0.765	0.523	0.975
Meconium Adj.[c]	Blautia obeum	WISC sum	Species	1.622	1.999		
Meconium Adj.[c]	Blautia obeum	WISC sum	Interaction	-0.161	0.279		
Meconium Adj.[c]	Blautia wexlerae	Block Design	Caffeine	-0.242	0.28	0.745	0.99
Meconium Adj.[c]	Blautia wexlerae	Block Design	Species	0.248	0.898		
Meconium Adj.[c]	Blautia wexlerae	Block Design	Interaction	-0.027	0.092		
Meconium Adj.[c]	Blautia wexlerae	Coding	Caffeine	0.167	0.241	0.567	0.975
Meconium Adj.[c]	Blautia wexlerae	Coding	Species	0.175	0.775		

Exposure Window	Species	Outcome	Variable	Effect Estimate[a]	Std. Error	LRT[a]	
						p-value	q-value
Meconium Adj.[c]	Blautia wexlerae	Coding	Interaction	-0.041	0.079		
Meconium Adj.[c]	Blautia wexlerae	Digit span	Caffeine	-0.5	0.202	0.106	0.844
Meconium Adj.[c]	Blautia wexlerae	Digit span	Species	-1.012	0.65		
Meconium Adj.[c]	Blautia wexlerae	Digit span	Interaction	0.098	0.066		
Meconium Adj.[c]	Blautia wexlerae	Information	Caffeine	-0.088	0.196	0.824	0.994
Meconium Adj.[c]	Blautia wexlerae	Information	Species	0.427	0.629		
Meconium Adj.[c]	Blautia wexlerae	Information	Interaction	-0.013	0.064		
Meconium Adj.[c]	Blautia wexlerae	QTAC	Caffeine	-0.693	0.719	0.82	0.994
Meconium Adj.[c]	Blautia wexlerae	QTAC	Species	0.228	2.308		
Meconium Adj.[c]	Blautia wexlerae	QTAC	Interaction	-0.048	0.235		
Meconium Adj.[c]	Blautia wexlerae	Vocabulary	Caffeine	0.052	0.303	0.267	0.884
Meconium Adj.[c]	Blautia wexlerae	Vocabulary	Species	1.305	0.974		
Meconium Adj.[c]	Blautia wexlerae	Vocabulary	Interaction	-0.1	0.099		
Meconium Adj.[c]	Blautia wexlerae	WISC sum	Caffeine	-0.611	0.706	0.691	0.975
Meconium Adj.[c]	Blautia wexlerae	WISC sum	Species	1.144	2.267		
Meconium Adj.[c]	Blautia wexlerae	WISC sum	Interaction	-0.083	0.231		
Meconium Adj.[c]	Collinsella aerofaciens	Block Design	Caffeine	-0.392	0.36	0.705	0.975
Meconium Adj.[c]	Collinsella aerofaciens	Block Design	Species	-0.105	0.331		
Meconium Adj.[c]	Collinsella aerofaciens	Block Design	Interaction	0.013	0.037		
Meconium Adj.[c]	Collinsella aerofaciens	Coding	Caffeine	-0.177	0.311	0.175	0.844
Meconium Adj.[c]	Collinsella aerofaciens	Coding	Species	-0.377	0.287		
Meconium Adj.[c]	Collinsella aerofaciens	Coding	Interaction	0.04	0.032		
Meconium Adj.[c]	Collinsella aerofaciens	Digit span	Caffeine	-0.368	0.268	0.75	0.992
Meconium Adj.[c]	Collinsella aerofaciens	Digit span	Species	-0.078	0.247		
Meconium Adj.[c]	Collinsella aerofaciens	Digit span	Interaction	0.008	0.028		
Meconium Adj.[c]	Collinsella aerofaciens	Information	Caffeine	0.145	0.261	0.139	0.844
Meconium Adj.[c]	Collinsella aerofaciens	Information	Species	0.326	0.24		
Meconium Adj.[c]	Collinsella aerofaciens	Information	Interaction	-0.036	0.027		
Meconium Adj.[c]	Collinsella aerofaciens	QTAC	Caffeine	-0.912	0.927	0.892	0.994
Meconium Adj.[c]	Collinsella aerofaciens	QTAC	Species	-0.035	0.853		
Meconium Adj.[c]	Collinsella aerofaciens	QTAC	Interaction	0.012	0.095		
Meconium Adj.[c]	Collinsella aerofaciens	Vocabulary	Caffeine	-0.203	0.408	0.846	0.994
Meconium Adj.[c]	Collinsella aerofaciens	Vocabulary	Species	-0.065	0.376		
Meconium Adj.[c]	Collinsella aerofaciens	Vocabulary	Interaction	0.007	0.042		
Meconium Adj.[c]	Collinsella aerofaciens	WISC sum	Caffeine	-0.995	0.915	0.713	0.975
Meconium Adj.[c]	Collinsella aerofaciens	WISC sum	Species	-0.3	0.842		

Exposure Window	Species	Outcome	Variable	Effect Estimate[a]	Std. Error	LRT[a]	
						p-value	q-value
Meconium Adj.[c]	Collinsella aerofaciens	WISC sum	Interaction	0.031	0.094		
Meconium Adj.[c]	Coprococcus catus	Block Design	Caffeine	-0.639	0.427	0.275	0.884
Meconium Adj.[c]	Coprococcus catus	Block Design	Species	-3.81	3.717		
Meconium Adj.[c]	Coprococcus catus	Block Design	Interaction	0.45	0.454		
Meconium Adj.[c]	Coprococcus catus	Coding	Caffeine	0.286	0.379	0.505	0.975
Meconium Adj.[c]	Coprococcus catus	Coding	Species	2.066	3.307		
Meconium Adj.[c]	Coprococcus catus	Coding	Interaction	-0.243	0.404		
Meconium Adj.[c]	Coprococcus catus	Digit span	Caffeine	-0.675	0.314	0.134	0.844
Meconium Adj.[c]	Coprococcus catus	Digit span	Species	-3.756	2.738		
Meconium Adj.[c]	Coprococcus catus	Digit span	Interaction	0.458	0.334		
Meconium Adj.[c]	Coprococcus catus	Information	Caffeine	-0.642	0.306	0.033	0.777
Meconium Adj.[c]	Coprococcus catus	Information	Species	-5.04	2.662		
Meconium Adj.[c]	Coprococcus catus	Information	Interaction	0.641	0.325		
Meconium Adj.[c]	Coprococcus catus	QTAC	Caffeine	-2.089	1.012	0.2	0.844
Meconium Adj.[c]	Coprococcus catus	QTAC	Species	-5.183	8.821		
Meconium Adj.[c]	Coprococcus catus	QTAC	Interaction	1.259	1.077		
Meconium Adj.[c]	Coprococcus catus	Vocabulary	Caffeine	-0.992	0.464	0.024	0.777
Meconium Adj.[c]	Coprococcus catus	Vocabulary	Species	-7.995	4.04		
Meconium Adj.[c]	Coprococcus catus	Vocabulary	Interaction	1.035	0.493		
Meconium Adj.[c]	Coprococcus catus	WISC sum	Caffeine	-2.663	1.041	0.023	0.777
Meconium Adj.[c]	Coprococcus catus	WISC sum	Species	-18.535	9.072		
Meconium Adj.[c]	Coprococcus catus	WISC sum	Interaction	2.341	1.108		
Meconium Adj.[c]	Coprococcus comes	Block Design	Caffeine	-0.809	0.346	0.04	0.777
Meconium Adj.[c]	Coprococcus comes	Block Design	Species	-2.662	1.458		
Meconium Adj.[c]	Coprococcus comes	Block Design	Interaction	0.328	0.174		
Meconium Adj.[c]	Coprococcus comes	Coding	Caffeine	0.106	0.314	0.905	0.994
Meconium Adj.[c]	Coprococcus comes	Coding	Species	0.45	1.323		
Meconium Adj.[c]	Coprococcus comes	Coding	Interaction	-0.017	0.157		
Meconium Adj.[c]	Coprococcus comes	Digit span	Caffeine	-0.631	0.259	0.104	0.844
Meconium Adj.[c]	Coprococcus comes	Digit span	Species	-1.373	1.092		
Meconium Adj.[c]	Coprococcus comes	Digit span	Interaction	0.193	0.13		
Meconium Adj.[c]	Coprococcus comes	Information	Caffeine	-0.292	0.265	0.379	0.971
Meconium Adj.[c]	Coprococcus comes	Information	Species	-0.837	1.118		
Meconium Adj.[c]	Coprococcus comes	Information	Interaction	0.106	0.133		
Meconium Adj.[c]	Coprococcus comes	QTAC	Caffeine	-2.354	0.847	0.016	0.777
Meconium Adj.[c]	Coprococcus comes	QTAC	Species	-6.471	3.567		

Exposure Window	Species	Outcome	Variable	Effect Estimate[a]	Std. Error	LRT[a]	
						p-value	q-value
Meconium Adj.[c]	Coprococcus comes	QTAC	Interaction	0.954	0.425		
Meconium Adj.[c]	Coprococcus comes	Vocabulary	Caffeine	-0.566	0.394	0.166	0.844
Meconium Adj.[c]	Coprococcus comes	Vocabulary	Species	-1.626	1.66		
Meconium Adj.[c]	Coprococcus comes	Vocabulary	Interaction	0.25	0.198		
Meconium Adj.[c]	Coprococcus comes	WISC sum	Caffeine	-2.192	0.858	0.031	0.777
Meconium Adj.[c]	Coprococcus comes	WISC sum	Species	-6.048	3.614		
Meconium Adj.[c]	Coprococcus comes	WISC sum	Interaction	0.861	0.43		
Meconium Adj.[c]	Coprococcus eutactus	Block Design	Caffeine	-0.463	0.24	0.065	0.777
Meconium Adj.[c]	Coprococcus eutactus	Block Design	Species	-1.762	0.995		
Meconium Adj.[c]	Coprococcus eutactus	Block Design	Interaction	0.184	0.108		
Meconium Adj.[c]	Coprococcus eutactus	Coding	Caffeine	0.063	0.216	0.512	0.975
Meconium Adj.[c]	Coprococcus eutactus	Coding	Species	-0.767	0.895		
Meconium Adj.[c]	Coprococcus eutactus	Coding	Interaction	0.058	0.098		
Meconium Adj.[c]	Coprococcus eutactus	Digit span	Caffeine	-0.409	0.183	0.263	0.884
Meconium Adj.[c]	Coprococcus eutactus	Digit span	Species	-0.615	0.755		
Meconium Adj.[c]	Coprococcus eutactus	Digit span	Interaction	0.084	0.082		
Meconium Adj.[c]	Coprococcus eutactus	Information	Caffeine	-0.136	0.183	0.707	0.975
Meconium Adj.[c]	Coprococcus eutactus	Information	Species	-0.42	0.757		
Meconium Adj.[c]	Coprococcus eutactus	Information	Interaction	0.028	0.083		
Meconium Adj.[c]	Coprococcus eutactus	QTAC	Caffeine	-0.73	0.643	0.837	0.994
Meconium Adj.[c]	Coprococcus eutactus	QTAC	Species	0.586	2.658		
Meconium Adj.[c]	Coprococcus eutactus	QTAC	Interaction	-0.054	0.29		
Meconium Adj.[c]	Coprococcus eutactus	Vocabulary	Caffeine	-0.108	0.28	0.884	0.994
Meconium Adj.[c]	Coprococcus eutactus	Vocabulary	Species	-0.118	1.158		
Meconium Adj.[c]	Coprococcus eutactus	Vocabulary	Interaction	-0.017	0.126		
Meconium Adj.[c]	Coprococcus eutactus	WISC sum	Caffeine	-1.053	0.615	0.183	0.844
Meconium Adj.[c]	Coprococcus eutactus	WISC sum	Species	-3.682	2.544		
Meconium Adj.[c]	Coprococcus eutactus	WISC sum	Interaction	0.337	0.277		
Meconium Adj.[c]	Dorea formicigenerans	Block Design	Caffeine	-0.268	0.397	0.904	0.994
Meconium Adj.[c]	Dorea formicigenerans	Block Design	Species	0.927	2.733		
Meconium Adj.[c]	Dorea formicigenerans	Block Design	Interaction	-0.036	0.329		
Meconium Adj.[c]	Dorea formicigenerans	Coding	Caffeine	-0.036	0.348	0.572	0.975
Meconium Adj.[c]	Dorea formicigenerans	Coding	Species	-1.874	2.392		
Meconium Adj.[c]	Dorea formicigenerans	Coding	Interaction	0.147	0.288		
Meconium Adj.[c]	Dorea formicigenerans	Digit span	Caffeine	-0.608	0.274	0.129	0.844
Meconium Adj.[c]	Dorea formicigenerans	Digit span	Species	-3.723	1.888		

Exposure Window	Species	Outcome	Variable	Effect Estimate[a]	Std. Error	LRT[a]	
						p-value	q-value
Meconium Adj.[c]	Dorea formicigenerans	Digit span	Interaction	0.315	0.227		
Meconium Adj.[c]	Dorea formicigenerans	Information	Caffeine	-0.749	0.272	0.003	0.408
Meconium Adj.[c]	Dorea formicigenerans	Information	Species	-4.903	1.871		
Meconium Adj.[c]	Dorea formicigenerans	Information	Interaction	0.622	0.225		
Meconium Adj.[c]	Dorea formicigenerans	QTAC	Caffeine	-0.9	1.031	0.864	0.994
Meconium Adj.[c]	Dorea formicigenerans	QTAC	Species	-1.343	7.095		
Meconium Adj.[c]	Dorea formicigenerans	QTAC	Interaction	0.132	0.854		
Meconium Adj.[c]	Dorea formicigenerans	Vocabulary	Caffeine	-0.781	0.437	0.059	0.777
Meconium Adj.[c]	Dorea formicigenerans	Vocabulary	Species	-4.844	3.008		
Meconium Adj.[c]	Dorea formicigenerans	Vocabulary	Interaction	0.628	0.362		
Meconium Adj.[c]	Dorea formicigenerans	WISC sum	Caffeine	-2.442	0.962	0.023	0.777
Meconium Adj.[c]	Dorea formicigenerans	WISC sum	Species	-14.417	6.623		
Meconium Adj.[c]	Dorea formicigenerans	WISC sum	Interaction	1.676	0.797		
Meconium Adj.[c]	Dorea longicatena	Block Design	Caffeine	-0.736	0.422	0.17	0.844
Meconium Adj.[c]	Dorea longicatena	Block Design	Species	-1.345	1.099		
Meconium Adj.[c]	Dorea longicatena	Block Design	Interaction	0.161	0.128		
Meconium Adj.[c]	Dorea longicatena	Coding	Caffeine	0.292	0.375	0.625	0.975
Meconium Adj.[c]	Dorea longicatena	Coding	Species	0.237	0.976		
Meconium Adj.[c]	Dorea longicatena	Coding	Interaction	-0.05	0.114		
Meconium Adj.[c]	Dorea longicatena	Digit span	Caffeine	-0.148	0.318	0.56	0.975
Meconium Adj.[c]	Dorea longicatena	Digit span	Species	0.336	0.828		
Meconium Adj.[c]	Dorea longicatena	Digit span	Interaction	-0.051	0.097		
Meconium Adj.[c]	Dorea longicatena	Information	Caffeine	-0.393	0.308	0.399	0.971
Meconium Adj.[c]	Dorea longicatena	Information	Species	-0.356	0.804		
Meconium Adj.[c]	Dorea longicatena	Information	Interaction	0.072	0.094		
Meconium Adj.[c]	Dorea longicatena	QTAC	Caffeine	-0.672	1.106	0.902	0.994
Meconium Adj.[c]	Dorea longicatena	QTAC	Species	0.321	2.884		
Meconium Adj.[c]	Dorea longicatena	QTAC	Interaction	-0.038	0.336		
Meconium Adj.[c]	Dorea longicatena	Vocabulary	Caffeine	-0.787	0.453	0.165	0.844
Meconium Adj.[c]	Dorea longicatena	Vocabulary	Species	-0.926	1.18		
Meconium Adj.[c]	Dorea longicatena	Vocabulary	Interaction	0.174	0.138		
Meconium Adj.[c]	Dorea longicatena	WISC sum	Caffeine	-1.772	1.07	0.301	0.932
Meconium Adj.[c]	Dorea longicatena	WISC sum	Species	-2.054	2.791		
Meconium Adj.[c]	Dorea longicatena	WISC sum	Interaction	0.305	0.325		
Meconium Adj.[c]	Eubacterium hallii	Block Design	Caffeine	0.132	0.416	0.197	0.844
Meconium Adj.[c]	Eubacterium hallii	Block Design	Species	1.668	1.354		

Exposure Window	Species	Outcome	Variable	Effect Estimate[a]	Std. Error	LRT[a]	
						p-value	q-value
Meconium Adj.[c]	Eubacterium hallii	Block Design	Interaction	-0.197	0.167		
Meconium Adj.[c]	Eubacterium hallii	Coding	Caffeine	0.126	0.369	0.86	0.994
Meconium Adj.[c]	Eubacterium hallii	Coding	Species	-0.115	1.201		
Meconium Adj.[c]	Eubacterium hallii	Coding	Interaction	-0.024	0.149		
Meconium Adj.[c]	Eubacterium hallii	Digit span	Caffeine	-0.211	0.311	0.72	0.975
Meconium Adj.[c]	Eubacterium hallii	Digit span	Species	0.56	1.013		
Meconium Adj.[c]	Eubacterium hallii	Digit span	Interaction	-0.041	0.125		
Meconium Adj.[c]	Eubacterium hallii	Information	Caffeine	0.22	0.304	0.169	0.844
Meconium Adj.[c]	Eubacterium hallii	Information	Species	1.456	0.989		
Meconium Adj.[c]	Eubacterium hallii	Information	Interaction	-0.154	0.122		
Meconium Adj.[c]	Eubacterium hallii	QTAC	Caffeine	-2.569	1.037	0.03	0.777
Meconium Adj.[c]	Eubacterium hallii	QTAC	Species	-6.953	3.376		
Meconium Adj.[c]	Eubacterium hallii	QTAC	Interaction	0.837	0.418		
Meconium Adj.[c]	Eubacterium hallii	Vocabulary	Caffeine	-0.28	0.467	0.63	0.975
Meconium Adj.[c]	Eubacterium hallii	Vocabulary	Species	-0.107	1.519		
Meconium Adj.[c]	Eubacterium hallii	Vocabulary	Interaction	0.082	0.188		
Meconium Adj.[c]	Eubacterium hallii	WISC sum	Caffeine	-0.013	1.055	0.388	0.971
Meconium Adj.[c]	Eubacterium hallii	WISC sum	Species	3.462	3.434		
Meconium Adj.[c]	Eubacterium hallii	WISC sum	Interaction	-0.333	0.425		
Meconium Adj.[c]	Eubacterium rectale	Block Design	Caffeine	-0.114	0.236	0.062	0.777
Meconium Adj.[c]	Eubacterium rectale	Block Design	Species	0.435	0.299		
Meconium Adj.[c]	Eubacterium rectale	Block Design	Interaction	-0.056	0.033		
Meconium Adj.[c]	Eubacterium rectale	Coding	Caffeine	0.136	0.216	0.662	0.975
Meconium Adj.[c]	Eubacterium rectale	Coding	Species	0.097	0.274		
Meconium Adj.[c]	Eubacterium rectale	Coding	Interaction	-0.012	0.03		
Meconium Adj.[c]	Eubacterium rectale	Digit span	Caffeine	-0.331	0.182	0.851	0.994
Meconium Adj.[c]	Eubacterium rectale	Digit span	Species	-0.002	0.231		
Meconium Adj.[c]	Eubacterium rectale	Digit span	Interaction	0.004	0.025		
Meconium Adj.[c]	Eubacterium rectale	Information	Caffeine	-0.178	0.18	0.41	0.971
Meconium Adj.[c]	Eubacterium rectale	Information	Species	-0.202	0.228		
Meconium Adj.[c]	Eubacterium rectale	Information	Interaction	0.019	0.025		
Meconium Adj.[c]	Eubacterium rectale	QTAC	Caffeine	-0.453	0.617	0.201	0.844
Meconium Adj.[c]	Eubacterium rectale	QTAC	Species	0.693	0.781		
Meconium Adj.[c]	Eubacterium rectale	QTAC	Interaction	-0.1	0.086		
Meconium Adj.[c]	Eubacterium rectale	Vocabulary	Caffeine	-0.086	0.271	0.617	0.975
Meconium Adj.[c]	Eubacterium rectale	Vocabulary	Species	0.021	0.343		

Exposure Window	Species	Outcome	Variable	Effect Estimate[a]	Std. Error	LRT[a]	
						p-value	q-value
Meconium Adj.[c]	Eubacterium rectale	Vocabulary	Interaction	-0.017	0.038		
Meconium Adj.[c]	Eubacterium rectale	WISC sum	Caffeine	-0.573	0.614	0.422	0.971
Meconium Adj.[c]	Eubacterium rectale	WISC sum	Species	0.349	0.777		
Meconium Adj.[c]	Eubacterium rectale	WISC sum	Interaction	-0.062	0.085		
Meconium Adj.[c]	Eubacterium siraeum	Block Design	Caffeine	-0.282	0.227	0.908	0.994
Meconium Adj.[c]	Eubacterium siraeum	Block Design	Species	-0.387	2.171		
Meconium Adj.[c]	Eubacterium siraeum	Block Design	Interaction	-0.024	0.234		
Meconium Adj.[c]	Eubacterium siraeum	Coding	Caffeine	0.08	0.21	0.789	0.994
Meconium Adj.[c]	Eubacterium siraeum	Coding	Species	-0.461	2.006		
Meconium Adj.[c]	Eubacterium siraeum	Coding	Interaction	0.052	0.216		
Meconium Adj.[c]	Eubacterium siraeum	Digit span	Caffeine	-0.335	0.177	0.721	0.975
Meconium Adj.[c]	Eubacterium siraeum	Digit span	Species	-0.543	1.691		
Meconium Adj.[c]	Eubacterium siraeum	Digit span	Interaction	0.059	0.182		
Meconium Adj.[c]	Eubacterium siraeum	Information	Caffeine	-0.13	0.176	0.901	0.994
Meconium Adj.[c]	Eubacterium siraeum	Information	Species	-0.18	1.685		
Meconium Adj.[c]	Eubacterium siraeum	Information	Interaction	0.02	0.182		
Meconium Adj.[c]	Eubacterium siraeum	QTAC	Caffeine	-0.82	0.609	0.821	0.994
Meconium Adj.[c]	Eubacterium siraeum	QTAC	Species	-0.598	5.819		
Meconium Adj.[c]	Eubacterium siraeum	QTAC	Interaction	0.128	0.627		
Meconium Adj.[c]	Eubacterium siraeum	Vocabulary	Caffeine	-0.09	0.262	0.498	0.975
Meconium Adj.[c]	Eubacterium siraeum	Vocabulary	Species	2.008	2.506		
Meconium Adj.[c]	Eubacterium siraeum	Vocabulary	Interaction	-0.166	0.27		
Meconium Adj.[c]	Eubacterium siraeum	WISC sum	Caffeine	-0.758	0.605	0.917	0.994
Meconium Adj.[c]	Eubacterium siraeum	WISC sum	Species	0.437	5.778		
Meconium Adj.[c]	Eubacterium siraeum	WISC sum	Interaction	-0.059	0.623		
Meconium Adj.[c]	Eubacterium sp CAG 180	Block Design	Caffeine	-0.363	0.227	0.44	0.975
Meconium Adj.[c]	Eubacterium sp CAG 180	Block Design	Species	-1.836	1.945		
Meconium Adj.[c]	Eubacterium sp CAG 180	Block Design	Interaction	0.168	0.24		
Meconium Adj.[c]	Eubacterium sp CAG 180	Coding	Caffeine	0.069	0.207	0.709	0.975
Meconium Adj.[c]	Eubacterium sp CAG 180	Coding	Species	-0.761	1.77		
Meconium Adj.[c]	Eubacterium sp CAG 180	Coding	Interaction	0.074	0.219		
Meconium Adj.[c]	Eubacterium sp CAG 180	Digit span	Caffeine	-0.311	0.171	0.872	0.994
Meconium Adj.[c]	Eubacterium sp CAG 180	Digit span	Species	0.103	1.468		
Meconium Adj.[c]	Eubacterium sp CAG 180	Digit span	Interaction	0.026	0.182		
Meconium Adj.[c]	Eubacterium sp CAG 180	Information	Caffeine	-0.042	0.158	0.217	0.844
Meconium Adj.[c]	Eubacterium sp CAG 180	Information	Species	2.092	1.349		

Exposure Window	Species	Outcome	Variable	Effect Estimate[a]	Std. Error	LRT[a]	
						p-value	q-value
Meconium Adj.[c]	Eubacterium sp CAG 180	Information	Interaction	-0.187	0.167		
Meconium Adj.[c]	Eubacterium sp CAG 180	QTAC	Caffeine	-0.904	0.604	0.476	0.975
Meconium Adj.[c]	Eubacterium sp CAG 180	QTAC	Species	-3.323	5.169		
Meconium Adj.[c]	Eubacterium sp CAG 180	QTAC	Interaction	0.413	0.639		
Meconium Adj.[c]	Eubacterium sp CAG 180	Vocabulary	Caffeine	-0.135	0.261	0.944	0.994
Meconium Adj.[c]	Eubacterium sp CAG 180	Vocabulary	Species	0.336	2.233		
Meconium Adj.[c]	Eubacterium sp CAG 180	Vocabulary	Interaction	0.018	0.276		
Meconium Adj.[c]	Eubacterium sp CAG 180	WISC sum	Caffeine	-0.782	0.593	0.862	0.994
Meconium Adj.[c]	Eubacterium sp CAG 180	WISC sum	Species	-0.066	5.074		
Meconium Adj.[c]	Eubacterium sp CAG 180	WISC sum	Interaction	0.099	0.627		
Meconium Adj.[c]	Faecalibacterium prausnitzii	Block Design	Caffeine	-0.001	0.341	0.217	0.844
Meconium Adj.[c]	Faecalibacterium prausnitzii	Block Design	Species	0.359	0.349		
Meconium Adj.[c]	Faecalibacterium prausnitzii	Block Design	Interaction	-0.05	0.045		
Meconium Adj.[c]	Faecalibacterium prausnitzii	Coding	Caffeine	-0.098	0.296	0.308	0.938
Meconium Adj.[c]	Faecalibacterium prausnitzii	Coding	Species	-0.176	0.303		
Meconium Adj.[c]	Faecalibacterium prausnitzii	Coding	Interaction	0.036	0.039		
Meconium Adj.[c]	Faecalibacterium prausnitzii	Digit span	Caffeine	-0.233	0.256	0.666	0.975
Meconium Adj.[c]	Faecalibacterium prausnitzii	Digit span	Species	0.133	0.263		
Meconium Adj.[c]	Faecalibacterium prausnitzii	Digit span	Interaction	-0.013	0.034		
Meconium Adj.[c]	Faecalibacterium prausnitzii	Information	Caffeine	-0.313	0.249	0.302	0.932
Meconium Adj.[c]	Faecalibacterium prausnitzii	Information	Species	-0.309	0.255		
Meconium Adj.[c]	Faecalibacterium prausnitzii	Information	Interaction	0.031	0.033		
Meconium Adj.[c]	Faecalibacterium prausnitzii	QTAC	Caffeine	-0.353	0.885	0.51	0.975
Meconium Adj.[c]	Faecalibacterium prausnitzii	QTAC	Species	0.639	0.907		
Meconium Adj.[c]	Faecalibacterium prausnitzii	QTAC	Interaction	-0.069	0.116		
Meconium Adj.[c]	Faecalibacterium prausnitzii	Vocabulary	Caffeine	-0.154	0.383	0.966	0.994
Meconium Adj.[c]	Faecalibacterium prausnitzii	Vocabulary	Species	-0.116	0.393		
Meconium Adj.[c]	Faecalibacterium prausnitzii	Vocabulary	Interaction	-0.002	0.05		
Meconium Adj.[c]	Faecalibacterium prausnitzii	WISC sum	Caffeine	-0.8	0.878	0.989	0.994
Meconium Adj.[c]	Faecalibacterium prausnitzii	WISC sum	Species	-0.108	0.9		
Meconium Adj.[c]	Faecalibacterium prausnitzii	WISC sum	Interaction	0.001	0.115		
Meconium Adj.[c]	Fusicatenibacter saccharivorans	Block Design	Caffeine	-0.402	0.384	0.691	0.975
Meconium Adj.[c]	Fusicatenibacter saccharivorans	Block Design	Species	-0.182	0.505		
Meconium Adj.[c]	Fusicatenibacter saccharivorans	Block Design	Interaction	0.019	0.054		
Meconium Adj.[c]	Fusicatenibacter saccharivorans	Coding	Caffeine	0.258	0.337	0.586	0.975
Meconium Adj.[c]	Fusicatenibacter saccharivorans	Coding	Species	0.153	0.442		

Exposure Window	Species	Outcome	Variable	Effect Estimate[a]	Std. Error	LRT[a]	
						p-value	q-value
Meconium Adj.[c]	Fusicatenibacter saccharivorans	Coding	Interaction	-0.023	0.047		
Meconium Adj.[c]	Fusicatenibacter saccharivorans	Digit span	Caffeine	-0.147	0.284	0.45	0.975
Meconium Adj.[c]	Fusicatenibacter saccharivorans	Digit span	Species	0.226	0.373		
Meconium Adj.[c]	Fusicatenibacter saccharivorans	Digit span	Interaction	-0.027	0.04		
Meconium Adj.[c]	Fusicatenibacter saccharivorans	Information	Caffeine	-0.295	0.283	0.421	0.971
Meconium Adj.[c]	Fusicatenibacter saccharivorans	Information	Species	-0.255	0.372		
Meconium Adj.[c]	Fusicatenibacter saccharivorans	Information	Interaction	0.029	0.04		
Meconium Adj.[c]	Fusicatenibacter saccharivorans	QTAC	Caffeine	-1.571	0.97	0.225	0.844
Meconium Adj.[c]	Fusicatenibacter saccharivorans	QTAC	Species	-1.56	1.274		
Meconium Adj.[c]	Fusicatenibacter saccharivorans	QTAC	Interaction	0.151	0.137		
Meconium Adj.[c]	Fusicatenibacter saccharivorans	Vocabulary	Caffeine	-0.548	0.422	0.267	0.884
Meconium Adj.[c]	Fusicatenibacter saccharivorans	Vocabulary	Species	-0.411	0.554		
Meconium Adj.[c]	Fusicatenibacter saccharivorans	Vocabulary	Interaction	0.06	0.06		
Meconium Adj.[c]	Fusicatenibacter saccharivorans	WISC sum	Caffeine	-1.134	0.975	0.642	0.975
Meconium Adj.[c]	Fusicatenibacter saccharivorans	WISC sum	Species	-0.47	1.28		
Meconium Adj.[c]	Fusicatenibacter saccharivorans	WISC sum	Interaction	0.058	0.138		
Meconium Adj.[c]	Methanobrevibacter smithii	Block Design	Caffeine	-0.51	0.277	0.144	0.844
Meconium Adj.[c]	Methanobrevibacter smithii	Block Design	Species	-1.2	1.024		
Meconium Adj.[c]	Methanobrevibacter smithii	Block Design	Interaction	0.154	0.115		
Meconium Adj.[c]	Methanobrevibacter smithii	Coding	Caffeine	-0.021	0.247	0.348	0.963
Meconium Adj.[c]	Methanobrevibacter smithii	Coding	Species	-0.638	0.914		
Meconium Adj.[c]	Methanobrevibacter smithii	Coding	Interaction	0.088	0.103		
Meconium Adj.[c]	Methanobrevibacter smithii	Digit span	Caffeine	-0.391	0.21	0.478	0.975
Meconium Adj.[c]	Methanobrevibacter smithii	Digit span	Species	-0.401	0.775		
Meconium Adj.[c]	Methanobrevibacter smithii	Digit span	Interaction	0.056	0.087		
Meconium Adj.[c]	Methanobrevibacter smithii	Information	Caffeine	-0.135	0.21	0.9	0.994
Meconium Adj.[c]	Methanobrevibacter smithii	Information	Species	-0.048	0.778		
Meconium Adj.[c]	Methanobrevibacter smithii	Information	Interaction	0.01	0.088		
Meconium Adj.[c]	Methanobrevibacter smithii	QTAC	Caffeine	-0.945	0.705	0.528	0.975
Meconium Adj.[c]	Methanobrevibacter smithii	QTAC	Species	-0.536	2.607		
Meconium Adj.[c]	Methanobrevibacter smithii	QTAC	Interaction	0.168	0.293		
Meconium Adj.[c]	Methanobrevibacter smithii	Vocabulary	Caffeine	-0.15	0.317	0.887	0.994
Meconium Adj.[c]	Methanobrevibacter smithii	Vocabulary	Species	0.113	1.173		
Meconium Adj.[c]	Methanobrevibacter smithii	Vocabulary	Interaction	0.017	0.132		
Meconium Adj.[c]	Methanobrevibacter smithii	WISC sum	Caffeine	-1.207	0.7	0.221	0.844
Meconium Adj.[c]	Methanobrevibacter smithii	WISC sum	Species	-2.175	2.587		

Exposure Window	Species	Outcome	Variable	Effect Estimate[a]	Std. Error	LRT[a]	
						p-value	q-value
Meconium Adj.[c]	Methanobrevibacter smithii	WISC sum	Interaction	0.324	0.291		
Meconium Adj.[c]	Prevotella copri	Block Design	Caffeine	-0.36	0.239	0.407	0.971
Meconium Adj.[c]	Prevotella copri	Block Design	Species	-1.415	1.927		
Meconium Adj.[c]	Prevotella copri	Block Design	Interaction	0.159	0.211		
Meconium Adj.[c]	Prevotella copri	Coding	Caffeine	0.012	0.209	0.213	0.844
Meconium Adj.[c]	Prevotella copri	Coding	Species	-1.891	1.685		
Meconium Adj.[c]	Prevotella copri	Coding	Interaction	0.209	0.185		
Meconium Adj.[c]	Prevotella copri	Digit span	Caffeine	-0.28	0.176	0.926	0.994
Meconium Adj.[c]	Prevotella copri	Digit span	Species	-0.141	1.417		
Meconium Adj.[c]	Prevotella copri	Digit span	Interaction	-0.013	0.155		
Meconium Adj.[c]	Prevotella copri	Information	Caffeine	-0.217	0.172	0.064	0.777
Meconium Adj.[c]	Prevotella copri	Information	Species	-2.438	1.384		
Meconium Adj.[c]	Prevotella copri	Information	Interaction	0.258	0.152		
Meconium Adj.[c]	Prevotella copri	QTAC	Caffeine	-0.654	0.601	0.268	0.884
Meconium Adj.[c]	Prevotella copri	QTAC	Species	5.717	4.842		
Meconium Adj.[c]	Prevotella copri	QTAC	Interaction	-0.534	0.53		
Meconium Adj.[c]	Prevotella copri	Vocabulary	Caffeine	-0.064	0.27	0.475	0.975
Meconium Adj.[c]	Prevotella copri	Vocabulary	Species	1.22	2.177		
Meconium Adj.[c]	Prevotella copri	Vocabulary	Interaction	-0.154	0.238		
Meconium Adj.[c]	Prevotella copri	WISC sum	Caffeine	-0.909	0.603	0.342	0.963
Meconium Adj.[c]	Prevotella copri	WISC sum	Species	-4.665	4.853		
Meconium Adj.[c]	Prevotella copri	WISC sum	Interaction	0.458	0.531		
Meconium Adj.[c]	Roseburia faecis	Block Design	Caffeine	-0.286	0.254	0.985	0.994
Meconium Adj.[c]	Roseburia faecis	Block Design	Species	-0.017	1.696		
Meconium Adj.[c]	Roseburia faecis	Block Design	Interaction	-0.003	0.199		
Meconium Adj.[c]	Roseburia faecis	Coding	Caffeine	-0.017	0.213	0.158	0.844
Meconium Adj.[c]	Roseburia faecis	Coding	Species	-2.006	1.419		
Meconium Adj.[c]	Roseburia faecis	Coding	Interaction	0.214	0.167		
Meconium Adj.[c]	Roseburia faecis	Digit span	Caffeine	-0.285	0.186	0.625	0.975
Meconium Adj.[c]	Roseburia faecis	Digit span	Species	0.65	1.243		
Meconium Adj.[c]	Roseburia faecis	Digit span	Interaction	-0.064	0.146		
Meconium Adj.[c]	Roseburia faecis	Information	Caffeine	-0.153	0.184	0.609	0.975
Meconium Adj.[c]	Roseburia faecis	Information	Species	-0.684	1.231		
Meconium Adj.[c]	Roseburia faecis	Information	Interaction	0.067	0.144		
Meconium Adj.[c]	Roseburia faecis	QTAC	Caffeine	-0.53	0.644	0.451	0.975
Meconium Adj.[c]	Roseburia faecis	QTAC	Species	2.615	4.299		

Exposure Window	Species	Outcome	Variable	Effect Estimate[a]	Std. Error	LRT[a]	
						p-value	q-value
Meconium Adj.[c]	Roseburia faecis	QTAC	Interaction	-0.344	0.504		
Meconium Adj.[c]	Roseburia faecis	Vocabulary	Caffeine	-0.312	0.278	0.145	0.844
Meconium Adj.[c]	Roseburia faecis	Vocabulary	Species	-2.613	1.855		
Meconium Adj.[c]	Roseburia faecis	Vocabulary	Interaction	0.29	0.218		
Meconium Adj.[c]	Roseburia faecis	WISC sum	Caffeine	-1.052	0.626	0.259	0.884
Meconium Adj.[c]	Roseburia faecis	WISC sum	Species	-4.67	4.18		
Meconium Adj.[c]	Roseburia faecis	WISC sum	Interaction	0.503	0.49		
Meconium Adj.[c]	Roseburia intestinalis	Block Design	Caffeine	-0.244	0.253	0.725	0.975
Meconium Adj.[c]	Roseburia intestinalis	Block Design	Species	0.752	1.917		
Meconium Adj.[c]	Roseburia intestinalis	Block Design	Interaction	-0.078	0.246		
Meconium Adj.[c]	Roseburia intestinalis	Coding	Caffeine	0.131	0.223	0.686	0.975
Meconium Adj.[c]	Roseburia intestinalis	Coding	Species	0.498	1.692		
Meconium Adj.[c]	Roseburia intestinalis	Coding	Interaction	-0.079	0.217		
Meconium Adj.[c]	Roseburia intestinalis	Digit span	Caffeine	-0.297	0.179	0.798	0.994
Meconium Adj.[c]	Roseburia intestinalis	Digit span	Species	0.297	1.356		
Meconium Adj.[c]	Roseburia intestinalis	Digit span	Interaction	0.04	0.174		
Meconium Adj.[c]	Roseburia intestinalis	Information	Caffeine	-0.14	0.185	0.949	0.994
Meconium Adj.[c]	Roseburia intestinalis	Information	Species	-0.278	1.399		
Meconium Adj.[c]	Roseburia intestinalis	Information	Interaction	-0.01	0.179		
Meconium Adj.[c]	Roseburia intestinalis	QTAC	Caffeine	-0.329	0.624	0.064	0.777
Meconium Adj.[c]	Roseburia intestinalis	QTAC	Species	6.927	4.731		
Meconium Adj.[c]	Roseburia intestinalis	QTAC	Interaction	-1.032	0.606		
Meconium Adj.[c]	Roseburia intestinalis	Vocabulary	Caffeine	-0.201	0.287	0.702	0.975
Meconium Adj.[c]	Roseburia intestinalis	Vocabulary	Species	-0.835	2.172		
Meconium Adj.[c]	Roseburia intestinalis	Vocabulary	Interaction	0.096	0.278		
Meconium Adj.[c]	Roseburia intestinalis	WISC sum	Caffeine	-0.751	0.644	0.956	0.994
Meconium Adj.[c]	Roseburia intestinalis	WISC sum	Species	0.433	4.883		
Meconium Adj.[c]	Roseburia intestinalis	WISC sum	Interaction	-0.032	0.626		
Meconium Adj.[c]	Ruminococcus bicirculans	Block Design	Caffeine	-0.342	0.293	0.76	0.994
Meconium Adj.[c]	Ruminococcus bicirculans	Block Design	Species	-0.488	1.547		
Meconium Adj.[c]	Ruminococcus bicirculans	Block Design	Interaction	0.049	0.178		
Meconium Adj.[c]	Ruminococcus bicirculans	Coding	Caffeine	0.017	0.258	0.583	0.975
Meconium Adj.[c]	Ruminococcus bicirculans	Coding	Species	-0.713	1.363		
Meconium Adj.[c]	Ruminococcus bicirculans	Coding	Interaction	0.078	0.157		
Meconium Adj.[c]	Ruminococcus bicirculans	Digit span	Caffeine	-0.265	0.218	0.674	0.975
Meconium Adj.[c]	Ruminococcus bicirculans	Digit span	Species	0.532	1.148		

Exposure Window	Species	Outcome	Variable	Effect Estimate[a]	Std. Error	LRT[a]	
						p-value	q-value
Meconium Adj.[c]	Ruminococcus bicirculans	Digit span	Interaction	-0.05	0.132		
Meconium Adj.[c]	Ruminococcus bicirculans	Information	Caffeine	-0.237	0.216	0.366	0.971
Meconium Adj.[c]	Ruminococcus bicirculans	Information	Species	-0.957	1.138		
Meconium Adj.[c]	Ruminococcus bicirculans	Information	Interaction	0.107	0.131		
Meconium Adj.[c]	Ruminococcus bicirculans	QTAC	Caffeine	-1.025	0.754	0.582	0.975
Meconium Adj.[c]	Ruminococcus bicirculans	QTAC	Species	-1.824	3.977		
Meconium Adj.[c]	Ruminococcus bicirculans	QTAC	Interaction	0.228	0.458		
Meconium Adj.[c]	Ruminococcus bicirculans	Vocabulary	Caffeine	-0.457	0.309	0.072	0.808
Meconium Adj.[c]	Ruminococcus bicirculans	Vocabulary	Species	-3.225	1.63		
Meconium Adj.[c]	Ruminococcus bicirculans	Vocabulary	Interaction	0.31	0.188		
Meconium Adj.[c]	Ruminococcus bicirculans	WISC sum	Caffeine	-1.284	0.729	0.22	0.844
Meconium Adj.[c]	Ruminococcus bicirculans	WISC sum	Species	-4.851	3.842		
Meconium Adj.[c]	Ruminococcus bicirculans	WISC sum	Interaction	0.495	0.442		
Meconium Adj.[c]	Ruminococcus bromii	Block Design	Caffeine	-0.422	0.414	0.667	0.975
Meconium Adj.[c]	Ruminococcus bromii	Block Design	Species	-0.06	0.312		
Meconium Adj.[c]	Ruminococcus bromii	Block Design	Interaction	0.015	0.037		
Meconium Adj.[c]	Ruminococcus bromii	Coding	Caffeine	0.084	0.36	0.93	0.994
Meconium Adj.[c]	Ruminococcus bromii	Coding	Species	0.061	0.271		
Meconium Adj.[c]	Ruminococcus bromii	Coding	Interaction	0.003	0.032		
Meconium Adj.[c]	Ruminococcus bromii	Digit span	Caffeine	-0.488	0.302	0.434	0.975
Meconium Adj.[c]	Ruminococcus bromii	Digit span	Species	-0.087	0.227		
Meconium Adj.[c]	Ruminococcus bromii	Digit span	Interaction	0.019	0.027		
Meconium Adj.[c]	Ruminococcus bromii	Information	Caffeine	-0.145	0.306	0.904	0.994
Meconium Adj.[c]	Ruminococcus bromii	Information	Species	0.026	0.23		
Meconium Adj.[c]	Ruminococcus bromii	Information	Interaction	0.003	0.028		
Meconium Adj.[c]	Ruminococcus bromii	QTAC	Caffeine	-1.862	1.06	0.187	0.844
Meconium Adj.[c]	Ruminococcus bromii	QTAC	Species	-0.994	0.797		
Meconium Adj.[c]	Ruminococcus bromii	QTAC	Interaction	0.115	0.096		
Meconium Adj.[c]	Ruminococcus bromii	Vocabulary	Caffeine	0.062	0.468	0.576	0.975
Meconium Adj.[c]	Ruminococcus bromii	Vocabulary	Species	0.243	0.352		
Meconium Adj.[c]	Ruminococcus bromii	Vocabulary	Interaction	-0.021	0.042		
Meconium Adj.[c]	Ruminococcus bromii	WISC sum	Caffeine	-0.908	1.008	0.826	0.994
Meconium Adj.[c]	Ruminococcus bromii	WISC sum	Species	0.183	0.758		
Meconium Adj.[c]	Ruminococcus bromii	WISC sum	Interaction	0.018	0.091		
Meconium Adj.[c]	Ruminococcus lactaris	Block Design	Caffeine	-0.35	0.253	0.798	0.994
Meconium Adj.[c]	Ruminococcus lactaris	Block Design	Species	-0.005	1.842		

Exposure Window	Species	Outcome	Variable	Effect Estimate[a]	Std. Error	LRT[a]	
						p-value	q-value
Meconium Adj.[c]	Ruminococcus lactaris	Block Design	Interaction	0.047	0.203		
Meconium Adj.[c]	Ruminococcus lactaris	Coding	Caffeine	0.138	0.227	0.681	0.975
Meconium Adj.[c]	Ruminococcus lactaris	Coding	Species	0.654	1.656		
Meconium Adj.[c]	Ruminococcus lactaris	Coding	Interaction	-0.068	0.183		
Meconium Adj.[c]	Ruminococcus lactaris	Digit span	Caffeine	-0.212	0.188	0.312	0.941
Meconium Adj.[c]	Ruminococcus lactaris	Digit span	Species	0.994	1.368		
Meconium Adj.[c]	Ruminococcus lactaris	Digit span	Interaction	-0.139	0.151		
Meconium Adj.[c]	Ruminococcus lactaris	Information	Caffeine	-0.302	0.183	0.037	0.777
Meconium Adj.[c]	Ruminococcus lactaris	Information	Species	-2.495	1.332		
Meconium Adj.[c]	Ruminococcus lactaris	Information	Interaction	0.283	0.147		
Meconium Adj.[c]	Ruminococcus lactaris	QTAC	Caffeine	-0.504	0.659	0.363	0.971
Meconium Adj.[c]	Ruminococcus lactaris	QTAC	Species	3.938	4.801		
Meconium Adj.[c]	Ruminococcus lactaris	QTAC	Interaction	-0.437	0.53		
Meconium Adj.[c]	Ruminococcus lactaris	Vocabulary	Caffeine	-0.366	0.284	0.11	0.844
Meconium Adj.[c]	Ruminococcus lactaris	Vocabulary	Species	-2.833	2.069		
Meconium Adj.[c]	Ruminococcus lactaris	Vocabulary	Interaction	0.334	0.228		
Meconium Adj.[c]	Ruminococcus lactaris	WISC sum	Caffeine	-1.092	0.647	0.332	0.963
Meconium Adj.[c]	Ruminococcus lactaris	WISC sum	Species	-3.685	4.718		
Meconium Adj.[c]	Ruminococcus lactaris	WISC sum	Interaction	0.458	0.521		
Meconium Adj.[c]	Ruminococcus torques	Block Design	Caffeine	-0.122	0.394	0.529	0.975
Meconium Adj.[c]	Ruminococcus torques	Block Design	Species	0.699	0.803		
Meconium Adj.[c]	Ruminococcus torques	Block Design	Interaction	-0.053	0.092		
Meconium Adj.[c]	Ruminococcus torques	Coding	Caffeine	0.006	0.35	0.684	0.975
Meconium Adj.[c]	Ruminococcus torques	Coding	Species	-0.465	0.713		
Meconium Adj.[c]	Ruminococcus torques	Coding	Interaction	0.03	0.082		
Meconium Adj.[c]	Ruminococcus torques	Digit span	Caffeine	-0.571	0.282	0.201	0.844
Meconium Adj.[c]	Ruminococcus torques	Digit span	Species	-0.914	0.575		
Meconium Adj.[c]	Ruminococcus torques	Digit span	Interaction	0.077	0.066		
Meconium Adj.[c]	Ruminococcus torques	Information	Caffeine	-0.205	0.299	0.703	0.975
Meconium Adj.[c]	Ruminococcus torques	Information	Species	-0.272	0.61		
Meconium Adj.[c]	Ruminococcus torques	Information	Interaction	0.024	0.07		
Meconium Adj.[c]	Ruminococcus torques	QTAC	Caffeine	-1.863	1.009	0.172	0.844
Meconium Adj.[c]	Ruminococcus torques	QTAC	Species	-1.977	2.056		
Meconium Adj.[c]	Ruminococcus torques	QTAC	Interaction	0.294	0.236		
Meconium Adj.[c]	Ruminococcus torques	Vocabulary	Caffeine	-0.448	0.456	0.383	0.971
Meconium Adj.[c]	Ruminococcus torques	Vocabulary	Species	-0.736	0.93		

Exposure Window	Species	Outcome	Variable	Effect Estimate[a]	Std. Error	LRT[a]	
						p-value	q-value
Meconium Adj.[c]	Ruminococcus torques	Vocabulary	Interaction	0.084	0.107		
Meconium Adj.[c]	Ruminococcus torques	WISC sum	Caffeine	-1.34	1.02	0.451	0.975
Meconium Adj.[c]	Ruminococcus torques	WISC sum	Species	-1.686	2.079		
Meconium Adj.[c]	Ruminococcus torques	WISC sum	Interaction	0.163	0.239		
Meconium Adj.[c]	Streptococcus thermophilus	Block Design	Caffeine	-0.537	0.27	0.084	0.844
Meconium Adj.[c]	Streptococcus thermophilus	Block Design	Species	-2.351	1.68		
Meconium Adj.[c]	Streptococcus thermophilus	Block Design	Interaction	0.316	0.199		
Meconium Adj.[c]	Streptococcus thermophilus	Coding	Caffeine	-0.015	0.238	0.512	0.975
Meconium Adj.[c]	Streptococcus thermophilus	Coding	Species	-1.336	1.481		
Meconium Adj.[c]	Streptococcus thermophilus	Coding	Interaction	0.104	0.175		
Meconium Adj.[c]	Streptococcus thermophilus	Digit span	Caffeine	-0.227	0.205	0.523	0.975
Meconium Adj.[c]	Streptococcus thermophilus	Digit span	Species	0.992	1.275		
Meconium Adj.[c]	Streptococcus thermophilus	Digit span	Interaction	-0.087	0.151		
Meconium Adj.[c]	Streptococcus thermophilus	Information	Caffeine	0.089	0.199	0.075	0.808
Meconium Adj.[c]	Streptococcus thermophilus	Information	Species	2.232	1.237		
Meconium Adj.[c]	Streptococcus thermophilus	Information	Interaction	-0.24	0.147		
Meconium Adj.[c]	Streptococcus thermophilus	QTAC	Caffeine	-0.902	0.607	0.914	0.994
Meconium Adj.[c]	Streptococcus thermophilus	QTAC	Species	-2.479	3.775		
Meconium Adj.[c]	Streptococcus thermophilus	QTAC	Interaction	-0.044	0.447		
Meconium Adj.[c]	Streptococcus thermophilus	Vocabulary	Caffeine	-0.073	0.307	0.815	0.994
Meconium Adj.[c]	Streptococcus thermophilus	Vocabulary	Species	1	1.907		
Meconium Adj.[c]	Streptococcus thermophilus	Vocabulary	Interaction	-0.048	0.226		
Meconium Adj.[c]	Streptococcus thermophilus	WISC sum	Caffeine	-0.763	0.702	0.923	0.994
Meconium Adj.[c]	Streptococcus thermophilus	WISC sum	Species	0.539	4.369		
Meconium Adj.[c]	Streptococcus thermophilus	WISC sum	Interaction	0.045	0.518		

[a] Models are adjusted for whether the child was ever breastfed, sex, mode of birth, and socioeconomic status. Effect estimates for caffeine are the change in outcome score for each doubling of exposure. Effect estimates for species are the change in outcome score for each 1% increase in species relative abundance. Effect estimates for the interaction are for the multiplicative interaction between acetaminophen and species relative abundance.

[b] Likelihood ratio test comparing to model without interaction

[c] Meconium Adj. includes cross-sectional exposure in the model

Table S11. Acetaminophen-species interactions in associations with neurodevelopment

Exposure Window	Species	Outcome	Variable	Effect Estimate[a]	Std. Error	LRT[b]	
						p-value	q-value
Meconium	Agathobaculum butyriciproducens	Block Design	Acetaminophen	0.127	1.164	0.2308	0.764
Meconium	Agathobaculum butyriciproducens	Block Design	Species	-0.937	0.547		
Meconium	Agathobaculum butyriciproducens	Block Design	Interaction	1.015	0.933		
Meconium	Agathobaculum butyriciproducens	Coding	Acetaminophen	-0.089	1.05	0.487	0.813
Meconium	Agathobaculum butyriciproducens	Coding	Species	-0.457	0.493		
Meconium	Agathobaculum butyriciproducens	Coding	Interaction	0.537	0.842		
Meconium	Agathobaculum butyriciproducens	Digit span	Acetaminophen	-0.787	0.914	0.356	0.795
Meconium	Agathobaculum butyriciproducens	Digit span	Species	-0.055	0.43		
Meconium	Agathobaculum butyriciproducens	Digit span	Interaction	0.622	0.733		
Meconium	Agathobaculum butyriciproducens	Information	Acetaminophen	-0.128	0.881	0.911	0.968
Meconium	Agathobaculum butyriciproducens	Information	Species	0.546	0.414		
Meconium	Agathobaculum butyriciproducens	Information	Interaction	-0.072	0.706		
Meconium	Agathobaculum butyriciproducens	QTAC	Acetaminophen	-3.347	3.148	0.957	0.978
Meconium	Agathobaculum butyriciproducens	QTAC	Species	-1.348	1.479		
Meconium	Agathobaculum butyriciproducens	QTAC	Interaction	0.126	2.524		
Meconium	Agathobaculum butyriciproducens	Vocabulary	Acetaminophen	1.095	1.348	0.325	0.774
Meconium	Agathobaculum butyriciproducens	Vocabulary	Species	0.726	0.634		
Meconium	Agathobaculum butyriciproducens	Vocabulary	Interaction	-0.978	1.081		
Meconium	Agathobaculum butyriciproducens	WISC sum	Acetaminophen	0.219	3.131	0.625	0.842
Meconium	Agathobaculum butyriciproducens	WISC sum	Species	-0.176	1.471		
Meconium	Agathobaculum butyriciproducens	WISC sum	Interaction	1.123	2.51		
Meconium	Akkermansia muciniphila	Block Design	Acetaminophen	1.076	1.004	0.634	0.842
Meconium	Akkermansia muciniphila	Block Design	Species	0.119	0.273		
Meconium	Akkermansia muciniphila	Block Design	Interaction	-0.146	0.335		
Meconium	Akkermansia muciniphila	Coding	Acetaminophen	0.156	0.884	0.686	0.872
Meconium	Akkermansia muciniphila	Coding	Species	-0.03	0.24		
Meconium	Akkermansia muciniphila	Coding	Interaction	0.109	0.295		
Meconium	Akkermansia muciniphila	Digit span	Acetaminophen	0.062	0.761	0.416	0.813
Meconium	Akkermansia muciniphila	Digit span	Species	0.027	0.207		
Meconium	Akkermansia muciniphila	Digit span	Interaction	-0.19	0.254		
Meconium	Akkermansia muciniphila	Information	Acetaminophen	-0.28	0.759	0.698	0.872
Meconium	Akkermansia muciniphila	Information	Species	-0.033	0.206		
Meconium	Akkermansia muciniphila	Information	Interaction	0.09	0.253		
Meconium	Akkermansia muciniphila	QTAC	Acetaminophen	-1.491	2.492	0.222	0.744
Meconium	Akkermansia muciniphila	QTAC	Species	-0.169	0.677		

Exposure Window	Species	Outcome	Variable	Effect Estimate[a]	Std. Error	LRT[b]	
						p-value	q-value
Meconium	Akkermansia muciniphila	QTAC	Interaction	-0.935	0.832		
Meconium	Akkermansia muciniphila	Vocabulary	Acetaminophen	0.596	1.141	0.606	0.842
Meconium	Akkermansia muciniphila	Vocabulary	Species	0.065	0.31		
Meconium	Akkermansia muciniphila	Vocabulary	Interaction	-0.18	0.381		
Meconium	Akkermansia muciniphila	WISC sum	Acetaminophen	1.61	2.62	0.693	0.872
Meconium	Akkermansia muciniphila	WISC sum	Species	0.149	0.711		
Meconium	Akkermansia muciniphila	WISC sum	Interaction	-0.316	0.874		
Meconium	Alistipes putredinis	Block Design	Acetaminophen	1.026	1.09	0.929	0.978
Meconium	Alistipes putredinis	Block Design	Species	-0.078	0.935		
Meconium	Alistipes putredinis	Block Design	Interaction	-0.087	1.061		
Meconium	Alistipes putredinis	Coding	Acetaminophen	0.612	0.923	0.253	0.77
Meconium	Alistipes putredinis	Coding	Species	1.271	0.792		
Meconium	Alistipes putredinis	Coding	Interaction	-0.945	0.899		
Meconium	Alistipes putredinis	Digit span	Acetaminophen	0.214	0.824	0.391	0.813
Meconium	Alistipes putredinis	Digit span	Species	0.209	0.707		
Meconium	Alistipes putredinis	Digit span	Interaction	-0.631	0.802		
Meconium	Alistipes putredinis	Information	Acetaminophen	-0.398	0.791	0.982	0.986
Meconium	Alistipes putredinis	Information	Species	0.533	0.679		
Meconium	Alistipes putredinis	Information	Interaction	0.016	0.77		
Meconium	Alistipes putredinis	QTAC	Acetaminophen	-2.884	2.886	0.62	0.842
Meconium	Alistipes putredinis	QTAC	Species	1.516	2.477		
Meconium	Alistipes putredinis	QTAC	Interaction	-1.275	2.809		
Meconium	Alistipes putredinis	Vocabulary	Acetaminophen	0.747	1.234	0.441	0.813
Meconium	Alistipes putredinis	Vocabulary	Species	0.763	1.059		
Meconium	Alistipes putredinis	Vocabulary	Interaction	-0.849	1.201		
Meconium	Alistipes putredinis	WISC sum	Acetaminophen	2.201	2.802	0.32	0.774
Meconium	Alistipes putredinis	WISC sum	Species	2.699	2.405		
Meconium	Alistipes putredinis	WISC sum	Interaction	-2.496	2.727		
Meconium	Anaerostipes hadrus	Block Design	Acetaminophen	-0.545	1.746	0.289	0.774
Meconium	Anaerostipes hadrus	Block Design	Species	-0.012	0.139		
Meconium	Anaerostipes hadrus	Block Design	Interaction	0.279	0.286		
Meconium	Anaerostipes hadrus	Coding	Acetaminophen	0.253	1.535	0.941	0.978
Meconium	Anaerostipes hadrus	Coding	Species	0.117	0.122		
Meconium	Anaerostipes hadrus	Coding	Interaction	0.017	0.252		
Meconium	Anaerostipes hadrus	Digit span	Acetaminophen	0.33	1.355	0.58	0.835
Meconium	Anaerostipes hadrus	Digit span	Species	0.048	0.108		

Exposure Window	Species	Outcome	Variable	Effect Estimate[a]	Std. Error	LRT[b]	
						p-value	q-value
Meconium	Anaerostipes hadrus	Digit span	Interaction	-0.113	0.222		
Meconium	Anaerostipes hadrus	Information	Acetaminophen	1.047	1.307	0.253	0.77
Meconium	Anaerostipes hadrus	Information	Species	0.133	0.104		
Meconium	Anaerostipes hadrus	Information	Interaction	-0.226	0.214		
Meconium	Anaerostipes hadrus	QTAC	Acetaminophen	-8.59	4.559	0.146	0.634
Meconium	Anaerostipes hadrus	QTAC	Species	0.055	0.363		
Meconium	Anaerostipes hadrus	QTAC	Interaction	1.006	0.748		
Meconium	Anaerostipes hadrus	Vocabulary	Acetaminophen	1.962	1.97	0.296	0.774
Meconium	Anaerostipes hadrus	Vocabulary	Species	0.201	0.157		
Meconium	Anaerostipes hadrus	Vocabulary	Interaction	-0.311	0.323		
Meconium	Anaerostipes hadrus	WISC sum	Acetaminophen	3.046	4.512	0.602	0.842
Meconium	Anaerostipes hadrus	WISC sum	Species	0.488	0.359		
Meconium	Anaerostipes hadrus	WISC sum	Interaction	-0.353	0.74		
Meconium	Asaccharobacter celatus	Block Design	Acetaminophen	1.256	1.257	0.622	0.842
Meconium	Asaccharobacter celatus	Block Design	Species	-0.253	0.524		
Meconium	Asaccharobacter celatus	Block Design	Interaction	-0.641	1.42		
Meconium	Asaccharobacter celatus	Coding	Acetaminophen	0.827	1.109	0.473	0.813
Meconium	Asaccharobacter celatus	Coding	Species	0.033	0.462		
Meconium	Asaccharobacter celatus	Coding	Interaction	-0.825	1.253		
Meconium	Asaccharobacter celatus	Digit span	Acetaminophen	0.196	0.966	0.486	0.813
Meconium	Asaccharobacter celatus	Digit span	Species	0.284	0.403		
Meconium	Asaccharobacter celatus	Digit span	Interaction	-0.697	1.091		
Meconium	Asaccharobacter celatus	Information	Acetaminophen	-0.46	0.953	0.584	0.835
Meconium	Asaccharobacter celatus	Information	Species	0.04	0.397		
Meconium	Asaccharobacter celatus	Information	Interaction	0.541	1.077		
Meconium	Asaccharobacter celatus	QTAC	Acetaminophen	-4.94	3.334	0.427	0.813
Meconium	Asaccharobacter celatus	QTAC	Species	0.345	1.389		
Meconium	Asaccharobacter celatus	QTAC	Interaction	2.746	3.766		
Meconium	Asaccharobacter celatus	Vocabulary	Acetaminophen	0.801	1.437	0.612	0.842
Meconium	Asaccharobacter celatus	Vocabulary	Species	0.182	0.599		
Meconium	Asaccharobacter celatus	Vocabulary	Interaction	-0.754	1.623		
Meconium	Asaccharobacter celatus	WISC sum	Acetaminophen	2.621	3.286	0.485	0.813
Meconium	Asaccharobacter celatus	WISC sum	Species	0.285	1.37		
Meconium	Asaccharobacter celatus	WISC sum	Interaction	-2.377	3.713		
Meconium	Bacteroides vulgatus	Block Design	Acetaminophen	1.479	1.034	0.187	0.734
Meconium	Bacteroides vulgatus	Block Design	Species	0.824	0.753		

Exposure Window	Species	Outcome	Variable	Effect Estimate[a]	Std. Error	LRT[b]	
						p-value	q-value
Meconium	Bacteroides vulgatus	Block Design	Interaction	-1.171	0.961		
Meconium	Bacteroides vulgatus	Coding	Acetaminophen	0.33	0.899	0.574	0.835
Meconium	Bacteroides vulgatus	Coding	Species	0.89	0.655		
Meconium	Bacteroides vulgatus	Coding	Interaction	-0.43	0.835		
Meconium	Bacteroides vulgatus	Digit span	Acetaminophen	-0.173	0.804	0.636	0.842
Meconium	Bacteroides vulgatus	Digit span	Species	0.417	0.586		
Meconium	Bacteroides vulgatus	Digit span	Interaction	-0.324	0.747		
Meconium	Bacteroides vulgatus	Information	Acetaminophen	-0.033	0.791	0.623	0.842
Meconium	Bacteroides vulgatus	Information	Species	0.376	0.576		
Meconium	Bacteroides vulgatus	Information	Interaction	-0.331	0.735		
Meconium	Bacteroides vulgatus	QTAC	Acetaminophen	-6.018	2.623	0.105	0.634
Meconium	Bacteroides vulgatus	QTAC	Species	-0.058	1.91		
Meconium	Bacteroides vulgatus	QTAC	Interaction	3.66	2.438		
Meconium	Bacteroides vulgatus	Vocabulary	Acetaminophen	0.182	1.197	0.776	0.891
Meconium	Bacteroides vulgatus	Vocabulary	Species	-0.201	0.872		
Meconium	Bacteroides vulgatus	Vocabulary	Interaction	0.289	1.112		
Meconium	Bacteroides vulgatus	WISC sum	Acetaminophen	1.784	2.699	0.393	0.813
Meconium	Bacteroides vulgatus	WISC sum	Species	2.306	1.966		
Meconium	Bacteroides vulgatus	WISC sum	Interaction	-1.966	2.508		
Meconium	Bifidobacterium adolescentis	Block Design	Acetaminophen	2.29	1.107	0.035	0.622
Meconium	Bifidobacterium adolescentis	Block Design	Species	0.077	0.085		
Meconium	Bifidobacterium adolescentis	Block Design	Interaction	-0.257	0.13		
Meconium	Bifidobacterium adolescentis	Coding	Acetaminophen	-0.626	0.987	0.088	0.634
Meconium	Bifidobacterium adolescentis	Coding	Species	-0.125	0.076		
Meconium	Bifidobacterium adolescentis	Coding	Interaction	0.184	0.116		
Meconium	Bifidobacterium adolescentis	Digit span	Acetaminophen	0.887	0.846	0.021	0.613
Meconium	Bifidobacterium adolescentis	Digit span	Species	0.08	0.065		
Meconium	Bifidobacterium adolescentis	Digit span	Interaction	-0.215	0.1		
Meconium	Bifidobacterium adolescentis	Information	Acetaminophen	0.225	0.869	0.478	0.813
Meconium	Bifidobacterium adolescentis	Information	Species	-0.005	0.067		
Meconium	Bifidobacterium adolescentis	Information	Interaction	-0.067	0.102		
Meconium	Bifidobacterium adolescentis	QTAC	Acetaminophen	-1.559	2.995	0.324	0.774
Meconium	Bifidobacterium adolescentis	QTAC	Species	-0.082	0.231		
Meconium	Bifidobacterium adolescentis	QTAC	Interaction	-0.32	0.353		
Meconium	Bifidobacterium adolescentis	Vocabulary	Acetaminophen	1.115	1.263	0.31	0.774
Meconium	Bifidobacterium adolescentis	Vocabulary	Species	-0.063	0.097		

Exposure Window	Species	Outcome	Variable	Effect Estimate[a]	Std. Error	LRT[b]	
						p-value	q-value
Meconium	Bifidobacterium adolescentis	Vocabulary	Interaction	-0.139	0.149		
Meconium	Bifidobacterium adolescentis	WISC sum	Acetaminophen	3.891	2.871	0.115	0.634
Meconium	Bifidobacterium adolescentis	WISC sum	Species	-0.037	0.221		
Meconium	Bifidobacterium adolescentis	WISC sum	Interaction	-0.494	0.338		
Meconium	Bifidobacterium animalis	Block Design	Acetaminophen	0.65	0.886	0.276	0.774
Meconium	Bifidobacterium animalis	Block Design	Species	0.206	0.196		
Meconium	Bifidobacterium animalis	Block Design	Interaction	0.354	0.353		
Meconium	Bifidobacterium animalis	Coding	Acetaminophen	0.21	0.819	0.558	0.829
Meconium	Bifidobacterium animalis	Coding	Species	0.031	0.182		
Meconium	Bifidobacterium animalis	Coding	Interaction	0.175	0.326		
Meconium	Bifidobacterium animalis	Digit span	Acetaminophen	-0.251	0.72	0.964	0.978
Meconium	Bifidobacterium animalis	Digit span	Species	-0.026	0.16		
Meconium	Bifidobacterium animalis	Digit span	Interaction	-0.012	0.287		
Meconium	Bifidobacterium animalis	Information	Acetaminophen	-0.245	0.704	0.552	0.829
Meconium	Bifidobacterium animalis	Information	Species	0.011	0.156		
Meconium	Bifidobacterium animalis	Information	Interaction	0.153	0.28		
Meconium	Bifidobacterium animalis	QTAC	Acetaminophen	-3.304	2.485	0.894	0.958
Meconium	Bifidobacterium animalis	QTAC	Species	0.213	0.551		
Meconium	Bifidobacterium animalis	QTAC	Interaction	-0.121	0.99		
Meconium	Bifidobacterium animalis	Vocabulary	Acetaminophen	0.063	1.043	0.321	0.774
Meconium	Bifidobacterium animalis	Vocabulary	Species	0.073	0.231		
Meconium	Bifidobacterium animalis	Vocabulary	Interaction	0.379	0.416		
Meconium	Bifidobacterium animalis	WISC sum	Acetaminophen	0.426	2.348	0.224	0.744
Meconium	Bifidobacterium animalis	WISC sum	Species	0.294	0.521		
Meconium	Bifidobacterium animalis	WISC sum	Interaction	1.049	0.936		
Meconium	Bifidobacterium bifidum	Block Design	Acetaminophen	1.076	1.014	0.702	0.872
Meconium	Bifidobacterium bifidum	Block Design	Species	0.061	0.171		
Meconium	Bifidobacterium bifidum	Block Design	Interaction	-0.077	0.219		
Meconium	Bifidobacterium bifidum	Coding	Acetaminophen	0.049	0.861	0.545	0.829
Meconium	Bifidobacterium bifidum	Coding	Species	-0.21	0.146		
Meconium	Bifidobacterium bifidum	Coding	Interaction	0.103	0.186		
Meconium	Bifidobacterium bifidum	Digit span	Acetaminophen	-0.086	0.767	0.493	0.813
Meconium	Bifidobacterium bifidum	Digit span	Species	-0.012	0.13		
Meconium	Bifidobacterium bifidum	Digit span	Interaction	-0.104	0.165		
Meconium	Bifidobacterium bifidum	Information	Acetaminophen	-0.099	0.731	0.71	0.872
Meconium	Bifidobacterium bifidum	Information	Species	-0.108	0.124		

Exposure Window	Species	Outcome	Variable	Effect Estimate[a]	Std. Error	LRT[b]	
						p-value	q-value
Meconium	Bifidobacterium bifidum	Information	Interaction	-0.054	0.158		
Meconium	Bifidobacterium bifidum	QTAC	Acetaminophen	-3.676	2.692	0.741	0.877
Meconium	Bifidobacterium bifidum	QTAC	Species	-0.027	0.455		
Meconium	Bifidobacterium bifidum	QTAC	Interaction	0.176	0.581		
Meconium	Bifidobacterium bifidum	Vocabulary	Acetaminophen	0.357	1.138	0.843	0.92
Meconium	Bifidobacterium bifidum	Vocabulary	Species	-0.094	0.192		
Meconium	Bifidobacterium bifidum	Vocabulary	Interaction	-0.044	0.245		
Meconium	Bifidobacterium bifidum	WISC sum	Acetaminophen	1.297	2.531	0.725	0.872
Meconium	Bifidobacterium bifidum	WISC sum	Species	-0.364	0.428		
Meconium	Bifidobacterium bifidum	WISC sum	Interaction	-0.176	0.546		
Meconium	Bifidobacterium longum	Block Design	Acetaminophen	-0.893	1.278	0.034	0.622
Meconium	Bifidobacterium longum	Block Design	Species	-0.043	0.057		
Meconium	Bifidobacterium longum	Block Design	Interaction	0.241	0.121		
Meconium	Bifidobacterium longum	Coding	Acetaminophen	-0.11	1.175	0.561	0.829
Meconium	Bifidobacterium longum	Coding	Species	-0.009	0.052		
Meconium	Bifidobacterium longum	Coding	Interaction	0.059	0.112		
Meconium	Bifidobacterium longum	Digit span	Acetaminophen	-0.89	1.015	0.442	0.813
Meconium	Bifidobacterium longum	Digit span	Species	-0.047	0.045		
Meconium	Bifidobacterium longum	Digit span	Interaction	0.068	0.096		
Meconium	Bifidobacterium longum	Information	Acetaminophen	-1.254	0.983	0.093	0.634
Meconium	Bifidobacterium longum	Information	Species	-0.032	0.044		
Meconium	Bifidobacterium longum	Information	Interaction	0.145	0.093		
Meconium	Bifidobacterium longum	QTAC	Acetaminophen	-1.287	3.425	0.576	0.835
Meconium	Bifidobacterium longum	QTAC	Species	0.268	0.152		
Meconium	Bifidobacterium longum	QTAC	Interaction	-0.167	0.325		
Meconium	Bifidobacterium longum	Vocabulary	Acetaminophen	-0.975	1.48	0.141	0.634
Meconium	Bifidobacterium longum	Vocabulary	Species	0.004	0.066		
Meconium	Bifidobacterium longum	Vocabulary	Interaction	0.191	0.141		
Meconium	Bifidobacterium longum	WISC sum	Acetaminophen	-4.122	3.291	0.017	0.613
Meconium	Bifidobacterium longum	WISC sum	Species	-0.127	0.146		
Meconium	Bifidobacterium longum	WISC sum	Interaction	0.706	0.313		
Meconium	Bifidobacterium pseudocatenulatum	Block Design	Acetaminophen	0.557	1.191	0.654	0.855
Meconium	Bifidobacterium pseudocatenulatum	Block Design	Species	-0.056	0.114		
Meconium	Bifidobacterium pseudocatenulatum	Block Design	Interaction	0.165	0.401		
Meconium	Bifidobacterium pseudocatenulatum	Coding	Acetaminophen	1.33	1.018	0.13	0.634
Meconium	Bifidobacterium pseudocatenulatum	Coding	Species	0.129	0.097		

Exposure Window	Species	Outcome	Variable	Effect Estimate[a]	Std. Error	LRT[b]	
						p-value	q-value
Meconium	Bifidobacterium pseudocatenulatum	Coding	Interaction	-0.48	0.343		
Meconium	Bifidobacterium pseudocatenulatum	Digit span	Acetaminophen	-1.082	0.896	0.147	0.634
Meconium	Bifidobacterium pseudocatenulatum	Digit span	Species	-0.083	0.085		
Meconium	Bifidobacterium pseudocatenulatum	Digit span	Interaction	0.405	0.302		
Meconium	Bifidobacterium pseudocatenulatum	Information	Acetaminophen	-0.175	0.903	0.964	0.978
Meconium	Bifidobacterium pseudocatenulatum	Information	Species	-0.01	0.086		
Meconium	Bifidobacterium pseudocatenulatum	Information	Interaction	0.012	0.304		
Meconium	Bifidobacterium pseudocatenulatum	QTAC	Acetaminophen	-3.321	3.101	0.851	0.92
Meconium	Bifidobacterium pseudocatenulatum	QTAC	Species	0.38	0.296		
Meconium	Bifidobacterium pseudocatenulatum	QTAC	Interaction	0.18	1.044		
Meconium	Bifidobacterium pseudocatenulatum	Vocabulary	Acetaminophen	-0.841	1.324	0.122	0.634
Meconium	Bifidobacterium pseudocatenulatum	Vocabulary	Species	0.002	0.126		
Meconium	Bifidobacterium pseudocatenulatum	Vocabulary	Interaction	0.637	0.446		
Meconium	Bifidobacterium pseudocatenulatum	WISC sum	Acetaminophen	-0.211	3.097	0.439	0.813
Meconium	Bifidobacterium pseudocatenulatum	WISC sum	Species	-0.019	0.295		
Meconium	Bifidobacterium pseudocatenulatum	WISC sum	Interaction	0.74	1.043		
Meconium	Blautia obeum	Block Design	Acetaminophen	-0.22	1.221	0.16	0.68
Meconium	Blautia obeum	Block Design	Species	-0.101	0.152		
Meconium	Blautia obeum	Block Design	Interaction	0.623	0.479		
Meconium	Blautia obeum	Coding	Acetaminophen	1.433	1.05	0.215	0.742
Meconium	Blautia obeum	Coding	Species	0.237	0.131		
Meconium	Blautia obeum	Coding	Interaction	-0.472	0.413		
Meconium	Blautia obeum	Digit span	Acetaminophen	-0.34	0.912	0.459	0.813
Meconium	Blautia obeum	Digit span	Species	0.199	0.114		
Meconium	Blautia obeum	Digit span	Interaction	0.243	0.358		
Meconium	Blautia obeum	Information	Acetaminophen	0.024	0.934	0.977	0.985
Meconium	Blautia obeum	Information	Species	0.099	0.116		
Meconium	Blautia obeum	Information	Interaction	-0.01	0.367		
Meconium	Blautia obeum	QTAC	Acetaminophen	-5.821	3.24	0.357	0.795
Meconium	Blautia obeum	QTAC	Species	-0.508	0.403		
Meconium	Blautia obeum	QTAC	Interaction	1.076	1.272		
Meconium	Blautia obeum	Vocabulary	Acetaminophen	-0.889	1.373	0.098	0.634
Meconium	Blautia obeum	Vocabulary	Species	0.057	0.171		
Meconium	Blautia obeum	Vocabulary	Interaction	0.828	0.539		
Meconium	Blautia obeum	WISC sum	Acetaminophen	0.008	3.129	0.284	0.774
Meconium	Blautia obeum	WISC sum	Species	0.491	0.389		

Exposure Window	Species	Outcome	Variable	Effect Estimate[a]	Std. Error	LRT[b]	
						p-value	q-value
Meconium	Blautia obeum	WISC sum	Interaction	1.213	1.229		
Meconium	Blautia wexlerae	Block Design	Acetaminophen	1.456	1.118	0.371	0.8
Meconium	Blautia wexlerae	Block Design	Species	0.195	0.303		
Meconium	Blautia wexlerae	Block Design	Interaction	-0.311	0.379		
Meconium	Blautia wexlerae	Coding	Acetaminophen	1.007	0.959	0.2	0.734
Meconium	Blautia wexlerae	Coding	Species	0.033	0.26		
Meconium	Blautia wexlerae	Coding	Interaction	-0.384	0.325		
Meconium	Blautia wexlerae	Digit span	Acetaminophen	-1.056	0.839	0.085	0.634
Meconium	Blautia wexlerae	Digit span	Species	-0.347	0.228		
Meconium	Blautia wexlerae	Digit span	Interaction	0.455	0.284		
Meconium	Blautia wexlerae	Information	Acetaminophen	0.216	0.794	0.395	0.813
Meconium	Blautia wexlerae	Information	Species	0.453	0.215		
Meconium	Blautia wexlerae	Information	Interaction	-0.21	0.269		
Meconium	Blautia wexlerae	QTAC	Acetaminophen	-5.064	2.948	0.285	0.774
Meconium	Blautia wexlerae	QTAC	Species	-0.909	0.8		
Meconium	Blautia wexlerae	QTAC	Interaction	0.982	0.999		
Meconium	Blautia wexlerae	Vocabulary	Acetaminophen	1.384	1.207	0.106	0.634
Meconium	Blautia wexlerae	Vocabulary	Species	0.745	0.327		
Meconium	Blautia wexlerae	Vocabulary	Interaction	-0.612	0.409		
Meconium	Blautia wexlerae	WISC sum	Acetaminophen	3.006	2.873	0.236	0.764
Meconium	Blautia wexlerae	WISC sum	Species	1.079	0.78		
Meconium	Blautia wexlerae	WISC sum	Interaction	-1.062	0.973		
Meconium	Collinsella aerofaciens	Block Design	Acetaminophen	0.631	1.328	0.747	0.88
Meconium	Collinsella aerofaciens	Block Design	Species	-0.033	0.095		
Meconium	Collinsella aerofaciens	Block Design	Interaction	0.033	0.113		
Meconium	Collinsella aerofaciens	Coding	Acetaminophen	-0.419	1.158	0.331	0.774
Meconium	Collinsella aerofaciens	Coding	Species	-0.072	0.083		
Meconium	Collinsella aerofaciens	Coding	Interaction	0.088	0.098		
Meconium	Collinsella aerofaciens	Digit span	Acetaminophen	-1.321	0.992	0.111	0.634
Meconium	Collinsella aerofaciens	Digit span	Species	-0.106	0.071		
Meconium	Collinsella aerofaciens	Digit span	Interaction	0.124	0.084		
Meconium	Collinsella aerofaciens	Information	Acetaminophen	-0.381	1.003	0.725	0.872
Meconium	Collinsella aerofaciens	Information	Species	-0.009	0.072		
Meconium	Collinsella aerofaciens	Information	Interaction	0.027	0.085		
Meconium	Collinsella aerofaciens	QTAC	Acetaminophen	-1.988	3.52	0.564	0.829
Meconium	Collinsella aerofaciens	QTAC	Species	0.08	0.252		

Exposure Window	Species	Outcome	Variable	Effect Estimate[a]	Std. Error	LRT[b]	
						p-value	q-value
Meconium	Collinsella aerofaciens	QTAC	Interaction	-0.158	0.299		
Meconium	Collinsella aerofaciens	Vocabulary	Acetaminophen	-0.261	1.508	0.562	0.829
Meconium	Collinsella aerofaciens	Vocabulary	Species	-0.044	0.108		
Meconium	Collinsella aerofaciens	Vocabulary	Interaction	0.068	0.128		
Meconium	Collinsella aerofaciens	WISC sum	Acetaminophen	-1.751	3.405	0.201	0.734
Meconium	Collinsella aerofaciens	WISC sum	Species	-0.265	0.243		
Meconium	Collinsella aerofaciens	WISC sum	Interaction	0.341	0.289		
Meconium	Coprococcus catus	Block Design	Acetaminophen	0.912	1.421	0.992	0.992
Meconium	Coprococcus catus	Block Design	Species	-0.418	0.855		
Meconium	Coprococcus catus	Block Design	Interaction	-0.014	1.584		
Meconium	Coprococcus catus	Coding	Acetaminophen	1.621	1.228	0.141	0.634
Meconium	Coprococcus catus	Coding	Species	0.588	0.739		
Meconium	Coprococcus catus	Coding	Interaction	-1.862	1.369		
Meconium	Coprococcus catus	Digit span	Acetaminophen	-0.627	1.091	0.643	0.846
Meconium	Coprococcus catus	Digit span	Species	-0.436	0.656		
Meconium	Coprococcus catus	Digit span	Interaction	0.516	1.216		
Meconium	Coprococcus catus	Information	Acetaminophen	-1.882	1.018	0.019	0.613
Meconium	Coprococcus catus	Information	Species	-0.696	0.613		
Meconium	Coprococcus catus	Information	Interaction	2.512	1.135		
Meconium	Coprococcus catus	QTAC	Acetaminophen	0.813	3.476	0.102	0.634
Meconium	Coprococcus catus	QTAC	Species	5.789	2.091		
Meconium	Coprococcus catus	QTAC	Interaction	-5.878	3.874		
Meconium	Coprococcus catus	Vocabulary	Acetaminophen	-0.798	1.607	0.324	0.774
Meconium	Coprococcus catus	Vocabulary	Species	-0.318	0.967		
Meconium	Coprococcus catus	Vocabulary	Interaction	1.623	1.791		
Meconium	Coprococcus catus	WISC sum	Acetaminophen	-0.773	3.695	0.463	0.813
Meconium	Coprococcus catus	WISC sum	Species	-1.28	2.223		
Meconium	Coprococcus catus	WISC sum	Interaction	2.774	4.119		
Meconium	Coprococcus comes	Block Design	Acetaminophen	0.311	1.318	0.492	0.813
Meconium	Coprococcus comes	Block Design	Species	-0.186	0.4		
Meconium	Coprococcus comes	Block Design	Interaction	0.406	0.645		
Meconium	Coprococcus comes	Coding	Acetaminophen	1.448	1.127	0.134	0.634
Meconium	Coprococcus comes	Coding	Species	0.56	0.342		
Meconium	Coprococcus comes	Coding	Interaction	-0.765	0.551		
Meconium	Coprococcus comes	Digit span	Acetaminophen	-0.777	1.006	0.456	0.813
Meconium	Coprococcus comes	Digit span	Species	0.049	0.305		

Exposure Window	Species	Outcome	Variable	Effect Estimate[a]	Std. Error	LRT[b]	
						p-value	q-value
Meconium	Coprococcus comes	Digit span	Interaction	0.337	0.492		
Meconium	Coprococcus comes	Information	Acetaminophen	-0.898	0.987	0.251	0.77
Meconium	Coprococcus comes	Information	Species	-0.243	0.299		
Meconium	Coprococcus comes	Information	Interaction	0.51	0.483		
Meconium	Coprococcus comes	QTAC	Acetaminophen	-4.29	3.366	0.716	0.872
Meconium	Coprococcus comes	QTAC	Species	1.33	1.021		
Meconium	Coprococcus comes	QTAC	Interaction	0.549	1.646		
Meconium	Coprococcus comes	Vocabulary	Acetaminophen	-0.49	1.483	0.426	0.813
Meconium	Coprococcus comes	Vocabulary	Species	0.11	0.45		
Meconium	Coprococcus comes	Vocabulary	Interaction	0.53	0.725		
Meconium	Coprococcus comes	WISC sum	Acetaminophen	-0.407	3.409	0.506	0.819
Meconium	Coprococcus comes	WISC sum	Species	0.29	1.034		
Meconium	Coprococcus comes	WISC sum	Interaction	1.018	1.667		
Meconium	Coprococcus eutactus	Block Design	Acetaminophen	0.869	1.011	0.622	0.842
Meconium	Coprococcus eutactus	Block Design	Species	-0.359	0.461		
Meconium	Coprococcus eutactus	Block Design	Interaction	0.243	0.537		
Meconium	Coprococcus eutactus	Coding	Acetaminophen	0.346	0.89	0.711	0.872
Meconium	Coprococcus eutactus	Coding	Species	-0.295	0.405		
Meconium	Coprococcus eutactus	Coding	Interaction	0.161	0.473		
Meconium	Coprococcus eutactus	Digit span	Acetaminophen	-0.562	0.777	0.417	0.813
Meconium	Coprococcus eutactus	Digit span	Species	-0.141	0.354		
Meconium	Coprococcus eutactus	Digit span	Interaction	0.307	0.413		
Meconium	Coprococcus eutactus	Information	Acetaminophen	0.119	0.765	0.467	0.813
Meconium	Coprococcus eutactus	Information	Species	0.127	0.349		
Meconium	Coprococcus eutactus	Information	Interaction	-0.271	0.407		
Meconium	Coprococcus eutactus	QTAC	Acetaminophen	-2.183	2.68	0.33	0.774
Meconium	Coprococcus eutactus	QTAC	Species	0.677	1.221		
Meconium	Coprococcus eutactus	QTAC	Interaction	-1.276	1.424		
Meconium	Coprococcus eutactus	Vocabulary	Acetaminophen	0.857	1.145	0.357	0.795
Meconium	Coprococcus eutactus	Vocabulary	Species	0.194	0.522		
Meconium	Coprococcus eutactus	Vocabulary	Interaction	-0.514	0.609		
Meconium	Coprococcus eutactus	WISC sum	Acetaminophen	1.629	2.639	0.953	0.978
Meconium	Coprococcus eutactus	WISC sum	Species	-0.473	1.202		
Meconium	Coprococcus eutactus	WISC sum	Interaction	-0.075	1.402		
Meconium	Dorea formicigenerans	Block Design	Acetaminophen	2.066	1.592	0.364	0.795
Meconium	Dorea formicigenerans	Block Design	Species	1.008	0.884		

Exposure Window	Species	Outcome	Variable	Effect Estimate[a]	Std. Error	LRT[b]	
						p-value	q-value
Meconium	Dorea formicigenerans	Block Design	Interaction	-1.311	1.572		
Meconium	Dorea formicigenerans	Coding	Acetaminophen	-0.833	1.391	0.298	0.774
Meconium	Dorea formicigenerans	Coding	Species	-1.078	0.773		
Meconium	Dorea formicigenerans	Coding	Interaction	1.315	1.374		
Meconium	Dorea formicigenerans	Digit span	Acetaminophen	-2.162	1.119	0.039	0.622
Meconium	Dorea formicigenerans	Digit span	Species	-1.921	0.622		
Meconium	Dorea formicigenerans	Digit span	Interaction	2.129	1.105		
Meconium	Dorea formicigenerans	Information	Acetaminophen	-1.1	1.21	0.299	0.774
Meconium	Dorea formicigenerans	Information	Species	-0.364	0.672		
Meconium	Dorea formicigenerans	Information	Interaction	1.141	1.194		
Meconium	Dorea formicigenerans	QTAC	Acetaminophen	-8.13	4.204	0.141	0.634
Meconium	Dorea formicigenerans	QTAC	Species	-2.13	2.334		
Meconium	Dorea formicigenerans	QTAC	Interaction	5.658	4.151		
Meconium	Dorea formicigenerans	Vocabulary	Acetaminophen	-0.143	1.839	0.733	0.872
Meconium	Dorea formicigenerans	Vocabulary	Species	-0.071	1.021		
Meconium	Dorea formicigenerans	Vocabulary	Interaction	0.568	1.816		
Meconium	Dorea formicigenerans	WISC sum	Acetaminophen	-2.173	4.154	0.308	0.774
Meconium	Dorea formicigenerans	WISC sum	Species	-2.427	2.307		
Meconium	Dorea formicigenerans	WISC sum	Interaction	3.842	4.102		
Meconium	Dorea longicatena	Block Design	Acetaminophen	2.202	1.538	0.255	0.77
Meconium	Dorea longicatena	Block Design	Species	0.121	0.286		
Meconium	Dorea longicatena	Block Design	Interaction	-0.493	0.471		
Meconium	Dorea longicatena	Coding	Acetaminophen	0.245	1.368	0.959	0.978
Meconium	Dorea longicatena	Coding	Species	-0.128	0.254		
Meconium	Dorea longicatena	Coding	Interaction	0.019	0.419		
Meconium	Dorea longicatena	Digit span	Acetaminophen	-1.275	1.168	0.271	0.774
Meconium	Dorea longicatena	Digit span	Species	-0.323	0.217		
Meconium	Dorea longicatena	Digit span	Interaction	0.362	0.358		
Meconium	Dorea longicatena	Information	Acetaminophen	-1.439	1.129	0.111	0.634
Meconium	Dorea longicatena	Information	Species	0.009	0.21		
Meconium	Dorea longicatena	Information	Interaction	0.51	0.346		
Meconium	Dorea longicatena	QTAC	Acetaminophen	-8.254	4.02	0.113	0.634
Meconium	Dorea longicatena	QTAC	Species	-1.071	0.747		
Meconium	Dorea longicatena	QTAC	Interaction	1.807	1.231		
Meconium	Dorea longicatena	Vocabulary	Acetaminophen	0.089	1.7	0.773	0.891
Meconium	Dorea longicatena	Vocabulary	Species	0.417	0.316		

Exposure Window	Species	Outcome	Variable	Effect Estimate[a]	Std. Error	LRT[b]	
						p-value	q-value
Meconium	Dorea longicatena	Vocabulary	Interaction	0.138	0.52		
Meconium	Dorea longicatena	WISC sum	Acetaminophen	-0.179	4.046	0.636	0.842
Meconium	Dorea longicatena	WISC sum	Species	0.096	0.752		
Meconium	Dorea longicatena	WISC sum	Interaction	0.536	1.239		
Meconium	Eubacterium hallii	Block Design	Acetaminophen	1.972	1.829	0.489	0.813
Meconium	Eubacterium hallii	Block Design	Species	0.331	0.403		
Meconium	Eubacterium hallii	Block Design	Interaction	-0.458	0.723		
Meconium	Eubacterium hallii	Coding	Acetaminophen	-0.382	1.593	0.629	0.842
Meconium	Eubacterium hallii	Coding	Species	-0.43	0.351		
Meconium	Eubacterium hallii	Coding	Interaction	0.278	0.629		
Meconium	Eubacterium hallii	Digit span	Acetaminophen	1.433	1.366	0.142	0.634
Meconium	Eubacterium hallii	Digit span	Species	0.525	0.301		
Meconium	Eubacterium hallii	Digit span	Interaction	-0.733	0.54		
Meconium	Eubacterium hallii	Information	Acetaminophen	0.6	1.38	0.528	0.829
Meconium	Eubacterium hallii	Information	Species	0.275	0.304		
Meconium	Eubacterium hallii	Information	Interaction	-0.315	0.545		
Meconium	Eubacterium hallii	QTAC	Acetaminophen	-7.094	4.854	0.334	0.774
Meconium	Eubacterium hallii	QTAC	Species	-0.549	1.071		
Meconium	Eubacterium hallii	QTAC	Interaction	1.703	1.918		
Meconium	Eubacterium hallii	Vocabulary	Acetaminophen	0.386	2.061	0.966	0.978
Meconium	Eubacterium hallii	Vocabulary	Species	0.439	0.455		
Meconium	Eubacterium hallii	Vocabulary	Interaction	0.032	0.814		
Meconium	Eubacterium hallii	WISC sum	Acetaminophen	4.01	4.741	0.486	0.813
Meconium	Eubacterium hallii	WISC sum	Species	1.14	1.046		
Meconium	Eubacterium hallii	WISC sum	Interaction	-1.196	1.873		
Meconium	Eubacterium rectale	Block Design	Acetaminophen	1.12	1.116	0.52	0.829
Meconium	Eubacterium rectale	Block Design	Species	-0.03	0.085		
Meconium	Eubacterium rectale	Block Design	Interaction	-0.213	0.361		
Meconium	Eubacterium rectale	Coding	Acetaminophen	0.304	0.99	0.964	0.978
Meconium	Eubacterium rectale	Coding	Species	-0.013	0.076		
Meconium	Eubacterium rectale	Coding	Interaction	-0.013	0.32		
Meconium	Eubacterium rectale	Digit span	Acetaminophen	-0.292	0.861	0.761	0.888
Meconium	Eubacterium rectale	Digit span	Species	0.025	0.066		
Meconium	Eubacterium rectale	Digit span	Interaction	0.078	0.279		
Meconium	Eubacterium rectale	Information	Acetaminophen	0.555	0.792	0.02	0.613
Meconium	Eubacterium rectale	Information	Species	-0.035	0.06		

Exposure Window	Species	Outcome	Variable	Effect Estimate[a]	Std. Error	LRT[b]	
						p-value	q-value
Meconium	Eubacterium rectale	Information	Interaction	-0.561	0.256		
Meconium	Eubacterium rectale	QTAC	Acetaminophen	-5.512	2.93	0.263	0.771
Meconium	Eubacterium rectale	QTAC	Species	-0.221	0.224		
Meconium	Eubacterium rectale	QTAC	Interaction	0.978	0.948		
Meconium	Eubacterium rectale	Vocabulary	Acetaminophen	0.237	1.234	0.498	0.813
Meconium	Eubacterium rectale	Vocabulary	Species	-0.134	0.094		
Meconium	Eubacterium rectale	Vocabulary	Interaction	-0.248	0.399		
Meconium	Eubacterium rectale	WISC sum	Acetaminophen	1.924	2.848	0.259	0.77
Meconium	Eubacterium rectale	WISC sum	Species	-0.188	0.217		
Meconium	Eubacterium rectale	WISC sum	Interaction	-0.958	0.921		
Meconium	Eubacterium siraeum	Block Design	Acetaminophen	0.99	0.953	0.48	0.813
Meconium	Eubacterium siraeum	Block Design	Species	-0.511	0.319		
Meconium	Eubacterium siraeum	Block Design	Interaction	-0.685	1.058		
Meconium	Eubacterium siraeum	Coding	Acetaminophen	0.484	0.875	0.668	0.868
Meconium	Eubacterium siraeum	Coding	Species	0.087	0.292		
Meconium	Eubacterium siraeum	Coding	Interaction	-0.381	0.971		
Meconium	Eubacterium siraeum	Digit span	Acetaminophen	-0.108	0.763	0.562	0.829
Meconium	Eubacterium siraeum	Digit span	Species	0.012	0.255		
Meconium	Eubacterium siraeum	Digit span	Interaction	-0.45	0.847		
Meconium	Eubacterium siraeum	Information	Acetaminophen	-0.245	0.751	0.671	0.868
Meconium	Eubacterium siraeum	Information	Species	0.021	0.251		
Meconium	Eubacterium siraeum	Information	Interaction	0.324	0.833		
Meconium	Eubacterium siraeum	QTAC	Acetaminophen	-3.918	2.631	0.509	0.819
Meconium	Eubacterium siraeum	QTAC	Species	0.103	0.879		
Meconium	Eubacterium siraeum	QTAC	Interaction	1.767	2.92		
Meconium	Eubacterium siraeum	Vocabulary	Acetaminophen	0.847	1.093	0.364	0.795
Meconium	Eubacterium siraeum	Vocabulary	Species	0.615	0.365		
Meconium	Eubacterium siraeum	Vocabulary	Interaction	-1.011	1.213		
Meconium	Eubacterium siraeum	WISC sum	Acetaminophen	1.968	2.577	0.401	0.813
Meconium	Eubacterium siraeum	WISC sum	Species	0.225	0.861		
Meconium	Eubacterium siraeum	WISC sum	Interaction	-2.204	2.86		
Meconium	Eubacterium sp CAG 180	Block Design	Acetaminophen	0.242	0.926	0.042	0.622
Meconium	Eubacterium sp CAG 180	Block Design	Species	-0.9	0.373		
Meconium	Eubacterium sp CAG 180	Block Design	Interaction	1.161	0.611		
Meconium	Eubacterium sp CAG 180	Coding	Acetaminophen	-0.093	0.853	0.168	0.702
Meconium	Eubacterium sp CAG 180	Coding	Species	-0.432	0.344		

Exposure Window	Species	Outcome	Variable	Effect Estimate[a]	Std. Error	LRT[b]	
						p-value	q-value
Meconium	Eubacterium sp CAG 180	Coding	Interaction	0.717	0.563		
Meconium	Eubacterium sp CAG 180	Digit span	Acetaminophen	-0.974	0.696	0.01	0.613
Meconium	Eubacterium sp CAG 180	Digit span	Species	-0.088	0.28		
Meconium	Eubacterium sp CAG 180	Digit span	Interaction	1.128	0.459		
Meconium	Eubacterium sp CAG 180	Information	Acetaminophen	-0.445	0.676	0.31	0.774
Meconium	Eubacterium sp CAG 180	Information	Species	0.472	0.272		
Meconium	Eubacterium sp CAG 180	Information	Interaction	0.417	0.446		
Meconium	Eubacterium sp CAG 180	QTAC	Acetaminophen	-4.562	2.593	0.207	0.734
Meconium	Eubacterium sp CAG 180	QTAC	Species	-0.802	1.045		
Meconium	Eubacterium sp CAG 180	QTAC	Interaction	1.994	1.712		
Meconium	Eubacterium sp CAG 180	Vocabulary	Acetaminophen	-0.497	1.058	0.049	0.623
Meconium	Eubacterium sp CAG 180	Vocabulary	Species	0.031	0.426		
Meconium	Eubacterium sp CAG 180	Vocabulary	Interaction	1.283	0.698		
Meconium	Eubacterium sp CAG 180	WISC sum	Acetaminophen	-1.768	2.298	0.001	0.311
Meconium	Eubacterium sp CAG 180	WISC sum	Species	-0.917	0.926		
Meconium	Eubacterium sp CAG 180	WISC sum	Interaction	4.706	1.517		
Meconium	Faecalibacterium prausnitzii	Block Design	Acetaminophen	2.172	1.604	0.308	0.774
Meconium	Faecalibacterium prausnitzii	Block Design	Species	0.036	0.104		
Meconium	Faecalibacterium prausnitzii	Block Design	Interaction	-0.178	0.189		
Meconium	Faecalibacterium prausnitzii	Coding	Acetaminophen	-0.757	1.388	0.335	0.774
Meconium	Faecalibacterium prausnitzii	Coding	Species	0.048	0.09		
Meconium	Faecalibacterium prausnitzii	Coding	Interaction	0.145	0.164		
Meconium	Faecalibacterium prausnitzii	Digit span	Acetaminophen	0.862	1.222	0.215	0.742
Meconium	Faecalibacterium prausnitzii	Digit span	Species	0.095	0.079		
Meconium	Faecalibacterium prausnitzii	Digit span	Interaction	-0.165	0.144		
Meconium	Faecalibacterium prausnitzii	Information	Acetaminophen	1.575	1.166	0.064	0.634
Meconium	Faecalibacterium prausnitzii	Information	Species	0.006	0.076		
Meconium	Faecalibacterium prausnitzii	Information	Interaction	-0.238	0.138		
Meconium	Faecalibacterium prausnitzii	QTAC	Acetaminophen	-10.044	4.106	0.038	0.622
Meconium	Faecalibacterium prausnitzii	QTAC	Species	-0.162	0.267		
Meconium	Faecalibacterium prausnitzii	QTAC	Interaction	0.94	0.485		
Meconium	Faecalibacterium prausnitzii	Vocabulary	Acetaminophen	2.832	1.748	0.073	0.634
Meconium	Faecalibacterium prausnitzii	Vocabulary	Species	-0.016	0.114		
Meconium	Faecalibacterium prausnitzii	Vocabulary	Interaction	-0.345	0.206		
Meconium	Faecalibacterium prausnitzii	WISC sum	Acetaminophen	6.684	4.095	0.083	0.634
Meconium	Faecalibacterium prausnitzii	WISC sum	Species	0.168	0.266		

Exposure Window	Species	Outcome	Variable	Effect Estimate[a]	Std. Error	LRT[b]	
						p-value	q-value
Meconium	Faecalibacterium prausnitzii	WISC sum	Interaction	-0.779	0.484		
Meconium	Fusicatenibacter saccharivorans	Block Design	Acetaminophen	1.257	1.689	0.815	0.92
Meconium	Fusicatenibacter saccharivorans	Block Design	Species	-0.014	0.154		
Meconium	Fusicatenibacter saccharivorans	Block Design	Interaction	-0.046	0.216		
Meconium	Fusicatenibacter saccharivorans	Coding	Acetaminophen	1.32	1.472	0.415	0.813
Meconium	Fusicatenibacter saccharivorans	Coding	Species	0.018	0.134		
Meconium	Fusicatenibacter saccharivorans	Coding	Interaction	-0.141	0.188		
Meconium	Fusicatenibacter saccharivorans	Digit span	Acetaminophen	-0.921	1.29	0.48	0.813
Meconium	Fusicatenibacter saccharivorans	Digit span	Species	-0.103	0.118		
Meconium	Fusicatenibacter saccharivorans	Digit span	Interaction	0.107	0.165		
Meconium	Fusicatenibacter saccharivorans	Information	Acetaminophen	1.121	1.258	0.198	0.734
Meconium	Fusicatenibacter saccharivorans	Information	Species	0.101	0.115		
Meconium	Fusicatenibacter saccharivorans	Information	Interaction	-0.191	0.161		
Meconium	Fusicatenibacter saccharivorans	QTAC	Acetaminophen	-8.305	4.377	0.134	0.634
Meconium	Fusicatenibacter saccharivorans	QTAC	Species	-0.59	0.399		
Meconium	Fusicatenibacter saccharivorans	QTAC	Interaction	0.777	0.56		
Meconium	Fusicatenibacter saccharivorans	Vocabulary	Acetaminophen	0.822	1.9	0.677	0.87
Meconium	Fusicatenibacter saccharivorans	Vocabulary	Species	0.161	0.173		
Meconium	Fusicatenibacter saccharivorans	Vocabulary	Interaction	-0.093	0.243		
Meconium	Fusicatenibacter saccharivorans	WISC sum	Acetaminophen	3.598	4.392	0.479	0.813
Meconium	Fusicatenibacter saccharivorans	WISC sum	Species	0.162	0.401		
Meconium	Fusicatenibacter saccharivorans	WISC sum	Interaction	-0.364	0.562		
Meconium	Methanobrevibacter smithii	Block Design	Acetaminophen	1.056	0.989	0.779	0.891
Meconium	Methanobrevibacter smithii	Block Design	Species	0.217	0.268		
Meconium	Methanobrevibacter smithii	Block Design	Interaction	-0.122	0.474		
Meconium	Methanobrevibacter smithii	Coding	Acetaminophen	-0.125	0.852	0.132	0.634
Meconium	Methanobrevibacter smithii	Coding	Species	-0.05	0.231		
Meconium	Methanobrevibacter smithii	Coding	Interaction	0.57	0.409		
Meconium	Methanobrevibacter smithii	Digit span	Acetaminophen	-0.601	0.751	0.204	0.734
Meconium	Methanobrevibacter smithii	Digit span	Species	-0.044	0.204		
Meconium	Methanobrevibacter smithii	Digit span	Interaction	0.422	0.36		
Meconium	Methanobrevibacter smithii	Information	Acetaminophen	-0.615	0.727	0.076	0.634
Meconium	Methanobrevibacter smithii	Information	Species	-0.105	0.197		
Meconium	Methanobrevibacter smithii	Information	Interaction	0.576	0.349		
Meconium	Methanobrevibacter smithii	QTAC	Acetaminophen	-3.792	2.593	0.542	0.829
Meconium	Methanobrevibacter smithii	QTAC	Species	0.46	0.704		

Exposure Window	Species	Outcome	Variable	Effect Estimate[a]	Std. Error	LRT[b]	
						p-value	q-value
Meconium	Methanobrevibacter smithii	QTAC	Interaction	0.695	1.244		
Meconium	Methanobrevibacter smithii	Vocabulary	Acetaminophen	0.024	1.103	0.373	0.8
Meconium	Methanobrevibacter smithii	Vocabulary	Species	0.168	0.3		
Meconium	Methanobrevibacter smithii	Vocabulary	Interaction	0.433	0.529		
Meconium	Methanobrevibacter smithii	WISC sum	Acetaminophen	-0.261	2.459	0.086	0.634
Meconium	Methanobrevibacter smithii	WISC sum	Species	0.186	0.668		
Meconium	Methanobrevibacter smithii	WISC sum	Interaction	1.879	1.18		
Meconium	Prevotella copri	Block Design	Acetaminophen	0.838	0.982	0.819	0.92
Meconium	Prevotella copri	Block Design	Species	-0.072	0.336		
Meconium	Prevotella copri	Block Design	Interaction	0.126	0.604		
Meconium	Prevotella copri	Coding	Acetaminophen	0.112	0.86	0.476	0.813
Meconium	Prevotella copri	Coding	Species	-0.063	0.295		
Meconium	Prevotella copri	Coding	Interaction	0.346	0.529		
Meconium	Prevotella copri	Digit span	Acetaminophen	-0.179	0.736	0.846	0.92
Meconium	Prevotella copri	Digit span	Species	-0.284	0.252		
Meconium	Prevotella copri	Digit span	Interaction	-0.08	0.453		
Meconium	Prevotella copri	Information	Acetaminophen	-0.641	0.711	0.044	0.622
Meconium	Prevotella copri	Information	Species	-0.335	0.243		
Meconium	Prevotella copri	Information	Interaction	0.821	0.437		
Meconium	Prevotella copri	QTAC	Acetaminophen	-2.519	2.567	0.318	0.774
Meconium	Prevotella copri	QTAC	Species	0.979	0.879		
Meconium	Prevotella copri	QTAC	Interaction	-1.449	1.58		
Meconium	Prevotella copri	Vocabulary	Acetaminophen	0.484	1.113	0.708	0.872
Meconium	Prevotella copri	Vocabulary	Species	-0.071	0.381		
Meconium	Prevotella copri	Vocabulary	Interaction	-0.235	0.685		
Meconium	Prevotella copri	WISC sum	Acetaminophen	0.614	2.533	0.494	0.813
Meconium	Prevotella copri	WISC sum	Species	-0.824	0.867		
Meconium	Prevotella copri	WISC sum	Interaction	0.978	1.559		
Meconium	Roseburia faecis	Block Design	Acetaminophen	0.521	1.055	0.44	0.813
Meconium	Roseburia faecis	Block Design	Species	-0.205	0.253		
Meconium	Roseburia faecis	Block Design	Interaction	0.219	0.309		
Meconium	Roseburia faecis	Coding	Acetaminophen	0.232	0.909	0.836	0.92
Meconium	Roseburia faecis	Coding	Species	-0.204	0.218		
Meconium	Roseburia faecis	Coding	Interaction	0.05	0.266		
Meconium	Roseburia faecis	Digit span	Acetaminophen	-0.397	0.808	0.719	0.872
Meconium	Roseburia faecis	Digit span	Species	0.033	0.193		

Exposure Window	Species	Outcome	Variable	Effect Estimate[a]	Std. Error	LRT[b]	
						p-value	q-value
Meconium	Roseburia faecis	Digit span	Interaction	0.078	0.236		
Meconium	Roseburia faecis	Information	Acetaminophen	-0.045	0.79	0.784	0.893
Meconium	Roseburia faecis	Information	Species	-0.065	0.189		
Meconium	Roseburia faecis	Information	Interaction	-0.058	0.231		
Meconium	Roseburia faecis	QTAC	Acetaminophen	-2.179	2.752	0.363	0.795
Meconium	Roseburia faecis	QTAC	Species	0.079	0.659		
Meconium	Roseburia faecis	QTAC	Interaction	-0.673	0.805		
Meconium	Roseburia faecis	Vocabulary	Acetaminophen	-0.836	1.144	0.038	0.622
Meconium	Roseburia faecis	Vocabulary	Species	-0.601	0.274		
Meconium	Roseburia faecis	Vocabulary	Interaction	0.649	0.335		
Meconium	Roseburia faecis	WISC sum	Acetaminophen	-0.525	2.681	0.195	0.734
Meconium	Roseburia faecis	WISC sum	Species	-1.041	0.642		
Meconium	Roseburia faecis	WISC sum	Interaction	0.938	0.784		
Meconium	Roseburia intestinalis	Block Design	Acetaminophen	1.02	1.018	0.77	0.891
Meconium	Roseburia intestinalis	Block Design	Species	0.341	0.621		
Meconium	Roseburia intestinalis	Block Design	Interaction	-0.223	0.835		
Meconium	Roseburia intestinalis	Coding	Acetaminophen	0.932	0.876	0.11	0.634
Meconium	Roseburia intestinalis	Coding	Species	0.526	0.535		
Meconium	Roseburia intestinalis	Coding	Interaction	-1.066	0.719		
Meconium	Roseburia intestinalis	Digit span	Acetaminophen	-0.128	0.74	0.551	0.829
Meconium	Roseburia intestinalis	Digit span	Species	0.829	0.451		
Meconium	Roseburia intestinalis	Digit span	Interaction	-0.332	0.607		
Meconium	Roseburia intestinalis	Information	Acetaminophen	0.471	0.738	0.06	0.634
Meconium	Roseburia intestinalis	Information	Species	0.357	0.45		
Meconium	Roseburia intestinalis	Information	Interaction	-1.063	0.606		
Meconium	Roseburia intestinalis	QTAC	Acetaminophen	-3.576	2.684	0.789	0.894
Meconium	Roseburia intestinalis	QTAC	Species	-1.321	1.637		
Meconium	Roseburia intestinalis	QTAC	Interaction	0.54	2.202		
Meconium	Roseburia intestinalis	Vocabulary	Acetaminophen	0.277	1.163	0.924	0.978
Meconium	Roseburia intestinalis	Vocabulary	Species	-0.051	0.709		
Meconium	Roseburia intestinalis	Vocabulary	Interaction	0.083	0.954		
Meconium	Roseburia intestinalis	WISC sum	Acetaminophen	2.573	2.612	0.188	0.734
Meconium	Roseburia intestinalis	WISC sum	Species	2.001	1.593		
Meconium	Roseburia intestinalis	WISC sum	Interaction	-2.601	2.143		
Meconium	Ruminococcus bicirculans	Block Design	Acetaminophen	1.132	1.093	0.711	0.872
Meconium	Ruminococcus bicirculans	Block Design	Species	-0.049	0.34		

Exposure Window	Species	Outcome	Variable	Effect Estimate[a]	Std. Error	LRT[b]	
						p-value	q-value
Meconium	Ruminococcus bicirculans	Block Design	Interaction	-0.185	0.546		
Meconium	Ruminococcus bicirculans	Coding	Acetaminophen	-0.716	0.919	0.029	0.622
Meconium	Ruminococcus bicirculans	Coding	Species	-0.39	0.286		
Meconium	Ruminococcus bicirculans	Coding	Interaction	0.941	0.458		
Meconium	Ruminococcus bicirculans	Digit span	Acetaminophen	0.036	0.837	0.472	0.813
Meconium	Ruminococcus bicirculans	Digit span	Species	0.182	0.26		
Meconium	Ruminococcus bicirculans	Digit span	Interaction	-0.275	0.418		
Meconium	Ruminococcus bicirculans	Information	Acetaminophen	-0.418	0.823	0.499	0.813
Meconium	Ruminococcus bicirculans	Information	Species	-0.178	0.256		
Meconium	Ruminococcus bicirculans	Information	Interaction	0.255	0.411		
Meconium	Ruminococcus bicirculans	QTAC	Acetaminophen	-5.182	2.864	0.225	0.744
Meconium	Ruminococcus bicirculans	QTAC	Species	-0.214	0.891		
Meconium	Ruminococcus bicirculans	QTAC	Interaction	1.598	1.429		
Meconium	Ruminococcus bicirculans	Vocabulary	Acetaminophen	-0.708	1.149	0.065	0.634
Meconium	Ruminococcus bicirculans	Vocabulary	Species	-0.962	0.358		
Meconium	Ruminococcus bicirculans	Vocabulary	Interaction	0.984	0.574		
Meconium	Ruminococcus bicirculans	WISC sum	Acetaminophen	-0.673	2.765	0.178	0.728
Meconium	Ruminococcus bicirculans	WISC sum	Species	-1.397	0.861		
Meconium	Ruminococcus bicirculans	WISC sum	Interaction	1.718	1.38		
Meconium	Ruminococcus bromii	Block Design	Acetaminophen	-0.965	1.48	0.111	0.634
Meconium	Ruminococcus bromii	Block Design	Species	-0.04	0.087		
Meconium	Ruminococcus bromii	Block Design	Interaction	0.202	0.136		
Meconium	Ruminococcus bromii	Coding	Acetaminophen	-0.34	1.314	0.621	0.842
Meconium	Ruminococcus bromii	Coding	Species	0.052	0.077		
Meconium	Ruminococcus bromii	Coding	Interaction	0.055	0.121		
Meconium	Ruminococcus bromii	Digit span	Acetaminophen	-1.543	1.119	0.191	0.734
Meconium	Ruminococcus bromii	Digit span	Species	0.024	0.066		
Meconium	Ruminococcus bromii	Digit span	Interaction	0.124	0.103		
Meconium	Ruminococcus bromii	Information	Acetaminophen	-2.215	1.076	0.016	0.613
Meconium	Ruminococcus bromii	Information	Species	-0.051	0.063		
Meconium	Ruminococcus bromii	Information	Interaction	0.224	0.099		
Meconium	Ruminococcus bromii	QTAC	Acetaminophen	-1.646	4.053	0.554	0.829
Meconium	Ruminococcus bromii	QTAC	Species	0.108	0.238		
Meconium	Ruminococcus bromii	QTAC	Interaction	-0.202	0.373		
Meconium	Ruminococcus bromii	Vocabulary	Acetaminophen	-2.261	1.659	0.05	0.623
Meconium	Ruminococcus bromii	Vocabulary	Species	-0.063	0.098		

Exposure Window	Species	Outcome	Variable	Effect Estimate[a]	Std. Error	LRT[b]	
						p-value	q-value
Meconium	Ruminococcus bromii	Vocabulary	Interaction	0.28	0.153		
Meconium	Ruminococcus bromii	WISC sum	Acetaminophen	-7.324	3.501	0.004	0.481
Meconium	Ruminococcus bromii	WISC sum	Species	-0.078	0.206		
Meconium	Ruminococcus bromii	WISC sum	Interaction	0.885	0.322		
Meconium	Ruminococcus lactaris	Block Design	Acetaminophen	0.753	1.072	0.731	0.872
Meconium	Ruminococcus lactaris	Block Design	Species	0.319	0.367		
Meconium	Ruminococcus lactaris	Block Design	Interaction	0.224	0.71		
Meconium	Ruminococcus lactaris	Coding	Acetaminophen	0.435	0.959	0.837	0.92
Meconium	Ruminococcus lactaris	Coding	Species	0.097	0.328		
Meconium	Ruminococcus lactaris	Coding	Interaction	-0.12	0.635		
Meconium	Ruminococcus lactaris	Digit span	Acetaminophen	-0.892	0.806	0.141	0.634
Meconium	Ruminococcus lactaris	Digit span	Species	-0.472	0.276		
Meconium	Ruminococcus lactaris	Digit span	Interaction	0.728	0.534		
Meconium	Ruminococcus lactaris	Information	Acetaminophen	0.586	0.798	0.076	0.634
Meconium	Ruminococcus lactaris	Information	Species	0.233	0.273		
Meconium	Ruminococcus lactaris	Information	Interaction	-0.872	0.529		
Meconium	Ruminococcus lactaris	QTAC	Acetaminophen	-6.604	2.748	0.023	0.613
Meconium	Ruminococcus lactaris	QTAC	Species	-1.093	0.941		
Meconium	Ruminococcus lactaris	QTAC	Interaction	3.884	1.821		
Meconium	Ruminococcus lactaris	Vocabulary	Acetaminophen	0.462	1.238	0.837	0.92
Meconium	Ruminococcus lactaris	Vocabulary	Species	0.171	0.424		
Meconium	Ruminococcus lactaris	Vocabulary	Interaction	-0.155	0.82		
Meconium	Ruminococcus lactaris	WISC sum	Acetaminophen	1.345	2.838	0.91	0.968
Meconium	Ruminococcus lactaris	WISC sum	Species	0.348	0.972		
Meconium	Ruminococcus lactaris	WISC sum	Interaction	-0.195	1.881		
Meconium	Ruminococcus torques	Block Design	Acetaminophen	2.651	1.418	0.101	0.634
Meconium	Ruminococcus torques	Block Design	Species	0.439	0.21		
Meconium	Ruminococcus torques	Block Design	Interaction	-0.521	0.343		
Meconium	Ruminococcus torques	Coding	Acetaminophen	0.594	1.286	0.758	0.888
Meconium	Ruminococcus torques	Coding	Species	-0.166	0.191		
Meconium	Ruminococcus torques	Coding	Interaction	-0.088	0.311		
Meconium	Ruminococcus torques	Digit span	Acetaminophen	-0.144	1.087	0.848	0.92
Meconium	Ruminococcus torques	Digit span	Species	-0.257	0.161		
Meconium	Ruminococcus torques	Digit span	Interaction	-0.046	0.263		
Meconium	Ruminococcus torques	Information	Acetaminophen	0.148	1.122	0.714	0.872
Meconium	Ruminococcus torques	Information	Species	-0.034	0.167		

Exposure Window	Species	Outcome	Variable	Effect Estimate[a]	Std. Error	LRT[b]	
						p-value	q-value
Meconium	Ruminococcus torques	Information	Interaction	-0.091	0.271		
Meconium	Ruminococcus torques	QTAC	Acetaminophen	-7.787	3.818	0.11	0.634
Meconium	Ruminococcus torques	QTAC	Species	-0.063	0.567		
Meconium	Ruminococcus torques	QTAC	Interaction	1.367	0.923		
Meconium	Ruminococcus torques	Vocabulary	Acetaminophen	1.031	1.692	0.564	0.829
Meconium	Ruminococcus torques	Vocabulary	Species	0.064	0.251		
Meconium	Ruminococcus torques	Vocabulary	Interaction	-0.216	0.409		
Meconium	Ruminococcus torques	WISC sum	Acetaminophen	4.279	3.818	0.258	0.77
Meconium	Ruminococcus torques	WISC sum	Species	0.047	0.567		
Meconium	Ruminococcus torques	WISC sum	Interaction	-0.962	0.923		
Meconium	Streptococcus thermophilus	Block Design	Acetaminophen	1.402	1.157	0.56	0.829
Meconium	Streptococcus thermophilus	Block Design	Species	0.565	0.517		
Meconium	Streptococcus thermophilus	Block Design	Interaction	-0.348	0.652		
Meconium	Streptococcus thermophilus	Coding	Acetaminophen	0.181	1.007	0.965	0.978
Meconium	Streptococcus thermophilus	Coding	Species	-0.443	0.45		
Meconium	Streptococcus thermophilus	Coding	Interaction	0.023	0.567		
Meconium	Streptococcus thermophilus	Digit span	Acetaminophen	-1.022	0.866	0.098	0.634
Meconium	Streptococcus thermophilus	Digit span	Species	-0.193	0.387		
Meconium	Streptococcus thermophilus	Digit span	Interaction	0.749	0.488		
Meconium	Streptococcus thermophilus	Information	Acetaminophen	0.35	0.868	0.418	0.813
Meconium	Streptococcus thermophilus	Information	Species	0.526	0.387		
Meconium	Streptococcus thermophilus	Information	Interaction	-0.363	0.489		
Meconium	Streptococcus thermophilus	QTAC	Acetaminophen	-3.981	2.579	0.864	0.931
Meconium	Streptococcus thermophilus	QTAC	Species	-2.908	1.152		
Meconium	Streptococcus thermophilus	QTAC	Interaction	-0.227	1.452		
Meconium	Streptococcus thermophilus	Vocabulary	Acetaminophen	0.921	1.278	0.586	0.835
Meconium	Streptococcus thermophilus	Vocabulary	Species	0.902	0.571		
Meconium	Streptococcus thermophilus	Vocabulary	Interaction	-0.359	0.72		
Meconium	Streptococcus thermophilus	WISC sum	Acetaminophen	1.832	2.995	0.847	0.92
Meconium	Streptococcus thermophilus	WISC sum	Species	1.358	1.337		
Meconium	Streptococcus thermophilus	WISC sum	Interaction	-0.299	1.686		
Meconium Adj.[c]	Agathobaculum butyriciproducens	Block Design	Acetaminophen	0.092	1.189	0.234	0.735
Meconium Adj.[c]	Agathobaculum butyriciproducens	Block Design	Species	-0.942	0.554		
Meconium Adj.[c]	Agathobaculum butyriciproducens	Block Design	Interaction	0.197	0.929		
Meconium Adj.[c]	Agathobaculum butyriciproducens	Coding	Acetaminophen	-0.196	1.066	0.463	0.836
Meconium Adj.[c]	Agathobaculum butyriciproducens	Coding	Species	-0.472	0.497		

Exposure Window	Species	Outcome	Variable	Effect Estimate[a]	Std. Error	LRT[b]	
						p-value	q-value
Meconium Adj.[c]	Agathobaculum butyriciproducens	Coding	Interaction	0.608	0.833		
Meconium Adj.[c]	Agathobaculum butyriciproducens	Digit span	Acetaminophen	-0.769	0.935	0.36	0.79
Meconium Adj.[c]	Agathobaculum butyriciproducens	Digit span	Species	-0.052	0.435		
Meconium Adj.[c]	Agathobaculum butyriciproducens	Digit span	Interaction	-0.103	0.73		
Meconium Adj.[c]	Agathobaculum butyriciproducens	Information	Acetaminophen	-0.303	0.878	0.964	0.985
Meconium Adj.[c]	Agathobaculum butyriciproducens	Information	Species	0.521	0.409		
Meconium Adj.[c]	Agathobaculum butyriciproducens	Information	Interaction	0.991	0.686		
Meconium Adj.[c]	Agathobaculum butyriciproducens	QTAC	Acetaminophen	-2.607	3.105	0.979	0.992
Meconium Adj.[c]	Agathobaculum butyriciproducens	QTAC	Species	-1.245	1.447		
Meconium Adj.[c]	Agathobaculum butyriciproducens	QTAC	Interaction	-4.174	2.426		
Meconium Adj.[c]	Agathobaculum butyriciproducens	Vocabulary	Acetaminophen	0.921	1.364	0.342	0.79
Meconium Adj.[c]	Agathobaculum butyriciproducens	Vocabulary	Species	0.702	0.635		
Meconium Adj.[c]	Agathobaculum butyriciproducens	Vocabulary	Interaction	0.978	1.066		
Meconium Adj.[c]	Agathobaculum butyriciproducens	WISC sum	Acetaminophen	-0.255	3.155	0.585	0.839
Meconium Adj.[c]	Agathobaculum butyriciproducens	WISC sum	Species	-0.242	1.47		
Meconium Adj.[c]	Agathobaculum butyriciproducens	WISC sum	Interaction	2.67	2.465		
Meconium Adj.[c]	Akkermansia muciniphila	Block Design	Acetaminophen	1.06	1.021	0.626	0.854
Meconium Adj.[c]	Akkermansia muciniphila	Block Design	Species	0.12	0.276		
Meconium Adj.[c]	Akkermansia muciniphila	Block Design	Interaction	0.155	0.964		
Meconium Adj.[c]	Akkermansia muciniphila	Coding	Acetaminophen	0.101	0.894	0.722	0.882
Meconium Adj.[c]	Akkermansia muciniphila	Coding	Species	-0.027	0.242		
Meconium Adj.[c]	Akkermansia muciniphila	Coding	Interaction	0.537	0.844		
Meconium Adj.[c]	Akkermansia muciniphila	Digit span	Acetaminophen	0.065	0.774	0.419	0.836
Meconium Adj.[c]	Akkermansia muciniphila	Digit span	Species	0.027	0.209		
Meconium Adj.[c]	Akkermansia muciniphila	Digit span	Interaction	-0.036	0.73		
Meconium Adj.[c]	Akkermansia muciniphila	Information	Acetaminophen	-0.379	0.754	0.775	0.896
Meconium Adj.[c]	Akkermansia muciniphila	Information	Species	-0.026	0.204		
Meconium Adj.[c]	Akkermansia muciniphila	Information	Interaction	0.99	0.711		
Meconium Adj.[c]	Akkermansia muciniphila	QTAC	Acetaminophen	-1.119	2.458	0.258	0.746
Meconium Adj.[c]	Akkermansia muciniphila	QTAC	Species	-0.192	0.665		
Meconium Adj.[c]	Akkermansia muciniphila	QTAC	Interaction	-3.688	2.319		
Meconium Adj.[c]	Akkermansia muciniphila	Vocabulary	Acetaminophen	0.485	1.145	0.547	0.836
Meconium Adj.[c]	Akkermansia muciniphila	Vocabulary	Species	0.072	0.31		
Meconium Adj.[c]	Akkermansia muciniphila	Vocabulary	Interaction	1.106	1.081		
Meconium Adj.[c]	Akkermansia muciniphila	WISC sum	Acetaminophen	1.332	2.625	0.626	0.854
Meconium Adj.[c]	Akkermansia muciniphila	WISC sum	Species	0.166	0.71		

Exposure Window	Species	Outcome	Variable	Effect Estimate[a]	Std. Error	LRT[b]	
						p-value	q-value
Meconium Adj.[c]	Akkermansia muciniphila	WISC sum	Interaction	2.751	2.476		
Meconium Adj.[c]	Alistipes putredinis	Block Design	Acetaminophen	1.002	1.138	0.945	0.978
Meconium Adj.[c]	Alistipes putredinis	Block Design	Species	-0.089	0.955		
Meconium Adj.[c]	Alistipes putredinis	Block Design	Interaction	0.083	0.984		
Meconium Adj.[c]	Alistipes putredinis	Coding	Acetaminophen	0.45	0.958	0.327	0.787
Meconium Adj.[c]	Alistipes putredinis	Coding	Species	1.198	0.804		
Meconium Adj.[c]	Alistipes putredinis	Coding	Interaction	0.576	0.829		
Meconium Adj.[c]	Alistipes putredinis	Digit span	Acetaminophen	0.305	0.858	0.349	0.79
Meconium Adj.[c]	Alistipes putredinis	Digit span	Species	0.25	0.72		
Meconium Adj.[c]	Alistipes putredinis	Digit span	Interaction	-0.325	0.742		
Meconium Adj.[c]	Alistipes putredinis	Information	Acetaminophen	-0.74	0.795	0.685	0.867
Meconium Adj.[c]	Alistipes putredinis	Information	Species	0.379	0.668		
Meconium Adj.[c]	Alistipes putredinis	Information	Interaction	1.219	0.688		
Meconium Adj.[c]	Alistipes putredinis	QTAC	Acetaminophen	-1.61	2.896	0.372	0.79
Meconium Adj.[c]	Alistipes putredinis	QTAC	Species	2.092	2.431		
Meconium Adj.[c]	Alistipes putredinis	QTAC	Interaction	-4.535	2.505		
Meconium Adj.[c]	Alistipes putredinis	Vocabulary	Acetaminophen	0.479	1.276	0.564	0.836
Meconium Adj.[c]	Alistipes putredinis	Vocabulary	Species	0.642	1.072		
Meconium Adj.[c]	Alistipes putredinis	Vocabulary	Interaction	0.953	1.104		
Meconium Adj.[c]	Alistipes putredinis	WISC sum	Acetaminophen	1.497	2.889	0.439	0.836
Meconium Adj.[c]	Alistipes putredinis	WISC sum	Species	2.381	2.425		
Meconium Adj.[c]	Alistipes putredinis	WISC sum	Interaction	2.506	2.499		
Meconium Adj.[c]	Anaerostipes hadrus	Block Design	Acetaminophen	-0.697	1.811	0.264	0.746
Meconium Adj.[c]	Anaerostipes hadrus	Block Design	Species	-0.01	0.141		
Meconium Adj.[c]	Anaerostipes hadrus	Block Design	Interaction	0.361	0.97		
Meconium Adj.[c]	Anaerostipes hadrus	Coding	Acetaminophen	-0.057	1.58	0.808	0.901
Meconium Adj.[c]	Anaerostipes hadrus	Coding	Species	0.121	0.123		
Meconium Adj.[c]	Anaerostipes hadrus	Coding	Interaction	0.74	0.846		
Meconium Adj.[c]	Anaerostipes hadrus	Digit span	Acetaminophen	0.402	1.407	0.555	0.836
Meconium Adj.[c]	Anaerostipes hadrus	Digit span	Species	0.047	0.109		
Meconium Adj.[c]	Anaerostipes hadrus	Digit span	Interaction	-0.172	0.754		
Meconium Adj.[c]	Anaerostipes hadrus	Information	Acetaminophen	0.623	1.325	0.378	0.79
Meconium Adj.[c]	Anaerostipes hadrus	Information	Species	0.138	0.103		
Meconium Adj.[c]	Anaerostipes hadrus	Information	Interaction	1.011	0.71		
Meconium Adj.[c]	Anaerostipes hadrus	QTAC	Acetaminophen	-7.111	4.621	0.231	0.735
Meconium Adj.[c]	Anaerostipes hadrus	QTAC	Species	0.038	0.359		

Exposure Window	Species	Outcome	Variable	Effect Estimate[a]	Std. Error	LRT[b]	
						p-value	q-value
Meconium Adj.[c]	Anaerostipes hadrus	QTAC	Interaction	-3.53	2.475		
Meconium Adj.[c]	Anaerostipes hadrus	Vocabulary	Acetaminophen	1.53	2.024	0.391	0.802
Meconium Adj.[c]	Anaerostipes hadrus	Vocabulary	Species	0.206	0.157		
Meconium Adj.[c]	Anaerostipes hadrus	Vocabulary	Interaction	1.032	1.084		
Meconium Adj.[c]	Anaerostipes hadrus	WISC sum	Acetaminophen	1.801	4.605	0.773	0.896
Meconium Adj.[c]	Anaerostipes hadrus	WISC sum	Species	0.502	0.358		
Meconium Adj.[c]	Anaerostipes hadrus	WISC sum	Interaction	2.971	2.467		
Meconium Adj.[c]	Asaccharobacter celatus	Block Design	Acetaminophen	1.285	1.316	0.616	0.854
Meconium Adj.[c]	Asaccharobacter celatus	Block Design	Species	-0.258	0.533		
Meconium Adj.[c]	Asaccharobacter celatus	Block Design	Interaction	-0.085	0.997		
Meconium Adj.[c]	Asaccharobacter celatus	Coding	Acetaminophen	0.67	1.158	0.571	0.836
Meconium Adj.[c]	Asaccharobacter celatus	Coding	Species	0.058	0.469		
Meconium Adj.[c]	Asaccharobacter celatus	Coding	Interaction	0.467	0.877		
Meconium Adj.[c]	Asaccharobacter celatus	Digit span	Acetaminophen	0.255	1.011	0.462	0.836
Meconium Adj.[c]	Asaccharobacter celatus	Digit span	Species	0.275	0.409		
Meconium Adj.[c]	Asaccharobacter celatus	Digit span	Interaction	-0.173	0.765		
Meconium Adj.[c]	Asaccharobacter celatus	Information	Acetaminophen	-0.876	0.965	0.327	0.787
Meconium Adj.[c]	Asaccharobacter celatus	Information	Species	0.107	0.391		
Meconium Adj.[c]	Asaccharobacter celatus	Information	Interaction	1.233	0.73		
Meconium Adj.[c]	Asaccharobacter celatus	QTAC	Acetaminophen	-3.622	3.395	0.682	0.867
Meconium Adj.[c]	Asaccharobacter celatus	QTAC	Species	0.133	1.375		
Meconium Adj.[c]	Asaccharobacter celatus	QTAC	Interaction	-3.903	2.57		
Meconium Adj.[c]	Asaccharobacter celatus	Vocabulary	Acetaminophen	0.451	1.489	0.791	0.897
Meconium Adj.[c]	Asaccharobacter celatus	Vocabulary	Species	0.238	0.603		
Meconium Adj.[c]	Asaccharobacter celatus	Vocabulary	Interaction	1.036	1.128		
Meconium Adj.[c]	Asaccharobacter celatus	WISC sum	Acetaminophen	1.784	3.403	0.658	0.856
Meconium Adj.[c]	Asaccharobacter celatus	WISC sum	Species	0.42	1.378		
Meconium Adj.[c]	Asaccharobacter celatus	WISC sum	Interaction	2.478	2.576		
Meconium Adj.[c]	Bacteroides vulgatus	Block Design	Acetaminophen	1.467	1.049	0.185	0.687
Meconium Adj.[c]	Bacteroides vulgatus	Block Design	Species	0.813	0.766		
Meconium Adj.[c]	Bacteroides vulgatus	Block Design	Interaction	0.144	0.964		
Meconium Adj.[c]	Bacteroides vulgatus	Coding	Acetaminophen	0.3	0.911	0.56	0.836
Meconium Adj.[c]	Bacteroides vulgatus	Coding	Species	0.863	0.664		
Meconium Adj.[c]	Bacteroides vulgatus	Coding	Interaction	0.354	0.837		
Meconium Adj.[c]	Bacteroides vulgatus	Digit span	Acetaminophen	-0.156	0.816	0.645	0.854
Meconium Adj.[c]	Bacteroides vulgatus	Digit span	Species	0.432	0.595		

Exposure Window	Species	Outcome	Variable	Effect Estimate[a]	Std. Error	LRT[b]	
						p-value	q-value
Meconium Adj.[c]	Bacteroides vulgatus	Digit span	Interaction	-0.206	0.75		
Meconium Adj.[c]	Bacteroides vulgatus	Information	Acetaminophen	-0.117	0.785	0.57	0.836
Meconium Adj.[c]	Bacteroides vulgatus	Information	Species	0.301	0.573		
Meconium Adj.[c]	Bacteroides vulgatus	Information	Interaction	0.998	0.721		
Meconium Adj.[c]	Bacteroides vulgatus	QTAC	Acetaminophen	-5.562	2.49	0.066	0.652
Meconium Adj.[c]	Bacteroides vulgatus	QTAC	Species	0.355	1.817		
Meconium Adj.[c]	Bacteroides vulgatus	QTAC	Interaction	-5.489	2.288		
Meconium Adj.[c]	Bacteroides vulgatus	Vocabulary	Acetaminophen	0.093	1.201	0.81	0.901
Meconium Adj.[c]	Bacteroides vulgatus	Vocabulary	Species	-0.281	0.876		
Meconium Adj.[c]	Bacteroides vulgatus	Vocabulary	Interaction	1.074	1.104		
Meconium Adj.[c]	Bacteroides vulgatus	WISC sum	Acetaminophen	1.587	2.71	0.364	0.79
Meconium Adj.[c]	Bacteroides vulgatus	WISC sum	Species	2.128	1.977		
Meconium Adj.[c]	Bacteroides vulgatus	WISC sum	Interaction	2.365	2.49		
Meconium Adj.[c]	Bifidobacterium adolescentis	Block Design	Acetaminophen	2.274	1.117	0.03	0.643
Meconium Adj.[c]	Bifidobacterium adolescentis	Block Design	Species	0.074	0.086		
Meconium Adj.[c]	Bifidobacterium adolescentis	Block Design	Interaction	0.517	0.939		
Meconium Adj.[c]	Bifidobacterium adolescentis	Coding	Acetaminophen	-0.643	0.994	0.11	0.652
Meconium Adj.[c]	Bifidobacterium adolescentis	Coding	Species	-0.128	0.077		
Meconium Adj.[c]	Bifidobacterium adolescentis	Coding	Interaction	0.555	0.836		
Meconium Adj.[c]	Bifidobacterium adolescentis	Digit span	Acetaminophen	0.882	0.856	0.021	0.553
Meconium Adj.[c]	Bifidobacterium adolescentis	Digit span	Species	0.079	0.066		
Meconium Adj.[c]	Bifidobacterium adolescentis	Digit span	Interaction	0.138	0.72		
Meconium Adj.[c]	Bifidobacterium adolescentis	Information	Acetaminophen	0.186	0.848	0.319	0.784
Meconium Adj.[c]	Bifidobacterium adolescentis	Information	Species	-0.011	0.065		
Meconium Adj.[c]	Bifidobacterium adolescentis	Information	Interaction	1.247	0.713		
Meconium Adj.[c]	Bifidobacterium adolescentis	QTAC	Acetaminophen	-1.45	2.963	0.431	0.836
Meconium Adj.[c]	Bifidobacterium adolescentis	QTAC	Species	-0.065	0.228		
Meconium Adj.[c]	Bifidobacterium adolescentis	QTAC	Interaction	-3.469	2.491		
Meconium Adj.[c]	Bifidobacterium adolescentis	Vocabulary	Acetaminophen	1.064	1.242	0.205	0.707
Meconium Adj.[c]	Bifidobacterium adolescentis	Vocabulary	Species	-0.071	0.096		
Meconium Adj.[c]	Bifidobacterium adolescentis	Vocabulary	Interaction	1.616	1.044		
Meconium Adj.[c]	Bifidobacterium adolescentis	WISC sum	Acetaminophen	3.764	2.805	0.061	0.652
Meconium Adj.[c]	Bifidobacterium adolescentis	WISC sum	Species	-0.056	0.216		
Meconium Adj.[c]	Bifidobacterium adolescentis	WISC sum	Interaction	4.073	2.359		
Meconium Adj.[c]	Bifidobacterium animalis	Block Design	Acetaminophen	0.614	0.914	0.266	0.746
Meconium Adj.[c]	Bifidobacterium animalis	Block Design	Species	0.199	0.202		

Exposure Window	Species	Outcome	Variable	Effect Estimate[a]	Std. Error	LRT[b]	
						p-value	q-value
Meconium Adj.[c]	Bifidobacterium animalis	Block Design	Interaction	0.19	0.93		
Meconium Adj.[c]	Bifidobacterium animalis	Coding	Acetaminophen	0.085	0.839	0.452	0.836
Meconium Adj.[c]	Bifidobacterium animalis	Coding	Species	0.005	0.186		
Meconium Adj.[c]	Bifidobacterium animalis	Coding	Interaction	0.66	0.854		
Meconium Adj.[c]	Bifidobacterium animalis	Digit span	Acetaminophen	-0.228	0.743	0.935	0.977
Meconium Adj.[c]	Bifidobacterium animalis	Digit span	Species	-0.022	0.164		
Meconium Adj.[c]	Bifidobacterium animalis	Digit span	Interaction	-0.121	0.756		
Meconium Adj.[c]	Bifidobacterium animalis	Information	Acetaminophen	-0.457	0.705	0.342	0.79
Meconium Adj.[c]	Bifidobacterium animalis	Information	Species	-0.033	0.156		
Meconium Adj.[c]	Bifidobacterium animalis	Information	Interaction	1.119	0.718		
Meconium Adj.[c]	Bifidobacterium animalis	QTAC	Acetaminophen	-2.437	2.464	0.583	0.839
Meconium Adj.[c]	Bifidobacterium animalis	QTAC	Species	0.393	0.545		
Meconium Adj.[c]	Bifidobacterium animalis	QTAC	Interaction	-4.575	2.509		
Meconium Adj.[c]	Bifidobacterium animalis	Vocabulary	Acetaminophen	-0.165	1.06	0.217	0.717
Meconium Adj.[c]	Bifidobacterium animalis	Vocabulary	Species	0.025	0.234		
Meconium Adj.[c]	Bifidobacterium animalis	Vocabulary	Interaction	1.203	1.079		
Meconium Adj.[c]	Bifidobacterium animalis	WISC sum	Acetaminophen	-0.152	2.376	0.136	0.652
Meconium Adj.[c]	Bifidobacterium animalis	WISC sum	Species	0.174	0.526		
Meconium Adj.[c]	Bifidobacterium animalis	WISC sum	Interaction	3.05	2.42		
Meconium Adj.[c]	Bifidobacterium bifidum	Block Design	Acetaminophen	1.062	1.029	0.684	0.867
Meconium Adj.[c]	Bifidobacterium bifidum	Block Design	Species	0.063	0.174		
Meconium Adj.[c]	Bifidobacterium bifidum	Block Design	Interaction	0.177	0.969		
Meconium Adj.[c]	Bifidobacterium bifidum	Coding	Acetaminophen	0.004	0.868	0.62	0.854
Meconium Adj.[c]	Bifidobacterium bifidum	Coding	Species	-0.201	0.147		
Meconium Adj.[c]	Bifidobacterium bifidum	Coding	Interaction	0.584	0.818		
Meconium Adj.[c]	Bifidobacterium bifidum	Digit span	Acetaminophen	-0.083	0.778	0.501	0.836
Meconium Adj.[c]	Bifidobacterium bifidum	Digit span	Species	-0.013	0.132		
Meconium Adj.[c]	Bifidobacterium bifidum	Digit span	Interaction	-0.029	0.733		
Meconium Adj.[c]	Bifidobacterium bifidum	Information	Acetaminophen	-0.187	0.718	0.526	0.836
Meconium Adj.[c]	Bifidobacterium bifidum	Information	Species	-0.09	0.122		
Meconium Adj.[c]	Bifidobacterium bifidum	Information	Interaction	1.124	0.676		
Meconium Adj.[c]	Bifidobacterium bifidum	QTAC	Acetaminophen	-3.327	2.629	0.54	0.836
Meconium Adj.[c]	Bifidobacterium bifidum	QTAC	Species	-0.098	0.445		
Meconium Adj.[c]	Bifidobacterium bifidum	QTAC	Interaction	-4.452	2.476		
Meconium Adj.[c]	Bifidobacterium bifidum	Vocabulary	Acetaminophen	0.268	1.14	0.72	0.882
Meconium Adj.[c]	Bifidobacterium bifidum	Vocabulary	Species	-0.076	0.193		

Exposure Window	Species	Outcome	Variable	Effect Estimate[a]	Std. Error	LRT[b]	
						p-value	q-value
Meconium Adj.[c]	Bifidobacterium bifidum	Vocabulary	Interaction	1.13	1.073		
Meconium Adj.[c]	Bifidobacterium bifidum	WISC sum	Acetaminophen	1.063	2.52	0.585	0.839
Meconium Adj.[c]	Bifidobacterium bifidum	WISC sum	Species	-0.316	0.427		
Meconium Adj.[c]	Bifidobacterium bifidum	WISC sum	Interaction	2.987	2.374		
Meconium Adj.[c]	Bifidobacterium longum	Block Design	Acetaminophen	-0.932	1.299	0.032	0.643
Meconium Adj.[c]	Bifidobacterium longum	Block Design	Species	-0.038	0.059		
Meconium Adj.[c]	Bifidobacterium longum	Block Design	Interaction	0.298	0.966		
Meconium Adj.[c]	Bifidobacterium longum	Coding	Acetaminophen	-0.2	1.186	0.53	0.836
Meconium Adj.[c]	Bifidobacterium longum	Coding	Species	0.001	0.054		
Meconium Adj.[c]	Bifidobacterium longum	Coding	Interaction	0.682	0.882		
Meconium Adj.[c]	Bifidobacterium longum	Digit span	Acetaminophen	-0.85	1.031	0.455	0.836
Meconium Adj.[c]	Bifidobacterium longum	Digit span	Species	-0.051	0.047		
Meconium Adj.[c]	Bifidobacterium longum	Digit span	Interaction	-0.308	0.766		
Meconium Adj.[c]	Bifidobacterium longum	Information	Acetaminophen	-1.408	0.968	0.069	0.652
Meconium Adj.[c]	Bifidobacterium longum	Information	Species	-0.014	0.044		
Meconium Adj.[c]	Bifidobacterium longum	Information	Interaction	1.172	0.72		
Meconium Adj.[c]	Bifidobacterium longum	QTAC	Acetaminophen	-0.851	3.413	0.52	0.836
Meconium Adj.[c]	Bifidobacterium longum	QTAC	Species	0.215	0.156		
Meconium Adj.[c]	Bifidobacterium longum	QTAC	Interaction	-3.309	2.538		
Meconium Adj.[c]	Bifidobacterium longum	Vocabulary	Acetaminophen	-1.173	1.471	0.115	0.652
Meconium Adj.[c]	Bifidobacterium longum	Vocabulary	Species	0.028	0.067		
Meconium Adj.[c]	Bifidobacterium longum	Vocabulary	Interaction	1.501	1.094		
Meconium Adj.[c]	Bifidobacterium longum	WISC sum	Acetaminophen	-4.562	3.272	0.012	0.546
Meconium Adj.[c]	Bifidobacterium longum	WISC sum	Species	-0.074	0.149		
Meconium Adj.[c]	Bifidobacterium longum	WISC sum	Interaction	3.346	2.433		
Meconium Adj.[c]	Bifidobacterium pseudocatenulatum	Block Design	Acetaminophen	0.465	1.243	0.615	0.854
Meconium Adj.[c]	Bifidobacterium pseudocatenulatum	Block Design	Species	-0.065	0.119		
Meconium Adj.[c]	Bifidobacterium pseudocatenulatum	Block Design	Interaction	0.296	0.993		
Meconium Adj.[c]	Bifidobacterium pseudocatenulatum	Coding	Acetaminophen	1.256	1.063	0.152	0.657
Meconium Adj.[c]	Bifidobacterium pseudocatenulatum	Coding	Species	0.122	0.101		
Meconium Adj.[c]	Bifidobacterium pseudocatenulatum	Coding	Interaction	0.238	0.849		
Meconium Adj.[c]	Bifidobacterium pseudocatenulatum	Digit span	Acetaminophen	-1.126	0.936	0.143	0.652
Meconium Adj.[c]	Bifidobacterium pseudocatenulatum	Digit span	Species	-0.087	0.089		
Meconium Adj.[c]	Bifidobacterium pseudocatenulatum	Digit span	Interaction	0.142	0.747		
Meconium Adj.[c]	Bifidobacterium pseudocatenulatum	Information	Acetaminophen	-0.521	0.918	0.722	0.882
Meconium Adj.[c]	Bifidobacterium pseudocatenulatum	Information	Species	-0.043	0.087		

Exposure Window	Species	Outcome	Variable	Effect Estimate[a]	Std. Error	LRT[b]	
						p-value	q-value
Meconium Adj.[c]	Bifidobacterium pseudocatenulatum	Information	Interaction	1.112	0.733		
Meconium Adj.[c]	Bifidobacterium pseudocatenulatum	QTAC	Acetaminophen	-1.707	3.073	0.811	0.901
Meconium Adj.[c]	Bifidobacterium pseudocatenulatum	QTAC	Species	0.532	0.293		
Meconium Adj.[c]	Bifidobacterium pseudocatenulatum	QTAC	Interaction	-5.197	2.453		
Meconium Adj.[c]	Bifidobacterium pseudocatenulatum	Vocabulary	Acetaminophen	-1.243	1.36	0.075	0.652
Meconium Adj.[c]	Bifidobacterium pseudocatenulatum	Vocabulary	Species	-0.036	0.13		
Meconium Adj.[c]	Bifidobacterium pseudocatenulatum	Vocabulary	Interaction	1.294	1.085		
Meconium Adj.[c]	Bifidobacterium pseudocatenulatum	WISC sum	Acetaminophen	-1.168	3.179	0.308	0.783
Meconium Adj.[c]	Bifidobacterium pseudocatenulatum	WISC sum	Species	-0.109	0.303		
Meconium Adj.[c]	Bifidobacterium pseudocatenulatum	WISC sum	Interaction	3.081	2.538		
Meconium Adj.[c]	Blautia obeum	Block Design	Acetaminophen	-0.219	1.236	0.163	0.678
Meconium Adj.[c]	Blautia obeum	Block Design	Species	-0.101	0.154		
Meconium Adj.[c]	Blautia obeum	Block Design	Interaction	-0.039	0.952		
Meconium Adj.[c]	Blautia obeum	Coding	Acetaminophen	1.42	1.054	0.169	0.678
Meconium Adj.[c]	Blautia obeum	Coding	Species	0.237	0.131		
Meconium Adj.[c]	Blautia obeum	Coding	Interaction	0.685	0.812		
Meconium Adj.[c]	Blautia obeum	Digit span	Acetaminophen	-0.335	0.922	0.429	0.836
Meconium Adj.[c]	Blautia obeum	Digit span	Species	0.199	0.115		
Meconium Adj.[c]	Blautia obeum	Digit span	Interaction	-0.248	0.711		
Meconium Adj.[c]	Blautia obeum	Information	Acetaminophen	0.005	0.922	0.787	0.896
Meconium Adj.[c]	Blautia obeum	Information	Species	0.099	0.115		
Meconium Adj.[c]	Blautia obeum	Information	Interaction	1.017	0.711		
Meconium Adj.[c]	Blautia obeum	QTAC	Acetaminophen	-5.736	3.145	0.208	0.707
Meconium Adj.[c]	Blautia obeum	QTAC	Species	-0.508	0.391		
Meconium Adj.[c]	Blautia obeum	QTAC	Interaction	-4.552	2.422		
Meconium Adj.[c]	Blautia obeum	Vocabulary	Acetaminophen	-0.904	1.38	0.126	0.652
Meconium Adj.[c]	Blautia obeum	Vocabulary	Species	0.057	0.172		
Meconium Adj.[c]	Blautia obeum	Vocabulary	Interaction	0.78	1.063		
Meconium Adj.[c]	Blautia obeum	WISC sum	Acetaminophen	-0.033	3.136	0.357	0.79
Meconium Adj.[c]	Blautia obeum	WISC sum	Species	0.492	0.39		
Meconium Adj.[c]	Blautia obeum	WISC sum	Interaction	2.194	2.416		
Meconium Adj.[c]	Blautia wexlerae	Block Design	Acetaminophen	1.449	1.155	0.378	0.79
Meconium Adj.[c]	Blautia wexlerae	Block Design	Species	0.194	0.312		
Meconium Adj.[c]	Blautia wexlerae	Block Design	Interaction	0.028	0.967		
Meconium Adj.[c]	Blautia wexlerae	Coding	Acetaminophen	0.874	0.985	0.239	0.738
Meconium Adj.[c]	Blautia wexlerae	Coding	Species	0.002	0.266		

Exposure Window	Species	Outcome	Variable	Effect Estimate[a]	Std. Error	LRT[b]	
						p-value	q-value
Meconium Adj.[c]	Blautia wexlerae	Coding	Interaction	0.562	0.825		
Meconium Adj.[c]	Blautia wexlerae	Digit span	Acetaminophen	-1.071	0.866	0.085	0.652
Meconium Adj.[c]	Blautia wexlerae	Digit span	Species	-0.351	0.234		
Meconium Adj.[c]	Blautia wexlerae	Digit span	Interaction	0.064	0.725		
Meconium Adj.[c]	Blautia wexlerae	Information	Acetaminophen	0.026	0.806	0.496	0.836
Meconium Adj.[c]	Blautia wexlerae	Information	Species	0.41	0.217		
Meconium Adj.[c]	Blautia wexlerae	Information	Interaction	0.806	0.675		
Meconium Adj.[c]	Blautia wexlerae	QTAC	Acetaminophen	-4.157	2.957	0.389	0.802
Meconium Adj.[c]	Blautia wexlerae	QTAC	Species	-0.701	0.798		
Meconium Adj.[c]	Blautia wexlerae	QTAC	Interaction	-3.853	2.476		
Meconium Adj.[c]	Blautia wexlerae	Vocabulary	Acetaminophen	1.227	1.24	0.129	0.652
Meconium Adj.[c]	Blautia wexlerae	Vocabulary	Species	0.709	0.334		
Meconium Adj.[c]	Blautia wexlerae	Vocabulary	Interaction	0.667	1.038		
Meconium Adj.[c]	Blautia wexlerae	WISC sum	Acetaminophen	2.505	2.94	0.29	0.783
Meconium Adj.[c]	Blautia wexlerae	WISC sum	Species	0.964	0.793		
Meconium Adj.[c]	Blautia wexlerae	WISC sum	Interaction	2.126	2.461		
Meconium Adj.[c]	Collinsella aerofaciens	Block Design	Acetaminophen	0.628	1.344	0.762	0.896
Meconium Adj.[c]	Collinsella aerofaciens	Block Design	Species	-0.033	0.096		
Meconium Adj.[c]	Collinsella aerofaciens	Block Design	Interaction	0.124	0.973		
Meconium Adj.[c]	Collinsella aerofaciens	Coding	Acetaminophen	-0.428	1.167	0.375	0.79
Meconium Adj.[c]	Collinsella aerofaciens	Coding	Species	-0.072	0.083		
Meconium Adj.[c]	Collinsella aerofaciens	Coding	Interaction	0.532	0.845		
Meconium Adj.[c]	Collinsella aerofaciens	Digit span	Acetaminophen	-1.317	1.003	0.106	0.652
Meconium Adj.[c]	Collinsella aerofaciens	Digit span	Species	-0.106	0.072		
Meconium Adj.[c]	Collinsella aerofaciens	Digit span	Interaction	-0.202	0.726		
Meconium Adj.[c]	Collinsella aerofaciens	Information	Acetaminophen	-0.399	0.992	0.862	0.92
Meconium Adj.[c]	Collinsella aerofaciens	Information	Species	-0.008	0.071		
Meconium Adj.[c]	Collinsella aerofaciens	Information	Interaction	0.998	0.718		
Meconium Adj.[c]	Collinsella aerofaciens	QTAC	Acetaminophen	-1.915	3.447	0.708	0.882
Meconium Adj.[c]	Collinsella aerofaciens	QTAC	Species	0.077	0.246		
Meconium Adj.[c]	Collinsella aerofaciens	QTAC	Interaction	-4.146	2.496		
Meconium Adj.[c]	Collinsella aerofaciens	Vocabulary	Acetaminophen	-0.279	1.511	0.644	0.854
Meconium Adj.[c]	Collinsella aerofaciens	Vocabulary	Species	-0.043	0.108		
Meconium Adj.[c]	Collinsella aerofaciens	Vocabulary	Interaction	0.993	1.094		
Meconium Adj.[c]	Collinsella aerofaciens	WISC sum	Acetaminophen	-1.795	3.406	0.248	0.746
Meconium Adj.[c]	Collinsella aerofaciens	WISC sum	Species	-0.262	0.243		

Exposure Window	Species	Outcome	Variable	Effect Estimate[a]	Std. Error	LRT[b]	
						p-value	q-value
Meconium Adj.[c]	Collinsella aerofaciens	WISC sum	Interaction	2.446	2.466		
Meconium Adj.[c]	Coprococcus catus	Block Design	Acetaminophen	0.897	1.45	0.995	0.999
Meconium Adj.[c]	Coprococcus catus	Block Design	Species	-0.413	0.868		
Meconium Adj.[c]	Coprococcus catus	Block Design	Interaction	0.079	0.964		
Meconium Adj.[c]	Coprococcus catus	Coding	Acetaminophen	1.518	1.246	0.147	0.652
Meconium Adj.[c]	Coprococcus catus	Coding	Species	0.626	0.746		
Meconium Adj.[c]	Coprococcus catus	Coding	Interaction	0.555	0.828		
Meconium Adj.[c]	Coprococcus catus	Digit span	Acetaminophen	-0.598	1.112	0.65	0.854
Meconium Adj.[c]	Coprococcus catus	Digit span	Species	-0.447	0.666		
Meconium Adj.[c]	Coprococcus catus	Digit span	Interaction	-0.152	0.739		
Meconium Adj.[c]	Coprococcus catus	Information	Acetaminophen	-2.082	1.007	0.013	0.546
Meconium Adj.[c]	Coprococcus catus	Information	Species	-0.623	0.603		
Meconium Adj.[c]	Coprococcus catus	Information	Interaction	1.073	0.669		
Meconium Adj.[c]	Coprococcus catus	QTAC	Acetaminophen	1.528	3.426	0.078	0.652
Meconium Adj.[c]	Coprococcus catus	QTAC	Species	5.529	2.051		
Meconium Adj.[c]	Coprococcus catus	QTAC	Interaction	-3.847	2.276		
Meconium Adj.[c]	Coprococcus catus	Vocabulary	Acetaminophen	-1.001	1.619	0.298	0.783
Meconium Adj.[c]	Coprococcus catus	Vocabulary	Species	-0.244	0.969		
Meconium Adj.[c]	Coprococcus catus	Vocabulary	Interaction	1.092	1.076		
Meconium Adj.[c]	Coprococcus catus	WISC sum	Acetaminophen	-1.266	3.717	0.429	0.836
Meconium Adj.[c]	Coprococcus catus	WISC sum	Species	-1.101	2.225		
Meconium Adj.[c]	Coprococcus catus	WISC sum	Interaction	2.647	2.47		
Meconium Adj.[c]	Coprococcus comes	Block Design	Acetaminophen	0.28	1.349	0.487	0.836
Meconium Adj.[c]	Coprococcus comes	Block Design	Species	-0.178	0.408		
Meconium Adj.[c]	Coprococcus comes	Block Design	Interaction	0.153	0.979		
Meconium Adj.[c]	Coprococcus comes	Coding	Acetaminophen	1.305	1.143	0.144	0.652
Meconium Adj.[c]	Coprococcus comes	Coding	Species	0.598	0.346		
Meconium Adj.[c]	Coprococcus comes	Coding	Interaction	0.711	0.829		
Meconium Adj.[c]	Coprococcus comes	Digit span	Acetaminophen	-0.78	1.029	0.456	0.836
Meconium Adj.[c]	Coprococcus comes	Digit span	Species	0.05	0.311		
Meconium Adj.[c]	Coprococcus comes	Digit span	Interaction	0.016	0.747		
Meconium Adj.[c]	Coprococcus comes	Information	Acetaminophen	-1.114	0.983	0.205	0.707
Meconium Adj.[c]	Coprococcus comes	Information	Species	-0.185	0.297		
Meconium Adj.[c]	Coprococcus comes	Information	Interaction	1.071	0.713		
Meconium Adj.[c]	Coprococcus comes	QTAC	Acetaminophen	-3.61	3.366	0.773	0.896
Meconium Adj.[c]	Coprococcus comes	QTAC	Species	1.147	1.018		

Exposure Window	Species	Outcome	Variable	Effect Estimate[a]	Std. Error	LRT[b]	
						p-value	q-value
Meconium Adj.[c]	Coprococcus comes	QTAC	Interaction	-3.373	2.442		
Meconium Adj.[c]	Coprococcus comes	Vocabulary	Acetaminophen	-0.758	1.49	0.377	0.79
Meconium Adj.[c]	Coprococcus comes	Vocabulary	Species	0.182	0.451		
Meconium Adj.[c]	Coprococcus comes	Vocabulary	Interaction	1.327	1.081		
Meconium Adj.[c]	Coprococcus comes	WISC sum	Acetaminophen	-1.068	3.415	0.448	0.836
Meconium Adj.[c]	Coprococcus comes	WISC sum	Species	0.467	1.033		
Meconium Adj.[c]	Coprococcus comes	WISC sum	Interaction	3.278	2.478		
Meconium Adj.[c]	Coprococcus eutactus	Block Design	Acetaminophen	0.801	1.03	0.567	0.836
Meconium Adj.[c]	Coprococcus eutactus	Block Design	Species	-0.433	0.49		
Meconium Adj.[c]	Coprococcus eutactus	Block Design	Interaction	0.489	1.02		
Meconium Adj.[c]	Coprococcus eutactus	Coding	Acetaminophen	0.211	0.896	0.573	0.836
Meconium Adj.[c]	Coprococcus eutactus	Coding	Species	-0.44	0.426		
Meconium Adj.[c]	Coprococcus eutactus	Coding	Interaction	0.965	0.887		
Meconium Adj.[c]	Coprococcus eutactus	Digit span	Acetaminophen	-0.54	0.794	0.444	0.836
Meconium Adj.[c]	Coprococcus eutactus	Digit span	Species	-0.117	0.377		
Meconium Adj.[c]	Coprococcus eutactus	Digit span	Interaction	-0.16	0.786		
Meconium Adj.[c]	Coprococcus eutactus	Information	Acetaminophen	-0.045	0.759	0.643	0.854
Meconium Adj.[c]	Coprococcus eutactus	Information	Species	-0.05	0.361		
Meconium Adj.[c]	Coprococcus eutactus	Information	Interaction	1.177	0.751		
Meconium Adj.[c]	Coprococcus eutactus	QTAC	Acetaminophen	-1.498	2.624	0.183	0.687
Meconium Adj.[c]	Coprococcus eutactus	QTAC	Species	1.418	1.247		
Meconium Adj.[c]	Coprococcus eutactus	QTAC	Interaction	-4.916	2.597		
Meconium Adj.[c]	Coprococcus eutactus	Vocabulary	Acetaminophen	0.679	1.153	0.468	0.836
Meconium Adj.[c]	Coprococcus eutactus	Vocabulary	Species	0.002	0.548		
Meconium Adj.[c]	Coprococcus eutactus	Vocabulary	Interaction	1.277	1.141		
Meconium Adj.[c]	Coprococcus eutactus	WISC sum	Acetaminophen	1.107	2.63	0.845	0.91
Meconium Adj.[c]	Coprococcus eutactus	WISC sum	Species	-1.038	1.25		
Meconium Adj.[c]	Coprococcus eutactus	WISC sum	Interaction	3.749	2.603		
Meconium Adj.[c]	Dorea formicigenerans	Block Design	Acetaminophen	2.045	1.614	0.361	0.79
Meconium Adj.[c]	Dorea formicigenerans	Block Design	Species	1.018	0.896		
Meconium Adj.[c]	Dorea formicigenerans	Block Design	Interaction	0.193	0.947		
Meconium Adj.[c]	Dorea formicigenerans	Coding	Acetaminophen	-0.888	1.405	0.303	0.783
Meconium Adj.[c]	Dorea formicigenerans	Coding	Species	-1.05	0.78		
Meconium Adj.[c]	Dorea formicigenerans	Coding	Interaction	0.512	0.824		
Meconium Adj.[c]	Dorea formicigenerans	Digit span	Acetaminophen	-2.137	1.134	0.038	0.648
Meconium Adj.[c]	Dorea formicigenerans	Digit span	Species	-1.934	0.629		

Exposure Window	Species	Outcome	Variable	Effect Estimate[a]	Std. Error	LRT[b]	
						p-value	q-value
Meconium Adj.[c]	Dorea formicigenerans	Digit span	Interaction	-0.239	0.665		
Meconium Adj.[c]	Dorea formicigenerans	Information	Acetaminophen	-1.207	1.197	0.303	0.783
Meconium Adj.[c]	Dorea formicigenerans	Information	Species	-0.309	0.665		
Meconium Adj.[c]	Dorea formicigenerans	Information	Interaction	0.998	0.702		
Meconium Adj.[c]	Dorea formicigenerans	QTAC	Acetaminophen	-7.667	4.101	0.116	0.652
Meconium Adj.[c]	Dorea formicigenerans	QTAC	Species	-2.37	2.277		
Meconium Adj.[c]	Dorea formicigenerans	QTAC	Interaction	-4.339	2.405		
Meconium Adj.[c]	Dorea formicigenerans	Vocabulary	Acetaminophen	-0.253	1.845	0.747	0.896
Meconium Adj.[c]	Dorea formicigenerans	Vocabulary	Species	-0.014	1.024		
Meconium Adj.[c]	Dorea formicigenerans	Vocabulary	Interaction	1.033	1.082		
Meconium Adj.[c]	Dorea formicigenerans	WISC sum	Acetaminophen	-2.439	4.16	0.314	0.783
Meconium Adj.[c]	Dorea formicigenerans	WISC sum	Species	-2.289	2.31		
Meconium Adj.[c]	Dorea formicigenerans	WISC sum	Interaction	2.497	2.44		
Meconium Adj.[c]	Dorea longicatena	Block Design	Acetaminophen	2.18	1.561	0.254	0.746
Meconium Adj.[c]	Dorea longicatena	Block Design	Species	0.118	0.29		
Meconium Adj.[c]	Dorea longicatena	Block Design	Interaction	0.181	0.952		
Meconium Adj.[c]	Dorea longicatena	Coding	Acetaminophen	0.168	1.379	0.973	0.989
Meconium Adj.[c]	Dorea longicatena	Coding	Species	-0.14	0.256		
Meconium Adj.[c]	Dorea longicatena	Coding	Interaction	0.629	0.841		
Meconium Adj.[c]	Dorea longicatena	Digit span	Acetaminophen	-1.268	1.186	0.27	0.748
Meconium Adj.[c]	Dorea longicatena	Digit span	Species	-0.322	0.22		
Meconium Adj.[c]	Dorea longicatena	Digit span	Interaction	-0.061	0.723		
Meconium Adj.[c]	Dorea longicatena	Information	Acetaminophen	-1.55	1.121	0.11	0.652
Meconium Adj.[c]	Dorea longicatena	Information	Species	-0.009	0.208		
Meconium Adj.[c]	Dorea longicatena	Information	Interaction	0.917	0.684		
Meconium Adj.[c]	Dorea longicatena	QTAC	Acetaminophen	-7.747	3.935	0.093	0.652
Meconium Adj.[c]	Dorea longicatena	QTAC	Species	-0.991	0.731		
Meconium Adj.[c]	Dorea longicatena	QTAC	Interaction	-4.177	2.4		
Meconium Adj.[c]	Dorea longicatena	Vocabulary	Acetaminophen	-0.013	1.712	0.785	0.896
Meconium Adj.[c]	Dorea longicatena	Vocabulary	Species	0.401	0.318		
Meconium Adj.[c]	Dorea longicatena	Vocabulary	Interaction	0.841	1.044		
Meconium Adj.[c]	Dorea longicatena	WISC sum	Acetaminophen	-0.483	4.056	0.648	0.854
Meconium Adj.[c]	Dorea longicatena	WISC sum	Species	0.048	0.753		
Meconium Adj.[c]	Dorea longicatena	WISC sum	Interaction	2.507	2.473		
Meconium Adj.[c]	Eubacterium hallii	Block Design	Acetaminophen	1.907	1.889	0.514	0.836
Meconium Adj.[c]	Eubacterium hallii	Block Design	Species	0.338	0.41		

Exposure Window	Species	Outcome	Variable	Effect Estimate[a]	Std. Error	LRT[b]	
						p-value	q-value
Meconium Adj.[c]	Eubacterium hallii	Block Design	Interaction	0.173	0.991		
Meconium Adj.[c]	Eubacterium hallii	Coding	Acetaminophen	-0.548	1.64	0.571	0.836
Meconium Adj.[c]	Eubacterium hallii	Coding	Species	-0.413	0.356		
Meconium Adj.[c]	Eubacterium hallii	Coding	Interaction	0.439	0.861		
Meconium Adj.[c]	Eubacterium hallii	Digit span	Acetaminophen	1.466	1.411	0.141	0.652
Meconium Adj.[c]	Eubacterium hallii	Digit span	Species	0.522	0.306		
Meconium Adj.[c]	Eubacterium hallii	Digit span	Interaction	-0.089	0.74		
Meconium Adj.[c]	Eubacterium hallii	Information	Acetaminophen	0.165	1.383	0.715	0.882
Meconium Adj.[c]	Eubacterium hallii	Information	Species	0.32	0.3		
Meconium Adj.[c]	Eubacterium hallii	Information	Interaction	1.149	0.726		
Meconium Adj.[c]	Eubacterium hallii	QTAC	Acetaminophen	-5.508	4.853	0.484	0.836
Meconium Adj.[c]	Eubacterium hallii	QTAC	Species	-0.715	1.054		
Meconium Adj.[c]	Eubacterium hallii	QTAC	Interaction	-4.186	2.547		
Meconium Adj.[c]	Eubacterium hallii	Vocabulary	Acetaminophen	-0.162	2.084	0.783	0.896
Meconium Adj.[c]	Eubacterium hallii	Vocabulary	Species	0.497	0.453		
Meconium Adj.[c]	Eubacterium hallii	Vocabulary	Interaction	1.447	1.094		
Meconium Adj.[c]	Eubacterium hallii	WISC sum	Acetaminophen	2.829	4.806	0.628	0.854
Meconium Adj.[c]	Eubacterium hallii	WISC sum	Species	1.263	1.044		
Meconium Adj.[c]	Eubacterium hallii	WISC sum	Interaction	3.118	2.523		
Meconium Adj.[c]	Eubacterium rectale	Block Design	Acetaminophen	1.119	1.132	0.52	0.836
Meconium Adj.[c]	Eubacterium rectale	Block Design	Species	-0.03	0.089		
Meconium Adj.[c]	Eubacterium rectale	Block Design	Interaction	0.007	0.988		
Meconium Adj.[c]	Eubacterium rectale	Coding	Acetaminophen	0.269	0.998	0.961	0.985
Meconium Adj.[c]	Eubacterium rectale	Coding	Species	0	0.079		
Meconium Adj.[c]	Eubacterium rectale	Coding	Interaction	0.571	0.872		
Meconium Adj.[c]	Eubacterium rectale	Digit span	Acetaminophen	-0.29	0.873	0.761	0.896
Meconium Adj.[c]	Eubacterium rectale	Digit span	Species	0.024	0.069		
Meconium Adj.[c]	Eubacterium rectale	Digit span	Interaction	-0.037	0.763		
Meconium Adj.[c]	Eubacterium rectale	Information	Acetaminophen	0.501	0.787	0.017	0.546
Meconium Adj.[c]	Eubacterium rectale	Information	Species	-0.015	0.062		
Meconium Adj.[c]	Eubacterium rectale	Information	Interaction	0.872	0.687		
Meconium Adj.[c]	Eubacterium rectale	QTAC	Acetaminophen	-5.198	2.826	0.234	0.735
Meconium Adj.[c]	Eubacterium rectale	QTAC	Species	-0.337	0.223		
Meconium Adj.[c]	Eubacterium rectale	QTAC	Interaction	-5.06	2.468		
Meconium Adj.[c]	Eubacterium rectale	Vocabulary	Acetaminophen	0.197	1.246	0.493	0.836
Meconium Adj.[c]	Eubacterium rectale	Vocabulary	Species	-0.119	0.098		

Exposure Window	Species	Outcome	Variable	Effect Estimate[a]	Std. Error	LRT[b]	
						p-value	q-value
Meconium Adj.[c]	Eubacterium rectale	Vocabulary	Interaction	0.633	1.088		
Meconium Adj.[c]	Eubacterium rectale	WISC sum	Acetaminophen	1.797	2.864	0.253	0.746
Meconium Adj.[c]	Eubacterium rectale	WISC sum	Species	-0.141	0.226		
Meconium Adj.[c]	Eubacterium rectale	WISC sum	Interaction	2.046	2.501		
Meconium Adj.[c]	Eubacterium siraeum	Block Design	Acetaminophen	0.925	1.001	0.549	0.836
Meconium Adj.[c]	Eubacterium siraeum	Block Design	Species	-0.529	0.331		
Meconium Adj.[c]	Eubacterium siraeum	Block Design	Interaction	0.236	0.972		
Meconium Adj.[c]	Eubacterium siraeum	Coding	Acetaminophen	0.344	0.916	0.819	0.905
Meconium Adj.[c]	Eubacterium siraeum	Coding	Species	0.049	0.302		
Meconium Adj.[c]	Eubacterium siraeum	Coding	Interaction	0.508	0.889		
Meconium Adj.[c]	Eubacterium siraeum	Digit span	Acetaminophen	-0.039	0.801	0.51	0.836
Meconium Adj.[c]	Eubacterium siraeum	Digit span	Species	0.031	0.264		
Meconium Adj.[c]	Eubacterium siraeum	Digit span	Interaction	-0.25	0.777		
Meconium Adj.[c]	Eubacterium siraeum	Information	Acetaminophen	-0.577	0.764	0.35	0.79
Meconium Adj.[c]	Eubacterium siraeum	Information	Species	-0.069	0.252		
Meconium Adj.[c]	Eubacterium siraeum	Information	Interaction	1.197	0.742		
Meconium Adj.[c]	Eubacterium siraeum	QTAC	Acetaminophen	-2.715	2.674	0.907	0.959
Meconium Adj.[c]	Eubacterium siraeum	QTAC	Species	0.431	0.883		
Meconium Adj.[c]	Eubacterium siraeum	QTAC	Interaction	-4.343	2.595		
Meconium Adj.[c]	Eubacterium siraeum	Vocabulary	Acetaminophen	0.679	1.145	0.485	0.836
Meconium Adj.[c]	Eubacterium siraeum	Vocabulary	Species	0.57	0.378		
Meconium Adj.[c]	Eubacterium siraeum	Vocabulary	Interaction	0.606	1.111		
Meconium Adj.[c]	Eubacterium siraeum	WISC sum	Acetaminophen	1.332	2.683	0.596	0.85
Meconium Adj.[c]	Eubacterium siraeum	WISC sum	Species	0.051	0.886		
Meconium Adj.[c]	Eubacterium siraeum	WISC sum	Interaction	2.297	2.604		
Meconium Adj.[c]	Eubacterium sp CAG 180	Block Design	Acetaminophen	0.215	0.944	0.042	0.648
Meconium Adj.[c]	Eubacterium sp CAG 180	Block Design	Species	-0.905	0.378		
Meconium Adj.[c]	Eubacterium sp CAG 180	Block Design	Interaction	0.206	0.899		
Meconium Adj.[c]	Eubacterium sp CAG 180	Coding	Acetaminophen	-0.171	0.865	0.17	0.678
Meconium Adj.[c]	Eubacterium sp CAG 180	Coding	Species	-0.444	0.346		
Meconium Adj.[c]	Eubacterium sp CAG 180	Coding	Interaction	0.594	0.824		
Meconium Adj.[c]	Eubacterium sp CAG 180	Digit span	Acetaminophen	-0.944	0.709	0.009	0.546
Meconium Adj.[c]	Eubacterium sp CAG 180	Digit span	Species	-0.083	0.284		
Meconium Adj.[c]	Eubacterium sp CAG 180	Digit span	Interaction	-0.234	0.675		
Meconium Adj.[c]	Eubacterium sp CAG 180	Information	Acetaminophen	-0.558	0.675	0.314	0.783
Meconium Adj.[c]	Eubacterium sp CAG 180	Information	Species	0.455	0.27		

Exposure Window	Species	Outcome	Variable	Effect Estimate[a]	Std. Error	LRT[b]	
						p-value	q-value
Meconium Adj.[c]	Eubacterium sp CAG 180	Information	Interaction	0.86	0.643		
Meconium Adj.[c]	Eubacterium sp CAG 180	QTAC	Acetaminophen	-3.999	2.548	0.177	0.679
Meconium Adj.[c]	Eubacterium sp CAG 180	QTAC	Species	-0.715	1.02		
Meconium Adj.[c]	Eubacterium sp CAG 180	QTAC	Interaction	-4.294	2.427		
Meconium Adj.[c]	Eubacterium sp CAG 180	Vocabulary	Acetaminophen	-0.612	1.07	0.049	0.648
Meconium Adj.[c]	Eubacterium sp CAG 180	Vocabulary	Species	0.013	0.428		
Meconium Adj.[c]	Eubacterium sp CAG 180	Vocabulary	Interaction	0.88	1.019		
Meconium Adj.[c]	Eubacterium sp CAG 180	WISC sum	Acetaminophen	-2.07	2.314	0.001	0.292
Meconium Adj.[c]	Eubacterium sp CAG 180	WISC sum	Species	-0.964	0.926		
Meconium Adj.[c]	Eubacterium sp CAG 180	WISC sum	Interaction	2.306	2.204		
Meconium Adj.[c]	Faecalibacterium prausnitzii	Block Design	Acetaminophen	2.26	1.724	0.307	0.783
Meconium Adj.[c]	Faecalibacterium prausnitzii	Block Design	Species	0.04	0.108		
Meconium Adj.[c]	Faecalibacterium prausnitzii	Block Design	Interaction	-0.152	1		
Meconium Adj.[c]	Faecalibacterium prausnitzii	Coding	Acetaminophen	-1.26	1.473	0.204	0.707
Meconium Adj.[c]	Faecalibacterium prausnitzii	Coding	Species	0.029	0.092		
Meconium Adj.[c]	Faecalibacterium prausnitzii	Coding	Interaction	0.867	0.854		
Meconium Adj.[c]	Faecalibacterium prausnitzii	Digit span	Acetaminophen	1.107	1.308	0.171	0.678
Meconium Adj.[c]	Faecalibacterium prausnitzii	Digit span	Species	0.104	0.082		
Meconium Adj.[c]	Faecalibacterium prausnitzii	Digit span	Interaction	-0.422	0.759		
Meconium Adj.[c]	Faecalibacterium prausnitzii	Information	Acetaminophen	1.154	1.238	0.146	0.652
Meconium Adj.[c]	Faecalibacterium prausnitzii	Information	Species	-0.01	0.077		
Meconium Adj.[c]	Faecalibacterium prausnitzii	Information	Interaction	0.727	0.717		
Meconium Adj.[c]	Faecalibacterium prausnitzii	QTAC	Acetaminophen	-8.25	4.332	0.108	0.652
Meconium Adj.[c]	Faecalibacterium prausnitzii	QTAC	Species	-0.096	0.271		
Meconium Adj.[c]	Faecalibacterium prausnitzii	QTAC	Interaction	-3.095	2.512		
Meconium Adj.[c]	Faecalibacterium prausnitzii	Vocabulary	Acetaminophen	2.498	1.873	0.124	0.652
Meconium Adj.[c]	Faecalibacterium prausnitzii	Vocabulary	Species	-0.029	0.117		
Meconium Adj.[c]	Faecalibacterium prausnitzii	Vocabulary	Interaction	0.575	1.086		
Meconium Adj.[c]	Faecalibacterium prausnitzii	WISC sum	Acetaminophen	5.759	4.38	0.147	0.652
Meconium Adj.[c]	Faecalibacterium prausnitzii	WISC sum	Species	0.134	0.274		
Meconium Adj.[c]	Faecalibacterium prausnitzii	WISC sum	Interaction	1.595	2.539		
Meconium Adj.[c]	Fusicatenibacter saccharivorans	Block Design	Acetaminophen	1.232	1.743	0.825	0.905
Meconium Adj.[c]	Fusicatenibacter saccharivorans	Block Design	Species	-0.015	0.156		
Meconium Adj.[c]	Fusicatenibacter saccharivorans	Block Design	Interaction	0.072	0.971		
Meconium Adj.[c]	Fusicatenibacter saccharivorans	Coding	Acetaminophen	1.164	1.513	0.462	0.836
Meconium Adj.[c]	Fusicatenibacter saccharivorans	Coding	Species	0.014	0.136		

Exposure Window	Species	Outcome	Variable	Effect Estimate[a]	Std. Error	LRT[b]	
						p-value	q-value
Meconium Adj.[c]	Fusicatenibacter saccharivorans	Coding	Interaction	0.451	0.843		
Meconium Adj.[c]	Fusicatenibacter saccharivorans	Digit span	Acetaminophen	-0.885	1.33	0.496	0.836
Meconium Adj.[c]	Fusicatenibacter saccharivorans	Digit span	Species	-0.103	0.119		
Meconium Adj.[c]	Fusicatenibacter saccharivorans	Digit span	Interaction	-0.104	0.741		
Meconium Adj.[c]	Fusicatenibacter saccharivorans	Information	Acetaminophen	0.802	1.271	0.262	0.746
Meconium Adj.[c]	Fusicatenibacter saccharivorans	Information	Species	0.095	0.114		
Meconium Adj.[c]	Fusicatenibacter saccharivorans	Information	Interaction	0.922	0.708		
Meconium Adj.[c]	Fusicatenibacter saccharivorans	QTAC	Acetaminophen	-6.913	4.369	0.192	0.691
Meconium Adj.[c]	Fusicatenibacter saccharivorans	QTAC	Species	-0.562	0.392		
Meconium Adj.[c]	Fusicatenibacter saccharivorans	QTAC	Interaction	-4.026	2.434		
Meconium Adj.[c]	Fusicatenibacter saccharivorans	Vocabulary	Acetaminophen	0.439	1.935	0.786	0.896
Meconium Adj.[c]	Fusicatenibacter saccharivorans	Vocabulary	Species	0.154	0.173		
Meconium Adj.[c]	Fusicatenibacter saccharivorans	Vocabulary	Interaction	1.105	1.078		
Meconium Adj.[c]	Fusicatenibacter saccharivorans	WISC sum	Acetaminophen	2.752	4.478	0.569	0.836
Meconium Adj.[c]	Fusicatenibacter saccharivorans	WISC sum	Species	0.146	0.401		
Meconium Adj.[c]	Fusicatenibacter saccharivorans	WISC sum	Interaction	2.446	2.495		
Meconium Adj.[c]	Methanobrevibacter smithii	Block Design	Acetaminophen	1.065	1.017	0.776	0.896
Meconium Adj.[c]	Methanobrevibacter smithii	Block Design	Species	0.22	0.278		
Meconium Adj.[c]	Methanobrevibacter smithii	Block Design	Interaction	-0.047	0.977		
Meconium Adj.[c]	Methanobrevibacter smithii	Coding	Acetaminophen	-0.23	0.872	0.117	0.652
Meconium Adj.[c]	Methanobrevibacter smithii	Coding	Species	-0.084	0.238		
Meconium Adj.[c]	Methanobrevibacter smithii	Coding	Interaction	0.565	0.837		
Meconium Adj.[c]	Methanobrevibacter smithii	Digit span	Acetaminophen	-0.573	0.772	0.212	0.71
Meconium Adj.[c]	Methanobrevibacter smithii	Digit span	Species	-0.035	0.211		
Meconium Adj.[c]	Methanobrevibacter smithii	Digit span	Interaction	-0.151	0.742		
Meconium Adj.[c]	Methanobrevibacter smithii	Information	Acetaminophen	-0.815	0.727	0.051	0.648
Meconium Adj.[c]	Methanobrevibacter smithii	Information	Species	-0.169	0.199		
Meconium Adj.[c]	Methanobrevibacter smithii	Information	Interaction	1.074	0.698		
Meconium Adj.[c]	Methanobrevibacter smithii	QTAC	Acetaminophen	-2.865	2.54	0.648	0.854
Meconium Adj.[c]	Methanobrevibacter smithii	QTAC	Species	0.76	0.694		
Meconium Adj.[c]	Methanobrevibacter smithii	QTAC	Interaction	-4.971	2.438		
Meconium Adj.[c]	Methanobrevibacter smithii	Vocabulary	Acetaminophen	-0.136	1.126	0.335	0.79
Meconium Adj.[c]	Methanobrevibacter smithii	Vocabulary	Species	0.116	0.308		
Meconium Adj.[c]	Methanobrevibacter smithii	Vocabulary	Interaction	0.859	1.081		
Meconium Adj.[c]	Methanobrevibacter smithii	WISC sum	Acetaminophen	-0.69	2.501	0.07	0.652
Meconium Adj.[c]	Methanobrevibacter smithii	WISC sum	Species	0.047	0.684		

Exposure Window	Species	Outcome	Variable	Effect Estimate[a]	Std. Error	LRT[b]	
						p-value	q-value
Meconium Adj.[c]	Methanobrevibacter smithii	WISC sum	Interaction	2.301	2.402		
Meconium Adj.[c]	Prevotella copri	Block Design	Acetaminophen	0.802	1.011	0.797	0.899
Meconium Adj.[c]	Prevotella copri	Block Design	Species	-0.086	0.348		
Meconium Adj.[c]	Prevotella copri	Block Design	Interaction	0.187	0.983		
Meconium Adj.[c]	Prevotella copri	Coding	Acetaminophen	-0.015	0.88	0.405	0.824
Meconium Adj.[c]	Prevotella copri	Coding	Species	-0.112	0.303		
Meconium Adj.[c]	Prevotella copri	Coding	Interaction	0.659	0.855		
Meconium Adj.[c]	Prevotella copri	Digit span	Acetaminophen	-0.185	0.758	0.853	0.914
Meconium Adj.[c]	Prevotella copri	Digit span	Species	-0.286	0.261		
Meconium Adj.[c]	Prevotella copri	Digit span	Interaction	0.028	0.737		
Meconium Adj.[c]	Prevotella copri	Information	Acetaminophen	-0.892	0.702	0.018	0.546
Meconium Adj.[c]	Prevotella copri	Information	Species	-0.433	0.242		
Meconium Adj.[c]	Prevotella copri	Information	Interaction	1.3	0.682		
Meconium Adj.[c]	Prevotella copri	QTAC	Acetaminophen	-1.54	2.515	0.172	0.678
Meconium Adj.[c]	Prevotella copri	QTAC	Species	1.36	0.866		
Meconium Adj.[c]	Prevotella copri	QTAC	Interaction	-5.066	2.444		
Meconium Adj.[c]	Prevotella copri	Vocabulary	Acetaminophen	0.27	1.133	0.831	0.907
Meconium Adj.[c]	Prevotella copri	Vocabulary	Species	-0.154	0.39		
Meconium Adj.[c]	Prevotella copri	Vocabulary	Interaction	1.106	1.101		
Meconium Adj.[c]	Prevotella copri	WISC sum	Acetaminophen	-0.02	2.556	0.367	0.79
Meconium Adj.[c]	Prevotella copri	WISC sum	Species	-1.071	0.88		
Meconium Adj.[c]	Prevotella copri	WISC sum	Interaction	3.279	2.484		
Meconium Adj.[c]	Roseburia faecis	Block Design	Acetaminophen	0.518	1.069	0.453	0.836
Meconium Adj.[c]	Roseburia faecis	Block Design	Species	-0.203	0.257		
Meconium Adj.[c]	Roseburia faecis	Block Design	Interaction	0.066	0.972		
Meconium Adj.[c]	Roseburia faecis	Coding	Acetaminophen	0.198	0.913	0.958	0.985
Meconium Adj.[c]	Roseburia faecis	Coding	Species	-0.187	0.219		
Meconium Adj.[c]	Roseburia faecis	Coding	Interaction	0.708	0.83		
Meconium Adj.[c]	Roseburia faecis	Digit span	Acetaminophen	-0.385	0.818	0.68	0.867
Meconium Adj.[c]	Roseburia faecis	Digit span	Species	0.027	0.196		
Meconium Adj.[c]	Roseburia faecis	Digit span	Interaction	-0.239	0.743		
Meconium Adj.[c]	Roseburia faecis	Information	Acetaminophen	-0.102	0.775	0.565	0.836
Meconium Adj.[c]	Roseburia faecis	Information	Species	-0.037	0.186		
Meconium Adj.[c]	Roseburia faecis	Information	Interaction	1.166	0.704		
Meconium Adj.[c]	Roseburia faecis	QTAC	Acetaminophen	-1.997	2.713	0.512	0.836
Meconium Adj.[c]	Roseburia faecis	QTAC	Species	-0.009	0.651		

Exposure Window	Species	Outcome	Variable	Effect Estimate[a]	Std. Error	LRT[b]	
						p-value	q-value
Meconium Adj.[c]	Roseburia faecis	QTAC	Interaction	-3.711	2.466		
Meconium Adj.[c]	Roseburia faecis	Vocabulary	Acetaminophen	-0.878	1.15	0.054	0.648
Meconium Adj.[c]	Roseburia faecis	Vocabulary	Species	-0.581	0.276		
Meconium Adj.[c]	Roseburia faecis	Vocabulary	Interaction	0.854	1.045		
Meconium Adj.[c]	Roseburia faecis	WISC sum	Acetaminophen	-0.65	2.681	0.266	0.746
Meconium Adj.[c]	Roseburia faecis	WISC sum	Species	-0.981	0.644		
Meconium Adj.[c]	Roseburia faecis	WISC sum	Interaction	2.556	2.436		
Meconium Adj.[c]	Roseburia intestinalis	Block Design	Acetaminophen	1.012	1.038	0.769	0.896
Meconium Adj.[c]	Roseburia intestinalis	Block Design	Species	0.338	0.631		
Meconium Adj.[c]	Roseburia intestinalis	Block Design	Interaction	0.062	0.969		
Meconium Adj.[c]	Roseburia intestinalis	Coding	Acetaminophen	0.851	0.887	0.103	0.652
Meconium Adj.[c]	Roseburia intestinalis	Coding	Species	0.492	0.539		
Meconium Adj.[c]	Roseburia intestinalis	Coding	Interaction	0.651	0.827		
Meconium Adj.[c]	Roseburia intestinalis	Digit span	Acetaminophen	-0.083	0.752	0.559	0.836
Meconium Adj.[c]	Roseburia intestinalis	Digit span	Species	0.847	0.457		
Meconium Adj.[c]	Roseburia intestinalis	Digit span	Interaction	-0.355	0.702		
Meconium Adj.[c]	Roseburia intestinalis	Information	Acetaminophen	0.326	0.727	0.046	0.648
Meconium Adj.[c]	Roseburia intestinalis	Information	Species	0.298	0.441		
Meconium Adj.[c]	Roseburia intestinalis	Information	Interaction	1.162	0.678		
Meconium Adj.[c]	Roseburia intestinalis	QTAC	Acetaminophen	-3.082	2.652	0.749	0.896
Meconium Adj.[c]	Roseburia intestinalis	QTAC	Species	-1.119	1.612		
Meconium Adj.[c]	Roseburia intestinalis	QTAC	Interaction	-3.959	2.475		
Meconium Adj.[c]	Roseburia intestinalis	Vocabulary	Acetaminophen	0.145	1.172	0.944	0.978
Meconium Adj.[c]	Roseburia intestinalis	Vocabulary	Species	-0.105	0.712		
Meconium Adj.[c]	Roseburia intestinalis	Vocabulary	Interaction	1.055	1.094		
Meconium Adj.[c]	Roseburia intestinalis	WISC sum	Acetaminophen	2.251	2.626	0.174	0.678
Meconium Adj.[c]	Roseburia intestinalis	WISC sum	Species	1.869	1.596		
Meconium Adj.[c]	Roseburia intestinalis	WISC sum	Interaction	2.574	2.451		
Meconium Adj.[c]	Ruminococcus bicirculans	Block Design	Acetaminophen	1.129	1.107	0.693	0.873
Meconium Adj.[c]	Ruminococcus bicirculans	Block Design	Species	-0.037	0.355		
Meconium Adj.[c]	Ruminococcus bicirculans	Block Design	Interaction	0.141	0.989		
Meconium Adj.[c]	Ruminococcus bicirculans	Coding	Acetaminophen	-0.723	0.929	0.036	0.648
Meconium Adj.[c]	Ruminococcus bicirculans	Coding	Species	-0.369	0.298		
Meconium Adj.[c]	Ruminococcus bicirculans	Coding	Interaction	0.243	0.831		
Meconium Adj.[c]	Ruminococcus bicirculans	Digit span	Acetaminophen	0.035	0.847	0.477	0.836
Meconium Adj.[c]	Ruminococcus bicirculans	Digit span	Species	0.184	0.272		

Exposure Window	Species	Outcome	Variable	Effect Estimate[a]	Std. Error	LRT[b]	
						p-value	q-value
Meconium Adj.[c]	Ruminococcus bicirculans	Digit span	Interaction	0.018	0.758		
Meconium Adj.[c]	Ruminococcus bicirculans	Information	Acetaminophen	-0.442	0.817	0.684	0.867
Meconium Adj.[c]	Ruminococcus bicirculans	Information	Species	-0.098	0.262		
Meconium Adj.[c]	Ruminococcus bicirculans	Information	Interaction	0.936	0.73		
Meconium Adj.[c]	Ruminococcus bicirculans	QTAC	Acetaminophen	-5.057	2.769	0.099	0.652
Meconium Adj.[c]	Ruminococcus bicirculans	QTAC	Species	-0.634	0.888		
Meconium Adj.[c]	Ruminococcus bicirculans	QTAC	Interaction	-4.87	2.475		
Meconium Adj.[c]	Ruminococcus bicirculans	Vocabulary	Acetaminophen	-0.718	1.162	0.082	0.652
Meconium Adj.[c]	Ruminococcus bicirculans	Vocabulary	Species	-0.928	0.372		
Meconium Adj.[c]	Ruminococcus bicirculans	Vocabulary	Interaction	0.391	1.038		
Meconium Adj.[c]	Ruminococcus bicirculans	WISC sum	Acetaminophen	-0.718	2.784	0.235	0.735
Meconium Adj.[c]	Ruminococcus bicirculans	WISC sum	Species	-1.247	0.892		
Meconium Adj.[c]	Ruminococcus bicirculans	WISC sum	Interaction	1.729	2.488		
Meconium Adj.[c]	Ruminococcus bromii	Block Design	Acetaminophen	-0.976	1.5	0.113	0.652
Meconium Adj.[c]	Ruminococcus bromii	Block Design	Species	-0.038	0.089		
Meconium Adj.[c]	Ruminococcus bromii	Block Design	Interaction	0.13	0.935		
Meconium Adj.[c]	Ruminococcus bromii	Coding	Acetaminophen	-0.397	1.321	0.656	0.856
Meconium Adj.[c]	Ruminococcus bromii	Coding	Species	0.059	0.078		
Meconium Adj.[c]	Ruminococcus bromii	Coding	Interaction	0.672	0.824		
Meconium Adj.[c]	Ruminococcus bromii	Digit span	Acetaminophen	-1.538	1.135	0.19	0.691
Meconium Adj.[c]	Ruminococcus bromii	Digit span	Species	0.023	0.067		
Meconium Adj.[c]	Ruminococcus bromii	Digit span	Interaction	-0.056	0.707		
Meconium Adj.[c]	Ruminococcus bromii	Information	Acetaminophen	-2.301	1.061	0.018	0.546
Meconium Adj.[c]	Ruminococcus bromii	Information	Species	-0.04	0.063		
Meconium Adj.[c]	Ruminococcus bromii	Information	Interaction	1.01	0.661		
Meconium Adj.[c]	Ruminococcus bromii	QTAC	Acetaminophen	-1.289	3.97	0.614	0.854
Meconium Adj.[c]	Ruminococcus bromii	QTAC	Species	0.064	0.235		
Meconium Adj.[c]	Ruminococcus bromii	QTAC	Interaction	-4.171	2.474		
Meconium Adj.[c]	Ruminococcus bromii	Vocabulary	Acetaminophen	-2.349	1.662	0.054	0.648
Meconium Adj.[c]	Ruminococcus bromii	Vocabulary	Species	-0.052	0.098		
Meconium Adj.[c]	Ruminococcus bromii	Vocabulary	Interaction	1.027	1.036		
Meconium Adj.[c]	Ruminococcus bromii	WISC sum	Acetaminophen	-7.562	3.479	0.004	0.515
Meconium Adj.[c]	Ruminococcus bromii	WISC sum	Species	-0.049	0.206		
Meconium Adj.[c]	Ruminococcus bromii	WISC sum	Interaction	2.784	2.168		
Meconium Adj.[c]	Ruminococcus lactaris	Block Design	Acetaminophen	0.721	1.103	0.718	0.882
Meconium Adj.[c]	Ruminococcus lactaris	Block Design	Species	0.315	0.372		

Exposure Window	Species	Outcome	Variable	Effect Estimate[a]	Std. Error	LRT[b]	
						p-value	q-value
Meconium Adj.[c]	Ruminococcus lactaris	Block Design	Interaction	0.152	0.95		
Meconium Adj.[c]	Ruminococcus lactaris	Coding	Acetaminophen	0.317	0.982	0.898	0.955
Meconium Adj.[c]	Ruminococcus lactaris	Coding	Species	0.084	0.331		
Meconium Adj.[c]	Ruminococcus lactaris	Coding	Interaction	0.562	0.846		
Meconium Adj.[c]	Ruminococcus lactaris	Digit span	Acetaminophen	-0.89	0.83	0.143	0.652
Meconium Adj.[c]	Ruminococcus lactaris	Digit span	Species	-0.472	0.28		
Meconium Adj.[c]	Ruminococcus lactaris	Digit span	Interaction	-0.01	0.715		
Meconium Adj.[c]	Ruminococcus lactaris	Information	Acetaminophen	0.398	0.804	0.098	0.652
Meconium Adj.[c]	Ruminococcus lactaris	Information	Species	0.211	0.271		
Meconium Adj.[c]	Ruminococcus lactaris	Information	Interaction	0.899	0.693		
Meconium Adj.[c]	Ruminococcus lactaris	QTAC	Acetaminophen	-5.825	2.745	0.031	0.643
Meconium Adj.[c]	Ruminococcus lactaris	QTAC	Species	-1.002	0.926		
Meconium Adj.[c]	Ruminococcus lactaris	QTAC	Interaction	-3.715	2.364		
Meconium Adj.[c]	Ruminococcus lactaris	Vocabulary	Acetaminophen	0.249	1.261	0.923	0.972
Meconium Adj.[c]	Ruminococcus lactaris	Vocabulary	Species	0.146	0.425		
Meconium Adj.[c]	Ruminococcus lactaris	Vocabulary	Interaction	1.016	1.086		
Meconium Adj.[c]	Ruminococcus lactaris	WISC sum	Acetaminophen	0.795	2.882	0.992	0.999
Meconium Adj.[c]	Ruminococcus lactaris	WISC sum	Species	0.284	0.972		
Meconium Adj.[c]	Ruminococcus lactaris	WISC sum	Interaction	2.619	2.482		
Meconium Adj.[c]	Ruminococcus torques	Block Design	Acetaminophen	2.665	1.458	0.102	0.652
Meconium Adj.[c]	Ruminococcus torques	Block Design	Species	0.44	0.214		
Meconium Adj.[c]	Ruminococcus torques	Block Design	Interaction	-0.052	0.919		
Meconium Adj.[c]	Ruminococcus torques	Coding	Acetaminophen	0.431	1.314	0.824	0.905
Meconium Adj.[c]	Ruminococcus torques	Coding	Species	-0.178	0.193		
Meconium Adj.[c]	Ruminococcus torques	Coding	Interaction	0.583	0.828		
Meconium Adj.[c]	Ruminococcus torques	Digit span	Acetaminophen	-0.116	1.118	0.836	0.908
Meconium Adj.[c]	Ruminococcus torques	Digit span	Species	-0.255	0.164		
Meconium Adj.[c]	Ruminococcus torques	Digit span	Interaction	-0.1	0.704		
Meconium Adj.[c]	Ruminococcus torques	Information	Acetaminophen	-0.134	1.127	0.841	0.91
Meconium Adj.[c]	Ruminococcus torques	Information	Species	-0.054	0.165		
Meconium Adj.[c]	Ruminococcus torques	Information	Interaction	1.007	0.71		
Meconium Adj.[c]	Ruminococcus torques	QTAC	Acetaminophen	-6.686	3.801	0.148	0.652
Meconium Adj.[c]	Ruminococcus torques	QTAC	Species	0.017	0.557		
Meconium Adj.[c]	Ruminococcus torques	QTAC	Interaction	-3.935	2.395		
Meconium Adj.[c]	Ruminococcus torques	Vocabulary	Acetaminophen	0.756	1.723	0.639	0.854
Meconium Adj.[c]	Ruminococcus torques	Vocabulary	Species	0.044	0.253		

Exposure Window	Species	Outcome	Variable	Effect Estimate[a]	Std. Error	LRT[b]	
						p-value	q-value
Meconium Adj.[c]	Ruminococcus torques	Vocabulary	Interaction	0.983	1.086		
Meconium Adj.[c]	Ruminococcus torques	WISC sum	Acetaminophen	3.602	3.88	0.308	0.783
Meconium Adj.[c]	Ruminococcus torques	WISC sum	Species	-0.003	0.569		
Meconium Adj.[c]	Ruminococcus torques	WISC sum	Interaction	2.421	2.445		
Meconium Adj.[c]	Streptococcus thermophilus	Block Design	Acetaminophen	1.402	1.173	0.561	0.836
Meconium Adj.[c]	Streptococcus thermophilus	Block Design	Species	0.565	0.523		
Meconium Adj.[c]	Streptococcus thermophilus	Block Design	Interaction	0.015	0.965		
Meconium Adj.[c]	Streptococcus thermophilus	Coding	Acetaminophen	0.128	1.009	0.927	0.972
Meconium Adj.[c]	Streptococcus thermophilus	Coding	Species	-0.446	0.45		
Meconium Adj.[c]	Streptococcus thermophilus	Coding	Interaction	0.828	0.83		
Meconium Adj.[c]	Streptococcus thermophilus	Digit span	Acetaminophen	-0.996	0.875	0.085	0.652
Meconium Adj.[c]	Streptococcus thermophilus	Digit span	Species	-0.191	0.39		
Meconium Adj.[c]	Streptococcus thermophilus	Digit span	Interaction	-0.415	0.72		
Meconium Adj.[c]	Streptococcus thermophilus	Information	Acetaminophen	0.288	0.86	0.316	0.783
Meconium Adj.[c]	Streptococcus thermophilus	Information	Species	0.522	0.383		
Meconium Adj.[c]	Streptococcus thermophilus	Information	Interaction	0.963	0.708		
Meconium Adj.[c]	Streptococcus thermophilus	QTAC	Acetaminophen	-3.806	2.562	0.999	0.999
Meconium Adj.[c]	Streptococcus thermophilus	QTAC	Species	-2.897	1.142		
Meconium Adj.[c]	Streptococcus thermophilus	QTAC	Interaction	-2.728	2.108		
Meconium Adj.[c]	Streptococcus thermophilus	Vocabulary	Acetaminophen	0.871	1.287	0.52	0.836
Meconium Adj.[c]	Streptococcus thermophilus	Vocabulary	Species	0.899	0.574		
Meconium Adj.[c]	Streptococcus thermophilus	Vocabulary	Interaction	0.779	1.059		
Meconium Adj.[c]	Streptococcus thermophilus	WISC sum	Acetaminophen	1.694	3.007	0.755	0.896
Meconium Adj.[c]	Streptococcus thermophilus	WISC sum	Species	1.349	1.341		
Meconium Adj.[c]	Streptococcus thermophilus	WISC sum	Interaction	2.171	2.475		

[a] Models are adjusted for whether the child was ever breastfed, sex, mode of birth, and socioeconomic status. Effect estimates for acetaminophen are the difference in outcome score between exposed and unexposed. Effect estimates for species are the change in outcome score for each 1% increase in species relative abundance. Effect estimates for the interaction are for the multiplicative interaction between acetaminophen and species relative abundance.

[b] Likelihood ratio test comparing to model without interaction

[c] Meconium Adj. includes cross-sectional exposure in the model

Table S12. Caffeine-pathway interactions in association with neurodevelopment

Exposure Window	Pathway	Outcome	Variable	Effect Estimate[a]	Std. Error	LRT[b]	
						p-value	q-value
Meconium	ARGSYN.PWY	Block Design	Caffeine	2.227	2.505	0.275	0.95
Meconium	ARGSYN.PWY	Block Design	Pathway	291.368	325.422		
Meconium	ARGSYN.PWY	Block Design	Interaction	-37.864	37.686		
Meconium	ARGSYN.PWY	Coding	Caffeine	-0.558	2.244	0.751	0.95
Meconium	ARGSYN.PWY	Coding	Pathway	-49.589	291.442		
Meconium	ARGSYN.PWY	Coding	Interaction	9.801	33.751		
Meconium	ARGSYN.PWY	Digit span	Caffeine	-2.256	1.778	0.242	0.95
Meconium	ARGSYN.PWY	Digit span	Pathway	-149.726	231		
Meconium	ARGSYN.PWY	Digit span	Interaction	28.825	26.751		
Meconium	ARGSYN.PWY	Information	Caffeine	-1.656	1.908	0.377	0.95
Meconium	ARGSYN.PWY	Information	Pathway	-214.754	247.884		
Meconium	ARGSYN.PWY	Information	Interaction	23.308	28.706		
Meconium	ARGSYN.PWY	QTAC	Caffeine	-11.93	6.566	0.068	0.95
Meconium	ARGSYN.PWY	QTAC	Pathway	-1313.095	852.785		
Meconium	ARGSYN.PWY	QTAC	Interaction	167.586	98.758		
Meconium	ARGSYN.PWY	Vocabulary	Caffeine	-1.853	2.896	0.519	0.95
Meconium	ARGSYN.PWY	Vocabulary	Pathway	-206.724	376.209		
Meconium	ARGSYN.PWY	Vocabulary	Interaction	25.778	43.567		
Meconium	ARGSYN.PWY	WISC sum	Caffeine	-4.097	6.517	0.579	0.95
Meconium	ARGSYN.PWY	WISC sum	Pathway	-329.425	846.436		
Meconium	ARGSYN.PWY	WISC sum	Interaction	49.848	98.022		
Meconium	ARGSYNBSUB.PWY	Block Design	Caffeine	2.249	2.426	0.256	0.95
Meconium	ARGSYNBSUB.PWY	Block Design	Pathway	279.3	298.905		
Meconium	ARGSYNBSUB.PWY	Block Design	Interaction	-36.733	35.095		
Meconium	ARGSYNBSUB.PWY	Coding	Caffeine	-0.442	2.178	0.788	0.95
Meconium	ARGSYNBSUB.PWY	Coding	Pathway	-37.518	268.311		
Meconium	ARGSYNBSUB.PWY	Coding	Interaction	7.733	31.502		
Meconium	ARGSYNBSUB.PWY	Digit span	Caffeine	-2.484	1.722	0.178	0.95
Meconium	ARGSYNBSUB.PWY	Digit span	Pathway	-172.889	212.127		
Meconium	ARGSYNBSUB.PWY	Digit span	Interaction	30.986	24.906		
Meconium	ARGSYNBSUB.PWY	Information	Caffeine	-1.698	1.848	0.348	0.95
Meconium	ARGSYNBSUB.PWY	Information	Pathway	-209.076	227.659		
Meconium	ARGSYNBSUB.PWY	Information	Interaction	23.049	26.73		
Meconium	ARGSYNBSUB.PWY	QTAC	Caffeine	-10.965	6.404	0.087	0.95
Meconium	ARGSYNBSUB.PWY	QTAC	Pathway	-1160.62	789.007		

Exposure Window	Pathway	Outcome	Variable	Effect Estimate[a]	Std. Error	LRT[b]	
						p-value	q-value
Meconium	ARGSYNBSUB.PWY	QTAC	Interaction	147.316	92.638		
Meconium	ARGSYNBSUB.PWY	Vocabulary	Caffeine	-1.889	2.807	0.496	0.95
Meconium	ARGSYNBSUB.PWY	Vocabulary	Pathway	-206.295	345.8		
Meconium	ARGSYNBSUB.PWY	Vocabulary	Interaction	25.335	40.601		
Meconium	ARGSYNBSUB.PWY	WISC sum	Caffeine	-4.263	6.319	0.548	0.95
Meconium	ARGSYNBSUB.PWY	WISC sum	Pathway	-346.478	778.592		
Meconium	ARGSYNBSUB.PWY	WISC sum	Interaction	50.37	91.415		
Meconium	ARO.PWY	Block Design	Caffeine	3.345	2.443	0.109	0.95
Meconium	ARO.PWY	Block Design	Pathway	414.871	313.106		
Meconium	ARO.PWY	Block Design	Interaction	-54.507	36.695		
Meconium	ARO.PWY	Coding	Caffeine	-0.619	2.217	0.73	0.95
Meconium	ARO.PWY	Coding	Pathway	-43.024	284.063		
Meconium	ARO.PWY	Coding	Interaction	10.519	33.291		
Meconium	ARO.PWY	Digit span	Caffeine	-2.469	1.792	0.199	0.95
Meconium	ARO.PWY	Digit span	Pathway	-190.23	229.621		
Meconium	ARO.PWY	Digit span	Interaction	31.893	26.911		
Meconium	ARO.PWY	Information	Caffeine	-1.668	1.887	0.365	0.95
Meconium	ARO.PWY	Information	Pathway	-223.318	241.763		
Meconium	ARO.PWY	Information	Interaction	23.562	28.334		
Meconium	ARO.PWY	QTAC	Caffeine	-8.216	6.657	0.225	0.95
Meconium	ARO.PWY	QTAC	Pathway	-923.105	853.055		
Meconium	ARO.PWY	QTAC	Interaction	111.8	99.974		
Meconium	ARO.PWY	Vocabulary	Caffeine	-1.088	2.872	0.713	0.95
Meconium	ARO.PWY	Vocabulary	Pathway	-154.186	368.061		
Meconium	ARO.PWY	Vocabulary	Interaction	14.505	43.135		
Meconium	ARO.PWY	WISC sum	Caffeine	-2.498	6.493	0.771	0.95
Meconium	ARO.PWY	WISC sum	Pathway	-195.888	832.04		
Meconium	ARO.PWY	WISC sum	Interaction	25.973	97.511		
Meconium	BRANCHED.CHAIN.AA.SYN.PWY	Block Design	Caffeine	3.226	2.146	0.077	0.95
Meconium	BRANCHED.CHAIN.AA.SYN.PWY	Block Design	Pathway	405.339	279.11		
Meconium	BRANCHED.CHAIN.AA.SYN.PWY	Block Design	Interaction	-54.99	33.505		
Meconium	BRANCHED.CHAIN.AA.SYN.PWY	Coding	Caffeine	0.334	1.968	0.9	0.95
Meconium	BRANCHED.CHAIN.AA.SYN.PWY	Coding	Pathway	-0.305	256.006		
Meconium	BRANCHED.CHAIN.AA.SYN.PWY	Coding	Interaction	-3.526	30.731		
Meconium	BRANCHED.CHAIN.AA.SYN.PWY	Digit span	Caffeine	-2.359	1.619	0.171	0.95
Meconium	BRANCHED.CHAIN.AA.SYN.PWY	Digit span	Pathway	-234.152	210.528		

Exposure Window	Pathway	Outcome	Variable	Effect Estimate[a]	Std. Error	LRT[b]	
						p-value	q-value
Meconium	BRANCHED.CHAIN.AA.SYN.PWY	Digit span	Interaction	31.958	25.272		
Meconium	BRANCHED.CHAIN.AA.SYN.PWY	Information	Caffeine	0.499	1.641	0.689	0.95
Meconium	BRANCHED.CHAIN.AA.SYN.PWY	Information	Pathway	4.555	213.439		
Meconium	BRANCHED.CHAIN.AA.SYN.PWY	Information	Interaction	-9.379	25.622		
Meconium	BRANCHED.CHAIN.AA.SYN.PWY	QTAC	Caffeine	-11.243	5.739	0.05	0.95
Meconium	BRANCHED.CHAIN.AA.SYN.PWY	QTAC	Pathway	-1280.276	746.382		
Meconium	BRANCHED.CHAIN.AA.SYN.PWY	QTAC	Interaction	163.709	89.597		
Meconium	BRANCHED.CHAIN.AA.SYN.PWY	Vocabulary	Caffeine	-0.715	2.455	0.786	0.95
Meconium	BRANCHED.CHAIN.AA.SYN.PWY	Vocabulary	Pathway	-204.293	319.339		
Meconium	BRANCHED.CHAIN.AA.SYN.PWY	Vocabulary	Interaction	9.543	38.334		
Meconium	BRANCHED.CHAIN.AA.SYN.PWY	WISC sum	Caffeine	0.984	5.592	0.741	0.95
Meconium	BRANCHED.CHAIN.AA.SYN.PWY	WISC sum	Pathway	-28.856	727.303		
Meconium	BRANCHED.CHAIN.AA.SYN.PWY	WISC sum	Interaction	-26.393	87.307		
Meconium	CALVIN.PWY	Block Design	Caffeine	1.404	1.568	0.238	0.95
Meconium	CALVIN.PWY	Block Design	Pathway	242.218	240.817		
Meconium	CALVIN.PWY	Block Design	Interaction	-29.909	27.489		
Meconium	CALVIN.PWY	Coding	Caffeine	0.636	1.409	0.677	0.95
Meconium	CALVIN.PWY	Coding	Pathway	72.439	216.367		
Meconium	CALVIN.PWY	Coding	Interaction	-9.405	24.698		
Meconium	CALVIN.PWY	Digit span	Caffeine	-1.302	1.147	0.356	0.95
Meconium	CALVIN.PWY	Digit span	Pathway	-87.082	176.191		
Meconium	CALVIN.PWY	Digit span	Interaction	17.049	20.112		
Meconium	CALVIN.PWY	Information	Caffeine	-0.378	1.205	0.811	0.95
Meconium	CALVIN.PWY	Information	Pathway	-45.196	185.084		
Meconium	CALVIN.PWY	Information	Interaction	4.633	21.127		
Meconium	CALVIN.PWY	QTAC	Caffeine	-4.303	4.222	0.357	0.95
Meconium	CALVIN.PWY	QTAC	Pathway	-603.266	648.565		
Meconium	CALVIN.PWY	QTAC	Interaction	62.599	74.033		
Meconium	CALVIN.PWY	Vocabulary	Caffeine	-0.208	1.82	0.966	0.971
Meconium	CALVIN.PWY	Vocabulary	Pathway	-22.999	279.61		
Meconium	CALVIN.PWY	Vocabulary	Interaction	1.251	31.917		
Meconium	CALVIN.PWY	WISC sum	Caffeine	0.152	4.103	0.803	0.95
Meconium	CALVIN.PWY	WISC sum	Pathway	159.379	630.236		
Meconium	CALVIN.PWY	WISC sum	Interaction	-16.382	71.941		
Meconium	COA.PWY.1	Block Design	Caffeine	3.876	4.642	0.329	0.95
Meconium	COA.PWY.1	Block Design	Pathway	520.574	637.503		

Exposure Window	Pathway	Outcome	Variable	Effect Estimate[a]	Std. Error	LRT[b]	
						p-value	q-value
Meconium	COA.PWY.1	Block Design	Interaction	-69.137	76.985		
Meconium	COA.PWY.1	Coding	Caffeine	-2.043	4.131	0.571	0.95
Meconium	COA.PWY.1	Coding	Pathway	-226.931	567.411		
Meconium	COA.PWY.1	Coding	Interaction	35.56	68.521		
Meconium	COA.PWY.1	Digit span	Caffeine	-4.137	3.337	0.214	0.95
Meconium	COA.PWY.1	Digit span	Pathway	-389.015	458.306		
Meconium	COA.PWY.1	Digit span	Interaction	63.352	55.345		
Meconium	COA.PWY.1	Information	Caffeine	-6.246	3.427	0.055	0.95
Meconium	COA.PWY.1	Information	Pathway	-832.292	470.738		
Meconium	COA.PWY.1	Information	Interaction	101.743	56.847		
Meconium	COA.PWY.1	QTAC	Caffeine	-12.43	12.414	0.308	0.95
Meconium	COA.PWY.1	QTAC	Pathway	-1395.434	1704.9		
Meconium	COA.PWY.1	QTAC	Interaction	193.026	205.885		
Meconium	COA.PWY.1	Vocabulary	Caffeine	-1.185	5.348	0.83	0.95
Meconium	COA.PWY.1	Vocabulary	Pathway	-218.693	734.546		
Meconium	COA.PWY.1	Vocabulary	Interaction	17.383	88.704		
Meconium	COA.PWY.1	WISC sum	Caffeine	-9.736	12.03	0.416	0.95
Meconium	COA.PWY.1	WISC sum	Pathway	-1146.358	1652.159		
Meconium	COA.PWY.1	WISC sum	Interaction	148.902	199.516		
Meconium	COMPLETE.ARO.PWY	Block Design	Caffeine	3.142	2.32	0.11	0.95
Meconium	COMPLETE.ARO.PWY	Block Design	Pathway	432.264	312.357		
Meconium	COMPLETE.ARO.PWY	Block Design	Interaction	-55.225	37.248		
Meconium	COMPLETE.ARO.PWY	Coding	Caffeine	-0.146	2.109	0.902	0.95
Meconium	COMPLETE.ARO.PWY	Coding	Pathway	-3.559	283.947		
Meconium	COMPLETE.ARO.PWY	Coding	Interaction	3.817	33.86		
Meconium	COMPLETE.ARO.PWY	Digit span	Caffeine	-2.479	1.693	0.17	0.95
Meconium	COMPLETE.ARO.PWY	Digit span	Pathway	-209.411	227.873		
Meconium	COMPLETE.ARO.PWY	Digit span	Interaction	34.437	27.174		
Meconium	COMPLETE.ARO.PWY	Information	Caffeine	-1.234	1.795	0.493	0.95
Meconium	COMPLETE.ARO.PWY	Information	Pathway	-167.856	241.691		
Meconium	COMPLETE.ARO.PWY	Information	Interaction	18.109	28.821		
Meconium	COMPLETE.ARO.PWY	QTAC	Caffeine	-8.245	6.281	0.195	0.95
Meconium	COMPLETE.ARO.PWY	QTAC	Pathway	-1049.802	845.548		
Meconium	COMPLETE.ARO.PWY	QTAC	Interaction	120.482	100.831		
Meconium	COMPLETE.ARO.PWY	Vocabulary	Caffeine	-1.081	2.721	0.702	0.95
Meconium	COMPLETE.ARO.PWY	Vocabulary	Pathway	-151.615	366.334		

Exposure Window	Pathway	Outcome	Variable	Effect Estimate[a]	Std. Error	LRT[b]	
						p-value	q-value
Meconium	COMPLETE.ARO.PWY	Vocabulary	Interaction	15.301	43.685		
Meconium	COMPLETE.ARO.PWY	WISC sum	Caffeine	-1.798	6.149	0.856	0.95
Meconium	COMPLETE.ARO.PWY	WISC sum	Pathway	-100.177	827.777		
Meconium	COMPLETE.ARO.PWY	WISC sum	Interaction	16.439	98.712		
Meconium	DTDPRHAMSYN.PWY	Block Design	Caffeine	-0.099	1.222	0.901	0.95
Meconium	DTDPRHAMSYN.PWY	Block Design	Pathway	-42.6	181.193		
Meconium	DTDPRHAMSYN.PWY	Block Design	Interaction	-2.322	20.321		
Meconium	DTDPRHAMSYN.PWY	Coding	Caffeine	1.891	1.064	0.07	0.95
Meconium	DTDPRHAMSYN.PWY	Coding	Pathway	226.913	157.744		
Meconium	DTDPRHAMSYN.PWY	Coding	Interaction	-29.814	17.691		
Meconium	DTDPRHAMSYN.PWY	Digit span	Caffeine	-0.524	0.927	0.807	0.95
Meconium	DTDPRHAMSYN.PWY	Digit span	Pathway	-24.21	137.377		
Meconium	DTDPRHAMSYN.PWY	Digit span	Interaction	3.442	15.407		
Meconium	DTDPRHAMSYN.PWY	Information	Caffeine	0.106	0.943	0.801	0.95
Meconium	DTDPRHAMSYN.PWY	Information	Pathway	18.042	139.833		
Meconium	DTDPRHAMSYN.PWY	Information	Interaction	-3.61	15.682		
Meconium	DTDPRHAMSYN.PWY	QTAC	Caffeine	-3.566	3.318	0.355	0.95
Meconium	DTDPRHAMSYN.PWY	QTAC	Pathway	-408.321	491.824		
Meconium	DTDPRHAMSYN.PWY	QTAC	Interaction	46.886	55.158		
Meconium	DTDPRHAMSYN.PWY	Vocabulary	Caffeine	-0.189	1.427	0.967	0.971
Meconium	DTDPRHAMSYN.PWY	Vocabulary	Pathway	-14.097	211.585		
Meconium	DTDPRHAMSYN.PWY	Vocabulary	Interaction	0.89	23.729		
Meconium	DTDPRHAMSYN.PWY	WISC sum	Caffeine	1.185	3.178	0.517	0.95
Meconium	DTDPRHAMSYN.PWY	WISC sum	Pathway	164.048	471.107		
Meconium	DTDPRHAMSYN.PWY	WISC sum	Interaction	-31.414	52.835		
Meconium	GLUTORN.PWY	Block Design	Caffeine	2.16	2.012	0.186	0.95
Meconium	GLUTORN.PWY	Block Design	Pathway	326.815	286.982		
Meconium	GLUTORN.PWY	Block Design	Interaction	-40.848	33.431		
Meconium	GLUTORN.PWY	Coding	Caffeine	-0.198	1.812	0.861	0.95
Meconium	GLUTORN.PWY	Coding	Pathway	-12.823	258.454		
Meconium	GLUTORN.PWY	Coding	Interaction	4.815	30.108		
Meconium	GLUTORN.PWY	Digit span	Caffeine	-2.301	1.454	0.143	0.95
Meconium	GLUTORN.PWY	Digit span	Pathway	-212.907	207.392		
Meconium	GLUTORN.PWY	Digit span	Interaction	32.744	24.16		
Meconium	GLUTORN.PWY	Information	Caffeine	-1.282	1.534	0.401	0.95
Meconium	GLUTORN.PWY	Information	Pathway	-195.533	218.815		

Exposure Window	Pathway	Outcome	Variable	Effect Estimate[a]	Std. Error	LRT[b]	
						p-value	q-value
Meconium	GLUTORN.PWY	Information	Interaction	19.659	25.49		
Meconium	GLUTORN.PWY	QTAC	Caffeine	-9.791	5.285		
Meconium	GLUTORN.PWY	QTAC	Pathway	-1147.982	753.883	0.067	0.95
Meconium	GLUTORN.PWY	QTAC	Interaction	149.579	87.822		
Meconium	GLUTORN.PWY	Vocabulary	Caffeine	-1.632	2.334		
Meconium	GLUTORN.PWY	Vocabulary	Pathway	-200.585	333.003	0.485	0.95
Meconium	GLUTORN.PWY	Vocabulary	Interaction	24.84	38.792		
Meconium	GLUTORN.PWY	WISC sum	Caffeine	-3.252	5.269		
Meconium	GLUTORN.PWY	WISC sum	Pathway	-295.033	751.683	0.607	0.95
Meconium	GLUTORN.PWY	WISC sum	Interaction	41.21	87.565		
Meconium	GLYCOGENSYNTH.PWY	Block Design	Caffeine	0.44	1.23		
Meconium	GLYCOGENSYNTH.PWY	Block Design	Pathway	108.026	190.085	0.511	0.95
Meconium	GLYCOGENSYNTH.PWY	Block Design	Interaction	-12.65	21.017		
Meconium	GLYCOGENSYNTH.PWY	Coding	Caffeine	0.022	1.086		
Meconium	GLYCOGENSYNTH.PWY	Coding	Pathway	-51.513	167.907	0.911	0.95
Meconium	GLYCOGENSYNTH.PWY	Coding	Interaction	1.896	18.565		
Meconium	GLYCOGENSYNTH.PWY	Digit span	Caffeine	-0.449	0.907		
Meconium	GLYCOGENSYNTH.PWY	Digit span	Pathway	18.896	140.246	0.898	0.95
Meconium	GLYCOGENSYNTH.PWY	Digit span	Interaction	1.814	15.506		
Meconium	GLYCOGENSYNTH.PWY	Information	Caffeine	-0.225	0.936		
Meconium	GLYCOGENSYNTH.PWY	Information	Pathway	-18.113	144.678	0.898	0.95
Meconium	GLYCOGENSYNTH.PWY	Information	Interaction	1.878	15.997		
Meconium	GLYCOGENSYNTH.PWY	QTAC	Caffeine	-4.459	3.24		
Meconium	GLYCOGENSYNTH.PWY	QTAC	Pathway	-660.089	500.801	0.203	0.95
Meconium	GLYCOGENSYNTH.PWY	QTAC	Interaction	64.995	55.371		
Meconium	GLYCOGENSYNTH.PWY	Vocabulary	Caffeine	-0.841	1.409		
Meconium	GLYCOGENSYNTH.PWY	Vocabulary	Pathway	-109.908	217.846	0.581	0.95
Meconium	GLYCOGENSYNTH.PWY	Vocabulary	Interaction	12.184	24.086		
Meconium	GLYCOGENSYNTH.PWY	WISC sum	Caffeine	-1.053	3.187		
Meconium	GLYCOGENSYNTH.PWY	WISC sum	Pathway	-52.611	492.695	0.918	0.95
Meconium	GLYCOGENSYNTH.PWY	WISC sum	Interaction	5.122	54.475		
Meconium	ILEUSYN.PWY	Block Design	Caffeine	3.986	2.602		
Meconium	ILEUSYN.PWY	Block Design	Pathway	465.085	299.929	0.076	0.95
Meconium	ILEUSYN.PWY	Block Design	Interaction	-57.311	34.783		
Meconium	ILEUSYN.PWY	Coding	Caffeine	0.876	2.383	0.722	0.95
Meconium	ILEUSYN.PWY	Coding	Pathway	74.897	274.635		

Exposure Window	Pathway	Outcome	Variable	Effect Estimate[a]	Std. Error	LRT[b]	
						p-value	q-value
Meconium	ILEUSYN.PWY	Coding	Interaction	-10.365	31.85		
Meconium	ILEUSYN.PWY	Digit span	Caffeine	-2.911	1.893		
Meconium	ILEUSYN.PWY	Digit span	Pathway	-227.289	218.153	0.138	0.95
Meconium	ILEUSYN.PWY	Digit span	Interaction	34.686	25.3		
Meconium	ILEUSYN.PWY	Information	Caffeine	-2.608	1.93		
Meconium	ILEUSYN.PWY	Information	Pathway	-354.498	222.417	0.16	0.95
Meconium	ILEUSYN.PWY	Information	Interaction	33.517	25.794		
Meconium	ILEUSYN.PWY	QTAC	Caffeine	-9.006	7.056		
Meconium	ILEUSYN.PWY	QTAC	Pathway	-819.12	813.219	0.206	0.95
Meconium	ILEUSYN.PWY	QTAC	Interaction	109.909	94.31		
Meconium	ILEUSYN.PWY	Vocabulary	Caffeine	-2.544	3.03		
Meconium	ILEUSYN.PWY	Vocabulary	Pathway	-327.965	349.255	0.385	0.95
Meconium	ILEUSYN.PWY	Vocabulary	Interaction	32.309	40.504		
Meconium	ILEUSYN.PWY	WISC sum	Caffeine	-3.201	6.902		
Meconium	ILEUSYN.PWY	WISC sum	Pathway	-369.771	795.506	0.697	0.95
Meconium	ILEUSYN.PWY	WISC sum	Interaction	32.837	92.256		
Meconium	PEPTIDOGLYCANSYN.PWY	Block Design	Caffeine	-1.872	3.759		
Meconium	PEPTIDOGLYCANSYN.PWY	Block Design	Pathway	-220.2	487.388	0.644	0.95
Meconium	PEPTIDOGLYCANSYN.PWY	Block Design	Interaction	24.756	58.577		
Meconium	PEPTIDOGLYCANSYN.PWY	Coding	Caffeine	0.47	3.321		
Meconium	PEPTIDOGLYCANSYN.PWY	Coding	Pathway	115.332	430.704	0.899	0.95
Meconium	PEPTIDOGLYCANSYN.PWY	Coding	Interaction	-5.984	51.765		
Meconium	PEPTIDOGLYCANSYN.PWY	Digit span	Caffeine	-1.691	2.718		
Meconium	PEPTIDOGLYCANSYN.PWY	Digit span	Pathway	-54.871	352.442	0.587	0.95
Meconium	PEPTIDOGLYCANSYN.PWY	Digit span	Interaction	21.064	42.359		
Meconium	PEPTIDOGLYCANSYN.PWY	Information	Caffeine	-0.552	2.838		
Meconium	PEPTIDOGLYCANSYN.PWY	Information	Pathway	3.214	367.963	0.871	0.95
Meconium	PEPTIDOGLYCANSYN.PWY	Information	Interaction	6.58	44.224		
Meconium	PEPTIDOGLYCANSYN.PWY	QTAC	Caffeine	-20.323	9.605		
Meconium	PEPTIDOGLYCANSYN.PWY	QTAC	Pathway	-2323.232	1245.504	0.03	0.95
Meconium	PEPTIDOGLYCANSYN.PWY	QTAC	Interaction	304.338	149.693		
Meconium	PEPTIDOGLYCANSYN.PWY	Vocabulary	Caffeine	-2.329	4.255		
Meconium	PEPTIDOGLYCANSYN.PWY	Vocabulary	Pathway	-163.01	551.723	0.579	0.95
Meconium	PEPTIDOGLYCANSYN.PWY	Vocabulary	Interaction	33.75	66.31		
Meconium	PEPTIDOGLYCANSYN.PWY	WISC sum	Caffeine	-5.974	9.514	0.555	0.95
Meconium	PEPTIDOGLYCANSYN.PWY	WISC sum	Pathway	-319.535	1233.665		

Exposure Window	Pathway	Outcome	Variable	Effect Estimate[a]	Std. Error	LRT[b]	
						p-value	q-value
Meconium	PEPTIDOGLYCANSYN.PWY	WISC sum	Interaction	80.166	148.27		
Meconium	PWY.1042	Block Design	Caffeine	1.866	2.21	0.287	0.95
Meconium	PWY.1042	Block Design	Pathway	222.122	256.465		
Meconium	PWY.1042	Block Design	Interaction	-28.921	29.545		
Meconium	PWY.1042	Coding	Caffeine	0.944	1.976	0.639	0.95
Meconium	PWY.1042	Coding	Pathway	124.319	229.212		
Meconium	PWY.1042	Coding	Interaction	-11.354	26.405		
Meconium	PWY.1042	Digit span	Caffeine	-1.512	1.643	0.427	0.95
Meconium	PWY.1042	Digit span	Pathway	-102.679	190.62		
Meconium	PWY.1042	Digit span	Interaction	16.02	21.959		
Meconium	PWY.1042	Information	Caffeine	-0.919	1.687	0.601	0.95
Meconium	PWY.1042	Information	Pathway	-114.753	195.768		
Meconium	PWY.1042	Information	Interaction	10.789	22.552		
Meconium	PWY.1042	QTAC	Caffeine	-4.907	5.953	0.451	0.95
Meconium	PWY.1042	QTAC	Pathway	-379.247	690.735		
Meconium	PWY.1042	QTAC	Interaction	55.078	79.572		
Meconium	PWY.1042	Vocabulary	Caffeine	0.284	2.557	0.856	0.95
Meconium	PWY.1042	Vocabulary	Pathway	17.475	296.635		
Meconium	PWY.1042	Vocabulary	Interaction	-5.675	34.172		
Meconium	PWY.1042	WISC sum	Caffeine	0.662	5.778	0.787	0.95
Meconium	PWY.1042	WISC sum	Pathway	146.483	670.36		
Meconium	PWY.1042	WISC sum	Interaction	-19.141	77.225		
Meconium	PWY.3841	Block Design	Caffeine	1.354	4.24	0.671	0.95
Meconium	PWY.3841	Block Design	Pathway	219.827	684.493		
Meconium	PWY.3841	Block Design	Interaction	-30.999	79.6		
Meconium	PWY.3841	Coding	Caffeine	1.141	3.736	0.763	0.95
Meconium	PWY.3841	Coding	Pathway	260.444	603.232		
Meconium	PWY.3841	Coding	Interaction	-19.355	70.15		
Meconium	PWY.3841	Digit span	Caffeine	-2.541	3.009	0.416	0.95
Meconium	PWY.3841	Digit span	Pathway	-179.325	485.846		
Meconium	PWY.3841	Digit span	Interaction	42.189	56.499		
Meconium	PWY.3841	Information	Caffeine	-3.933	3.118	0.183	0.95
Meconium	PWY.3841	Information	Pathway	-499.029	503.401		
Meconium	PWY.3841	Information	Interaction	71.937	58.541		
Meconium	PWY.3841	QTAC	Caffeine	-5.637	11.382	0.642	0.95
Meconium	PWY.3841	QTAC	Pathway	-836.6	1837.609		

Exposure Window	Pathway	Outcome	Variable	Effect Estimate[a]	Std. Error	LRT[b]	
						p-value	q-value
Meconium	PWY.3841	QTAC	Interaction	90.882	213.696		
Meconium	PWY.3841	Vocabulary	Caffeine	0.614	4.866	0.865	0.95
Meconium	PWY.3841	Vocabulary	Pathway	128.102	785.641		
Meconium	PWY.3841	Vocabulary	Interaction	-14.205	91.362		
Meconium	PWY.3841	WISC sum	Caffeine	-3.364	10.822	0.79	0.95
Meconium	PWY.3841	WISC sum	Pathway	-69.98	1747.186		
Meconium	PWY.3841	WISC sum	Interaction	49.568	203.181		
Meconium	PWY.4242	Block Design	Caffeine	1.137	1.922	0.422	0.95
Meconium	PWY.4242	Block Design	Pathway	170.116	332.072		
Meconium	PWY.4242	Block Design	Interaction	-28.273	38.345		
Meconium	PWY.4242	Coding	Caffeine	0.399	1.727	0.845	0.95
Meconium	PWY.4242	Coding	Pathway	89.185	298.314		
Meconium	PWY.4242	Coding	Interaction	-6.171	34.447		
Meconium	PWY.4242	Digit span	Caffeine	-0.243	1.402	0.938	0.953
Meconium	PWY.4242	Digit span	Pathway	111.978	242.135		
Meconium	PWY.4242	Digit span	Interaction	-1.991	27.96		
Meconium	PWY.4242	Information	Caffeine	-0.74	1.476	0.64	0.95
Meconium	PWY.4242	Information	Pathway	-127.688	254.922		
Meconium	PWY.4242	Information	Interaction	12.591	29.436		
Meconium	PWY.4242	QTAC	Caffeine	-4.964	5.182	0.382	0.95
Meconium	PWY.4242	QTAC	Pathway	-579.136	895.148		
Meconium	PWY.4242	QTAC	Interaction	83.002	103.365		
Meconium	PWY.4242	Vocabulary	Caffeine	-0.854	2.186	0.708	0.95
Meconium	PWY.4242	Vocabulary	Pathway	-240.985	377.525		
Meconium	PWY.4242	Vocabulary	Interaction	14.938	43.594		
Meconium	PWY.4242	WISC sum	Caffeine	-0.301	5.036	0.923	0.95
Meconium	PWY.4242	WISC sum	Pathway	2.605	869.955		
Meconium	PWY.4242	WISC sum	Interaction	-8.906	100.456		
Meconium	PWY.5097	Block Design	Caffeine	2.006	2.868	0.381	0.95
Meconium	PWY.5097	Block Design	Pathway	386.988	449.574		
Meconium	PWY.5097	Block Design	Interaction	-40.965	50.889		
Meconium	PWY.5097	Coding	Caffeine	2.712	2.501	0.253	0.95
Meconium	PWY.5097	Coding	Pathway	491.311	392.052		
Meconium	PWY.5097	Coding	Interaction	-46.699	44.378		
Meconium	PWY.5097	Digit span	Caffeine	-1.931	2.008	0.387	0.95
Meconium	PWY.5097	Digit span	Pathway	-87.032	314.763		

Exposure Window	Pathway	Outcome	Variable	Effect Estimate[a]	Std. Error	LRT[b]	
						p-value	q-value
Meconium	PWY.5097	Digit span	Interaction	28.297	35.63		
Meconium	PWY.5097	Information	Caffeine	-1.62	2.169	0.451	0.95
Meconium	PWY.5097	Information	Pathway	-178.965	339.946		
Meconium	PWY.5097	Information	Interaction	26.581	38.48		
Meconium	PWY.5097	QTAC	Caffeine	-9.182	7.652	0.233	0.95
Meconium	PWY.5097	QTAC	Pathway	-1228.622	1199.422		
Meconium	PWY.5097	QTAC	Interaction	149.067	135.768		
Meconium	PWY.5097	Vocabulary	Caffeine	-1.04	3.31	0.767	0.95
Meconium	PWY.5097	Vocabulary	Pathway	-133.615	518.897		
Meconium	PWY.5097	Vocabulary	Interaction	15.96	58.736		
Meconium	PWY.5097	WISC sum	Caffeine	0.126	7.306	0.887	0.95
Meconium	PWY.5097	WISC sum	Pathway	478.687	1145.119		
Meconium	PWY.5097	WISC sum	Interaction	-16.825	129.622		
Meconium	PWY.5103	Block Design	Caffeine	2.779	1.912	0.083	0.95
Meconium	PWY.5103	Block Design	Pathway	352.531	253.923		
Meconium	PWY.5103	Block Design	Interaction	-49.308	30.657		
Meconium	PWY.5103	Coding	Caffeine	0.406	1.75	0.853	0.95
Meconium	PWY.5103	Coding	Pathway	3.304	232.498		
Meconium	PWY.5103	Coding	Interaction	-4.743	28.07		
Meconium	PWY.5103	Digit span	Caffeine	-1.867	1.454	0.245	0.95
Meconium	PWY.5103	Digit span	Pathway	-188.888	193.089		
Meconium	PWY.5103	Digit span	Interaction	24.98	23.312		
Meconium	PWY.5103	Information	Caffeine	0.794	1.461	0.503	0.95
Meconium	PWY.5103	Information	Pathway	50.92	194.043		
Meconium	PWY.5103	Information	Interaction	-14.396	23.427		
Meconium	PWY.5103	QTAC	Caffeine	-9.851	5.129	0.057	0.95
Meconium	PWY.5103	QTAC	Pathway	-1155.736	681.223		
Meconium	PWY.5103	QTAC	Interaction	146.029	82.245		
Meconium	PWY.5103	Vocabulary	Caffeine	-0.445	2.175	0.862	0.95
Meconium	PWY.5103	Vocabulary	Pathway	-174.057	288.856		
Meconium	PWY.5103	Vocabulary	Interaction	5.531	34.874		
Meconium	PWY.5103	WISC sum	Caffeine	1.666	4.934	0.601	0.95
Meconium	PWY.5103	WISC sum	Pathway	43.81	655.426		
Meconium	PWY.5103	WISC sum	Interaction	-37.936	79.131		
Meconium	PWY.5686	Block Design	Caffeine	4.119	3.212	0.138	0.95
Meconium	PWY.5686	Block Design	Pathway	530.118	422.313		

Exposure Window	Pathway	Outcome	Variable	Effect Estimate[a]	Std. Error	LRT[b]	
						p-value	q-value
Meconium	PWY.5686	Block Design	Interaction	-66.426	48.381		
Meconium	PWY.5686	Coding	Caffeine	-2.81	2.883	0.272	0.95
Meconium	PWY.5686	Coding	Pathway	-357.193	379.049		
Meconium	PWY.5686	Coding	Interaction	43.887	43.424		
Meconium	PWY.5686	Digit span	Caffeine	-2.484	2.359	0.322	0.95
Meconium	PWY.5686	Digit span	Pathway	-179.297	310.152		
Meconium	PWY.5686	Digit span	Interaction	32.354	35.531		
Meconium	PWY.5686	Information	Caffeine	-1.332	2.487	0.593	0.95
Meconium	PWY.5686	Information	Pathway	-165.948	327.028		
Meconium	PWY.5686	Information	Interaction	18.361	37.465		
Meconium	PWY.5686	QTAC	Caffeine	-11.055	8.671	0.198	0.95
Meconium	PWY.5686	QTAC	Pathway	-1413.316	1140.163		
Meconium	PWY.5686	QTAC	Interaction	155.248	130.618		
Meconium	PWY.5686	Vocabulary	Caffeine	-0.31	3.721	0.954	0.966
Meconium	PWY.5686	Vocabulary	Pathway	-126.522	489.339		
Meconium	PWY.5686	Vocabulary	Interaction	2.93	56.059		
Meconium	PWY.5686	WISC sum	Caffeine	-2.817	8.487	0.79	0.95
Meconium	PWY.5686	WISC sum	Pathway	-298.841	1116.05		
Meconium	PWY.5686	WISC sum	Interaction	31.105	127.856		
Meconium	PWY.6121	Block Design	Caffeine	1.429	2.389	0.435	0.95
Meconium	PWY.6121	Block Design	Pathway	203.925	342.682		
Meconium	PWY.6121	Block Design	Interaction	-28.974	40.444		
Meconium	PWY.6121	Coding	Caffeine	0.433	2.138	0.864	0.95
Meconium	PWY.6121	Coding	Pathway	54.239	306.75		
Meconium	PWY.6121	Coding	Interaction	-5.682	36.203		
Meconium	PWY.6121	Digit span	Caffeine	-1.32	1.69	0.534	0.95
Meconium	PWY.6121	Digit span	Pathway	-26.914	242.535		
Meconium	PWY.6121	Digit span	Interaction	16.3	28.625		
Meconium	PWY.6121	Information	Caffeine	-1.462	1.8	0.42	0.95
Meconium	PWY.6121	Information	Pathway	-142.112	258.218		
Meconium	PWY.6121	Information	Interaction	22.532	30.475		
Meconium	PWY.6121	QTAC	Caffeine	-7.724	6.377	0.235	0.95
Meconium	PWY.6121	QTAC	Pathway	-1007.288	914.848		
Meconium	PWY.6121	QTAC	Interaction	118.019	107.973		
Meconium	PWY.6121	Vocabulary	Caffeine	-1.713	2.746	0.534	0.95
Meconium	PWY.6121	Vocabulary	Pathway	-194.668	393.923		

Exposure Window	Pathway	Outcome	Variable	Effect Estimate[a]	Std. Error	LRT[b]	
						p-value	q-value
Meconium	PWY.6121	Vocabulary	Interaction	26.527	46.492		
Meconium	PWY.6121	WISC sum	Caffeine	-2.634	6.166	0.748	0.95
Meconium	PWY.6121	WISC sum	Pathway	-105.531	884.566		
Meconium	PWY.6121	WISC sum	Interaction	30.703	104.399		
Meconium	PWY.6122	Block Design	Caffeine	2.575	2.723	0.254	0.95
Meconium	PWY.6122	Block Design	Pathway	345.655	372.778		
Meconium	PWY.6122	Block Design	Interaction	-45.316	43.162		
Meconium	PWY.6122	Coding	Caffeine	-0.354	2.454	0.839	0.95
Meconium	PWY.6122	Coding	Pathway	-56.622	335.989		
Meconium	PWY.6122	Coding	Interaction	7.217	38.902		
Meconium	PWY.6122	Digit span	Caffeine	-1.56	1.915	0.497	0.95
Meconium	PWY.6122	Digit span	Pathway	-33.064	262.265		
Meconium	PWY.6122	Digit span	Interaction	18.909	30.366		
Meconium	PWY.6122	Information	Caffeine	0.232	2.086	0.847	0.95
Meconium	PWY.6122	Information	Pathway	86.637	285.579		
Meconium	PWY.6122	Information	Interaction	-5.826	33.066		
Meconium	PWY.6122	QTAC	Caffeine	-15.029	7.082	0.032	0.95
Meconium	PWY.6122	QTAC	Pathway	-1882.52	969.68		
Meconium	PWY.6122	QTAC	Interaction	226.157	112.274		
Meconium	PWY.6122	Vocabulary	Caffeine	-0.771	3.149	0.833	0.95
Meconium	PWY.6122	Vocabulary	Pathway	-24.268	431.191		
Meconium	PWY.6122	Vocabulary	Interaction	9.606	49.925		
Meconium	PWY.6122	WISC sum	Caffeine	0.122	7.059	0.88	0.95
Meconium	PWY.6122	WISC sum	Pathway	318.337	966.527		
Meconium	PWY.6122	WISC sum	Interaction	-15.409	111.909		
Meconium	PWY.6151	Block Design	Caffeine	3.039	2.167	0.097	0.95
Meconium	PWY.6151	Block Design	Pathway	456.807	316.105		
Meconium	PWY.6151	Block Design	Interaction	-56.706	36.778		
Meconium	PWY.6151	Coding	Caffeine	0.259	1.971	0.927	0.95
Meconium	PWY.6151	Coding	Pathway	58.027	287.531		
Meconium	PWY.6151	Coding	Interaction	-2.797	33.453		
Meconium	PWY.6151	Digit span	Caffeine	-2.08	1.627	0.239	0.95
Meconium	PWY.6151	Digit span	Pathway	-218.066	237.283		
Meconium	PWY.6151	Digit span	Interaction	29.972	27.607		
Meconium	PWY.6151	Information	Caffeine	-0.742	1.671	0.678	0.95
Meconium	PWY.6151	Information	Pathway	-137.005	243.747		

Exposure Window	Pathway	Outcome	Variable	Effect Estimate[a]	Std. Error	LRT[b]	
						p-value	q-value
Meconium	PWY.6151	Information	Interaction	10.794	28.359		
Meconium	PWY.6151	QTAC	Caffeine	-8.806	5.855	0.137	0.95
Meconium	PWY.6151	QTAC	Pathway	-1133.978	853.948		
Meconium	PWY.6151	QTAC	Interaction	136.548	99.355		
Meconium	PWY.6151	Vocabulary	Caffeine	0.601	2.541	0.751	0.95
Meconium	PWY.6151	Vocabulary	Pathway	60.865	370.634		
Meconium	PWY.6151	Vocabulary	Interaction	-12.526	43.122		
Meconium	PWY.6151	WISC sum	Caffeine	1.077	5.748	0.726	0.95
Meconium	PWY.6151	WISC sum	Pathway	220.628	838.419		
Meconium	PWY.6151	WISC sum	Interaction	-31.264	97.548		
Meconium	PWY.6163	Block Design	Caffeine	2.408	2.263	0.197	0.95
Meconium	PWY.6163	Block Design	Pathway	303.173	288.265		
Meconium	PWY.6163	Block Design	Interaction	-40.256	33.826		
Meconium	PWY.6163	Coding	Caffeine	0.349	2.033	0.883	0.95
Meconium	PWY.6163	Coding	Pathway	76.575	258.983		
Meconium	PWY.6163	Coding	Interaction	-4.086	30.39		
Meconium	PWY.6163	Digit span	Caffeine	-2.223	1.653	0.217	0.95
Meconium	PWY.6163	Digit span	Pathway	-172.919	210.65		
Meconium	PWY.6163	Digit span	Interaction	28.117	24.718		
Meconium	PWY.6163	Information	Caffeine	-1.922	1.721	0.25	0.95
Meconium	PWY.6163	Information	Pathway	-250.936	219.282		
Meconium	PWY.6163	Information	Interaction	27.289	25.731		
Meconium	PWY.6163	QTAC	Caffeine	-6.711	6.113	0.294	0.95
Meconium	PWY.6163	QTAC	Pathway	-664.408	778.859		
Meconium	PWY.6163	QTAC	Interaction	88.23	91.394		
Meconium	PWY.6163	Vocabulary	Caffeine	-1.485	2.632	0.573	0.95
Meconium	PWY.6163	Vocabulary	Pathway	-187.609	335.348		
Meconium	PWY.6163	Vocabulary	Interaction	20.326	39.351		
Meconium	PWY.6163	WISC sum	Caffeine	-2.872	5.948	0.7	0.95
Meconium	PWY.6163	WISC sum	Pathway	-231.716	757.776		
Meconium	PWY.6163	WISC sum	Interaction	31.39	88.92		
Meconium	PWY.6277	Block Design	Caffeine	2.575	2.723	0.254	0.95
Meconium	PWY.6277	Block Design	Pathway	345.655	372.778		
Meconium	PWY.6277	Block Design	Interaction	-45.316	43.162		
Meconium	PWY.6277	Coding	Caffeine	-0.354	2.454	0.839	0.95
Meconium	PWY.6277	Coding	Pathway	-56.622	335.989		

Exposure Window	Pathway	Outcome	Variable	Effect Estimate[a]	Std. Error	LRT[b]	
						p-value	q-value
Meconium	PWY.6277	Coding	Interaction	7.217	38.902		
Meconium	PWY.6277	Digit span	Caffeine	-1.56	1.915	0.497	0.95
Meconium	PWY.6277	Digit span	Pathway	-33.064	262.265		
Meconium	PWY.6277	Digit span	Interaction	18.909	30.366		
Meconium	PWY.6277	Information	Caffeine	0.232	2.086	0.847	0.95
Meconium	PWY.6277	Information	Pathway	86.637	285.579		
Meconium	PWY.6277	Information	Interaction	-5.826	33.066		
Meconium	PWY.6277	QTAC	Caffeine	-15.029	7.082	0.032	0.95
Meconium	PWY.6277	QTAC	Pathway	-1882.52	969.68		
Meconium	PWY.6277	QTAC	Interaction	226.157	112.274		
Meconium	PWY.6277	Vocabulary	Caffeine	-0.771	3.149	0.833	0.95
Meconium	PWY.6277	Vocabulary	Pathway	-24.268	431.191		
Meconium	PWY.6277	Vocabulary	Interaction	9.606	49.925		
Meconium	PWY.6277	WISC sum	Caffeine	0.122	7.059	0.88	0.95
Meconium	PWY.6277	WISC sum	Pathway	318.337	966.527		
Meconium	PWY.6277	WISC sum	Interaction	-15.409	111.909		
Meconium	PWY.6385	Block Design	Caffeine	0.972	3.492	0.693	0.95
Meconium	PWY.6385	Block Design	Pathway	167.226	509.374		
Meconium	PWY.6385	Block Design	Interaction	-21.152	58.508		
Meconium	PWY.6385	Coding	Caffeine	2.124	3.041	0.463	0.95
Meconium	PWY.6385	Coding	Pathway	392.1	443.484		
Meconium	PWY.6385	Coding	Interaction	-34.29	50.939		
Meconium	PWY.6385	Digit span	Caffeine	-2.392	2.488	0.369	0.95
Meconium	PWY.6385	Digit span	Pathway	-164.676	362.833		
Meconium	PWY.6385	Digit span	Interaction	34.423	41.676		
Meconium	PWY.6385	Information	Caffeine	-4.764	2.527	0.049	0.95
Meconium	PWY.6385	Information	Pathway	-598.201	368.506		
Meconium	PWY.6385	Information	Interaction	77.764	42.327		
Meconium	PWY.6385	QTAC	Caffeine	-7.165	9.274	0.456	0.95
Meconium	PWY.6385	QTAC	Pathway	-706.02	1352.669		
Meconium	PWY.6385	QTAC	Interaction	106.225	155.37		
Meconium	PWY.6385	Vocabulary	Caffeine	-4.028	3.957	0.285	0.95
Meconium	PWY.6385	Vocabulary	Pathway	-570.604	577.176		
Meconium	PWY.6385	Vocabulary	Interaction	65.274	66.296		
Meconium	PWY.6385	WISC sum	Caffeine	-8.088	8.843	0.37	0.95
Meconium	PWY.6385	WISC sum	Pathway	-774.155	1289.754		

Exposure Window	Pathway	Outcome	Variable	Effect Estimate[a]	Std. Error	LRT[b]	
						p-value	q-value
Meconium	PWY.6385	WISC sum	Interaction	122.019	148.144		
Meconium	PWY.6386	Block Design	Caffeine	1.685	3.781	0.568	0.95
Meconium	PWY.6386	Block Design	Pathway	235.46	479.72		
Meconium	PWY.6386	Block Design	Interaction	-29.164	55.786		
Meconium	PWY.6386	Coding	Caffeine	1.247	3.333	0.703	0.95
Meconium	PWY.6386	Coding	Pathway	214.587	422.86		
Meconium	PWY.6386	Coding	Interaction	-17.182	49.174		
Meconium	PWY.6386	Digit span	Caffeine	-1.441	2.698	0.657	0.95
Meconium	PWY.6386	Digit span	Pathway	-5.971	342.297		
Meconium	PWY.6386	Digit span	Interaction	16.17	39.805		
Meconium	PWY.6386	Information	Caffeine	-1.542	2.861	0.588	0.95
Meconium	PWY.6386	Information	Pathway	-147.075	363.02		
Meconium	PWY.6386	Information	Interaction	20.937	42.215		
Meconium	PWY.6386	QTAC	Caffeine	-16.754	9.812	0.08	0.95
Meconium	PWY.6386	QTAC	Pathway	-1807.247	1244.928		
Meconium	PWY.6386	QTAC	Interaction	235.224	144.772		
Meconium	PWY.6386	Vocabulary	Caffeine	-2.443	4.326	0.562	0.95
Meconium	PWY.6386	Vocabulary	Pathway	-265.976	548.862		
Meconium	PWY.6386	Vocabulary	Interaction	33.934	63.827		
Meconium	PWY.6386	WISC sum	Caffeine	-2.494	9.676	0.85	0.95
Meconium	PWY.6386	WISC sum	Pathway	31.026	1227.616		
Meconium	PWY.6386	WISC sum	Interaction	24.695	142.758		
Meconium	PWY.6387	Block Design	Caffeine	1.785	4.016	0.573	0.95
Meconium	PWY.6387	Block Design	Pathway	239.812	524.052		
Meconium	PWY.6387	Block Design	Interaction	-31.643	61.28		
Meconium	PWY.6387	Coding	Caffeine	1.341	3.546	0.699	0.95
Meconium	PWY.6387	Coding	Pathway	231.221	462.681		
Meconium	PWY.6387	Coding	Interaction	-19.132	54.104		
Meconium	PWY.6387	Digit span	Caffeine	-1.647	2.887	0.62	0.95
Meconium	PWY.6387	Digit span	Pathway	-37.552	376.716		
Meconium	PWY.6387	Digit span	Interaction	19.986	44.051		
Meconium	PWY.6387	Information	Caffeine	-1.878	3.042	0.528	0.95
Meconium	PWY.6387	Information	Pathway	-211.848	397.002		
Meconium	PWY.6387	Information	Interaction	26.854	46.424		
Meconium	PWY.6387	QTAC	Caffeine	-16.763	10.465	0.1	0.95
Meconium	PWY.6387	QTAC	Pathway	-1850.103	1365.667		

Exposure Window	Pathway	Outcome	Variable	Effect Estimate[a]	Std. Error	LRT[b]	
						p-value	q-value
Meconium	PWY.6387	QTAC	Interaction	243.482	159.695		
Meconium	PWY.6387	Vocabulary	Caffeine	-2.893	4.593		
Meconium	PWY.6387	Vocabulary	Pathway	-333.419	599.402	0.514	0.95
Meconium	PWY.6387	Vocabulary	Interaction	41.98	70.091		
Meconium	PWY.6387	WISC sum	Caffeine	-3.292	10.323		
Meconium	PWY.6387	WISC sum	Pathway	-111.787	1347.116	0.792	0.95
Meconium	PWY.6387	WISC sum	Interaction	38.046	157.525		
Meconium	PWY.6609	Block Design	Caffeine	-0.458	1.678		
Meconium	PWY.6609	Block Design	Pathway	-99.447	264.252	0.9	0.95
Meconium	PWY.6609	Block Design	Interaction	3.342	29.109		
Meconium	PWY.6609	Coding	Caffeine	1.038	1.501		
Meconium	PWY.6609	Coding	Pathway	173.151	236.367	0.488	0.95
Meconium	PWY.6609	Coding	Interaction	-16.552	26.038		
Meconium	PWY.6609	Digit span	Caffeine	0.059	1.26		
Meconium	PWY.6609	Digit span	Pathway	88.064	198.532	0.737	0.95
Meconium	PWY.6609	Digit span	Interaction	-6.731	21.87		
Meconium	PWY.6609	Information	Caffeine	-1.112	1.282		
Meconium	PWY.6609	Information	Pathway	-143.283	201.942	0.396	0.95
Meconium	PWY.6609	Information	Interaction	17.32	22.245		
Meconium	PWY.6609	QTAC	Caffeine	-1.13	4.574		
Meconium	PWY.6609	QTAC	Pathway	-72.838	720.475	0.935	0.953
Meconium	PWY.6609	QTAC	Interaction	5.965	79.365		
Meconium	PWY.6609	Vocabulary	Caffeine	-0.301	1.947		
Meconium	PWY.6609	Vocabulary	Pathway	-57.709	306.6	0.924	0.95
Meconium	PWY.6609	Vocabulary	Interaction	2.962	33.774		
Meconium	PWY.6609	WISC sum	Caffeine	-0.772	4.398		
Meconium	PWY.6609	WISC sum	Pathway	-39.224	692.675	0.996	0.996
Meconium	PWY.6609	WISC sum	Interaction	0.34	76.303		
Meconium	PWY.6737	Block Design	Caffeine	1.072	2.05		
Meconium	PWY.6737	Block Design	Pathway	61.091	253.825	0.47	0.95
Meconium	PWY.6737	Block Design	Interaction	-19.18	28.962		
Meconium	PWY.6737	Coding	Caffeine	1.181	1.893		
Meconium	PWY.6737	Coding	Pathway	168.469	234.309	0.53	0.95
Meconium	PWY.6737	Coding	Interaction	-15.388	26.735		
Meconium	PWY.6737	Digit span	Caffeine	-1.207	1.58	0.538	0.95
Meconium	PWY.6737	Digit span	Pathway	-69.205	195.606		

Exposure Window	Pathway	Outcome	Variable	Effect Estimate[a]	Std. Error	LRT[b]	
						p-value	q-value
Meconium	PWY.6737	Digit span	Interaction	12.603	22.319		
Meconium	PWY.6737	Information	Caffeine	-0.625	1.623	0.73	0.95
Meconium	PWY.6737	Information	Pathway	-90.472	200.987		
Meconium	PWY.6737	Information	Interaction	7.24	22.933		
Meconium	PWY.6737	QTAC	Caffeine	1.802	5.671	0.613	0.95
Meconium	PWY.6737	QTAC	Pathway	494.627	702.131		
Meconium	PWY.6737	QTAC	Interaction	-37.165	80.114		
Meconium	PWY.6737	Vocabulary	Caffeine	0.34	2.456	0.831	0.95
Meconium	PWY.6737	Vocabulary	Pathway	20.225	304.124		
Meconium	PWY.6737	Vocabulary	Interaction	-6.792	34.701		
Meconium	PWY.6737	WISC sum	Caffeine	0.761	5.531	0.763	0.95
Meconium	PWY.6737	WISC sum	Pathway	90.108	684.797		
Meconium	PWY.6737	WISC sum	Interaction	-21.517	78.136		
Meconium	PWY.7111	Block Design	Caffeine	3.674	2.564	0.095	0.95
Meconium	PWY.7111	Block Design	Pathway	421.374	294.778		
Meconium	PWY.7111	Block Design	Interaction	-53.197	34.334		
Meconium	PWY.7111	Coding	Caffeine	1.318	2.339	0.57	0.95
Meconium	PWY.7111	Coding	Pathway	127.952	268.883		
Meconium	PWY.7111	Coding	Interaction	-16.319	31.318		
Meconium	PWY.7111	Digit span	Caffeine	-2.839	1.865	0.144	0.95
Meconium	PWY.7111	Digit span	Pathway	-220.428	214.415		
Meconium	PWY.7111	Digit span	Interaction	33.749	24.974		
Meconium	PWY.7111	Information	Caffeine	-2.714	1.885	0.133	0.95
Meconium	PWY.7111	Information	Pathway	-368.547	216.739		
Meconium	PWY.7111	Information	Interaction	35.052	25.244		
Meconium	PWY.7111	QTAC	Caffeine	-8.213	6.951	0.246	0.95
Meconium	PWY.7111	QTAC	Pathway	-722.523	799.063		
Meconium	PWY.7111	QTAC	Interaction	99.369	93.07		
Meconium	PWY.7111	Vocabulary	Caffeine	-2.868	2.968	0.314	0.95
Meconium	PWY.7111	Vocabulary	Pathway	-367.397	341.158		
Meconium	PWY.7111	Vocabulary	Interaction	36.761	39.736		
Meconium	PWY.7111	WISC sum	Caffeine	-3.429	6.773	0.664	0.95
Meconium	PWY.7111	WISC sum	Pathway	-407.045	778.662		
Meconium	PWY.7111	WISC sum	Interaction	36.046	90.694		
Meconium	PWY.7219	Block Design	Caffeine	1.445	2.441	0.44	0.95
Meconium	PWY.7219	Block Design	Pathway	179.151	315.31		

Exposure Window	Pathway	Outcome	Variable	Effect Estimate[a]	Std. Error	LRT[b]	
						p-value	q-value
Meconium	PWY.7219	Block Design	Interaction	-26.584	37.496		
Meconium	PWY.7219	Coding	Caffeine	-0.371	2.163	0.817	0.95
Meconium	PWY.7219	Coding	Pathway	-2.153	279.417		
Meconium	PWY.7219	Coding	Interaction	7.037	33.228		
Meconium	PWY.7219	Digit span	Caffeine	-0.817	1.738	0.766	0.95
Meconium	PWY.7219	Digit span	Pathway	41.22	224.524		
Meconium	PWY.7219	Digit span	Interaction	7.27	26.7		
Meconium	PWY.7219	Information	Caffeine	-1.611	1.854	0.38	0.95
Meconium	PWY.7219	Information	Pathway	-181.646	239.5		
Meconium	PWY.7219	Information	Interaction	22.981	28.481		
Meconium	PWY.7219	QTAC	Caffeine	-8.372	6.51	0.205	0.95
Meconium	PWY.7219	QTAC	Pathway	-972.333	840.962		
Meconium	PWY.7219	QTAC	Interaction	116.899	100.005		
Meconium	PWY.7219	Vocabulary	Caffeine	-2.204	2.793	0.416	0.95
Meconium	PWY.7219	Vocabulary	Pathway	-307.19	360.725		
Meconium	PWY.7219	Vocabulary	Interaction	31.998	42.897		
Meconium	PWY.7219	WISC sum	Caffeine	-3.558	6.334	0.632	0.95
Meconium	PWY.7219	WISC sum	Pathway	-270.619	818.137		
Meconium	PWY.7219	WISC sum	Interaction	42.701	97.291		
Meconium	PWY.7221	Block Design	Caffeine	3.632	2.638	0.107	0.95
Meconium	PWY.7221	Block Design	Pathway	507.93	350.388		
Meconium	PWY.7221	Block Design	Interaction	-59.878	40.124		
Meconium	PWY.7221	Coding	Caffeine	-0.301	2.398	0.849	0.95
Meconium	PWY.7221	Coding	Pathway	-81.939	318.539		
Meconium	PWY.7221	Coding	Interaction	6.344	36.477		
Meconium	PWY.7221	Digit span	Caffeine	-1.348	1.962	0.579	0.95
Meconium	PWY.7221	Digit span	Pathway	-38.452	260.612		
Meconium	PWY.7221	Digit span	Interaction	15.161	29.843		
Meconium	PWY.7221	Information	Caffeine	0.219	2.051	0.86	0.95
Meconium	PWY.7221	Information	Pathway	26.209	272.39		
Meconium	PWY.7221	Information	Interaction	-5.041	31.192		
Meconium	PWY.7221	QTAC	Caffeine	-14.396	6.879	0.033	0.95
Meconium	PWY.7221	QTAC	Pathway	-1966.328	913.86		
Meconium	PWY.7221	QTAC	Interaction	208.926	104.649		
Meconium	PWY.7221	Vocabulary	Caffeine	-1.392	3.094	0.659	0.95
Meconium	PWY.7221	Vocabulary	Pathway	-153.416	410.945		

Exposure Window	Pathway	Outcome	Variable	Effect Estimate[a]	Std. Error	LRT[b]	
						p-value	q-value
Meconium	PWY.7221	Vocabulary	Interaction	19.026	47.058		
Meconium	PWY.7221	WISC sum	Caffeine	0.809	6.981	0.802	0.95
Meconium	PWY.7221	WISC sum	Pathway	260.332	927.324		
Meconium	PWY.7221	WISC sum	Interaction	-24.389	106.191		
Meconium	PWY.7400	Block Design	Caffeine	2.225	2.503	0.275	0.95
Meconium	PWY.7400	Block Design	Pathway	292.374	326.186		
Meconium	PWY.7400	Block Design	Interaction	-37.978	37.784		
Meconium	PWY.7400	Coding	Caffeine	-0.548	2.242	0.755	0.95
Meconium	PWY.7400	Coding	Pathway	-48.236	292.112		
Meconium	PWY.7400	Coding	Interaction	9.681	33.837		
Meconium	PWY.7400	Digit span	Caffeine	-2.249	1.777	0.243	0.95
Meconium	PWY.7400	Digit span	Pathway	-149.665	231.614		
Meconium	PWY.7400	Digit span	Interaction	28.824	26.829		
Meconium	PWY.7400	Information	Caffeine	-1.663	1.906	0.374	0.95
Meconium	PWY.7400	Information	Pathway	-216.721	248.42		
Meconium	PWY.7400	Information	Interaction	23.488	28.776		
Meconium	PWY.7400	QTAC	Caffeine	-11.913	6.559	0.068	0.95
Meconium	PWY.7400	QTAC	Pathway	-1314.176	854.724		
Meconium	PWY.7400	QTAC	Interaction	167.915	99.008		
Meconium	PWY.7400	Vocabulary	Caffeine	-1.884	2.893	0.511	0.95
Meconium	PWY.7400	Vocabulary	Pathway	-211.326	377.021		
Meconium	PWY.7400	Vocabulary	Interaction	26.334	43.673		
Meconium	PWY.7400	WISC sum	Caffeine	-4.119	6.51	0.576	0.95
Meconium	PWY.7400	WISC sum	Pathway	-333.574	848.38		
Meconium	PWY.7400	WISC sum	Interaction	50.349	98.273		
Meconium	TRNA.CHARGING.PWY	Block Design	Caffeine	0.915	3.974	0.741	0.95
Meconium	TRNA.CHARGING.PWY	Block Design	Pathway	115.397	569.088		
Meconium	TRNA.CHARGING.PWY	Block Design	Interaction	-19.673	65.057		
Meconium	TRNA.CHARGING.PWY	Coding	Caffeine	-0.227	3.475	0.921	0.95
Meconium	TRNA.CHARGING.PWY	Coding	Pathway	71.274	497.655		
Meconium	TRNA.CHARGING.PWY	Coding	Interaction	5.19	56.891		
Meconium	TRNA.CHARGING.PWY	Digit span	Caffeine	-1.839	2.857	0.564	0.95
Meconium	TRNA.CHARGING.PWY	Digit span	Pathway	-80.967	409.222		
Meconium	TRNA.CHARGING.PWY	Digit span	Interaction	24.756	46.781		
Meconium	TRNA.CHARGING.PWY	Information	Caffeine	-1.677	2.997	0.571	0.95
Meconium	TRNA.CHARGING.PWY	Information	Pathway	-162.773	429.202		

Exposure Window	Pathway	Outcome	Variable	Effect Estimate[a]	Std. Error	LRT[b]	
						p-value	q-value
Meconium	TRNA.CHARGING.PWY	Information	Interaction	25.454	49.065		
Meconium	TRNA.CHARGING.PWY	QTAC	Caffeine	-14.588	10.393	0.151	0.95
Meconium	TRNA.CHARGING.PWY	QTAC	Pathway	-1696.417	1488.496		
Meconium	TRNA.CHARGING.PWY	QTAC	Interaction	225.741	170.162		
Meconium	TRNA.CHARGING.PWY	Vocabulary	Caffeine	-0.885	4.552	0.859	0.95
Meconium	TRNA.CHARGING.PWY	Vocabulary	Pathway	-44.082	651.982		
Meconium	TRNA.CHARGING.PWY	Vocabulary	Interaction	12.082	74.533		
Meconium	TRNA.CHARGING.PWY	WISC sum	Caffeine	-3.714	10.13	0.753	0.95
Meconium	TRNA.CHARGING.PWY	WISC sum	Pathway	-101.152	1450.777		
Meconium	TRNA.CHARGING.PWY	WISC sum	Interaction	47.808	165.85		
Meconium	UNINTEGRATED	Block Design	Caffeine	10.007	7.751	0.151	0.95
Meconium	UNINTEGRATED	Block Design	Pathway	1.265	0.912		
Meconium	UNINTEGRATED	Block Design	Interaction	-0.144	0.108		
Meconium	UNINTEGRATED	Coding	Caffeine	4.629	6.978	0.479	0.95
Meconium	UNINTEGRATED	Coding	Pathway	0.43	0.821		
Meconium	UNINTEGRATED	Coding	Interaction	-0.063	0.097		
Meconium	UNINTEGRATED	Digit span	Caffeine	-1.804	5.883	0.782	0.95
Meconium	UNINTEGRATED	Digit span	Pathway	-0.113	0.692		
Meconium	UNINTEGRATED	Digit span	Interaction	0.021	0.082		
Meconium	UNINTEGRATED	Information	Caffeine	-6.357	5.844	0.246	0.95
Meconium	UNINTEGRATED	Information	Pathway	-0.881	0.687		
Meconium	UNINTEGRATED	Information	Interaction	0.087	0.081		
Meconium	UNINTEGRATED	QTAC	Caffeine	26.64	20.82	0.154	0.95
Meconium	UNINTEGRATED	QTAC	Pathway	2.973	2.449		
Meconium	UNINTEGRATED	QTAC	Interaction	-0.383	0.29		
Meconium	UNINTEGRATED	Vocabulary	Caffeine	0.892	9.078	0.901	0.95
Meconium	UNINTEGRATED	Vocabulary	Pathway	0.103	1.068		
Meconium	UNINTEGRATED	Vocabulary	Interaction	-0.014	0.127		
Meconium	UNINTEGRATED	WISC sum	Caffeine	7.367	20.418	0.664	0.95
Meconium	UNINTEGRATED	WISC sum	Pathway	0.805	2.402		
Meconium	UNINTEGRATED	WISC sum	Interaction	-0.113	0.285		
Meconium	UNMAPPED	Block Design	Caffeine	-3.561	2.313	0.125	0.95
Meconium	UNMAPPED	Block Design	Pathway	-1.275	0.86		
Meconium	UNMAPPED	Block Design	Interaction	0.144	0.101		
Meconium	UNMAPPED	Coding	Caffeine	-1.111	2.093	0.525	0.95
Meconium	UNMAPPED	Coding	Pathway	-0.369	0.778		

Exposure Window	Pathway	Outcome	Variable	Effect Estimate[a]	Std. Error	LRT[b]	
						p-value	q-value
Meconium	UNMAPPED	Coding	Interaction	0.053	0.092		
Meconium	UNMAPPED	Digit span	Caffeine	0.346	1.759	0.679	0.95
Meconium	UNMAPPED	Digit span	Pathway	0.181	0.654		
Meconium	UNMAPPED	Digit span	Interaction	-0.029	0.077		
Meconium	UNMAPPED	Information	Caffeine	1.541	1.754	0.303	0.95
Meconium	UNMAPPED	Information	Pathway	0.757	0.652		
Meconium	UNMAPPED	Information	Interaction	-0.073	0.077		
Meconium	UNMAPPED	QTAC	Caffeine	-7.182	6.289	0.268	0.95
Meconium	UNMAPPED	QTAC	Pathway	-2.22	2.339		
Meconium	UNMAPPED	QTAC	Interaction	0.281	0.275		
Meconium	UNMAPPED	Vocabulary	Caffeine	-0.418	2.717	0.911	0.95
Meconium	UNMAPPED	Vocabulary	Pathway	-0.075	1.01		
Meconium	UNMAPPED	Vocabulary	Interaction	0.012	0.119		
Meconium	UNMAPPED	WISC sum	Caffeine	-3.202	6.113	0.662	0.95
Meconium	UNMAPPED	WISC sum	Pathway	-0.78	2.273		
Meconium	UNMAPPED	WISC sum	Interaction	0.107	0.268		
Meconium	VALSYN.PWY	Block Design	Caffeine	3.986	2.602	0.076	0.95
Meconium	VALSYN.PWY	Block Design	Pathway	465.085	299.929		
Meconium	VALSYN.PWY	Block Design	Interaction	-57.311	34.783		
Meconium	VALSYN.PWY	Coding	Caffeine	0.876	2.383	0.722	0.95
Meconium	VALSYN.PWY	Coding	Pathway	74.897	274.635		
Meconium	VALSYN.PWY	Coding	Interaction	-10.365	31.85		
Meconium	VALSYN.PWY	Digit span	Caffeine	-2.911	1.893	0.138	0.95
Meconium	VALSYN.PWY	Digit span	Pathway	-227.289	218.153		
Meconium	VALSYN.PWY	Digit span	Interaction	34.686	25.3		
Meconium	VALSYN.PWY	Information	Caffeine	-2.608	1.93	0.16	0.95
Meconium	VALSYN.PWY	Information	Pathway	-354.498	222.417		
Meconium	VALSYN.PWY	Information	Interaction	33.517	25.794		
Meconium	VALSYN.PWY	QTAC	Caffeine	-9.006	7.056	0.206	0.95
Meconium	VALSYN.PWY	QTAC	Pathway	-819.12	813.219		
Meconium	VALSYN.PWY	QTAC	Interaction	109.909	94.31		
Meconium	VALSYN.PWY	Vocabulary	Caffeine	-2.544	3.03	0.385	0.95
Meconium	VALSYN.PWY	Vocabulary	Pathway	-327.965	349.255		
Meconium	VALSYN.PWY	Vocabulary	Interaction	32.309	40.504		
Meconium	VALSYN.PWY	WISC sum	Caffeine	-3.201	6.902	0.697	0.95
Meconium	VALSYN.PWY	WISC sum	Pathway	-369.771	795.506		

Exposure Window	Pathway	Outcome	Variable	Effect Estimate[a]	Std. Error	LRT[b]	
						p-value	q-value
Meconium	VALSYN.PWY	WISC sum	Interaction	32.837	92.256		
Meconium Adj.[c]	ARGSYN.PWY	Block Design	Caffeine	2.267	2.537	0.267	0.923
Meconium Adj.[c]	ARGSYN.PWY	Block Design	Pathway	299.692	330.155		
Meconium Adj.[c]	ARGSYN.PWY	Block Design	Interaction	0.292	0.939		
Meconium Adj.[c]	ARGSYN.PWY	Coding	Caffeine	-0.466	2.256	0.786	0.974
Meconium Adj.[c]	ARGSYN.PWY	Coding	Pathway	-30.428	293.672		
Meconium Adj.[c]	ARGSYN.PWY	Coding	Interaction	0.672	0.835		
Meconium Adj.[c]	ARGSYN.PWY	Digit span	Caffeine	-2.253	1.803	0.243	0.923
Meconium Adj.[c]	ARGSYN.PWY	Digit span	Pathway	-149.264	234.641		
Meconium Adj.[c]	ARGSYN.PWY	Digit span	Interaction	0.016	0.667		
Meconium Adj.[c]	ARGSYN.PWY	Information	Caffeine	-1.528	1.893	0.411	0.974
Meconium Adj.[c]	ARGSYN.PWY	Information	Pathway	-188.098	246.372		
Meconium Adj.[c]	ARGSYN.PWY	Information	Interaction	0.934	0.7		
Meconium Adj.[c]	ARGSYN.PWY	QTAC	Caffeine	-12.576	6.347	0.043	0.868
Meconium Adj.[c]	ARGSYN.PWY	QTAC	Pathway	-1446.852	826.053		
Meconium Adj.[c]	ARGSYN.PWY	QTAC	Interaction	-4.688	2.348		
Meconium Adj.[c]	ARGSYN.PWY	Vocabulary	Caffeine	-1.704	2.9	0.554	0.974
Meconium Adj.[c]	ARGSYN.PWY	Vocabulary	Pathway	-175.826	377.364		
Meconium Adj.[c]	ARGSYN.PWY	Vocabulary	Interaction	1.083	1.073		
Meconium Adj.[c]	ARGSYN.PWY	WISC sum	Caffeine	-3.684	6.481	0.624	0.974
Meconium Adj.[c]	ARGSYN.PWY	WISC sum	Pathway	-243.924	843.472		
Meconium Adj.[c]	ARGSYN.PWY	WISC sum	Interaction	2.997	2.398		
Meconium Adj.[c]	ARGSYNBSUB.PWY	Block Design	Caffeine	2.321	2.461	0.243	0.923
Meconium Adj.[c]	ARGSYNBSUB.PWY	Block Design	Pathway	291.634	304.282		
Meconium Adj.[c]	ARGSYNBSUB.PWY	Block Design	Interaction	0.325	0.942		
Meconium Adj.[c]	ARGSYNBSUB.PWY	Coding	Caffeine	-0.294	2.195	0.848	0.974
Meconium Adj.[c]	ARGSYNBSUB.PWY	Coding	Pathway	-12.091	271.399		
Meconium Adj.[c]	ARGSYNBSUB.PWY	Coding	Interaction	0.669	0.84		
Meconium Adj.[c]	ARGSYNBSUB.PWY	Digit span	Caffeine	-2.485	1.749	0.179	0.922
Meconium Adj.[c]	ARGSYNBSUB.PWY	Digit span	Pathway	-173.119	216.263		
Meconium Adj.[c]	ARGSYNBSUB.PWY	Digit span	Interaction	-0.006	0.669		
Meconium Adj.[c]	ARGSYNBSUB.PWY	Information	Caffeine	-1.496	1.839	0.407	0.974
Meconium Adj.[c]	ARGSYNBSUB.PWY	Information	Pathway	-174.276	227.335		
Meconium Adj.[c]	ARGSYNBSUB.PWY	Information	Interaction	0.916	0.704		
Meconium Adj.[c]	ARGSYNBSUB.PWY	QTAC	Caffeine	-12.047	6.183	0.047	0.868
Meconium Adj.[c]	ARGSYNBSUB.PWY	QTAC	Pathway	-1346.99	764.384		

Exposure Window	Pathway	Outcome	Variable	Effect Estimate[a]	Std. Error	LRT[b]	
						p-value	q-value
Meconium Adj.[c]	ARGSYNBSUB.PWY	QTAC	Interaction	-4.903	2.365		
Meconium Adj.[c]	ARGSYNBSUB.PWY	Vocabulary	Caffeine	-1.656	2.818	0.555	0.974
Meconium Adj.[c]	ARGSYNBSUB.PWY	Vocabulary	Pathway	-166.135	348.385		
Meconium Adj.[c]	ARGSYNBSUB.PWY	Vocabulary	Interaction	1.057	1.078		
Meconium Adj.[c]	ARGSYNBSUB.PWY	WISC sum	Caffeine	-3.61	6.303	0.623	0.974
Meconium Adj.[c]	ARGSYNBSUB.PWY	WISC sum	Pathway	-233.987	779.238		
Meconium Adj.[c]	ARGSYNBSUB.PWY	WISC sum	Interaction	2.96	2.411		
Meconium Adj.[c]	ARO.PWY	Block Design	Caffeine	3.495	2.488	0.097	0.874
Meconium Adj.[c]	ARO.PWY	Block Design	Pathway	437.112	319.677		
Meconium Adj.[c]	ARO.PWY	Block Design	Interaction	0.433	0.924		
Meconium Adj.[c]	ARO.PWY	Coding	Caffeine	-0.396	2.247	0.816	0.974
Meconium Adj.[c]	ARO.PWY	Coding	Pathway	-10.104	288.7		
Meconium Adj.[c]	ARO.PWY	Coding	Interaction	0.641	0.835		
Meconium Adj.[c]	ARO.PWY	Digit span	Caffeine	-2.52	1.828	0.192	0.923
Meconium Adj.[c]	ARO.PWY	Digit span	Pathway	-197.786	234.945		
Meconium Adj.[c]	ARO.PWY	Digit span	Interaction	-0.147	0.679		
Meconium Adj.[c]	ARO.PWY	Information	Caffeine	-1.358	1.888	0.465	0.974
Meconium Adj.[c]	ARO.PWY	Information	Pathway	-177.488	242.658		
Meconium Adj.[c]	ARO.PWY	Information	Interaction	0.892	0.701		
Meconium Adj.[c]	ARO.PWY	QTAC	Caffeine	-9.971	6.447	0.117	0.922
Meconium Adj.[c]	ARO.PWY	QTAC	Pathway	-1182.798	828.448		
Meconium Adj.[c]	ARO.PWY	QTAC	Interaction	-5.053	2.395		
Meconium Adj.[c]	ARO.PWY	Vocabulary	Caffeine	-0.737	2.901	0.817	0.974
Meconium Adj.[c]	ARO.PWY	Vocabulary	Pathway	-102.389	372.759		
Meconium Adj.[c]	ARO.PWY	Vocabulary	Interaction	1.008	1.078		
Meconium Adj.[c]	ARO.PWY	WISC sum	Caffeine	-1.517	6.519	0.903	0.974
Meconium Adj.[c]	ARO.PWY	WISC sum	Pathway	-50.655	837.684		
Meconium Adj.[c]	ARO.PWY	WISC sum	Interaction	2.826	2.421		
Meconium Adj.[c]	BRANCHED.CHAIN.AA.SYN.PWY	Block Design	Caffeine	3.282	2.174	0.073	0.868
Meconium Adj.[c]	BRANCHED.CHAIN.AA.SYN.PWY	Block Design	Pathway	414.982	283.193		
Meconium Adj.[c]	BRANCHED.CHAIN.AA.SYN.PWY	Block Design	Interaction	0.349	0.913		
Meconium Adj.[c]	BRANCHED.CHAIN.AA.SYN.PWY	Coding	Caffeine	0.428	1.985	0.857	0.974
Meconium Adj.[c]	BRANCHED.CHAIN.AA.SYN.PWY	Coding	Pathway	15.759	258.661		
Meconium Adj.[c]	BRANCHED.CHAIN.AA.SYN.PWY	Coding	Interaction	0.581	0.834		
Meconium Adj.[c]	BRANCHED.CHAIN.AA.SYN.PWY	Digit span	Caffeine	-2.385	1.641	0.166	0.922
Meconium Adj.[c]	BRANCHED.CHAIN.AA.SYN.PWY	Digit span	Pathway	-238.639	213.849		

Exposure Window	Pathway	Outcome	Variable	Effect Estimate[a]	Std. Error	LRT[b]	
						p-value	q-value
Meconium Adj.[c]	BRANCHED.CHAIN.AA.SYN.PWY	Digit span	Interaction	-0.162	0.69		
Meconium Adj.[c]	BRANCHED.CHAIN.AA.SYN.PWY	Information	Caffeine	0.646	1.629	0.609	0.974
Meconium Adj.[c]	BRANCHED.CHAIN.AA.SYN.PWY	Information	Pathway	29.898	212.254		
Meconium Adj.[c]	BRANCHED.CHAIN.AA.SYN.PWY	Information	Interaction	0.916	0.684		
Meconium Adj.[c]	BRANCHED.CHAIN.AA.SYN.PWY	QTAC	Caffeine	-12.028	5.526	0.027	0.868
Meconium Adj.[c]	BRANCHED.CHAIN.AA.SYN.PWY	QTAC	Pathway	-1415.327	719.925		
Meconium Adj.[c]	BRANCHED.CHAIN.AA.SYN.PWY	QTAC	Interaction	-4.882	2.322		
Meconium Adj.[c]	BRANCHED.CHAIN.AA.SYN.PWY	Vocabulary	Caffeine	-0.571	2.468	0.837	0.974
Meconium Adj.[c]	BRANCHED.CHAIN.AA.SYN.PWY	Vocabulary	Pathway	-179.378	321.582		
Meconium Adj.[c]	BRANCHED.CHAIN.AA.SYN.PWY	Vocabulary	Interaction	0.901	1.037		
Meconium Adj.[c]	BRANCHED.CHAIN.AA.SYN.PWY	WISC sum	Caffeine	1.4	5.591	0.674	0.974
Meconium Adj.[c]	BRANCHED.CHAIN.AA.SYN.PWY	WISC sum	Pathway	42.622	728.348		
Meconium Adj.[c]	BRANCHED.CHAIN.AA.SYN.PWY	WISC sum	Interaction	2.584	2.349		
Meconium Adj.[c]	CALVIN.PWY	Block Design	Caffeine	1.485	1.595	0.218	0.923
Meconium Adj.[c]	CALVIN.PWY	Block Design	Pathway	257.991	245.989		
Meconium Adj.[c]	CALVIN.PWY	Block Design	Interaction	0.405	0.941		
Meconium Adj.[c]	CALVIN.PWY	Coding	Caffeine	0.766	1.426	0.602	0.974
Meconium Adj.[c]	CALVIN.PWY	Coding	Pathway	97.64	219.904		
Meconium Adj.[c]	CALVIN.PWY	Coding	Interaction	0.647	0.841		
Meconium Adj.[c]	CALVIN.PWY	Digit span	Caffeine	-1.321	1.169	0.35	0.974
Meconium Adj.[c]	CALVIN.PWY	Digit span	Pathway	-90.792	180.349		
Meconium Adj.[c]	CALVIN.PWY	Digit span	Interaction	-0.095	0.69		
Meconium Adj.[c]	CALVIN.PWY	Information	Caffeine	-0.181	1.2	0.958	0.978
Meconium Adj.[c]	CALVIN.PWY	Information	Pathway	-7.143	185.14		
Meconium Adj.[c]	CALVIN.PWY	Information	Interaction	0.977	0.708		
Meconium Adj.[c]	CALVIN.PWY	QTAC	Caffeine	-5.334	4.081	0.211	0.923
Meconium Adj.[c]	CALVIN.PWY	QTAC	Pathway	-802.448	629.458		
Meconium Adj.[c]	CALVIN.PWY	QTAC	Interaction	-5.114	2.407		
Meconium Adj.[c]	CALVIN.PWY	Vocabulary	Caffeine	0.008	1.834	0.925	0.978
Meconium Adj.[c]	CALVIN.PWY	Vocabulary	Pathway	18.784	282.819		
Meconium Adj.[c]	CALVIN.PWY	Vocabulary	Interaction	1.073	1.082		
Meconium Adj.[c]	CALVIN.PWY	WISC sum	Caffeine	0.758	4.105	0.673	0.974
Meconium Adj.[c]	CALVIN.PWY	WISC sum	Pathway	276.481	633.172		
Meconium Adj.[c]	CALVIN.PWY	WISC sum	Interaction	3.007	2.421		
Meconium Adj.[c]	COA.PWY.1	Block Design	Caffeine	4.124	4.725	0.303	0.974
Meconium Adj.[c]	COA.PWY.1	Block Design	Pathway	554.833	648.952		

Exposure Window	Pathway	Outcome	Variable	Effect Estimate[a]	Std. Error	LRT[b] p-value	LRT[b] q-value
Meconium Adj.[c]	COA.PWY.1	Block Design	Interaction	0.4	0.937		
Meconium Adj.[c]	COA.PWY.1	Coding	Caffeine	-1.693	4.191	0.637	0.974
Meconium Adj.[c]	COA.PWY.1	Coding	Pathway	-178.657	575.621		
Meconium Adj.[c]	COA.PWY.1	Coding	Interaction	0.563	0.831		
Meconium Adj.[c]	COA.PWY.1	Digit span	Caffeine	-4.276	3.4	0.201	0.923
Meconium Adj.[c]	COA.PWY.1	Digit span	Pathway	-408.111	466.96		
Meconium Adj.[c]	COA.PWY.1	Digit span	Interaction	-0.223	0.674		
Meconium Adj.[c]	COA.PWY.1	Information	Caffeine	-5.723	3.432	0.075	0.868
Meconium Adj.[c]	COA.PWY.1	Information	Pathway	-760.28	471.392		
Meconium Adj.[c]	COA.PWY.1	Information	Interaction	0.84	0.681		
Meconium Adj.[c]	COA.PWY.1	QTAC	Caffeine	-15.495	12.041	0.18	0.922
Meconium Adj.[c]	COA.PWY.1	QTAC	Pathway	-1817.671	1653.754		
Meconium Adj.[c]	COA.PWY.1	QTAC	Interaction	-4.925	2.388		
Meconium Adj.[c]	COA.PWY.1	Vocabulary	Caffeine	-0.528	5.391	0.937	0.978
Meconium Adj.[c]	COA.PWY.1	Vocabulary	Pathway	-128.149	740.46		
Meconium Adj.[c]	COA.PWY.1	Vocabulary	Interaction	1.056	1.069		
Meconium Adj.[c]	COA.PWY.1	WISC sum	Caffeine	-8.096	12.092	0.504	0.974
Meconium Adj.[c]	COA.PWY.1	WISC sum	Pathway	-920.363	1660.754		
Meconium Adj.[c]	COA.PWY.1	WISC sum	Interaction	2.636	2.398		
Meconium Adj.[c]	COMPLETE.ARO.PWY	Block Design	Caffeine	3.353	2.372	0.093	0.868
Meconium Adj.[c]	COMPLETE.ARO.PWY	Block Design	Pathway	464.039	320.428		
Meconium Adj.[c]	COMPLETE.ARO.PWY	Block Design	Interaction	0.507	0.931		
Meconium Adj.[c]	COMPLETE.ARO.PWY	Coding	Caffeine	0.124	2.149	0.985	0.988
Meconium Adj.[c]	COMPLETE.ARO.PWY	Coding	Pathway	37.058	290.226		
Meconium Adj.[c]	COMPLETE.ARO.PWY	Coding	Interaction	0.648	0.843		
Meconium Adj.[c]	COMPLETE.ARO.PWY	Digit span	Caffeine	-2.555	1.735	0.161	0.922
Meconium Adj.[c]	COMPLETE.ARO.PWY	Digit span	Pathway	-220.821	234.416		
Meconium Adj.[c]	COMPLETE.ARO.PWY	Digit span	Interaction	-0.182	0.681		
Meconium Adj.[c]	COMPLETE.ARO.PWY	Information	Caffeine	-0.855	1.805	0.651	0.974
Meconium Adj.[c]	COMPLETE.ARO.PWY	Information	Pathway	-110.628	243.841		
Meconium Adj.[c]	COMPLETE.ARO.PWY	Information	Interaction	0.913	0.708		
Meconium Adj.[c]	COMPLETE.ARO.PWY	QTAC	Caffeine	-10.462	6.077	0.08	0.868
Meconium Adj.[c]	COMPLETE.ARO.PWY	QTAC	Pathway	-1383.924	820.832		
Meconium Adj.[c]	COMPLETE.ARO.PWY	QTAC	Interaction	-5.332	2.385		
Meconium Adj.[c]	COMPLETE.ARO.PWY	Vocabulary	Caffeine	-0.661	2.763	0.834	0.974
Meconium Adj.[c]	COMPLETE.ARO.PWY	Vocabulary	Pathway	-88.279	373.159		

Exposure Window	Pathway	Outcome	Variable	Effect Estimate[a]	Std. Error	LRT[b]	
						p-value	q-value
Meconium Adj.[c]	COMPLETE.ARO.PWY	Vocabulary	Interaction	1.011	1.084		
Meconium Adj.[c]	COMPLETE.ARO.PWY	WISC sum	Caffeine	-0.593	6.201	0.971	0.982
Meconium Adj.[c]	COMPLETE.ARO.PWY	WISC sum	Pathway	81.37	837.599		
Meconium Adj.[c]	COMPLETE.ARO.PWY	WISC sum	Interaction	2.897	2.434		
Meconium Adj.[c]	DTDPRHAMSYN.PWY	Block Design	Caffeine	-0.089	1.235	0.892	0.974
Meconium Adj.[c]	DTDPRHAMSYN.PWY	Block Design	Pathway	-42.135	183.085		
Meconium Adj.[c]	DTDPRHAMSYN.PWY	Block Design	Interaction	0.368	0.924		
Meconium Adj.[c]	DTDPRHAMSYN.PWY	Coding	Caffeine	1.911	1.068	0.065	0.868
Meconium Adj.[c]	DTDPRHAMSYN.PWY	Coding	Pathway	227.762	158.316		
Meconium Adj.[c]	DTDPRHAMSYN.PWY	Coding	Interaction	0.671	0.799		
Meconium Adj.[c]	DTDPRHAMSYN.PWY	Digit span	Caffeine	-0.528	0.938	0.803	0.974
Meconium Adj.[c]	DTDPRHAMSYN.PWY	Digit span	Pathway	-24.39	139.015		
Meconium Adj.[c]	DTDPRHAMSYN.PWY	Digit span	Interaction	-0.143	0.702		
Meconium Adj.[c]	DTDPRHAMSYN.PWY	Information	Caffeine	0.135	0.932	0.768	0.974
Meconium Adj.[c]	DTDPRHAMSYN.PWY	Information	Pathway	19.308	138.054		
Meconium Adj.[c]	DTDPRHAMSYN.PWY	Information	Interaction	1.001	0.697		
Meconium Adj.[c]	DTDPRHAMSYN.PWY	QTAC	Caffeine	-3.699	3.214	0.31	0.974
Meconium Adj.[c]	DTDPRHAMSYN.PWY	QTAC	Pathway	-414.18	476.311		
Meconium Adj.[c]	DTDPRHAMSYN.PWY	QTAC	Interaction	-4.634	2.404		
Meconium Adj.[c]	DTDPRHAMSYN.PWY	Vocabulary	Caffeine	-0.158	1.427	0.988	0.988
Meconium Adj.[c]	DTDPRHAMSYN.PWY	Vocabulary	Pathway	-12.73	211.524		
Meconium Adj.[c]	DTDPRHAMSYN.PWY	Vocabulary	Interaction	1.081	1.067		
Meconium Adj.[c]	DTDPRHAMSYN.PWY	WISC sum	Caffeine	1.271	3.156	0.488	0.974
Meconium Adj.[c]	DTDPRHAMSYN.PWY	WISC sum	Pathway	167.815	467.747		
Meconium Adj.[c]	DTDPRHAMSYN.PWY	WISC sum	Interaction	2.979	2.361		
Meconium Adj.[c]	GLUTORN.PWY	Block Design	Caffeine	2.214	2.038	0.176	0.922
Meconium Adj.[c]	GLUTORN.PWY	Block Design	Pathway	337.677	291.501		
Meconium Adj.[c]	GLUTORN.PWY	Block Design	Interaction	0.349	0.934		
Meconium Adj.[c]	GLUTORN.PWY	Coding	Caffeine	-0.095	1.824	0.913	0.978
Meconium Adj.[c]	GLUTORN.PWY	Coding	Pathway	7.987	260.91		
Meconium Adj.[c]	GLUTORN.PWY	Coding	Interaction	0.668	0.836		
Meconium Adj.[c]	GLUTORN.PWY	Digit span	Caffeine	-2.311	1.475	0.142	0.922
Meconium Adj.[c]	GLUTORN.PWY	Digit span	Pathway	-215.042	210.997		
Meconium Adj.[c]	GLUTORN.PWY	Digit span	Interaction	-0.069	0.676		
Meconium Adj.[c]	GLUTORN.PWY	Information	Caffeine	-1.144	1.526	0.453	0.974
Meconium Adj.[c]	GLUTORN.PWY	Information	Pathway	-167.615	218.219		

Exposure Window	Pathway	Outcome	Variable	Effect Estimate[a]	Std. Error	LRT[b]	
						p-value	q-value
Meconium Adj.[c]	GLUTORN.PWY	Information	Interaction	0.896	0.699		
Meconium Adj.[c]	GLUTORN.PWY	QTAC	Caffeine	-10.522	5.107	0.038	0.868
Meconium Adj.[c]	GLUTORN.PWY	QTAC	Pathway	-1296.105	730.386		
Meconium Adj.[c]	GLUTORN.PWY	QTAC	Interaction	-4.753	2.341		
Meconium Adj.[c]	GLUTORN.PWY	Vocabulary	Caffeine	-1.469	2.341	0.533	0.974
Meconium Adj.[c]	GLUTORN.PWY	Vocabulary	Pathway	-167.493	334.763		
Meconium Adj.[c]	GLUTORN.PWY	Vocabulary	Interaction	1.062	1.073		
Meconium Adj.[c]	GLUTORN.PWY	WISC sum	Caffeine	-2.806	5.253	0.673	0.974
Meconium Adj.[c]	GLUTORN.PWY	WISC sum	Pathway	-204.486	751.302		
Meconium Adj.[c]	GLUTORN.PWY	WISC sum	Interaction	2.905	2.408		
Meconium Adj.[c]	GLYCOGENSYNTH.PWY	Block Design	Caffeine	0.456	1.244	0.502	0.974
Meconium Adj.[c]	GLYCOGENSYNTH.PWY	Block Design	Pathway	109.309	192.201		
Meconium Adj.[c]	GLYCOGENSYNTH.PWY	Block Design	Interaction	0.322	0.941		
Meconium Adj.[c]	GLYCOGENSYNTH.PWY	Coding	Caffeine	0.056	1.092	0.939	0.978
Meconium Adj.[c]	GLYCOGENSYNTH.PWY	Coding	Pathway	-48.886	168.684		
Meconium Adj.[c]	GLYCOGENSYNTH.PWY	Coding	Interaction	0.66	0.826		
Meconium Adj.[c]	GLYCOGENSYNTH.PWY	Digit span	Caffeine	-0.459	0.918	0.889	0.974
Meconium Adj.[c]	GLYCOGENSYNTH.PWY	Digit span	Pathway	18.135	141.881		
Meconium Adj.[c]	GLYCOGENSYNTH.PWY	Digit span	Interaction	-0.191	0.695		
Meconium Adj.[c]	GLYCOGENSYNTH.PWY	Information	Caffeine	-0.175	0.926	0.945	0.978
Meconium Adj.[c]	GLYCOGENSYNTH.PWY	Information	Pathway	-14.191	143.004		
Meconium Adj.[c]	GLYCOGENSYNTH.PWY	Information	Interaction	0.985	0.7		
Meconium Adj.[c]	GLYCOGENSYNTH.PWY	QTAC	Caffeine	-4.692	3.139	0.158	0.922
Meconium Adj.[c]	GLYCOGENSYNTH.PWY	QTAC	Pathway	-678.322	485.05		
Meconium Adj.[c]	GLYCOGENSYNTH.PWY	QTAC	Interaction	-4.581	2.374		
Meconium Adj.[c]	GLYCOGENSYNTH.PWY	Vocabulary	Caffeine	-0.787	1.41	0.607	0.974
Meconium Adj.[c]	GLYCOGENSYNTH.PWY	Vocabulary	Pathway	-105.683	217.913		
Meconium Adj.[c]	GLYCOGENSYNTH.PWY	Vocabulary	Interaction	1.062	1.067		
Meconium Adj.[c]	GLYCOGENSYNTH.PWY	WISC sum	Caffeine	-0.909	3.174	0.959	0.978
Meconium Adj.[c]	GLYCOGENSYNTH.PWY	WISC sum	Pathway	-41.316	490.417		
Meconium Adj.[c]	GLYCOGENSYNTH.PWY	WISC sum	Interaction	2.838	2.401		
Meconium Adj.[c]	ILEUSYN.PWY	Block Design	Caffeine	4.351	2.687	0.06	0.868
Meconium Adj.[c]	ILEUSYN.PWY	Block Design	Pathway	514.528	312.467		
Meconium Adj.[c]	ILEUSYN.PWY	Block Design	Interaction	0.592	0.952		
Meconium Adj.[c]	ILEUSYN.PWY	Coding	Caffeine	1.294	2.454	0.589	0.974
Meconium Adj.[c]	ILEUSYN.PWY	Coding	Pathway	131.428	285.34		

Exposure Window	Pathway	Outcome	Variable	Effect Estimate[a]	Std. Error	LRT[b]	
						p-value	q-value
Meconium Adj.[c]	ILEUSYN.PWY	Coding	Interaction	0.677	0.869		
Meconium Adj.[c]	ILEUSYN.PWY	Digit span	Caffeine	-2.97	1.963	0.139	0.922
Meconium Adj.[c]	ILEUSYN.PWY	Digit span	Pathway	-235.247	228.316		
Meconium Adj.[c]	ILEUSYN.PWY	Digit span	Interaction	-0.095	0.695		
Meconium Adj.[c]	ILEUSYN.PWY	Information	Caffeine	-2.241	1.984	0.237	0.923
Meconium Adj.[c]	ILEUSYN.PWY	Information	Pathway	-304.853	230.778		
Meconium Adj.[c]	ILEUSYN.PWY	Information	Interaction	0.595	0.703		
Meconium Adj.[c]	ILEUSYN.PWY	QTAC	Caffeine	-12.252	6.933	0.071	0.868
Meconium Adj.[c]	ILEUSYN.PWY	QTAC	Pathway	-1258.574	806.258		
Meconium Adj.[c]	ILEUSYN.PWY	QTAC	Interaction	-5.264	2.456		
Meconium Adj.[c]	ILEUSYN.PWY	Vocabulary	Caffeine	-2.071	3.125	0.494	0.974
Meconium Adj.[c]	ILEUSYN.PWY	Vocabulary	Pathway	-263.933	363.437		
Meconium Adj.[c]	ILEUSYN.PWY	Vocabulary	Interaction	0.767	1.107		
Meconium Adj.[c]	ILEUSYN.PWY	WISC sum	Caffeine	-1.637	7.071	0.892	0.974
Meconium Adj.[c]	ILEUSYN.PWY	WISC sum	Pathway	-158.077	822.293		
Meconium Adj.[c]	ILEUSYN.PWY	WISC sum	Interaction	2.536	2.504		
Meconium Adj.[c]	PEPTIDOGLYCANSYN.PWY	Block Design	Caffeine	-1.884	3.801	0.642	0.974
Meconium Adj.[c]	PEPTIDOGLYCANSYN.PWY	Block Design	Pathway	-219.148	492.888		
Meconium Adj.[c]	PEPTIDOGLYCANSYN.PWY	Block Design	Interaction	0.286	0.942		
Meconium Adj.[c]	PEPTIDOGLYCANSYN.PWY	Coding	Caffeine	0.443	3.337	0.905	0.974
Meconium Adj.[c]	PEPTIDOGLYCANSYN.PWY	Coding	Pathway	117.721	432.745		
Meconium Adj.[c]	PEPTIDOGLYCANSYN.PWY	Coding	Interaction	0.649	0.827		
Meconium Adj.[c]	PEPTIDOGLYCANSYN.PWY	Digit span	Caffeine	-1.688	2.752	0.588	0.974
Meconium Adj.[c]	PEPTIDOGLYCANSYN.PWY	Digit span	Pathway	-55.074	356.8		
Meconium Adj.[c]	PEPTIDOGLYCANSYN.PWY	Digit span	Interaction	-0.055	0.682		
Meconium Adj.[c]	PEPTIDOGLYCANSYN.PWY	Information	Caffeine	-0.596	2.798	0.856	0.974
Meconium Adj.[c]	PEPTIDOGLYCANSYN.PWY	Information	Pathway	6.973	362.832		
Meconium Adj.[c]	PEPTIDOGLYCANSYN.PWY	Information	Interaction	1.021	0.693		
Meconium Adj.[c]	PEPTIDOGLYCANSYN.PWY	QTAC	Caffeine	-20.134	9.3	0.025	0.868
Meconium Adj.[c]	PEPTIDOGLYCANSYN.PWY	QTAC	Pathway	-2339.636	1205.871		
Meconium Adj.[c]	PEPTIDOGLYCANSYN.PWY	QTAC	Interaction	-4.457	2.304		
Meconium Adj.[c]	PEPTIDOGLYCANSYN.PWY	Vocabulary	Caffeine	-2.378	4.245	0.566	0.974
Meconium Adj.[c]	PEPTIDOGLYCANSYN.PWY	Vocabulary	Pathway	-158.769	550.393		
Meconium Adj.[c]	PEPTIDOGLYCANSYN.PWY	Vocabulary	Interaction	1.152	1.052		
Meconium Adj.[c]	PEPTIDOGLYCANSYN.PWY	WISC sum	Caffeine	-6.103	9.433	0.538	0.974
Meconium Adj.[c]	PEPTIDOGLYCANSYN.PWY	WISC sum	Pathway	-308.296	1223.2		

Exposure Window	Pathway	Outcome	Variable	Effect Estimate[a]	Std. Error	LRT[b]	
						p-value	q-value
Meconium Adj.[c]	PEPTIDOGLYCANSYN.PWY	WISC sum	Interaction	3.053	2.337		
Meconium Adj.[c]	PWY.1042	Block Design	Caffeine	1.996	2.256	0.263	0.923
Meconium Adj.[c]	PWY.1042	Block Design	Pathway	240.483	263.012		
Meconium Adj.[c]	PWY.1042	Block Design	Interaction	0.386	0.947		
Meconium Adj.[c]	PWY.1042	Coding	Caffeine	1.198	2.001	0.541	0.974
Meconium Adj.[c]	PWY.1042	Coding	Pathway	159.967	233.246		
Meconium Adj.[c]	PWY.1042	Coding	Interaction	0.748	0.84		
Meconium Adj.[c]	PWY.1042	Digit span	Caffeine	-1.558	1.68	0.414	0.974
Meconium Adj.[c]	PWY.1042	Digit span	Pathway	-109.056	195.802		
Meconium Adj.[c]	PWY.1042	Digit span	Interaction	-0.134	0.705		
Meconium Adj.[c]	PWY.1042	Information	Caffeine	-0.609	1.691	0.748	0.974
Meconium Adj.[c]	PWY.1042	Information	Pathway	-71.254	197.145		
Meconium Adj.[c]	PWY.1042	Information	Interaction	0.913	0.71		
Meconium Adj.[c]	PWY.1042	QTAC	Caffeine	-6.539	5.814	0.274	0.923
Meconium Adj.[c]	PWY.1042	QTAC	Pathway	-608.346	677.69		
Meconium Adj.[c]	PWY.1042	QTAC	Interaction	-4.81	2.44		
Meconium Adj.[c]	PWY.1042	Vocabulary	Caffeine	0.646	2.584	0.734	0.974
Meconium Adj.[c]	PWY.1042	Vocabulary	Pathway	68.346	301.209		
Meconium Adj.[c]	PWY.1042	Vocabulary	Interaction	1.068	1.085		
Meconium Adj.[c]	PWY.1042	WISC sum	Caffeine	1.674	5.802	0.639	0.974
Meconium Adj.[c]	PWY.1042	WISC sum	Pathway	288.485	676.345		
Meconium Adj.[c]	PWY.1042	WISC sum	Interaction	2.981	2.436		
Meconium Adj.[c]	PWY.3841	Block Design	Caffeine	1.513	4.311	0.642	0.974
Meconium Adj.[c]	PWY.3841	Block Design	Pathway	247.355	696.66		
Meconium Adj.[c]	PWY.3841	Block Design	Interaction	0.323	0.945		
Meconium Adj.[c]	PWY.3841	Coding	Caffeine	1.471	3.774	0.689	0.974
Meconium Adj.[c]	PWY.3841	Coding	Pathway	317.526	609.875		
Meconium Adj.[c]	PWY.3841	Coding	Interaction	0.67	0.827		
Meconium Adj.[c]	PWY.3841	Digit span	Caffeine	-2.604	3.063	0.405	0.974
Meconium Adj.[c]	PWY.3841	Digit span	Pathway	-190.327	494.975		
Meconium Adj.[c]	PWY.3841	Digit span	Interaction	-0.129	0.671		
Meconium Adj.[c]	PWY.3841	Information	Caffeine	-3.472	3.103	0.233	0.923
Meconium Adj.[c]	PWY.3841	Information	Pathway	-419.237	501.361		
Meconium Adj.[c]	PWY.3841	Information	Interaction	0.936	0.68		
Meconium Adj.[c]	PWY.3841	QTAC	Caffeine	-7.989	11.067	0.472	0.974
Meconium Adj.[c]	PWY.3841	QTAC	Pathway	-1243.731	1788.349		

Exposure Window	Pathway	Outcome	Variable	Effect Estimate[a]	Std. Error	LRT[b]	
						p-value	q-value
Meconium Adj.[c]	PWY.3841	QTAC	Interaction	-4.776	2.426		
Meconium Adj.[c]	PWY.3841	Vocabulary	Caffeine	1.159	4.891	0.768	0.974
Meconium Adj.[c]	PWY.3841	Vocabulary	Pathway	222.389	790.324		
Meconium Adj.[c]	PWY.3841	Vocabulary	Interaction	1.106	1.072		
Meconium Adj.[c]	PWY.3841	WISC sum	Caffeine	-1.933	10.819	0.903	0.974
Meconium Adj.[c]	PWY.3841	WISC sum	Pathway	177.705	1748.332		
Meconium Adj.[c]	PWY.3841	WISC sum	Interaction	2.906	2.372		
Meconium Adj.[c]	PWY.4242	Block Design	Caffeine	1.122	1.944	0.426	0.974
Meconium Adj.[c]	PWY.4242	Block Design	Pathway	167.197	335.881		
Meconium Adj.[c]	PWY.4242	Block Design	Interaction	0.299	0.926		
Meconium Adj.[c]	PWY.4242	Coding	Caffeine	0.369	1.738	0.857	0.974
Meconium Adj.[c]	PWY.4242	Coding	Pathway	83.434	300.232		
Meconium Adj.[c]	PWY.4242	Coding	Interaction	0.59	0.828		
Meconium Adj.[c]	PWY.4242	Digit span	Caffeine	-0.235	1.419	0.933	0.978
Meconium Adj.[c]	PWY.4242	Digit span	Pathway	113.562	245.055		
Meconium Adj.[c]	PWY.4242	Digit span	Interaction	-0.162	0.676		
Meconium Adj.[c]	PWY.4242	Information	Caffeine	-0.789	1.458	0.608	0.974
Meconium Adj.[c]	PWY.4242	Information	Pathway	-137.369	251.832		
Meconium Adj.[c]	PWY.4242	Information	Interaction	0.993	0.695		
Meconium Adj.[c]	PWY.4242	QTAC	Caffeine	-4.736	5.024	0.386	0.974
Meconium Adj.[c]	PWY.4242	QTAC	Pathway	-534.571	867.916		
Meconium Adj.[c]	PWY.4242	QTAC	Interaction	-4.57	2.394		
Meconium Adj.[c]	PWY.4242	Vocabulary	Caffeine	-0.91	2.182	0.685	0.974
Meconium Adj.[c]	PWY.4242	Vocabulary	Pathway	-251.849	376.982		
Meconium Adj.[c]	PWY.4242	Vocabulary	Interaction	1.114	1.04		
Meconium Adj.[c]	PWY.4242	WISC sum	Caffeine	-0.443	5.013	0.944	0.978
Meconium Adj.[c]	PWY.4242	WISC sum	Pathway	-25.025	865.979		
Meconium Adj.[c]	PWY.4242	WISC sum	Interaction	2.833	2.388		
Meconium Adj.[c]	PWY.5097	Block Design	Caffeine	2.08	2.908	0.366	0.974
Meconium Adj.[c]	PWY.5097	Block Design	Pathway	394.385	455.054		
Meconium Adj.[c]	PWY.5097	Block Design	Interaction	0.316	0.943		
Meconium Adj.[c]	PWY.5097	Coding	Caffeine	2.846	2.524	0.228	0.923
Meconium Adj.[c]	PWY.5097	Coding	Pathway	504.75	394.963		
Meconium Adj.[c]	PWY.5097	Coding	Interaction	0.575	0.818		
Meconium Adj.[c]	PWY.5097	Digit span	Caffeine	-2.017	2.031	0.361	0.974
Meconium Adj.[c]	PWY.5097	Digit span	Pathway	-95.641	317.809		

Exposure Window	Pathway	Outcome	Variable	Effect Estimate[a]	Std. Error	LRT[b]	
						p-value	q-value
Meconium Adj.[c]	PWY.5097	Digit span	Interaction	-0.368	0.658		
Meconium Adj.[c]	PWY.5097	Information	Caffeine	-1.41	2.158	0.511	0.974
Meconium Adj.[c]	PWY.5097	Information	Pathway	-157.908	337.655		
Meconium Adj.[c]	PWY.5097	Information	Interaction	0.9	0.7		
Meconium Adj.[c]	PWY.5097	QTAC	Caffeine	-10.341	7.384	0.155	0.922
Meconium Adj.[c]	PWY.5097	QTAC	Pathway	-1344.469	1155.494		
Meconium Adj.[c]	PWY.5097	QTAC	Interaction	-4.953	2.394		
Meconium Adj.[c]	PWY.5097	Vocabulary	Caffeine	-0.791	3.321	0.829	0.974
Meconium Adj.[c]	PWY.5097	Vocabulary	Pathway	-108.691	519.635		
Meconium Adj.[c]	PWY.5097	Vocabulary	Interaction	1.066	1.077		
Meconium Adj.[c]	PWY.5097	WISC sum	Caffeine	0.708	7.318	0.816	0.974
Meconium Adj.[c]	PWY.5097	WISC sum	Pathway	536.895	1145.064		
Meconium Adj.[c]	PWY.5097	WISC sum	Interaction	2.489	2.372		
Meconium Adj.[c]	PWY.5103	Block Design	Caffeine	2.801	1.934	0.081	0.868
Meconium Adj.[c]	PWY.5103	Block Design	Pathway	357.158	257.118		
Meconium Adj.[c]	PWY.5103	Block Design	Interaction	0.299	0.911		
Meconium Adj.[c]	PWY.5103	Coding	Caffeine	0.449	1.763	0.83	0.974
Meconium Adj.[c]	PWY.5103	Coding	Pathway	12.174	234.348		
Meconium Adj.[c]	PWY.5103	Coding	Interaction	0.573	0.83		
Meconium Adj.[c]	PWY.5103	Digit span	Caffeine	-1.878	1.472	0.241	0.923
Meconium Adj.[c]	PWY.5103	Digit span	Pathway	-191.164	195.672		
Meconium Adj.[c]	PWY.5103	Digit span	Interaction	-0.147	0.693		
Meconium Adj.[c]	PWY.5103	Information	Caffeine	0.862	1.447	0.458	0.974
Meconium Adj.[c]	PWY.5103	Information	Pathway	65.262	192.35		
Meconium Adj.[c]	PWY.5103	Information	Interaction	0.927	0.681		
Meconium Adj.[c]	PWY.5103	QTAC	Caffeine	-10.203	4.945	0.038	0.868
Meconium Adj.[c]	PWY.5103	QTAC	Pathway	-1229.169	657.437		
Meconium Adj.[c]	PWY.5103	QTAC	Interaction	-4.745	2.328		
Meconium Adj.[c]	PWY.5103	Vocabulary	Caffeine	-0.377	2.181	0.891	0.974
Meconium Adj.[c]	PWY.5103	Vocabulary	Pathway	-159.784	289.976		
Meconium Adj.[c]	PWY.5103	Vocabulary	Interaction	0.922	1.027		
Meconium Adj.[c]	PWY.5103	WISC sum	Caffeine	1.857	4.923	0.564	0.974
Meconium Adj.[c]	PWY.5103	WISC sum	Pathway	83.646	654.555		
Meconium Adj.[c]	PWY.5103	WISC sum	Interaction	2.574	2.318		
Meconium Adj.[c]	PWY.5686	Block Design	Caffeine	4.135	3.247	0.136	0.922
Meconium Adj.[c]	PWY.5686	Block Design	Pathway	532.38	426.988		

Exposure Window	Pathway	Outcome	Variable	Effect Estimate[a]	Std. Error	LRT[b]	
						p-value	q-value
Meconium Adj.[c]	PWY.5686	Block Design	Interaction	0.314	0.919		
Meconium Adj.[c]	PWY.5686	Coding	Caffeine	-2.779	2.9	0.275	0.923
Meconium Adj.[c]	PWY.5686	Coding	Pathway	-352.94	381.345		
Meconium Adj.[c]	PWY.5686	Coding	Interaction	0.59	0.82		
Meconium Adj.[c]	PWY.5686	Digit span	Caffeine	-2.491	2.387	0.32	0.974
Meconium Adj.[c]	PWY.5686	Digit span	Pathway	-180.334	313.865		
Meconium Adj.[c]	PWY.5686	Digit span	Interaction	-0.144	0.675		
Meconium Adj.[c]	PWY.5686	Information	Caffeine	-1.281	2.459	0.601	0.974
Meconium Adj.[c]	PWY.5686	Information	Pathway	-158.935	323.31		
Meconium Adj.[c]	PWY.5686	Information	Interaction	0.973	0.696		
Meconium Adj.[c]	PWY.5686	QTAC	Caffeine	-11.297	8.391	0.167	0.922
Meconium Adj.[c]	PWY.5686	QTAC	Pathway	-1446.595	1103.438		
Meconium Adj.[c]	PWY.5686	QTAC	Interaction	-4.618	2.374		
Meconium Adj.[c]	PWY.5686	Vocabulary	Caffeine	-0.254	3.72	0.968	0.982
Meconium Adj.[c]	PWY.5686	Vocabulary	Pathway	-118.804	489.183		
Meconium Adj.[c]	PWY.5686	Vocabulary	Interaction	1.071	1.052		
Meconium Adj.[c]	PWY.5686	WISC sum	Caffeine	-2.67	8.45	0.803	0.974
Meconium Adj.[c]	PWY.5686	WISC sum	Pathway	-278.632	1111.09		
Meconium Adj.[c]	PWY.5686	WISC sum	Interaction	2.805	2.39		
Meconium Adj.[c]	PWY.6121	Block Design	Caffeine	1.475	2.42	0.422	0.974
Meconium Adj.[c]	PWY.6121	Block Design	Pathway	212.642	347.572		
Meconium Adj.[c]	PWY.6121	Block Design	Interaction	0.299	0.938		
Meconium Adj.[c]	PWY.6121	Coding	Caffeine	0.53	2.154	0.822	0.974
Meconium Adj.[c]	PWY.6121	Coding	Pathway	72.468	309.355		
Meconium Adj.[c]	PWY.6121	Coding	Interaction	0.626	0.834		
Meconium Adj.[c]	PWY.6121	Digit span	Caffeine	-1.326	1.715	0.532	0.974
Meconium Adj.[c]	PWY.6121	Digit span	Pathway	-28.117	246.297		
Meconium Adj.[c]	PWY.6121	Digit span	Interaction	-0.041	0.664		
Meconium Adj.[c]	PWY.6121	Information	Caffeine	-1.306	1.779	0.469	0.974
Meconium Adj.[c]	PWY.6121	Information	Pathway	-112.874	255.548		
Meconium Adj.[c]	PWY.6121	Information	Interaction	1.004	0.689		
Meconium Adj.[c]	PWY.6121	QTAC	Caffeine	-8.477	6.161	0.167	0.922
Meconium Adj.[c]	PWY.6121	QTAC	Pathway	-1147.729	885.04		
Meconium Adj.[c]	PWY.6121	QTAC	Interaction	-4.822	2.387		
Meconium Adj.[c]	PWY.6121	Vocabulary	Caffeine	-1.546	2.75	0.576	0.974
Meconium Adj.[c]	PWY.6121	Vocabulary	Pathway	-163.514	395.108		

Exposure Window	Pathway	Outcome	Variable	Effect Estimate[a]	Std. Error	LRT[b]	
						p-value	q-value
Meconium Adj.[c]	PWY.6121	Vocabulary	Interaction	1.07	1.066		
Meconium Adj.[c]	PWY.6121	WISC sum	Caffeine	-2.172	6.136		
Meconium Adj.[c]	PWY.6121	WISC sum	Pathway	-19.395	881.444	0.811	0.974
Meconium Adj.[c]	PWY.6121	WISC sum	Interaction	2.958	2.378		
Meconium Adj.[c]	PWY.6122	Block Design	Caffeine	2.592	2.754		
Meconium Adj.[c]	PWY.6122	Block Design	Pathway	349.679	377.187	0.251	0.923
Meconium Adj.[c]	PWY.6122	Block Design	Interaction	0.286	0.928		
Meconium Adj.[c]	PWY.6122	Coding	Caffeine	-0.318	2.468		
Meconium Adj.[c]	PWY.6122	Coding	Pathway	-48.095	338.13	0.853	0.974
Meconium Adj.[c]	PWY.6122	Coding	Interaction	0.606	0.832		
Meconium Adj.[c]	PWY.6122	Digit span	Caffeine	-1.562	1.939		
Meconium Adj.[c]	PWY.6122	Digit span	Pathway	-33.658	265.668	0.496	0.974
Meconium Adj.[c]	PWY.6122	Digit span	Interaction	-0.042	0.654		
Meconium Adj.[c]	PWY.6122	Information	Caffeine	0.292	2.058		
Meconium Adj.[c]	PWY.6122	Information	Pathway	100.908	281.858	0.815	0.974
Meconium Adj.[c]	PWY.6122	Information	Interaction	1.015	0.694		
Meconium Adj.[c]	PWY.6122	QTAC	Caffeine	-15.307	6.828		
Meconium Adj.[c]	PWY.6122	QTAC	Pathway	-1948.26	935.277	0.021	0.868
Meconium Adj.[c]	PWY.6122	QTAC	Interaction	-4.675	2.302		
Meconium Adj.[c]	PWY.6122	Vocabulary	Caffeine	-0.705	3.146		
Meconium Adj.[c]	PWY.6122	Vocabulary	Pathway	-8.591	430.897	0.852	0.974
Meconium Adj.[c]	PWY.6122	Vocabulary	Interaction	1.115	1.061		
Meconium Adj.[c]	PWY.6122	WISC sum	Caffeine	0.3	7.01		
Meconium Adj.[c]	PWY.6122	WISC sum	Pathway	360.243	960.221	0.853	0.974
Meconium Adj.[c]	PWY.6122	WISC sum	Interaction	2.98	2.363		
Meconium Adj.[c]	PWY.6151	Block Design	Caffeine	3.113	2.196		
Meconium Adj.[c]	PWY.6151	Block Design	Pathway	468.662	320.441	0.09	0.868
Meconium Adj.[c]	PWY.6151	Block Design	Interaction	0.399	0.917		
Meconium Adj.[c]	PWY.6151	Coding	Caffeine	0.376	1.988		
Meconium Adj.[c]	PWY.6151	Coding	Pathway	76.748	290.084	0.874	0.974
Meconium Adj.[c]	PWY.6151	Coding	Interaction	0.629	0.83		
Meconium Adj.[c]	PWY.6151	Digit span	Caffeine	-2.113	1.651		
Meconium Adj.[c]	PWY.6151	Digit span	Pathway	-223.34	240.906	0.231	0.923
Meconium Adj.[c]	PWY.6151	Digit span	Interaction	-0.177	0.689		
Meconium Adj.[c]	PWY.6151	Information	Caffeine	-0.566	1.659	0.762	0.974
Meconium Adj.[c]	PWY.6151	Information	Pathway	-108.945	242.123		

Exposure Window	Pathway	Outcome	Variable	Effect Estimate[a]	Std. Error	LRT[b]	
						p-value	q-value
Meconium Adj.[c]	PWY.6151	Information	Interaction	0.943	0.693		
Meconium Adj.[c]	PWY.6151	QTAC	Caffeine	-9.711	5.652	0.083	0.868
Meconium Adj.[c]	PWY.6151	QTAC	Pathway	-1278.832	824.903		
Meconium Adj.[c]	PWY.6151	QTAC	Interaction	-4.87	2.36		
Meconium Adj.[c]	PWY.6151	Vocabulary	Caffeine	0.803	2.548	0.681	0.974
Meconium Adj.[c]	PWY.6151	Vocabulary	Pathway	93.066	371.821		
Meconium Adj.[c]	PWY.6151	Vocabulary	Interaction	1.083	1.064		
Meconium Adj.[c]	PWY.6151	WISC sum	Caffeine	1.612	5.735	0.644	0.974
Meconium Adj.[c]	PWY.6151	WISC sum	Pathway	306.191	836.957		
Meconium Adj.[c]	PWY.6151	WISC sum	Interaction	2.877	2.395		
Meconium Adj.[c]	PWY.6163	Block Design	Caffeine	2.573	2.316	0.176	0.922
Meconium Adj.[c]	PWY.6163	Block Design	Pathway	328.718	296.846		
Meconium Adj.[c]	PWY.6163	Block Design	Interaction	0.417	0.946		
Meconium Adj.[c]	PWY.6163	Coding	Caffeine	0.65	2.065	0.754	0.974
Meconium Adj.[c]	PWY.6163	Coding	Pathway	123.177	264.66		
Meconium Adj.[c]	PWY.6163	Coding	Interaction	0.761	0.844		
Meconium Adj.[c]	PWY.6163	Digit span	Caffeine	-2.27	1.696	0.212	0.923
Meconium Adj.[c]	PWY.6163	Digit span	Pathway	-180.184	217.368		
Meconium Adj.[c]	PWY.6163	Digit span	Interaction	-0.119	0.693		
Meconium Adj.[c]	PWY.6163	Information	Caffeine	-1.587	1.735	0.348	0.974
Meconium Adj.[c]	PWY.6163	Information	Pathway	-199.188	222.441		
Meconium Adj.[c]	PWY.6163	Information	Interaction	0.845	0.709		
Meconium Adj.[c]	PWY.6163	QTAC	Caffeine	-8.712	5.959	0.144	0.922
Meconium Adj.[c]	PWY.6163	QTAC	Pathway	-974.004	763.858		
Meconium Adj.[c]	PWY.6163	QTAC	Interaction	-5.057	2.435		
Meconium Adj.[c]	PWY.6163	Vocabulary	Caffeine	-1.094	2.673	0.693	0.974
Meconium Adj.[c]	PWY.6163	Vocabulary	Pathway	-127.151	342.686		
Meconium Adj.[c]	PWY.6163	Vocabulary	Interaction	0.988	1.092		
Meconium Adj.[c]	PWY.6163	WISC sum	Caffeine	-1.728	5.998	0.866	0.974
Meconium Adj.[c]	PWY.6163	WISC sum	Pathway	-54.627	768.956		
Meconium Adj.[c]	PWY.6163	WISC sum	Interaction	2.893	2.451		
Meconium Adj.[c]	PWY.6277	Block Design	Caffeine	2.592	2.754	0.251	0.923
Meconium Adj.[c]	PWY.6277	Block Design	Pathway	349.679	377.187		
Meconium Adj.[c]	PWY.6277	Block Design	Interaction	0.286	0.928		
Meconium Adj.[c]	PWY.6277	Coding	Caffeine	-0.318	2.468	0.853	0.974
Meconium Adj.[c]	PWY.6277	Coding	Pathway	-48.095	338.13		

Exposure Window	Pathway	Outcome	Variable	Effect Estimate[a]	Std. Error	LRT[b]	
						p-value	q-value
Meconium Adj.[c]	PWY.6277	Coding	Interaction	0.606	0.832		
Meconium Adj.[c]	PWY.6277	Digit span	Caffeine	-1.562	1.939	0.496	0.974
Meconium Adj.[c]	PWY.6277	Digit span	Pathway	-33.658	265.668		
Meconium Adj.[c]	PWY.6277	Digit span	Interaction	-0.042	0.654		
Meconium Adj.[c]	PWY.6277	Information	Caffeine	0.292	2.058	0.815	0.974
Meconium Adj.[c]	PWY.6277	Information	Pathway	100.908	281.858		
Meconium Adj.[c]	PWY.6277	Information	Interaction	1.015	0.694		
Meconium Adj.[c]	PWY.6277	QTAC	Caffeine	-15.307	6.828	0.021	0.868
Meconium Adj.[c]	PWY.6277	QTAC	Pathway	-1948.26	935.277		
Meconium Adj.[c]	PWY.6277	QTAC	Interaction	-4.675	2.302		
Meconium Adj.[c]	PWY.6277	Vocabulary	Caffeine	-0.705	3.146	0.852	0.974
Meconium Adj.[c]	PWY.6277	Vocabulary	Pathway	-8.591	430.897		
Meconium Adj.[c]	PWY.6277	Vocabulary	Interaction	1.115	1.061		
Meconium Adj.[c]	PWY.6277	WISC sum	Caffeine	0.3	7.01	0.853	0.974
Meconium Adj.[c]	PWY.6277	WISC sum	Pathway	360.243	960.221		
Meconium Adj.[c]	PWY.6277	WISC sum	Interaction	2.98	2.363		
Meconium Adj.[c]	PWY.6385	Block Design	Caffeine	1.161	3.567	0.652	0.974
Meconium Adj.[c]	PWY.6385	Block Design	Pathway	193.936	519.873		
Meconium Adj.[c]	PWY.6385	Block Design	Interaction	0.351	0.95		
Meconium Adj.[c]	PWY.6385	Coding	Caffeine	2.492	3.084	0.387	0.974
Meconium Adj.[c]	PWY.6385	Coding	Pathway	444.12	449.522		
Meconium Adj.[c]	PWY.6385	Coding	Interaction	0.683	0.821		
Meconium Adj.[c]	PWY.6385	Digit span	Caffeine	-2.519	2.541	0.344	0.974
Meconium Adj.[c]	PWY.6385	Digit span	Pathway	-182.497	370.389		
Meconium Adj.[c]	PWY.6385	Digit span	Interaction	-0.234	0.677		
Meconium Adj.[c]	PWY.6385	Information	Caffeine	-4.329	2.54	0.072	0.868
Meconium Adj.[c]	PWY.6385	Information	Pathway	-536.714	370.198		
Meconium Adj.[c]	PWY.6385	Information	Interaction	0.808	0.676		
Meconium Adj.[c]	PWY.6385	QTAC	Caffeine	-9.83	9.021	0.271	0.923
Meconium Adj.[c]	PWY.6385	QTAC	Pathway	-1082.688	1314.895		
Meconium Adj.[c]	PWY.6385	QTAC	Interaction	-4.948	2.402		
Meconium Adj.[c]	PWY.6385	Vocabulary	Caffeine	-3.521	4.009	0.352	0.974
Meconium Adj.[c]	PWY.6385	Vocabulary	Pathway	-498.924	584.421		
Meconium Adj.[c]	PWY.6385	Vocabulary	Interaction	0.942	1.067		
Meconium Adj.[c]	PWY.6385	WISC sum	Caffeine	-6.715	8.919	0.466	0.974
Meconium Adj.[c]	PWY.6385	WISC sum	Pathway	-580.08	1299.983		

Exposure Window	Pathway	Outcome	Variable	Effect Estimate[a]	Std. Error	LRT[b]	
						p-value	q-value
Meconium Adj.[c]	PWY.6385	WISC sum	Interaction	2.549	2.374		
Meconium Adj.[c]	PWY.6386	Block Design	Caffeine	1.721	3.825	0.561	0.974
Meconium Adj.[c]	PWY.6386	Block Design	Pathway	241.547	485.408		
Meconium Adj.[c]	PWY.6386	Block Design	Interaction	0.304	0.94		
Meconium Adj.[c]	PWY.6386	Coding	Caffeine	1.324	3.35	0.681	0.974
Meconium Adj.[c]	PWY.6386	Coding	Pathway	227.46	425.196		
Meconium Adj.[c]	PWY.6386	Coding	Interaction	0.644	0.823		
Meconium Adj.[c]	PWY.6386	Digit span	Caffeine	-1.451	2.732	0.654	0.974
Meconium Adj.[c]	PWY.6386	Digit span	Pathway	-7.686	346.739		
Meconium Adj.[c]	PWY.6386	Digit span	Interaction	-0.086	0.671		
Meconium Adj.[c]	PWY.6386	Information	Caffeine	-1.425	2.828	0.612	0.974
Meconium Adj.[c]	PWY.6386	Information	Pathway	-127.417	358.934		
Meconium Adj.[c]	PWY.6386	Information	Interaction	0.983	0.695		
Meconium Adj.[c]	PWY.6386	QTAC	Caffeine	-17.306	9.481	0.058	0.868
Meconium Adj.[c]	PWY.6386	QTAC	Pathway	-1899.855	1203.285		
Meconium Adj.[c]	PWY.6386	QTAC	Interaction	-4.631	2.329		
Meconium Adj.[c]	PWY.6386	Vocabulary	Caffeine	-2.317	4.328	0.58	0.974
Meconium Adj.[c]	PWY.6386	Vocabulary	Pathway	-244.695	549.263		
Meconium Adj.[c]	PWY.6386	Vocabulary	Interaction	1.064	1.063		
Meconium Adj.[c]	PWY.6386	WISC sum	Caffeine	-2.148	9.62	0.88	0.974
Meconium Adj.[c]	PWY.6386	WISC sum	Pathway	89.209	1220.884		
Meconium Adj.[c]	PWY.6386	WISC sum	Interaction	2.909	2.363		
Meconium Adj.[c]	PWY.6387	Block Design	Caffeine	1.839	4.064	0.563	0.974
Meconium Adj.[c]	PWY.6387	Block Design	Pathway	248.878	530.63		
Meconium Adj.[c]	PWY.6387	Block Design	Interaction	0.302	0.94		
Meconium Adj.[c]	PWY.6387	Coding	Caffeine	1.458	3.565	0.67	0.974
Meconium Adj.[c]	PWY.6387	Coding	Pathway	250.888	465.434		
Meconium Adj.[c]	PWY.6387	Coding	Interaction	0.655	0.825		
Meconium Adj.[c]	PWY.6387	Digit span	Caffeine	-1.661	2.925	0.617	0.974
Meconium Adj.[c]	PWY.6387	Digit span	Pathway	-39.944	381.87		
Meconium Adj.[c]	PWY.6387	Digit span	Interaction	-0.08	0.677		
Meconium Adj.[c]	PWY.6387	Information	Caffeine	-1.704	3.01	0.562	0.974
Meconium Adj.[c]	PWY.6387	Information	Pathway	-182.652	393.031		
Meconium Adj.[c]	PWY.6387	Information	Interaction	0.973	0.696		
Meconium Adj.[c]	PWY.6387	QTAC	Caffeine	-17.594	10.116	0.07	0.868
Meconium Adj.[c]	PWY.6387	QTAC	Pathway	-1989.833	1320.791		

Exposure Window	Pathway	Outcome	Variable	Effect Estimate[a]	Std. Error	LRT[b]	
						p-value	q-value
Meconium Adj.[c]	PWY.6387	QTAC	Interaction	-4.656	2.34		
Meconium Adj.[c]	PWY.6387	Vocabulary	Caffeine	-2.704	4.598	0.539	0.974
Meconium Adj.[c]	PWY.6387	Vocabulary	Pathway	-301.679	600.336		
Meconium Adj.[c]	PWY.6387	Vocabulary	Interaction	1.058	1.064		
Meconium Adj.[c]	PWY.6387	WISC sum	Caffeine	-2.773	10.27	0.833	0.974
Meconium Adj.[c]	PWY.6387	WISC sum	Pathway	-24.51	1340.905		
Meconium Adj.[c]	PWY.6387	WISC sum	Interaction	2.908	2.376		
Meconium Adj.[c]	PWY.6609	Block Design	Caffeine	-0.463	1.697	0.898	0.974
Meconium Adj.[c]	PWY.6609	Block Design	Pathway	-99.944	267.224		
Meconium Adj.[c]	PWY.6609	Block Design	Interaction	0.285	0.927		
Meconium Adj.[c]	PWY.6609	Coding	Caffeine	1.026	1.509	0.489	0.974
Meconium Adj.[c]	PWY.6609	Coding	Pathway	172.1	237.724		
Meconium Adj.[c]	PWY.6609	Coding	Interaction	0.603	0.824		
Meconium Adj.[c]	PWY.6609	Digit span	Caffeine	0.062	1.276	0.735	0.974
Meconium Adj.[c]	PWY.6609	Digit span	Pathway	88.292	200.914		
Meconium Adj.[c]	PWY.6609	Digit span	Interaction	-0.13	0.697		
Meconium Adj.[c]	PWY.6609	Information	Caffeine	-1.131	1.266	0.378	0.974
Meconium Adj.[c]	PWY.6609	Information	Pathway	-144.999	199.468		
Meconium Adj.[c]	PWY.6609	Information	Interaction	0.984	0.692		
Meconium Adj.[c]	PWY.6609	QTAC	Caffeine	-1.039	4.438	0.945	0.978
Meconium Adj.[c]	PWY.6609	QTAC	Pathway	-64.866	698.998		
Meconium Adj.[c]	PWY.6609	QTAC	Interaction	-4.573	2.424		
Meconium Adj.[c]	PWY.6609	Vocabulary	Caffeine	-0.322	1.946	0.915	0.978
Meconium Adj.[c]	PWY.6609	Vocabulary	Pathway	-59.569	306.578		
Meconium Adj.[c]	PWY.6609	Vocabulary	Interaction	1.067	1.063		
Meconium Adj.[c]	PWY.6609	WISC sum	Caffeine	-0.828	4.378	0.988	0.988
Meconium Adj.[c]	PWY.6609	WISC sum	Pathway	-44.121	689.498		
Meconium Adj.[c]	PWY.6609	WISC sum	Interaction	2.809	2.391		
Meconium Adj.[c]	PWY.6737	Block Design	Caffeine	1.078	2.078	0.469	0.974
Meconium Adj.[c]	PWY.6737	Block Design	Pathway	62.361	257.768		
Meconium Adj.[c]	PWY.6737	Block Design	Interaction	0.057	0.908		
Meconium Adj.[c]	PWY.6737	Coding	Caffeine	1.259	1.899	0.496	0.974
Meconium Adj.[c]	PWY.6737	Coding	Pathway	185.013	235.638		
Meconium Adj.[c]	PWY.6737	Coding	Interaction	0.739	0.83		
Meconium Adj.[c]	PWY.6737	Digit span	Caffeine	-1.213	1.601	0.536	0.974
Meconium Adj.[c]	PWY.6737	Digit span	Pathway	-70.372	198.641		

Exposure Window	Pathway	Outcome	Variable	Effect Estimate[a]	Std. Error	LRT[b]	
						p-value	q-value
Meconium Adj.[c]	PWY.6737	Digit span	Interaction	-0.052	0.699		
Meconium Adj.[c]	PWY.6737	Information	Caffeine	-0.529	1.611	0.778	0.974
Meconium Adj.[c]	PWY.6737	Information	Pathway	-69.909	199.913		
Meconium Adj.[c]	PWY.6737	Information	Interaction	0.918	0.704		
Meconium Adj.[c]	PWY.6737	QTAC	Caffeine	1.364	5.546	0.666	0.974
Meconium Adj.[c]	PWY.6737	QTAC	Pathway	401.231	688.047		
Meconium Adj.[c]	PWY.6737	QTAC	Interaction	-4.17	2.422		
Meconium Adj.[c]	PWY.6737	Vocabulary	Caffeine	0.446	2.463	0.79	0.974
Meconium Adj.[c]	PWY.6737	Vocabulary	Pathway	42.792	305.532		
Meconium Adj.[c]	PWY.6737	Vocabulary	Interaction	1.008	1.076		
Meconium Adj.[c]	PWY.6737	WISC sum	Caffeine	1.041	5.522	0.715	0.974
Meconium Adj.[c]	PWY.6737	WISC sum	Pathway	149.885	685.065		
Meconium Adj.[c]	PWY.6737	WISC sum	Interaction	2.669	2.412		
Meconium Adj.[c]	PWY.7111	Block Design	Caffeine	3.997	2.649	0.077	0.868
Meconium Adj.[c]	PWY.7111	Block Design	Pathway	465.502	307.435		
Meconium Adj.[c]	PWY.7111	Block Design	Interaction	0.538	0.956		
Meconium Adj.[c]	PWY.7111	Coding	Caffeine	1.755	2.405	0.446	0.974
Meconium Adj.[c]	PWY.7111	Coding	Pathway	187.602	279.098		
Meconium Adj.[c]	PWY.7111	Coding	Interaction	0.728	0.868		
Meconium Adj.[c]	PWY.7111	Digit span	Caffeine	-2.891	1.934	0.146	0.922
Meconium Adj.[c]	PWY.7111	Digit span	Pathway	-227.449	224.462		
Meconium Adj.[c]	PWY.7111	Digit span	Interaction	-0.086	0.698		
Meconium Adj.[c]	PWY.7111	Information	Caffeine	-2.378	1.94	0.198	0.923
Meconium Adj.[c]	PWY.7111	Information	Pathway	-322.689	225.152		
Meconium Adj.[c]	PWY.7111	Information	Interaction	0.559	0.7		
Meconium Adj.[c]	PWY.7111	QTAC	Caffeine	-11.326	6.842	0.092	0.868
Meconium Adj.[c]	PWY.7111	QTAC	Pathway	-1147.458	794.122		
Meconium Adj.[c]	PWY.7111	QTAC	Interaction	-5.183	2.471		
Meconium Adj.[c]	PWY.7111	Vocabulary	Caffeine	-2.438	3.062	0.406	0.974
Meconium Adj.[c]	PWY.7111	Vocabulary	Pathway	-308.763	355.359		
Meconium Adj.[c]	PWY.7111	Vocabulary	Interaction	0.715	1.106		
Meconium Adj.[c]	PWY.7111	WISC sum	Caffeine	-1.955	6.942	0.849	0.974
Meconium Adj.[c]	PWY.7111	WISC sum	Pathway	-205.798	805.705		
Meconium Adj.[c]	PWY.7111	WISC sum	Interaction	2.455	2.507		
Meconium Adj.[c]	PWY.7219	Block Design	Caffeine	1.399	2.474	0.452	0.974
Meconium Adj.[c]	PWY.7219	Block Design	Pathway	172.814	319.614		

Exposure Window	Pathway	Outcome	Variable	Effect Estimate[a]	Std. Error	LRT[b] p-value	LRT[b] q-value
Meconium Adj.[c]	PWY.7219	Block Design	Interaction	0.274	0.934		
Meconium Adj.[c]	PWY.7219	Coding	Caffeine	-0.469	2.18	0.779	0.974
Meconium Adj.[c]	PWY.7219	Coding	Pathway	-15.761	281.74		
Meconium Adj.[c]	PWY.7219	Coding	Interaction	0.589	0.824		
Meconium Adj.[c]	PWY.7219	Digit span	Caffeine	-0.788	1.762	0.78	0.974
Meconium Adj.[c]	PWY.7219	Digit span	Pathway	45.223	227.641		
Meconium Adj.[c]	PWY.7219	Digit span	Interaction	-0.173	0.666		
Meconium Adj.[c]	PWY.7219	Information	Caffeine	-1.779	1.832	0.318	0.974
Meconium Adj.[c]	PWY.7219	Information	Pathway	-205.083	236.755		
Meconium Adj.[c]	PWY.7219	Information	Interaction	1.015	0.692		
Meconium Adj.[c]	PWY.7219	QTAC	Caffeine	-7.642	6.341	0.232	0.923
Meconium Adj.[c]	PWY.7219	QTAC	Pathway	-870.406	819.317		
Meconium Adj.[c]	PWY.7219	QTAC	Interaction	-4.412	2.395		
Meconium Adj.[c]	PWY.7219	Vocabulary	Caffeine	-2.394	2.792	0.37	0.974
Meconium Adj.[c]	PWY.7219	Vocabulary	Pathway	-333.671	360.755		
Meconium Adj.[c]	PWY.7219	Vocabulary	Interaction	1.146	1.055		
Meconium Adj.[c]	PWY.7219	WISC sum	Caffeine	-4.03	6.313	0.57	0.974
Meconium Adj.[c]	PWY.7219	WISC sum	Pathway	-336.477	815.753		
Meconium Adj.[c]	PWY.7219	WISC sum	Interaction	2.851	2.385		
Meconium Adj.[c]	PWY.7221	Block Design	Caffeine	3.592	2.701	0.115	0.922
Meconium Adj.[c]	PWY.7221	Block Design	Pathway	501.819	360.148		
Meconium Adj.[c]	PWY.7221	Block Design	Interaction	0.091	0.934		
Meconium Adj.[c]	PWY.7221	Coding	Caffeine	-0.598	2.436	0.745	0.974
Meconium Adj.[c]	PWY.7221	Coding	Pathway	-127.477	324.804		
Meconium Adj.[c]	PWY.7221	Coding	Interaction	0.682	0.843		
Meconium Adj.[c]	PWY.7221	Digit span	Caffeine	-1.265	2.007	0.615	0.974
Meconium Adj.[c]	PWY.7221	Digit span	Pathway	-25.635	267.648		
Meconium Adj.[c]	PWY.7221	Digit span	Interaction	-0.192	0.694		
Meconium Adj.[c]	PWY.7221	Information	Caffeine	-0.223	2.048	0.952	0.978
Meconium Adj.[c]	PWY.7221	Information	Pathway	-41.645	273.082		
Meconium Adj.[c]	PWY.7221	Information	Interaction	1.016	0.709		
Meconium Adj.[c]	PWY.7221	QTAC	Caffeine	-12.757	6.832	0.055	0.868
Meconium Adj.[c]	PWY.7221	QTAC	Pathway	-1714.992	911.012		
Meconium Adj.[c]	PWY.7221	QTAC	Interaction	-3.763	2.364		
Meconium Adj.[c]	PWY.7221	Vocabulary	Caffeine	-1.9	3.123	0.534	0.974
Meconium Adj.[c]	PWY.7221	Vocabulary	Pathway	-231.322	416.419		

Exposure Window	Pathway	Outcome	Variable	Effect Estimate[a]	Std. Error	LRT[b]	
						p-value	q-value
Meconium Adj.[c]	PWY.7221	Vocabulary	Interaction	1.166	1.08		
Meconium Adj.[c]	PWY.7221	WISC sum	Caffeine	-0.395	7.037	0.95	0.978
Meconium Adj.[c]	PWY.7221	WISC sum	Pathway	75.74	938.274		
Meconium Adj.[c]	PWY.7221	WISC sum	Interaction	2.764	2.434		
Meconium Adj.[c]	PWY.7400	Block Design	Caffeine	2.265	2.534	0.267	0.923
Meconium Adj.[c]	PWY.7400	Block Design	Pathway	300.647	330.919		
Meconium Adj.[c]	PWY.7400	Block Design	Interaction	0.291	0.939		
Meconium Adj.[c]	PWY.7400	Coding	Caffeine	-0.457	2.254	0.789	0.974
Meconium Adj.[c]	PWY.7400	Coding	Pathway	-29.109	294.324		
Meconium Adj.[c]	PWY.7400	Coding	Interaction	0.673	0.835		
Meconium Adj.[c]	PWY.7400	Digit span	Caffeine	-2.247	1.802	0.245	0.923
Meconium Adj.[c]	PWY.7400	Digit span	Pathway	-149.163	235.255		
Meconium Adj.[c]	PWY.7400	Digit span	Interaction	0.018	0.667		
Meconium Adj.[c]	PWY.7400	Information	Caffeine	-1.536	1.891	0.407	0.974
Meconium Adj.[c]	PWY.7400	Information	Pathway	-190.19	246.902		
Meconium Adj.[c]	PWY.7400	Information	Interaction	0.934	0.7		
Meconium Adj.[c]	PWY.7400	QTAC	Caffeine	-12.548	6.341	0.044	0.868
Meconium Adj.[c]	PWY.7400	QTAC	Pathway	-1447.24	828.005		
Meconium Adj.[c]	PWY.7400	QTAC	Interaction	-4.683	2.349		
Meconium Adj.[c]	PWY.7400	Vocabulary	Caffeine	-1.737	2.896	0.545	0.974
Meconium Adj.[c]	PWY.7400	Vocabulary	Pathway	-180.543	378.161		
Meconium Adj.[c]	PWY.7400	Vocabulary	Interaction	1.083	1.073		
Meconium Adj.[c]	PWY.7400	WISC sum	Caffeine	-3.712	6.474	0.62	0.974
Meconium Adj.[c]	PWY.7400	WISC sum	Pathway	-248.358	845.357		
Meconium Adj.[c]	PWY.7400	WISC sum	Interaction	2.999	2.398		
Meconium Adj.[c]	TRNA.CHARGING.PWY	Block Design	Caffeine	0.827	4.033	0.76	0.974
Meconium Adj.[c]	TRNA.CHARGING.PWY	Block Design	Pathway	104.281	577.1		
Meconium Adj.[c]	TRNA.CHARGING.PWY	Block Design	Interaction	0.255	0.942		
Meconium Adj.[c]	TRNA.CHARGING.PWY	Coding	Caffeine	-0.454	3.502	0.864	0.974
Meconium Adj.[c]	TRNA.CHARGING.PWY	Coding	Pathway	42.723	501.134		
Meconium Adj.[c]	TRNA.CHARGING.PWY	Coding	Interaction	0.654	0.818		
Meconium Adj.[c]	TRNA.CHARGING.PWY	Digit span	Caffeine	-1.818	2.902	0.57	0.974
Meconium Adj.[c]	TRNA.CHARGING.PWY	Digit span	Pathway	-78.338	415.321		
Meconium Adj.[c]	TRNA.CHARGING.PWY	Digit span	Interaction	-0.06	0.678		
Meconium Adj.[c]	TRNA.CHARGING.PWY	Information	Caffeine	-2.036	2.962	0.477	0.974
Meconium Adj.[c]	TRNA.CHARGING.PWY	Information	Pathway	-207.951	423.953		

Exposure Window	Pathway	Outcome	Variable	Effect Estimate[a]	Std. Error	LRT[b]	
						p-value	q-value
Meconium Adj.[c]	TRNA.CHARGING.PWY	Information	Interaction	1.034	0.692		
Meconium Adj.[c]	TRNA.CHARGING.PWY	QTAC	Caffeine	-13.108	10.154	0.183	0.922
Meconium Adj.[c]	TRNA.CHARGING.PWY	QTAC	Pathway	-1510.099	1453.175		
Meconium Adj.[c]	TRNA.CHARGING.PWY	QTAC	Interaction	-4.266	2.371		
Meconium Adj.[c]	TRNA.CHARGING.PWY	Vocabulary	Caffeine	-1.272	4.562	0.786	0.974
Meconium Adj.[c]	TRNA.CHARGING.PWY	Vocabulary	Pathway	-92.674	652.923		
Meconium Adj.[c]	TRNA.CHARGING.PWY	Vocabulary	Interaction	1.113	1.065		
Meconium Adj.[c]	TRNA.CHARGING.PWY	WISC sum	Caffeine	-4.753	10.088	0.665	0.974
Meconium Adj.[c]	TRNA.CHARGING.PWY	WISC sum	Pathway	-231.959	1443.66		
Meconium Adj.[c]	TRNA.CHARGING.PWY	WISC sum	Interaction	2.995	2.356		
Meconium Adj.[c]	UNINTEGRATED	Block Design	Caffeine	10.161	7.842	0.144	0.922
Meconium Adj.[c]	UNINTEGRATED	Block Design	Pathway	1.285	0.923		
Meconium Adj.[c]	UNINTEGRATED	Block Design	Interaction	0.363	0.921		
Meconium Adj.[c]	UNINTEGRATED	Coding	Caffeine	4.893	7.024	0.451	0.974
Meconium Adj.[c]	UNINTEGRATED	Coding	Pathway	0.464	0.826		
Meconium Adj.[c]	UNINTEGRATED	Coding	Interaction	0.623	0.825		
Meconium Adj.[c]	UNINTEGRATED	Digit span	Caffeine	-1.861	5.961	0.774	0.974
Meconium Adj.[c]	UNINTEGRATED	Digit span	Pathway	-0.121	0.701		
Meconium Adj.[c]	UNINTEGRATED	Digit span	Interaction	-0.136	0.7		
Meconium Adj.[c]	UNINTEGRATED	Information	Caffeine	-5.963	5.79	0.267	0.923
Meconium Adj.[c]	UNINTEGRATED	Information	Pathway	-0.831	0.681		
Meconium Adj.[c]	UNINTEGRATED	Information	Interaction	0.928	0.68		
Meconium Adj.[c]	UNINTEGRATED	QTAC	Caffeine	24.754	20.239	0.167	0.922
Meconium Adj.[c]	UNINTEGRATED	QTAC	Pathway	2.734	2.382		
Meconium Adj.[c]	UNINTEGRATED	QTAC	Interaction	-4.441	2.376		
Meconium Adj.[c]	UNINTEGRATED	Vocabulary	Caffeine	1.349	9.087	0.855	0.974
Meconium Adj.[c]	UNINTEGRATED	Vocabulary	Pathway	0.161	1.069		
Meconium Adj.[c]	UNINTEGRATED	Vocabulary	Interaction	1.078	1.067		
Meconium Adj.[c]	UNINTEGRATED	WISC sum	Caffeine	8.58	20.336	0.611	0.974
Meconium Adj.[c]	UNINTEGRATED	WISC sum	Pathway	0.959	2.393		
Meconium Adj.[c]	UNINTEGRATED	WISC sum	Interaction	2.856	2.388		
Meconium Adj.[c]	UNMAPPED	Block Design	Caffeine	-3.606	2.34	0.12	0.922
Meconium Adj.[c]	UNMAPPED	Block Design	Pathway	-1.291	0.87		
Meconium Adj.[c]	UNMAPPED	Block Design	Interaction	0.357	0.918		
Meconium Adj.[c]	UNMAPPED	Coding	Caffeine	-1.19	2.106	0.497	0.974
Meconium Adj.[c]	UNMAPPED	Coding	Pathway	-0.397	0.783		

Exposure Window	Pathway	Outcome	Variable	Effect Estimate[a]	Std. Error	LRT[b]	
						p-value	q-value
Meconium Adj.[c]	UNMAPPED	Coding	Interaction	0.626	0.826		
Meconium Adj.[c]	UNMAPPED	Digit span	Caffeine	0.364	1.782		
Meconium Adj.[c]	UNMAPPED	Digit span	Pathway	0.188	0.663	0.672	0.974
Meconium Adj.[c]	UNMAPPED	Digit span	Interaction	-0.143	0.699		
Meconium Adj.[c]	UNMAPPED	Information	Caffeine	1.422	1.737		
Meconium Adj.[c]	UNMAPPED	Information	Pathway	0.715	0.646	0.326	0.974
Meconium Adj.[c]	UNMAPPED	Information	Interaction	0.946	0.681		
Meconium Adj.[c]	UNMAPPED	QTAC	Caffeine	-6.62	6.115		
Meconium Adj.[c]	UNMAPPED	QTAC	Pathway	-2.018	2.274	0.29	0.963
Meconium Adj.[c]	UNMAPPED	QTAC	Interaction	-4.465	2.398		
Meconium Adj.[c]	UNMAPPED	Vocabulary	Caffeine	-0.553	2.72		
Meconium Adj.[c]	UNMAPPED	Vocabulary	Pathway	-0.124	1.011	0.868	0.974
Meconium Adj.[c]	UNMAPPED	Vocabulary	Interaction	1.077	1.066		
Meconium Adj.[c]	UNMAPPED	WISC sum	Caffeine	-3.562	6.088		
Meconium Adj.[c]	UNMAPPED	WISC sum	Pathway	-0.91	2.264	0.611	0.974
Meconium Adj.[c]	UNMAPPED	WISC sum	Interaction	2.862	2.387		
Meconium Adj.[c]	VALSYN.PWY	Block Design	Caffeine	4.351	2.687		
Meconium Adj.[c]	VALSYN.PWY	Block Design	Pathway	514.528	312.467	0.06	0.868
Meconium Adj.[c]	VALSYN.PWY	Block Design	Interaction	0.592	0.952		
Meconium Adj.[c]	VALSYN.PWY	Coding	Caffeine	1.294	2.454		
Meconium Adj.[c]	VALSYN.PWY	Coding	Pathway	131.428	285.34	0.589	0.974
Meconium Adj.[c]	VALSYN.PWY	Coding	Interaction	0.677	0.869		
Meconium Adj.[c]	VALSYN.PWY	Digit span	Caffeine	-2.97	1.963		
Meconium Adj.[c]	VALSYN.PWY	Digit span	Pathway	-235.247	228.316	0.139	0.922
Meconium Adj.[c]	VALSYN.PWY	Digit span	Interaction	-0.095	0.695		
Meconium Adj.[c]	VALSYN.PWY	Information	Caffeine	-2.241	1.984		
Meconium Adj.[c]	VALSYN.PWY	Information	Pathway	-304.853	230.778	0.237	0.923
Meconium Adj.[c]	VALSYN.PWY	Information	Interaction	0.595	0.703		
Meconium Adj.[c]	VALSYN.PWY	QTAC	Caffeine	-12.252	6.933		
Meconium Adj.[c]	VALSYN.PWY	QTAC	Pathway	-1258.574	806.258	0.071	0.868
Meconium Adj.[c]	VALSYN.PWY	QTAC	Interaction	-5.264	2.456		
Meconium Adj.[c]	VALSYN.PWY	Vocabulary	Caffeine	-2.071	3.125		
Meconium Adj.[c]	VALSYN.PWY	Vocabulary	Pathway	-263.933	363.437	0.494	0.974
Meconium Adj.[c]	VALSYN.PWY	Vocabulary	Interaction	0.767	1.107		
Meconium Adj.[c]	VALSYN.PWY	WISC sum	Caffeine	-1.637	7.071	0.892	0.974
Meconium Adj.[c]	VALSYN.PWY	WISC sum	Pathway	-158.077	822.293		

Exposure Window	Pathway	Outcome	Variable	Effect Estimate[a]	Std. Error	LRT[b]	
						p-value	q-value
Meconium Adj.[c]	VALSYN.PWY	WISC sum	Interaction	2.536	2.504		

[a] Models are adjusted for whether the child was ever breastfed, sex, mode of birth, and socioeconomic status. Effect estimates for caffeine are the change in outcome score for each doubling of exposure. Effect estimates for pathway are the change in outcome score for each 1% increase in pathway relative abundance. Effect estimates for the interaction are for the multiplicative interaction between acetaminophen and pathway relative abundance.
[b] Likelihood ratio test comparing to model without interaction
[c] Meconium Adj. includes cross-sectional exposure in the model

Table S13. Acetaminophen-pathway interactions in association with neurodevelopment

Exposure Window	Pathway	Outcome	Variable	Effect Estimate[a]	Std. Error	LRT[b]	
						p-value	q-value
Meconium	ARGSYN.PWY	Block Design	Acetaminophen	-5.834	9.511	0.442	0.851
Meconium	ARGSYN.PWY	Block Design	Pathway	-79.257	99.458		
Meconium	ARGSYN.PWY	Block Design	Interaction	98.79	139.898		
Meconium	ARGSYN.PWY	Coding	Acetaminophen	-7.513	8.298	0.296	0.718
Meconium	ARGSYN.PWY	Coding	Pathway	-20.842	86.779		
Meconium	ARGSYN.PWY	Coding	Interaction	117.269	122.064		
Meconium	ARGSYN.PWY	Digit span	Acetaminophen	-12.489	6.862	0.051	0.68
Meconium	ARGSYN.PWY	Digit span	Pathway	-13.828	71.76		
Meconium	ARGSYN.PWY	Digit span	Interaction	183.446	100.938		
Meconium	ARGSYN.PWY	Information	Acetaminophen	-4.855	7.188	0.478	0.857
Meconium	ARGSYN.PWY	Information	Pathway	-59.571	75.164		
Meconium	ARGSYN.PWY	Information	Interaction	68.866	105.727		
Meconium	ARGSYN.PWY	QTAC	Acetaminophen	-31.534	25.065	0.219	0.708
Meconium	ARGSYN.PWY	QTAC	Pathway	-179.349	262.108		
Meconium	ARGSYN.PWY	QTAC	Interaction	417.815	368.683		
Meconium	ARGSYN.PWY	Vocabulary	Acetaminophen	-13.815	10.675	0.149	0.68
Meconium	ARGSYN.PWY	Vocabulary	Pathway	-99.26	111.635		
Meconium	ARGSYN.PWY	Vocabulary	Interaction	209.311	157.027		
Meconium	ARGSYN.PWY	WISC sum	Acetaminophen	-44.507	23.861	0.039	0.68
Meconium	ARGSYN.PWY	WISC sum	Pathway	-272.758	249.525		
Meconium	ARGSYN.PWY	WISC sum	Interaction	677.681	350.984		
Meconium	ARGSYNBSUB.PWY	Block Design	Acetaminophen	-6.112	9.418	0.419	0.851
Meconium	ARGSYNBSUB.PWY	Block Design	Pathway	-76.541	92.758		
Meconium	ARGSYNBSUB.PWY	Block Design	Interaction	98.927	133.269		
Meconium	ARGSYNBSUB.PWY	Coding	Acetaminophen	-5.465	8.272	0.436	0.851
Meconium	ARGSYNBSUB.PWY	Coding	Pathway	-8.031	81.477		
Meconium	ARGSYNBSUB.PWY	Coding	Interaction	83.678	117.06		
Meconium	ARGSYNBSUB.PWY	Digit span	Acetaminophen	-12.571	6.82	0.048	0.68
Meconium	ARGSYNBSUB.PWY	Digit span	Pathway	-17.961	67.173		
Meconium	ARGSYNBSUB.PWY	Digit span	Interaction	177.699	96.509		
Meconium	ARGSYNBSUB.PWY	Information	Acetaminophen	-4.559	7.124	0.503	0.857
Meconium	ARGSYNBSUB.PWY	Information	Pathway	-54.75	70.169		
Meconium	ARGSYNBSUB.PWY	Information	Interaction	61.944	100.814		
Meconium	ARGSYNBSUB.PWY	QTAC	Acetaminophen	-28.348	24.931	0.274	0.718
Meconium	ARGSYNBSUB.PWY	QTAC	Pathway	-181.882	245.559		

Exposure Window	Pathway	Outcome	Variable	Effect Estimate[a]	Std. Error	LRT[b] p-value	LRT[b] q-value
Meconium	ARGSYNBSUB.PWY	QTAC	Interaction	355.395	352.803		
Meconium	ARGSYNBSUB.PWY	Vocabulary	Acetaminophen	-13.638	10.582	0.151	0.68
Meconium	ARGSYNBSUB.PWY	Vocabulary	Pathway	-97.699	104.229		
Meconium	ARGSYNBSUB.PWY	Vocabulary	Interaction	198.66	149.749		
Meconium	ARGSYNBSUB.PWY	WISC sum	Acetaminophen	-42.345	23.752	0.048	0.68
Meconium	ARGSYNBSUB.PWY	WISC sum	Pathway	-254.982	233.943		
Meconium	ARGSYNBSUB.PWY	WISC sum	Interaction	620.907	336.113		
Meconium	ARO.PWY	Block Design	Acetaminophen	-9.369	10.262	0.279	0.718
Meconium	ARO.PWY	Block Design	Pathway	-112.229	97.361		
Meconium	ARO.PWY	Block Design	Interaction	150.462	150.887		
Meconium	ARO.PWY	Coding	Acetaminophen	-0.597	9.099	0.9	0.974
Meconium	ARO.PWY	Coding	Pathway	46.642	86.325		
Meconium	ARO.PWY	Coding	Interaction	15.315	133.784		
Meconium	ARO.PWY	Digit span	Acetaminophen	-8.619	7.797	0.236	0.718
Meconium	ARO.PWY	Digit span	Pathway	-0.863	73.98		
Meconium	ARO.PWY	Digit span	Interaction	125.076	114.652		
Meconium	ARO.PWY	Information	Acetaminophen	1.622	7.836	0.797	0.913
Meconium	ARO.PWY	Information	Pathway	-22.062	74.347		
Meconium	ARO.PWY	Information	Interaction	-27.103	115.222		
Meconium	ARO.PWY	QTAC	Acetaminophen	-33.711	27.262	0.227	0.718
Meconium	ARO.PWY	QTAC	Pathway	-276.276	258.653		
Meconium	ARO.PWY	QTAC	Interaction	445.948	400.852		
Meconium	ARO.PWY	Vocabulary	Acetaminophen	-10.159	11.727	0.332	0.761
Meconium	ARO.PWY	Vocabulary	Pathway	-107.115	111.259		
Meconium	ARO.PWY	Vocabulary	Interaction	153.634	172.427		
Meconium	ARO.PWY	WISC sum	Acetaminophen	-27.123	26.838	0.251	0.718
Meconium	ARO.PWY	WISC sum	Pathway	-195.627	254.638		
Meconium	ARO.PWY	WISC sum	Interaction	417.385	394.631		
Meconium	BRANCHED.CHAIN.AA.SYN.PWY	Block Design	Acetaminophen	2.24	8.952	0.86	0.963
Meconium	BRANCHED.CHAIN.AA.SYN.PWY	Block Design	Pathway	-22.353	102.342		
Meconium	BRANCHED.CHAIN.AA.SYN.PWY	Block Design	Interaction	-22.524	139.799		
Meconium	BRANCHED.CHAIN.AA.SYN.PWY	Coding	Acetaminophen	0.286	7.896	0.997	0.997
Meconium	BRANCHED.CHAIN.AA.SYN.PWY	Coding	Pathway	-21.811	90.275		
Meconium	BRANCHED.CHAIN.AA.SYN.PWY	Coding	Interaction	-0.371	123.315		
Meconium	BRANCHED.CHAIN.AA.SYN.PWY	Digit span	Acetaminophen	2.811	6.883	0.629	0.86
Meconium	BRANCHED.CHAIN.AA.SYN.PWY	Digit span	Pathway	40.001	78.693		

Exposure Window	Pathway	Outcome	Variable	Effect Estimate[a]	Std. Error	LRT[b]	
						p-value	q-value
Meconium	BRANCHED.CHAIN.AA.SYN.PWY	Digit span	Interaction	-47.6	107.494		
Meconium	BRANCHED.CHAIN.AA.SYN.PWY	Information	Acetaminophen	-3.129	6.565	0.647	0.86
Meconium	BRANCHED.CHAIN.AA.SYN.PWY	Information	Pathway	-103.931	75.059		
Meconium	BRANCHED.CHAIN.AA.SYN.PWY	Information	Interaction	43.043	102.53		
Meconium	BRANCHED.CHAIN.AA.SYN.PWY	QTAC	Acetaminophen	-1.81	23.858	0.94	0.983
Meconium	BRANCHED.CHAIN.AA.SYN.PWY	QTAC	Pathway	-12.714	272.768		
Meconium	BRANCHED.CHAIN.AA.SYN.PWY	QTAC	Interaction	-25.526	372.598		
Meconium	BRANCHED.CHAIN.AA.SYN.PWY	Vocabulary	Acetaminophen	-14.091	9.604	0.113	0.68
Meconium	BRANCHED.CHAIN.AA.SYN.PWY	Vocabulary	Pathway	-252.1	109.796		
Meconium	BRANCHED.CHAIN.AA.SYN.PWY	Vocabulary	Interaction	220.056	149.98		
Meconium	BRANCHED.CHAIN.AA.SYN.PWY	WISC sum	Acetaminophen	-11.883	22.736	0.554	0.857
Meconium	BRANCHED.CHAIN.AA.SYN.PWY	WISC sum	Pathway	-360.195	259.941		
Meconium	BRANCHED.CHAIN.AA.SYN.PWY	WISC sum	Interaction	192.604	355.077		
Meconium	CALVIN.PWY	Block Design	Acetaminophen	-6.054	6.753	0.26	0.718
Meconium	CALVIN.PWY	Block Design	Pathway	-76.387	83.358		
Meconium	CALVIN.PWY	Block Design	Interaction	119.839	115.525		
Meconium	CALVIN.PWY	Coding	Acetaminophen	2.76	6.015	0.656	0.863
Meconium	CALVIN.PWY	Coding	Pathway	19.345	74.247		
Meconium	CALVIN.PWY	Coding	Interaction	-42.024	102.899		
Meconium	CALVIN.PWY	Digit span	Acetaminophen	-5.506	5.123	0.255	0.718
Meconium	CALVIN.PWY	Digit span	Pathway	-0.374	63.239		
Meconium	CALVIN.PWY	Digit span	Interaction	91.851	87.643		
Meconium	CALVIN.PWY	Information	Acetaminophen	3.376	5.139	0.449	0.851
Meconium	CALVIN.PWY	Information	Pathway	21.589	63.431		
Meconium	CALVIN.PWY	Information	Interaction	-61.03	87.909		
Meconium	CALVIN.PWY	QTAC	Acetaminophen	-21.918	17.83	0.259	0.718
Meconium	CALVIN.PWY	QTAC	Pathway	-280.613	220.096		
Meconium	CALVIN.PWY	QTAC	Interaction	317.155	305.03		
Meconium	CALVIN.PWY	Vocabulary	Acetaminophen	-2.664	7.77	0.674	0.863
Meconium	CALVIN.PWY	Vocabulary	Pathway	-40.697	95.909		
Meconium	CALVIN.PWY	Vocabulary	Interaction	51.157	132.92		
Meconium	CALVIN.PWY	WISC sum	Acetaminophen	-8.087	17.787	0.567	0.857
Meconium	CALVIN.PWY	WISC sum	Pathway	-76.524	219.561		
Meconium	CALVIN.PWY	WISC sum	Interaction	159.792	304.288		
Meconium	COA.PWY.1	Block Design	Acetaminophen	-15.657	16.71	0.282	0.718
Meconium	COA.PWY.1	Block Design	Pathway	-97.412	137.209		

Exposure Window	Pathway	Outcome	Variable	Effect Estimate[a]	Std. Error	LRT[b]	
						p-value	q-value
Meconium	COA.PWY.1	Block Design	Interaction	274.994	277.596		
Meconium	COA.PWY.1	Coding	Acetaminophen	-18.27	14.494	0.161	0.68
Meconium	COA.PWY.1	Coding	Pathway	-7.423	119.011		
Meconium	COA.PWY.1	Coding	Interaction	311.86	240.778		
Meconium	COA.PWY.1	Digit span	Acetaminophen	-20.32	12.296	0.076	0.68
Meconium	COA.PWY.1	Digit span	Pathway	30.211	100.966		
Meconium	COA.PWY.1	Digit span	Interaction	337.488	204.27		
Meconium	COA.PWY.1	Information	Acetaminophen	-29.399	11.932	0.01	0.68
Meconium	COA.PWY.1	Information	Pathway	-141.581	97.978		
Meconium	COA.PWY.1	Information	Interaction	486.505	198.226		
Meconium	COA.PWY.1	QTAC	Acetaminophen	36.655	44.471	0.329	0.761
Meconium	COA.PWY.1	QTAC	Pathway	258.411	365.164		
Meconium	COA.PWY.1	QTAC	Interaction	-663.31	738.785		
Meconium	COA.PWY.1	Vocabulary	Acetaminophen	-24.708	18.772	0.151	0.68
Meconium	COA.PWY.1	Vocabulary	Pathway	-186.645	154.14		
Meconium	COA.PWY.1	Vocabulary	Interaction	414.214	311.85		
Meconium	COA.PWY.1	WISC sum	Acetaminophen	-108.354	40.567	0.005	0.68
Meconium	COA.PWY.1	WISC sum	Pathway	-402.85	333.105		
Meconium	COA.PWY.1	WISC sum	Interaction	1825.061	673.924		
Meconium	COMPLETE.ARO.PWY	Block Design	Acetaminophen	-6.75	9.397	0.375	0.8
Meconium	COMPLETE.ARO.PWY	Block Design	Pathway	-78.927	97.198		
Meconium	COMPLETE.ARO.PWY	Block Design	Interaction	120.716	148.147		
Meconium	COMPLETE.ARO.PWY	Coding	Acetaminophen	4.172	8.293	0.618	0.86
Meconium	COMPLETE.ARO.PWY	Coding	Pathway	63.527	85.786		
Meconium	COMPLETE.ARO.PWY	Coding	Interaction	-59.792	130.754		
Meconium	COMPLETE.ARO.PWY	Digit span	Acetaminophen	-7.578	7.076	0.252	0.718
Meconium	COMPLETE.ARO.PWY	Digit span	Pathway	3.65	73.191		
Meconium	COMPLETE.ARO.PWY	Digit span	Interaction	117.704	111.557		
Meconium	COMPLETE.ARO.PWY	Information	Acetaminophen	0.703	7.142	0.891	0.968
Meconium	COMPLETE.ARO.PWY	Information	Pathway	-18.381	73.881		
Meconium	COMPLETE.ARO.PWY	Information	Interaction	-14.162	112.609		
Meconium	COMPLETE.ARO.PWY	QTAC	Acetaminophen	-21.39	24.888	0.434	0.851
Meconium	COMPLETE.ARO.PWY	QTAC	Pathway	-259.858	257.442		
Meconium	COMPLETE.ARO.PWY	QTAC	Interaction	281.853	392.389		
Meconium	COMPLETE.ARO.PWY	Vocabulary	Acetaminophen	-12.939	10.571	0.174	0.68
Meconium	COMPLETE.ARO.PWY	Vocabulary	Pathway	-127.525	109.343		

Exposure Window	Pathway	Outcome	Variable	Effect Estimate[a]	Std. Error	LRT[b]	
						p-value	q-value
Meconium	COMPLETE.ARO.PWY	Vocabulary	Interaction	209.231	166.658		
Meconium	COMPLETE.ARO.PWY	WISC sum	Acetaminophen	-22.392	24.442	0.292	0.718
Meconium	COMPLETE.ARO.PWY	WISC sum	Pathway	-157.655	252.826		
Meconium	COMPLETE.ARO.PWY	WISC sum	Interaction	373.697	385.353		
Meconium	DTDPRHAMSYN.PWY	Block Design	Acetaminophen	1.539	5.638	0.877	0.965
Meconium	DTDPRHAMSYN.PWY	Block Design	Pathway	-61.178	64.309		
Meconium	DTDPRHAMSYN.PWY	Block Design	Interaction	-12.932	91.184		
Meconium	DTDPRHAMSYN.PWY	Coding	Acetaminophen	12.405	4.688	0.006	0.68
Meconium	DTDPRHAMSYN.PWY	Coding	Pathway	70.971	53.48		
Meconium	DTDPRHAMSYN.PWY	Coding	Interaction	-198.676	75.83		
Meconium	DTDPRHAMSYN.PWY	Digit span	Acetaminophen	0.872	4.429	0.772	0.906
Meconium	DTDPRHAMSYN.PWY	Digit span	Pathway	-1.099	50.523		
Meconium	DTDPRHAMSYN.PWY	Digit span	Interaction	-18.988	71.638		
Meconium	DTDPRHAMSYN.PWY	Information	Acetaminophen	4.856	4.273	0.195	0.702
Meconium	DTDPRHAMSYN.PWY	Information	Pathway	19.939	48.745		
Meconium	DTDPRHAMSYN.PWY	Information	Interaction	-82.637	69.116		
Meconium	DTDPRHAMSYN.PWY	QTAC	Acetaminophen	-8.504	15.27	0.717	0.873
Meconium	DTDPRHAMSYN.PWY	QTAC	Pathway	-101.156	174.188		
Meconium	DTDPRHAMSYN.PWY	QTAC	Interaction	81.884	246.983		
Meconium	DTDPRHAMSYN.PWY	Vocabulary	Acetaminophen	4.774	6.525	0.449	0.851
Meconium	DTDPRHAMSYN.PWY	Vocabulary	Pathway	24.256	74.436		
Meconium	DTDPRHAMSYN.PWY	Vocabulary	Interaction	-73.308	105.543		
Meconium	DTDPRHAMSYN.PWY	WISC sum	Acetaminophen	24.447	14.387	0.074	0.68
Meconium	DTDPRHAMSYN.PWY	WISC sum	Pathway	52.889	164.111		
Meconium	DTDPRHAMSYN.PWY	WISC sum	Interaction	-386.54	232.695		
Meconium	GLUTORN.PWY	Block Design	Acetaminophen	-5.552	7.832	0.369	0.8
Meconium	GLUTORN.PWY	Block Design	Pathway	-72.406	91.432		
Meconium	GLUTORN.PWY	Block Design	Interaction	104.391	126.431		
Meconium	GLUTORN.PWY	Coding	Acetaminophen	-3.527	6.891	0.528	0.857
Meconium	GLUTORN.PWY	Coding	Pathway	0.005	80.453		
Meconium	GLUTORN.PWY	Coding	Interaction	64.294	111.249		
Meconium	GLUTORN.PWY	Digit span	Acetaminophen	-9.329	5.806	0.085	0.68
Meconium	GLUTORN.PWY	Digit span	Pathway	-31.557	67.778		
Meconium	GLUTORN.PWY	Digit span	Interaction	149.68	93.722		
Meconium	GLUTORN.PWY	Information	Acetaminophen	-2.837	5.909	0.63	0.86
Meconium	GLUTORN.PWY	Information	Pathway	-59.422	68.99		

Exposure Window	Pathway	Outcome	Variable	Effect Estimate[a]	Std. Error	LRT[b] p-value	LRT[b] q-value
Meconium	GLUTORN.PWY	Information	Interaction	42.139	95.398		
Meconium	GLUTORN.PWY	QTAC	Acetaminophen	-30.595	20.526	0.146	0.68
Meconium	GLUTORN.PWY	QTAC	Pathway	-197.941	239.629		
Meconium	GLUTORN.PWY	QTAC	Interaction	444.928	331.355		
Meconium	GLUTORN.PWY	Vocabulary	Acetaminophen	-11.053	8.808	0.159	0.68
Meconium	GLUTORN.PWY	Vocabulary	Pathway	-93.97	102.832		
Meconium	GLUTORN.PWY	Vocabulary	Interaction	185.291	142.194		
Meconium	GLUTORN.PWY	WISC sum	Acetaminophen	-32.298	19.894	0.068	0.68
Meconium	GLUTORN.PWY	WISC sum	Pathway	-257.35	232.256		
Meconium	GLUTORN.PWY	WISC sum	Interaction	545.795	321.16		
Meconium	GLYCOGENSYNTH.PWY	Block Design	Acetaminophen	-1.759	5.871	0.617	0.86
Meconium	GLYCOGENSYNTH.PWY	Block Design	Pathway	-38.179	76.19		
Meconium	GLYCOGENSYNTH.PWY	Block Design	Interaction	43.221	94.366		
Meconium	GLYCOGENSYNTH.PWY	Coding	Acetaminophen	2.757	5.14	0.596	0.86
Meconium	GLYCOGENSYNTH.PWY	Coding	Pathway	-3.332	66.698		
Meconium	GLYCOGENSYNTH.PWY	Coding	Interaction	-40.103	82.61		
Meconium	GLYCOGENSYNTH.PWY	Digit span	Acetaminophen	-5.819	4.421	0.166	0.68
Meconium	GLYCOGENSYNTH.PWY	Digit span	Pathway	-37.009	57.377		
Meconium	GLYCOGENSYNTH.PWY	Digit span	Interaction	90.837	71.066		
Meconium	GLYCOGENSYNTH.PWY	Information	Acetaminophen	5.271	4.364	0.173	0.68
Meconium	GLYCOGENSYNTH.PWY	Information	Pathway	49.651	56.638		
Meconium	GLYCOGENSYNTH.PWY	Information	Interaction	-88.212	70.15		
Meconium	GLYCOGENSYNTH.PWY	QTAC	Acetaminophen	-20.695	15.197	0.215	0.708
Meconium	GLYCOGENSYNTH.PWY	QTAC	Pathway	-308.064	197.219		
Meconium	GLYCOGENSYNTH.PWY	QTAC	Interaction	279.338	244.269		
Meconium	GLYCOGENSYNTH.PWY	Vocabulary	Acetaminophen	1.502	6.696	0.844	0.958
Meconium	GLYCOGENSYNTH.PWY	Vocabulary	Pathway	5.024	86.898		
Meconium	GLYCOGENSYNTH.PWY	Vocabulary	Interaction	-19.337	107.629		
Meconium	GLYCOGENSYNTH.PWY	WISC sum	Acetaminophen	1.952	15.341	0.952	0.991
Meconium	GLYCOGENSYNTH.PWY	WISC sum	Pathway	-23.846	199.079		
Meconium	GLYCOGENSYNTH.PWY	WISC sum	Interaction	-13.594	246.572		
Meconium	ILEUSYN.PWY	Block Design	Acetaminophen	-4.98	9.327	0.492	0.857
Meconium	ILEUSYN.PWY	Block Design	Pathway	-53.816	89.422		
Meconium	ILEUSYN.PWY	Block Design	Interaction	76.887	121.977		
Meconium	ILEUSYN.PWY	Coding	Acetaminophen	-0.313	8.252	0.935	0.982
Meconium	ILEUSYN.PWY	Coding	Pathway	-12.143	79.115		

Exposure Window	Pathway	Outcome	Variable	Effect Estimate[a]	Std. Error	LRT[b]	
						p-value	q-value
Meconium	ILEUSYN.PWY	Coding	Interaction	8.044	107.918		
Meconium	ILEUSYN.PWY	Digit span	Acetaminophen	-3.135	7.002	0.623	0.86
Meconium	ILEUSYN.PWY	Digit span	Pathway	40.972	67.128		
Meconium	ILEUSYN.PWY	Digit span	Interaction	41.172	91.566		
Meconium	ILEUSYN.PWY	Information	Acetaminophen	-2.968	6.787	0.685	0.863
Meconium	ILEUSYN.PWY	Information	Pathway	-96.617	65.072		
Meconium	ILEUSYN.PWY	Information	Interaction	32.979	88.762		
Meconium	ILEUSYN.PWY	QTAC	Acetaminophen	-16.668	24.772	0.548	0.857
Meconium	ILEUSYN.PWY	QTAC	Pathway	-41.349	237.496		
Meconium	ILEUSYN.PWY	QTAC	Interaction	178.14	323.959		
Meconium	ILEUSYN.PWY	Vocabulary	Acetaminophen	-18.001	10.191	0.055	0.68
Meconium	ILEUSYN.PWY	Vocabulary	Pathway	-187.546	97.698		
Meconium	ILEUSYN.PWY	Vocabulary	Interaction	238.009	133.266		
Meconium	ILEUSYN.PWY	WISC sum	Acetaminophen	-29.398	23.88	0.169	0.68
Meconium	ILEUSYN.PWY	WISC sum	Pathway	-309.15	228.944		
Meconium	ILEUSYN.PWY	WISC sum	Interaction	397.091	312.294		
Meconium	PEPTIDOGLYCANSYN.PWY	Block Design	Acetaminophen	-9.581	14.492	0.43	0.851
Meconium	PEPTIDOGLYCANSYN.PWY	Block Design	Pathway	-100.488	146.647		
Meconium	PEPTIDOGLYCANSYN.PWY	Block Design	Interaction	162.862	224.818		
Meconium	PEPTIDOGLYCANSYN.PWY	Coding	Acetaminophen	4.595	12.734	0.717	0.873
Meconium	PEPTIDOGLYCANSYN.PWY	Coding	Pathway	103.854	128.86		
Meconium	PEPTIDOGLYCANSYN.PWY	Coding	Interaction	-65.63	197.55		
Meconium	PEPTIDOGLYCANSYN.PWY	Digit span	Acetaminophen	-11.351	10.883	0.265	0.718
Meconium	PEPTIDOGLYCANSYN.PWY	Digit span	Pathway	18.673	110.13		
Meconium	PEPTIDOGLYCANSYN.PWY	Digit span	Interaction	173.237	168.836		
Meconium	PEPTIDOGLYCANSYN.PWY	Information	Acetaminophen	-10.584	10.853	0.293	0.718
Meconium	PEPTIDOGLYCANSYN.PWY	Information	Pathway	-24.777	109.825		
Meconium	PEPTIDOGLYCANSYN.PWY	Information	Interaction	162.741	168.368		
Meconium	PEPTIDOGLYCANSYN.PWY	QTAC	Acetaminophen	6.714	38.703	0.777	0.906
Meconium	PEPTIDOGLYCANSYN.PWY	QTAC	Pathway	152.109	391.654		
Meconium	PEPTIDOGLYCANSYN.PWY	QTAC	Interaction	-155.76	600.427		
Meconium	PEPTIDOGLYCANSYN.PWY	Vocabulary	Acetaminophen	-16.243	16.24	0.265	0.718
Meconium	PEPTIDOGLYCANSYN.PWY	Vocabulary	Pathway	-11.64	164.344		
Meconium	PEPTIDOGLYCANSYN.PWY	Vocabulary	Interaction	258.458	251.948		
Meconium	PEPTIDOGLYCANSYN.PWY	WISC sum	Acetaminophen	-43.164	36.848	0.19	0.694
Meconium	PEPTIDOGLYCANSYN.PWY	WISC sum	Pathway	-14.377	372.875		

Exposure Window	Pathway	Outcome	Variable	Effect Estimate[a]	Std. Error	LRT[b]	
						p-value	q-value
Meconium	PEPTIDOGLYCANSYN.PWY	WISC sum	Interaction	691.667	571.637		
Meconium	PWY.1042	Block Design	Acetaminophen	-9.121	8.871	0.219	0.708
Meconium	PWY.1042	Block Design	Pathway	-77.946	77.25		
Meconium	PWY.1042	Block Design	Interaction	131.214	115.855		
Meconium	PWY.1042	Coding	Acetaminophen	2.933	7.892	0.724	0.877
Meconium	PWY.1042	Coding	Pathway	47.335	68.722		
Meconium	PWY.1042	Coding	Interaction	-33.289	103.065		
Meconium	PWY.1042	Digit span	Acetaminophen	-5.211	6.852	0.423	0.851
Meconium	PWY.1042	Digit span	Pathway	-1.127	59.666		
Meconium	PWY.1042	Digit span	Interaction	65.761	89.483		
Meconium	PWY.1042	Information	Acetaminophen	6.388	6.706	0.283	0.718
Meconium	PWY.1042	Information	Pathway	13.581	58.391		
Meconium	PWY.1042	Information	Interaction	-86.447	87.572		
Meconium	PWY.1042	QTAC	Acetaminophen	-32.713	23.474	0.172	0.68
Meconium	PWY.1042	QTAC	Pathway	-122.054	204.405		
Meconium	PWY.1042	QTAC	Interaction	386.91	306.555		
Meconium	PWY.1042	Vocabulary	Acetaminophen	0.134	10.236	0.989	0.997
Meconium	PWY.1042	Vocabulary	Pathway	-30.338	89.129		
Meconium	PWY.1042	Vocabulary	Interaction	1.668	133.671		
Meconium	PWY.1042	WISC sum	Acetaminophen	-4.877	23.494	0.779	0.906
Meconium	PWY.1042	WISC sum	Pathway	-48.495	204.581		
Meconium	PWY.1042	WISC sum	Interaction	78.906	306.82		
Meconium	PWY.3841	Block Design	Acetaminophen	-10.051	13.86	0.388	0.816
Meconium	PWY.3841	Block Design	Pathway	-126.503	182.08		
Meconium	PWY.3841	Block Design	Interaction	206.87	261.183		
Meconium	PWY.3841	Coding	Acetaminophen	7.041	12.153	0.548	0.857
Meconium	PWY.3841	Coding	Pathway	157.075	159.654		
Meconium	PWY.3841	Coding	Interaction	-126.158	229.014		
Meconium	PWY.3841	Digit span	Acetaminophen	-13.465	10.065	0.153	0.68
Meconium	PWY.3841	Digit span	Pathway	63.816	132.225		
Meconium	PWY.3841	Digit span	Interaction	250.435	189.669		
Meconium	PWY.3841	Information	Acetaminophen	-19.904	9.916	0.033	0.68
Meconium	PWY.3841	Information	Pathway	-71.548	130.273		
Meconium	PWY.3841	Information	Interaction	373.842	186.868		
Meconium	PWY.3841	QTAC	Acetaminophen	31.008	36.712	0.307	0.738
Meconium	PWY.3841	QTAC	Pathway	256.64	482.288		

Exposure Window	Pathway	Outcome	Variable	Effect Estimate[a]	Std. Error	LRT[b]	
						p-value	q-value
Meconium	PWY.3841	QTAC	Interaction	-649.28	691.812		
Meconium	PWY.3841	Vocabulary	Acetaminophen	-21.115	15.534	0.135	0.68
Meconium	PWY.3841	Vocabulary	Pathway	-182.983	204.077		
Meconium	PWY.3841	Vocabulary	Interaction	404.952	292.735		
Meconium	PWY.3841	WISC sum	Acetaminophen	-57.493	34.654	0.068	0.68
Meconium	PWY.3841	WISC sum	Pathway	-160.143	455.257		
Meconium	PWY.3841	WISC sum	Interaction	1109.941	653.039		
Meconium	PWY.4242	Block Design	Acetaminophen	1.42	8.846	0.934	0.982
Meconium	PWY.4242	Block Design	Pathway	-59.514	100.323		
Meconium	PWY.4242	Block Design	Interaction	-12.921	170.412		
Meconium	PWY.4242	Coding	Acetaminophen	2.581	7.801	0.764	0.906
Meconium	PWY.4242	Coding	Pathway	63.445	88.471		
Meconium	PWY.4242	Coding	Interaction	-41.315	150.28		
Meconium	PWY.4242	Digit span	Acetaminophen	-4.503	6.657	0.464	0.857
Meconium	PWY.4242	Digit span	Pathway	50.439	75.497		
Meconium	PWY.4242	Digit span	Interaction	86.118	128.242		
Meconium	PWY.4242	Information	Acetaminophen	3.928	6.689	0.497	0.857
Meconium	PWY.4242	Information	Pathway	1.634	75.863		
Meconium	PWY.4242	Information	Interaction	-80.223	128.863		
Meconium	PWY.4242	QTAC	Acetaminophen	-22.194	23.505	0.378	0.8
Meconium	PWY.4242	QTAC	Pathway	-100.613	266.579		
Meconium	PWY.4242	QTAC	Interaction	366.771	452.821		
Meconium	PWY.4242	Vocabulary	Acetaminophen	-1.626	9.922	0.854	0.961
Meconium	PWY.4242	Vocabulary	Pathway	-130.107	112.531		
Meconium	PWY.4242	Vocabulary	Interaction	32.093	191.149		
Meconium	PWY.4242	WISC sum	Acetaminophen	1.8	23.214	0.968	0.996
Meconium	PWY.4242	WISC sum	Pathway	-74.103	263.279		
Meconium	PWY.4242	WISC sum	Interaction	-16.247	447.216		
Meconium	PWY.5097	Block Design	Acetaminophen	-10.775	11.292	0.258	0.718
Meconium	PWY.5097	Block Design	Pathway	-74.665	141.723		
Meconium	PWY.5097	Block Design	Interaction	206.951	198.832		
Meconium	PWY.5097	Coding	Acetaminophen	16.584	9.607	0.07	0.68
Meconium	PWY.5097	Coding	Pathway	244.456	120.574		
Meconium	PWY.5097	Coding	Interaction	-285.641	169.161		
Meconium	PWY.5097	Digit span	Acetaminophen	-8.138	8.302	0.293	0.718
Meconium	PWY.5097	Digit span	Pathway	71.237	104.196		

Exposure Window	Pathway	Outcome	Variable	Effect Estimate[a]	Std. Error	LRT[b]	
						p-value	q-value
Meconium	PWY.5097	Digit span	Interaction	141.338	146.182		
Meconium	PWY.5097	Information	Acetaminophen	-5.308	8.577	0.507	0.857
Meconium	PWY.5097	Information	Pathway	-2.607	107.644		
Meconium	PWY.5097	Information	Interaction	91.931	151.02		
Meconium	PWY.5097	QTAC	Acetaminophen	3.84	30.416	0.795	0.913
Meconium	PWY.5097	QTAC	Pathway	65.598	381.738		
Meconium	PWY.5097	QTAC	Interaction	-127.093	535.561		
Meconium	PWY.5097	Vocabulary	Acetaminophen	-12.383	12.881	0.282	0.718
Meconium	PWY.5097	Vocabulary	Pathway	-114.449	161.659		
Meconium	PWY.5097	Vocabulary	Interaction	224.485	226.8		
Meconium	PWY.5097	WISC sum	Acetaminophen	-20.019	29.087	0.42	0.851
Meconium	PWY.5097	WISC sum	Pathway	123.973	365.047		
Meconium	PWY.5097	WISC sum	Interaction	379.064	512.146		
Meconium	PWY.5103	Block Design	Acetaminophen	3.626	8.002	0.696	0.866
Meconium	PWY.5103	Block Design	Pathway	-14.925	93.248		
Meconium	PWY.5103	Block Design	Interaction	-46.13	129.087		
Meconium	PWY.5103	Coding	Acetaminophen	0.89	7.066	0.919	0.979
Meconium	PWY.5103	Coding	Pathway	-23	82.342		
Meconium	PWY.5103	Coding	Interaction	-10.581	113.989		
Meconium	PWY.5103	Digit span	Acetaminophen	3.461	6.162	0.507	0.857
Meconium	PWY.5103	Digit span	Pathway	32.805	71.805		
Meconium	PWY.5103	Digit span	Interaction	-60.436	99.403		
Meconium	PWY.5103	Information	Acetaminophen	-2.847	5.897	0.645	0.86
Meconium	PWY.5103	Information	Pathway	-94.324	68.715		
Meconium	PWY.5103	Information	Interaction	40.103	95.125		
Meconium	PWY.5103	QTAC	Acetaminophen	3.083	21.343	0.734	0.88
Meconium	PWY.5103	QTAC	Pathway	5.225	248.706		
Meconium	PWY.5103	QTAC	Interaction	-107.237	344.293		
Meconium	PWY.5103	Vocabulary	Acetaminophen	-11.673	8.581	0.142	0.68
Meconium	PWY.5103	Vocabulary	Pathway	-233.398	99.989		
Meconium	PWY.5103	Vocabulary	Interaction	187.863	138.419		
Meconium	PWY.5103	WISC sum	Acetaminophen	-6.543	20.253	0.711	0.873
Meconium	PWY.5103	WISC sum	Pathway	-332.843	236		
Meconium	PWY.5103	WISC sum	Interaction	110.819	326.703		
Meconium	PWY.5686	Block Design	Acetaminophen	2.008	12.25	0.921	0.979
Meconium	PWY.5686	Block Design	Pathway	-41.368	107.42		

Exposure Window	Pathway	Outcome	Variable	Effect Estimate[a]	Std. Error	LRT[b]	
						p-value	q-value
Meconium	PWY.5686	Block Design	Interaction	-16.604	182.867		
Meconium	PWY.5686	Coding	Acetaminophen	-5.571	10.772	0.548	0.857
Meconium	PWY.5686	Coding	Pathway	-6.475	94.461		
Meconium	PWY.5686	Coding	Interaction	88.426	160.806		
Meconium	PWY.5686	Digit span	Acetaminophen	-9.757	9.162	0.258	0.718
Meconium	PWY.5686	Digit span	Pathway	34.824	80.34		
Meconium	PWY.5686	Digit span	Interaction	142.477	136.768		
Meconium	PWY.5686	Information	Acetaminophen	0.798	9.281	0.911	0.977
Meconium	PWY.5686	Information	Pathway	-9.718	81.383		
Meconium	PWY.5686	Information	Interaction	-14.15	138.543		
Meconium	PWY.5686	QTAC	Acetaminophen	-17.017	32.468	0.646	0.86
Meconium	PWY.5686	QTAC	Pathway	-196.521	284.72		
Meconium	PWY.5686	QTAC	Interaction	203.977	484.695		
Meconium	PWY.5686	Vocabulary	Acetaminophen	-3.835	13.783	0.743	0.887
Meconium	PWY.5686	Vocabulary	Pathway	-126.059	120.862		
Meconium	PWY.5686	Vocabulary	Interaction	61.8	205.751		
Meconium	PWY.5686	WISC sum	Acetaminophen	-16.357	31.901	0.548	0.857
Meconium	PWY.5686	WISC sum	Pathway	-148.796	279.746		
Meconium	PWY.5686	WISC sum	Interaction	261.948	476.228		
Meconium	PWY.6121	Block Design	Acetaminophen	-7.161	9.257	0.345	0.776
Meconium	PWY.6121	Block Design	Pathway	-88.485	91.644		
Meconium	PWY.6121	Block Design	Interaction	133.393	153.719		
Meconium	PWY.6121	Coding	Acetaminophen	-2.552	8.234	0.698	0.866
Meconium	PWY.6121	Coding	Pathway	-2.612	81.512		
Meconium	PWY.6121	Coding	Interaction	48.649	136.724		
Meconium	PWY.6121	Digit span	Acetaminophen	-11.899	6.701	0.057	0.68
Meconium	PWY.6121	Digit span	Pathway	16.775	66.345		
Meconium	PWY.6121	Digit span	Interaction	197.402	111.283		
Meconium	PWY.6121	Information	Acetaminophen	-3.804	7.014	0.559	0.857
Meconium	PWY.6121	Information	Pathway	14.355	69.436		
Meconium	PWY.6121	Information	Interaction	62.435	116.469		
Meconium	PWY.6121	QTAC	Acetaminophen	-20.285	24.666	0.458	0.855
Meconium	PWY.6121	QTAC	Pathway	-213.238	244.196		
Meconium	PWY.6121	QTAC	Interaction	278.764	409.6		
Meconium	PWY.6121	Vocabulary	Acetaminophen	-12.255	10.467	0.189	0.694
Meconium	PWY.6121	Vocabulary	Pathway	-56.721	103.62		

Exposure Window	Pathway	Outcome	Variable	Effect Estimate[a]	Std. Error	LRT[b]	
						p-value	q-value
Meconium	PWY.6121	Vocabulary	Interaction	210.711	173.807		
Meconium	PWY.6121	WISC sum	Acetaminophen	-37.672	23.498	0.072	0.68
Meconium	PWY.6121	WISC sum	Pathway	-116.689	232.631		
Meconium	PWY.6121	WISC sum	Interaction	652.589	390.203		
Meconium	PWY.6122	Block Design	Acetaminophen	-9.772	10.434	0.268	0.718
Meconium	PWY.6122	Block Design	Pathway	-128.275	114.614		
Meconium	PWY.6122	Block Design	Interaction	164.891	161.672		
Meconium	PWY.6122	Coding	Acetaminophen	-4.206	9.301	0.591	0.86
Meconium	PWY.6122	Coding	Pathway	-23.525	102.164		
Meconium	PWY.6122	Coding	Interaction	71.016	144.11		
Meconium	PWY.6122	Digit span	Acetaminophen	-10.893	7.616	0.122	0.68
Meconium	PWY.6122	Digit span	Pathway	17.433	83.664		
Meconium	PWY.6122	Digit span	Interaction	168.817	118.014		
Meconium	PWY.6122	Information	Acetaminophen	-4.242	7.957	0.567	0.857
Meconium	PWY.6122	Information	Pathway	-5.242	87.402		
Meconium	PWY.6122	Information	Interaction	64.703	123.288		
Meconium	PWY.6122	QTAC	Acetaminophen	-10.512	28.114	0.784	0.906
Meconium	PWY.6122	QTAC	Pathway	-116.436	308.818		
Meconium	PWY.6122	QTAC	Interaction	109.467	435.612		
Meconium	PWY.6122	Vocabulary	Acetaminophen	-14.632	11.766	0.164	0.68
Meconium	PWY.6122	Vocabulary	Pathway	-70.755	129.247		
Meconium	PWY.6122	Vocabulary	Interaction	234.384	182.313		
Meconium	PWY.6122	WISC sum	Acetaminophen	-43.745	26.464	0.065	0.68
Meconium	PWY.6122	WISC sum	Pathway	-210.364	290.693		
Meconium	PWY.6122	WISC sum	Interaction	703.811	410.046		
Meconium	PWY.6151	Block Design	Acetaminophen	-6.651	8.486	0.33	0.761
Meconium	PWY.6151	Block Design	Pathway	-83.929	94.961		
Meconium	PWY.6151	Block Design	Interaction	126.687	141.557		
Meconium	PWY.6151	Coding	Acetaminophen	7.477	7.435	0.294	0.718
Meconium	PWY.6151	Coding	Pathway	94.462	83.196		
Meconium	PWY.6151	Coding	Interaction	-119.604	124.02		
Meconium	PWY.6151	Digit span	Acetaminophen	-7.651	6.47	0.212	0.708
Meconium	PWY.6151	Digit span	Pathway	-32.139	72.401		
Meconium	PWY.6151	Digit span	Interaction	124.153	107.927		
Meconium	PWY.6151	Information	Acetaminophen	1.024	6.407	0.838	0.956
Meconium	PWY.6151	Information	Pathway	-39.14	71.698		

Exposure Window	Pathway	Outcome	Variable	Effect Estimate[a]	Std. Error	LRT[b]	
						p-value	q-value
Meconium	PWY.6151	Information	Interaction	-19.97	106.879		
Meconium	PWY.6151	QTAC	Acetaminophen	-3.606	22.796	0.99	0.997
Meconium	PWY.6151	QTAC	Pathway	-11.222	255.093		
Meconium	PWY.6151	QTAC	Interaction	4.158	380.264		
Meconium	PWY.6151	Vocabulary	Acetaminophen	-5.87	9.67	0.484	0.857
Meconium	PWY.6151	Vocabulary	Pathway	-95.808	108.21		
Meconium	PWY.6151	Vocabulary	Interaction	103.527	161.307		
Meconium	PWY.6151	WISC sum	Acetaminophen	-11.67	22.255	0.528	0.857
Meconium	PWY.6151	WISC sum	Pathway	-156.553	249.032		
Meconium	PWY.6151	WISC sum	Interaction	214.794	371.229		
Meconium	PWY.6163	Block Design	Acetaminophen	-11.652	9.37	0.148	0.68
Meconium	PWY.6163	Block Design	Pathway	-124.842	89.402		
Meconium	PWY.6163	Block Design	Interaction	183.051	136.7		
Meconium	PWY.6163	Coding	Acetaminophen	3.303	8.366	0.706	0.872
Meconium	PWY.6163	Coding	Pathway	70.222	79.816		
Meconium	PWY.6163	Coding	Interaction	-42.102	122.043		
Meconium	PWY.6163	Digit span	Acetaminophen	-9.057	7.178	0.178	0.68
Meconium	PWY.6163	Digit span	Pathway	-18.635	68.491		
Meconium	PWY.6163	Digit span	Interaction	130.191	104.726		
Meconium	PWY.6163	Information	Acetaminophen	-0.288	7.227	0.99	0.997
Meconium	PWY.6163	Information	Pathway	-30.706	68.95		
Meconium	PWY.6163	Information	Interaction	1.23	105.428		
Meconium	PWY.6163	QTAC	Acetaminophen	-32.162	25.138	0.212	0.708
Meconium	PWY.6163	QTAC	Pathway	-200.955	239.84		
Meconium	PWY.6163	QTAC	Interaction	422.265	366.727		
Meconium	PWY.6163	Vocabulary	Acetaminophen	-13.653	10.704	0.157	0.68
Meconium	PWY.6163	Vocabulary	Pathway	-119.922	102.125		
Meconium	PWY.6163	Vocabulary	Interaction	204.195	156.155		
Meconium	PWY.6163	WISC sum	Acetaminophen	-31.346	24.538	0.15	0.68
Meconium	PWY.6163	WISC sum	Pathway	-223.882	234.122		
Meconium	PWY.6163	WISC sum	Interaction	476.565	357.985		
Meconium	PWY.6277	Block Design	Acetaminophen	-9.772	10.434	0.268	0.718
Meconium	PWY.6277	Block Design	Pathway	-128.275	114.614		
Meconium	PWY.6277	Block Design	Interaction	164.891	161.672		
Meconium	PWY.6277	Coding	Acetaminophen	-4.206	9.301	0.591	0.86
Meconium	PWY.6277	Coding	Pathway	-23.525	102.164		

Exposure Window	Pathway	Outcome	Variable	Effect Estimate[a]	Std. Error	LRT[b]	
						p-value	q-value
Meconium	PWY.6277	Coding	Interaction	71.016	144.11		
Meconium	PWY.6277	Digit span	Acetaminophen	-10.893	7.616	0.122	0.68
Meconium	PWY.6277	Digit span	Pathway	17.433	83.664		
Meconium	PWY.6277	Digit span	Interaction	168.817	118.014		
Meconium	PWY.6277	Information	Acetaminophen	-4.242	7.957	0.567	0.857
Meconium	PWY.6277	Information	Pathway	-5.242	87.402		
Meconium	PWY.6277	Information	Interaction	64.703	123.288		
Meconium	PWY.6277	QTAC	Acetaminophen	-10.512	28.114	0.784	0.906
Meconium	PWY.6277	QTAC	Pathway	-116.436	308.818		
Meconium	PWY.6277	QTAC	Interaction	109.467	435.612		
Meconium	PWY.6277	Vocabulary	Acetaminophen	-14.632	11.766	0.164	0.68
Meconium	PWY.6277	Vocabulary	Pathway	-70.755	129.247		
Meconium	PWY.6277	Vocabulary	Interaction	234.384	182.313		
Meconium	PWY.6277	WISC sum	Acetaminophen	-43.745	26.464	0.065	0.68
Meconium	PWY.6277	WISC sum	Pathway	-210.364	290.693		
Meconium	PWY.6277	WISC sum	Interaction	703.811	410.046		
Meconium	PWY.6385	Block Design	Acetaminophen	-4.667	12.714	0.632	0.86
Meconium	PWY.6385	Block Design	Pathway	-52.705	135.738		
Meconium	PWY.6385	Block Design	Interaction	93.592	213.282		
Meconium	PWY.6385	Coding	Acetaminophen	15.072	10.782	0.141	0.68
Meconium	PWY.6385	Coding	Pathway	211.243	115.118		
Meconium	PWY.6385	Coding	Interaction	-246.036	180.883		
Meconium	PWY.6385	Digit span	Acetaminophen	-3.832	9.538	0.672	0.863
Meconium	PWY.6385	Digit span	Pathway	86.388	101.833		
Meconium	PWY.6385	Digit span	Interaction	62.003	160.008		
Meconium	PWY.6385	Information	Acetaminophen	-9.725	9.444	0.266	0.718
Meconium	PWY.6385	Information	Pathway	-9.022	100.826		
Meconium	PWY.6385	Information	Interaction	162.185	158.426		
Meconium	PWY.6385	QTAC	Acetaminophen	2.641	33.77	0.849	0.959
Meconium	PWY.6385	QTAC	Pathway	161.802	360.541		
Meconium	PWY.6385	QTAC	Interaction	-98.711	566.511		
Meconium	PWY.6385	Vocabulary	Acetaminophen	-16.191	14.266	0.209	0.708
Meconium	PWY.6385	Vocabulary	Pathway	-131.048	152.305		
Meconium	PWY.6385	Vocabulary	Interaction	277.477	239.313		
Meconium	PWY.6385	WISC sum	Acetaminophen	-19.342	32.716	0.488	0.857
Meconium	PWY.6385	WISC sum	Pathway	104.856	349.294		

Exposure Window	Pathway	Outcome	Variable	Effect Estimate[a]	Std. Error	LRT[b]	
						p-value	q-value
Meconium	PWY.6385	WISC sum	Interaction	349.221	548.838		
Meconium	PWY.6386	Block Design	Acetaminophen	-10.535	13.876		
Meconium	PWY.6386	Block Design	Pathway	-89.462	132.477	0.369	0.8
Meconium	PWY.6386	Block Design	Interaction	167.365	202.677		
Meconium	PWY.6386	Coding	Acetaminophen	2.226	12.19		
Meconium	PWY.6386	Coding	Pathway	90.099	116.38	0.87	0.965
Meconium	PWY.6386	Coding	Interaction	-26.639	178.05		
Meconium	PWY.6386	Digit span	Acetaminophen	-13.392	10.24		
Meconium	PWY.6386	Digit span	Pathway	25.118	97.769	0.161	0.68
Meconium	PWY.6386	Digit span	Interaction	193.8	149.577		
Meconium	PWY.6386	Information	Acetaminophen	-12.645	10.377		
Meconium	PWY.6386	Information	Pathway	-60.644	99.072	0.19	0.694
Meconium	PWY.6386	Information	Interaction	183.322	151.569		
Meconium	PWY.6386	QTAC	Acetaminophen	-3.456	37.105		
Meconium	PWY.6386	QTAC	Pathway	100.464	354.262	0.995	0.997
Meconium	PWY.6386	QTAC	Interaction	2.94	541.985		
Meconium	PWY.6386	Vocabulary	Acetaminophen	-23.682	15.465		
Meconium	PWY.6386	Vocabulary	Pathway	-142.186	147.65	0.093	0.68
Meconium	PWY.6386	Vocabulary	Interaction	351.63	225.89		
Meconium	PWY.6386	WISC sum	Acetaminophen	-58.028	34.934		
Meconium	PWY.6386	WISC sum	Pathway	-177.075	333.533	0.067	0.68
Meconium	PWY.6386	WISC sum	Interaction	869.478	510.271		
Meconium	PWY.6387	Block Design	Acetaminophen	-9.716	14.951		
Meconium	PWY.6387	Block Design	Pathway	-94.736	142.62	0.439	0.851
Meconium	PWY.6387	Block Design	Interaction	160.798	226.299		
Meconium	PWY.6387	Coding	Acetaminophen	2.419	13.131		
Meconium	PWY.6387	Coding	Pathway	92.572	125.262	0.867	0.965
Meconium	PWY.6387	Coding	Interaction	-30.56	198.756		
Meconium	PWY.6387	Digit span	Acetaminophen	-13.221	11.102		
Meconium	PWY.6387	Digit span	Pathway	29.522	105.905	0.201	0.708
Meconium	PWY.6387	Digit span	Interaction	198.116	168.043		
Meconium	PWY.6387	Information	Acetaminophen	-11.054	11.243		
Meconium	PWY.6387	Information	Pathway	-64.131	107.251	0.29	0.718
Meconium	PWY.6387	Information	Interaction	165.548	170.178		
Meconium	PWY.6387	QTAC	Acetaminophen	-3.983	39.909	0.984	0.997
Meconium	PWY.6387	QTAC	Pathway	111.588	380.697		

Exposure Window	Pathway	Outcome	Variable	Effect Estimate[a]	Std. Error	LRT[b]	
						p-value	q-value
Meconium	PWY.6387	QTAC	Interaction	11.182	604.061		
Meconium	PWY.6387	Vocabulary	Acetaminophen	-23.807	16.7	0.118	0.68
Meconium	PWY.6387	Vocabulary	Pathway	-139.727	159.302		
Meconium	PWY.6387	Vocabulary	Interaction	366.156	252.768		
Meconium	PWY.6387	WISC sum	Acetaminophen	-55.379	37.992	0.107	0.68
Meconium	PWY.6387	WISC sum	Pathway	-176.5	362.409		
Meconium	PWY.6387	WISC sum	Interaction	860.059	575.044		
Meconium	PWY.6609	Block Design	Acetaminophen	-0.002	7.633	0.905	0.974
Meconium	PWY.6609	Block Design	Pathway	-78.408	80.586		
Meconium	PWY.6609	Block Design	Interaction	14.194	129.615		
Meconium	PWY.6609	Coding	Acetaminophen	10.348	6.625	0.102	0.68
Meconium	PWY.6609	Coding	Pathway	100.778	69.953		
Meconium	PWY.6609	Coding	Interaction	-170.687	112.513		
Meconium	PWY.6609	Digit span	Acetaminophen	-2.087	5.95	0.733	0.88
Meconium	PWY.6609	Digit span	Pathway	5.749	62.826		
Meconium	PWY.6609	Digit span	Interaction	31.562	101.05		
Meconium	PWY.6609	Information	Acetaminophen	3.003	5.844	0.555	0.857
Meconium	PWY.6609	Information	Pathway	27.625	61.706		
Meconium	PWY.6609	Information	Interaction	-53.685	99.249		
Meconium	PWY.6609	QTAC	Acetaminophen	-19.882	20.432	0.377	0.8
Meconium	PWY.6609	QTAC	Pathway	-174.052	215.727		
Meconium	PWY.6609	QTAC	Interaction	281.458	346.977		
Meconium	PWY.6609	Vocabulary	Acetaminophen	5.32	8.773	0.528	0.857
Meconium	PWY.6609	Vocabulary	Pathway	1.508	92.632		
Meconium	PWY.6609	Vocabulary	Interaction	-86.106	148.989		
Meconium	PWY.6609	WISC sum	Acetaminophen	16.582	20.072	0.398	0.828
Meconium	PWY.6609	WISC sum	Pathway	57.252	211.92		
Meconium	PWY.6609	WISC sum	Interaction	-264.721	340.853		
Meconium	PWY.6737	Block Design	Acetaminophen	-6.506	8.557	0.373	0.8
Meconium	PWY.6737	Block Design	Pathway	-145.006	80.069		
Meconium	PWY.6737	Block Design	Interaction	95.574	116.767		
Meconium	PWY.6737	Coding	Acetaminophen	-2.732	7.776	0.643	0.86
Meconium	PWY.6737	Coding	Pathway	24.203	72.766		
Meconium	PWY.6737	Coding	Interaction	44.992	106.116		
Meconium	PWY.6737	Digit span	Acetaminophen	-2.946	6.808	0.646	0.86
Meconium	PWY.6737	Digit span	Pathway	15.51	63.706		

Exposure Window	Pathway	Outcome	Variable	Effect Estimate[a]	Std. Error	LRT[b]	
						p-value	q-value
Meconium	PWY.6737	Digit span	Interaction	39.046	92.905		
Meconium	PWY.6737	Information	Acetaminophen	4.304	6.667	0.449	0.851
Meconium	PWY.6737	Information	Pathway	-2.957	62.381		
Meconium	PWY.6737	Information	Interaction	-63.215	90.972		
Meconium	PWY.6737	QTAC	Acetaminophen	-42.733	22.726	0.058	0.68
Meconium	PWY.6737	QTAC	Pathway	-150.439	212.657		
Meconium	PWY.6737	QTAC	Interaction	548.193	310.123		
Meconium	PWY.6737	Vocabulary	Acetaminophen	-5.101	10.103	0.568	0.857
Meconium	PWY.6737	Vocabulary	Pathway	-72.21	94.54		
Meconium	PWY.6737	Vocabulary	Interaction	72.12	137.87		
Meconium	PWY.6737	WISC sum	Acetaminophen	-12.981	23.122	0.515	0.857
Meconium	PWY.6737	WISC sum	Pathway	-180.46	216.36		
Meconium	PWY.6737	WISC sum	Interaction	188.517	315.523		
Meconium	PWY.7111	Block Design	Acetaminophen	-5.417	9.142	0.454	0.853
Meconium	PWY.7111	Block Design	Pathway	-64.301	86.9		
Meconium	PWY.7111	Block Design	Interaction	82.352	119.765		
Meconium	PWY.7111	Coding	Acetaminophen	1.32	8.103	0.891	0.968
Meconium	PWY.7111	Coding	Pathway	3.252	77.031		
Meconium	PWY.7111	Coding	Interaction	-13.257	106.164		
Meconium	PWY.7111	Digit span	Acetaminophen	-3.181	6.889	0.614	0.86
Meconium	PWY.7111	Digit span	Pathway	37.685	65.487		
Meconium	PWY.7111	Digit span	Interaction	41.674	90.255		
Meconium	PWY.7111	Information	Acetaminophen	-3.44	6.631	0.623	0.86
Meconium	PWY.7111	Information	Pathway	-102.257	63.038		
Meconium	PWY.7111	Information	Interaction	39.113	86.879		
Meconium	PWY.7111	QTAC	Acetaminophen	-14.458	24.343	0.609	0.86
Meconium	PWY.7111	QTAC	Pathway	-21.088	231.405		
Meconium	PWY.7111	QTAC	Interaction	149.528	318.923		
Meconium	PWY.7111	Vocabulary	Acetaminophen	-18.85	9.938	0.04	0.68
Meconium	PWY.7111	Vocabulary	Pathway	-194.936	94.469		
Meconium	PWY.7111	Vocabulary	Interaction	249.461	130.197		
Meconium	PWY.7111	WISC sum	Acetaminophen	-29.568	23.388	0.158	0.68
Meconium	PWY.7111	WISC sum	Pathway	-320.557	222.323		
Meconium	PWY.7111	WISC sum	Interaction	399.343	306.407		
Meconium	PWY.7219	Block Design	Acetaminophen	4.595	9.784	0.675	0.863
Meconium	PWY.7219	Block Design	Pathway	-21.381	84.51		

Exposure Window	Pathway	Outcome	Variable	Effect Estimate[a]	Std. Error	LRT[b] p-value	LRT[b] q-value
Meconium	PWY.7219	Block Design	Interaction	-57.11	148.974		
Meconium	PWY.7219	Coding	Acetaminophen	-4.179	8.527		
Meconium	PWY.7219	Coding	Pathway	36.716	73.653	0.556	0.857
Meconium	PWY.7219	Coding	Interaction	70.136	129.835		
Meconium	PWY.7219	Digit span	Acetaminophen	-3.877	7.259		
Meconium	PWY.7219	Digit span	Pathway	70.89	62.702	0.573	0.86
Meconium	PWY.7219	Digit span	Interaction	57.026	110.531		
Meconium	PWY.7219	Information	Acetaminophen	-6.849	7.364		
Meconium	PWY.7219	Information	Pathway	-32.302	63.602	0.32	0.76
Meconium	PWY.7219	Information	Interaction	102.609	112.117		
Meconium	PWY.7219	QTAC	Acetaminophen	-14.252	26.071		
Meconium	PWY.7219	QTAC	Pathway	-113.784	225.18	0.649	0.86
Meconium	PWY.7219	QTAC	Interaction	165.547	396.946		
Meconium	PWY.7219	Vocabulary	Acetaminophen	-9.4	11.049		
Meconium	PWY.7219	Vocabulary	Pathway	-96.687	95.436	0.339	0.77
Meconium	PWY.7219	Vocabulary	Interaction	147.799	168.233		
Meconium	PWY.7219	WISC sum	Acetaminophen	-19.711	25.411		
Meconium	PWY.7219	WISC sum	Pathway	-42.764	219.484	0.367	0.8
Meconium	PWY.7219	WISC sum	Interaction	320.46	386.905		
Meconium	PWY.7221	Block Design	Acetaminophen	6.012	11.406		
Meconium	PWY.7221	Block Design	Pathway	21.273	131.613	0.625	0.86
Meconium	PWY.7221	Block Design	Interaction	-77.74	173.617		
Meconium	PWY.7221	Coding	Acetaminophen	5.617	10.031		
Meconium	PWY.7221	Coding	Pathway	23.982	115.751	0.565	0.857
Meconium	PWY.7221	Coding	Interaction	-80.607	152.692		
Meconium	PWY.7221	Digit span	Acetaminophen	-4.357	8.67		
Meconium	PWY.7221	Digit span	Pathway	30.981	100.044	0.607	0.86
Meconium	PWY.7221	Digit span	Interaction	62.131	131.973		
Meconium	PWY.7221	Information	Acetaminophen	-0.539	8.631		
Meconium	PWY.7221	Information	Pathway	-27.234	99.598	0.959	0.994
Meconium	PWY.7221	Information	Interaction	6.19	131.385		
Meconium	PWY.7221	QTAC	Acetaminophen	-11.173	29.996		
Meconium	PWY.7221	QTAC	Pathway	-303.92	346.128	0.772	0.906
Meconium	PWY.7221	QTAC	Interaction	120.801	456.595		
Meconium	PWY.7221	Vocabulary	Acetaminophen	-16.605	12.744	0.149	0.68
Meconium	PWY.7221	Vocabulary	Pathway	-149.676	147.059		

Exposure Window	Pathway	Outcome	Variable	Effect Estimate[a]	Std. Error	LRT[b]	
						p-value	q-value
Meconium	PWY.7221	Vocabulary	Interaction	258.516	193.993		
Meconium	PWY.7221	WISC sum	Acetaminophen	-9.872	29.781	0.685	0.863
Meconium	PWY.7221	WISC sum	Pathway	-100.674	343.646		
Meconium	PWY.7221	WISC sum	Interaction	168.491	453.321		
Meconium	PWY.7400	Block Design	Acetaminophen	-5.802	9.501	0.443	0.851
Meconium	PWY.7400	Block Design	Pathway	-79.104	99.685		
Meconium	PWY.7400	Block Design	Interaction	98.67	140.233		
Meconium	PWY.7400	Coding	Acetaminophen	-7.515	8.288	0.296	0.718
Meconium	PWY.7400	Coding	Pathway	-20.717	86.962		
Meconium	PWY.7400	Coding	Interaction	117.716	122.335		
Meconium	PWY.7400	Digit span	Acetaminophen	-12.45	6.857	0.052	0.68
Meconium	PWY.7400	Digit span	Pathway	-13.947	71.95		
Meconium	PWY.7400	Digit span	Interaction	183.499	101.216		
Meconium	PWY.7400	Information	Acetaminophen	-4.866	7.179	0.476	0.857
Meconium	PWY.7400	Information	Pathway	-60.299	75.321		
Meconium	PWY.7400	Information	Interaction	69.246	105.958		
Meconium	PWY.7400	QTAC	Acetaminophen	-31.774	25.028	0.214	0.708
Meconium	PWY.7400	QTAC	Pathway	-180.886	262.601		
Meconium	PWY.7400	QTAC	Interaction	422.852	369.417		
Meconium	PWY.7400	Vocabulary	Acetaminophen	-13.897	10.66	0.147	0.68
Meconium	PWY.7400	Vocabulary	Pathway	-100.248	111.85		
Meconium	PWY.7400	Vocabulary	Interaction	211.245	157.346		
Meconium	PWY.7400	WISC sum	Acetaminophen	-44.53	23.831	0.039	0.68
Meconium	PWY.7400	WISC sum	Pathway	-274.315	250.049		
Meconium	PWY.7400	WISC sum	Interaction	680.375	351.759		
Meconium	TRNA.CHARGING.PWY	Block Design	Acetaminophen	-7.952	13.708	0.481	0.857
Meconium	TRNA.CHARGING.PWY	Block Design	Pathway	-117.428	142.436		
Meconium	TRNA.CHARGING.PWY	Block Design	Interaction	145.005	224.213		
Meconium	TRNA.CHARGING.PWY	Coding	Acetaminophen	-10.188	11.82	0.33	0.761
Meconium	TRNA.CHARGING.PWY	Coding	Pathway	47.491	122.815		
Meconium	TRNA.CHARGING.PWY	Coding	Interaction	173.025	193.326		
Meconium	TRNA.CHARGING.PWY	Digit span	Acetaminophen	-14.253	10.107	0.133	0.68
Meconium	TRNA.CHARGING.PWY	Digit span	Pathway	26.324	105.013		
Meconium	TRNA.CHARGING.PWY	Digit span	Interaction	229.944	165.304		
Meconium	TRNA.CHARGING.PWY	Information	Acetaminophen	-18.603	9.982	0.047	0.68
Meconium	TRNA.CHARGING.PWY	Information	Pathway	-74.31	103.718		

Exposure Window	Pathway	Outcome	Variable	Effect Estimate[a]	Std. Error	LRT[b]	
						p-value	q-value
Meconium	TRNA.CHARGING.PWY	Information	Interaction	302.859	163.264		
Meconium	TRNA.CHARGING.PWY	QTAC	Acetaminophen	-16.295	36.436	0.696	0.866
Meconium	TRNA.CHARGING.PWY	QTAC	Pathway	118.536	378.59		
Meconium	TRNA.CHARGING.PWY	QTAC	Interaction	213.191	595.948		
Meconium	TRNA.CHARGING.PWY	Vocabulary	Acetaminophen	-18.882	15.394	0.175	0.68
Meconium	TRNA.CHARGING.PWY	Vocabulary	Pathway	-74.146	159.954		
Meconium	TRNA.CHARGING.PWY	Vocabulary	Interaction	315.09	251.788		
Meconium	TRNA.CHARGING.PWY	WISC sum	Acetaminophen	-69.878	33.77	0.025	0.68
Meconium	TRNA.CHARGING.PWY	WISC sum	Pathway	-192.068	350.88		
Meconium	TRNA.CHARGING.PWY	WISC sum	Interaction	1165.924	552.328		
Meconium	UNINTEGRATED	Block Design	Acetaminophen	-10.361	30.422	0.684	0.863
Meconium	UNINTEGRATED	Block Design	Pathway	0.008	0.333		
Meconium	UNINTEGRATED	Block Design	Interaction	0.157	0.421		
Meconium	UNINTEGRATED	Coding	Acetaminophen	15.582	26.748	0.532	0.857
Meconium	UNINTEGRATED	Coding	Pathway	0.053	0.293		
Meconium	UNINTEGRATED	Coding	Interaction	-0.212	0.37		
Meconium	UNINTEGRATED	Digit span	Acetaminophen	-0.807	23.479	0.979	0.997
Meconium	UNINTEGRATED	Digit span	Pathway	0.043	0.257		
Meconium	UNINTEGRATED	Digit span	Interaction	0.008	0.325		
Meconium	UNINTEGRATED	Information	Acetaminophen	34.975	22.067	0.086	0.68
Meconium	UNINTEGRATED	Information	Pathway	0.138	0.242		
Meconium	UNINTEGRATED	Information	Interaction	-0.487	0.305		
Meconium	UNINTEGRATED	QTAC	Acetaminophen	-150.943	77.597	0.042	0.68
Meconium	UNINTEGRATED	QTAC	Pathway	-1.587	0.85		
Meconium	UNINTEGRATED	QTAC	Interaction	2.039	1.073		
Meconium	UNINTEGRATED	Vocabulary	Acetaminophen	-1.07	34.789	0.965	0.996
Meconium	UNINTEGRATED	Vocabulary	Pathway	-0.019	0.381		
Meconium	UNINTEGRATED	Vocabulary	Interaction	0.019	0.481		
Meconium	UNINTEGRATED	WISC sum	Acetaminophen	38.319	79.475	0.609	0.86
Meconium	UNINTEGRATED	WISC sum	Pathway	0.223	0.871		
Meconium	UNINTEGRATED	WISC sum	Interaction	-0.515	1.099		
Meconium	UNMAPPED	Block Design	Acetaminophen	4.473	8.721	0.661	0.863
Meconium	UNMAPPED	Block Design	Pathway	-0.009	0.309		
Meconium	UNMAPPED	Block Design	Interaction	-0.157	0.392		
Meconium	UNMAPPED	Coding	Acetaminophen	-4.245	7.677	0.518	0.857
Meconium	UNMAPPED	Coding	Pathway	-0.064	0.272		

Exposure Window	Pathway	Outcome	Variable	Effect Estimate[a]	Std. Error	LRT[b]	
						p-value	q-value
Meconium	UNMAPPED	Coding	Interaction	0.205	0.345		
Meconium	UNMAPPED	Digit span	Acetaminophen	0.655	6.733	0.886	0.968
Meconium	UNMAPPED	Digit span	Pathway	-0.024	0.238		
Meconium	UNMAPPED	Digit span	Interaction	-0.04	0.303		
Meconium	UNMAPPED	Information	Acetaminophen	-9.991	6.34	0.096	0.68
Meconium	UNMAPPED	Information	Pathway	-0.113	0.225		
Meconium	UNMAPPED	Information	Interaction	0.44	0.285		
Meconium	UNMAPPED	QTAC	Acetaminophen	35.212	22.431	0.062	0.68
Meconium	UNMAPPED	QTAC	Pathway	1.338	0.795		
Meconium	UNMAPPED	QTAC	Interaction	-1.752	1.008		
Meconium	UNMAPPED	Vocabulary	Acetaminophen	1.732	9.977	0.875	0.965
Meconium	UNMAPPED	Vocabulary	Pathway	0.059	0.353		
Meconium	UNMAPPED	Vocabulary	Interaction	-0.064	0.449		
Meconium	UNMAPPED	WISC sum	Acetaminophen	-7.375	22.823	0.684	0.863
Meconium	UNMAPPED	WISC sum	Pathway	-0.152	0.808		
Meconium	UNMAPPED	WISC sum	Interaction	0.383	1.026		
Meconium	VALSYN.PWY	Block Design	Acetaminophen	-4.98	9.327	0.492	0.857
Meconium	VALSYN.PWY	Block Design	Pathway	-53.816	89.422		
Meconium	VALSYN.PWY	Block Design	Interaction	76.887	121.977		
Meconium	VALSYN.PWY	Coding	Acetaminophen	-0.313	8.252	0.935	0.982
Meconium	VALSYN.PWY	Coding	Pathway	-12.143	79.115		
Meconium	VALSYN.PWY	Coding	Interaction	8.044	107.918		
Meconium	VALSYN.PWY	Digit span	Acetaminophen	-3.135	7.002	0.623	0.86
Meconium	VALSYN.PWY	Digit span	Pathway	40.972	67.128		
Meconium	VALSYN.PWY	Digit span	Interaction	41.172	91.566		
Meconium	VALSYN.PWY	Information	Acetaminophen	-2.968	6.787	0.685	0.863
Meconium	VALSYN.PWY	Information	Pathway	-96.617	65.072		
Meconium	VALSYN.PWY	Information	Interaction	32.979	88.762		
Meconium	VALSYN.PWY	QTAC	Acetaminophen	-16.668	24.772	0.548	0.857
Meconium	VALSYN.PWY	QTAC	Pathway	-41.349	237.496		
Meconium	VALSYN.PWY	QTAC	Interaction	178.14	323.959		
Meconium	VALSYN.PWY	Vocabulary	Acetaminophen	-18.001	10.191	0.055	0.68
Meconium	VALSYN.PWY	Vocabulary	Pathway	-187.546	97.698		
Meconium	VALSYN.PWY	Vocabulary	Interaction	238.009	133.266		
Meconium	VALSYN.PWY	WISC sum	Acetaminophen	-29.398	23.88	0.169	0.68
Meconium	VALSYN.PWY	WISC sum	Pathway	-309.15	228.944		

Exposure Window	Pathway	Outcome	Variable	Effect Estimate[a]	Std. Error	LRT[b]	
						p-value	q-value
Meconium	VALSYN.PWY	WISC sum	Interaction	397.091	312.294		
Meconium Adj.[c]	ARGSYN.PWY	Block Design	Acetaminophen	-5.816	9.632	0.444	0.853
Meconium Adj.[c]	ARGSYN.PWY	Block Design	Pathway	-78.553	101.28		
Meconium Adj.[c]	ARGSYN.PWY	Block Design	Interaction	0.062	0.961		
Meconium Adj.[c]	ARGSYN.PWY	Coding	Acetaminophen	-7.335	8.348	0.309	0.74
Meconium Adj.[c]	ARGSYN.PWY	Coding	Pathway	-13.799	87.77		
Meconium Adj.[c]	ARGSYN.PWY	Coding	Interaction	0.619	0.833		
Meconium Adj.[c]	ARGSYN.PWY	Digit span	Acetaminophen	-12.5	6.95	0.051	0.688
Meconium Adj.[c]	ARGSYN.PWY	Digit span	Pathway	-14.252	73.076		
Meconium Adj.[c]	ARGSYN.PWY	Digit span	Interaction	-0.037	0.693		
Meconium Adj.[c]	ARGSYN.PWY	Information	Acetaminophen	-4.577	7.116	0.507	0.863
Meconium Adj.[c]	ARGSYN.PWY	Information	Pathway	-48.541	74.822		
Meconium Adj.[c]	ARGSYN.PWY	Information	Interaction	0.969	0.71		
Meconium Adj.[c]	ARGSYN.PWY	QTAC	Acetaminophen	-32.778	24.44	0.176	0.688
Meconium Adj.[c]	ARGSYN.PWY	QTAC	Pathway	-228.665	256.971		
Meconium Adj.[c]	ARGSYN.PWY	QTAC	Interaction	-4.333	2.438		
Meconium Adj.[c]	ARGSYN.PWY	Vocabulary	Acetaminophen	-13.526	10.694	0.157	0.688
Meconium Adj.[c]	ARGSYN.PWY	Vocabulary	Pathway	-87.804	112.442		
Meconium Adj.[c]	ARGSYN.PWY	Vocabulary	Interaction	1.006	1.067		
Meconium Adj.[c]	ARGSYN.PWY	WISC sum	Acetaminophen	-43.755	23.808	0.041	0.688
Meconium Adj.[c]	ARGSYN.PWY	WISC sum	Pathway	-242.949	250.333		
Meconium Adj.[c]	ARGSYN.PWY	WISC sum	Interaction	2.619	2.375		
Meconium Adj.[c]	ARGSYNBSUB.PWY	Block Design	Acetaminophen	-6.093	9.541	0.421	0.848
Meconium Adj.[c]	ARGSYNBSUB.PWY	Block Design	Pathway	-75.95	94.634		
Meconium Adj.[c]	ARGSYNBSUB.PWY	Block Design	Interaction	0.049	0.962		
Meconium Adj.[c]	ARGSYNBSUB.PWY	Coding	Acetaminophen	-5.225	8.324	0.458	0.853
Meconium Adj.[c]	ARGSYNBSUB.PWY	Coding	Pathway	-0.446	82.56		
Meconium Adj.[c]	ARGSYNBSUB.PWY	Coding	Interaction	0.623	0.839		
Meconium Adj.[c]	ARGSYNBSUB.PWY	Digit span	Acetaminophen	-12.589	6.909	0.048	0.688
Meconium Adj.[c]	ARGSYNBSUB.PWY	Digit span	Pathway	-18.521	68.53		
Meconium Adj.[c]	ARGSYNBSUB.PWY	Digit span	Interaction	-0.046	0.697		
Meconium Adj.[c]	ARGSYNBSUB.PWY	Information	Acetaminophen	-4.188	7.058	0.543	0.883
Meconium Adj.[c]	ARGSYNBSUB.PWY	Information	Pathway	-43.027	70.005		
Meconium Adj.[c]	ARGSYNBSUB.PWY	Information	Interaction	0.963	0.712		
Meconium Adj.[c]	ARGSYNBSUB.PWY	QTAC	Acetaminophen	-30.053	24.286	0.216	0.699
Meconium Adj.[c]	ARGSYNBSUB.PWY	QTAC	Pathway	-235.786	240.875		

Exposure Window	Pathway	Outcome	Variable	Effect Estimate[a]	Std. Error	LRT[b]	
						p-value	q-value
Meconium Adj.[c]	ARGSYNBSUB.PWY	QTAC	Interaction	-4.429	2.449		
Meconium Adj.[c]	ARGSYNBSUB.PWY	Vocabulary	Acetaminophen	-13.258	10.61	0.162	0.688
Meconium Adj.[c]	ARGSYNBSUB.PWY	Vocabulary	Pathway	-85.698	105.228		
Meconium Adj.[c]	ARGSYNBSUB.PWY	Vocabulary	Interaction	0.986	1.07		
Meconium Adj.[c]	ARGSYNBSUB.PWY	WISC sum	Acetaminophen	-41.353	23.724	0.052	0.688
Meconium Adj.[c]	ARGSYNBSUB.PWY	WISC sum	Pathway	-223.642	235.297		
Meconium Adj.[c]	ARGSYNBSUB.PWY	WISC sum	Interaction	2.575	2.392		
Meconium Adj.[c]	ARO.PWY	Block Design	Acetaminophen	-9.378	10.388	0.279	0.724
Meconium Adj.[c]	ARO.PWY	Block Design	Pathway	-111.771	98.674		
Meconium Adj.[c]	ARO.PWY	Block Design	Interaction	0.091	0.947		
Meconium Adj.[c]	ARO.PWY	Coding	Acetaminophen	-0.654	9.148	0.902	0.959
Meconium Adj.[c]	ARO.PWY	Coding	Pathway	49.784	86.893		
Meconium Adj.[c]	ARO.PWY	Coding	Interaction	0.624	0.834		
Meconium Adj.[c]	ARO.PWY	Digit span	Acetaminophen	-8.613	7.893	0.236	0.709
Meconium Adj.[c]	ARO.PWY	Digit span	Pathway	-1.241	74.977		
Meconium Adj.[c]	ARO.PWY	Digit span	Interaction	-0.075	0.72		
Meconium Adj.[c]	ARO.PWY	Information	Acetaminophen	1.533	7.748	0.788	0.928
Meconium Adj.[c]	ARO.PWY	Information	Pathway	-17.103	73.59		
Meconium Adj.[c]	ARO.PWY	Information	Interaction	0.985	0.706		
Meconium Adj.[c]	ARO.PWY	QTAC	Acetaminophen	-33.319	26.569	0.207	0.688
Meconium Adj.[c]	ARO.PWY	QTAC	Pathway	-297.972	252.369		
Meconium Adj.[c]	ARO.PWY	QTAC	Interaction	-4.312	2.423		
Meconium Adj.[c]	ARO.PWY	Vocabulary	Acetaminophen	-10.25	11.746	0.329	0.761
Meconium Adj.[c]	ARO.PWY	Vocabulary	Pathway	-102.09	111.567		
Meconium Adj.[c]	ARO.PWY	Vocabulary	Interaction	0.999	1.071		
Meconium Adj.[c]	ARO.PWY	WISC sum	Acetaminophen	-27.362	26.789	0.246	0.709
Meconium Adj.[c]	ARO.PWY	WISC sum	Pathway	-182.421	254.457		
Meconium Adj.[c]	ARO.PWY	WISC sum	Interaction	2.625	2.443		
Meconium Adj.[c]	BRANCHED.CHAIN.AA.SYN.PWY	Block Design	Acetaminophen	2.428	9.164	0.843	0.938
Meconium Adj.[c]	BRANCHED.CHAIN.AA.SYN.PWY	Block Design	Pathway	-20.026	104.977		
Meconium Adj.[c]	BRANCHED.CHAIN.AA.SYN.PWY	Block Design	Interaction	0.133	0.972		
Meconium Adj.[c]	BRANCHED.CHAIN.AA.SYN.PWY	Coding	Acetaminophen	1.098	8.04	0.9	0.959
Meconium Adj.[c]	BRANCHED.CHAIN.AA.SYN.PWY	Coding	Pathway	-11.765	92.101		
Meconium Adj.[c]	BRANCHED.CHAIN.AA.SYN.PWY	Coding	Interaction	0.574	0.853		
Meconium Adj.[c]	BRANCHED.CHAIN.AA.SYN.PWY	Digit span	Acetaminophen	2.726	7.048	0.644	0.885
Meconium Adj.[c]	BRANCHED.CHAIN.AA.SYN.PWY	Digit span	Pathway	38.954	80.731		

Exposure Window	Pathway	Outcome	Variable	Effect Estimate[a]	Std. Error	LRT[b]	
						p-value	q-value
Meconium Adj.[c]	BRANCHED.CHAIN.AA.SYN.PWY	Digit span	Interaction	-0.06	0.748		
Meconium Adj.[c]	BRANCHED.CHAIN.AA.SYN.PWY	Information	Acetaminophen	-1.82	6.58	0.825	0.938
Meconium Adj.[c]	BRANCHED.CHAIN.AA.SYN.PWY	Information	Pathway	-87.73	75.369		
Meconium Adj.[c]	BRANCHED.CHAIN.AA.SYN.PWY	Information	Interaction	0.926	0.698		
Meconium Adj.[c]	BRANCHED.CHAIN.AA.SYN.PWY	QTAC	Acetaminophen	-7.954	23.558	0.811	0.938
Meconium Adj.[c]	BRANCHED.CHAIN.AA.SYN.PWY	QTAC	Pathway	-88.721	269.849		
Meconium Adj.[c]	BRANCHED.CHAIN.AA.SYN.PWY	QTAC	Interaction	-4.345	2.5		
Meconium Adj.[c]	BRANCHED.CHAIN.AA.SYN.PWY	Vocabulary	Acetaminophen	-13.1	9.779	0.146	0.688
Meconium Adj.[c]	BRANCHED.CHAIN.AA.SYN.PWY	Vocabulary	Pathway	-239.841	112.012		
Meconium Adj.[c]	BRANCHED.CHAIN.AA.SYN.PWY	Vocabulary	Interaction	0.701	1.038		
Meconium Adj.[c]	BRANCHED.CHAIN.AA.SYN.PWY	WISC sum	Acetaminophen	-8.667	23.034	0.673	0.885
Meconium Adj.[c]	BRANCHED.CHAIN.AA.SYN.PWY	WISC sum	Pathway	-320.408	263.854		
Meconium Adj.[c]	BRANCHED.CHAIN.AA.SYN.PWY	WISC sum	Interaction	2.274	2.444		
Meconium Adj.[c]	CALVIN.PWY	Block Design	Acetaminophen	-6.042	6.854	0.263	0.71
Meconium Adj.[c]	CALVIN.PWY	Block Design	Pathway	-76.112	85.173		
Meconium Adj.[c]	CALVIN.PWY	Block Design	Interaction	0.023	0.957		
Meconium Adj.[c]	CALVIN.PWY	Coding	Acetaminophen	3.07	6.065	0.604	0.885
Meconium Adj.[c]	CALVIN.PWY	Coding	Pathway	26.7	75.375		
Meconium Adj.[c]	CALVIN.PWY	Coding	Interaction	0.612	0.847		
Meconium Adj.[c]	CALVIN.PWY	Digit span	Acetaminophen	-5.563	5.198	0.251	0.709
Meconium Adj.[c]	CALVIN.PWY	Digit span	Pathway	-1.724	64.597		
Meconium Adj.[c]	CALVIN.PWY	Digit span	Interaction	-0.112	0.726		
Meconium Adj.[c]	CALVIN.PWY	Information	Acetaminophen	3.911	5.076	0.357	0.783
Meconium Adj.[c]	CALVIN.PWY	Information	Pathway	34.308	63.079		
Meconium Adj.[c]	CALVIN.PWY	Information	Interaction	1.058	0.709		
Meconium Adj.[c]	CALVIN.PWY	QTAC	Acetaminophen	-24.306	17.287	0.172	0.688
Meconium Adj.[c]	CALVIN.PWY	QTAC	Pathway	-337.436	214.826		
Meconium Adj.[c]	CALVIN.PWY	QTAC	Interaction	-4.728	2.414		
Meconium Adj.[c]	CALVIN.PWY	Vocabulary	Acetaminophen	-2.164	7.806	0.739	0.907
Meconium Adj.[c]	CALVIN.PWY	Vocabulary	Pathway	-28.818	97.007		
Meconium Adj.[c]	CALVIN.PWY	Vocabulary	Interaction	0.988	1.09		
Meconium Adj.[c]	CALVIN.PWY	WISC sum	Acetaminophen	-6.789	17.817	0.634	0.885
Meconium Adj.[c]	CALVIN.PWY	WISC sum	Pathway	-45.646	221.412		
Meconium Adj.[c]	CALVIN.PWY	WISC sum	Interaction	2.569	2.488		
Meconium Adj.[c]	COA.PWY.1	Block Design	Acetaminophen	-15.674	17.103	0.287	0.729
Meconium Adj.[c]	COA.PWY.1	Block Design	Pathway	-97.472	139.188		

Exposure Window	Pathway	Outcome	Variable	Effect Estimate[a]	Std. Error	LRT[b]	
						p-value	q-value
Meconium Adj.[c]	COA.PWY.1	Block Design	Interaction	-0.007	0.962		
Meconium Adj.[c]	COA.PWY.1	Coding	Acetaminophen	-17.218	14.791	0.191	0.688
Meconium Adj.[c]	COA.PWY.1	Coding	Pathway	-3.761	120.375		
Meconium Adj.[c]	COA.PWY.1	Coding	Interaction	0.403	0.832		
Meconium Adj.[c]	COA.PWY.1	Digit span	Acetaminophen	-21.197	12.55	0.066	0.688
Meconium Adj.[c]	COA.PWY.1	Digit span	Pathway	27.158	102.133		
Meconium Adj.[c]	COA.PWY.1	Digit span	Interaction	-0.336	0.706		
Meconium Adj.[c]	COA.PWY.1	Information	Acetaminophen	-27.368	12.015	0.015	0.688
Meconium Adj.[c]	COA.PWY.1	Information	Pathway	-134.512	97.785		
Meconium Adj.[c]	COA.PWY.1	Information	Interaction	0.778	0.676		
Meconium Adj.[c]	COA.PWY.1	QTAC	Acetaminophen	26.238	44.112	0.47	0.853
Meconium Adj.[c]	COA.PWY.1	QTAC	Pathway	222.148	359.001		
Meconium Adj.[c]	COA.PWY.1	QTAC	Interaction	-3.99	2.48		
Meconium Adj.[c]	COA.PWY.1	Vocabulary	Acetaminophen	-22.484	19.063	0.194	0.688
Meconium Adj.[c]	COA.PWY.1	Vocabulary	Pathway	-178.904	155.144		
Meconium Adj.[c]	COA.PWY.1	Vocabulary	Interaction	0.852	1.072		
Meconium Adj.[c]	COA.PWY.1	WISC sum	Acetaminophen	-103.942	41.248	0.007	0.688
Meconium Adj.[c]	COA.PWY.1	WISC sum	Pathway	-387.492	335.689		
Meconium Adj.[c]	COA.PWY.1	WISC sum	Interaction	1.69	2.319		
Meconium Adj.[c]	COMPLETE.ARO.PWY	Block Design	Acetaminophen	-6.733	9.514	0.377	0.793
Meconium Adj.[c]	COMPLETE.ARO.PWY	Block Design	Pathway	-78.276	98.662		
Meconium Adj.[c]	COMPLETE.ARO.PWY	Block Design	Interaction	0.086	0.955		
Meconium Adj.[c]	COMPLETE.ARO.PWY	Coding	Acetaminophen	4.298	8.34	0.596	0.885
Meconium Adj.[c]	COMPLETE.ARO.PWY	Coding	Pathway	68.282	86.485		
Meconium Adj.[c]	COMPLETE.ARO.PWY	Coding	Interaction	0.626	0.837		
Meconium Adj.[c]	COMPLETE.ARO.PWY	Digit span	Acetaminophen	-7.595	7.164	0.25	0.709
Meconium Adj.[c]	COMPLETE.ARO.PWY	Digit span	Pathway	2.986	74.287		
Meconium Adj.[c]	COMPLETE.ARO.PWY	Digit span	Interaction	-0.088	0.719		
Meconium Adj.[c]	COMPLETE.ARO.PWY	Information	Acetaminophen	0.902	7.061	0.847	0.938
Meconium Adj.[c]	COMPLETE.ARO.PWY	Information	Pathway	-10.826	73.22		
Meconium Adj.[c]	COMPLETE.ARO.PWY	Information	Interaction	0.995	0.709		
Meconium Adj.[c]	COMPLETE.ARO.PWY	QTAC	Acetaminophen	-22.28	24.216	0.378	0.793
Meconium Adj.[c]	COMPLETE.ARO.PWY	QTAC	Pathway	-293.515	251.117		
Meconium Adj.[c]	COMPLETE.ARO.PWY	QTAC	Interaction	-4.434	2.431		
Meconium Adj.[c]	COMPLETE.ARO.PWY	Vocabulary	Acetaminophen	-12.746	10.596	0.18	0.688
Meconium Adj.[c]	COMPLETE.ARO.PWY	Vocabulary	Pathway	-120.208	109.879		

Exposure Window	Pathway	Outcome	Variable	Effect Estimate[a]	Std. Error	LRT[b]	
						p-value	q-value
Meconium Adj.[c]	COMPLETE.ARO.PWY	Vocabulary	Interaction	0.964	1.064		
Meconium Adj.[c]	COMPLETE.ARO.PWY	WISC sum	Acetaminophen	-21.873	24.414	0.303	0.74
Meconium Adj.[c]	COMPLETE.ARO.PWY	WISC sum	Pathway	-138.043	253.169		
Meconium Adj.[c]	COMPLETE.ARO.PWY	WISC sum	Interaction	2.584	2.451		
Meconium Adj.[c]	DTDPRHAMSYN.PWY	Block Design	Acetaminophen	1.449	5.715	0.885	0.954
Meconium Adj.[c]	DTDPRHAMSYN.PWY	Block Design	Pathway	-62.631	65.31		
Meconium Adj.[c]	DTDPRHAMSYN.PWY	Block Design	Interaction	0.238	0.942		
Meconium Adj.[c]	DTDPRHAMSYN.PWY	Coding	Acetaminophen	12.199	4.728	0.006	0.688
Meconium Adj.[c]	DTDPRHAMSYN.PWY	Coding	Pathway	67.691	54.035		
Meconium Adj.[c]	DTDPRHAMSYN.PWY	Coding	Interaction	0.537	0.779		
Meconium Adj.[c]	DTDPRHAMSYN.PWY	Digit span	Acetaminophen	0.916	4.492	0.767	0.921
Meconium Adj.[c]	DTDPRHAMSYN.PWY	Digit span	Pathway	-0.402	51.335		
Meconium Adj.[c]	DTDPRHAMSYN.PWY	Digit span	Interaction	-0.114	0.741		
Meconium Adj.[c]	DTDPRHAMSYN.PWY	Information	Acetaminophen	4.468	4.224	0.205	0.688
Meconium Adj.[c]	DTDPRHAMSYN.PWY	Information	Pathway	13.725	48.27		
Meconium Adj.[c]	DTDPRHAMSYN.PWY	Information	Interaction	1.018	0.696		
Meconium Adj.[c]	DTDPRHAMSYN.PWY	QTAC	Acetaminophen	-6.93	14.978	0.761	0.918
Meconium Adj.[c]	DTDPRHAMSYN.PWY	QTAC	Pathway	-76.001	171.179		
Meconium Adj.[c]	DTDPRHAMSYN.PWY	QTAC	Interaction	-4.122	2.469		
Meconium Adj.[c]	DTDPRHAMSYN.PWY	Vocabulary	Acetaminophen	4.382	6.546	0.468	0.853
Meconium Adj.[c]	DTDPRHAMSYN.PWY	Vocabulary	Pathway	17.989	74.812		
Meconium Adj.[c]	DTDPRHAMSYN.PWY	Vocabulary	Interaction	1.027	1.079		
Meconium Adj.[c]	DTDPRHAMSYN.PWY	WISC sum	Acetaminophen	23.414	14.362	0.077	0.688
Meconium Adj.[c]	DTDPRHAMSYN.PWY	WISC sum	Pathway	36.373	164.139		
Meconium Adj.[c]	DTDPRHAMSYN.PWY	WISC sum	Interaction	2.706	2.368		
Meconium Adj.[c]	GLUTORN.PWY	Block Design	Acetaminophen	-5.56	7.928	0.369	0.793
Meconium Adj.[c]	GLUTORN.PWY	Block Design	Pathway	-71.668	92.803		
Meconium Adj.[c]	GLUTORN.PWY	Block Design	Interaction	0.104	0.958		
Meconium Adj.[c]	GLUTORN.PWY	Coding	Acetaminophen	-3.577	6.927	0.528	0.87
Meconium Adj.[c]	GLUTORN.PWY	Coding	Pathway	4.526	81.083		
Meconium Adj.[c]	GLUTORN.PWY	Coding	Interaction	0.639	0.837		
Meconium Adj.[c]	GLUTORN.PWY	Digit span	Acetaminophen	-9.326	5.878	0.085	0.688
Meconium Adj.[c]	GLUTORN.PWY	Digit span	Pathway	-31.861	68.801		
Meconium Adj.[c]	GLUTORN.PWY	Digit span	Interaction	-0.043	0.71		
Meconium Adj.[c]	GLUTORN.PWY	Information	Acetaminophen	-2.913	5.85	0.627	0.885
Meconium Adj.[c]	GLUTORN.PWY	Information	Pathway	-52.63	68.474		

Exposure Window	Pathway	Outcome	Variable	Effect Estimate[a]	Std. Error	LRT[b]	
						p-value	q-value
Meconium Adj.[c]	GLUTORN.PWY	Information	Interaction	0.96	0.707		
Meconium Adj.[c]	GLUTORN.PWY	QTAC	Acetaminophen	-30.261	20.04	0.13	0.688
Meconium Adj.[c]	GLUTORN.PWY	QTAC	Pathway	-227.715	234.568		
Meconium Adj.[c]	GLUTORN.PWY	QTAC	Interaction	-4.206	2.421		
Meconium Adj.[c]	GLUTORN.PWY	Vocabulary	Acetaminophen	-11.137	8.812	0.155	0.688
Meconium Adj.[c]	GLUTORN.PWY	Vocabulary	Pathway	-86.548	103.146		
Meconium Adj.[c]	GLUTORN.PWY	Vocabulary	Interaction	1.048	1.065		
Meconium Adj.[c]	GLUTORN.PWY	WISC sum	Acetaminophen	-32.513	19.828	0.065	0.688
Meconium Adj.[c]	GLUTORN.PWY	WISC sum	Pathway	-238.18	232.093		
Meconium Adj.[c]	GLUTORN.PWY	WISC sum	Interaction	2.708	2.396		
Meconium Adj.[c]	GLYCOGENSYNTH.PWY	Block Design	Acetaminophen	-1.77	5.943	0.618	0.885
Meconium Adj.[c]	GLYCOGENSYNTH.PWY	Block Design	Pathway	-38.747	77.201		
Meconium Adj.[c]	GLYCOGENSYNTH.PWY	Block Design	Interaction	0.149	0.962		
Meconium Adj.[c]	GLYCOGENSYNTH.PWY	Coding	Acetaminophen	2.708	5.166	0.587	0.885
Meconium Adj.[c]	GLYCOGENSYNTH.PWY	Coding	Pathway	-5.774	67.116		
Meconium Adj.[c]	GLYCOGENSYNTH.PWY	Coding	Interaction	0.639	0.836		
Meconium Adj.[c]	GLYCOGENSYNTH.PWY	Digit span	Acetaminophen	-5.806	4.473	0.165	0.688
Meconium Adj.[c]	GLYCOGENSYNTH.PWY	Digit span	Pathway	-36.346	58.114		
Meconium Adj.[c]	GLYCOGENSYNTH.PWY	Digit span	Interaction	-0.173	0.724		
Meconium Adj.[c]	GLYCOGENSYNTH.PWY	Information	Acetaminophen	5.191	4.3	0.156	0.688
Meconium Adj.[c]	GLYCOGENSYNTH.PWY	Information	Pathway	45.664	55.86		
Meconium Adj.[c]	GLYCOGENSYNTH.PWY	Information	Interaction	1.043	0.696		
Meconium Adj.[c]	GLYCOGENSYNTH.PWY	QTAC	Acetaminophen	-20.386	14.872	0.192	0.688
Meconium Adj.[c]	GLYCOGENSYNTH.PWY	QTAC	Pathway	-292.607	193.198		
Meconium Adj.[c]	GLYCOGENSYNTH.PWY	QTAC	Interaction	-4.044	2.408		
Meconium Adj.[c]	GLYCOGENSYNTH.PWY	Vocabulary	Acetaminophen	1.422	6.701	0.833	0.938
Meconium Adj.[c]	GLYCOGENSYNTH.PWY	Vocabulary	Pathway	0.99	87.053		
Meconium Adj.[c]	GLYCOGENSYNTH.PWY	Vocabulary	Interaction	1.056	1.085		
Meconium Adj.[c]	GLYCOGENSYNTH.PWY	WISC sum	Acetaminophen	1.745	15.305	0.94	0.983
Meconium Adj.[c]	GLYCOGENSYNTH.PWY	WISC sum	Pathway	-34.212	198.824		
Meconium Adj.[c]	GLYCOGENSYNTH.PWY	WISC sum	Interaction	2.713	2.478		
Meconium Adj.[c]	ILEUSYN.PWY	Block Design	Acetaminophen	-4.946	9.457	0.496	0.862
Meconium Adj.[c]	ILEUSYN.PWY	Block Design	Pathway	-52.793	91.813		
Meconium Adj.[c]	ILEUSYN.PWY	Block Design	Interaction	0.065	0.975		
Meconium Adj.[c]	ILEUSYN.PWY	Coding	Acetaminophen	-0.01	8.322	0.973	0.988
Meconium Adj.[c]	ILEUSYN.PWY	Coding	Pathway	-3.207	80.792		

Exposure Window	Pathway	Outcome	Variable	Effect Estimate[a]	Std. Error	LRT[b]	
						p-value	q-value
Meconium Adj.[c]	ILEUSYN.PWY	Coding	Interaction	0.569	0.858		
Meconium Adj.[c]	ILEUSYN.PWY	Digit span	Acetaminophen	-3.107	7.099	0.628	0.885
Meconium Adj.[c]	ILEUSYN.PWY	Digit span	Pathway	41.804	68.922		
Meconium Adj.[c]	ILEUSYN.PWY	Digit span	Interaction	0.053	0.732		
Meconium Adj.[c]	ILEUSYN.PWY	Information	Acetaminophen	-2.548	6.775	0.741	0.907
Meconium Adj.[c]	ILEUSYN.PWY	Information	Pathway	-84.218	65.773		
Meconium Adj.[c]	ILEUSYN.PWY	Information	Interaction	0.789	0.698		
Meconium Adj.[c]	ILEUSYN.PWY	QTAC	Acetaminophen	-18.955	24.24	0.457	0.853
Meconium Adj.[c]	ILEUSYN.PWY	QTAC	Pathway	-108.754	235.336		
Meconium Adj.[c]	ILEUSYN.PWY	QTAC	Interaction	-4.291	2.498		
Meconium Adj.[c]	ILEUSYN.PWY	Vocabulary	Acetaminophen	-17.586	10.263	0.061	0.688
Meconium Adj.[c]	ILEUSYN.PWY	Vocabulary	Pathway	-175.284	99.64		
Meconium Adj.[c]	ILEUSYN.PWY	Vocabulary	Interaction	0.781	1.058		
Meconium Adj.[c]	ILEUSYN.PWY	WISC sum	Acetaminophen	-28.195	23.965	0.186	0.688
Meconium Adj.[c]	ILEUSYN.PWY	WISC sum	Pathway	-273.698	232.664		
Meconium Adj.[c]	ILEUSYN.PWY	WISC sum	Interaction	2.257	2.47		
Meconium Adj.[c]	PEPTIDOGLYCANSYN.PWY	Block Design	Acetaminophen	-9.594	14.669	0.43	0.853
Meconium Adj.[c]	PEPTIDOGLYCANSYN.PWY	Block Design	Pathway	-99.668	148.592		
Meconium Adj.[c]	PEPTIDOGLYCANSYN.PWY	Block Design	Interaction	0.117	0.956		
Meconium Adj.[c]	PEPTIDOGLYCANSYN.PWY	Coding	Acetaminophen	4.527	12.805	0.714	0.89
Meconium Adj.[c]	PEPTIDOGLYCANSYN.PWY	Coding	Pathway	108.177	129.708		
Meconium Adj.[c]	PEPTIDOGLYCANSYN.PWY	Coding	Interaction	0.618	0.835		
Meconium Adj.[c]	PEPTIDOGLYCANSYN.PWY	Digit span	Acetaminophen	-11.343	11.017	0.265	0.71
Meconium Adj.[c]	PEPTIDOGLYCANSYN.PWY	Digit span	Pathway	18.18	111.598		
Meconium Adj.[c]	PEPTIDOGLYCANSYN.PWY	Digit span	Interaction	-0.071	0.718		
Meconium Adj.[c]	PEPTIDOGLYCANSYN.PWY	Information	Acetaminophen	-10.699	10.694	0.282	0.725
Meconium Adj.[c]	PEPTIDOGLYCANSYN.PWY	Information	Pathway	-17.493	108.324		
Meconium Adj.[c]	PEPTIDOGLYCANSYN.PWY	Information	Interaction	1.041	0.697		
Meconium Adj.[c]	PEPTIDOGLYCANSYN.PWY	QTAC	Acetaminophen	7.175	37.844	0.772	0.922
Meconium Adj.[c]	PEPTIDOGLYCANSYN.PWY	QTAC	Pathway	122.786	383.333		
Meconium Adj.[c]	PEPTIDOGLYCANSYN.PWY	QTAC	Interaction	-4.19	2.466		
Meconium Adj.[c]	PEPTIDOGLYCANSYN.PWY	Vocabulary	Acetaminophen	-16.363	16.228	0.26	0.71
Meconium Adj.[c]	PEPTIDOGLYCANSYN.PWY	Vocabulary	Pathway	-4.011	164.385		
Meconium Adj.[c]	PEPTIDOGLYCANSYN.PWY	Vocabulary	Interaction	1.09	1.058		
Meconium Adj.[c]	PEPTIDOGLYCANSYN.PWY	WISC sum	Acetaminophen	-43.472	36.685	0.183	0.688
Meconium Adj.[c]	PEPTIDOGLYCANSYN.PWY	WISC sum	Pathway	5.185	371.594		

Exposure Window	Pathway	Outcome	Variable	Effect Estimate[a]	Std. Error	LRT[b]	
						p-value	q-value
Meconium Adj.[c]	PEPTIDOGLYCANSYN.PWY	WISC sum	Interaction	2.795	2.391		
Meconium Adj.[c]	PWY.1042	Block Design	Acetaminophen	-9.106	8.988	0.221	0.704
Meconium Adj.[c]	PWY.1042	Block Design	Pathway	-77.517	78.81		
Meconium Adj.[c]	PWY.1042	Block Design	Interaction	0.042	0.954		
Meconium Adj.[c]	PWY.1042	Coding	Acetaminophen	3.175	7.935	0.689	0.885
Meconium Adj.[c]	PWY.1042	Coding	Pathway	54.108	69.573		
Meconium Adj.[c]	PWY.1042	Coding	Interaction	0.664	0.842		
Meconium Adj.[c]	PWY.1042	Digit span	Acetaminophen	-5.246	6.941	0.42	0.848
Meconium Adj.[c]	PWY.1042	Digit span	Pathway	-2.082	60.86		
Meconium Adj.[c]	PWY.1042	Digit span	Interaction	-0.094	0.736		
Meconium Adj.[c]	PWY.1042	Information	Acetaminophen	6.758	6.624	0.238	0.709
Meconium Adj.[c]	PWY.1042	Information	Pathway	23.919	58.082		
Meconium Adj.[c]	PWY.1042	Information	Interaction	1.013	0.703		
Meconium Adj.[c]	PWY.1042	QTAC	Acetaminophen	-34.299	22.883	0.129	0.688
Meconium Adj.[c]	PWY.1042	QTAC	Pathway	-166.409	200.649		
Meconium Adj.[c]	PWY.1042	QTAC	Interaction	-4.348	2.428		
Meconium Adj.[c]	PWY.1042	Vocabulary	Acetaminophen	0.494	10.266	0.969	0.988
Meconium Adj.[c]	PWY.1042	Vocabulary	Pathway	-20.263	90.011		
Meconium Adj.[c]	PWY.1042	Vocabulary	Interaction	0.988	1.089		
Meconium Adj.[c]	PWY.1042	WISC sum	Acetaminophen	-3.924	23.483	0.823	0.938
Meconium Adj.[c]	PWY.1042	WISC sum	Pathway	-21.835	205.906		
Meconium Adj.[c]	PWY.1042	WISC sum	Interaction	2.613	2.491		
Meconium Adj.[c]	PWY.3841	Block Design	Acetaminophen	-9.986	14.102	0.394	0.808
Meconium Adj.[c]	PWY.3841	Block Design	Pathway	-125.599	185.379		
Meconium Adj.[c]	PWY.3841	Block Design	Interaction	0.044	0.96		
Meconium Adj.[c]	PWY.3841	Coding	Acetaminophen	8.007	12.269	0.486	0.856
Meconium Adj.[c]	PWY.3841	Coding	Pathway	170.592	161.292		
Meconium Adj.[c]	PWY.3841	Coding	Interaction	0.661	0.835		
Meconium Adj.[c]	PWY.3841	Digit span	Acetaminophen	-13.709	10.233	0.147	0.688
Meconium Adj.[c]	PWY.3841	Digit span	Pathway	60.399	134.527		
Meconium Adj.[c]	PWY.3841	Digit span	Interaction	-0.167	0.697		
Meconium Adj.[c]	PWY.3841	Information	Acetaminophen	-18.575	9.866	0.044	0.688
Meconium Adj.[c]	PWY.3841	Information	Pathway	-52.948	129.694		
Meconium Adj.[c]	PWY.3841	Information	Interaction	0.91	0.672		
Meconium Adj.[c]	PWY.3841	QTAC	Acetaminophen	25.112	36.157	0.394	0.808
Meconium Adj.[c]	PWY.3841	QTAC	Pathway	174.108	475.311		

Exposure Window	Pathway	Outcome	Variable	Effect Estimate[a]	Std. Error	LRT[b]	
						p-value	q-value
Meconium Adj.[c]	PWY.3841	QTAC	Interaction	-4.038	2.462		
Meconium Adj.[c]	PWY.3841	Vocabulary	Acetaminophen	-19.819	15.671	0.161	0.688
Meconium Adj.[c]	PWY.3841	Vocabulary	Pathway	-164.833	206.003		
Meconium Adj.[c]	PWY.3841	Vocabulary	Interaction	0.888	1.067		
Meconium Adj.[c]	PWY.3841	WISC sum	Acetaminophen	-54.082	34.839	0.085	0.688
Meconium Adj.[c]	PWY.3841	WISC sum	Pathway	-112.389	457.995		
Meconium Adj.[c]	PWY.3841	WISC sum	Interaction	2.336	2.372		
Meconium Adj.[c]	PWY.4242	Block Design	Acetaminophen	1.63	9.013	0.911	0.961
Meconium Adj.[c]	PWY.4242	Block Design	Pathway	-58.671	101.606		
Meconium Adj.[c]	PWY.4242	Block Design	Interaction	0.192	0.963		
Meconium Adj.[c]	PWY.4242	Coding	Acetaminophen	3.22	7.905	0.688	0.885
Meconium Adj.[c]	PWY.4242	Coding	Pathway	65.998	89.118		
Meconium Adj.[c]	PWY.4242	Coding	Interaction	0.583	0.845		
Meconium Adj.[c]	PWY.4242	Digit span	Acetaminophen	-4.778	6.776	0.437	0.853
Meconium Adj.[c]	PWY.4242	Digit span	Pathway	49.341	76.386		
Meconium Adj.[c]	PWY.4242	Digit span	Interaction	-0.251	0.724		
Meconium Adj.[c]	PWY.4242	Information	Acetaminophen	5.149	6.616	0.355	0.783
Meconium Adj.[c]	PWY.4242	Information	Pathway	6.516	74.586		
Meconium Adj.[c]	PWY.4242	Information	Interaction	1.115	0.707		
Meconium Adj.[c]	PWY.4242	QTAC	Acetaminophen	-27.253	22.967	0.236	0.709
Meconium Adj.[c]	PWY.4242	QTAC	Pathway	-120.83	258.907		
Meconium Adj.[c]	PWY.4242	QTAC	Interaction	-4.617	2.454		
Meconium Adj.[c]	PWY.4242	Vocabulary	Acetaminophen	-0.403	9.979	0.977	0.988
Meconium Adj.[c]	PWY.4242	Vocabulary	Pathway	-125.223	112.498		
Meconium Adj.[c]	PWY.4242	Vocabulary	Interaction	1.115	1.066		
Meconium Adj.[c]	PWY.4242	WISC sum	Acetaminophen	4.818	23.311	0.838	0.938
Meconium Adj.[c]	PWY.4242	WISC sum	Pathway	-62.039	262.79		
Meconium Adj.[c]	PWY.4242	WISC sum	Interaction	2.755	2.49		
Meconium Adj.[c]	PWY.5097	Block Design	Acetaminophen	-10.794	11.483	0.26	0.71
Meconium Adj.[c]	PWY.5097	Block Design	Pathway	-74.616	143.51		
Meconium Adj.[c]	PWY.5097	Block Design	Interaction	-0.017	0.963		
Meconium Adj.[c]	PWY.5097	Coding	Acetaminophen	17.259	9.703	0.057	0.688
Meconium Adj.[c]	PWY.5097	Coding	Pathway	242.737	121.26		
Meconium Adj.[c]	PWY.5097	Coding	Interaction	0.605	0.813		
Meconium Adj.[c]	PWY.5097	Digit span	Acetaminophen	-8.59	8.408	0.264	0.71
Meconium Adj.[c]	PWY.5097	Digit span	Pathway	72.389	105.077		

Exposure Window	Pathway	Outcome	Variable	Effect Estimate[a]	Std. Error	LRT[b]	
						p-value	q-value
Meconium Adj.[c]	PWY.5097	Digit span	Interaction	-0.405	0.705		
Meconium Adj.[c]	PWY.5097	Information	Acetaminophen	-4.267	8.543	0.602	0.885
Meconium Adj.[c]	PWY.5097	Information	Pathway	-5.259	106.764		
Meconium Adj.[c]	PWY.5097	Information	Interaction	0.932	0.716		
Meconium Adj.[c]	PWY.5097	QTAC	Acetaminophen	-0.95	29.853	0.948	0.988
Meconium Adj.[c]	PWY.5097	QTAC	Pathway	77.8	373.09		
Meconium Adj.[c]	PWY.5097	QTAC	Interaction	-4.29	2.502		
Meconium Adj.[c]	PWY.5097	Vocabulary	Acetaminophen	-11.329	12.977	0.328	0.761
Meconium Adj.[c]	PWY.5097	Vocabulary	Pathway	-117.134	162.176		
Meconium Adj.[c]	PWY.5097	Vocabulary	Interaction	0.944	1.088		
Meconium Adj.[c]	PWY.5097	WISC sum	Acetaminophen	-17.72	29.322	0.477	0.853
Meconium Adj.[c]	PWY.5097	WISC sum	Pathway	118.116	366.45		
Meconium Adj.[c]	PWY.5097	WISC sum	Interaction	2.059	2.458		
Meconium Adj.[c]	PWY.5103	Block Design	Acetaminophen	3.873	8.214	0.674	0.885
Meconium Adj.[c]	PWY.5103	Block Design	Pathway	-12.084	95.679		
Meconium Adj.[c]	PWY.5103	Block Design	Interaction	0.175	0.972		
Meconium Adj.[c]	PWY.5103	Coding	Acetaminophen	1.725	7.213	0.81	0.938
Meconium Adj.[c]	PWY.5103	Coding	Pathway	-13.404	84.021		
Meconium Adj.[c]	PWY.5103	Coding	Interaction	0.591	0.854		
Meconium Adj.[c]	PWY.5103	Digit span	Acetaminophen	3.401	6.328	0.522	0.865
Meconium Adj.[c]	PWY.5103	Digit span	Pathway	32.121	73.704		
Meconium Adj.[c]	PWY.5103	Digit span	Interaction	-0.042	0.749		
Meconium Adj.[c]	PWY.5103	Information	Acetaminophen	-1.512	5.923	0.85	0.938
Meconium Adj.[c]	PWY.5103	Information	Pathway	-79.001	68.989		
Meconium Adj.[c]	PWY.5103	Information	Interaction	0.944	0.701		
Meconium Adj.[c]	PWY.5103	QTAC	Acetaminophen	-2.939	21.165	0.999	0.999
Meconium Adj.[c]	PWY.5103	QTAC	Pathway	-63.938	246.534		
Meconium Adj.[c]	PWY.5103	QTAC	Interaction	-4.261	2.505		
Meconium Adj.[c]	PWY.5103	Vocabulary	Acetaminophen	-10.659	8.759	0.189	0.688
Meconium Adj.[c]	PWY.5103	Vocabulary	Pathway	-221.746	102.028		
Meconium Adj.[c]	PWY.5103	Vocabulary	Interaction	0.718	1.037		
Meconium Adj.[c]	PWY.5103	WISC sum	Acetaminophen	-3.171	20.552	0.865	0.944
Meconium Adj.[c]	PWY.5103	WISC sum	Pathway	-294.114	239.388		
Meconium Adj.[c]	PWY.5103	WISC sum	Interaction	2.386	2.433		
Meconium Adj.[c]	PWY.5686	Block Design	Acetaminophen	2.169	12.444	0.908	0.961
Meconium Adj.[c]	PWY.5686	Block Design	Pathway	-40.574	108.849		

Exposure Window	Pathway	Outcome	Variable	Effect Estimate[a]	Std. Error	LRT[b]	
						p-value	q-value
Meconium Adj.[c]	PWY.5686	Block Design	Interaction	0.146	0.962		
Meconium Adj.[c]	PWY.5686	Coding	Acetaminophen	-4.981	10.891	0.594	0.885
Meconium Adj.[c]	PWY.5686	Coding	Pathway	-3.577	95.268		
Meconium Adj.[c]	PWY.5686	Coding	Interaction	0.533	0.842		
Meconium Adj.[c]	PWY.5686	Digit span	Acetaminophen	-9.981	9.3	0.247	0.709
Meconium Adj.[c]	PWY.5686	Digit span	Pathway	33.725	81.352		
Meconium Adj.[c]	PWY.5686	Digit span	Interaction	-0.202	0.719		
Meconium Adj.[c]	PWY.5686	Information	Acetaminophen	1.937	9.193	0.788	0.928
Meconium Adj.[c]	PWY.5686	Information	Pathway	-4.124	80.408		
Meconium Adj.[c]	PWY.5686	Information	Interaction	1.029	0.711		
Meconium Adj.[c]	PWY.5686	QTAC	Acetaminophen	-21.83	31.769	0.507	0.863
Meconium Adj.[c]	PWY.5686	QTAC	Pathway	-220.174	277.893		
Meconium Adj.[c]	PWY.5686	QTAC	Interaction	-4.352	2.456		
Meconium Adj.[c]	PWY.5686	Vocabulary	Acetaminophen	-2.709	13.85	0.819	0.938
Meconium Adj.[c]	PWY.5686	Vocabulary	Pathway	-120.526	121.144		
Meconium Adj.[c]	PWY.5686	Vocabulary	Interaction	1.018	1.071		
Meconium Adj.[c]	PWY.5686	WISC sum	Acetaminophen	-13.565	32.002	0.62	0.885
Meconium Adj.[c]	PWY.5686	WISC sum	Pathway	-135.075	279.93		
Meconium Adj.[c]	PWY.5686	WISC sum	Interaction	2.525	2.474		
Meconium Adj.[c]	PWY.6121	Block Design	Acetaminophen	-7.174	9.449	0.349	0.783
Meconium Adj.[c]	PWY.6121	Block Design	Pathway	-88.613	93.606		
Meconium Adj.[c]	PWY.6121	Block Design	Interaction	-0.01	0.96		
Meconium Adj.[c]	PWY.6121	Coding	Acetaminophen	-1.854	8.36	0.776	0.923
Meconium Adj.[c]	PWY.6121	Coding	Pathway	4.569	82.814		
Meconium Adj.[c]	PWY.6121	Coding	Interaction	0.557	0.85		
Meconium Adj.[c]	PWY.6121	Digit span	Acetaminophen	-12.189	6.831	0.053	0.688
Meconium Adj.[c]	PWY.6121	Digit span	Pathway	13.793	67.671		
Meconium Adj.[c]	PWY.6121	Digit span	Interaction	-0.231	0.694		
Meconium Adj.[c]	PWY.6121	Information	Acetaminophen	-2.539	6.985	0.71	0.89
Meconium Adj.[c]	PWY.6121	Information	Pathway	27.357	69.2		
Meconium Adj.[c]	PWY.6121	Information	Interaction	1.008	0.71		
Meconium Adj.[c]	PWY.6121	QTAC	Acetaminophen	-26.112	24.12	0.289	0.729
Meconium Adj.[c]	PWY.6121	QTAC	Pathway	-273.131	238.935		
Meconium Adj.[c]	PWY.6121	QTAC	Interaction	-4.644	2.451		
Meconium Adj.[c]	PWY.6121	Vocabulary	Acetaminophen	-11.143	10.595	0.236	0.709
Meconium Adj.[c]	PWY.6121	Vocabulary	Pathway	-45.292	104.953		

Exposure Window	Pathway	Outcome	Variable	Effect Estimate[a]	Std. Error	LRT[b]	
						p-value	q-value
Meconium Adj.[c]	PWY.6121	Vocabulary	Interaction	0.886	1.077		
Meconium Adj.[c]	PWY.6121	WISC sum	Acetaminophen	-34.899	23.738	0.097	0.688
Meconium Adj.[c]	PWY.6121	WISC sum	Pathway	-88.186	235.157		
Meconium Adj.[c]	PWY.6121	WISC sum	Interaction	2.21	2.413		
Meconium Adj.[c]	PWY.6122	Block Design	Acetaminophen	-9.792	10.645	0.271	0.712
Meconium Adj.[c]	PWY.6122	Block Design	Pathway	-128.504	116.97		
Meconium Adj.[c]	PWY.6122	Block Design	Interaction	-0.015	0.956		
Meconium Adj.[c]	PWY.6122	Coding	Acetaminophen	-3.469	9.442	0.659	0.885
Meconium Adj.[c]	PWY.6122	Coding	Pathway	-15.262	103.748		
Meconium Adj.[c]	PWY.6122	Coding	Interaction	0.536	0.848		
Meconium Adj.[c]	PWY.6122	Digit span	Acetaminophen	-11.184	7.762	0.115	0.688
Meconium Adj.[c]	PWY.6122	Digit span	Pathway	14.17	85.286		
Meconium Adj.[c]	PWY.6122	Digit span	Interaction	-0.211	0.697		
Meconium Adj.[c]	PWY.6122	Information	Acetaminophen	-2.876	7.927	0.71	0.89
Meconium Adj.[c]	PWY.6122	Information	Pathway	10.077	87.104		
Meconium Adj.[c]	PWY.6122	Information	Interaction	0.993	0.712		
Meconium Adj.[c]	PWY.6122	QTAC	Acetaminophen	-16.589	27.606	0.582	0.885
Meconium Adj.[c]	PWY.6122	QTAC	Pathway	-184.599	303.343		
Meconium Adj.[c]	PWY.6122	QTAC	Interaction	-4.418	2.478		
Meconium Adj.[c]	PWY.6122	Vocabulary	Acetaminophen	-13.411	11.902	0.205	0.688
Meconium Adj.[c]	PWY.6122	Vocabulary	Pathway	-57.057	130.78		
Meconium Adj.[c]	PWY.6122	Vocabulary	Interaction	0.888	1.068		
Meconium Adj.[c]	PWY.6122	WISC sum	Acetaminophen	-40.733	26.722	0.086	0.688
Meconium Adj.[c]	PWY.6122	WISC sum	Pathway	-176.576	293.626		
Meconium Adj.[c]	PWY.6122	WISC sum	Interaction	2.19	2.399		
Meconium Adj.[c]	PWY.6151	Block Design	Acetaminophen	-6.688	8.593	0.329	0.761
Meconium Adj.[c]	PWY.6151	Block Design	Pathway	-83.82	96.119		
Meconium Adj.[c]	PWY.6151	Block Design	Interaction	0.136	0.951		
Meconium Adj.[c]	PWY.6151	Coding	Acetaminophen	7.321	7.485	0.297	0.74
Meconium Adj.[c]	PWY.6151	Coding	Pathway	94.924	83.726		
Meconium Adj.[c]	PWY.6151	Coding	Interaction	0.577	0.828		
Meconium Adj.[c]	PWY.6151	Digit span	Acetaminophen	-7.623	6.552	0.213	0.697
Meconium Adj.[c]	PWY.6151	Digit span	Pathway	-32.219	73.285		
Meconium Adj.[c]	PWY.6151	Digit span	Interaction	-0.1	0.725		
Meconium Adj.[c]	PWY.6151	Information	Acetaminophen	0.755	6.333	0.852	0.938
Meconium Adj.[c]	PWY.6151	Information	Pathway	-38.345	70.837		

Exposure Window	Pathway	Outcome	Variable	Effect Estimate[a]	Std. Error	LRT[b]	
						p-value	q-value
Meconium Adj.[c]	PWY.6151	Information	Interaction	0.992	0.701		
Meconium Adj.[c]	PWY.6151	QTAC	Acetaminophen	-2.46	22.286	0.988	0.992
Meconium Adj.[c]	PWY.6151	QTAC	Pathway	-14.605	249.276		
Meconium Adj.[c]	PWY.6151	QTAC	Interaction	-4.227	2.466		
Meconium Adj.[c]	PWY.6151	Vocabulary	Acetaminophen	-6.148	9.685	0.47	0.853
Meconium Adj.[c]	PWY.6151	Vocabulary	Pathway	-94.988	108.327		
Meconium Adj.[c]	PWY.6151	Vocabulary	Interaction	1.024	1.072		
Meconium Adj.[c]	PWY.6151	WISC sum	Acetaminophen	-12.384	22.226	0.511	0.864
Meconium Adj.[c]	PWY.6151	WISC sum	Pathway	-154.448	248.605		
Meconium Adj.[c]	PWY.6151	WISC sum	Interaction	2.63	2.459		
Meconium Adj.[c]	PWY.6163	Block Design	Acetaminophen	-11.667	9.488	0.147	0.688
Meconium Adj.[c]	PWY.6163	Block Design	Pathway	-124.275	90.827		
Meconium Adj.[c]	PWY.6163	Block Design	Interaction	0.07	0.945		
Meconium Adj.[c]	PWY.6163	Coding	Acetaminophen	3.154	8.401	0.71	0.89
Meconium Adj.[c]	PWY.6163	Coding	Pathway	75.758	80.421		
Meconium Adj.[c]	PWY.6163	Coding	Interaction	0.686	0.837		
Meconium Adj.[c]	PWY.6163	Digit span	Acetaminophen	-9.052	7.269	0.178	0.688
Meconium Adj.[c]	PWY.6163	Digit span	Pathway	-18.831	69.586		
Meconium Adj.[c]	PWY.6163	Digit span	Interaction	-0.024	0.724		
Meconium Adj.[c]	PWY.6163	Information	Acetaminophen	-0.498	7.155	0.978	0.988
Meconium Adj.[c]	PWY.6163	Information	Pathway	-22.903	68.495		
Meconium Adj.[c]	PWY.6163	Information	Interaction	0.967	0.713		
Meconium Adj.[c]	PWY.6163	QTAC	Acetaminophen	-31.235	24.539	0.202	0.688
Meconium Adj.[c]	PWY.6163	QTAC	Pathway	-235.363	234.908		
Meconium Adj.[c]	PWY.6163	QTAC	Interaction	-4.265	2.445		
Meconium Adj.[c]	PWY.6163	Vocabulary	Acetaminophen	-13.874	10.718	0.15	0.688
Meconium Adj.[c]	PWY.6163	Vocabulary	Pathway	-111.714	102.603		
Meconium Adj.[c]	PWY.6163	Vocabulary	Interaction	1.017	1.068		
Meconium Adj.[c]	PWY.6163	WISC sum	Acetaminophen	-31.936	24.472	0.141	0.688
Meconium Adj.[c]	PWY.6163	WISC sum	Pathway	-201.965	234.263		
Meconium Adj.[c]	PWY.6163	WISC sum	Interaction	2.717	2.438		
Meconium Adj.[c]	PWY.6277	Block Design	Acetaminophen	-9.792	10.645	0.271	0.712
Meconium Adj.[c]	PWY.6277	Block Design	Pathway	-128.504	116.97		
Meconium Adj.[c]	PWY.6277	Block Design	Interaction	-0.015	0.956		
Meconium Adj.[c]	PWY.6277	Coding	Acetaminophen	-3.469	9.442	0.659	0.885
Meconium Adj.[c]	PWY.6277	Coding	Pathway	-15.262	103.748		

Exposure Window	Pathway	Outcome	Variable	Effect Estimate[a]	Std. Error	LRT[b]	
						p-value	q-value
Meconium Adj.[c]	PWY.6277	Coding	Interaction	0.536	0.848		
Meconium Adj.[c]	PWY.6277	Digit span	Acetaminophen	-11.184	7.762	0.115	0.688
Meconium Adj.[c]	PWY.6277	Digit span	Pathway	14.17	85.286		
Meconium Adj.[c]	PWY.6277	Digit span	Interaction	-0.211	0.697		
Meconium Adj.[c]	PWY.6277	Information	Acetaminophen	-2.876	7.927	0.71	0.89
Meconium Adj.[c]	PWY.6277	Information	Pathway	10.077	87.104		
Meconium Adj.[c]	PWY.6277	Information	Interaction	0.993	0.712		
Meconium Adj.[c]	PWY.6277	QTAC	Acetaminophen	-16.589	27.606	0.582	0.885
Meconium Adj.[c]	PWY.6277	QTAC	Pathway	-184.599	303.343		
Meconium Adj.[c]	PWY.6277	QTAC	Interaction	-4.418	2.478		
Meconium Adj.[c]	PWY.6277	Vocabulary	Acetaminophen	-13.411	11.902	0.205	0.688
Meconium Adj.[c]	PWY.6277	Vocabulary	Pathway	-57.057	130.78		
Meconium Adj.[c]	PWY.6277	Vocabulary	Interaction	0.888	1.068		
Meconium Adj.[c]	PWY.6277	WISC sum	Acetaminophen	-40.733	26.722	0.086	0.688
Meconium Adj.[c]	PWY.6277	WISC sum	Pathway	-176.576	293.626		
Meconium Adj.[c]	PWY.6277	WISC sum	Interaction	2.19	2.399		
Meconium Adj.[c]	PWY.6385	Block Design	Acetaminophen	-4.716	12.872	0.63	0.885
Meconium Adj.[c]	PWY.6385	Block Design	Pathway	-53.442	137.475		
Meconium Adj.[c]	PWY.6385	Block Design	Interaction	0.143	0.959		
Meconium Adj.[c]	PWY.6385	Coding	Acetaminophen	14.893	10.863	0.142	0.688
Meconium Adj.[c]	PWY.6385	Coding	Pathway	208.545	116.017		
Meconium Adj.[c]	PWY.6385	Coding	Interaction	0.524	0.809		
Meconium Adj.[c]	PWY.6385	Digit span	Acetaminophen	-3.779	9.654	0.675	0.885
Meconium Adj.[c]	PWY.6385	Digit span	Pathway	87.182	103.105		
Meconium Adj.[c]	PWY.6385	Digit span	Interaction	-0.154	0.719		
Meconium Adj.[c]	PWY.6385	Information	Acetaminophen	-10.068	9.324	0.245	0.709
Meconium Adj.[c]	PWY.6385	Information	Pathway	-14.192	99.575		
Meconium Adj.[c]	PWY.6385	Information	Interaction	1.005	0.695		
Meconium Adj.[c]	PWY.6385	QTAC	Acetaminophen	4.101	32.978	0.821	0.938
Meconium Adj.[c]	PWY.6385	QTAC	Pathway	183.781	352.198		
Meconium Adj.[c]	PWY.6385	QTAC	Interaction	-4.271	2.457		
Meconium Adj.[c]	PWY.6385	Vocabulary	Acetaminophen	-16.552	14.273	0.198	0.688
Meconium Adj.[c]	PWY.6385	Vocabulary	Pathway	-136.477	152.433		
Meconium Adj.[c]	PWY.6385	Vocabulary	Interaction	1.055	1.064		
Meconium Adj.[c]	PWY.6385	WISC sum	Acetaminophen	-20.222	32.681	0.471	0.853
Meconium Adj.[c]	PWY.6385	WISC sum	Pathway	91.617	349.025		

Exposure Window	Pathway	Outcome	Variable	Effect Estimate[a]	Std. Error	LRT[b] p-value	LRT[b] q-value
Meconium Adj.[c]	PWY.6385	WISC sum	Interaction	2.573	2.435		
Meconium Adj.[c]	PWY.6386	Block Design	Acetaminophen	-10.465	14.093		
Meconium Adj.[c]	PWY.6386	Block Design	Pathway	-88.782	134.561	0.374	0.793
Meconium Adj.[c]	PWY.6386	Block Design	Interaction	0.06	0.957		
Meconium Adj.[c]	PWY.6386	Coding	Acetaminophen	2.948	12.299		
Meconium Adj.[c]	PWY.6386	Coding	Pathway	97.075	117.433	0.813	0.938
Meconium Adj.[c]	PWY.6386	Coding	Interaction	0.611	0.835		
Meconium Adj.[c]	PWY.6386	Digit span	Acetaminophen	-13.599	10.393		
Meconium Adj.[c]	PWY.6386	Digit span	Pathway	23.117	99.235	0.155	0.688
Meconium Adj.[c]	PWY.6386	Digit span	Interaction	-0.175	0.706		
Meconium Adj.[c]	PWY.6386	Information	Acetaminophen	-11.524	10.305		
Meconium Adj.[c]	PWY.6386	Information	Pathway	-49.812	98.4	0.229	0.709
Meconium Adj.[c]	PWY.6386	Information	Interaction	0.949	0.7		
Meconium Adj.[c]	PWY.6386	QTAC	Acetaminophen	-8.454	36.377		
Meconium Adj.[c]	PWY.6386	QTAC	Pathway	52.158	347.341	0.86	0.942
Meconium Adj.[c]	PWY.6386	QTAC	Interaction	-4.232	2.471		
Meconium Adj.[c]	PWY.6386	Vocabulary	Acetaminophen	-22.623	15.568		
Meconium Adj.[c]	PWY.6386	Vocabulary	Pathway	-131.958	148.652	0.109	0.688
Meconium Adj.[c]	PWY.6386	Vocabulary	Interaction	0.896	1.058		
Meconium Adj.[c]	PWY.6386	WISC sum	Acetaminophen	-55.264	35.062		
Meconium Adj.[c]	PWY.6386	WISC sum	Pathway	-150.36	334.778	0.08	0.688
Meconium Adj.[c]	PWY.6386	WISC sum	Interaction	2.34	2.382		
Meconium Adj.[c]	PWY.6387	Block Design	Acetaminophen	-9.662	15.145		
Meconium Adj.[c]	PWY.6387	Block Design	Pathway	-93.893	144.634	0.442	0.853
Meconium Adj.[c]	PWY.6387	Block Design	Interaction	0.093	0.956		
Meconium Adj.[c]	PWY.6387	Coding	Acetaminophen	2.771	13.215		
Meconium Adj.[c]	PWY.6387	Coding	Pathway	98.097	126.203	0.837	0.938
Meconium Adj.[c]	PWY.6387	Coding	Interaction	0.611	0.834		
Meconium Adj.[c]	PWY.6387	Digit span	Acetaminophen	-13.29	11.244		
Meconium Adj.[c]	PWY.6387	Digit span	Pathway	28.446	107.376	0.199	0.688
Meconium Adj.[c]	PWY.6387	Digit span	Interaction	-0.119	0.71		
Meconium Adj.[c]	PWY.6387	Information	Acetaminophen	-10.486	11.12		
Meconium Adj.[c]	PWY.6387	Information	Pathway	-55.212	106.191	0.311	0.74
Meconium Adj.[c]	PWY.6387	Information	Interaction	0.986	0.702		
Meconium Adj.[c]	PWY.6387	QTAC	Acetaminophen	-6.401	39.042	0.916	0.961
Meconium Adj.[c]	PWY.6387	QTAC	Pathway	73.641	372.853		

Exposure Window	Pathway	Outcome	Variable	Effect Estimate[a]	Std. Error	LRT[b]	
						p-value	q-value
Meconium Adj.[c]	PWY.6387	QTAC	Interaction	-4.196	2.465		
Meconium Adj.[c]	PWY.6387	Vocabulary	Acetaminophen	-23.248	16.743	0.125	0.688
Meconium Adj.[c]	PWY.6387	Vocabulary	Pathway	-130.945	159.892		
Meconium Adj.[c]	PWY.6387	Vocabulary	Interaction	0.971	1.057		
Meconium Adj.[c]	PWY.6387	WISC sum	Acetaminophen	-53.914	37.959	0.113	0.688
Meconium Adj.[c]	PWY.6387	WISC sum	Pathway	-153.506	362.507		
Meconium Adj.[c]	PWY.6387	WISC sum	Interaction	2.542	2.397		
Meconium Adj.[c]	PWY.6609	Block Design	Acetaminophen	-0.163	7.79	0.889	0.954
Meconium Adj.[c]	PWY.6609	Block Design	Pathway	-79.431	81.812		
Meconium Adj.[c]	PWY.6609	Block Design	Interaction	0.152	0.951		
Meconium Adj.[c]	PWY.6609	Coding	Acetaminophen	9.883	6.741	0.117	0.688
Meconium Adj.[c]	PWY.6609	Coding	Pathway	97.823	70.789		
Meconium Adj.[c]	PWY.6609	Coding	Interaction	0.439	0.823		
Meconium Adj.[c]	PWY.6609	Digit span	Acetaminophen	-1.982	6.074	0.747	0.91
Meconium Adj.[c]	PWY.6609	Digit span	Pathway	6.417	63.788		
Meconium Adj.[c]	PWY.6609	Digit span	Interaction	-0.099	0.741		
Meconium Adj.[c]	PWY.6609	Information	Acetaminophen	1.964	5.831	0.668	0.885
Meconium Adj.[c]	PWY.6609	Information	Pathway	21.023	61.231		
Meconium Adj.[c]	PWY.6609	Information	Interaction	0.98	0.712		
Meconium Adj.[c]	PWY.6609	QTAC	Acetaminophen	-15.6	20.195	0.482	0.855
Meconium Adj.[c]	PWY.6609	QTAC	Pathway	-146.845	212.079		
Meconium Adj.[c]	PWY.6609	QTAC	Interaction	-4.038	2.464		
Meconium Adj.[c]	PWY.6609	Vocabulary	Acetaminophen	4.285	8.868	0.603	0.885
Meconium Adj.[c]	PWY.6609	Vocabulary	Pathway	-5.066	93.13		
Meconium Adj.[c]	PWY.6609	Vocabulary	Interaction	0.976	1.082		
Meconium Adj.[c]	PWY.6609	WISC sum	Acetaminophen	13.988	20.247	0.467	0.853
Meconium Adj.[c]	PWY.6609	WISC sum	Pathway	40.766	212.623		
Meconium Adj.[c]	PWY.6609	WISC sum	Interaction	2.446	2.471		
Meconium Adj.[c]	PWY.6737	Block Design	Acetaminophen	-6.64	8.706	0.366	0.793
Meconium Adj.[c]	PWY.6737	Block Design	Pathway	-147.008	82.12		
Meconium Adj.[c]	PWY.6737	Block Design	Interaction	-0.141	0.932		
Meconium Adj.[c]	PWY.6737	Coding	Acetaminophen	-2.121	7.858	0.713	0.89
Meconium Adj.[c]	PWY.6737	Coding	Pathway	33.317	74.117		
Meconium Adj.[c]	PWY.6737	Coding	Interaction	0.64	0.841		
Meconium Adj.[c]	PWY.6737	Digit span	Acetaminophen	-3.034	6.928	0.637	0.885
Meconium Adj.[c]	PWY.6737	Digit span	Pathway	14.187	65.344		

Exposure Window	Pathway	Outcome	Variable	Effect Estimate[a]	Std. Error	LRT[b]	
						p-value	q-value
Meconium Adj.[c]	PWY.6737	Digit span	Interaction	-0.093	0.741		
Meconium Adj.[c]	PWY.6737	Information	Acetaminophen	5.286	6.612	0.34	0.772
Meconium Adj.[c]	PWY.6737	Information	Pathway	11.702	62.37		
Meconium Adj.[c]	PWY.6737	Information	Interaction	1.029	0.708		
Meconium Adj.[c]	PWY.6737	QTAC	Acetaminophen	-47.119	22.112	0.028	0.688
Meconium Adj.[c]	PWY.6737	QTAC	Pathway	-215.879	208.564		
Meconium Adj.[c]	PWY.6737	QTAC	Interaction	-4.593	2.366		
Meconium Adj.[c]	PWY.6737	Vocabulary	Acetaminophen	-4.221	10.192	0.641	0.885
Meconium Adj.[c]	PWY.6737	Vocabulary	Pathway	-59.075	96.135		
Meconium Adj.[c]	PWY.6737	Vocabulary	Interaction	0.922	1.091		
Meconium Adj.[c]	PWY.6737	WISC sum	Acetaminophen	-10.73	23.273	0.592	0.885
Meconium Adj.[c]	PWY.6737	WISC sum	Pathway	-146.877	219.521		
Meconium Adj.[c]	PWY.6737	WISC sum	Interaction	2.357	2.49		
Meconium Adj.[c]	PWY.7111	Block Design	Acetaminophen	-5.401	9.268	0.456	0.853
Meconium Adj.[c]	PWY.7111	Block Design	Pathway	-63.786	89.336		
Meconium Adj.[c]	PWY.7111	Block Design	Interaction	0.032	0.975		
Meconium Adj.[c]	PWY.7111	Coding	Acetaminophen	1.623	8.168	0.853	0.938
Meconium Adj.[c]	PWY.7111	Coding	Pathway	12.679	78.725		
Meconium Adj.[c]	PWY.7111	Coding	Interaction	0.592	0.859		
Meconium Adj.[c]	PWY.7111	Digit span	Acetaminophen	-3.152	6.984	0.619	0.885
Meconium Adj.[c]	PWY.7111	Digit span	Pathway	38.571	67.319		
Meconium Adj.[c]	PWY.7111	Digit span	Interaction	0.056	0.734		
Meconium Adj.[c]	PWY.7111	Information	Acetaminophen	-3.05	6.625	0.674	0.885
Meconium Adj.[c]	PWY.7111	Information	Pathway	-90.111	63.856		
Meconium Adj.[c]	PWY.7111	Information	Interaction	0.763	0.697		
Meconium Adj.[c]	PWY.7111	QTAC	Acetaminophen	-16.643	23.831	0.516	0.865
Meconium Adj.[c]	PWY.7111	QTAC	Pathway	-89.071	229.704		
Meconium Adj.[c]	PWY.7111	QTAC	Interaction	-4.271	2.506		
Meconium Adj.[c]	PWY.7111	Vocabulary	Acetaminophen	-18.464	10.012	0.044	0.688
Meconium Adj.[c]	PWY.7111	Vocabulary	Pathway	-182.936	96.502		
Meconium Adj.[c]	PWY.7111	Vocabulary	Interaction	0.754	1.053		
Meconium Adj.[c]	PWY.7111	WISC sum	Acetaminophen	-28.444	23.481	0.174	0.688
Meconium Adj.[c]	PWY.7111	WISC sum	Pathway	-285.582	226.33		
Meconium Adj.[c]	PWY.7111	WISC sum	Interaction	2.197	2.469		
Meconium Adj.[c]	PWY.7219	Block Design	Acetaminophen	4.628	9.903	0.671	0.885
Meconium Adj.[c]	PWY.7219	Block Design	Pathway	-21.726	85.545		

Exposure Window	Pathway	Outcome	Variable	Effect Estimate[a]	Std. Error	LRT[b]	
						p-value	q-value
Meconium Adj.[c]	PWY.7219	Block Design	Interaction	0.175	0.957		
Meconium Adj.[c]	PWY.7219	Coding	Acetaminophen	-4.08	8.593	0.569	0.885
Meconium Adj.[c]	PWY.7219	Coding	Pathway	35.698	74.227		
Meconium Adj.[c]	PWY.7219	Coding	Interaction	0.517	0.83		
Meconium Adj.[c]	PWY.7219	Digit span	Acetaminophen	-3.916	7.343	0.566	0.885
Meconium Adj.[c]	PWY.7219	Digit span	Pathway	71.287	63.433		
Meconium Adj.[c]	PWY.7219	Digit span	Interaction	-0.202	0.709		
Meconium Adj.[c]	PWY.7219	Information	Acetaminophen	-6.659	7.278	0.333	0.762
Meconium Adj.[c]	PWY.7219	Information	Pathway	-34.254	62.863		
Meconium Adj.[c]	PWY.7219	Information	Interaction	0.992	0.703		
Meconium Adj.[c]	PWY.7219	QTAC	Acetaminophen	-15.062	25.475	0.593	0.885
Meconium Adj.[c]	PWY.7219	QTAC	Pathway	-105.458	220.056		
Meconium Adj.[c]	PWY.7219	QTAC	Interaction	-4.229	2.461		
Meconium Adj.[c]	PWY.7219	Vocabulary	Acetaminophen	-9.201	11.059	0.351	0.783
Meconium Adj.[c]	PWY.7219	Vocabulary	Pathway	-98.73	95.525		
Meconium Adj.[c]	PWY.7219	Vocabulary	Interaction	1.038	1.068		
Meconium Adj.[c]	PWY.7219	WISC sum	Acetaminophen	-19.229	25.398	0.381	0.793
Meconium Adj.[c]	PWY.7219	WISC sum	Pathway	-47.726	219.391		
Meconium Adj.[c]	PWY.7219	WISC sum	Interaction	2.52	2.453		
Meconium Adj.[c]	PWY.7221	Block Design	Acetaminophen	5.995	11.544	0.624	0.885
Meconium Adj.[c]	PWY.7221	Block Design	Pathway	19.696	133.532		
Meconium Adj.[c]	PWY.7221	Block Design	Interaction	0.162	0.963		
Meconium Adj.[c]	PWY.7221	Coding	Acetaminophen	5.554	10.09	0.56	0.885
Meconium Adj.[c]	PWY.7221	Coding	Pathway	18.057	116.722		
Meconium Adj.[c]	PWY.7221	Coding	Interaction	0.608	0.842		
Meconium Adj.[c]	PWY.7221	Digit span	Acetaminophen	-4.337	8.769	0.606	0.885
Meconium Adj.[c]	PWY.7221	Digit span	Pathway	32.954	101.441		
Meconium Adj.[c]	PWY.7221	Digit span	Interaction	-0.202	0.732		
Meconium Adj.[c]	PWY.7221	Information	Acetaminophen	-0.647	8.509	0.962	0.988
Meconium Adj.[c]	PWY.7221	Information	Pathway	-37.48	98.426		
Meconium Adj.[c]	PWY.7221	Information	Interaction	1.051	0.71		
Meconium Adj.[c]	PWY.7221	QTAC	Acetaminophen	-10.762	29.412	0.761	0.918
Meconium Adj.[c]	PWY.7221	QTAC	Pathway	-264.992	340.22		
Meconium Adj.[c]	PWY.7221	QTAC	Interaction	-3.994	2.455		
Meconium Adj.[c]	PWY.7221	Vocabulary	Acetaminophen	-16.712	12.751	0.146	0.688
Meconium Adj.[c]	PWY.7221	Vocabulary	Pathway	-159.841	147.495		

Exposure Window	Pathway	Outcome	Variable	Effect Estimate[a]	Std. Error	LRT[b]	
						p-value	q-value
Meconium Adj.[c]	PWY.7221	Vocabulary	Interaction	1.043	1.064		
Meconium Adj.[c]	PWY.7221	WISC sum	Acetaminophen	-10.146	29.727	0.683	0.885
Meconium Adj.[c]	PWY.7221	WISC sum	Pathway	-126.615	343.867		
Meconium Adj.[c]	PWY.7221	WISC sum	Interaction	2.662	2.481		
Meconium Adj.[c]	PWY.7400	Block Design	Acetaminophen	-5.785	9.622	0.445	0.853
Meconium Adj.[c]	PWY.7400	Block Design	Pathway	-78.392	101.511		
Meconium Adj.[c]	PWY.7400	Block Design	Interaction	0.062	0.961		
Meconium Adj.[c]	PWY.7400	Coding	Acetaminophen	-7.343	8.337	0.308	0.74
Meconium Adj.[c]	PWY.7400	Coding	Pathway	-13.64	87.951		
Meconium Adj.[c]	PWY.7400	Coding	Interaction	0.621	0.833		
Meconium Adj.[c]	PWY.7400	Digit span	Acetaminophen	-12.46	6.945	0.052	0.688
Meconium Adj.[c]	PWY.7400	Digit span	Pathway	-14.348	73.269		
Meconium Adj.[c]	PWY.7400	Digit span	Interaction	-0.035	0.694		
Meconium Adj.[c]	PWY.7400	Information	Acetaminophen	-4.598	7.107	0.504	0.863
Meconium Adj.[c]	PWY.7400	Information	Pathway	-49.256	74.979		
Meconium Adj.[c]	PWY.7400	Information	Interaction	0.968	0.71		
Meconium Adj.[c]	PWY.7400	QTAC	Acetaminophen	-32.972	24.404	0.172	0.688
Meconium Adj.[c]	PWY.7400	QTAC	Pathway	-230.253	257.463		
Meconium Adj.[c]	PWY.7400	QTAC	Interaction	-4.329	2.437		
Meconium Adj.[c]	PWY.7400	Vocabulary	Acetaminophen	-13.618	10.678	0.154	0.688
Meconium Adj.[c]	PWY.7400	Vocabulary	Pathway	-88.756	112.654		
Meconium Adj.[c]	PWY.7400	Vocabulary	Interaction	1.008	1.066		
Meconium Adj.[c]	PWY.7400	WISC sum	Acetaminophen	-43.804	23.776	0.04	0.688
Meconium Adj.[c]	PWY.7400	WISC sum	Pathway	-244.392	250.842		
Meconium Adj.[c]	PWY.7400	WISC sum	Interaction	2.624	2.374		
Meconium Adj.[c]	TRNA.CHARGING.PWY	Block Design	Acetaminophen	-8.05	13.891	0.477	0.853
Meconium Adj.[c]	TRNA.CHARGING.PWY	Block Design	Pathway	-117.44	144.167		
Meconium Adj.[c]	TRNA.CHARGING.PWY	Block Design	Interaction	0.14	0.954		
Meconium Adj.[c]	TRNA.CHARGING.PWY	Coding	Acetaminophen	-10.632	11.89	0.311	0.74
Meconium Adj.[c]	TRNA.CHARGING.PWY	Coding	Pathway	47.435	123.403		
Meconium Adj.[c]	TRNA.CHARGING.PWY	Coding	Interaction	0.638	0.817		
Meconium Adj.[c]	TRNA.CHARGING.PWY	Digit span	Acetaminophen	-14.218	10.243	0.134	0.688
Meconium Adj.[c]	TRNA.CHARGING.PWY	Digit span	Pathway	26.329	106.311		
Meconium Adj.[c]	TRNA.CHARGING.PWY	Digit span	Interaction	-0.05	0.704		
Meconium Adj.[c]	TRNA.CHARGING.PWY	Information	Acetaminophen	-19.353	9.809	0.035	0.688
Meconium Adj.[c]	TRNA.CHARGING.PWY	Information	Pathway	-74.405	101.801		

Exposure Window	Pathway	Outcome	Variable	Effect Estimate[a]	Std. Error	LRT[b]	
						p-value	q-value
Meconium Adj.[c]	TRNA.CHARGING.PWY	Information	Interaction	1.078	0.674		
Meconium Adj.[c]	TRNA.CHARGING.PWY	QTAC	Acetaminophen	-13.417	35.682	0.739	0.907
Meconium Adj.[c]	TRNA.CHARGING.PWY	QTAC	Pathway	118.899	370.332		
Meconium Adj.[c]	TRNA.CHARGING.PWY	QTAC	Interaction	-4.137	2.451		
Meconium Adj.[c]	TRNA.CHARGING.PWY	Vocabulary	Acetaminophen	-19.647	15.397	0.157	0.688
Meconium Adj.[c]	TRNA.CHARGING.PWY	Vocabulary	Pathway	-74.242	159.794		
Meconium Adj.[c]	TRNA.CHARGING.PWY	Vocabulary	Interaction	1.1	1.058		
Meconium Adj.[c]	TRNA.CHARGING.PWY	WISC sum	Acetaminophen	-71.9	33.568	0.019	0.688
Meconium Adj.[c]	TRNA.CHARGING.PWY	WISC sum	Pathway	-192.323	348.392		
Meconium Adj.[c]	TRNA.CHARGING.PWY	WISC sum	Interaction	2.905	2.306		
Meconium Adj.[c]	UNINTEGRATED	Block Design	Acetaminophen	-10.44	30.797	0.682	0.885
Meconium Adj.[c]	UNINTEGRATED	Block Design	Pathway	0.008	0.337		
Meconium Adj.[c]	UNINTEGRATED	Block Design	Interaction	0.138	0.956		
Meconium Adj.[c]	UNINTEGRATED	Coding	Acetaminophen	15.26	26.93	0.537	0.878
Meconium Adj.[c]	UNINTEGRATED	Coding	Pathway	0.051	0.295		
Meconium Adj.[c]	UNINTEGRATED	Coding	Interaction	0.568	0.836		
Meconium Adj.[c]	UNINTEGRATED	Digit span	Acetaminophen	-0.738	23.766	0.981	0.988
Meconium Adj.[c]	UNINTEGRATED	Digit span	Pathway	0.044	0.26		
Meconium Adj.[c]	UNINTEGRATED	Digit span	Interaction	-0.122	0.738		
Meconium Adj.[c]	UNINTEGRATED	Information	Acetaminophen	34.41	21.761	0.081	0.688
Meconium Adj.[c]	UNINTEGRATED	Information	Pathway	0.134	0.238		
Meconium Adj.[c]	UNINTEGRATED	Information	Interaction	0.996	0.676		
Meconium Adj.[c]	UNINTEGRATED	QTAC	Acetaminophen	-148.583	75.66	0.037	0.688
Meconium Adj.[c]	UNINTEGRATED	QTAC	Pathway	-1.573	0.829		
Meconium Adj.[c]	UNINTEGRATED	QTAC	Interaction	-4.162	2.349		
Meconium Adj.[c]	UNINTEGRATED	Vocabulary	Acetaminophen	-1.654	34.833	0.954	0.988
Meconium Adj.[c]	UNINTEGRATED	Vocabulary	Pathway	-0.023	0.381		
Meconium Adj.[c]	UNINTEGRATED	Vocabulary	Interaction	1.031	1.081		
Meconium Adj.[c]	UNINTEGRATED	WISC sum	Acetaminophen	36.838	79.369	0.615	0.885
Meconium Adj.[c]	UNINTEGRATED	WISC sum	Pathway	0.214	0.869		
Meconium Adj.[c]	UNINTEGRATED	WISC sum	Interaction	2.611	2.464		
Meconium Adj.[c]	UNMAPPED	Block Design	Acetaminophen	4.462	8.827	0.661	0.885
Meconium Adj.[c]	UNMAPPED	Block Design	Pathway	-0.009	0.313		
Meconium Adj.[c]	UNMAPPED	Block Design	Interaction	0.125	0.955		
Meconium Adj.[c]	UNMAPPED	Coding	Acetaminophen	-4.298	7.727	0.518	0.865
Meconium Adj.[c]	UNMAPPED	Coding	Pathway	-0.061	0.274		

Exposure Window	Pathway	Outcome	Variable	Effect Estimate[a]	Std. Error	LRT[b]	
						p-value	q-value
Meconium Adj.[c]	UNMAPPED	Coding	Interaction	0.578	0.836		
Meconium Adj.[c]	UNMAPPED	Digit span	Acetaminophen	0.667	6.814	0.887	0.954
Meconium Adj.[c]	UNMAPPED	Digit span	Pathway	-0.025	0.241		
Meconium Adj.[c]	UNMAPPED	Digit span	Interaction	-0.126	0.738		
Meconium Adj.[c]	UNMAPPED	Information	Acetaminophen	-10.084	6.243	0.089	0.688
Meconium Adj.[c]	UNMAPPED	Information	Pathway	-0.108	0.221		
Meconium Adj.[c]	UNMAPPED	Information	Interaction	1.021	0.676		
Meconium Adj.[c]	UNMAPPED	QTAC	Acetaminophen	35.593	21.877	0.054	0.688
Meconium Adj.[c]	UNMAPPED	QTAC	Pathway	1.316	0.775		
Meconium Adj.[c]	UNMAPPED	QTAC	Interaction	-4.175	2.368		
Meconium Adj.[c]	UNMAPPED	Vocabulary	Acetaminophen	1.638	9.988	0.869	0.944
Meconium Adj.[c]	UNMAPPED	Vocabulary	Pathway	0.064	0.354		
Meconium Adj.[c]	UNMAPPED	Vocabulary	Interaction	1.033	1.081		
Meconium Adj.[c]	UNMAPPED	WISC sum	Acetaminophen	-7.615	22.786	0.684	0.885
Meconium Adj.[c]	UNMAPPED	WISC sum	Pathway	-0.139	0.807		
Meconium Adj.[c]	UNMAPPED	WISC sum	Interaction	2.63	2.466		
Meconium Adj.[c]	VALSYN.PWY	Block Design	Acetaminophen	-4.946	9.457	0.496	0.862
Meconium Adj.[c]	VALSYN.PWY	Block Design	Pathway	-52.793	91.813		
Meconium Adj.[c]	VALSYN.PWY	Block Design	Interaction	0.065	0.975		
Meconium Adj.[c]	VALSYN.PWY	Coding	Acetaminophen	-0.01	8.322	0.973	0.988
Meconium Adj.[c]	VALSYN.PWY	Coding	Pathway	-3.207	80.792		
Meconium Adj.[c]	VALSYN.PWY	Coding	Interaction	0.569	0.858		
Meconium Adj.[c]	VALSYN.PWY	Digit span	Acetaminophen	-3.107	7.099	0.628	0.885
Meconium Adj.[c]	VALSYN.PWY	Digit span	Pathway	41.804	68.922		
Meconium Adj.[c]	VALSYN.PWY	Digit span	Interaction	0.053	0.732		
Meconium Adj.[c]	VALSYN.PWY	Information	Acetaminophen	-2.548	6.775	0.741	0.907
Meconium Adj.[c]	VALSYN.PWY	Information	Pathway	-84.218	65.773		
Meconium Adj.[c]	VALSYN.PWY	Information	Interaction	0.789	0.698		
Meconium Adj.[c]	VALSYN.PWY	QTAC	Acetaminophen	-18.955	24.24	0.457	0.853
Meconium Adj.[c]	VALSYN.PWY	QTAC	Pathway	-108.754	235.336		
Meconium Adj.[c]	VALSYN.PWY	QTAC	Interaction	-4.291	2.498		
Meconium Adj.[c]	VALSYN.PWY	Vocabulary	Acetaminophen	-17.586	10.263	0.061	0.688
Meconium Adj.[c]	VALSYN.PWY	Vocabulary	Pathway	-175.284	99.64		
Meconium Adj.[c]	VALSYN.PWY	Vocabulary	Interaction	0.781	1.058		
Meconium Adj.[c]	VALSYN.PWY	WISC sum	Acetaminophen	-28.195	23.965	0.186	0.688
Meconium Adj.[c]	VALSYN.PWY	WISC sum	Pathway	-273.698	232.664		

Exposure Window	Pathway	Outcome	Variable	Effect Estimate[a]	Std. Error	LRT[b]	
						p-value	q-value
Meconium Adj.[c]	VALSYN.PWY	WISC sum	Interaction	2.257	2.47		

[a] Models are adjusted for whether the child was ever breastfed, sex, mode of birth, and socioeconomic status. Effect estimates for acetaminophen are the difference in outcome score between exposed and unexposed. Effect estimates for species are the change in outcome score for each 1% increase in species relative abundance. Effect estimates for the interaction are for the multiplicative interaction between acetaminophen and species relative abundance.
[b] Likelihood ratio test comparing to model without interaction
[c] Meconium Adj. includes cross-sectional exposure in the model
